# Intramolecular thermal stepwise [2 + 2] cycloadditions: investigation of a stereoselective synthesis of [n.2.0]-bicyclolactones†
†Electronic supplementary information (ESI) available. See DOI: 10.1039/c6ob01661h



**DOI:** 10.1039/c6ob01661h

**Published:** 2016-09-20

**Authors:** Adam Throup, Laurence H. Patterson, Helen M. Sheldrake

**Affiliations:** a Institute of Cancer Therapeutics , University of Bradford , Bradford , BD7 1DP , UK . Email: h.sheldrake@bradford.ac.uk

## Abstract

Fused cyclobutanes are found in a range of natural products and formation of these motifs in a straightforward and easy manner represents an interesting synthetic challenge. To this end we investigated an intramolecular variant of the thermal enamine [2 + 2] cyclisation, developing a diastereoselective intramolecular enamine [2 + 2] cyclisation furnishing δ lactone and lactam fused cyclobutenes in good yield and excellent diastereoselectivity.

## Abstract 2

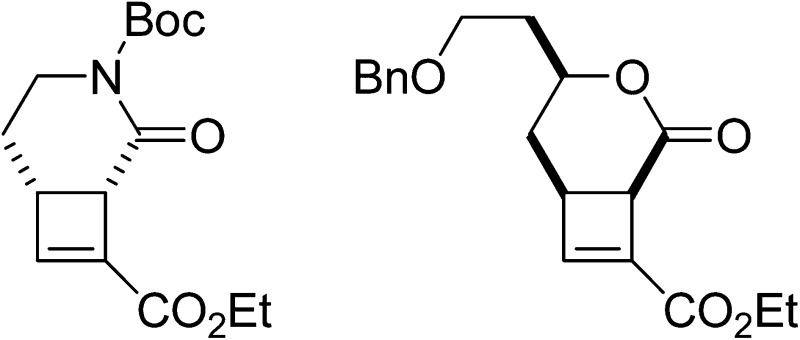
A new method for the synthesis of highly functionalised cyclobutenes.

## Introduction

**Fig. 1:**
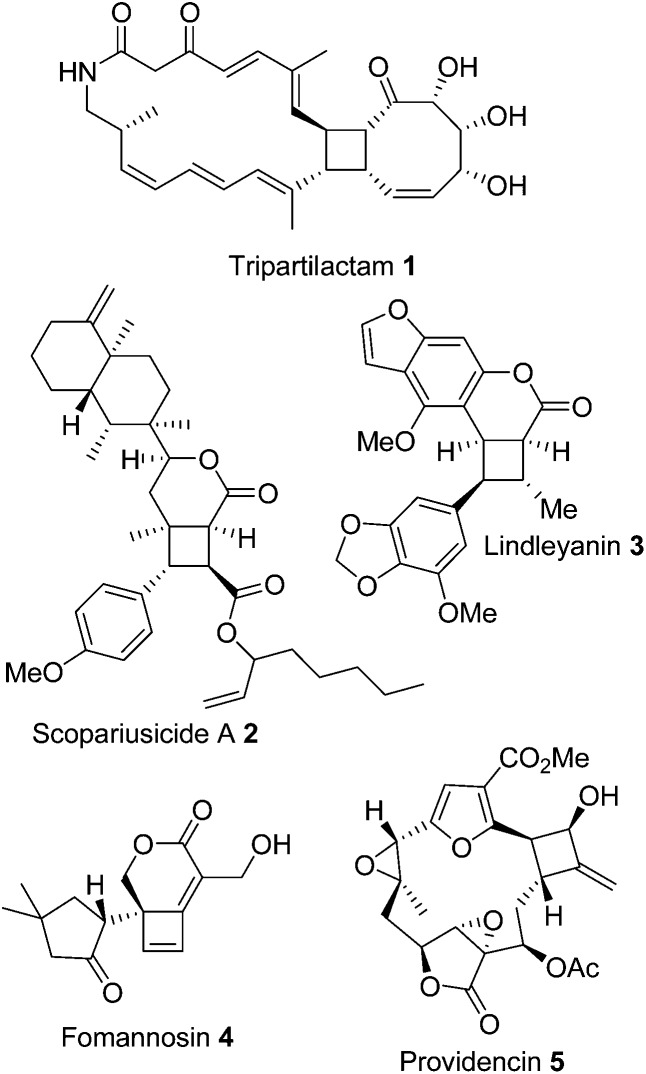
Examples of cyclobutane-containing complex natural product structures.

Cyclobutanes and cyclobutenes still provide a synthetic challenge^1–3^ particularly when fused to larger ring systems, a motif often found in natural products (Fig. 1).^4–8^ The photochemical [2 + 2] reactions traditionally used to access 4-membered rings can be problematic and unpredictable with a range of different products being produced; including dimers, regioisomers, and diastereomers.^9^ Several alternate methodologies have been developed to control the reaction outcome; for example, using photosensitising groups, or tuning the electronics of the system by using enol ethers and enones^10^ in combination (for a thorough review of recent developments in photochemical [2 + 2] cyclisations see Poplata *et al.* ^11^). Brannock has described a thermal enamine [2 + 2] cyclisation^12^ between an enamine and an electron deficient Michael acceptor alkene, which controls the regiochemistry of cyclisation and prevents homo-dimerization. The diastereoselectivity of this cyclisation is controlled by the reversibility of the Michael addition and subsequent cyclisation leads to the thermodynamic *trans* substituted cyclobutanes as the major product.^13^

Intramolecular variants of the photochemical [2 + 2] cyclisation have been developed and used in synthesis, but enamine [2 + 2] cyclisation has been underexploited to date and its full scope has not been explored. Intramolecular cycloaddition of ketenes or ketene-iminium salts with alkenes provides a useful route to [n.2.0] fused-cyclobutanones,^14–18^ however ketene cycloadditions provide a limited pattern of substituents on the cyclobutane ring and do not allow easy access to cyclobutenes. The development of an intramolecular enamine [2 + 2] cyclisation gives a complementary albeit contrasting route to functionalised fused cyclobutanes and cyclobutenes. The recent identification of the cyclobutene moiety as a suitable electrophile for targeted covalent modification of proteins^19^ suggests this functionality will have increasing importance in medicinal chemistry. The development of straightforward procedures for the synthesis of 4-membered rings leading to predictable product structures is therefore an important challenge.

## Results and discussion

To investigate the range of ring sizes accessible by the reaction, aldehyde tethered fumarates **7a–e** were synthesised from the corresponding diols (Table 1). The cyclisation precursors were treated with diethylamine in the presence of K_2_CO_3_ in acetonitrile to yield the intermediate cyclobutanes, which proved unstable and prone to hydrolysis so were not isolated. Treatment of the crude reaction mixture with MeI to give quaternary ammonium salts followed by Hoffmann elimination yielded the stable *cis*-fused cyclobutene (**9b**). The relative configuration of the ring junction was confirmed by nOe spectroscopy (see ESI†).

**Table 1:** Effect of tether length and heteroatom on the outcome of intramolecular enamine [2 + 2] cyclisation reactions

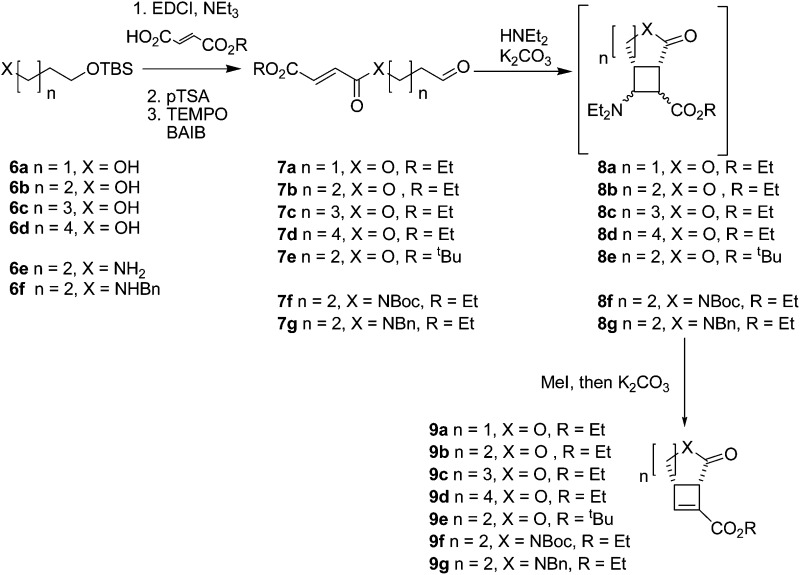			
Entry	Precursor	Product	Yield
1	**7a**	**9a**	0
2	**7b**	**9b**	43%
3	**7c**	**9c**	0
4	**7d**	**9d**	0
5	**7e**	**9e**	20%
7	**7f**	**9f**	35%
8	**7g**	**9g**	25%

Only the 6,4 fused lactones **9b** and **9e** were isolated. The other length tethers failed to cyclise; in each case most of the aldehyde was consumed but the desired compounds (**9a**, **9c**, **9d**) were not isolable and the products **9** or corresponding intermediates (**8a**, **8c**, **8d**) were not observable by ^1^H NMR of the crude reaction mixtures. This indicated failure of the cyclisation step.

**Fig. 2:**
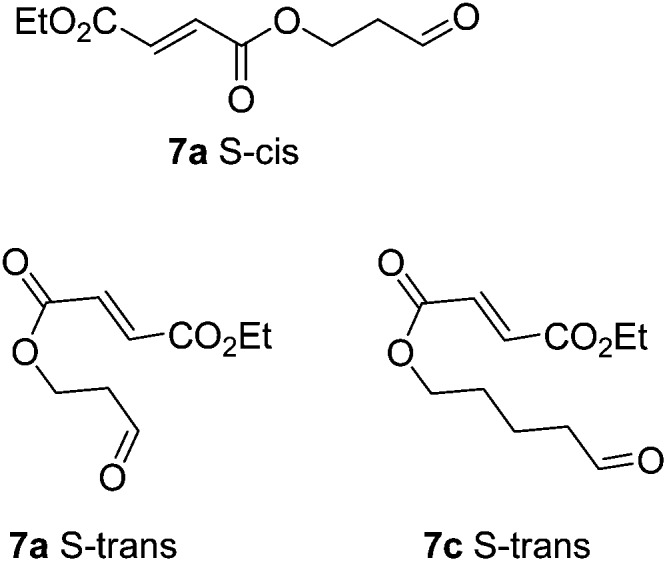
Non-reacting (S-*cis*) and reacting (S-*trans*) conformations of ester tethered cyclisation substrates.

Cyclisation of **7a** to form a 5,4 fused bicycle was probably prevented by the tether being too short to allow a favourable orientation of orbital overlap as the two reacting group approach one another; a conformation where the two halves of the molecule are close enough to allow cyclisation would place the π-system of the fumarate and aldehyde-derived enamine to be perpendicular to one another. Furthermore, Ghosh *et al.* ^20^ have shown that a similar ester tethered system did not undergo photochemical cyclisation since the preferred conformation (**7a** S-*cis* Fig. 2) holds the two ends of the chain at a distance from one another. Longer tethers (**7c,d**) will be affected by the entropic issues well-known for reducing efficiency of macrocycle formation. In this case, even the S-*trans* configuration does not bring the two ends of the molecule into sufficiently close proximity to allow a reaction. Small amounts of starting aldehyde were recovered from some reactions, along with complex mixtures of products also containing aldehyde groups (starting material was generally not recovered from successful reactions).

Attempts to optimise the reaction conditions were limited (Table 2) as it has already been established that this type of reaction tolerates a limited range of reagents and conditions; acetonitrile is required to stabilise the zwitterionic intermediates,^12,13^ (we have found that related reactions attempted in THF failed) and the presence of K_2_CO_3_ is required for cyclobutane formation.^13^ Decreasing the concentration of the reaction in an attempt to prevent polymerisation did not improve the yield of isolable cyclobutene products, which suggests polymerisation is not the major side reaction taking place. **7a** may decompose in the presence of base by elimination of the fumarate group to yield acrolein. Michael addition of diethylamine to the starting material would reversibly prevent productive reaction of any substrate. Diethylamine was the only amine investigated which gave a successful reaction. Cyclic amines (entries 13–15) caused complete decomposition of the reaction mixture; (*R*)-*N*,α-dimethylbenzylamine did not cause cyclobutane formation, or react with the fumarate group in **7b**. Chiral amine auxiliaries cannot be used to generate homochiral cyclobutanes by this method.

**Table 2:** Effect of concentration and base in cyclisation reactions

Entry	Precursor	Concentration	Amine	Yield
1	**7a**	0.045 M	Et_2_NH (1 eq.)	0
2	**7a**	0.045 M	Et_2_NH (2 eq.)	0
3	**7b**	0.050 M	Et_2_NH (1 eq.)	29 (**9b**)
4	**7b**	0.051 M	Et_2_NH (2 eq.)	37 (**9b**)
5	**7b**	0.066 M	Et_2_NH (2 eq.)	43 (**9b**)
6	**7b**	0.075 M	Et_2_NH (2 eq.)	41 (**9b**)
7	**7b**	0.034 M	Et_2_NH (10 eq.)	28 (**9b**)
8	**7e**	0.045 M	Et_2_NH (2 eq.)	20 (**9e**)
9	**7e**	0.097 M	Et_2_NH (2 eq.)	16 (**9e**)
10	**7c**	0.050 M	Et_2_NH (2 eq.)	0^*a*^
11	**7d**	0.032 M	Et_2_NH (2 eq.)	0^*b*^
12	**7d**	0.024 M	Et_2_NH (2 eq.)	0^*c*^
13	**7b**	0.050 M	Pyrrolidine (2 eq.)	0
14	**7b**	0.050 M	Morpholine (2 eq.)	0
15	**7b**	0.080 M	(*R*)-2-Methylpyrrolidine (2 eq.)	0
16	**7b**	0.080 M	(*R*)-*N*,α-Dimethylbenzylamine (2 eq.)	0

^*a*^10% aldehyde recovered.

^*b*^16% aldehyde recovered.

^*c*^35% aldehyde recovered.

Increasing the steric bulk in the substrate by switching from ethyl to ^*t*^butyl ester (**7e**) resulted in reduced yield of **9e** after increased reaction time (5 days compared to the standard 2 days for intramolecular reaction of ethyl esters).

After the successful preparation of the lactone fused cyclobutene, synthesis of lactam fused cyclobutenes was attempted. Lactam 6,4 fused cyclobutanes have previously been accessed by photochemistry,^21–23^ but the majority of work involves quinolone-based structures. Lactam 5,4 fused cyclobutanes are accessible through a wider range of methods, frequently relying on prior preparation of a 1,2-cyclobutane dicarboxylic acid or photocyclisation of a maleimide.^24–27^ Here, amide tethered aldehydes were synthesised containing a Boc protected **7f**, and benzyl protected **7g** amide (the corresponding unprotected aldehyde was not accessible due to side reactions during oxidation). When subjected to the cyclisation conditions both the Boc and benzyl protected amides cyclised forming lactam fused cyclobutenes **9f,g**, although in lower yields than their ester linked counterparts. The method therefore complements photochemical cyclisations, which tolerate the presence of an amide NH group.

### Diastereoselective intramolecular enamine [2 + 2] cyclisations

We hypothesised that the presence of a side chain within the tether would control the way the molecule would fold into a reactive conformation thus giving diastereocontrol. Using enantiopure starting materials would therefore allow the enantiospecific synthesis of fused cyclobutenes.

**Fig. 3:**
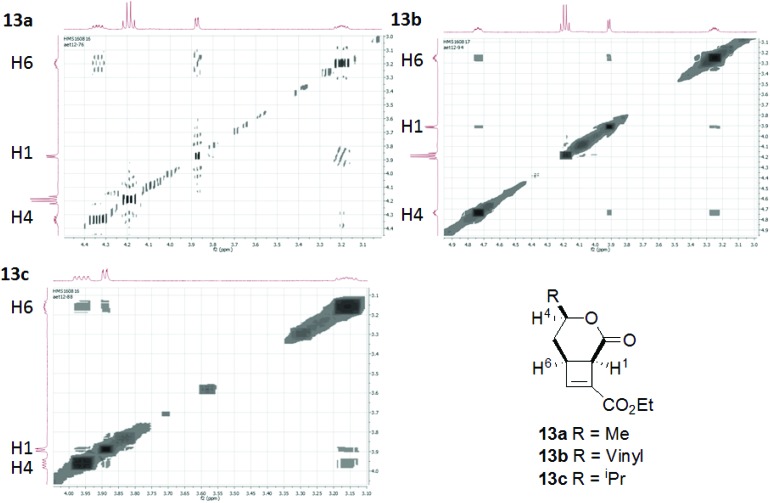
nOe correlations H1–H4–H6 of cyclobutenes **13a–c**.

Cyclisation substrates **12a–e** were prepared from a range of alcohols **10a–d** and amine **10e** by coupling with ethyl hydrogen fumarate, deprotection and oxidation to afford the required aldehydes. Cyclisation/eliminations proceeded in good to moderate yield over the 3 steps; all reactions yielded a single diastereomer (>95% de) (Table 3). This was shown to be the all *cis* compound by nOe spectroscopy; crosspeaks were observed between H-1 and H-6 indicating the expected *cis*-configuration at the ring junction, and between H-4 and H-6/H-1 indicating H-4 is on the same face of the molecule as the protons at the ring junction (*e.g.* Fig. 3).

**Table 3:** Intramolecular enamine [2 + 2] cyclisation reactions using a functionalised tether

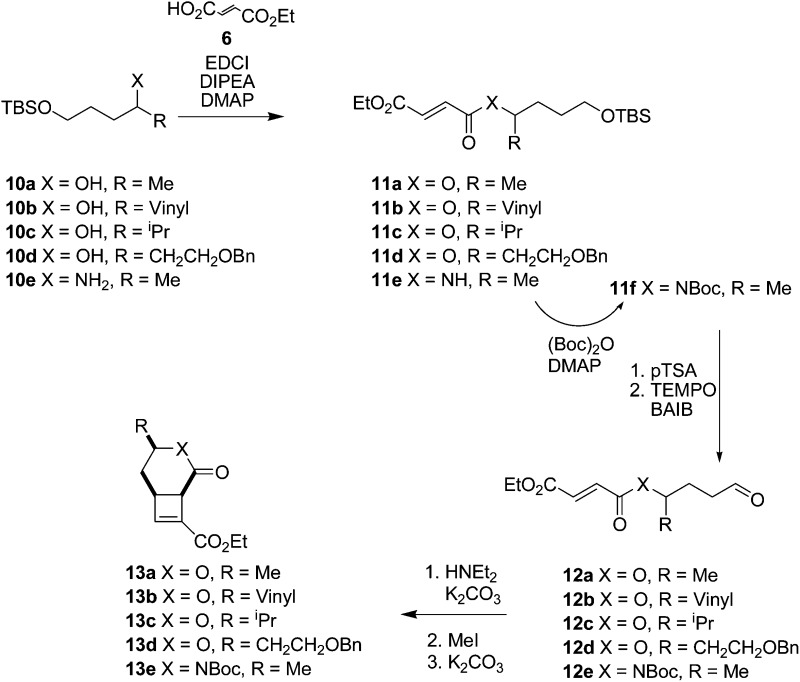				
Entry	Precursor	Product	Yield	de
1	**12a**	**13a**	54%	>95%
2	**12b**	**13b**	48%	>95%
3	**12c**	**13c**	47%	>95%
4	**12d**	**13d**	60%	>95%
5	**12e**	**13e**	30%	>95%

**Fig. 4:**
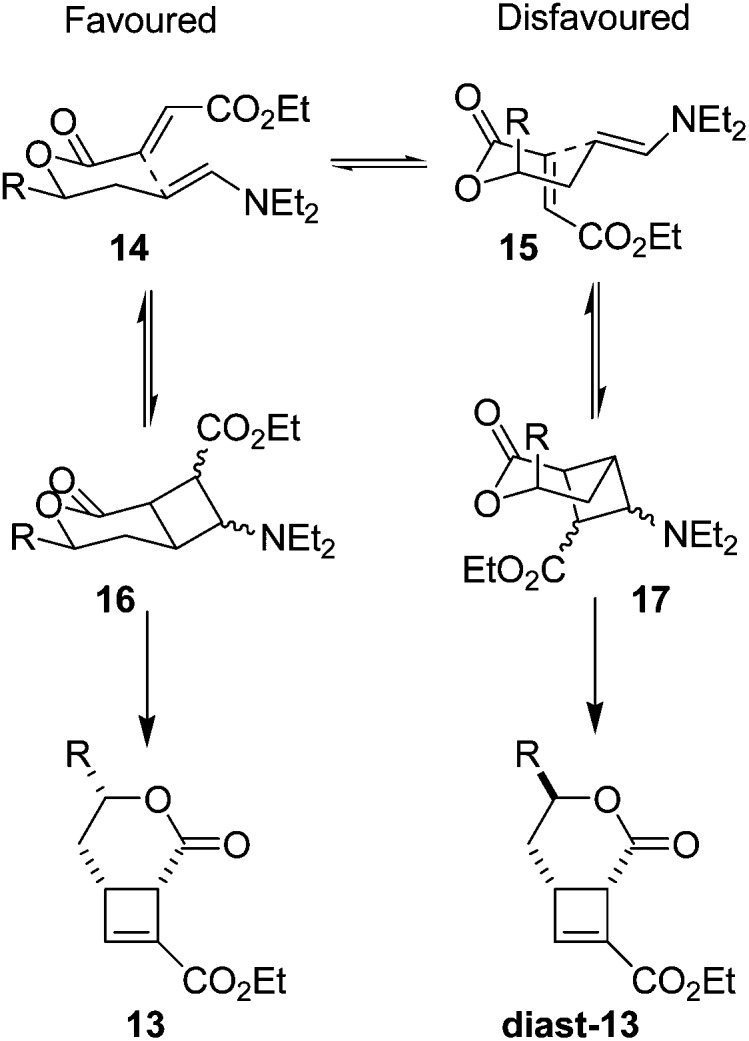
Proposed transition states for intramolecular cycloaddition. Pseudoequatorial arrangement of all sidechains during cyclisation leads to the observed product **13**.

The excellent diastereoselectivity, and the *cis* arrangement of groups around the 6-membered ring, is hypothesised to arise through a chair-like transition state **14** (Fig. 4) where the side chain ‘R’ in a pseudoequatorial position is favoured. This conformation allows efficient overlap of the two π systems to facilitate cyclisation. The alternative conformation **15** places the π systems near perpendicular to one another, making efficient overlap to form a *cis*-fused bicycle not possible.

**Fig. 5:**
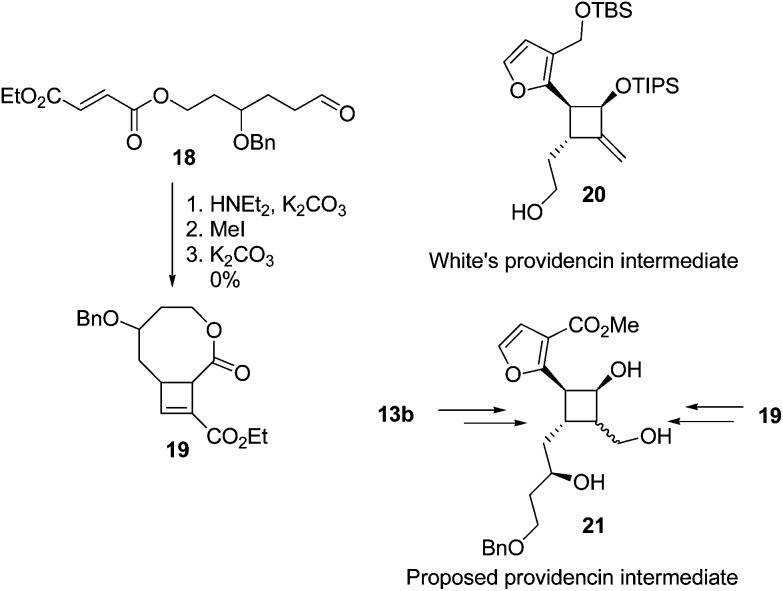
Attempted intramolecular enamine [2 + 2] cyclisation of a longer functionalised tether.

The yields of cyclobutenes **13** were generally higher than the yields of cyclobutenes **9** obtained from cyclising unsubstituted precursors, presumably due to the presence of the sidechain reducing the conformational flexibility of the substrate and favouring a reactive conformation. However, the increased yield did not generalise to the formation of larger ring systems (Fig. 5). Compound **19** (an isomer of **13d**) was of interest since both **19** and **13b** are suitable starting points for synthesis of the highly substituted cyclobutane ring system found in providencin **5**. Compound **21**, which is similar to known intermediates such as **20**,^28^ could be obtained from either **19** or **13b** by rhodium-catalysed addition^29^ of furan to the cyclobutene and further elaboration of the sidechains. Subjecting aldehyde **18** to the cyclisation conditions for up to 7 days returned unreacted starting material along with small amounts of decomposition products resulting from aldol condensations of **18**.

In conclusion, we have demonstrated that an intramolecular variant of the enamine [2 + 2] cyclisation allows straightforward access to 6,4 fused bicyclic systems in a diastereoselective manner. This methodology provides a new means to access functionalized fused cyclobutenes bearing sidechains that allow further synthetic elaboration. Applications of the methodology in drug discovery, and to the total synthesis of the biologically active natural product providencin, are in progress.

This work was funded by Yorkshire Cancer Research (Award reference number B002-PhD). We thank the EPSRC UK National Mass Spectrometry Facility at Swansea University for measurement of HRMS data.

### Example experimental procedure

#### (1S*,4S*,6S*)-Ethyl 4-methyl-2-oxo-3-oxabicyclo[4.2.0]oct-7-ene-8-carboxylate (**13a**)

Diethylamine (0.08 mL, 58 mg, 0.8 mmol) was added to a stirred solution of aldehyde **12a** (90 mg, 0.39 mmol) and K_2_CO_3_ (110 mg, 0.8 mmol) in MeCN (15 mL) and stirred at RT under a blanket of N_2_ for 65.5 hours. The reaction mixture was filtered through Celite® and concentrated *in vacuo*. The crude cyclobutane was dissolved in MeCN (15 mL) and MeI (0.12 mL, 277 mg, 1.95 mmol) was added and the reaction mixture stirred at RT under a blanket of N_2_ for 70 hours. The reaction mixture was concentrated *in vacuo* and the crude residue dissolved in MeCN (15 mL) and K_2_CO_3_ (110 mg, 0.8 mmol) was added and the reaction mixture heated at 65 °C for 24 hours. The reaction was cooled to RT and concentrated *in vacuo*. The crude residue was purified *via* column chromatography (1 : 1, EtOAc : PE) to give the title compound **13a** as a clear oil (45 mg, 0.21 mmol, 54% yield); *R*_f_ 0.22 (1 : 1, EtOAc : PE); IR 2984.7 cm^–1^ (CH), 1710.8 cm^–1^ (C=O); ^1^H NMR (400 MHz, CDCl_3_) *δ* 6.81 (s, 1H, H7), 4.40–4.28 (m, 1H, H4), 4.19 (q, *J* = 7.1 Hz, 2H, OC*H*_2_CH_3_), 3.88 (d, *J* = 4.8 Hz, 1H, H1), 3.27–3.14 (m, 1H, H6), 2.16 (ddd, *J* = 14.2, 7.8, 1.3 Hz, 1H, H5), 1.43 (app dt, *J* = 14.2, 10.7 Hz, 1H, H5′), 1.32 (d, *J* = 6.3 Hz, 3H, C*H*_3_), 1.25 (t, *J* = 7.1 Hz, 3H, OCH_2_C*H*_3_); ^13^C NMR (101 MHz, CDCl_3_) *δ* 169.9 (C), 161.2 (C), 148.7 (CH), 136.9 (C), 76.2 (CH), 60.8 (CH_2_), 42.2 (CH), 37.8 (CH), 35.4 (CH_2_), 21.0 (CH_3_), 14.2 (CH_3_); *m*/*z* (ES^+^) 233.1([M + Na]^+^, 100%) 211.1 ([M + H]^+^, 10%); HRMS found ([M + H]^+^) 211.0964. C_11_H_15_O_4_ req 211.0965; LCMS rt 2.71 minutes, *m*/*z* (ES^+^) 211.2 ([M + H]^+^, 100%), >95%.
