# Supplementary material for: Intramolecular thermal stepwise [2 + 2] cycloadditions: investigation of a stereoselective synthesis of [n.2.0]-bicyclolactones
Source: Org Biomol Chem. 2016 Sep 20;14(40):9554–9. doi: 10.1039/c6ob01661h (PMC5066561; doi:10.1039/c6ob01661h)
Supplement: Supplementary file 1 [file OB-140-C6OB01661H-s001.pdf]

Electronic Supplementary Material (ESI) for Organic & Biomolecular Chemistry.
This journal is © The Royal Society of Chemistry 2016

Intramolecular thermal stepwise [2+2] cycloadditions: investigation of a stereoselective synthesis of [n.2.0]-bicyclolactones

Adam Throup, Laurence H Patterson, Helen M Sheldrake*

Part I Synthetic methods

Contents

Materials and Instrumentation 1

Synthesis of Alcohols and Amines 2

Synthesis of Unbranched Alcohols and Amines (**6a**-**f**) 2

Branched Alcohols and Amine (**10a**-**e**) 4

Synthesis of Fumarates and Fumaramides 9

Synthesis of Unbranched Fumarates and Fumaramides 9

Synthesis of Branched Fumarates and Fumaramides 11

Boc Protection of Fumaramides 13

Synthesis of Aldehydes (**7a-g, 12a-e, 18**) 14

Synthesis of Cyclobutenes 18

Synthesis of Unsubstituted Cyclobutenes (**9a, e, f, g**) 18

Synthesis of Substituted Cyclobutenes (**13a-e**) 19

References 21

Part II NMR spectra of novel compounds and intermediates 22

Materials and Instrumentation

Commercially available reagents and solvents were purchased from Sigma-Aldrich®, Tokyo Chemistry Industry (TCI®), Alfa Aesar®, VWR® Fisher® and Merck®, and were used as received without additional purification.

Products were purified by column chromatography where stated using Merck 9385 silica gel 60 (40-63 µm). Analytical thin layer chromatography (TLC) was conducted on Merck silica gel 60 F_{254} aluminium backed plates. TLCs were visualised under short wavelength (254 nm) ultraviolet light and potassium permanganate ($KMnO_4$) stain, vanillin stain or ninhydrin stain.

Proton Nuclear Magnetic Resonance (^{1}H NMR) spectra were recorded using a Bruker AMX400 (400 MHz) spectrometer. Carbon Nuclear Magnetic Resonance (^{13}C) were performed on the same instrument operating at 101 MHz. Chemical shifts are reported in parts per million (δ, ppm). ^{1}H NMR chemical shifts

are reported relative to an internal reference (tetramethylsilane) or residual proton signals of the solvent. Multiplets are indicated as s, singlet; d, doublet; t, triplet; q, quartet; qn, quintet; m, multiplet; br broad; app apparent. Coupling constants (*J*) are expressed in Hertz (Hz). With the exception of sidechains and protecting groups, atoms have been identified using the numbering system in the systematic name of the compound (ie. H1 is the hydrogen atom or atoms bonded to C1 of the skeletal structure). ^{13}C NMR chemical shifts are reported relative to the signal of the solvent.
Routine mass spectra were carried out by the University of Bradford Centre for Chemical and Structural Analysis (Mr Andrew Healey) and were run on a Micromass Quattro Ultima spectrometer in the electron impact (EI), chemical ionisation (AP) or electrospray (ES) mode as stated. High resolution mass spectrometry (HRMS) was carried out by the EPSRC National Mass Spectrometry Facility, Swansea University.
Optical rotations where carried out on a Perkin Elmer polarimeter, model 341.
All moisture sensitive reactions were carried out under an inert atmosphere using dry argon or nitrogen.
LCMS analysis was performed on a Waters e2695 Separation module using a HICHROM column (3.5RPB, 15cm × 2.1mm), with a flow rate of 0.25 ml/min and mobile phase of water:MeOH:formic acid (5:5:0.1 at T=O mins,→1:9:0.1 at T=7mins, →5:5:0.1 at T=13 mins); eluting compounds were analysed by Waters 2998 PDA Detector (*uv* spectroscopy 210-400 nm) and QDA Detector (mass spectrometry).

Synthesis of Alcohols and Amines

Synthesis of Unbranched Alcohols and Amines (6a-f)

General procedure for protection of diols[1]

Diol (1.4 eq) was added to a stirred suspension of NaH (60% dispersion in oil, 1 eq) in THF at 0 °C under a blanket of N_2. The resultant mixture was stirred for 40 minutes, TBSCl (1 eq) was added and the reaction mixture stirred for a further 1 hour 45 minutes. The mixture was poured into saturated aqueous ammonium chloride solution and extracted with diethyl ether. The combined organic layers were dried ($MgSO_4$) and concentrated *in vacuo* to give the crude protected diols that were used without further purification.

3-((*tert*-butyldimethylsilyl)oxy)propan-1-ol (6a)

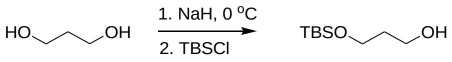

Clear oil (671 mg, 3.53 mmol, 71% yield); R_f 0.55 (1:1, EtOAc:PE); ^{1}H NMR (400 MHz, $CDCl_3$) δ 3.79 – 3.69 (m, 4H, H1+H3), 2.57 (br s, 1H, O*H*), 1.70 (qn *J* = 5.5 Hz, 2H, H2), 0.82 (s, 9H, ^{t}Bu), -0.00 (s, 6H, (C*H*$_3$)$_2$). Consistent with literature.[2]

4-((*tert*-butyldimethylsilyl)oxy)butan-1-ol (6b)

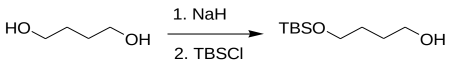

Clear oil (715 mg, 3.5 mmol, 100% yield); R_f 0.31 (2:8, EtOAc:PE); ^{1}H NMR (400 MHz, $CDCl_3$) δ 3.64 – 3.54 (m, 4H, H1+H4), 1.63 – 1.50 (m, 4H, H2+H3), 0.83 (s, 9H, ^{t}Bu), -0.00 (s, 6H, (C*H*$_3$)$_2$). Consistent with literature.[1]

5-((*tert*-butyldimethylsilyl)oxy)pentan-1-ol (6c)

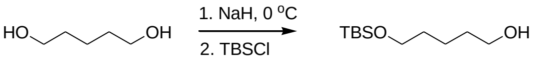

Clear oil (778 mg, 3.57 mmol, 71% yield); R_f 0.52 (1:1, EtOAc:PE); ^{1}H NMR (400 MHz, $CDCl_3$) δ 3.65 – 3.52 (m, 4H, H1+H5), 1.60 – 1.44 (m, 4H, H2+H4), 1.42 – 1.29 (m, 2H, H3), 0.84 (s, 9H, ^{t}Bu), 0.00 (s, 6H, $(CH_3)_2$). Consistent with literature.[3]

6-((*tert*-butyldimethylsilyl)oxy)hexan-1-ol (6d)

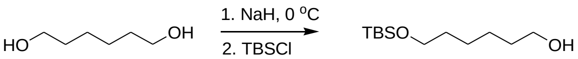

Crude product required purification *via* column chromatography (1:1, EtOAc:PE). Clear oil (515 mg, 2.22 mmol, 44% yield); R_f 0.48 (4:6, EtOAc:PE); ^{1}H NMR (400 MHz, $CDCl_3$) δ 3.63 – 3.51 (m, 4H, H1+H6), 1.59 – 1.42 (m, 4H, H2+H5), 1.37 – 1.28 (m, 4H, H3+H4), 0.85 (s, 9H, ^{t}Bu), 0.00 (s, 6H, $(CH_3)_2$). Consistent with literature.[4]

4-((*tert*-Butyldimethylsilyl)oxy)butan-1-amine (6e)

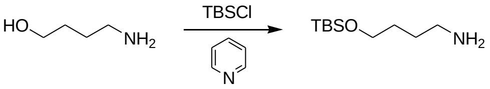

TBSCl (2.022 g, 13.48 mmol) was added to a stirred mixture of 4-aminobutan-1-ol (1.034 ml, 1 g, 11.23 mmol) and pyridine (2 ml, 1.956 g, 24.76 mmol) and stirred at RT for 16 hours. The reaction mixture was concentrated *in vacuo*, partitioned between DCM (15 ml) and saturated aqueous sodium bicarbonate solution (15 ml), dried ($MgSO_4$) and concentrated *in vacuo* to give the title compound **5e** as an orange oil (2.28 g, 11.23 mmol, 100% yield); R_f 0.14 (95:5, DCM:MeOH); ^{1}H NMR (400 MHz, $CDCl_3$) δ 3.58 (t, J = 5.6 Hz, 2H, H4), 2.75 (t, J = 6.4 Hz, 2H, H1), 1.58 – 1.46 (m, 4H, H2+H3), 0.84 (s, 9H, ^{t}Bu), 0.00 (s, 6H, $(CH_3)_2$). Consistent with literature.[5]

4-((*tert*-Butyldimethylsilyl)oxy)butanal

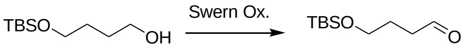

6b

DMSO (0.49 ml, 533 mg, 6.84 mmol) was added over 10 minutes to a stirred solution of oxalyl chloride (2 M in DCM, 2.05 ml, 4.10 mmol) in DCM (10 ml) at -42 °C under a blanket of N_2. 4-((*tert* butyldimethylsilyl)oxy)butan-1-ol **6b** (698 mg, 3.42 mmol) in DCM (4 ml) was added and the reaction stirred for a further 10 minutes. NEt_3 (1.04 ml, 549 mg, 7.52 mmol) was added and the reaction mixture stirred for 10 minutes, then allowed to warm to RT over 4 hours. The reaction was quenched with H_2O (30 ml), extracted with DCM (25 ml), dried ($MgSO_4$) and concentrated *in vacuo* to give the title compound as a clear oil (651 mg, 3.22 mmol, 94% yield); R_f 0.52 (2:8, EtOAc:PE); ^{1}H NMR (400 MHz, $CDCl_3$) δ 9.75 (t, J = 1.7 Hz, 1H, H1), 3.61 (t, J = 6.0 Hz, 2H, H4), 2.46 (td, J = 7.1, 1.7 Hz, 2H, H2), 1.87 – 1.72 (m, 2H, H3), 0.84 (s, 9H, ^{t}Bu), -0.00 (s, 6H, $(CH_3)_2$). Consistent with literature.[6]

_N_-Benzyl-4-((_tert_-butyldimethylsilyl)oxy)butan-1-amine (6f)

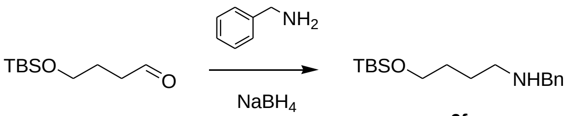

6f

Benzylamine (0.22 ml, 212 mg, 1.98 mmol) was added to a stirred solution of 4-((*tert*-butyldimethylsilyl)oxy)butanal (400 mg, 1.98 mmol) in MeOH (10 ml) under a blanket of argon and the reaction mixture stirred for 6.5 hours. The reaction mixture was then cooled to 0 °C and $NaBH_4$ (98 mg, 2.57 mmol) was added, the reaction warmed to RT and stirred for 17.5 hours. The reaction mixture was concentrated *in vacuo*, partitioned between EtOAc (20 ml) and saturated aqueous sodium bicarbonate (20 ml), dried ($MgSO_4$) and concentrated *in vacuo* to give the title compound **6f** as an white oil (576 mg, 1.98 mmol, 100% yield); ^{1}H NMR (400 MHz, $CDCl_3$) δ 7.28 (m, 5H, Ar), 3.75 (s, 2H, PhCH_2), 3.58 (dd, *J* = 7.9, 4.0 Hz, 2H, H4), 2.61 (t, *J* = 6.7 Hz, 2H, H1), 1.52 (dt, *J* = 6.3, 3.3 Hz, 4H, H2+H3), 0.85 (s, 9H, ^{t}Bu), -0.00 (s, 6H, (CH_3)$_2$). Consistent with literature.[7]

Branched Alcohols and Amine (10a-e)

<u>General Procedure for Addition to Aldehyde</u>

A solution of organo-lithium/Grignard reagent (1.3 eq) was added to a stirred solution of 4-((tert-butyldimethylsilyl)oxy)butanal (1 eq) in THF at -78 °C under a blanket of argon and stirred for 1-3 hour. The reaction was quenched with saturated aqueous ammonium chloride, extracted with EtOAc, washed with H_2O, dried ($MgSO_4$) and concentrated *in vacuo.* The crude residue was purified *via* column chromatography.

5-((_tert_-Butyldimethylsilyl)oxy)pentan-2-ol (10a)

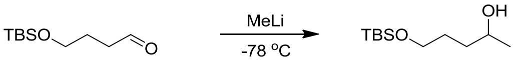

10a

Methyl lithium (1.6 M in THF, 10.98 ml, 17.56 mmol). Column chromatography (2:8, EtOAc:PE). Clear oil (2.01 g, 9.22 mmol, 68% yield); R_f 0.27 (2:8, EtOAc:PE); ^{1}H NMR (400 MHz, $CDCl_3$) δ 3.87 – 3.78 (m, 1H, H2), 3.73 – 3.62 (m, 2H, H5), 2.68 (s, 1H, O*H*), 1.70 – 1.56 (m, H3+H4), 1.20 (d, *J* = 6.3 Hz, 3H, H1), 0.91 (s, 9H, ^{t}Bu), 0.08 (s, 6H, (CH_3)$_2$). Consistent with literature.[8]

6-((_tert_-Butyldimethylsilyl)oxy)hex-1-en-3-ol (10b)

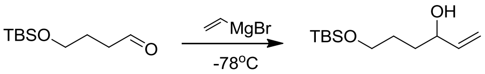

10b

Vinylmagnesium bromide (1 M in THF, 1.65 ml, 1.65 mmol). Crude product was used without further purification. Clear oil (250 mg, 1.09 mmol, 86% yield); R_f 0.51 (2:8, EtOAc:PE); ^{1}H NMR (400 MHz, $CDCl_3$) δ 5.81 (ddd, *J* = 17.1, 10.4, 5.8 Hz, 1H, H2), 5.17 (dt, *J* = 17.1, 1.4 Hz, 1H, H1), 5.03 (dt, *J* = 10.4, 1.4 Hz, 1H, H1'), 4.12 – 4.00 (m, 1H, H3), 3.60 (t, *J* = 5.6 Hz, 2H, H6), 2.53 (d, *J* = 4.2 Hz, 1H, O*H*), 1.62 – 1.52 (m, 4H, H4+H5), 0.83 (s, 9H, ^{t}Bu), -0.00 (s, 6H, (CH_3)$_2$). Consistent with literature.[9]

6-((_tert_-Butyldimethylsilyl)oxy)-2-methylhexan-3-ol (10c)

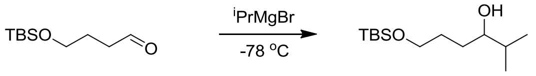

10c

iso-propylmagnesium bromide (2.9 M in THF, 1.34 ml, 3.9 mmol). Column chromatography (1:9, EtOAc:PE). Clear oil (529 mg, 2.15 mmol, 72% yield); R_f 0.46 (2:8, EtOAc:PE); ^{1}H NMR (400 MHz, $CDCl_3$) δ 3.68 (t, *J* = 4.7 Hz, 2H, H6), 3.41 – 3.31 (m, 1H, H3), 2.41 (d, *J* = 4.3 Hz, 1H, O*H*), 1.73 – 1.59 (m, 4H), 1.51 – 1.33 (m, 1H, H2), 0.93 (d, *J* = 3.9 Hz, 6H, C*H*$_3$),0.91 (s, 9H, ^{t}Bu), 0.07 (s, 6H, (C*H*$_3$)$_2$); ^{13}C NMR (101 MHz, $CDCl_3$) δ 76.4 (CH), 63.6 (CH_2), 33.6 (CH), 31.1 (CH_2), 29.4 (CH_2), 25.9 (CH_3), 18.7 (CH_3), 18.3 (C), 17.6 (CH_3), -5.4 (CH_3); *m/z* (AP+) 247.2 ([M+H]+, 100%); HRMS found ([M+H]+) 247.2089. $C_{13}H_{31}O_2Si$ req 247.2088.

Synthesis of Enantiopure alcohol 10d from (+)-Malic acid

(*R*)-Butane-1,2,4-triol[10]

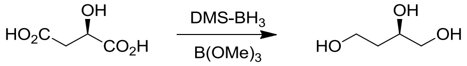

DMS·BH_3 (2 M in THF, 55.95 ml, 111.9 mmol) was added slowly to a stirred solution of malic acid (5 g, 37.31 mmol) and $B(OMe)_3$ (18.5 ml, 16.91 g, 164.16 mmol) in THF (50 ml) at 0 °C under a blanket of argon. The reaction mixture was stirred for 30 minutes before warming to RT and stirring for 5 days. The reaction was quenched with MeOH and the volatiles removed *in vacuo* to give the crude product that was used without further purification.

(*R*)-2-(2,2-Dimethyl-1,3-dioxolan-4-yl)ethanol

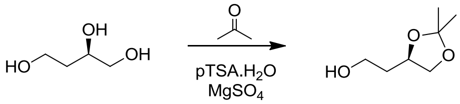

pTSA·H_2O (380 mg, 2 mmol) and $MgSO_4$ (2.28 g, 19 mmol) were added to a stirred solution of (*R*)-butane-1,2,4-triol (3.95 g, 37.31 mmol) in acetone (100 ml) and the reaction mixture heated at 56 °C under a blanket of argon for 44 hours. The reaction mixture was then cooled to RT, solid sodium bicarbonate (798 mg, 9.5 mmol) was added and the reaction mixture filtered through Celite® and concentrated *in vacuo*. The crude residue was purified *via* column chromatography (1:1, EtOAc:PE) to give the title compound as a clear oil (5.19 g, 35.55 mmol, 95% yield); R_f 0.22 (1:1, EtOAc:PE); $[\alpha]_D$ +3.6 (c 1.0 in $CHCl_3$) (lit.[10] $[\alpha]_D$ +3.1 (c 0.9 in $CHCl_3$)); ^{1}H NMR (400 MHz, $CDCl_3$) δ 4.31 – 4.21 (m, 1H), 4.08 (dd, *J* = 8.0, 6.1 Hz, 1H), 3.79 (dd, *J* = 10.2, 4.9 Hz, 2H), 3.58 (t, *J* = 7.6 Hz, 1H), 2.15 (t, *J* = 5.1 Hz, 1H), 1.81 (dd, *J* = 11.7, 6.2 Hz, 2H), 1.41 (s, 3H, C*H*$_3$), 1.35 (s, 3H, C*H*$_3$). Consistent with literature.[10]

(*R*)-4-(2-(Benzyloxy)ethyl)-2,2-dimethyl-1,3-dioxolane

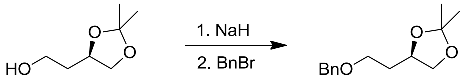

(*R*)-2-(2,2-dimethyl-1,3-dioxolan-4-yl)ethanol (3.31 g, 22.67 mmol) in DMF (10 ml) was added slowly to a stirred suspension of NaH (60% dispersion in oil, 1.08 g, 27.2 mmol) in DMF (40 ml) at RT under a blanket of N_2 and the reaction mixture stirred for 40 minutes. Benzyl bromide (3.23 ml, 4.65 g, 27.2 mmol) was added slowly while cooling and then stirred for 5 hours. The reaction mixture was quenched

with water, concentrated *in vacuo*, partitioned between EtOAc (80 ml) and water (80 ml), washed with brine (80 ml), dried ($MgSO_4$) and concentrated *in vacuo*. The crude residue was purified *via* column chromatography (1:9, EtOAc:PE) to give the title compound as a clear oil (3.09 g, 13.09 mmol, 58% yield); R_f 0.32 (1:9, EtOAc:PE); $[\alpha]_D$ -0.680 (c 1.0, $CHCl_3$) (lit.[11] $[\alpha]_D$ -0.60 (c 1.0, $CHCl_3$)); ^{1}H NMR (400 MHz, $CDCl_3$) δ 7.42 – 7.29 (m, 5H, Ar), 4.53 (s, 2H, PhCH_2), 4.29 – 4.19 (m, 1H), 4.09 (dd, *J* = 8.0, 5.9 Hz, 1H), 3.67 – 3.53 (m, 3H), 2.03 – 1.81 (m, 2H, CH_2CH_2), 1.42 (s, 3H, CH_3), 1.38 (s, 3H, CH_3). Consistent with literature.[12]

(*R*)-4-(Benzyloxy)butane-1,2-diol

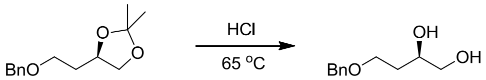

(*R*)-4-(2-(benzyloxy)ethyl)-2,2-dimethyl-1,3-dioxolane (3.09 g, 13.09 mmol) in THF (80 ml) and 2 M aqueous HCl (30 ml) was heated at 65 °C for 1 hour 25 minutes. The reaction mixture was allowed to cool to RT, diluted with EtOAc (150 ml), washed with brine (100 ml), dried ($MgSO_4$) and concentrated *in vacuo* to give the title compound as a yellow oil (2.46 g, 12.55 mmol, 96% yield); R_f 0.09 (1:1, EtOAc:PE); $[\alpha]_D$ -4.902 (c 1.0, $CHCl_3$) (lit.[11] $[\alpha]_D$ -4.2 (c 1.1, $CHCl_3$)); ^{1}H NMR (400 MHz, $CDCl_3$) δ 7.43 – 7.29 (m, 5H, Ar), 4.59 (s, 2H, PhCH_2), 4.09 (tt, *J* = 7.8, 4.0 Hz, 1H, H2), 3.86 (t, *J* = 5.5 Hz, 2H, H4), 3.53 (dd, *J* = 9.4, 3.6 Hz, 1H, H1), 3.43 (dd, *J* = 9.3, 7.6 Hz, 1H, H1'), 1.79 – 1.65 (m, 2H, H3). Consistent with literature.[13]

(*R*)-4-(Benzyloxy)-2-hydroxybutyl 4-methylbenzenesulfonate

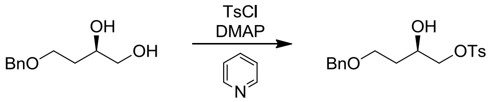

Tosyl chloride (2.38 g, 12.55 mmol) in $CHCl_3$ (10 ml) was added to a stirred solution of (*R*)-4-(benzyloxy)butane-1,2-diol (2.46 g, 12.55 mmol), pyridine (2.02 ml, 1.98 g, 25.1 mmol) and DMAP (159 mg, 1.3 mmol) in $CHCl_3$ (60 ml) at 0 °C under a blanket of argon and stirred for 70 hours. The reaction mixture was diluted with DCM (80 ml), washed with water (100 ml), dried ($MgSO_4$) and concentrated *in vacuo*. The crude residue was purified *via* column chromatography (4:6, EtOAc:PE) to give the title compound as a clear oil (2.20 g, 6.29 mmol, 50% yield); R_f 0.38 (1:1, EtOAc:PE); ^{1}H NMR (400 MHz, $CDCl_3$) δ 7.81 (d, *J* = 8.3 Hz, 2H, Ar), 7.39 – 7.29 (m, 7H, Ar), 4.51 (s, 2H, PhCH_2), 4.10 – 4.05 (m, 1H, H2), 4.05 – 3.95 (m, *J* = 16.1, 9.8, 5.2 Hz, 2H, H4), 3.73 – 3.60 (m, 2H, H1), 2.47 (s, 3H, PhCH_3), 1.80 (ddd, *J* = 11.9, 7.5, 4.6 Hz, 2H, H3). Consistent with literature.[14]

(*R*)-2-(2-(Benzyloxy)ethyl)oxirane

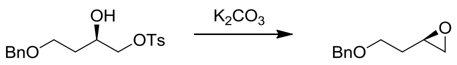

K_2CO_3 (1.74 g, 12.58 mmol) was added to a stirred solution of (*R*)-4-(benzyloxy)-2-hydroxybutyl 4-methylbenzenesulfonate (2.2 g, 6.29 mmol) in MeOH (70 ml) at RT and stirred for 2 hours. The reaction mixture was concentrated *in vacuo*, partitioned between diethyl ether (50 ml) and water (70 ml), the aqueous layer extracted with diethyl ether (2 × 50 ml), dried ($MgSO_4$) and concentrated *in vacuo* to give the title compound as a clear oil (1.097 g, 6.16 mmol, 98% yield); R_f 0.50 (1:1, EtOAc:PE); $[\alpha]_D$ +13.6 (c 1.0 in CH_3Cl) (lit.[15] $[\alpha]_D$ +14.5 (c 1.1 in CH_3Cl)); ^{1}H NMR (400 MHz, $CDCl_3$) δ 7.34 – 7.19 (m, 5H, Ar), 4.46 (s,

2H, PhC*H*$_2$), 3.60 – 3.49 (m, 2H, H2), 3.05 – 2.93 (m, 1H), 2.72 (t, *J* = 4.2 Hz, 1H), 2.45 (dd, *J* = 5.0, 2.8 Hz, 1H), 1.94 – 1.78 (m, 1H, H1), 1.80 – 1.62 (m, 1H, H1'). Consistent with literature.[15]

(*S*)-1-(Benzyloxy)hex-5-en-3-ol

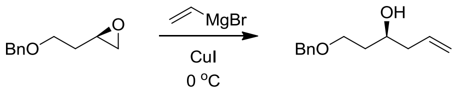

Vinylmagnesium bromide (1 M in THF, 9 ml, 9 mmol) was added to a stirred suspension of CuI (120 mg, 0.63 mmol) in THF (30 ml) at 0 °C under a blanket of argon and stirred for 5 minutes. (*R*)-2-(2-(benzyloxy)ethyl)oxirane (1.097 g, 6.16 mmol) in THF (10 ml) was added and stirred at 0 °C for 4 hours. The reaction mixture was partitioned between EtOAc (40 ml) and saturated aqueous ammonium chloride solution (40 ml), extracted with EtOAc (40 ml) and the combined organic layers dried ($MgSO_4$) and concentrated *in vacuo*. The crude residue was purified *via* column chromatography (3:7, EtOAc:PE) to give the title compound as a clear oil (909 mg, 4.43 mmol, 72% yield); R_f 0.42 (3:7, EtOAc:PE); $[\alpha]_D$ -4.998 (c 1.0, $CHCl_3$) (lit.[16] $[\alpha]_D$ -5.3 (c 1.2, $CHCl_3$)); ^{1}H NMR (400 MHz, $CDCl_3$) δ 7.40 – 7.29 (m, 5H, Ar), 5.86 (ddt, *J* = 17.3, 10.3, 7.1 Hz, 1H, H5), 5.17 – 5.09 (m, 2H, H6), 4.55 (s, 2H, PhC*H*$_2$), 3.94 – 3.86 (m, 1H, H3), 3.78 – 3.71 (m, 1H, H1), 3.71 – 3.64 (m, 1H, H1'), 2.84 (d, *J* = 2.9 Hz, 1H, O*H*), 2.27 (app t, *J* = 6.7 Hz, 2H, H4), 1.83 – 1.78 (m, 2H, H2). Consistent with literature.[17]

(*S*)-6-(Benzyloxy)hexane-1,4-diol

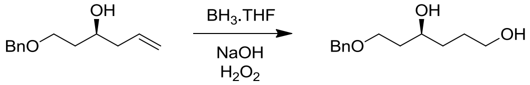

BH_3·THF (1 M in THF, 0.51 ml, 0.51 mmol) was added to a stirred solution of (*S*)-1-(benzyloxy)hex-5-en-3-ol (70 mg, 0.34 mmol) in THF (5 ml) at 0 °C under a blanket of N_2 and stirred for 2 hours 50 minutes. 1 M NaOH (0.68 ml, 0.68 mmol) and 30% aqueous H_2O_2 (0.25 ml) was added and the reaction mixture stirred for 17 hours. Saturated potassium tartrate (15 ml) and water (15 ml) was added and then extracted with DCM (2 × 30 ml), the combined organic layers were dried ($MgSO_4$) and concentrated *in vacuo*. The crude residue was purified *via* column chromatography (3:1, EtOAc:PE) to give the title compound as a white solid (36 mg, 0.16 mmol, 47% yield); R_f 0.20 (3:1, EtOAc:PE); $[\alpha]_D$ -0.729 (c 0.365, $CHCl_3$), (lit.[18] $[\alpha]_D$ -8.98 (c 3.4, $CHCl_3$)); ^{1}H NMR (400 MHz, $CDCl_3$) δ 7.33 – 7.19 (m, 5H, Ar), 4.46 (s, 2H, PhC*H*$_2$), 3.87 – 3.75 (m, 1H, H4), 3.68 (dt, *J* = 9.6, 4.8 Hz, 1H), 3.60 (app dt, *J* = 9.0, 5.3 Hz, 3H), 1.85 – 1.69 (m, 1H), 1.69 – 1.58 (m, 3H), 1.57 – 1.42 (m, 2H). Consistent with literature.[18]

(*S*)-1-(Benzyloxy)-6-((*tert*-butyldimethylsilyl)oxy)hexan-3-ol (10d)

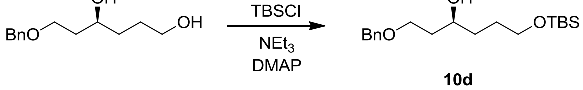

10d

TBSCl (512 mg, 3.41 mmol) was added to a stirred solution of (*S*)-6-(benzyloxy)hexane-1,4-diol (760 mg, 3.39 mmol), NEt_3 (0.48 ml, 344 mg, 3.41 mmol) and DMAP (49 mg, 0.4 mmol) in THF (30 ml) and the reaction mixture was stirred for 24 hours at RT under a blanket of N_2. Further TBSCl (512 mg, 3.41 mmol), NEt_3 (0.48 ml, 344 mg, 3.41 mmol) and DMAP (49 mg, 0.4 mmol) and the reaction heated at 65 °C for 24 hours. The reaction was cooled to RT, partitioned between saturated aqueous ammonium chloride solution (50 ml) and EtOAc (50 ml), dried ($MgSO_4$) and concentrated *in vacuo*. The crude residue

was purified *via* column chromatography (2:8, EtOAc:PE) to give the title compound **10d** as a clear oil (1.146 g, 3.39 mmol, quantitative yield); R_f 0.30 (2:8, EtOAc:PE); $[\alpha]_D$ -0.116 (c 1.10, $CHCl_3$); ^{1}H NMR (400 MHz, $CDCl_3$) δ 7.40 – 7.27 (m, 5H, Ar), 4.53 (s, 2H, PhC*H*$_2$), 3.87 – 3.79 (m, 1H, H3), 3.76 – 3.68 (m, 1H), 3.69 – 3.62 (m, 3H), 3.33 (d, *J* = 3.1 Hz, 1H, O*H*), 1.82 – 1.71 (m, 2H), 1.70 – 1.43 (m, 4H), 0.90 (s, 9H, ^{t}Bu), 0.06 (s, 6H, (C*H*$_3$)$_2$); ^{13}C NMR (101 MHz, $CDCl_3$) δ 138.1 (C), 128.5 (CH), 127.7 (2CH), 73.3 (CH_2), 70.7 (CH), 69.0 (CH_2), 63.4 (CH_2), 36.6 (CH_2), 34.4 (CH_2), 29.1 (CH_2), 26.0 (CH_3), 18.4 (C), -5.3 (CH_3); *m/z* (AP+) 339.4 ([M+H]+, 50%), 114.9 (100%); HRMS found ([M+H]+) 339.2351. $C_{19}H_{35}O_3Si$ req 339.2350.

5-((*tert*-Butyldimethylsilyl)oxy)pentan-2-yl methanesulfonate

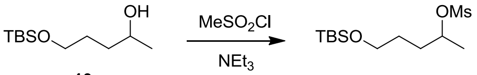

10a

NEt_3 (1.93 ml, 1.40 g, 13.83 mmol) was added to s attired solution of 5-((*tert*-butyldimethylsilyl)oxy)pentan-2-ol **10a** (2.01 g, 9.22 mmol) in DCM (100 ml) at 0 °C and stirred for 10 minutes. $MeSO_2Cl$ (0.85 ml, 1.26 g, 11.06 mmol) was added and the reaction mixture stirred for 5 hours. The reaction mixture was then washed with saturated aqueous sodium bicarbonate solution (100 ml), dried ($MgSO_4$) and concentrated *in vacuo* to give the title compound as a clear oil which was used immediately due to high reactivity (2.73 g, 9.22 mmol, quantitative); R_f 0.44 (2:8, EtOAc:PE).

((4-Azidopentyl)oxy)(tert-butyl)dimethylsilane

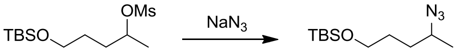

Sodium azide (408 mg, 6.28 mmol) was added to a stirred solution of 5-((*tert*-butyldimethylsilyl)oxy)pentan-2-yl methanesulfonate (929 mg, 3.14 mmol) in DMF (30 ml) at RT under a blanket of argon and the reaction mixture stirred for 23 hours. The reaction mixture was concentrated *in vacuo*, partitioned between EtOAc (40 ml) and saturated aqueous sodium bicarbonate solution (60 ml), extracted with EtOAc (50 ml), the combined organic layers washed with brine (60 ml), dried ($MgSO_4$) and concentrated *in vacuo*. The crude residue was purified *via* column chromatography (1:1, EtOAc:PE) to give the title compound as a yellow oil (620 mg, 2.55 mmol, 81% yield); ^{1}H NMR (400 MHz, $CDCl_3$) δ 3.57 (dd, *J* = 7.6, 3.5 Hz, 2H, H1), 3.42 – 3.40 (m, 1H, H4), 1.62 – 1.47 (m, 4H, H2+H3), 1.21 (d, *J* = 6.4 Hz, 3H, H5), 0.84 (s, 9H, ^{t}Bu), 0.00 (s, 6H, (C*H*$_2$)$_2$). Consistent with literature.[19]

5-((*tert*-Butyldimethylsilyl)oxy)pentan-2-amine (10e)

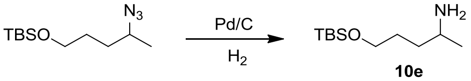

10e

10% Pd/C (160 mg) was added to a stirred solution of ((4-azidopentyl)oxy)(tert-butyl)dimethylsilane (1.595 g, 6.56 mmol) and NEt_3 (2 drops) in MeOH (60 ml) and stirred at RT under 1 atm H_2 for 21.5 hours. The reaction mixture was filtered through Celite® and concentrated *in vacuo* to give the title compound **10e** as a clear oil (1.423 g, 6.56 mmol, 100% yield); ^{1}H NMR (400 MHz, $CDCl_3$) δ 3.57 (t, *J* = 6.5 Hz, 2H, H5), 2.90 – 2.78 (m, 1H, H2), 1.58 – 1.40 (m, 2H), 1.39 – 1.22 (m, 2H), 1.02 (d, *J* = 6.4 Hz, 3H, H1), 0.84 (s, 9H, ^{t}Bu), -0.00 (s, 6H, (C*H*$_2$)$_2$). Consistent with literature.[20]

Synthesis of Fumarates and Fumaramides

General procedure for coupling with fumarate

EDCI (1.25 eq) was added to a stirred solution of ethyl or ^{t}butyl hydrogen fumarate (1.1 eq), alcohol/amine **6a**-**f** (1 eq), DIPEA (1.25 eq) and DMAP (0.1 eq) in DCM under a blanket of N_2 and the reaction mixture stirred for 24 hours. The reaction mixture was washed with 5% aqueous HCl and saturated aqueous sodium bicarbonate, dried ($MgSO_4$) and concentrated *in vacuo* to give the crude material (that was purified *via* column chromatography to give the fumarates).

Synthesis of Unbranched Fumarates and Fumaramides

3-((*tert*-Butyldimethylsilyl)oxy)propyl ethyl fumarate

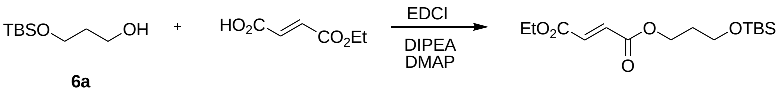

6a

Crude material was used without purification. Orange oil (941 mg, 2.98 mmol, 84% yield); R_f 0.70 (1:1, EtOAc:PE); ^{1}H NMR (400 MHz, $CDCl_3$) δ 6.80 (s, 2H, fumarate), 4.26 (t, *J* = 5.9 Hz, 2H, H1), 4.22 (q, *J* = 6.7 Hz, 2H, OC*H*$_2$CH$_3$), 3.66 (t, *J* = 6.0 Hz, 2H, H3), 1.91 – 1.77 (m, 2H, H2), 1.28 (t, *J* = 7.1 Hz, 3H, OCH$_2$C*H*$_3$), 0.84 (s, 9H, ^{t}Bu), 0.00 (s, 6H, (C*H*$_3$)$_2$); ^{13}C NMR (101 MHz, $CDCl_3$) δ 165.02 (C), 133.68 (CH), 133.55 (CH), 62.37 (CH$_2$), 61.36 (CH$_2$), 59.21 (CH$_2$), 30.95 (CH$_2$), 29.49 (C), 25.88 (CH$_3$), 14.11 (CH$_3$), -5.42 (CH$_3$); *m/z* (AP+) 317.0 ([M+H]+, 100%); HRMS found ([M+H]+) 317.1782. $C_{15}H_{29}O_5Si$ req 317.1779.

4-((*tert*-Butyldimethylsilyl)oxy)butyl ethyl fumarate

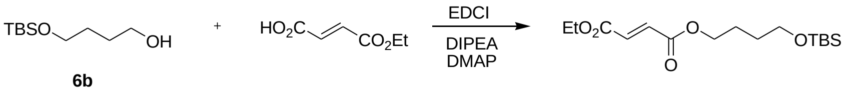

6b

Chromatography (1:9, EtOAc:PE). Yellow oil (844 mg, 2.56 mmol, 73% yield); R_f 0.55 (2:8, EtOAc:PE); ^{1}H NMR (400 MHz, $CDCl_3$) δ 6.80 (s, 2H, fumarate), 4.21 (dd, *J* = 12.8, 5.7 Hz, 2H, OC*H*$_2$CH$_3$), 4.17 (t, *J* = 5.2 Hz, 2H, H1), 3.59 (t, *J* = 6.2 Hz, 2H, H4), 1.75 – 1.65 (m, 2H, H2), 1.59 – 1.48 (m, 2H, H3), 1.27 (t, *J* = 7.1 Hz, 3H, OCH$_2$C*H*$_3$), 0.84 (s, 9H, ^{t}Bu), -0.00 (s, 6H, (C*H*$_3$)$_2$); ^{13}C NMR (101 MHz, $CDCl_3$) δ 165.1 (C), 165.0 (C), 133.6 (CH), 133.6 (CH), 65.3 (CH$_2$), 62.5 (CH$_2$), 61.3 (CH$_2$), 29.1 (CH$_2$), 25.9 (CH$_3$), 25.2 (CH$_2$), 18.3 (C), 14.1 (CH$_3$), -5.3 (CH$_3$); *m/z* (ES+) 331.2 ([M+H]+, 100%); HRMS found 331.1941. $C_{16}H_{31}O_5Si$ req. 331.1935.

5-((*tert*-Butyldimethylsilyl)oxy)pentyl ethyl fumarate

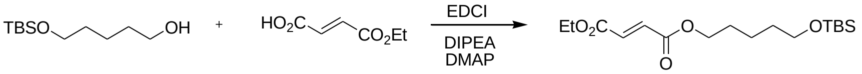

6c

Crude material was used without purification. Orange oil (1.068 g, 3.1 mmol, 87% yield); R_f 0.70 (1:1, EtOAc:PE); ^{1}H NMR (400 MHz, $CDCl_3$) δ 6.80 (s, 2H, fumarate), 4.21 (q, *J* = 7.1 Hz, 2H, OC*H*$_2$CH$_3$), 4.15 (t, *J* = 6.6 Hz, 2H, H1), 3.57 (t, *J* = 6.3 Hz, 2H, H5), 1.74 – 1.60 (m, 2H), 1.57 – 1.44 (m, 2H), 1.45 – 1.33 (m, 2H), 1.28 (t, *J* = 7.1 Hz, 3H, OCH$_2$C*H*$_3$), 0.84 (s, 9H, ^{t}Bu), 0.00 (s, 6H, (C*H*$_3$)$_2$); ^{13}C NMR (101 MHz, $CDCl_3$) δ 165.09 (C), 164.67 (C), 133.63 (CH), 65.40 (CH$_2$), 62.84 (CH$_2$), 61.34 (CH$_2$), 32.32 (CH$_2$), 28.30 (CH$_2$), 25.95 (CH$_2$),

22.25 (CH_3), 18.35 (C), 14.12 (CH_3), -5.29 (CH_3); *m/z* (AP+) 345.0 ([M+H]+, 100%); HRMS found ([M+H]+) 345.2095. $C_{17}H_{33}O_5Si$ req 345.2092.

6-((*tert*-Butyldimethylsilyl)oxy)hexyl ethyl fumarate

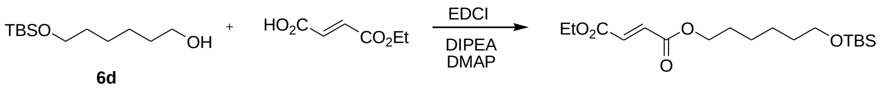

Chromatography (2:8, EtOAc:PE). Clear oil (429 mg, 1.2 mmol, 54% yield); R_f 0.60 (3:7, EtOAc:PE); ^{1}H NMR (400 MHz, $CDCl_3$) δ 6.80 (s, 2H, fumarate), 4.22 (q, *J* = 7.2 Hz, 2H, OCH_2CH_3), 4.15 (t, *J* = 6.6 Hz, 2H, H1), 3.56 (t, *J* = 6.4 Hz, 2H, H6), 1.69 – 1.58 (m, 2H, H3), 1.52 – 1.42 (m, 2H, H5), 1.36 – 1.30 (m, 4H, H3+H4), 1.28 (t, *J* = 7.1 Hz, 3H, OCH_2CH_3), 0.85 (s, 9H, ^{t}Bu), 0.00 (s, 6H, $(CH_3)_2$); ^{13}C NMR (101 MHz, $CDCl_3$) δ 165.31 (C), 165.24 (C), 133.86 (CH), 133.84 (CH), 65.64 (CH_2), 63.26 (CH_2), 61.55 (CH_2), 32.89 (CH_2), 28.75 (CH_2), 26.19 (CH_3), 25.93 (CH_2), 25.70 (CH_2), 18.58 (C), 14.33 (CH_3), -5.06 (CH_3); *m/z* (ES+) 359.2 ([M+H]+, 100%); HRMS found ([M+H]+) 359.2251. $C_{18}H_{35}O_5Si$ req 359.2248.

***tert*-butyl (4-((tert-butyldimethylsilyl)oxy)butyl) fumarate**

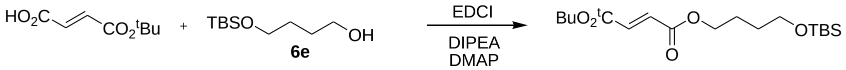

Chromatography (0:1→1:9, EtOAc:PE). Clear oil (1.914 g, 5.35 mmol, 59% yield); R_f 0.44 (1:9, EtOAc:PE); ^{1}H NMR (400 MHz, $CDCl_3$) δ 6.76 (m, 2H, fumarate), 4.21 (t, *J* = 6.6 Hz, 2H, H1), 3.64 (t, *J* = 6.2 Hz, 2H, H4), 1.79 – 1.71 (m, 2H, H2), 1.62 – 1.50 (m, 2H, H3), 1.45 (s, 9H, ^{t}Bu), 0.89 (s, 9H, ^{t}Bu), -0.05 (s, 6H, $(CH_3)_2$); ^{13}C NMR (101 MHz, $CDCl_3$) δ 165.29 (C), 164.20 (C), 135.60 (CH), 132.62 (CH), 81.89 (C), 65.21 (CH_2), 62.50 (CH_2), 29.14 (CH_2), 27.98 (CH_3), 25.94 (CH_3), 25.17 (CH_2), 18.32 (C), -5.33 (CH_3); *m/z* (ES+) 381.4 ([M+Na]+, 40%), 359.4 ([M+H]+, 20%), 325.4 (100%); HRMS found 245.1379 [M-TBS]+. $C_{12}H_{21}O_5$ req 245.1384.

(*E*)-Ethyl 4-((4-((*tert*-butyldimethylsilyl)oxy)butyl)amino)-4-oxobut-2-enoate

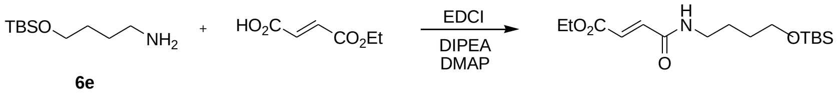

Column chromatography (2:8, EtOAc:PE). Yellow oil (1.38 g, 4.19 mmol, 38% yield); R_f 0.51 (1:1, EtOAc:PE); ^{1}H NMR (400 MHz, $CDCl_3$) δ 6.79 (d *J* = 15.4 Hz, 1H, fumarate), 6.77 (d, *J* = 15.4 Hz, 1H, fumarate), 4.18 (q, *J* = 7.1 Hz, 2H, OCH_2CH_3), 3.59 (t, *J* = 5.7 Hz, 2H, H1), 3.33 (q, *J* = 6.5 Hz, 2H, H4), 1.65 – 1.44 (m, 4H, H2+H3), 1.24 (t, *J* = 7.1 Hz, 3H, OCH_2CH_3), 0.83 (s, 9H, ^{t}Bu), 0.00 (s, 6H, $(CH_3)_2$); ^{13}C NMR (101 MHz, $CDCl_3$) δ 165.59 (C), 163.49 (C), 136.37 (CH), 130.28 (CH), 62.72 (CH_2), 61.12 (CH_2), 39.67 (CH_2), 30.06 (CH_2), 26.06 (CH_2), 25.96 (CH_3), 18.35 (C), 14.15 (CH_3), -5.30 (CH_3); *m/z* (ES+) 330.5 ([M+H]+, 100%); HRMS found ([M+H]+) 330.2098. $C_{16}H_{32}O_4NSi$ req. 330.2095.

(*E*)-Ethyl 4-(benzyl(4-((tert-butyldimethylsilyl)oxy)butyl)amino)-4-oxobut-2-enoate

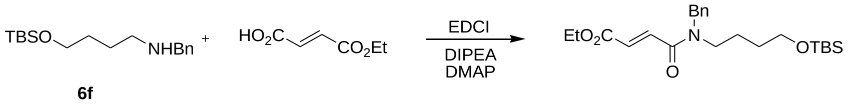

Column chromatography (2:8, EtOAc:PE). Clear oil (536 mg, 1.29 mmol, 65% yield) comprising a ~1:1 mixture of rotamers; R_f 0.43 (3:7, EtOAc:PE); ^{1}H NMR (400 MHz, $CDCl_3$) δ 7.40 (d, *J* = 15.2, 0.5H, fumarate), 7.37 – 7.15 (m, 6.5H, Ar), 6.89 (d, *J* = 15.2 Hz, 0.5H, fumarate), 6.86 (d, *J* = 15.2 Hz, 0.5H, fumarate), 4.68 (s, 1H, PhC*H*$_2$), 4.62 (s, 1H, PhC*H*$_2$), 4.26 (q, *J* = 7.1, 1H, OC*H*$_2$CH$_3$), 4.21 (q, *J* = 7.1, 1H, OC*H*$_2$CH$_3$), 3.59 (t, *J* = 6.0 Hz, 1H, H4), 3.58 (t, *J* = 6.0 Hz, 1H, H4), 3.44 (t, *J* = 7.5 Hz, 1H, H1), 3.35 (t, *J* = 7.5 Hz, 1H, H1), 1.65 – 1.56 (m, 2H, H2), 1.51 – 1.40 (m, 2H, H3), 1.32 (t, *J* = 7.2 Hz, 1.5H, OCH$_2$C*H*$_3$), 1.26 (t, *J* = 7.2 Hz, 1.5H, OCH$_2$C*H*$_3$), 0.87 (2s, 9H, ^{t}Bu), 0.03 (s, 3H, C*H*$_3$), 0.02 (s, 3H, (C*H*$_3$)$_2$); ^{13}C NMR (101 MHz, $CDCl_3$) δ 165.75 (C), 165,68 (C), 165.04 (C), 164.79 (C), 147.66 (CH), 137.04 (CH), 136.40 (CH), 134.10 (C), 133.72 (C), 131.80 (CH), 131.66 (CH), 128.98 (CH), 128.66 (CH), 128.06 (CH), 127.87 (CH), 127.53 (CH), 126.49 (CH), 62.67 (CH_2), 62.37 (CH_2), 61.10 (CH_2),60.20 (CH_2), 51.23 (CH2), 48.95 (CH_2), 47.32 (CH_2), 46.09 (CH_2), 30.12 (CH_2), 29.85 (CH_2), 25.94 (CH_3), 25.90 (CH_3), 25.86 (CH_2), 23.86 (CH_2), 20.71 (C), 18.26 (C), 14.17 (CH_3), 14.17 (CH_3), -5.34 (CH_3), -5.36 (CH_3); *m/z* (AP+) 420.1 ([M+H]+, 100%); HRMS found ([M+H]+) 420.2562. $C_{23}H_{38}O_4NSi$ req 420.2565.

Synthesis of Branched Fumarates and Fumaramides

5-((*tert*-Butyldimethylsilyl)oxy)pentan-2-yl ethyl fumarate (11a)

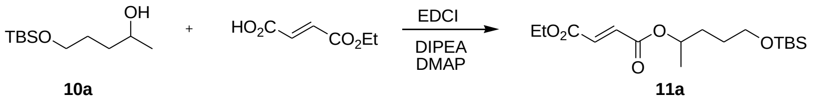

Column chromatography (2:8, EtOAc:PE). Clear oil (227 mg, 0.66 mmol, 56% yield); R_f 0.51 (2:8, EtOAc:PE); ^{1}H NMR (400 MHz, $CDCl_3$) δ 6.84 (s, 2H, fumarate), 5.05 (tq, *J* = 12.2, 6.2 Hz, 1H, H2), 4.27 (q, *J* = 7.1 Hz, 2H, OC*H*$_2$CH$_3$), 3.62 (t, *J* = 6.1 Hz, 2H, H5), 1.71 – 1.60 (m, 2H), 1.60 – 1.51 (m, 2H), 1.33 (t, *J* = 7.1 Hz, 3H, OCH$_2$C*H*$_3$), 1.29 (d, *J* = 6.2 Hz, 3H, H1), 0.90 (s, 9H, ^{t}Bu), 0.05 (s, 6H, (C*H*$_3$)$_2$); ^{13}C NMR (101 MHz, $CDCl_3$) δ 165.1 (C), 164.6 (C), 134.1 (CH), 133.4 (CH), 72.2 (CH), 62.7 (CH_2), 61.3 (CH_2), 32.2 (CH_2), 28.6 (CH_2), 25.9 (CH_3), 19.9 (CH_3), 18.3 (C), 14.1 (CH_3), -5.3 (CH_3); *m/z* (AP+) 345.2 ([M+H]+, 100%); HRMS found ([M+H]+) 345.2091. $C_{17}H_{33}O_5Si$ req 345.2092.

6-((*tert*-Butyldimethylsilyl)oxy)hex-1-en-3-yl ethyl fumarate (11b)

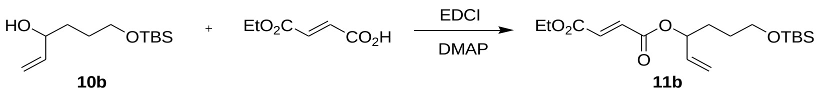

Column chromatography (1:9, EtOAc:PE). Pale yellow oil (227 mg, 0.64 mmol, 59% yield); R_f 0.56 (2:8, EtOAc:PE); ^{1}H NMR (400 MHz, $CDCl_3$) δ 6.81 (s, 2H, fumarate), 5.76 (ddd, J = 17.1, 10.5, 6.5 Hz, 1H, H2), 5.32 (dd, J = 12.9, 6.5 Hz, 1H, H1), 5.19-5.29 (m, 2H, H1'+H3), 4.22 (q, J = 7.1 Hz, 2H, OC*H*$_2$CH$_3$), 3.58 (t, J = 6.2 Hz, 2H, H6), 1.7-1.77 (m, 2H), 1.54-1.60 (m, 2H), 1.28 (t, J = 7.1 Hz, 3H, CH$_2$C*H*$_3$), 0.84 (s, 9H, ^{t}Bu), -0.00 (s, 6H, (C*H*$_3$)$_2$); ^{13}C NMR (101 MHz, $CDCl_3$) δ 165.0 (C), 164.3 (C), 135.9 (CH), 133.8 (CH), 133.7 (CH), 117.3 (CH_2), 75.8 (CH), 62.5 (CH_2), 61.3 (CH_2), 30.6 (CH_2), 28.2 (CH_2), 25.9 (CH_3), 18.3 (C), 14.1 (CH_3), -5.3 (CH_3); *m/z* (AP-) 356.0 ([M]-, 100%); HRMS Found ([M+H]+) 357.2097. $C_{18}H_{33}O_5Si$ req. 357.2092.

6-((*tert*-Butyldimethylsilyl)oxy)-2-methylhexan-3-yl ethyl fumarate (11c)

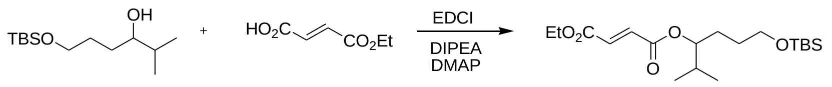

10c **11c**

Column chromatography (1:9, EtOAc:PE). Clear oil (146 mg, 0.39 mmol, 41% yield); R_f 0.37 (1:9, EtOAc:PE); ^{1}H NMR (400 MHz, $CDCl_3$) δ 6.82-6.81 (m, 2H, fumarate), 4.89 – 4.76 (m, 1H, H3), 4.23 (q, *J* = 7.2 Hz, 2H, OCH_2CH_3), 3.56 (td, *J* = 6.2, 2.7 Hz, 2H, H6), 1.85 (dq, *J* = 13.5, 6.8 Hz, 1H, H2), 1.70 – 1.38 (m, 4H, H4+H5), 1.29 (t, *J* = 7.2 Hz, 3H, OCH_2CH_3), 0.87 (d, *J* = 6.8 Hz, 6H, H1+CH_3), 0.84 (s, 9H, ^{t}Bu), -0.00 (s, 6H, $(CH_3)_2$); ^{13}C NMR (101 MHz, $CDCl_3$) δ 165.1 (C), 164.9 (C), 134.0 (CH), 133.4 (CH), 79.6 (CH), 62.6 (CH_2), 61.4 (CH_2), 31.4 (CH), 28.7 (CH_2), 27.3 (CH_2), 25.9 (CH_3), 18.5 (CH_3), 18.3 (C), 17.6 (CH_3), 14.2 (CH_3), -5.3 (CH_3); *m/z* (AP+) 373.2 ([M+H]+, 100%); HRMS found ([M+H]+) 373.2406. $C_{19}H_{37}O_5Si$ req 373.2405.

(*S*)-1-(Benzyloxy)-6-((*tert*-butyldimethylsilyl)oxy)hexan-3-yl ethyl fumarate (11d)

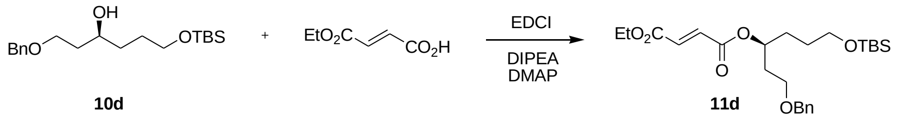

10d **11d**

Column chromatography (1:9, EtOAc:PE). Clear oil (1.435 g, 3.09 mmol, 91% yield); R_f 0.30 (1:9, EtOAc:PE); $[\alpha]_D$ +0.309 (c 0.21, $CHCl_3$); ^{1}H NMR (400 MHz, $CDCl_3$) δ 7.37 – 7.28 (m, 5H, Ar), 6.82 (s, 2H, fumarate), 5.26 – 5.13 (m, 1H, H3), 4.47 (s, 2H, ArCH_2), 4.27 (q, *J* = 7.1 Hz, 2H, OCH_2CH_3), 3.60 (t, *J* = 6.2 Hz, 2H, H6), 3.54 – 3.45 (m, 2H, H1), 1.93 (app q, *J* = 6.3 Hz, 2H), 1.76 – 1.62 (m, 2H), 1.61 – 1.48 (m, 2H), 1.33 (t, *J* = 7.1 Hz, 3H, OCH_2CH_3), 0.88 (s, 9H, ^{t}Bu), 0.04 (s, 6H, $(CH_3)_2$); ^{13}C NMR (101 MHz, $CDCl_3$) δ 165.1 (C), 164.7 (C), 138.2 (C), 133.9 (CH), 133.5 (CH), 128.4 (CH), 127.8 (CH), 127.6 (CH), 73.1 (CH_2), 72.9 (CH), 66.5 (CH_2), 62.7 (CH_2), 61.3 (CH_2), 34.3 (CH_2), 30.7 (CH_2), 28.4 (CH_2), 26.0 (CH_3), 18.3 (C), 14.1 (CH_3), -5.3 (CH_3); *m/z* (AP+) 464.4 ([M+H]+, 100%); HRMS found ([M+H]+) 465.2644. $C_{25}H_{41}O_6Si$ req 465.2667.

(*E*)-Ethyl 4-((5-((*tert*-butyldimethylsilyl)oxy)pentan-2-yl)amino)-4-oxobut-2-enoate (11e)

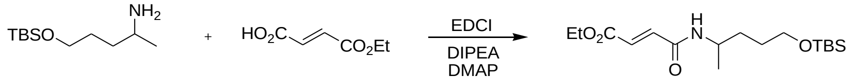

10e **11e**

Column chromatography (1:1, EtOAc:PE). Clear oil (1.311 g, 3.82 mmol, 58% yield); R_f 0.44 (3:7, EtOAc:PE); ^{1}H NMR (400 MHz, $CDCl_3$) δ 6.83 (d, *J* = 15.3 Hz, 1H, fumarate), 6.81 (d, *J* = 15.3 Hz, 1H, fumarate), 5.75 (d, *J* = 7.6 Hz, 1H, N*H*), 4.19 (q, *J* = 7.1 Hz, 2H, OCH_2CH_3), 4.08 – 3.96 (m, 1H, H2), 3.59-3.64 (m, 2H, H5), 1.50 (app s, 4H, CH_2CH_2), 1.25 (t, *J* = 7.1 Hz, 3H, OCH_2CH_3), 1.14 (d, *J* = 6.6 Hz, 3H, H1), 0.84 (s, 9H,^{t}Bu), -0.00 (s, 6H, $(CH_2)_2$); ^{13}C NMR (101 MHz, $CDCl_3$) δ 165.59 (C), 162.89 (C), 136.60 (CH), 130.23 (CH), 62.76 (CH_2), 61.09 (CH_2), 45.63 (CH), 32.98 (CH_2), 28.93 (CH_2), 25.96 (CH_3), 20.78 (CH_3), 18.36 (C), 14.15 (CH_3), -5.29 (CH_3); *m/z* (ES+) 344.3 ([M+H]+, 100%), 366.3 ([M+Na]+, 20%).

3-(benzyloxy)-6-((*tert*-butyldimethylsilyl)oxy)hexyl ethyl fumarate

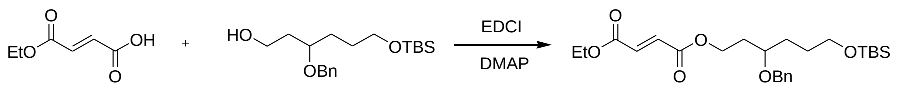

Column chromatography (1:9, EtOAc:PE) . Pale yellow oil (46 mg, 0.099 mmol, 66% yield); R_f 0.52 (2:8, EtOAc:PE); ^{1}H NMR (400 MHz, $CDCl_3$) δ 7.28 (m, 5H, Ph), 6.75 (s, 2H, fumarate), 4.51 (d, *J* = 11.4 Hz, 1H, PhC*H*H'), 4.40 (d, *J* = 11.4 Hz, 1H, PhCH*H*'), 4.27 (t, *J* = 6.8 Hz, 2H, H1), 4.22 (q, *J* = 7.1 Hz, 2H, O$CH_2$$CH_3$), 3.57 (t, *J* = 5.9 Hz, 2H, H6), 3.54 – 3.49 (m, 1H, H3), 1.85 (q, *J* = 6.4 Hz), 1.63 – 1.49 (m, 4H), 1.28 (t, *J* = 7.1 Hz, 3H, CH_2CH_3), 0.85 (s, 9H, ^{t}Bu), 0.00 (s, 6H, $(CH_3)_2$); ^{13}C NMR (101 MHz, $CDCl_3$) δ 165.0 (C), 165.0 (C), 138.5 (C), 133.7 (CH), 133.5 (CH), 128.4 (CH), 127.9 (CH), 127.6 (CH), 75.2 (CH), 71.0 (CH_2), 63.0 (CH_2), 62.4 (CH_2), 61.3 (CH_2), 33.0 (CH_2), 23.0 (CH_2), 28.2 (CH_2), 26.0 (CH_3), 18.3 (C), 14.1 (CH_3), -5.3 (CH_3); *m/z* (AP+) 465.3 ([M+H]+ 100%); HRMS Found ([M+H]+) 465.2666. $C_{25}H_{41}O_6Si$ req. 465.2667.

Boc Protection of Fumaramides

$(Boc)_2O$ (2 eq) was added to a stirred solution of fumaramide (1 eq), NEt_3 (1 eq) and DMAP (1 eq) in DCM and stirred at RT under a blanket N_2 for 16-24 hours. The reaction mixture was washed with 5% aqueous HCl and saturated aqueous sodium bicarbonate, dried ($MgSO_4$) and concentrated *in vacuo.* The crude residue was purified *via* column chromatography.

(*E*)-Ethyl 4-((*tert*-butoxycarbonyl)(4-((*tert*-butyldimethylsilyl)oxy)butyl) amino)-4-oxobut-2-enoate

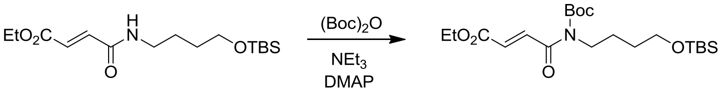

Column chromatography (2:8, EtOAc:PE). Yellow oil (493 mg, 1.15 mmol, 100% yield); R_f 0.64 (3:7, EtOAc:PE); ^{1}H NMR (400 MHz, $CDCl_3$) δ 7.61 (d, *J* = 15.5 Hz, 1H, fumarate), 6.60 (d, *J* = 15.5 Hz, 1H, fumarate), 4.21 (q, *J* = 7.1 Hz, 2H, O$CH_2$$CH_3$), 3.75 – 3.63 (m, 2H, H1), 3.58 (t, *J* = 6.2 Hz, 2H, H4), 1.63 – 1.52 (m, 2H, H2), 1.43 (app s, 11H, ^{t}Bu + H3), 1.27 (t, *J* = 7.1 Hz, 3H, OCH_2CH_3), 0.84 (s, 9H, ^{t}Bu), 0.00 (s, 6H, $(CH_3)_2$); ^{13}C NMR (101 MHz, $CDCl_3$) δ 171.20 (C), 167.26 (C), 165.61 (C), 137.93 (CH), 129.16 (CH), 84.08 (C), 62.74 (CH_2), 61.07 (CH_2), 60.42 (CH_2), 44.63 (CH_2), 30.18 (CH_2), 27.96 (CH_3), 25.95 (CH_3), 25.18 (C), 14.21 (CH_3), -5.30 (CH_3); *m/z* (ES-) 429.2 ([M]-, 100%); HRMS found ([M+H]+) 430.2609. $C_{21}H_{40}O_6NSi$ req. 430.2619.

(*E*)-Ethyl 4-((*tert*-butoxycarbonyl)(5-((*tert*-butyldimethylsilyl)oxy)pentan-2-yl)amino)-4-oxobut-2-enoate (11f)

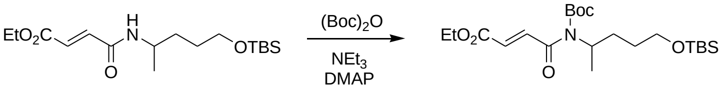

11e **11f**

Column chromatography (2:8, EtOAc:PE). Yellow oil (155 mg, 0.35 mmol, 100% yield); R_f 0.58 (3:7, EtOAc:PE); ^{1}H NMR (400 MHz, $CDCl_3$) δ 7.38 (d, *J* = 15.4 Hz, 1H, fumarate), 6.59 (d, *J* = 15.4 Hz, 1H, fumarate), 4.57 (tq, *J* = 15.7, 6.8 Hz, 1H, H2), 4.22 (q, *J* = 7.1 Hz, 2H, O$CH_2$$CH_3$), 3.55 (t, *J* = 6.4 Hz, 2H, H5), 1.85 (ddd, *J* = 13.3, 9.0, 6.5 Hz, 1H, H3), 1.64 (ddd, *J* = 13.3, 9.1, 6.1 Hz, 1H, H3), 1.50 (s, 9H, ^{t}Bu), 1.42 – 1.38 (m, 2H, H4), 1.29 (d, *J* = 6.8 Hz, 3H, H1), 1.28 (t, *J* = 7.2 Hz, 3H, OCH_2CH_3), 0.85 (s, 9H, ^{t}Bu), -0.00 (s, 6H, $(CH_2)_2$); ^{13}C NMR (101 MHz, $CDCl_3$) δ 167.52 (C), 165.63 (C), 153.08 (C), 138.37 (CH), 128.64 (CH), 84.13 (C), 62.74 (CH_2), 61.04 (CH_2), 51.80 (CH), 30.54 (CH_2), 30.09 (CH_2), 27.96 (CH_3), 25.96 (CH_3), 19.00 (CH_3), 18.35 (C), 14.18 (CH_3), -5.31 (CH_3); *m/z* (ES+) 444.3 ([M+H]+, 100%); HRMS found ([M+H]+) 444.2765. $C_{22}H_{42}O_6NSi$ req 444.2776.

Synthesis of Aldehydes (7a-g, 12a-e, 18)

General Procedure for Deprotection and Oxidation to Aldehydes

pTSA·H_2O (0.1 eq) was added to a stirred solution of TBS protected alcohol (1 eq) in DCM:MeOH (1:1) at 0 °C and stirred for 1-2 hours. The reaction mixture was concentrated *in vacuo* to give the deprotected alcohol which was redissolved in DCM. TEMPO (0.1 eq) and BAIB (1.1 eq) were added and the reaction mixture was stirred at RT under a blanket of N_2 for 1-3 hour. The reaction mixture was washed with saturated aqueous sodium thiosulfate solution, extracted with DCM, washed with saturated aqueous sodium bicarbonate, dried ($MgSO_4$) and concentrated *in vacuo*. The crude residue was purified *via* column chromatography to give the aldehydes **7a**-**g**.

Ethyl (3-oxopropyl) fumarate (7a)

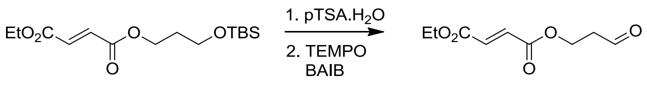

7a

Column chromatography (3:7, EtOAc:PE). Clear oil (93 mg, 0.47 mmol, 53% yield); R_f 0.67 (1:1, EtOAc:PE); ^{1}H NMR (400 MHz, $CDCl_3$) δ 9.83 (d, *J* = 0.9 Hz, 1H, H3), 6.89 – 6.78 (m, 2H, fumarate), 4.56 (t, *J* = 6.1 Hz, 2H, H1), 4.28 (q, *J* = 7.1 Hz, 2H, OC*H*$_2$CH$_3$), 2.87 (td, *J* = 6.1, 1.2 Hz, 2H, H2), 1.34 (t, *J* = 7.1 Hz, 3H, OCH$_2$C*H*$_3$); ^{13}C NMR (101 MHz, $CDCl_3$) δ 198.75 (CH), 164.77 (C), 164.75 (C), 134.37 (CH), 132.78 (CH), 61.43 (CH_2), 58.71 (CH_2), 42.49 (CH_2), 14.09 (CH_3); *m/z* (ES+) 223.0 ([M+Na]+, 100%); HRMS found ([M+H]+) 201.0754. $C_9H_{13}O_5$ req 201.0757.

Ethyl (4-oxobutyl) fumarate (7b)

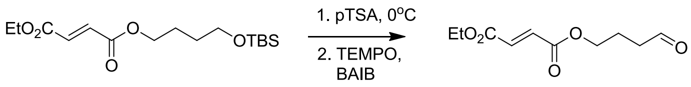

7b

Column chromatography (2:8→3:7, EtOAc:PE). Clear oil (106 mg, 0.50 mmol, 63% yield); R_f 0.51 (1:1, EtOAc:PE); ^{1}H NMR (400 MHz, $CDCl_3$) δ 9.74 (d, *J* = 1.0 Hz, 1H, H4), 6.77 (s, 2H, fumarate), 4.29 – 4.10 (m, 4H, OC*H*$_2$CH$_3$+H1), 2.52 (td, *J* = 7.1, 0.9 Hz, 2H, H3), 2.05 – 1.89 (m, 2H, H2), 1.25 (t, *J* = 7.1 Hz, 3H, OCH$_2$C*H*$_3$); ^{13}C NMR (101 MHz, $CDCl_3$) δ 200.8 (CH), 164.9 (C), 134.1 (CH), 133.1 (CH), 64.2 (CH_2), 61.4 (CH_2), 40.3 (CH_2), 21.2 (CH_2), 14.1 (CH_3); *m/z* (AP+) 215.1 ([M+H]+, 100%); HRMS Found ([M+NH$_4$]+) 232.1181. $C_{10}H_{18}O_5N$ req. 232.1179.

Ethyl (5-oxopentyl) fumarate (7c)

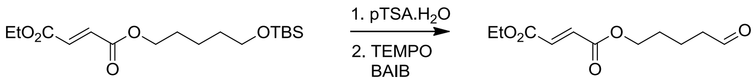

7c

Column chromatography (3:7, EtOAc:PE). Clear oil (127 mg, 0.56 mmol, 39% yield); R_f 0.46 (1:1, EtOAc:PE); ^{1}H NMR (400 MHz, $CDCl_3$) δ 9.72 (t, *J* = 1.2 Hz, 1H, H5), 6.78 (s, 2H, fumarate), 4.20 (q, *J* = 7.2 Hz, 2H, OC*H*$_2$CH$_3$), 4.17 – 4.13 (m, 2H, H1), 2.50 – 2.37 (m, 2H, H4), 1.75 – 1.60 (m, 4H, H2+H3), 1.26 (t, *J* = 7.1 Hz, 3H, OCH$_2$C*H*$_3$); ^{13}C NMR (101 MHz, $CDCl_3$) δ 201.76 (CH), 164.99 (C), 164.94 (C), 133.89 (CH),

133.35 (CH), 64.78 (CH_2), 61.39 (CH_2), 43.27 (CH_2), 27.91 (CH_2), 18.49 (CH_2), 14.11 (CH_3); *m/z* (AP-) 228.0 ([M]-, 100%); HRMS found ([M+NH_4]+) 246.1338. $C_{11}H_{20}O_5N$ req 246.1336.

Ethyl (6-oxohexyl) fumarate (7d)

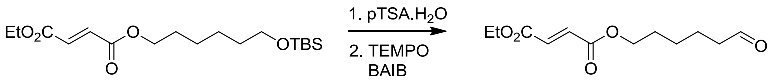

7d

Column chromatography (3:7, EtOAc:PE). Clear oil (205 mg, 0.85 mmol, 71% yield); R_f 0.33 (3:7, EtOAc:PE); ^{1}H NMR (400 MHz, $CDCl_3$) δ 9.80 (s, 1H, H6), 6.87 (s, 2H, fumarate), 4.28 (q, *J* = 7.1 Hz, 2H, OCH_2CH_3), 4.22 (t, *J* = 6.5 Hz, 2H, H1), 2.49 (td, *J* = 7.7, 6.8 Hz, 2H, H5), 1.79 – 1.64 (m, 4H), 1.50 – 1.40 (m, 2H), 1.34 (t, *J* = 7.1 Hz, 3H, OCH_2CH_3); ^{13}C NMR (101 MHz, $CDCl_3$) δ 202.30 (CH), 165.06 (C), 165.00 (C), 133.79 (CH), 133.48 (CH), 65.04 (CH_2), 61.40 (CH_2), 43.71 (CH_2), 28.34 (CH_2), 25.49 (CH_2), 21.61 (CH_2), 14.13 (CH_3); *m/z* (ES+) 243.3 ([M+H]+, 100%); HRMS found ([M+H]+) 243.1225. $C_{12}H_{19}O_5$ req 243.1227.

***tert*-butyl (4-oxobutyl) fumarate (7e)**

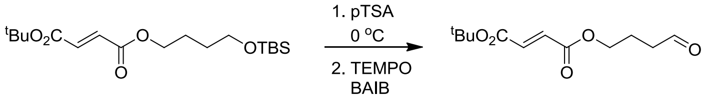

Column chromatography (2:8, EtOAc:PE). Clear oil (222 mg, 0.92 mmol, 62% yield); R_f 0.43 (3:7, EtOAc:PE); ^{1}H NMR (400 MHz, $CDCl_3$) δ 9.83 (s, 1H, H4), 6.78 (m, 2H, fumarate), 4.25 (t, *J* = 6.3 Hz, 2H, H1), 2.61 (td, *J* = 7.1, 0.8 Hz, 2H, H3), 2.13 – 1.99 (m, 2H, H2), 1.53 (s, 9H, ^{t}Bu); ^{13}C NMR (101 MHz, $CDCl_3$) δ 200.88 (CH), 165.10 (C), 164.05 (C), 136.02 (CH), 132.15 (CH), 82.03 (C), 64.11 (CH_2), 40.32 (CH_2), 27.97 (CH_3), 21.18 (CH_2); *m/z* not obtained due to instability.

(*E*)-Ethyl 4-((*tert*-butoxycarbonyl)(4-oxobutyl)amino)-4-oxobut-2-enoate (7f)

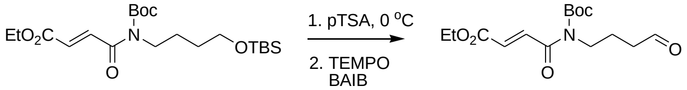

7g

Column chromatography (2:8, EtOAc:PE). Clear oil (230 mg, 0.73 mmol, 63% yield); R_f 0.34 (3:7, EtOAc:PE); ^{1}H NMR (400 MHz, $CDCl_3$) δ 9.80 (s, 1H, H4), 7.66 (d, *J* = 15.4 Hz, 1H, fumarate), 6.66 (d, *J* = 15.4 Hz, 1H, fumarate), 4.27 (q, *J* = 7.1 Hz, 2H, OCH_2CH_3), 3.85 – 3.72 (m, 2H, H1), 2.51 (t, *J* = 7.0 Hz, 2H, H3), 2.01 – 1.84 (m, 2H, H2), 1.57 (s, 9H, ^{t}Bu), 1.33 (t, *J* = 7.1 Hz, 3H, OCH_2CH_3); ^{13}C NMR (101 MHz, $CDCl_3$) δ 201.19 (CH), 167.36 (C), 165.49 (C), 152.53 (C), 137.61 (CH), 129.52 (CH), 84.62 (C), 61.14 (CH_2), 43.85 (CH_2), 41.08 (CH_2), 27.98 (CH_3), 20.95 (CH_2), 14.19 (CH_3); *m/z* (ES+) 313.3 ([M]-, 100%); HRMS found ([M+H]+) 314.1594. $C_{15}H_{24}O_6N$ req. 314.1598.

(*E*)-Ethyl 4-(benzyl(4-oxobutyl)amino)-4-oxobut-2-enoate (7g)

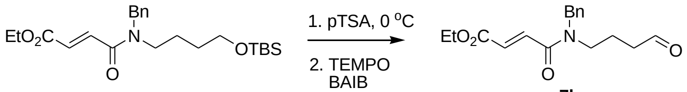

7h

Column chromatography (3:7, EtOAc:PE). Clear oil (360 mg, 1.19 mmol, 78% yield) comprising a mixture of rotamers; R_f 0.28 (1:1, EtOAc:PE); ^{1}H NMR (400 MHz, $CDCl_3$) δ 9.74 (s, 1H, H4), 7.46 (d, *J* = 15.2, 0.6H, fumarate), 7.16-7.37 (m, 5.4H, Ph+fumarate), 6.92 (d, *J* = 15.2, 0.4H, fumarate), 6.84 (d, *J* = 15.2, 0.6H,

fumarate), 4.68 (s, 0.8H, PhC*H*$_2$), 4.62 (s, 1.2H, PhC*H*$_2$), 4.26 (q, *J* = 7.1 Hz, 0.8H, OC*H*$_2$CH$_3$), 4.21 (q, *J* = 7.1 Hz, 1.2H, OC*H*$_2$CH$_3$), 3.44 (t, J = 7.0, 1.2H, H1), 3.33 (dd, J = 7.6, 8.1, 0.8H, H1), 2.44-2.50 (m, 2H, H3), 2.01 – 1.82 (m, 2H, H2), 1.23-1.34 (m, 3H, OCH$_2$C*H*$_3$); ^{13}C NMR (101 MHz, CDCl$_3$) δ 201.32 (CH), 200.31 (CH), 171.14 (C), 165.55 (C), 165.33 (C), 164.75 (C), 136.91 (C), 136.11 (C), 133.66 (CH), 133.37 (CH), 132.32 (CH), 132.05 (CH), 129.05 (CH), 128.72 (CH), 128.20 (CH), 128.02 (CH), 127.68 (CH), 126.58 (CH), 61.20 (CH$_2$), 60.38 (CH$_2$), 51.33 (CH$_2$), 49.06 (CH$_2$), 46.32 (CH$_2$), 45.54 (CH$_2$), 41.15 (CH$_2$), 40.43 (CH$_2$), 21.43 (CH$_2$), 21.04 (CH$_3$), 19.79 (CH$_2$), 14.19 (CH$_3$); *m/z* (AP+) 304.1 ([M+H]+, 100%); HRMS found ([M+O+H]+) 320.1495. $C_{17}H_{22}O_5N$ req 320.1492 (from spontaneous oxidation from aldehyde to carboxylic acid).

Ethyl (5-oxopentan-2-yl) fumarate (12a)

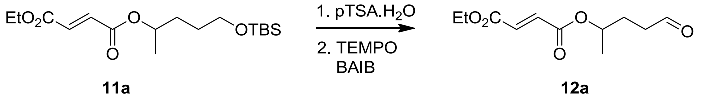

Column chromatography (2:8, EtOAc:PE). Clear oil (73 mg, 0.32 mmol, 84% yield); R$_f$ 0.33 (3:7, EtOAc:PE); ^{1}H NMR (400 MHz, CDCl$_3$) δ 9.79 (t, *J* = 1.1 Hz, 1H, H5), 6.83 (s, 2H, fumarate), 5.11 – 5.00 (m, 1H, H2), 4.27 (q, *J* = 7.1 Hz, 2H, OC*H*$_2$CH$_3$), 2.57 – 2.50 (m, 2H), 2.00 – 1.91 (m, 2H), 1.33 (t, *J* = 7.1 Hz, 3H, OCH$_2$C*H*$_3$), 1.31 (d, *J* = 6.2 Hz, 3H, H1); ^{13}C NMR (101 MHz, CDCl$_3$) δ 201.1 (CH), 165.0 (C), 164.5 (C), 133.9 (CH), 133.6 (CH), 71.3 (CH), 61.4 (CH$_2$), 39.8 (CH$_2$), 28.0 (CH$_2$), 19.9 (CH$_3$), 14.1 (CH$_3$); *m/z* data not obtainable due to instability.

Ethyl (6-oxohex-1-en-3-yl) fumarate (12b)

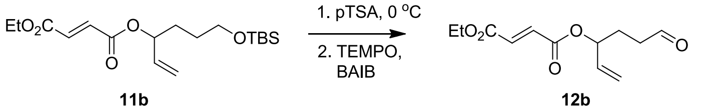

Column chromatography (3:7, EtOAc:PE). Clear oil (30 mg, 0.125 mmol, 63% yield); R$_f$ 0.54 (1:1, EtOAc:PE); ^{1}H NMR (400 MHz, CDCl$_3$) δ 9.80 (s, 1H, H6), 6.87 (s, 2H, fumarate), 5.81 (ddd, *J* = 17.1, 10.6, 6.4 Hz, 1H, H2), 5.41 (dd, *J* = 12.5, 6.4 Hz, 1H, H1), 5.35 – 5.25 (m, 2H, H1'+H3), 4.29 (q, *J* = 7.1 Hz, 2H, OC*H*$_2$CH$_3$), 2.55 (t, *J* = 7.2 Hz, 2H, H5), 2.11 – 2.00 (m, 2H, C*H*$_2$), 1.35 (t, *J* = 7.1 Hz, 3H, CH$_2$C*H*$_3$); ^{13}C NMR (101 MHz, CDCl$_3$) δ 200.7 (CH), 164.9 (C), 164.1 (C), 135.0 (CH), 134.2 (CH), 133.3 (CH), 118.1 (CH$_2$), 74.7 (CH), 61.4 (CH$_2$), 39.3 (CH$_2$), 26.3 (CH$_2$), 14.1 (CH$_3$); *m/z* (AP+) 241.1 ([M+H]+, 60%), 96.8 (100%); HRMS Found ([M+NH$_4$]+) 258.1338. $C_{12}H_{20}O_5N$ req. 258.1336.

Ethyl (2-methyl-6-oxohexan-3-yl) fumarate (12c)

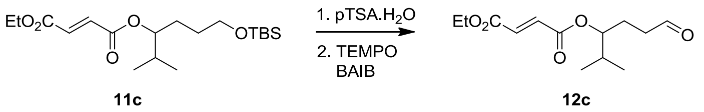

Column chromatography (1:9, EtOAc:PE). Clear oil (158 mg, 0.62 mmol, 49% yield); R$_f$ 0.28 (2:8, EtOAc:PE); ^{1}H NMR (400 MHz, CDCl$_3$) δ 9.77 (s, 1H, H6), 6.86 (s, 2H, fumarate), 4.87 (ddd, *J* = 9.1, 5.6, 3.4 Hz, 1H, H3), 4.28 (q, *J* = 7.1 Hz, 2H, OC*H*$_2$CH$_3$), 2.49 (t, *J* = 7.4 Hz, 2H, H5), 2.05 – 1.93 (m, 1H, H2), 1.93 – 1.82 (m, 2H), 1.34 (t, *J* = 7.1 Hz, 3H, OCH$_2$C*H*$_3$), 0.94 (d, *J* = 6.8 Hz, 6H, H1); ^{13}C NMR (101 MHz, CDCl$_3$) δ 201.2 (CH), 165.0 (C), 164.9 (C), 134.0 (CH), 133.5 (CH), 78.9 (CH), 61.5 (CH$_2$), 40.2 (CH$_2$), 31.7 (CH), 23.4

(CH_2), 18.3 (CH_3), 17.7 (CH_3), 14.1 (CH_3); *m/z* (AP+) 257.1 ([M+H]+, 100%); HRMS found ([$M+NH_4$]+) 274.1650. $C_{13}H_{24}O_5N$ req 274.1649.

(*S*)-1-(Benzyloxy)-6-oxohexan-3-yl ethyl fumarate (12d)

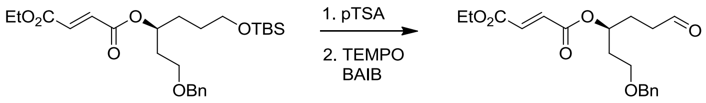

11d **12d**

Column chromatography (3:7, EtOAc:PE). Clear oil (776 mg, 2.23 mmol, 72% yield); R_f 0.30 (3:7, EtOAc:PE); $[\alpha]_D$ -0.206 (c 0.65, $CHCl_3$); ^{1}H NMR (400 MHz, $CDCl_3$) δ 9.76 (s, 1H, H6), 7.40 – 7.28 (m, 5H, Ar), 6.81 (s, 2H, fumarate), 5.28 – 5.12 (m, 1H, H3), 4.46 (s, 2H, ArC*H*$_2$), 4.27 (q, *J* = 7.1 Hz, 2H, OC*H*$_2$CH$_3$), 3.59 – 3.40 (m, 2H), 2.52 (t, *J* = 7.5 Hz, 2H, H5), 2.01 – 1.86 (m, 4H), 1.33 (t, *J* = 7.1 Hz, 3H, OCH$_2$C*H*$_3$); ^{13}C NMR (101 MHz, $CDCl_3$) δ 201.0 (CH), 164.9 (C), 164.6 (C), 138.0 (C), 134.0 (CH), 133.4 (CH), 128.4 (CH), 127.8 (CH), 127.7 (CH), 73.2 (CH_2), 72.2 (CH), 66.2 (CH_2), 61.4 (CH_2), 39.8 (CH_2), 34.3 (CH_2), 26.6 (CH_2), 14.1 (CH_3); *m/z* (ES+) 398.2 (100%), 366.2 ([$M+NH_4$]+, 70%); HRMS data not obtainable due to instability.

(*E*)-Ethyl 4-((*tert*-butoxycarbonyl)(5-oxopentan-2-yl)amino)-4-oxobut-2-enoate (12e)

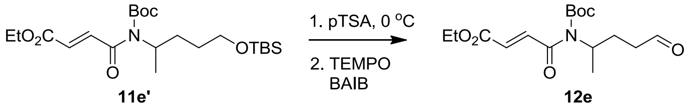

11e' **12e**

Column chromatography (2:8, EtOAc:PE). Clear oil (66 mg, 0.21 mmol, 39% yield); R_f 0.48 (3:7, EtOAc:PE); ^{1}H NMR (400 MHz, $CDCl_3$) δ 9.65 (s, 1H, H5), 7.31 (d, *J* = 15.4 Hz, 1H, fumarate), 6.54 (d, *J* = 15.4 Hz, 1H, fumarate), 4.59 – 4.43 (m, 1H, H2), 4.16 (q, *J* = 7.1 Hz, 2H, OC*H*$_2$CH$_3$), 2.42 – 2.20 (m, 2H, H4), 2.18 – 2.04 (m, 1H, H3), 1.92 – 1.78 (m, 1H, H3), 1.44 (s, 9H, ^{t}Bu), 1.26 (d, *J* = 6.9 Hz, 3H, H1), 1.21 (t, *J* = 7.1 Hz, 3H, OCH$_2$C*H*$_3$); ^{13}C NMR (101 MHz, $CDCl_3$) δ 201.21 (CH), 167.59 (C), 165.50 (C), 152.81 (C), 137.97 (CH), 129.11 (CH), 84.66 (C), 61.13 (CH_2), 51.34 (CH), 41.13 (CH_2), 27.94 (CH_3), 26.43 (CH_2), 18.84 (CH_3), 14.17 (CH_3); *m/z* (ES-) 326.2 ([M-H]-, 100%); HRMS found ([M+H]+) 326.1611. $C_{16}H_{24}O_6N$ req 326.1609.

3-(benzyloxy)-6-oxohexyl ethyl fumarate (18)

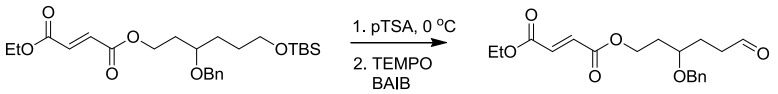

Column chromatography (3:7, EtOAc:PE). Clear oil (37 mg, 0.106 mmol, 53% yield); R_f 0.33 (3:7, EtOAc:PE); ^{1}H NMR (400 MHz, $CDCl_3$) δ 9.70 (d, *J* = 1.2 Hz, 1H, H6), 7.29 – 7.20 (m, 5H, Ph), 6.74 (s, 2H, fumarate), 4.41 (d, *J* = 2.4 Hz, 2H, PhC*H*$_2$), 4.24 (t, *J* = 6.6 Hz, 2H, H1), 4.19 (q, *J* = 7.2 Hz, 2H, OC*H*$_2$CH$_3$), 3.56 – 3.46 (m, 1H, H3), 2.46 (t, *J* = 7.2 Hz, 2H, H5), 1.97 – 1.73 (m, 4H, H2+H4), 1.26 (t, *J* = 7.1 Hz, 3H, CH$_2$C*H*$_3$); ^{13}C NMR (101 MHz, $CDCl_3$) δ 201.9 (CH), 164.9 (C), 164.9 (C), 137.9 (C), 133.9 (CH), 133.3 (CH), 128.5 (CH), 128.0 (CH), 127.9 (CH), 74.3 (CH), 71.2 (CH_2), 62.0 (CH_2), 61.4 (CH_2), 39.5 (CH_2), 32.8 (CH_2), 26.1 (CH_2), 14.1 (CH_3); *m/z* (ES+) 371.1 ([M+Na]+ 100%); HRMS found ([$M+NH_4$]+) 366.1911. $C_{19}H_{28}O_6N$ req. 366.1911.

Synthesis of Cyclobutenes

General Procedure for Enamine [2+2] cyclisation

Diethylamine (2 eq) was added to a stirred solution of aldehyde **7a**-**g** (1 eq) and K_2CO_3 (2 eq) in MeCN and stirred at RT under a blanket of N_2 for 24-48 hours. The reaction mixture was then filtered through Celite® and concentrated *in vacuo*. The crude cyclobutane **8** was dissolved in MeCN and MeI (5 eq) was added and the reaction mixture stirred at RT under a blanket of N_2 for 16-72 hours. The reaction mixture was concentrated *in vacuo* and the crude residue dissolved in MeCN and K_2CO_3 (2 eq) was added and the reaction mixture heated at 60 °C for 24 hours. The reaction mixture was cooled and concentrated *in vacuo*. The crude residue was purified *via* column chromatography.

Synthesis of Unsubstituted Cyclobutenes (9a, e, f, g)

(1S*,6S*)-Ethyl 2-oxo-3-oxabicyclo[4.2.0]oct-7-ene-8-carboxylate (9b)

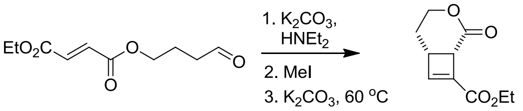

7b **9b**

Column chromatography (1:1, EtOAc:PE). Clear oil (12 mg, 0.061 mmol, 41% yield); R_f 0.21 (1:1, EtOAc:PE); IR 2933.9 cm^{-1} (CH Sp^3), 1715.8 cm^{-1} (C=O ester); ^{1}H NMR (400 MHz, $CDCl_3$) δ 6.86 (s, 1H, H7), 4.34 – 4.21 (m, 2H, H4), 4.17 (q, *J* = 7.1 Hz, 2H, OC*H*$_2$CH$_3$), 3.82 (d, *J* = 4.1 Hz, 1H, H1), 3.37 – 3.29 (m, 1H, H6), 2.11 – 2.00 (m, 1H, H5), 1.96 (dd, *J* = 4.5, 2.3 Hz, 1H, H5'), 1.24 (t, *J* = 7.1 Hz, 3H, CH$_2$C*H*$_3$); ^{13}C NMR (101 MHz, $CDCl_3$) δ 169.1 (C), 160.4 (C), 148.2 (CH), 135.8 (C), 64.6 (CH_2), 61.0 (CH_2), 45.1 (CH), 37.7 (CH), 26.0 (CH_2), 14.1 (CH_3); *m/z* (AP+) 196.7 ([M+H]+, 100%); HRMS Found ([M+H]+) 197.0808. $C_{10}H_{13}O_4$ req. 197.0808; LCMS rt 2.24, *m/z* (ES+) 197.1 ([M+H]+, 100%), >95%.

(1S*,6S*)-*tert*-butyl 2-oxo-3-oxabicyclo[4.2.0]oct-7-ene-8-carboxylate (9e)

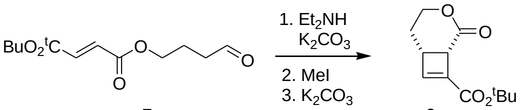

7e **9e**

Treated with diethylamine for 108 hours. Column chromatography (1:1, EtOAc:PE). Yellow oil (40 mg, 0.18 mmol, 20% yield); R_f 0.23 (1:1, EtOAc:PE); IR 2934.1 cm^{-1} (CH), 1714.9 (C=O); ^{1}H NMR (400 MHz, $CDCl_3$) δ 6.77 (s, 1H, H7), 4.34 – 4.17 (m, 2H, H4), 3.77 (d, *J* = 4.1 Hz, 1H, H1), 3.28 (appt, *J* = 4.1 Hz, 1H, H6), 2.10 – 1.96 (m, 1H, H5), 1.97 – 1.87 (m, 1H, H5'), 1.42 (s, 9H, ^{t}Bu); ^{13}C NMR (101 MHz, $CDCl_3$) δ 169.16 (C), 159.77 (C), 146.94 (CH), 137.07 (C), 82.03 (C), 64.50 (CH_2), 45.25 (CH), 37.20 (CH), 27.99 (CH_3), 26.02 (CH_2); *m/z* (AP+) 225.1 ([M+H]+, 20%), 169.0 ([M-^{t}Bu]+, 100%); HRMS found 225.1121. $C_{12}H_{17}O_4$ req 225.1121; LCMS rt3.78, *m/z* (ES+) 247.2 ([M+Na]+, 100%), >95%.

(1S*,6S*)-3-*tert*-Butyl 8-ethyl2-oxo-3-azabicyclo[4.2.0]oct-7-ene-3,8-dicarboxylate (9f)

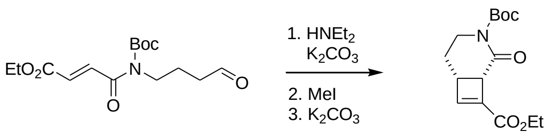

7g **9g**

Column chromatography (1:1, EtOAc:PE). Yellow oil (73 mg, 0.25 mmol, 36% yield); R_f 0.27 (1:1, EtOAc:PE); IR 2980.8 cm^{-1} (CH Sp^3), 1768.5 cm^{-1} (C=O carbamate) 1711.2 cm^{-1} (C=O ester); ^1H NMR (400 MHz, $CDCl_3$) δ 6.85 (s, 1H, H7), 4.19 – 4.11 (m, 2H, OCH_2CH_3), 4.01 (dt, *J* = 13.2, 3.2 Hz, 1H, H6), 3.77 (d, *J* = 4.1 Hz, 1H, H1), 3.35 – 3.21 (m, 2H, H4), 1.97 – 1.92 (m, 1H, H5), 1.90 – 1.80 (m, 1H, H5'), 1.45 (s, 9H, ^tBu), 1.22 (t, *J* = 7.2 Hz, 3H, OCH_2CH_3); ^{13}C NMR (101 MHz, $CDCl_3$) δ 168.90 (C), 160.82 (C), 152.28 (C), 148.42 (CH), 136.28 (C), 83.13 (C), 60.84 (CH_2), 49.18 (CH), 40.69 (CH), 39.13 (CH_2), 28.01 (CH_3), 26.24 (CH_2), 14.16 (CH_3); *m/z* (ES+) 296.1 ([M+H]+, 100%); HRMS found ([M+H]+) 296.1490. $C_{15}H_{22}O_5N$ req 296.1492; LCMS rt 5.74, *m/z* (ES+) 245.1 ([M+H]+, 100%), >95%.

(1S*,6S*)-Ethyl 3-benzyl-2-oxo-3-azabicyclo[4.2.0]oct-7-ene-8-carboxylate (9g)

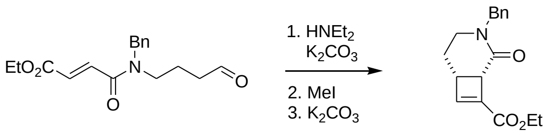

7h **9h**

Column chromatography (7:3, EtOAc:PE). Clear oil (82 mg, 0.29 mmol, 24% yield); R_f 0.21 (3:1, EtOAc:PE); IR 2933.9 cm^{-1} (CH Sp^3), 1708.1 cm^{-1} (C=O ester), 1634.9 cm^{-1} (C=O amide), 717.5 cm^{-1} (mono subs. Benzene); ^1H NMR (400 MHz, $CDCl_3$) δ 7.29 – 7.07 (m, 5H, Ar), 6.83 (s, 1H, H7), 4.68 (d, *J* = 14.8 Hz, 1H, PhC*H*H'), 4.45 (d, *J* = 14.8 Hz, 1H, PhCH*H*'), 4.19 (dd, *J* = 9.8, 3.7 Hz, 2H, OCH_2CH_3), 3.79 (d, *J* = 4.0 Hz, 1H, H1), 3.22 (app s, 1H, H6), 3.18 – 2.97 (m, 2H, H4), 1.81 – 1.63 (m, 2H, H5), 1.25 (t, *J* = 6.9 Hz, 3H, OCH_2CH_3); ^{13}C NMR (101 MHz, $CDCl_3$) δ 168.47 (C), 161.14 (C), 148.24 (CH), 137.33 (C), 136.14 (C), 128.57 (CH), 127.81 (CH), 127.38 (CH), 60.78 (CH_2), 50.78 (CH_2), 47.09 (CH), 42.88 (CH_2), 39.21 (CH), 26.66 (CH_2), 14.24 (CH_3); *m/z* (AP+) 286.1 ([M+H]+, 100%); HRMS found ([M+H]+) 286.1439. $C_{17}H_{20}O_3N$ req 286.1438; LCMS rt 5.80, *m/z* (ES+) 286.2 ([M+H]+, 100%), >95%.

Synthesis of Substituted Cyclobutenes (13a-e)

(1*S,4*S**,6*S**)-Ethyl 4-methyl-2-oxo-3-oxabicyclo[4.2.0]oct-7-ene-8-carboxylate (13a)**

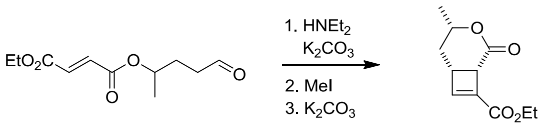

12a **13a**

Column chromatography (1:1, EtOAc:PE). Clear oil (45 mg, 0.21 mmol, 54% yield); R_f 0.22 (1:1, EtOAc:PE); IR 2984.7 cm^{-1} (CH Sp^3), 1710.8 cm^{-1} (C=O ester); ^1H NMR (400 MHz, $CDCl_3$) δ 6.81 (s, 1H, H7), 4.40 – 4.28 (m, 1H, H4), 4.19 (q, *J* = 7.1 Hz, 2H, OCH_2CH_3), 3.88 (d, *J* = 4.8 Hz, 1H, H1), 3.27 – 3.14 (m, 1H, H6), 2.16 (ddd, *J* = 14.2, 7.8, 1.3 Hz, 1H, H5), 1.43 (app dt, *J* = 14.2, 10.7 Hz, 1H, H5'), 1.32 (d, *J* = 6.3 Hz, 3H, CH_3), 1.25 (t, *J* = 7.1 Hz, 3H, OCH_2CH_3); ^{13}C NMR (101 MHz, $CDCl_3$) δ 169.9 (C), 161.2 (C), 148.7 (CH), 136.9 (C), 76.2 (CH), 60.8 (CH_2), 42.2 (CH), 37.8 (CH), 35.4 (CH_2), 21.0 (CH_3), 14.2 (CH_3); *m/z* (ES+)

233.1([M+Na]+, 100%) 211.1 ([M+H]+, 10%); HRMS found ([M+H]+) 211.0964. $C_{11}H_{15}O_4$ req 211.0965; LCMS rt 2.71, *m/z* (ES+) 211.2 ([M+H]+, 100%), >95%.

(1*S,4*R**,6*S**)-Ethyl 2-oxo-4-vinyl-3-oxabicyclo[4.2.0]oct-7-ene-8-carboxylate (13b)**

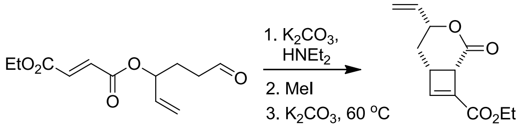

12b **13b**

Column chromatography (1:1, EtOAc:PE). Clear oil (30 mg, 0.135 mmol, 48% yield); R_f 0.28 (1:1, EtOAc:PE); IR 2993.3 cm-1 (C=C), 2942.9 cm^{-1} (CH Sp^3), 1721.1 cm^{-1} (C=O ester), 1711.7 cm^{-1} (C=O ester); ^{1}H NMR (400 MHz, CDCl$_3$) δ 6.82 (s, 1H, H7), 5.80 (ddd, *J* = 17.2, 10.6, 5.7 Hz, 1H, C*H*=CH$_2$), 5.29 (d, *J* = 17.2 Hz, 1H, CH=C*H*H'), 5.17 (d, *J* = 10.6 Hz, 1H, CH=CH*H*'), 4.79 – 4.69 (m, 1H, H4), 4.19 (q, *J* = 7.1 Hz, 2H, OC*H*$_2$CH$_3$), 3.92 (d, *J* = 4.5 Hz, 1H, H1), 3.26 (dddd, *J* = 9.0, 7.6, 4.6, 0.9 Hz, 1H, H6), 2.25 (ddd, *J* = 14.2, 7.6, 2.5 Hz, 1H, H5), 1.64 (dt, *J* = 14.2, 9.0 Hz, 1H, H5'), 1.24 (t, *J* = 7.1 Hz, 3H, CH$_2$C*H*$_3$); ^{13}C NMR (101 MHz, CDCl$_3$) δ 169.23 (C), 161.0 (C), 149.0 (CH), 136.7 (C), 135.5 (CH), 117.2 (CH$_2$), 79.6 (CH), 60.8 (CH$_2$), 42.8 (CH), 37.6 (CH), 33.5 (CH$_2$), 14.2 (CH$_3$); *m/z* (AP+) 223.2 ([M+H]+, 100%); HRMS Found ([M+H]+) 223.0965. $C_{12}H_{15}O_4$ req. 223.0967; LCMS rt 3.05, *m/z* (ES+) 245.1 ([M+Na]+, 100%), >95%.

(1*S,4*R**,6*S**)-Ethyl 4-isopropyl-2-oxo-3-oxabicyclo[4.2.0]oct-7-ene-8-carboxylate (13c)**

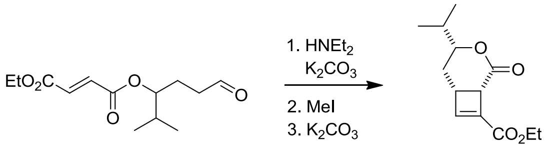

12c **13c**

Column chromatography (3:7, EtOAc:PE). Clear oil (70 mg, 0.29 mmol, 47% yield); R_f 0.20 (3:7, EtOAc:PE); IR 2970.1 cm^{-1} (CH Sp^3), 1720.2 cm^{-1} (C=O ester); ^{1}H NMR (400 MHz, CDCl$_3$) δ 6.80 (s, 1H, H7), 4.19 (q, *J* = 7.1 Hz, 2H, OC*H*$_2$CH$_3$), 3.96 (ddd, *J* = 10.9, 5.1, 0.9 Hz, 1H, H4), 3.89 (d, *J* = 4.5 Hz, 1H, H1), 3.19 – 3.12 (m, 1H, H6), 2.14 (ddd, *J* = 14.0, 7.9, 1.2 Hz, 1H, H5), 1.90 – 1.80 (m, 1H, C*H*(CH$_3$)$_2$), 1.39 (dt, *J* = 14.0, 10.9 Hz, 1H, H5'), 1.25 (t, *J* = 7.1 Hz, 3H, OCH$_2$C*H*$_3$), 0.92 (d, *J* = 4.6 Hz, 3H, C*H*$_3$), 0.91 (d, *J* = 4.6 Hz, 3H, C*H*$_3$); ^{13}C NMR (101 MHz, CDCl$_3$) δ 170.0 (C), 161.2 (C), 148.5 (CH), 137.0 (C), 84.3 (CH), 60.8 (CH$_2$), 42.7 (CH), 37.9 (CH), 31.9 (CH), 30.5 (CH$_2$), 17.9 (CH$_3$), 17.5 (CH$_3$), 14.2 (CH$_3$); *m/z* (AP+) 239.1 ([M+H]+, 100%); HRMS found ([M+H]+) 239.1279. $C_{13}H_{19}O_4$ req 239.1278; LCMS rt 5.03, *m/z* (ES+) 239.2 ([M+H]+, 100%), >95%.

(4*S*)-Ethyl 4-(2-(benzyloxy)ethyl)-2-oxo-3-oxabicyclo[4.2.0]oct-7-ene-8-carboxylate (13d)

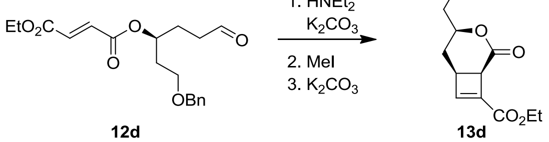

12d **13d**

Column chromatography (1:1, EtOAc:PE). Clear oil (364 mg, 1.10 mmol, 62% yield); R_f 0.38 (1:1, EtOAc:PE); [α]$_D$ +0.499 (c 0.57, CHCl$_3$); IR 2928.8 cm^{-1} (CH Sp^3), 1718.1 cm^{-1} (C=O ester); ^{1}H NMR (400 MHz, CDCl$_3$) δ 7.40 – 7.28 (m, 5H, Ar), 6.86 (s, 1H, H7), 4.52 (d, *J* = 11.7 Hz, 1H, ArC*H*H'), 4.48 (app d, *J* =

11.7 Hz, 2H, ArCH*H'* + H4), 4.26 (q, *J* = 7.1 Hz, 2H, OCH_2CH_3), 3.96 (d, *J* = 4.8 Hz, 1H, H1), 3.68 (td, *J* = 8.9, 4.6 Hz, 1H, BnOC*H*H'), 3.64 – 3.57 (m, 1H, BnOC*H*H'), 3.30 – 3.18 (m, 1H,H6), 2.25 (dd, *J* = 14.1, 7.9 Hz, 1H, H5), 2.04 – 1.83 (m, 2H), 1.52 (app dt, *J* = 13.9, 10.7 Hz, 1H, H5'), 1.32 (t, *J* = 7.1 Hz, 3H, OCH_2C*H_3*); ^{13}C NMR (101 MHz, CDCl$_3$) δ 169.8 (C), 161.2 (C), 148.6 (CH), 138.1 (C), 136.9 (C), 128.4 (CH), 127.7 (2CH), 77.0 (CH), 73.2 (CH$_2$), 65.8 (CH$_2$), 60.8 (CH$_2$), 42.6 (CH), 37.8 (CH), 35.4 (CH$_2$), 33.9 (CH$_2$), 14.2 (CH$_3$); *m/z* (ES+) 331.0 ([M+H]+, 100%); HRMS found ([M+H]+) 331.1541. $C_{19}H_{23}O_5$ req 331.1540; LCMS rt 7.73, *m/z* (ES+) 331.2 ([M+H]+, 100%), >95%.

(1S*,4S*,6S*)-3-*tert*-Butyl 8-ethyl 4-methyl-2-oxo-3-azabicyclo[4.2.0]oct-7-ene-3,8-dicarboxylate (13e)

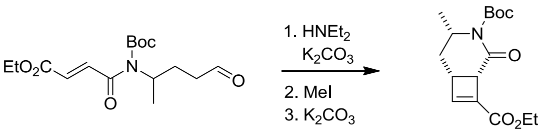

12e **13e**

Column chromatography (2:8, EtOAc:PE). Clear oil (128 mg, 0.43 mmol, 27% yield); R_f 0.50 (1:1, EtOAc:PE); IR 2980.5 cm^{-1} (CH Sp^3), 1763.3 cm^{-1} (C=O carbamate), 1712.2 cm^{-1} (C=O ester); ^{1}H NMR (400 MHz, CDCl$_3$) δ 6.84 (s, 1H, H7), 4.45 – 4.33 (m, 1H, H4), 4.17 (q, *J* = 7.1 Hz, 2H, OCH_2CH$_3$), 3.89 (d, *J* = 4.7 Hz, 1H, H1), 3.33 – 3.21 (m, 1H, H6), 2.01 (ddd, *J* = 14.0, 7.2, 4.3 Hz, 1H, H5), 1.72 (ddd, *J* = 14.0, 10.5, 3.6 Hz, 1H, H5'), 1.45 (s, 9H, ^{t}Bu), 1.29 – 1.18 (m, 6H, CH_3 + OCH$_2$CH_3); ^{13}C NMR (101 MHz, CDCl$_3$) δ 169.52 (C), 161.55 (C), 152.26 (C), 148.78 (CH), 136.91 (C), 83.17 (C), 60.65 (CH$_2$), 52.33 (CH), 46.56 (CH), 37.14 (CH), 33.19 (CH$_2$), 27.99 (CH$_3$), 19.18 (CH$_3$), 14.21 (CH$_3$); *m/z* (ES+) 332.0 ([M+Na]+, 100%), 310.1 ([M+H]+, 40%); HRMS found ([M+H]+) 310.1650. $C_{16}H_{24}O_5N$ req 310.1649; LCMS rt 7.20, *m/z* (ES+) 310.1 ([M+H]+, 100%), >95%.

References

1. E. J. Kang, E. J. Cho, M. K. Ji, Y. E. Lee, D. M. Shin, S. Y. Choi, Y. K. Chung, J.-S. Kim, H.-J. Kim, S.-G. Lee, M. S. Lah and E. Lee, *J. Org. Chem.*, 2005, **70**, 6321-6329.
2. B. M. Trost and T. R. Verhoeven, *J. Am. Chem. Soc.*, 1980, **102**, 4743-4763.
3. K. J. Frankowski, J. E. Golden, Y. Zeng, Y. Lei and J. Aubé, *J. Am. Chem. Soc.*, 2008, **130**, 6018-6024.
4. B.-S. Chen, L.-H. Yang, J.-L. Ye, T. Huang, Y.-P. Ruan, J. Fu and P.-Q. Huang, *Eur. J. Med. Chem.*, 2011, **46**, 5480-5486.
5. H.-L. Huang, C.-N. Yeh, K.-W. Chang, J.-T. Chen, K.-J. Lin, L.-W. Chiang, K.-C. Jeng, W.-T. Wang, K.-H. Lim, C. G. Chen, K.-I. Lin, Y.-C. Huang, W.-J. Lin, T.-C. Yen and C.-S. Yu, *Bioorg. Med. Chem. Lett.*, 2012, **22**, 3998-4003.
6. K. Asano and S. Matsubara, *J. Am. Chem. Soc.*, 2011, **133**, 16711-16713.
7. G. Bélanger, R. Larouche-Gauthier, F. Ménard, M. Nantel and F. Barabé, *J. Org. Chem.*, 2005, **71**, 704-712.
8. K. C. Nicolaou, E. A. Theodorakis, F. P. J. T. Rutjes, M. Sato, J. Tiebes, X. Y. Xiao, C. K. Hwang, M. E. Duggan and Z. Yang, *J. Am. Chem. Soc.*, 1995, **117**, 10239-10251.
9. N. Jana, T. Mahapatra and S. Nanda, *Tetrahedron: Asymmetry*, 2009, **20**, 2622-2628.
10. G. Pattenden, M. A. Gonzalez, P. B. Little, D. S. Millan, A. T. Plowright, J. A. Tornos and T. Ye, *Org. Biomol. Chem.*, 2003, **1**, 4173-4208.

11. A. Börner, J. Ward, W. Ruth, J. Holz, A. Kless, D. Heller and H. B. Kagan, *Tetrahedron*, 1994, **50**, 10419-10430.
12. J. C. Martin, D. F. Smee and J. P. H. Verheyden, *J. Org. Chem.*, 1985, **50**, 755-759.
13. L. R. Takaoka, A. J. Buckmelter, T. E. LaCruz and S. D. Rychnovsky, *J. Am. Chem. Soc.*, 2005, **127**, 528-529.
14. Y.-j. Liu, B. E. Tropp and R. Engel, *Can. J. Chem.*, 1993, **71**, 206-209.
15. B. J. Naysmith and M. A. Brimble, *Org. Lett.*, 2013, **15**, 2006-2009.
16. G. Koza, C. Theunissen, J. R. Al Dulayymi and M. S. Baird, *Tetrahedron*, 2009, **65**, 10214-10229.
17. A. Hassan, Y. Lu and M. J. Krische, *Org. Lett.*, 2009, **11**, 3112-3115.
18. D. Díez-Martin, N. R. Kotecha, S. V. Ley, S. Mantegani, J. C. Menéndez, H. M. Organ, A. D. White and B. J. Banks, *Tetrahedron*, 1992, **48**, 7899-7938.
19. A. Saito, K. Saito, A. Tanaka and T. Oritani, *Tetrahedron Letters*, 1997, **38**, 3955-3958.
20. S. P. Kotkar and A. Sudalai, *Tetrahedron: Asymmetry*, 2006, **17**, 1738-1742.

Part II NMR spectra of novel compounds and intermediates

7a-g (+ intermediate to each compound), **9b**, **9e**, **9f**, **9g**, **10c**, **10d**, **11a-f**, **12a-e**, **13a-e**, **18** (+two intermediates).
