# Supplementary material for: Intramolecular thermal stepwise [2 + 2] cycloadditions: investigation of a stereoselective synthesis of [n.2.0]-bicyclolactones
Source: Org Biomol Chem. 2016 Sep 20;14(40):9554–9. doi: 10.1039/c6ob01661h (PMC5066561; doi:10.1039/c6ob01661h)
Supplement: Supplementary file 2 [file OB-140-C6OB01661H-s002.pdf]

Electronic Supplementary Material (ESI) for Organic & Biomolecular Chemistry.
This journal is © The Royal Society of Chemistry 2016

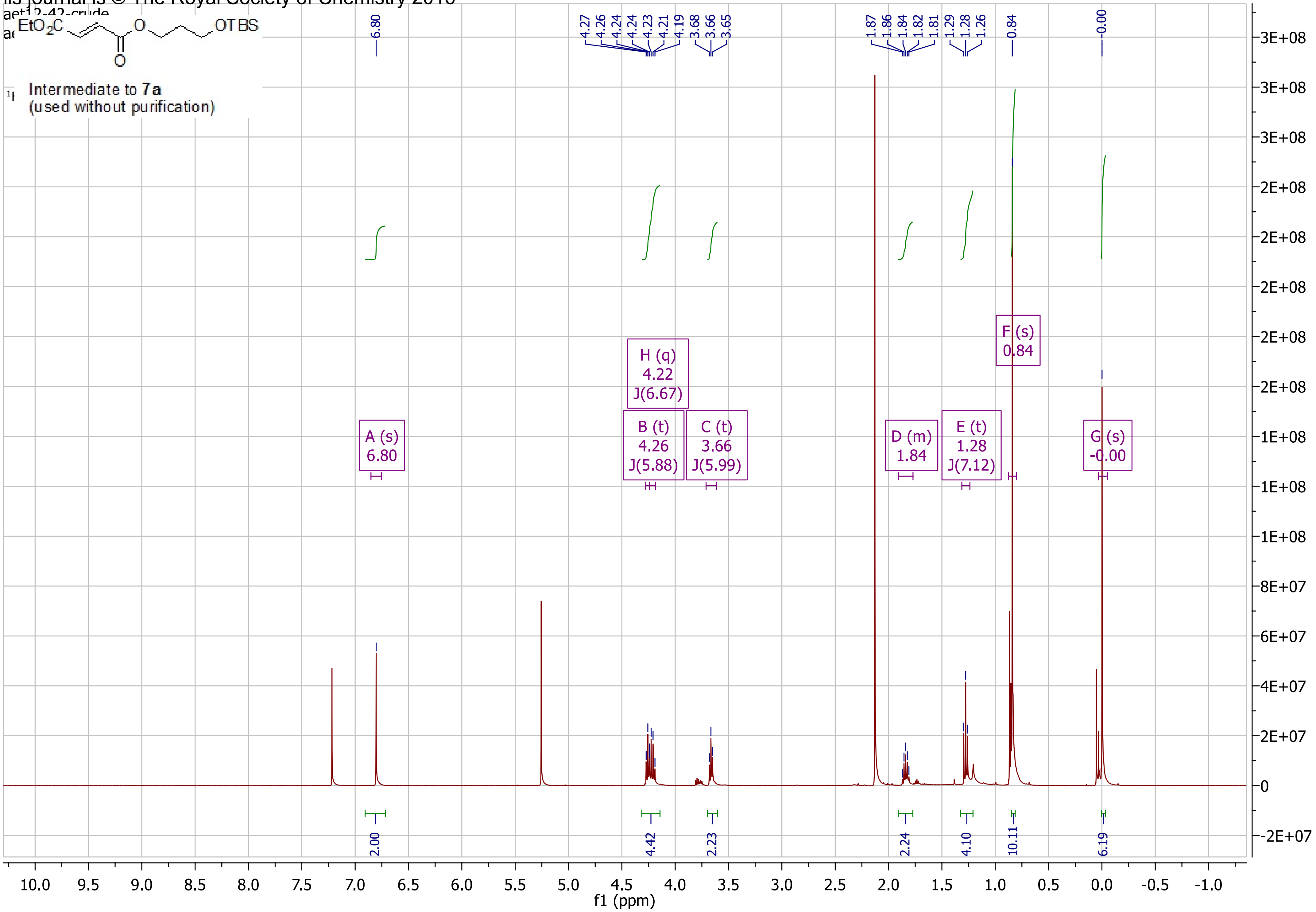

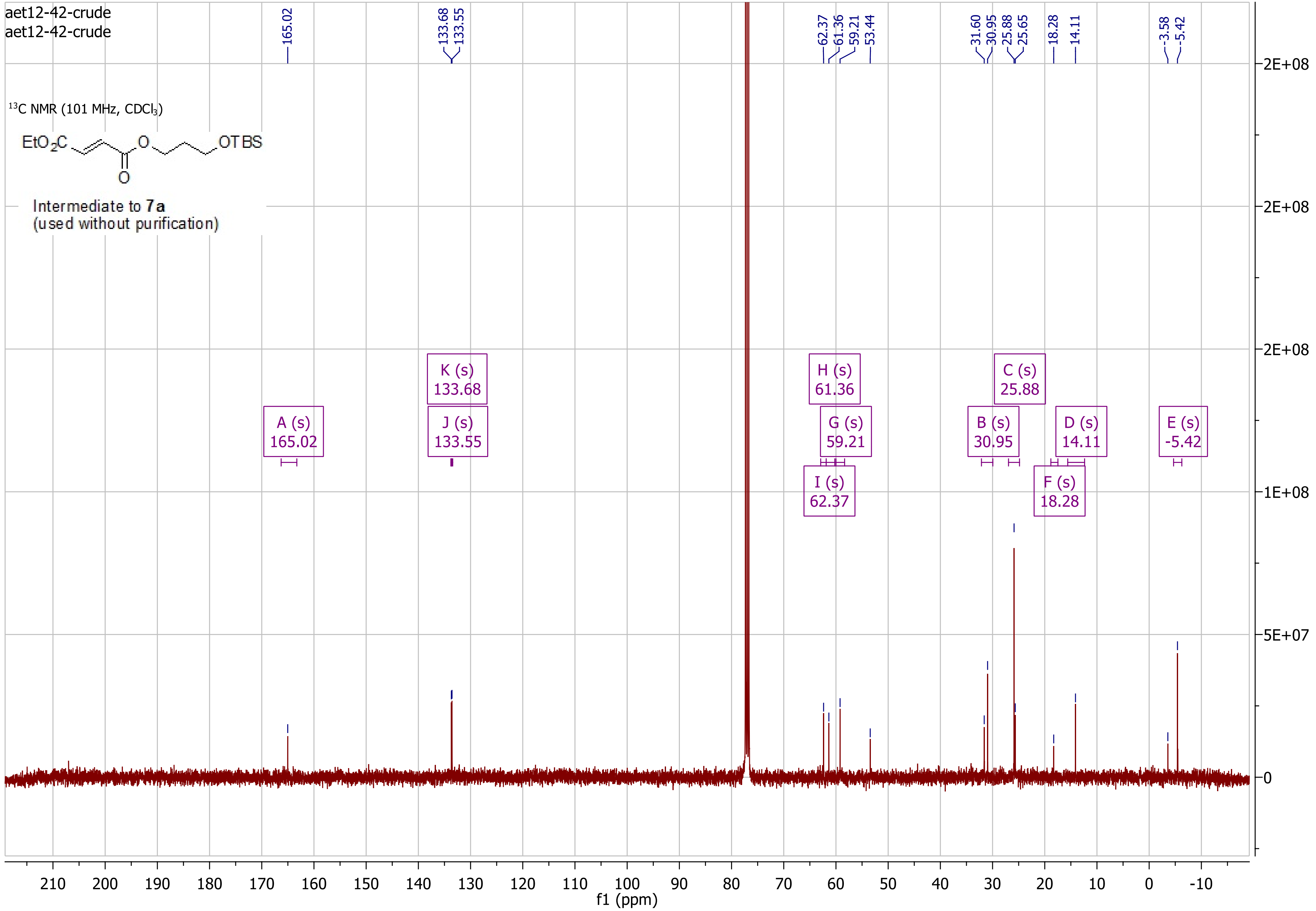
aet12-42-crude
aet12-42-crude
13C NMR (101 MHz, CDCl3)
EtO2C
O
OTBS
O
Intermediate to 7a
(used without purification)
165.02
133.68
133.55
62.37
61.36
59.21
53.44
31.60
30.95
25.88
25.65
18.28
14.11
-3.58
-5.42
A (s) 165.02
K (s) 133.68
J (s) 133.55
H (s) 61.36
G (s) 59.21
I (s) 62.37
C (s) 25.88
B (s) 30.95
D (s) 14.11
F (s) 18.28
E (s) -5.42
2E+08
2E+08
2E+08
1E+08
5E+07
0
210 200 190 180 170 160 150 140 130 120 110 100 90 80 70 60 50 40 30 20 10 0 -10
f1 (ppm)

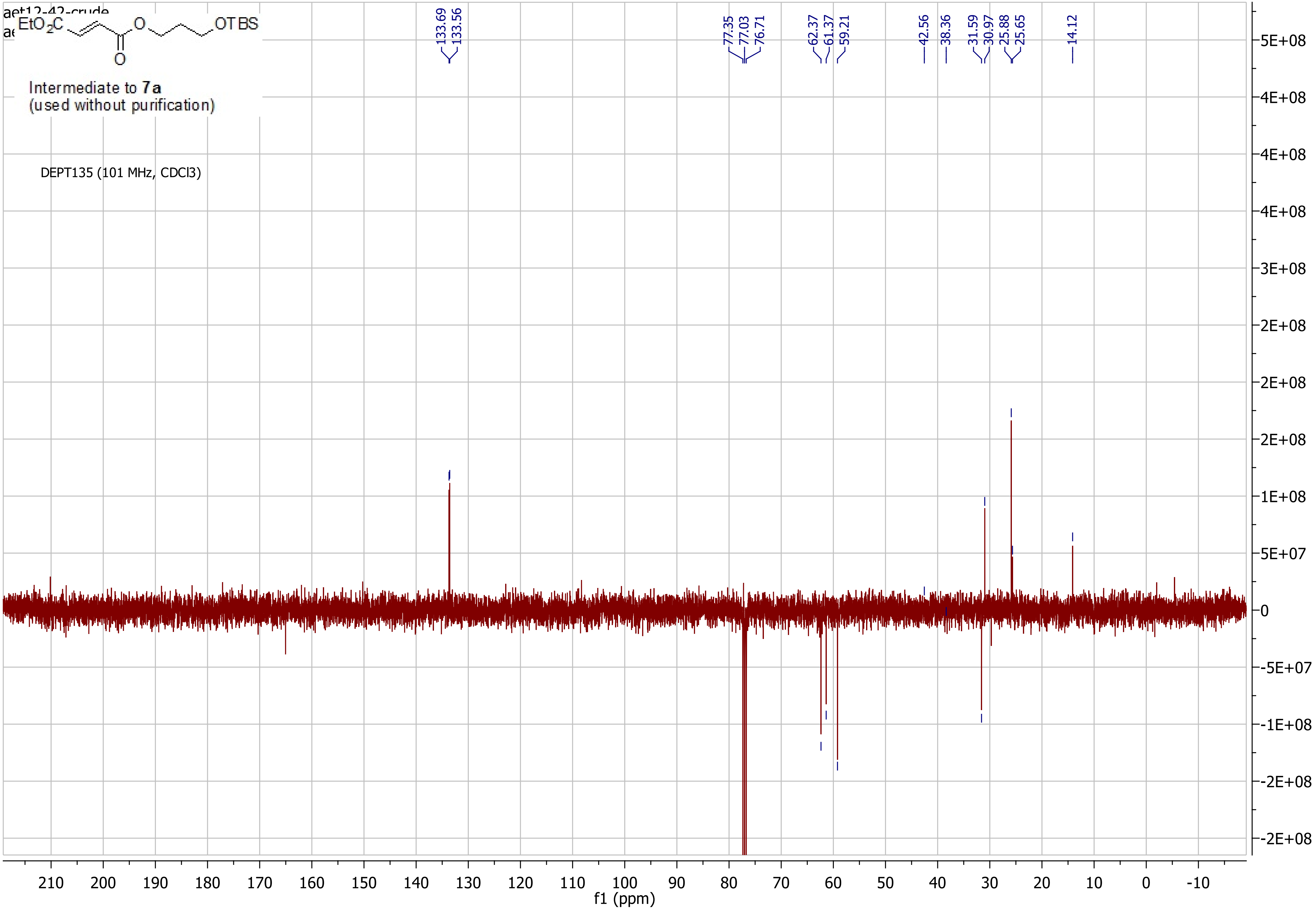
aet12-42-crude
EtO2C
O
OTBS
O
Intermediate to 7a
(used without purification)
DEPT135 (101 MHz, CDCl3)
133.69
133.56
77.35
77.03
76.71
62.37
61.37
59.21
42.56
38.36
31.59
30.97
25.88
25.65
14.12
f1 (ppm)

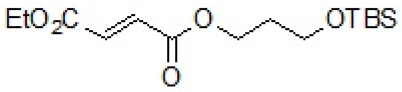

Intermediate to **7a**
(used without purification)

COSY (400 MHz, CDCl3)

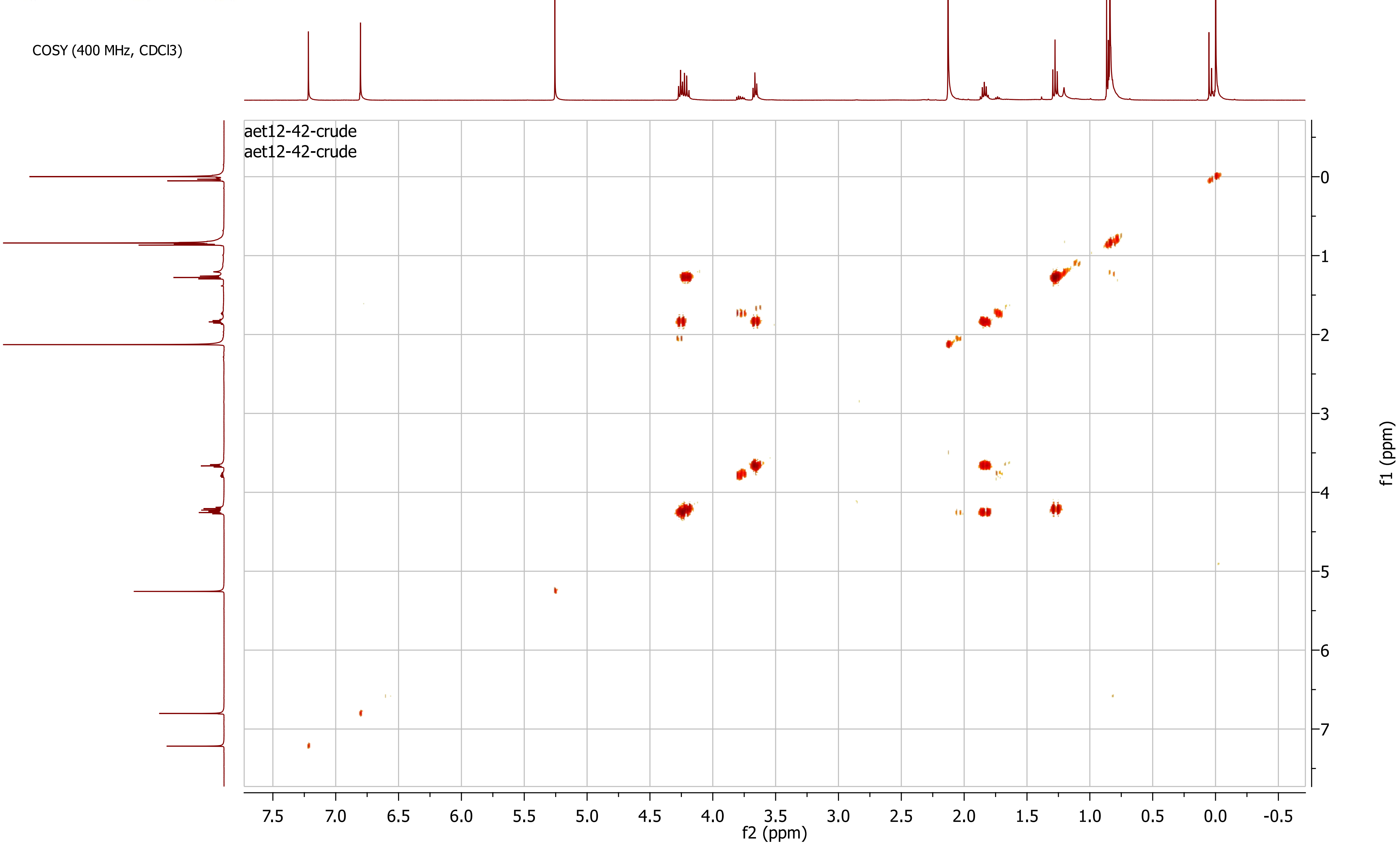

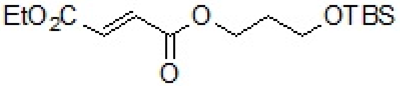

Intermediate to **7a**
(used without purification)

HMQC (CDCl3)

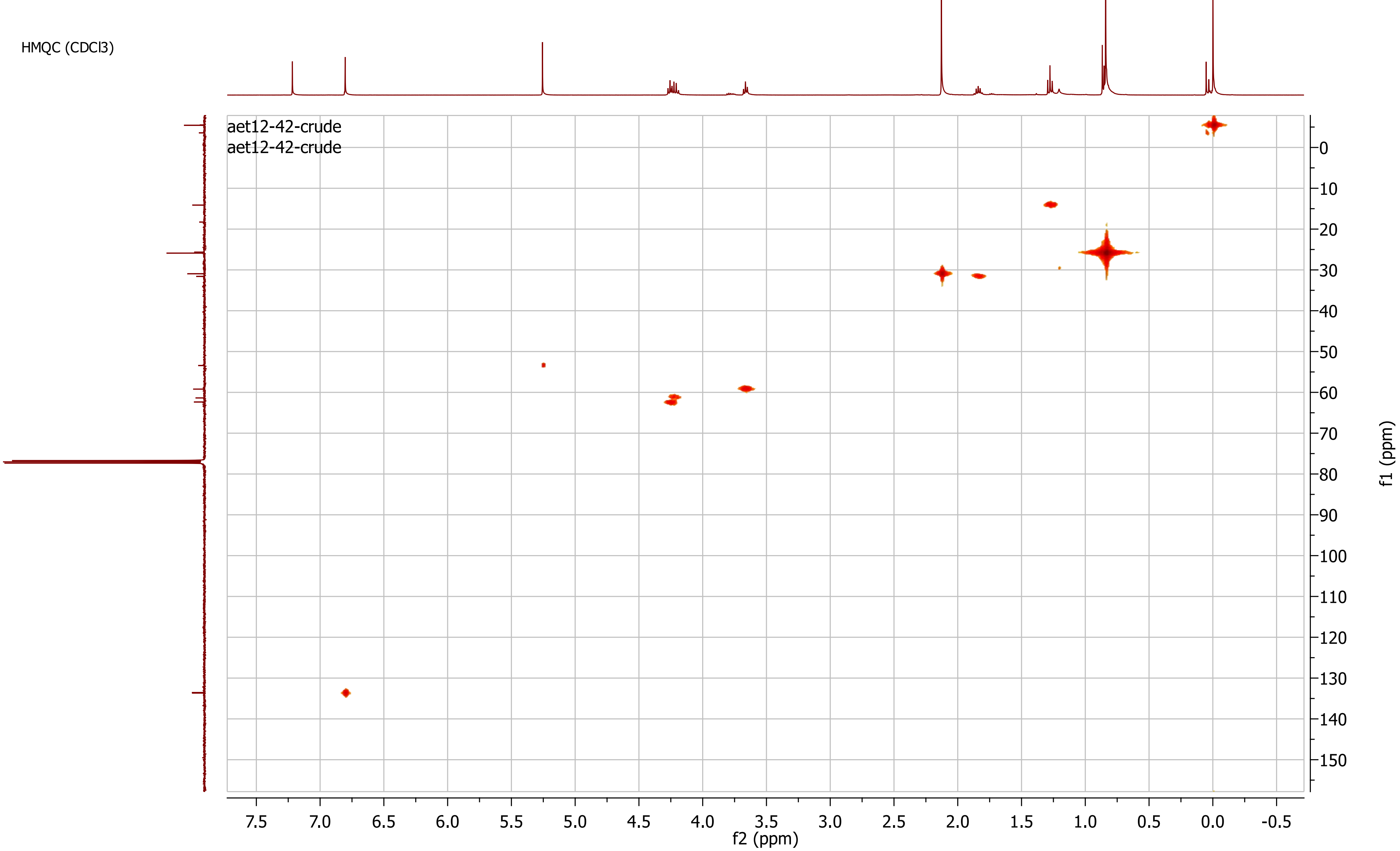

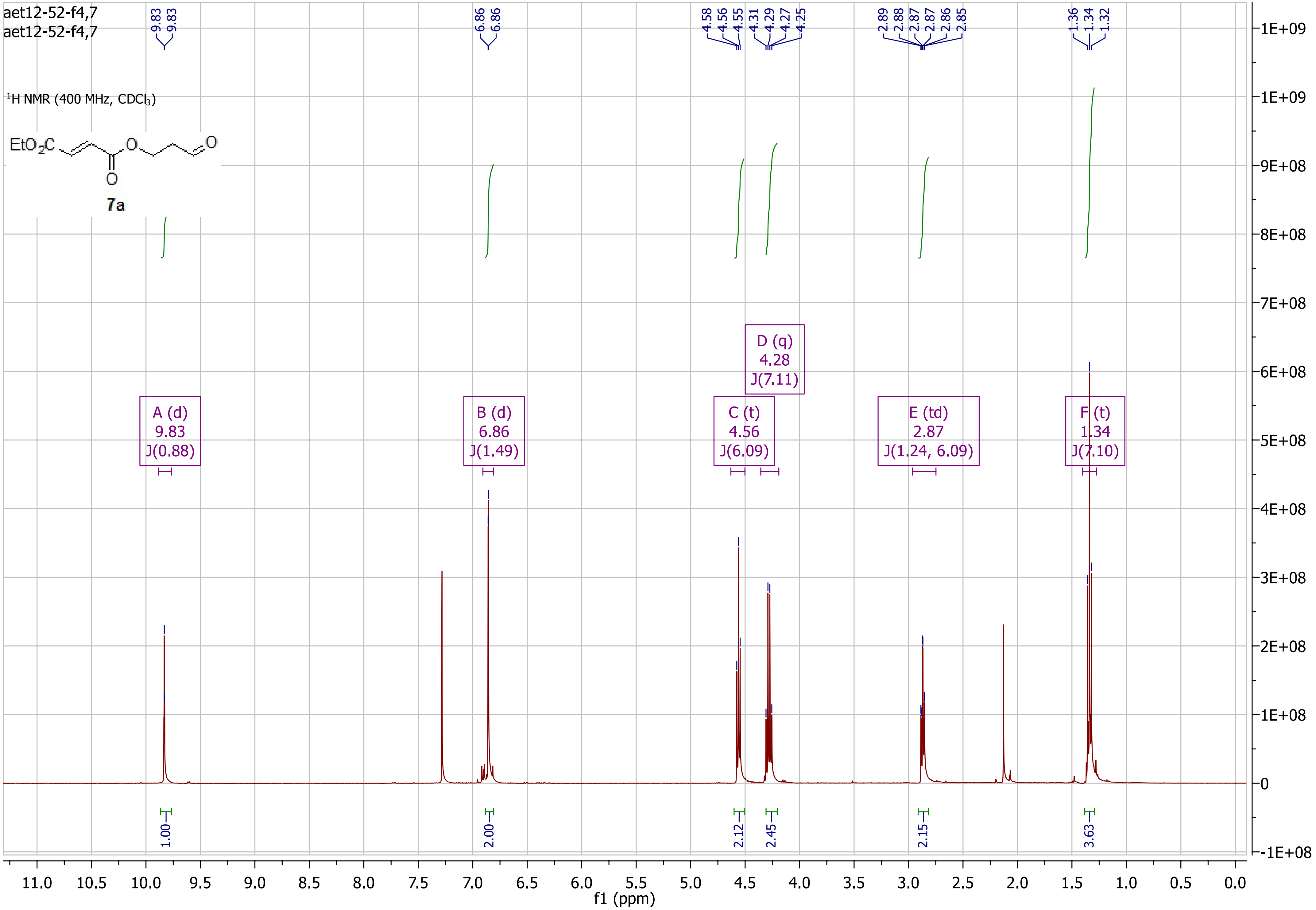
aet12-52-f4,7
aet12-52-f4,7
¹H NMR (400 MHz, CDCl3)
EtO2C O O
O
7a
9.83 9.83
6.86 6.86
4.58 4.56 4.55 4.31 4.29 4.27 4.25
2.89 2.88 2.87 2.87 2.86 2.85
1.36 1.34 1.32
A (d) 9.83 J(0.88)
B (d) 6.86 J(1.49)
C (t) 4.56 J(6.09)
D (q) 4.28 J(7.11)
E (td) 2.87 J(1.24, 6.09)
F (t) 1.34 J(7.10)
1.00
2.00
2.12
2.45
2.15
3.63
1E+09
1E+09
9E+08
8E+08
7E+08
6E+08
5E+08
4E+08
3E+08
2E+08
1E+08
0
-1E+08
11.0 10.5 10.0 9.5 9.0 8.5 8.0 7.5 7.0 6.5 6.0 5.5 5.0 4.5 4.0 3.5 3.0 2.5 2.0 1.5 1.0 0.5 0.0
f1 (ppm)

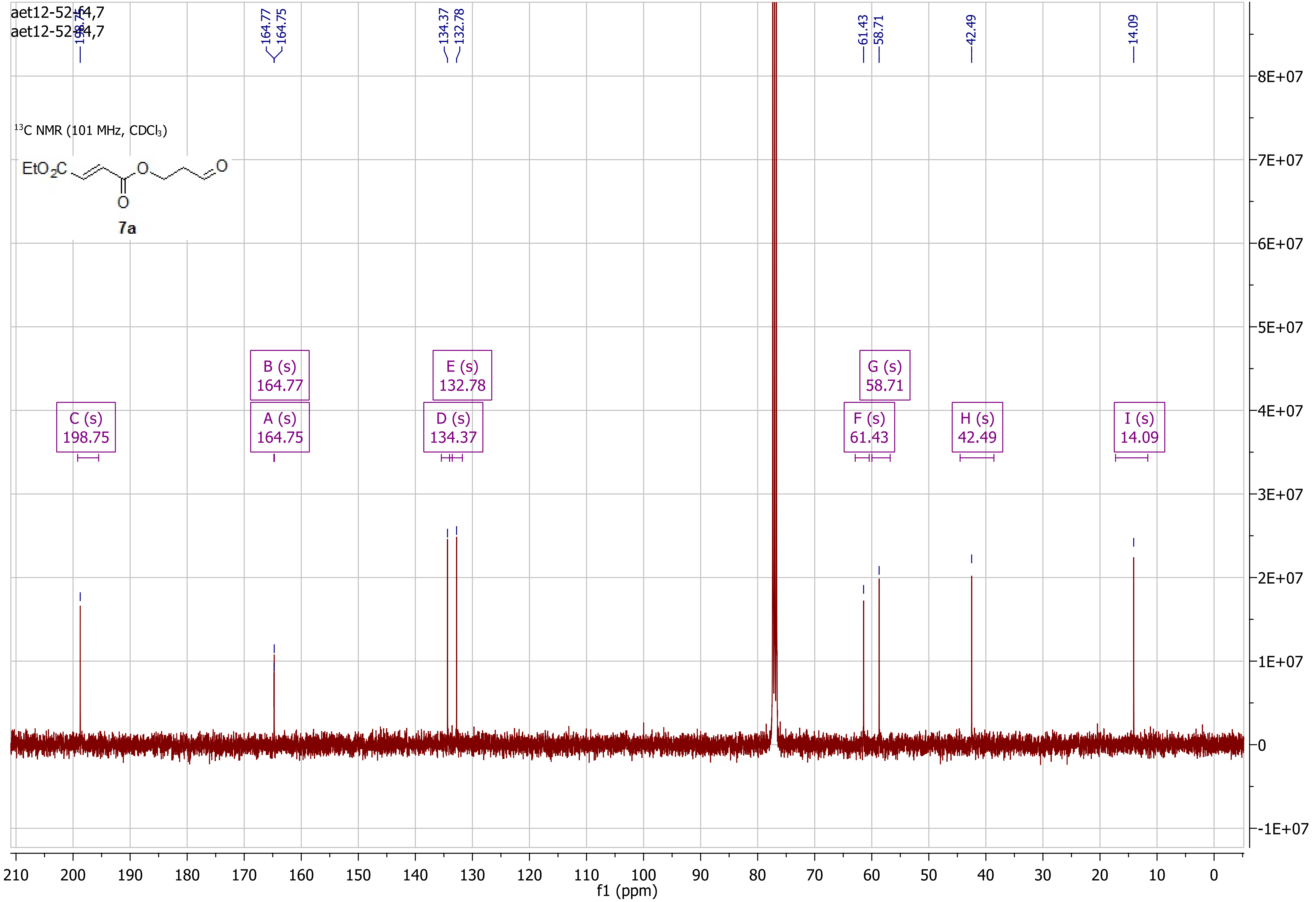

aet12-52-f4,7
aet12-52-f4,7
198.75
164.77
164.75
134.37
132.78
61.43
58.71
42.49
14.09
^{13}C NMR (101 MHz, $CDCl_3$)
EtO_2C
O
O
O
7a
C (s) 198.75
B (s) 164.77
A (s) 164.75
E (s) 132.78
D (s) 134.37
G (s) 58.71
F (s) 61.43
H (s) 42.49
I (s) 14.09
8E+07
7E+07
6E+07
5E+07
4E+07
3E+07
2E+07
1E+07
0
-1E+07
210 200 190 180 170 160 150 140 130 120 110 100 90 80 70 60 50 40 30 20 10 0
f1 (ppm)

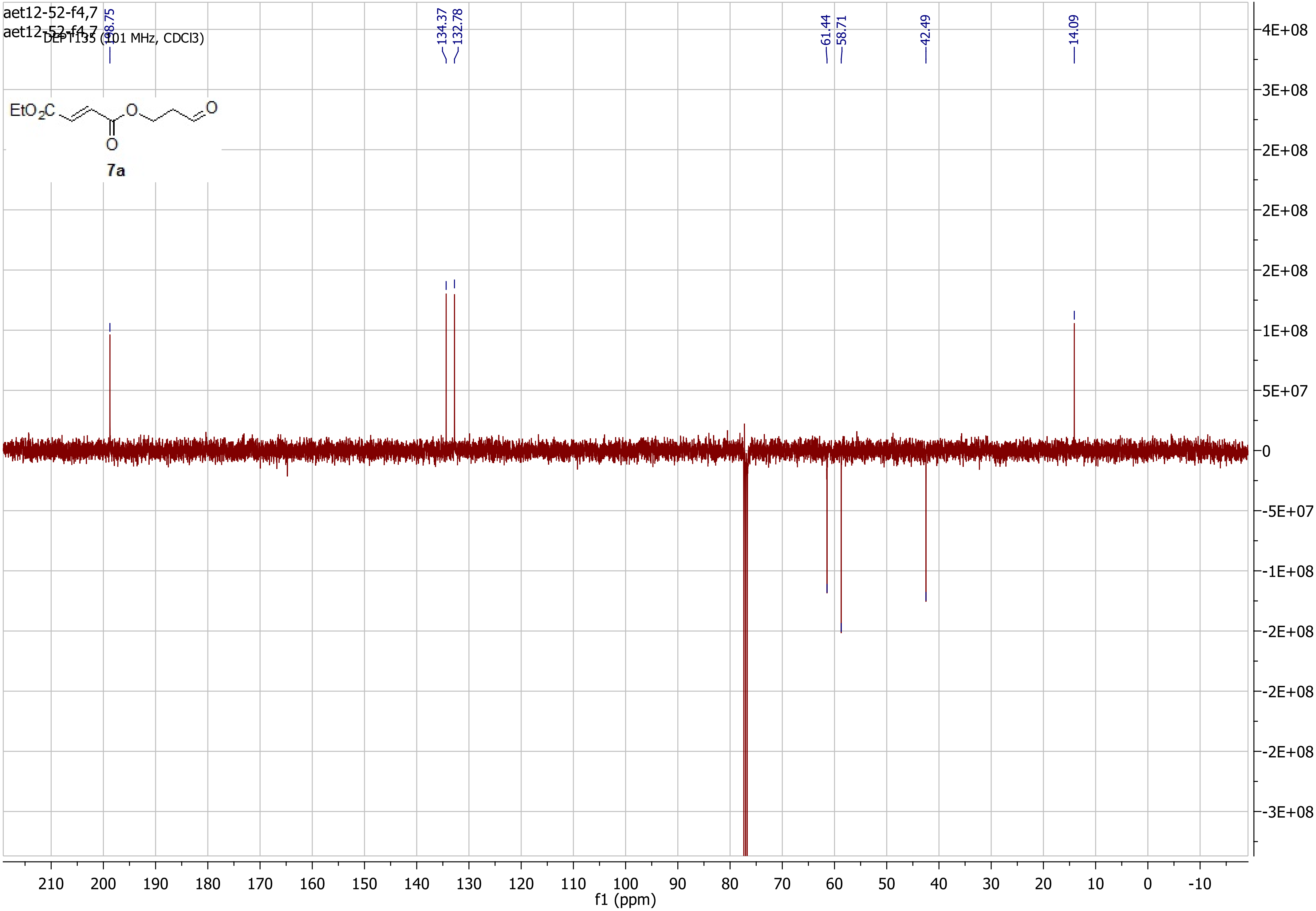
aet12-52-f4,7
aet12-52-f4,7
DEPT135 (101 MHz, CDCl3)
198.75
134.37
132.78
61.44
58.71
42.49
14.09
EtO2C
O
O
O
7a
4E+08
3E+08
2E+08
2E+08
2E+08
1E+08
5E+07
0
-5E+07
-1E+08
-2E+08
-2E+08
-2E+08
-3E+08
210 200 190 180 170 160 150 140 130 120 110 100 90 80 70 60 50 40 30 20 10 0 -10
f1 (ppm)

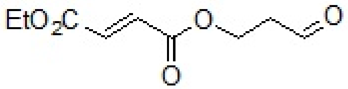

7a

COSY (400 MHz, CDCl3)

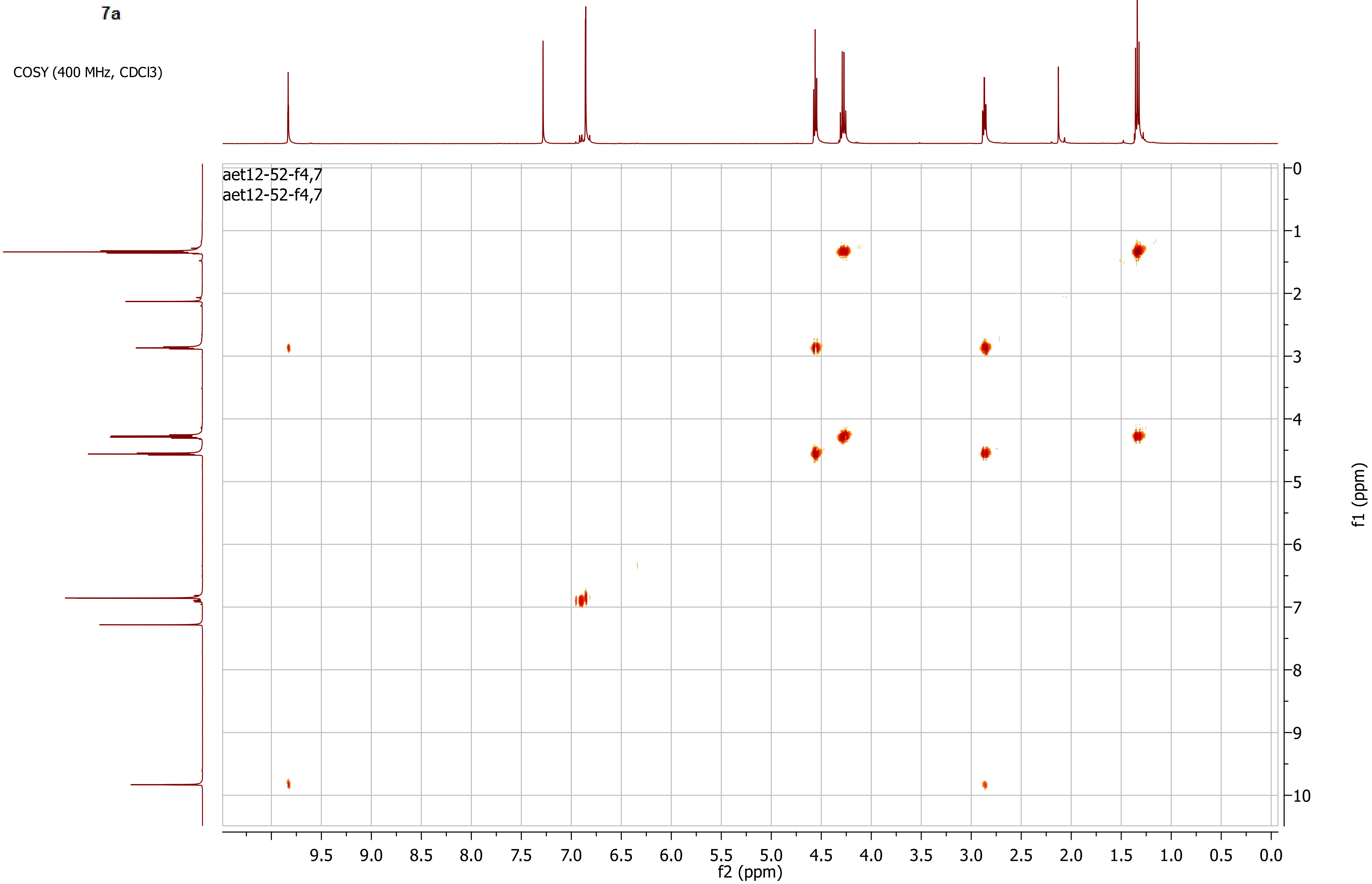

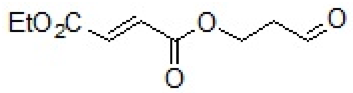

7a

HMQC (CDCl3)

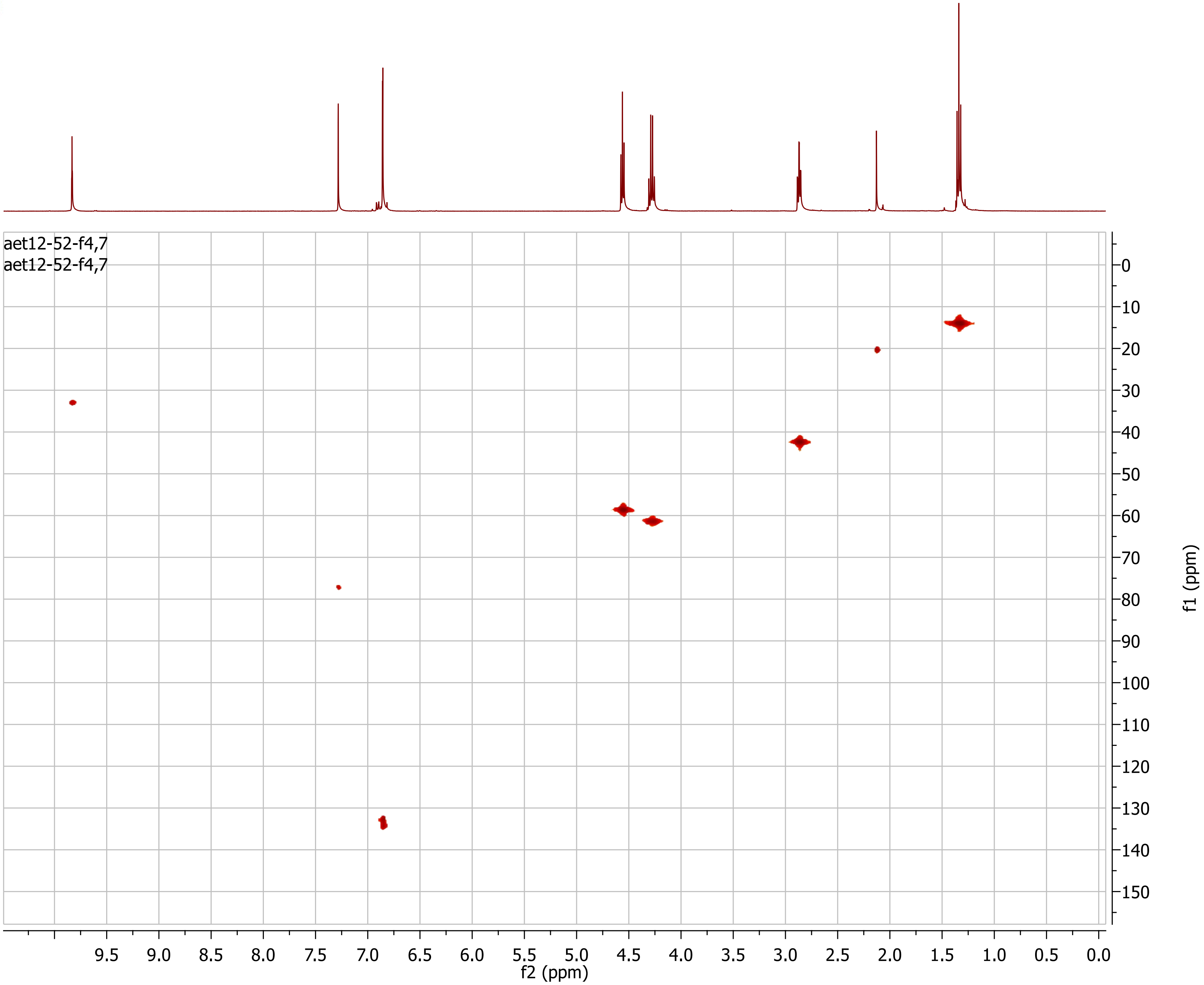

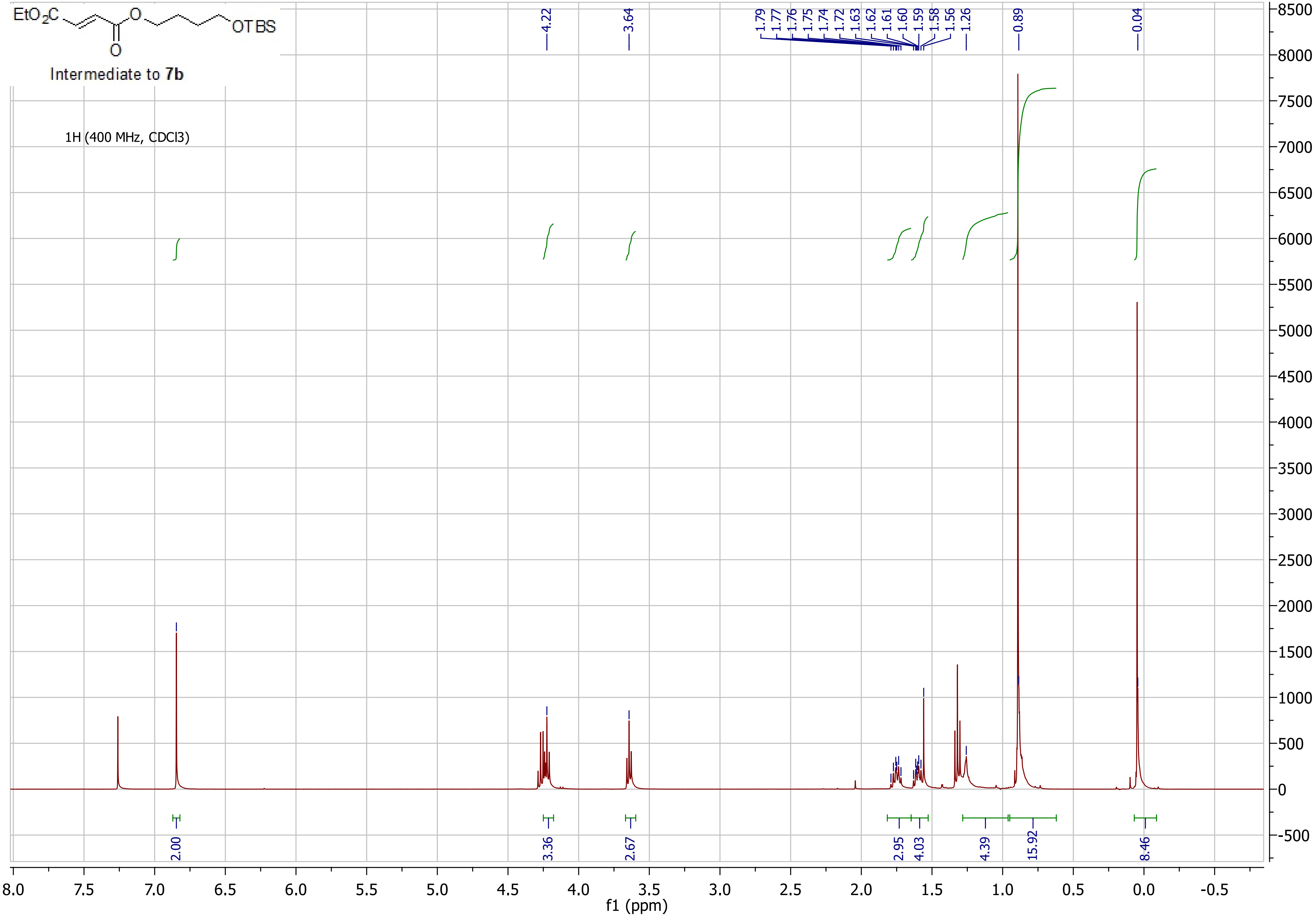
EtO2C
O
O
OTBS
Intermediate to 7b
1H (400 MHz, CDCl3)
4.22
3.64
1.79
1.77
1.76
1.75
1.74
1.72
1.63
1.62
1.61
1.60
1.59
1.58
1.56
1.26
0.89
0.04
2.00
3.36
2.67
2.95
4.03
4.39
15.92
8.46
f1 (ppm)

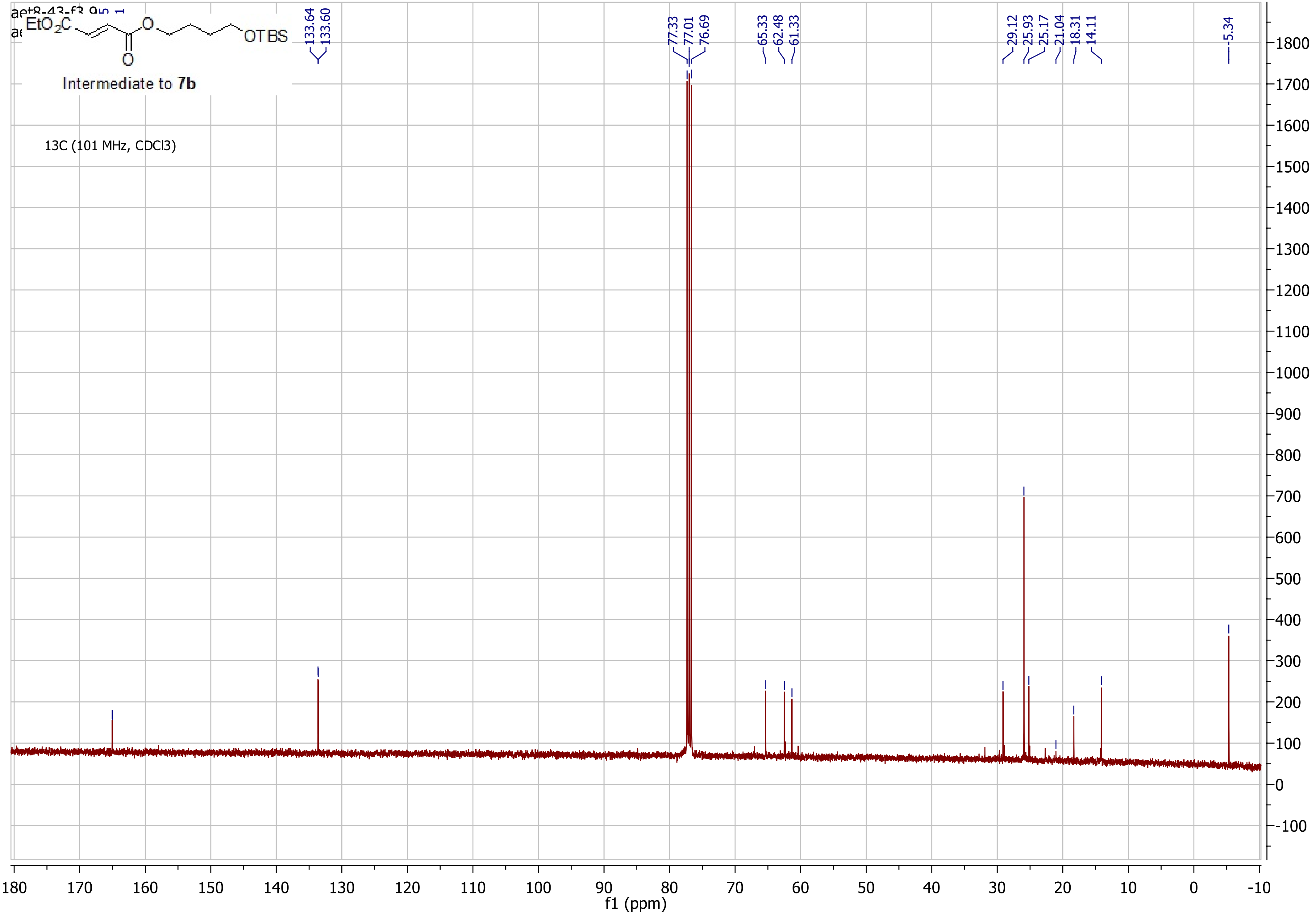

EtO2C
O
OTBS
Intermediate to 7b
13C (101 MHz, CDCl3)
133.64
133.60
77.33
77.01
76.69
65.33
62.48
61.33
29.12
25.93
25.17
21.04
18.31
14.11
-5.34
f1 (ppm)

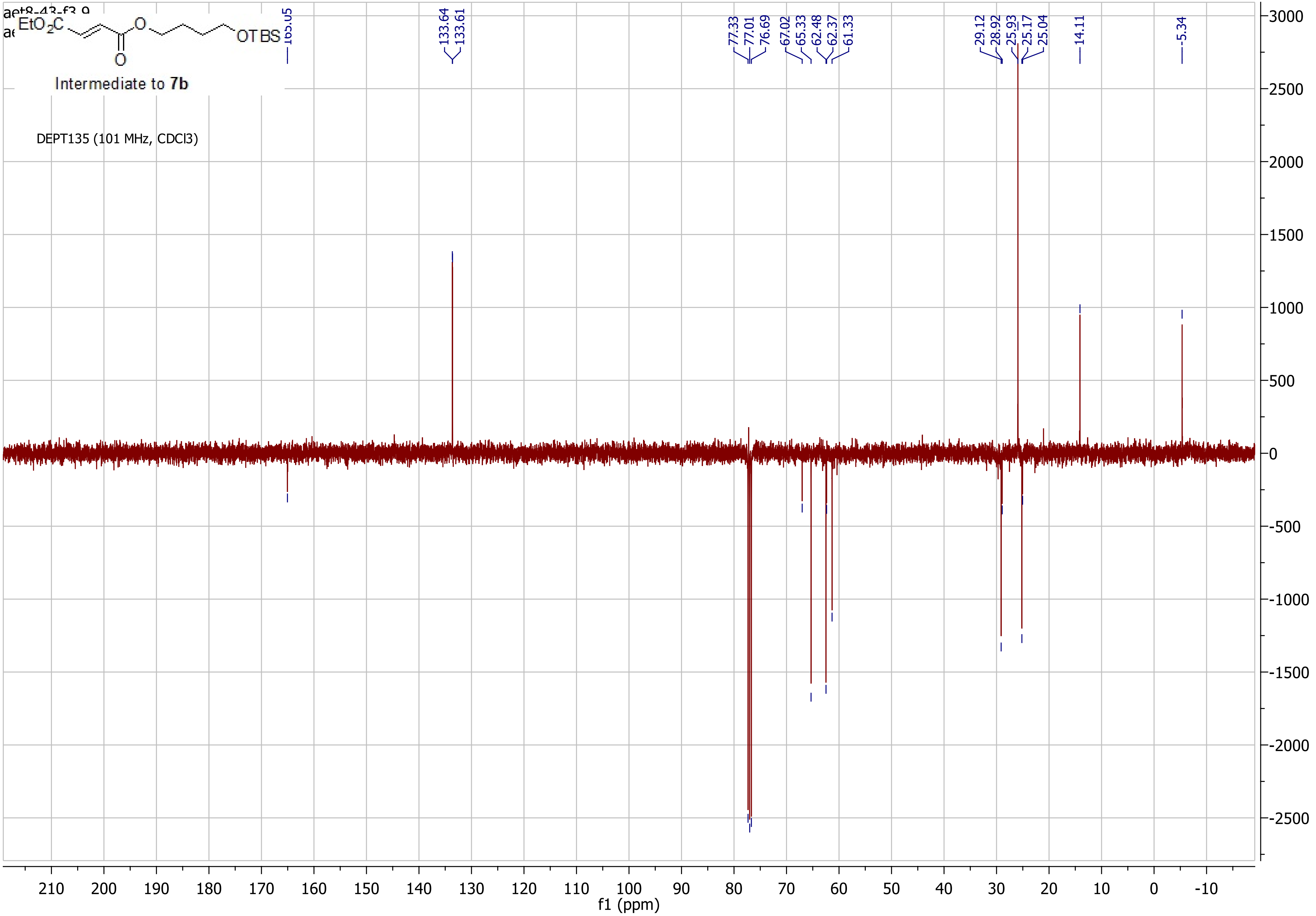

EtO2C
O
OTBS
O
Intermediate to 7b
DEPT135 (101 MHz, CDCl3)
133.64
133.61
77.33
77.01
76.69
67.02
65.33
62.48
62.37
61.33
29.12
28.92
25.93
25.17
25.04
14.11
-5.34
f1 (ppm)

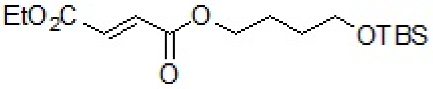

Intermediate to **7b**

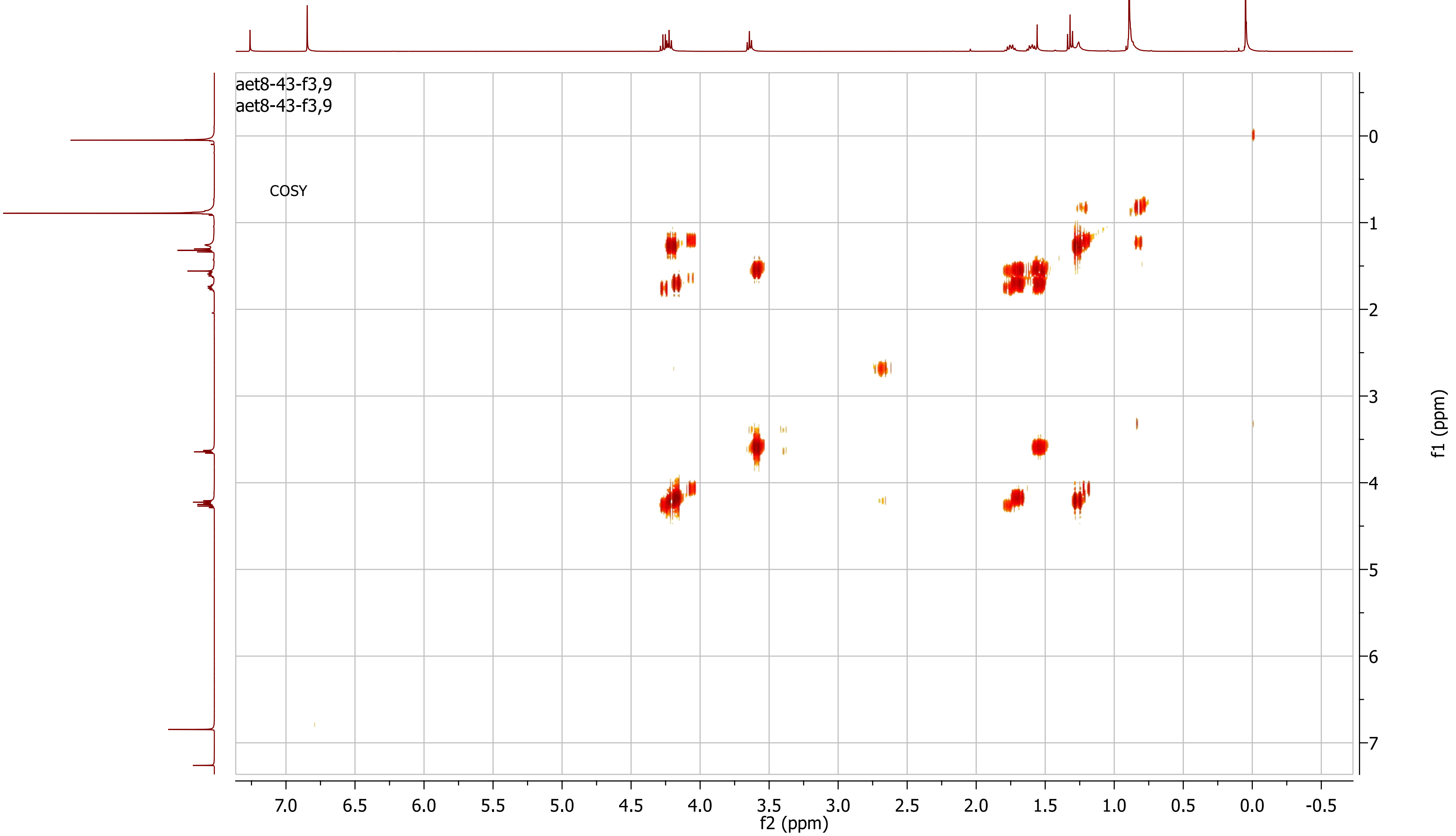

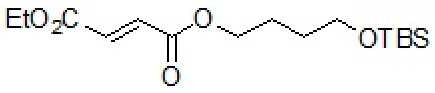

Intermediate to **7b**

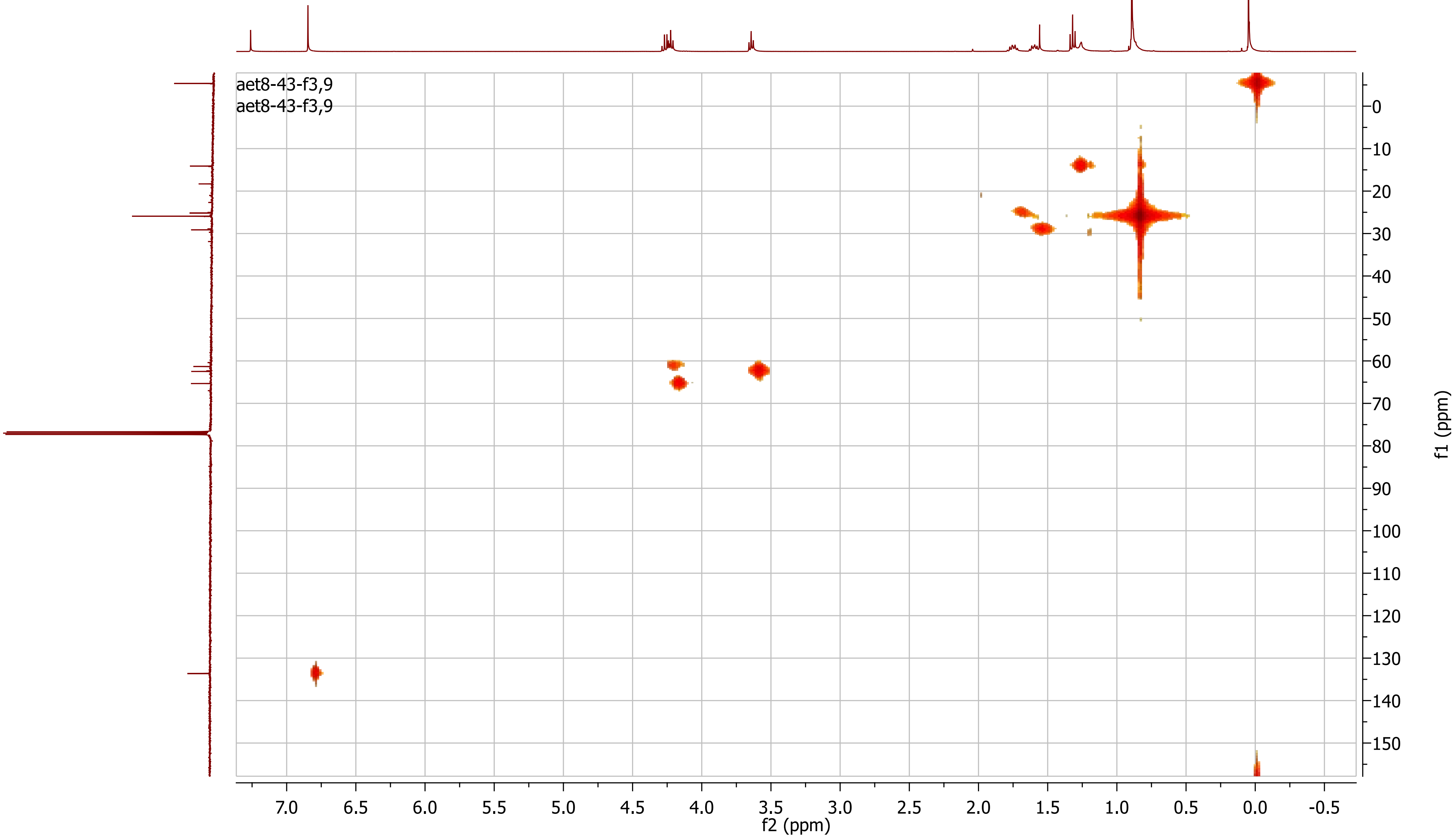

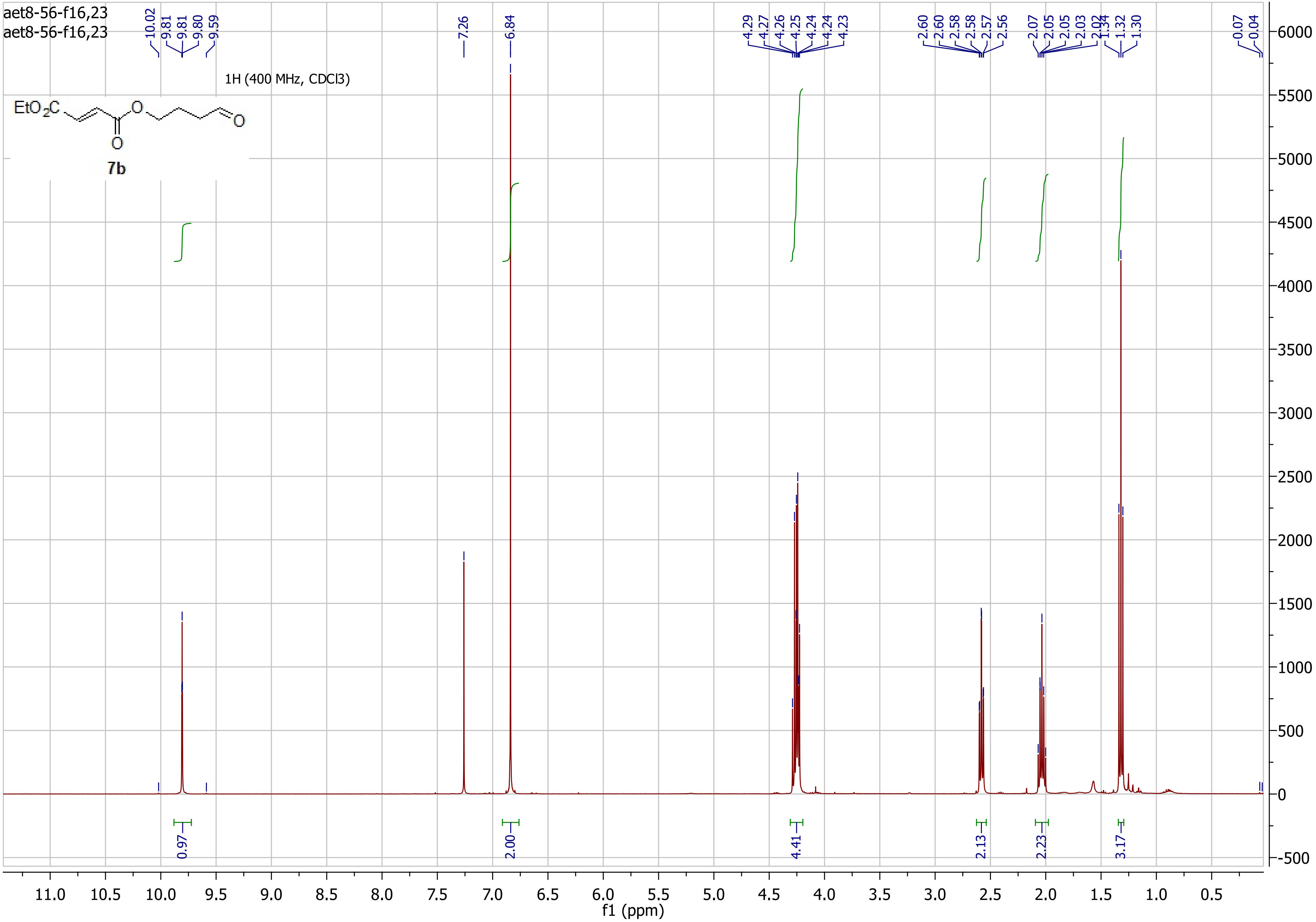
aet8-56-f16,23
aet8-56-f16,23
1H (400 MHz, CDCl3)
EtO2C
O
O
O
7b
10.02 9.81 9.81 9.80 9.59
7.26
6.84
4.29 4.27 4.26 4.25 4.24 4.24 4.23
2.60 2.60 2.58 2.58 2.57 2.56
2.07 2.05 2.05 2.03 2.02
1.34 1.32 1.30
0.07 0.04
0.97
2.00
4.41
2.13
2.23
3.17
f1 (ppm)

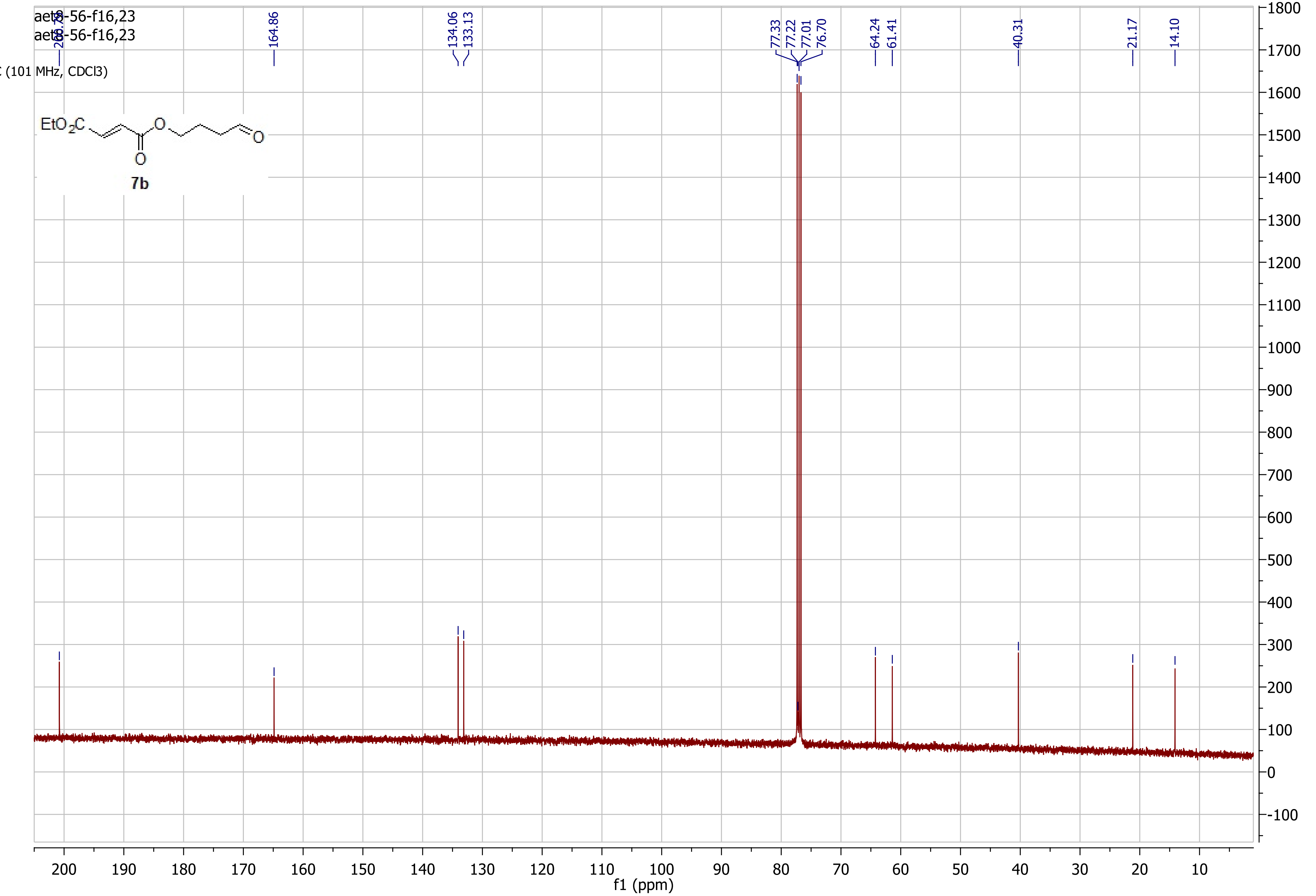

aet8-56-f16,23
aet8-56-f16,23
C (101 MHz, CDCl3)
164.86
134.06
133.13
77.33
77.22
77.01
76.70
64.24
61.41
40.31
21.17
14.10
EtO2C
O
O
O
7b
f1 (ppm)

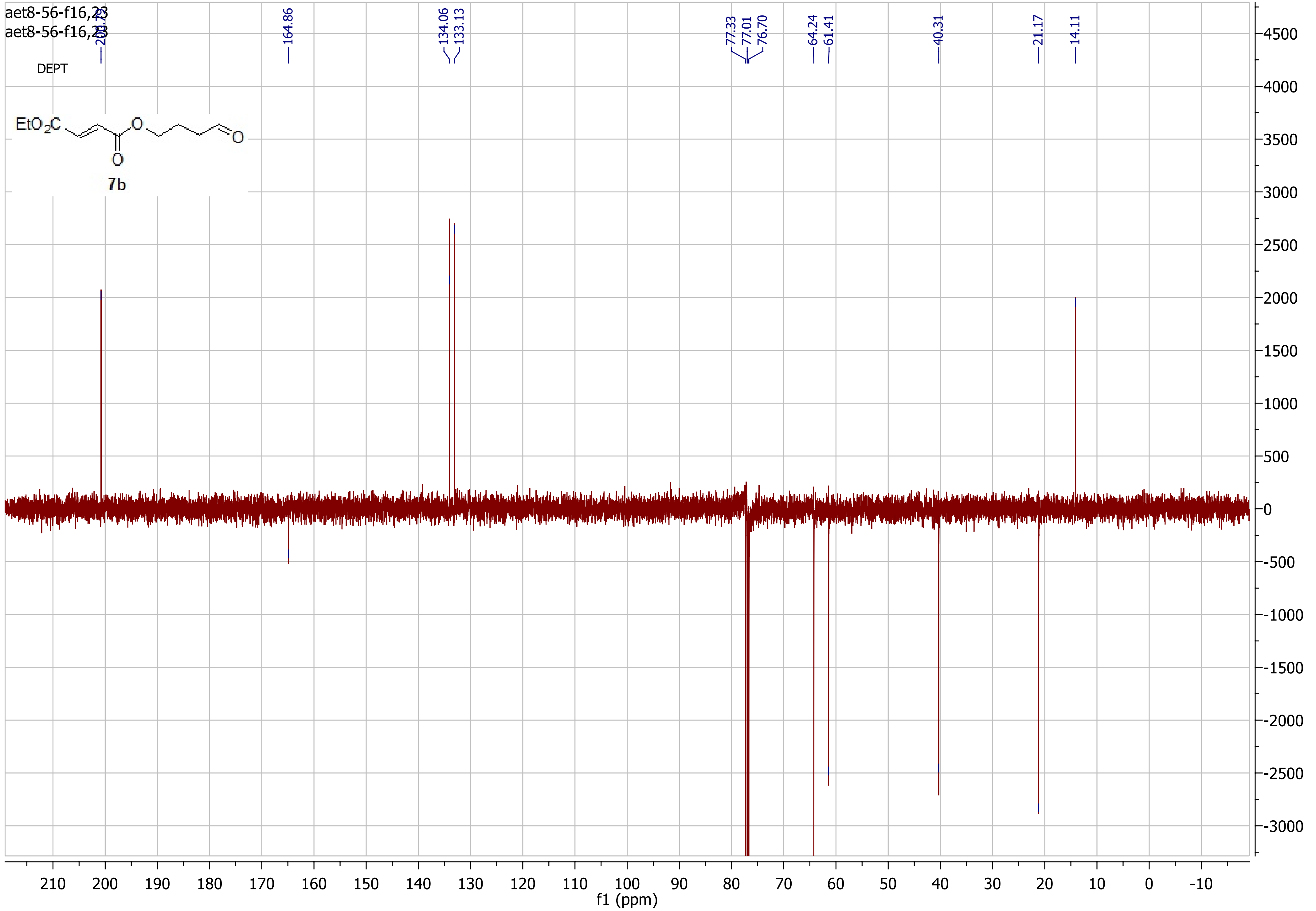

aet8-56-f16,23
aet8-56-f16,23
DEPT
EtO2C
O
O
O
7b
200.79
164.86
134.06
133.13
77.33
77.01
76.70
64.24
61.41
40.31
21.17
14.11
f1 (ppm)

COSY

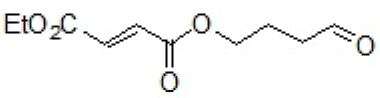
EtO2C
O
O
O

7b

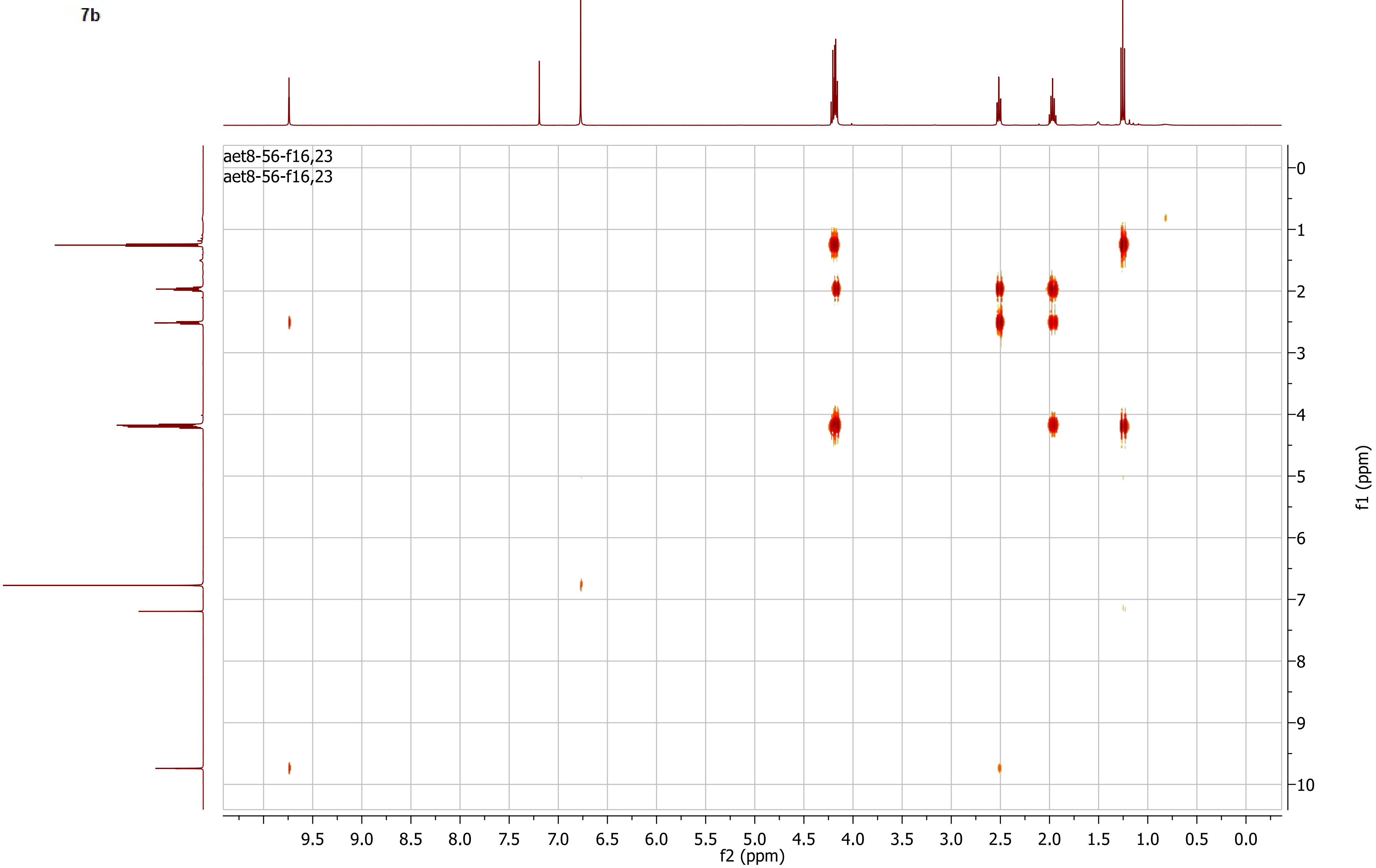
aet8-56-f16,23
aet8-56-f16,23
9.5 9.0 8.5 8.0 7.5 7.0 6.5 6.0 5.5 5.0 4.5 4.0 3.5 3.0 2.5 2.0 1.5 1.0 0.5 0.0
f2 (ppm)
0 1 2 3 4 5 6 7 8 9 10
f1 (ppm)

HMQC

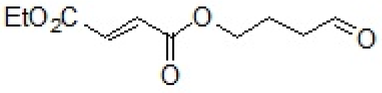

7b

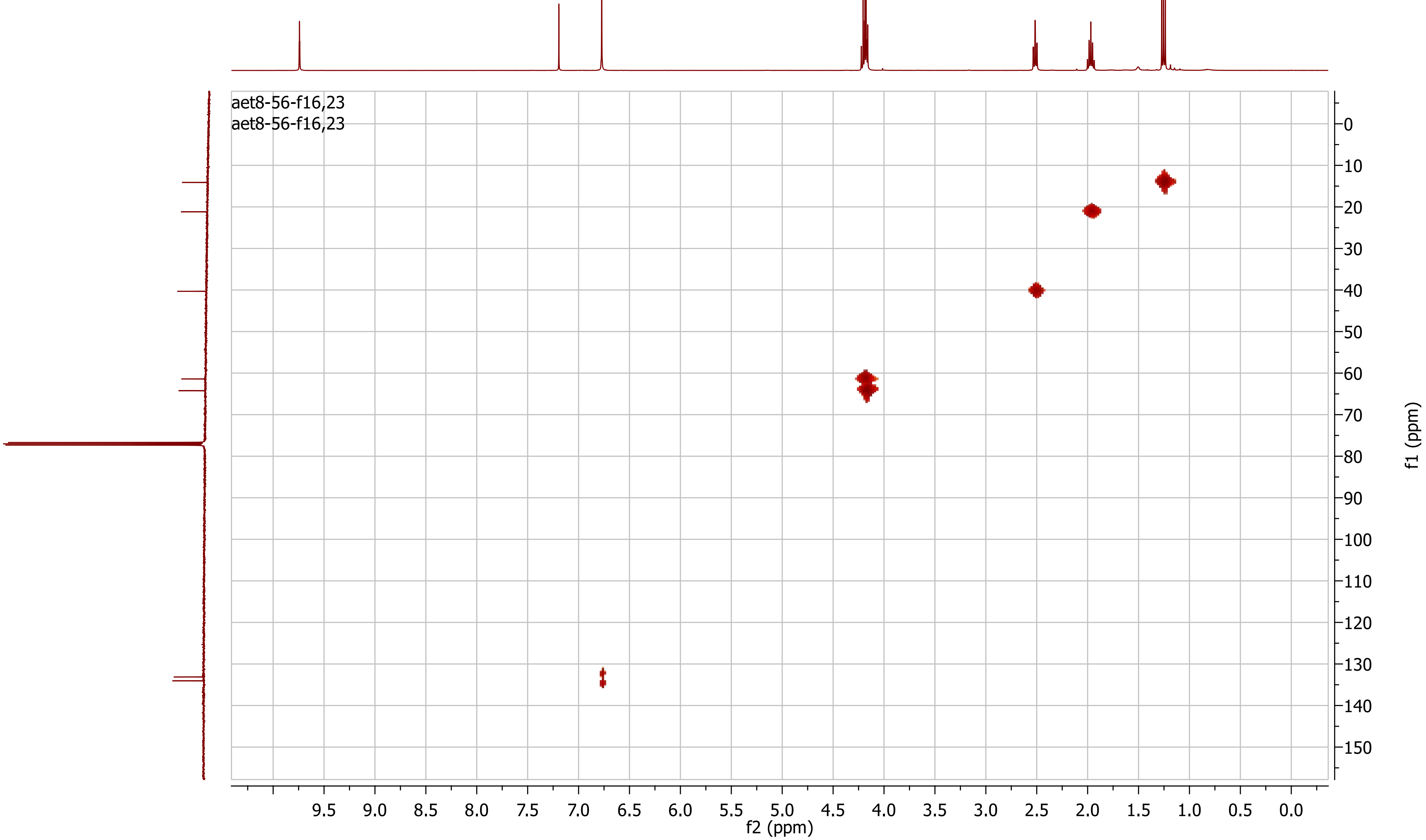

aet12-43-crude
aet12-43-crude

^{1}H NMR (400 MHz, $CDCl_3$)

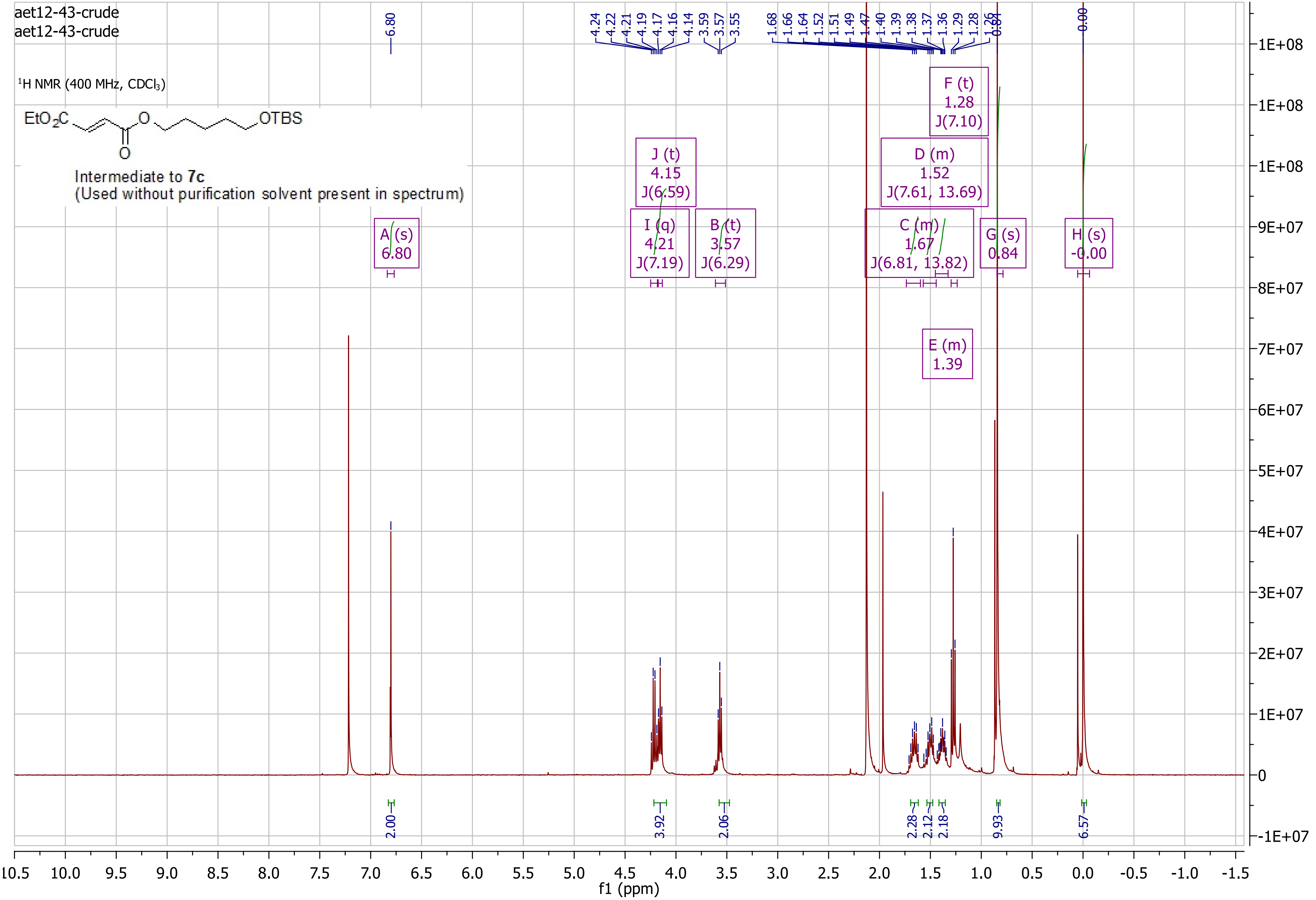

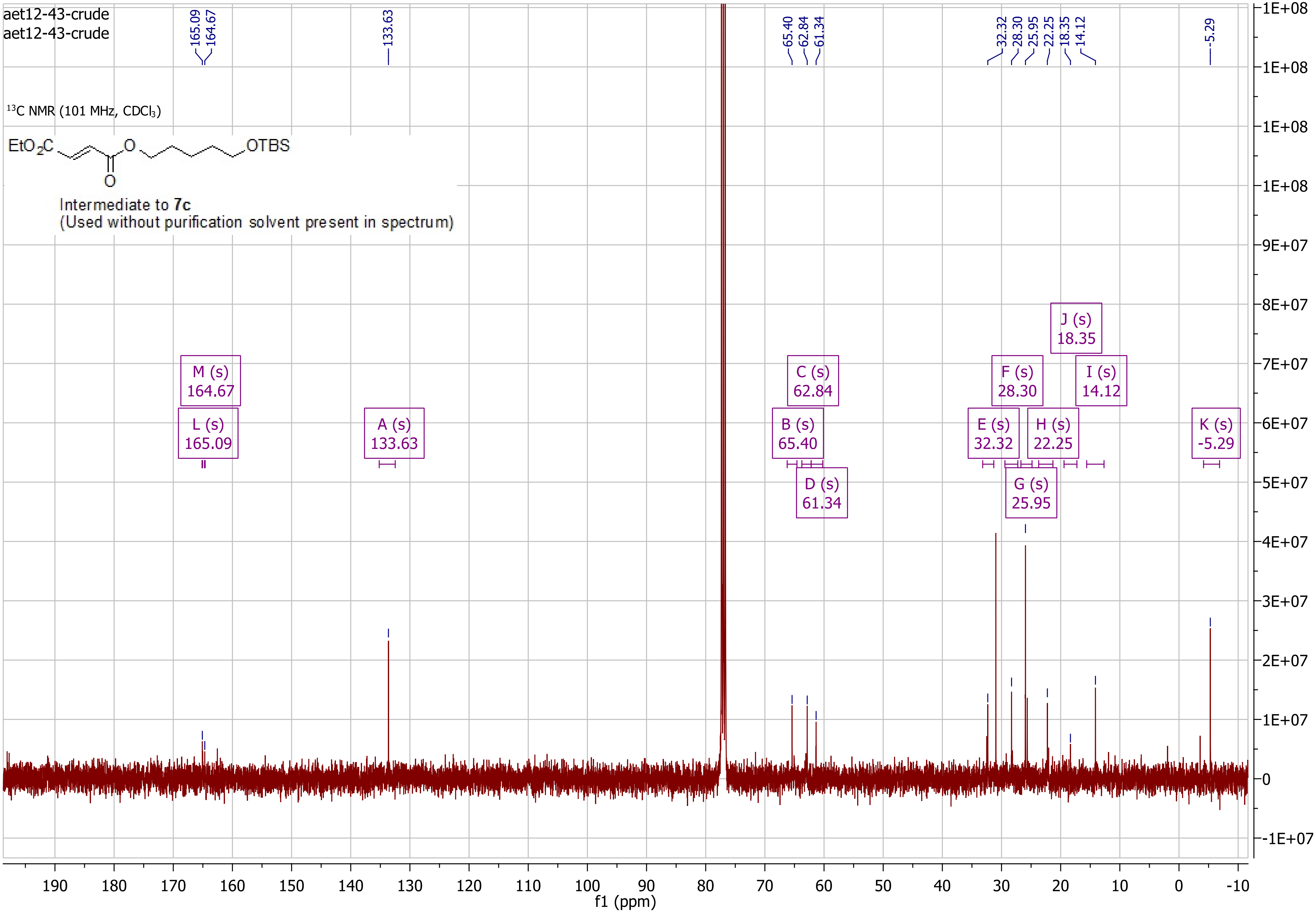
aet12-43-crude
aet12-43-crude
^{13}C NMR (101 MHz, $CDCl_3$)
EtO_2C
O
OTBS
O
Intermediate to **7c**
(Used without purification solvent present in spectrum)
165.09
164.67
133.63
65.40
62.84
61.34
32.32
28.30
25.95
22.25
18.35
14.12
-5.29
M (s) 164.67
L (s) 165.09
A (s) 133.63
C (s) 62.84
B (s) 65.40
D (s) 61.34
F (s) 28.30
E (s) 32.32
G (s) 25.95
H (s) 22.25
J (s) 18.35
I (s) 14.12
K (s) -5.29
f1 (ppm)

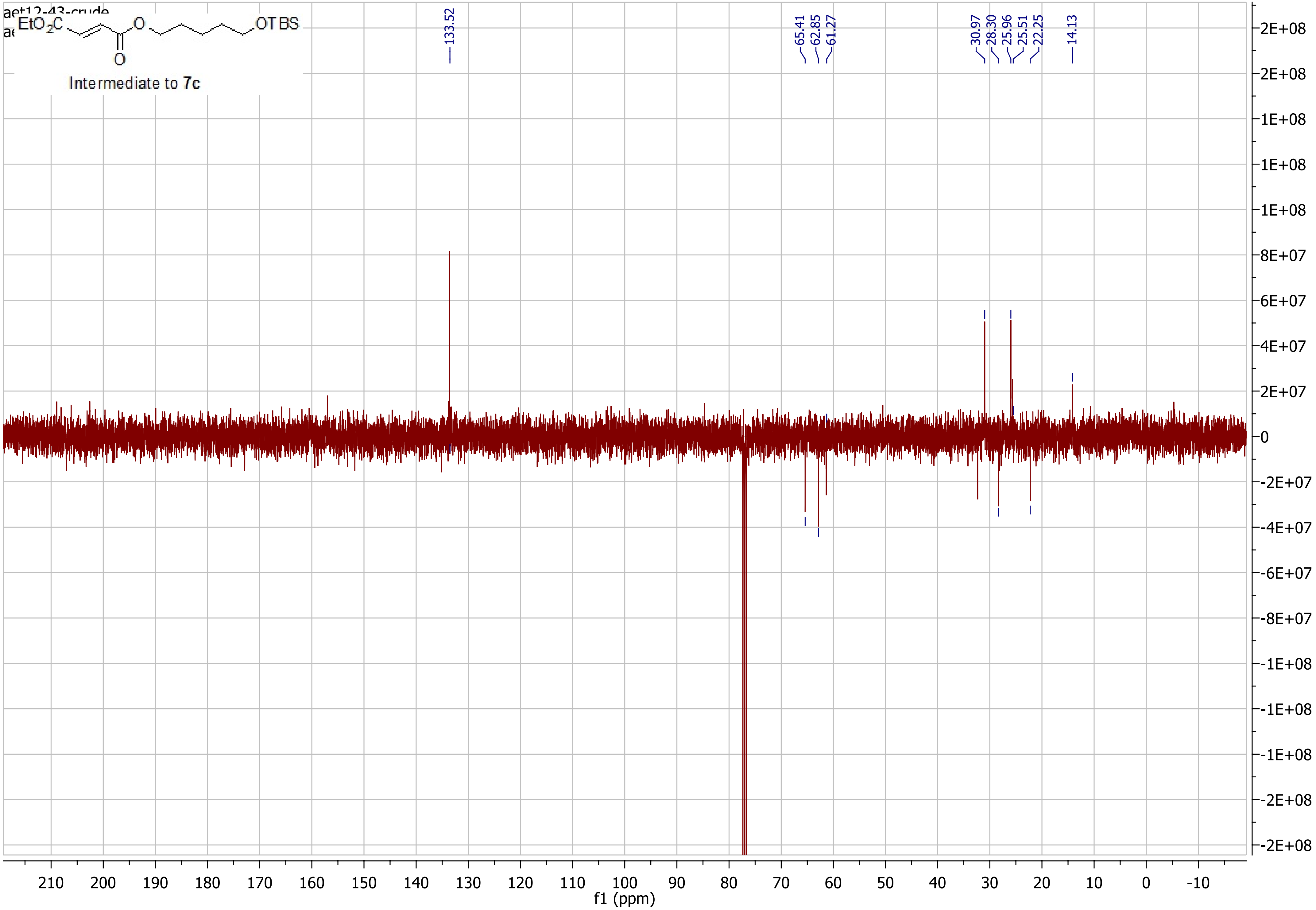
aet12-43-crude
EtO2C
O
O
OTBS
Intermediate to 7c
133.52
65.41
62.85
61.27
30.97
28.30
25.96
25.51
22.25
14.13
2E+08
1E+08
8E+07
6E+07
4E+07
2E+07
0
-2E+07
-4E+07
-6E+07
-8E+07
-1E+08
-2E+08
210
200
190
180
170
160
150
140
130
120
110
100
90
80
70
60
50
40
30
20
10
0
-10
f1 (ppm)

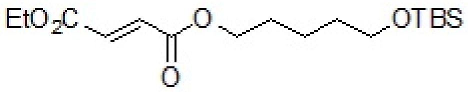

Intermediate to **7c**

COSY (CDCl3)

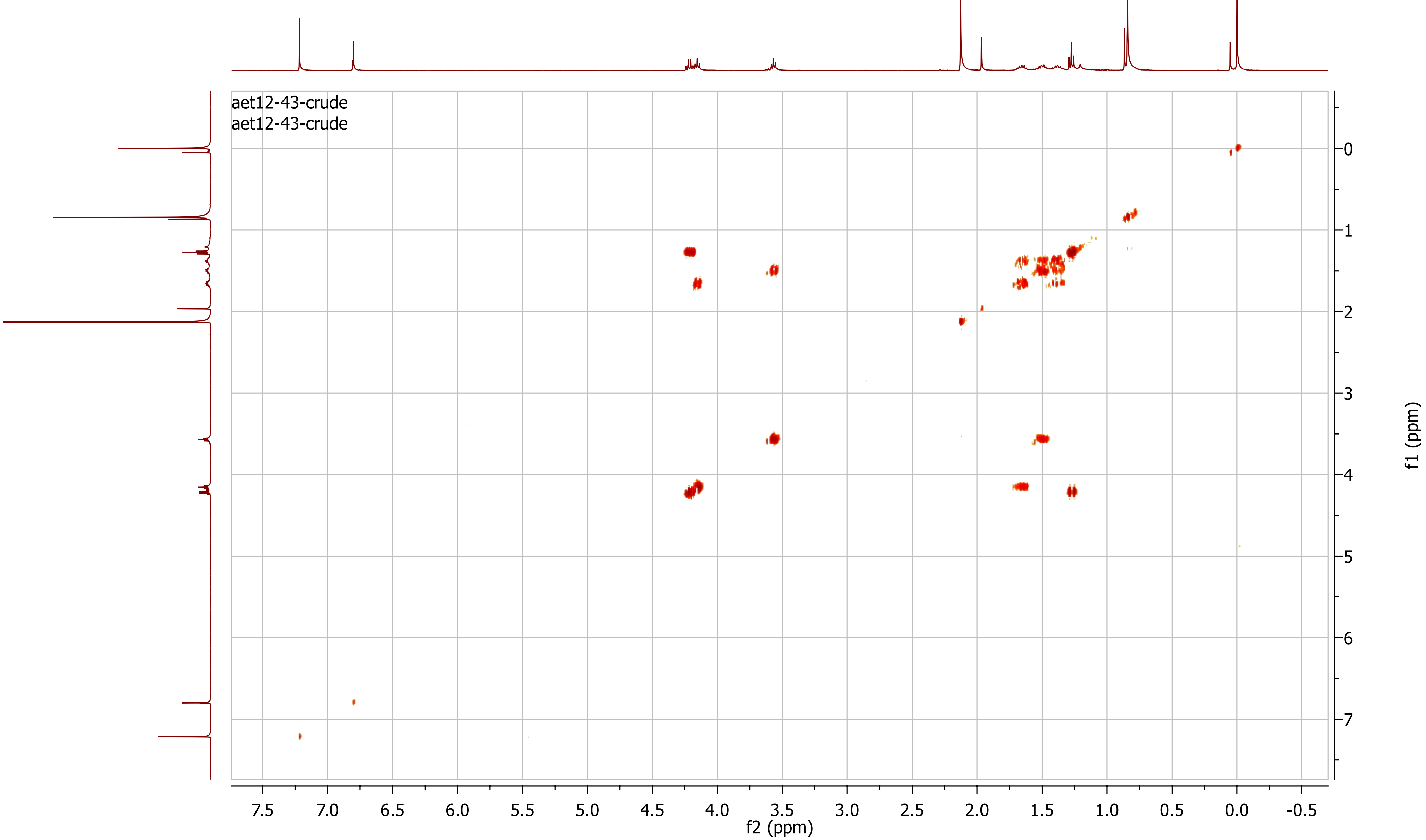

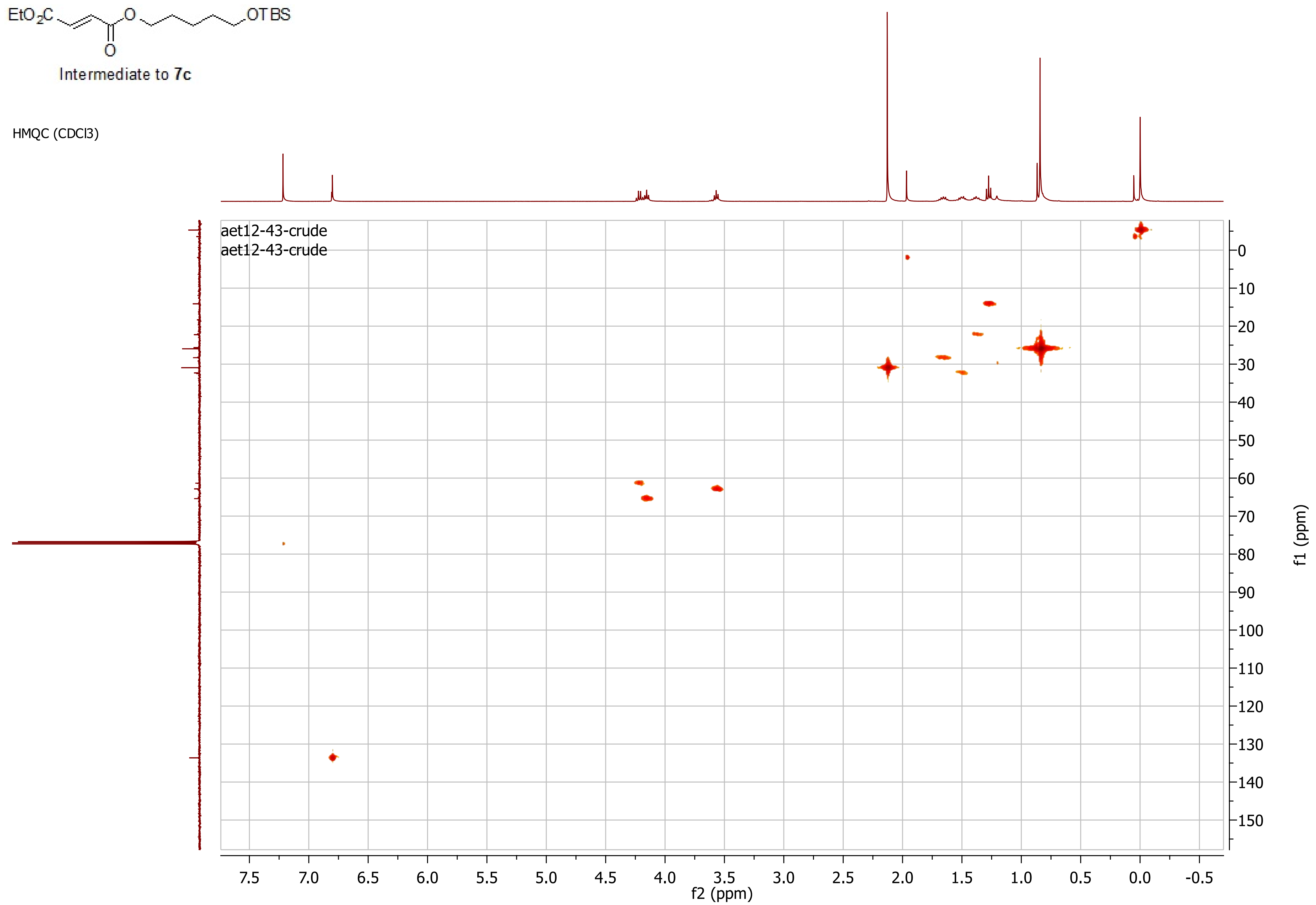
EtO2C
O
OTBS
O
Intermediate to 7c
HMQC (CDCl3)
aet12-43-crude
aet12-43-crude
f2 (ppm)
f1 (ppm)

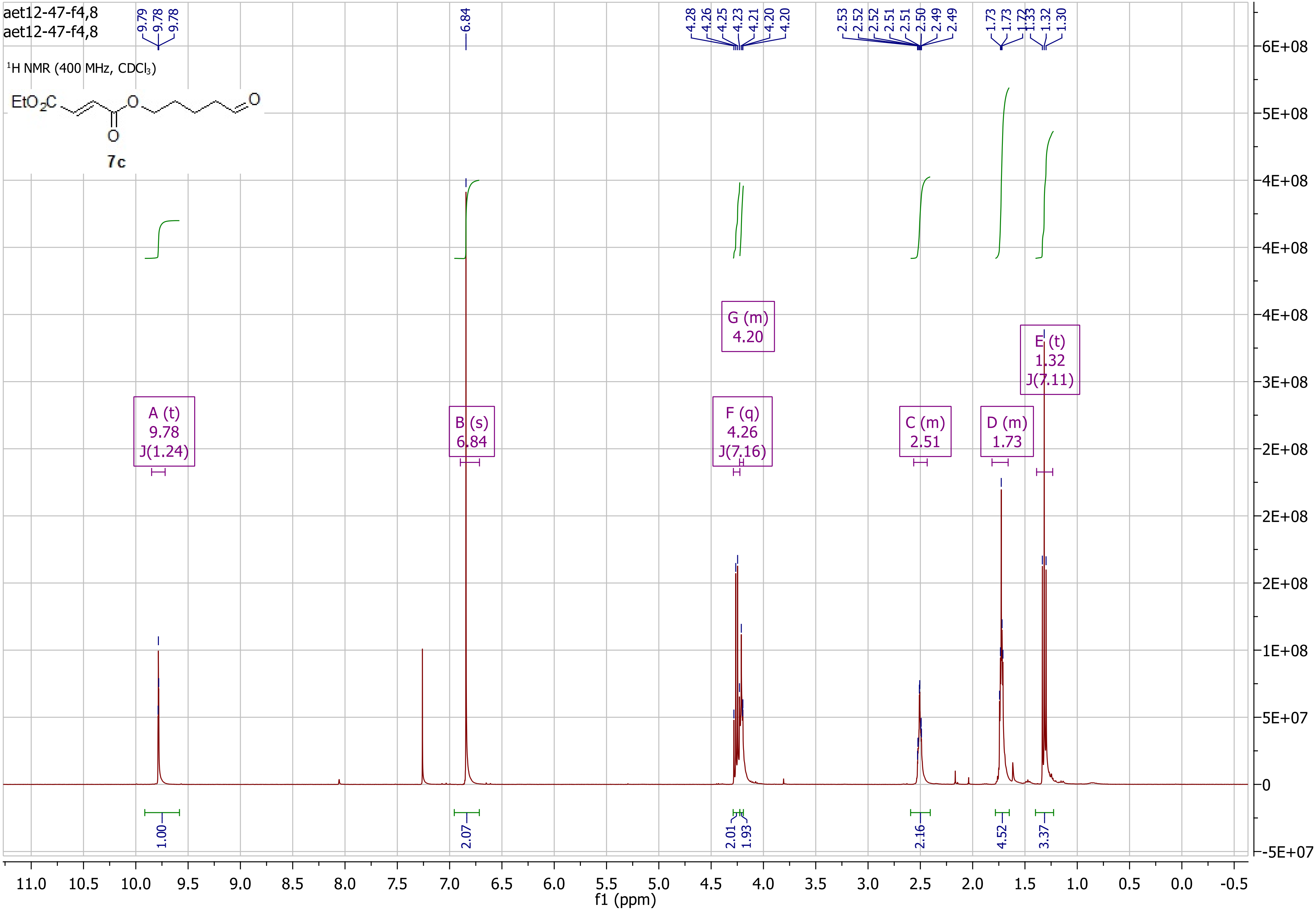

aet12-47-f4,8
aet12-47-f4,8
1H NMR (400 MHz, CDCl3)
EtO2C
O
O
O
7c
A (t) 9.78 J(1.24)
B (s) 6.84
F (q) 4.26 J(7.16)
G (m) 4.20
C (m) 2.51
D (m) 1.73
E (t) 1.32 J(7.11)
1.00
2.07
2.01
1.93
2.16
4.52
3.37
f1 (ppm)

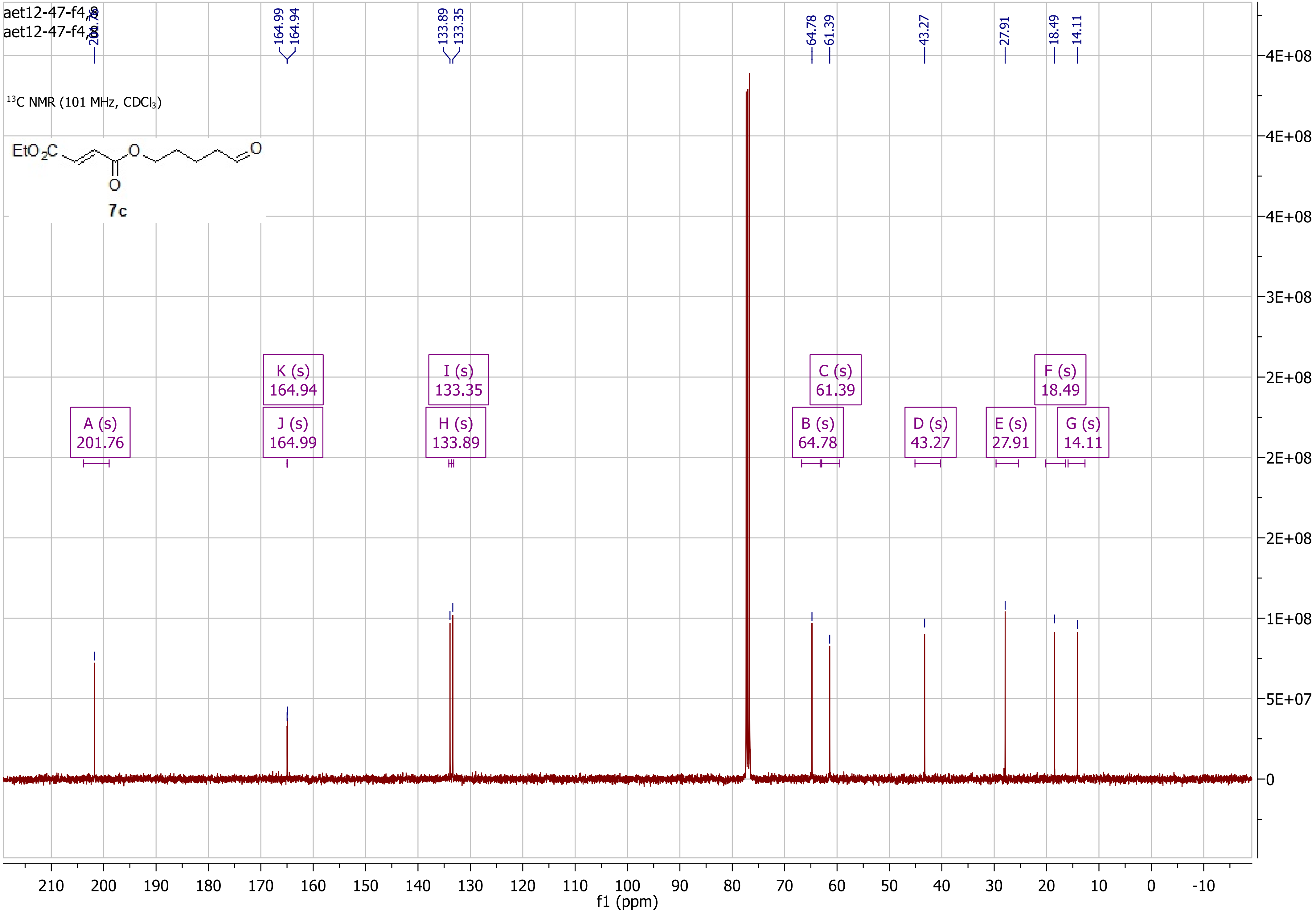

aet12-47-f4,8
aet12-47-f4,8
13C NMR (101 MHz, CDCl3)
EtO2C
O
O
O
7c
201.76
164.99
164.94
133.89
133.35
64.78
61.39
43.27
27.91
18.49
14.11
A (s) 201.76
J (s) 164.99
K (s) 164.94
H (s) 133.89
I (s) 133.35
B (s) 64.78
C (s) 61.39
D (s) 43.27
E (s) 27.91
F (s) 18.49
G (s) 14.11
f1 (ppm)

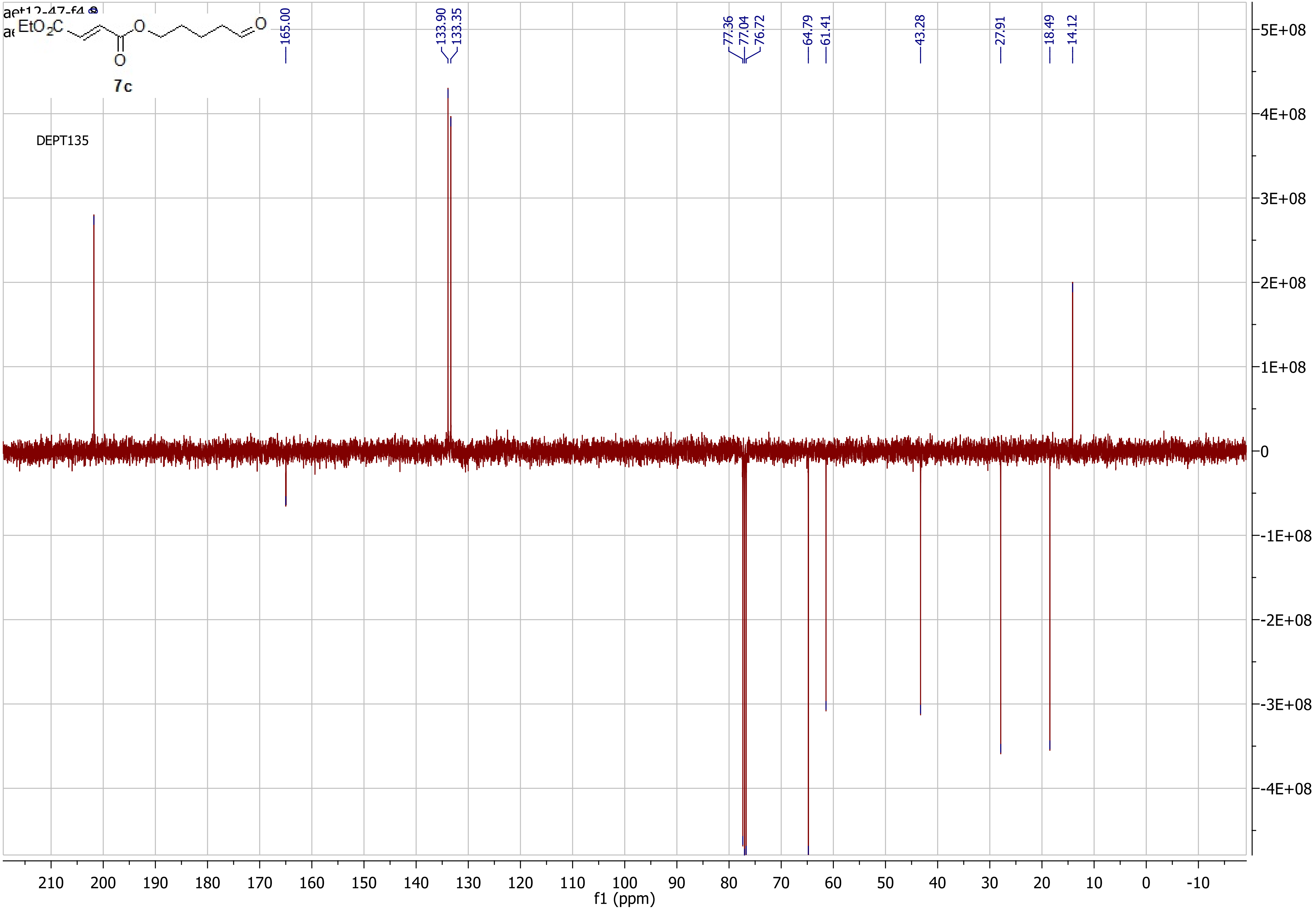

DEPT135
7c
165.00
133.90
133.35
77.36
77.04
76.72
64.79
61.41
43.28
27.91
18.49
14.12
f1 (ppm)

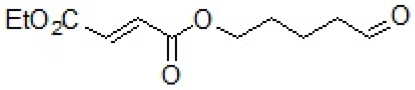

7c

COSY

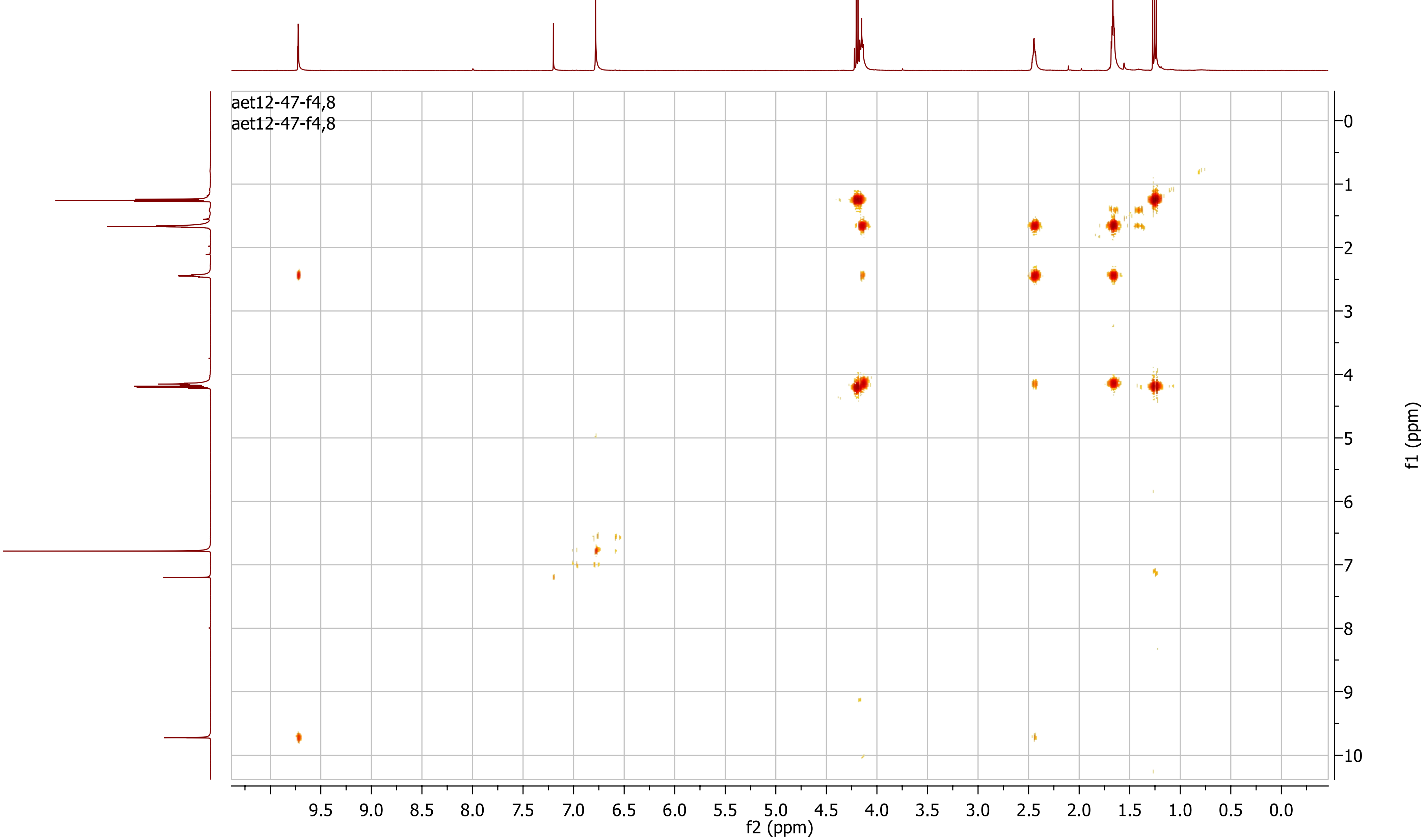

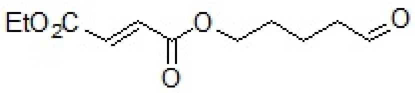
EtO2C
O
O
O

7c

HMQC

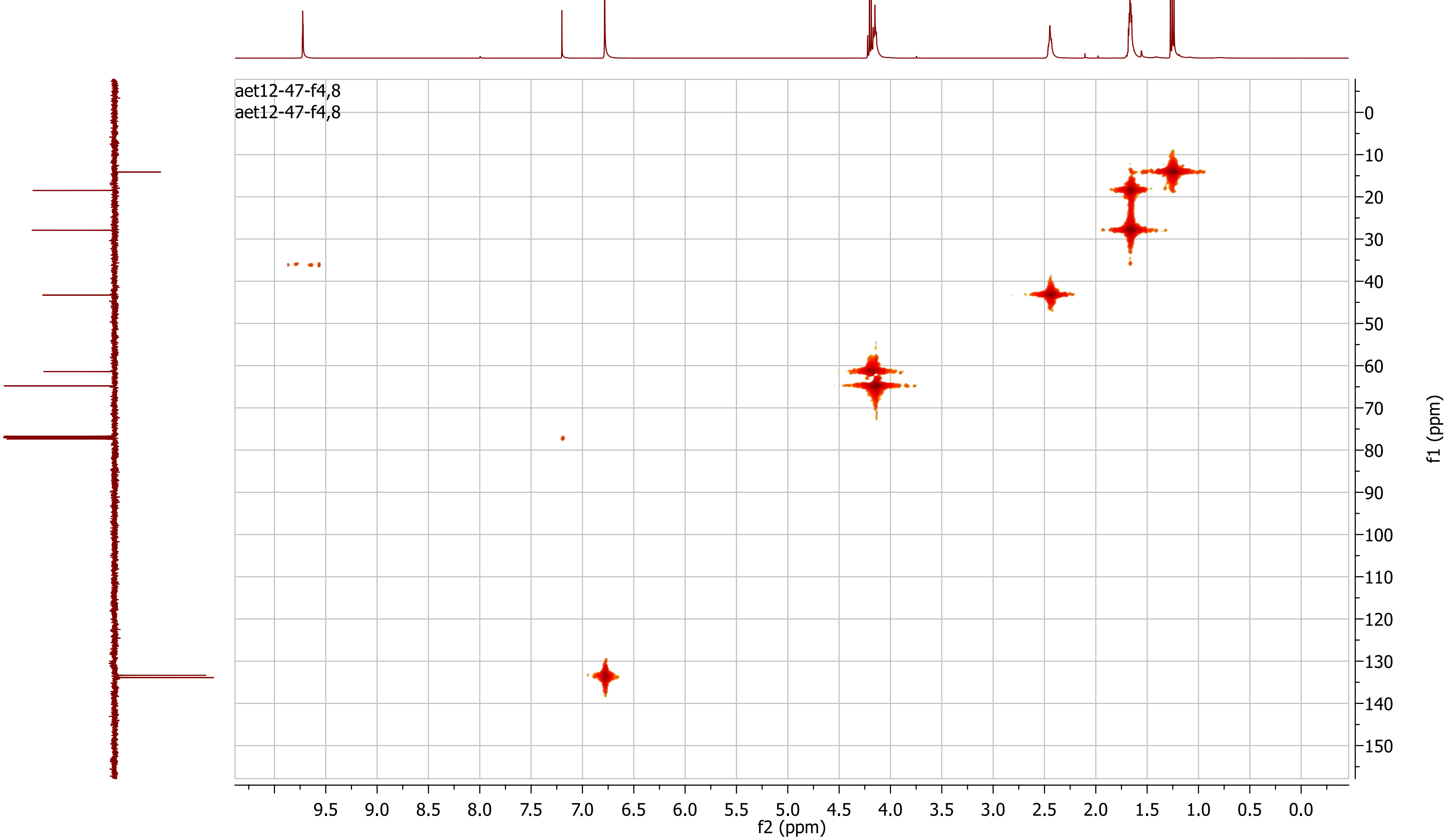
aet12-47-f4,8
aet12-47-f4,8
9.5 9.0 8.5 8.0 7.5 7.0 6.5 6.0 5.5 5.0 4.5 4.0 3.5 3.0 2.5 2.0 1.5 1.0 0.5 0.0
f2 (ppm)
0 10 20 30 40 50 60 70 80 90 100 110 120 130 140 150
f1 (ppm)

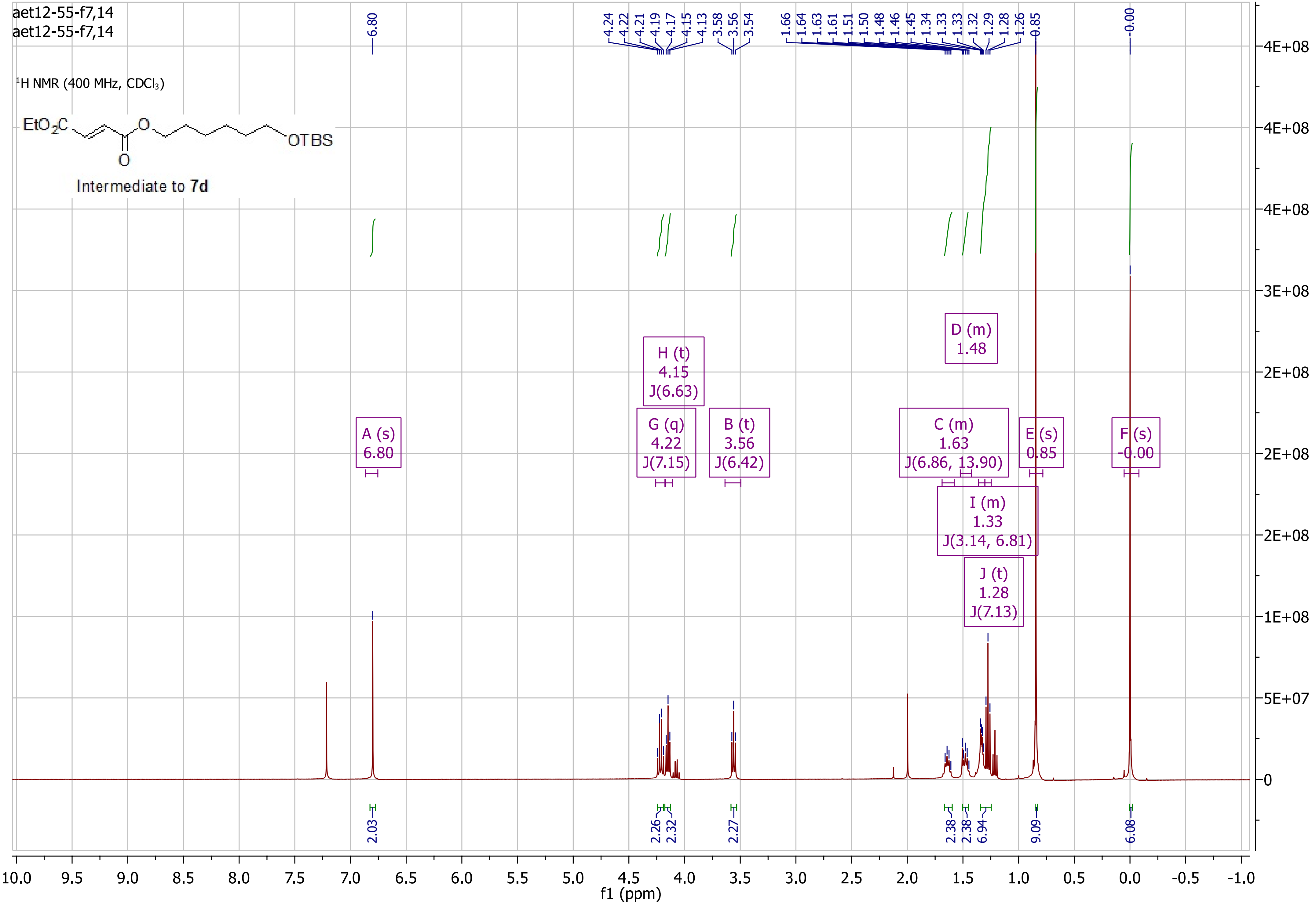

aet12-55-f7,14
aet12-55-f7,14
1H NMR (400 MHz, CDCl3)
EtO2C
O
OTBS
O
Intermediate to 7d
6.80
4.24
4.22
4.21
4.19
4.17
4.15
4.13
3.58
3.56
3.54
1.66
1.64
1.63
1.61
1.51
1.50
1.48
1.46
1.45
1.34
1.33
1.33
1.32
1.29
1.28
1.26
0.85
-0.00
A (s) 6.80
H (t) 4.15 J(6.63)
G (q) 4.22 J(7.15)
B (t) 3.56 J(6.42)
D (m) 1.48
C (m) 1.63 J(6.86, 13.90)
E (s) 0.85
F (s) -0.00
I (m) 1.33 J(3.14, 6.81)
J (t) 1.28 J(7.13)
2.03
2.26
2.32
2.27
2.38
2.38
6.94
9.09
6.08
f1 (ppm)

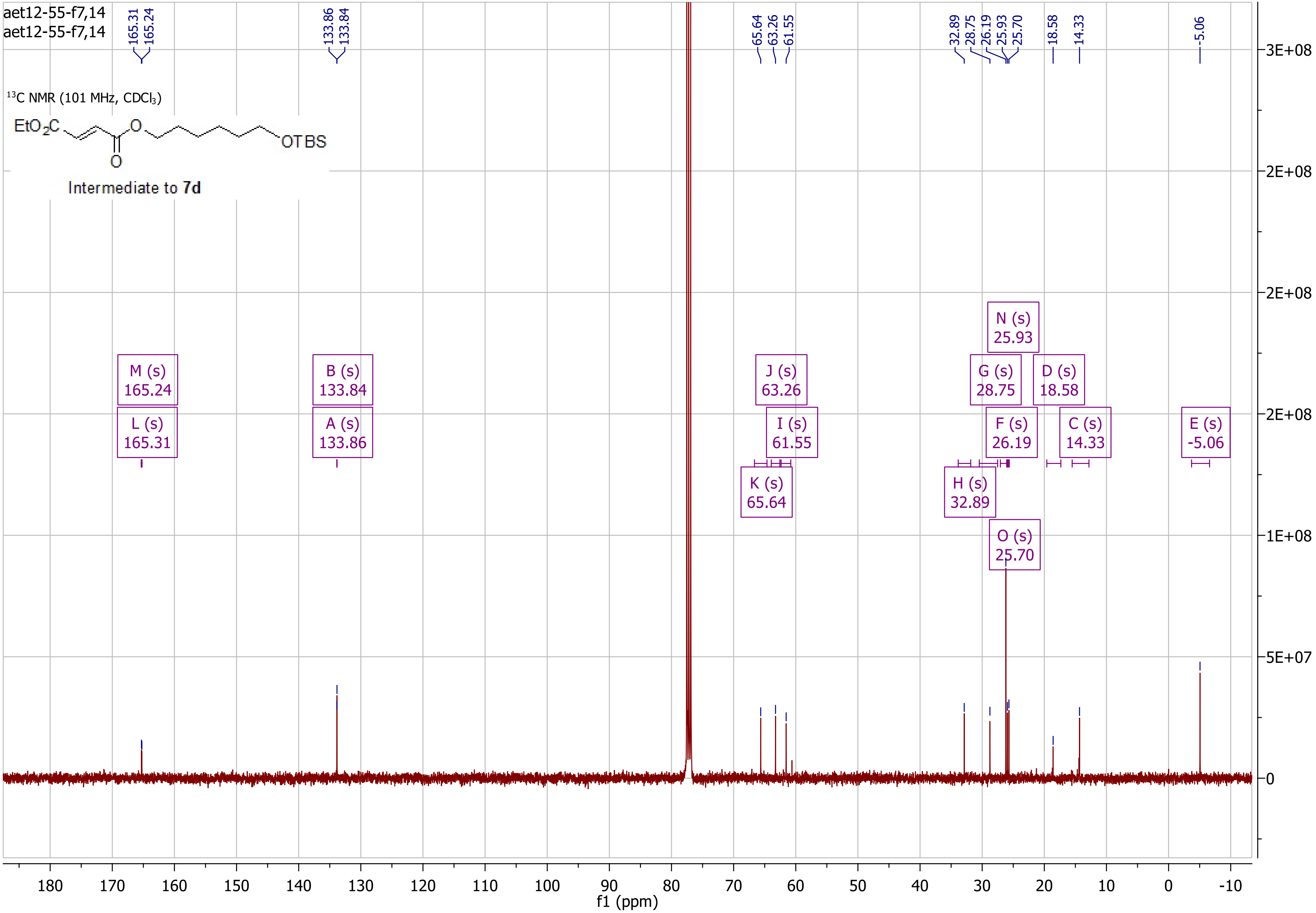
aet12-55-f7,14
aet12-55-f7,14
13C NMR (101 MHz, CDCl3)
EtO2C
O
O
OTBS
Intermediate to 7d
165.31
165.24
133.86
133.84
65.64
63.26
61.55
32.89
28.75
26.19
25.93
25.70
18.58
14.33
-5.06
M (s) 165.24
L (s) 165.31
B (s) 133.84
A (s) 133.86
J (s) 63.26
I (s) 61.55
K (s) 65.64
N (s) 25.93
G (s) 28.75
D (s) 18.58
F (s) 26.19
C (s) 14.33
H (s) 32.89
O (s) 25.70
E (s) -5.06
3E+08
2E+08
2E+08
2E+08
1E+08
5E+07
0
180
170
160
150
140
130
120
110
100
90
80
70
60
50
40
30
20
10
0
-10
f1 (ppm)

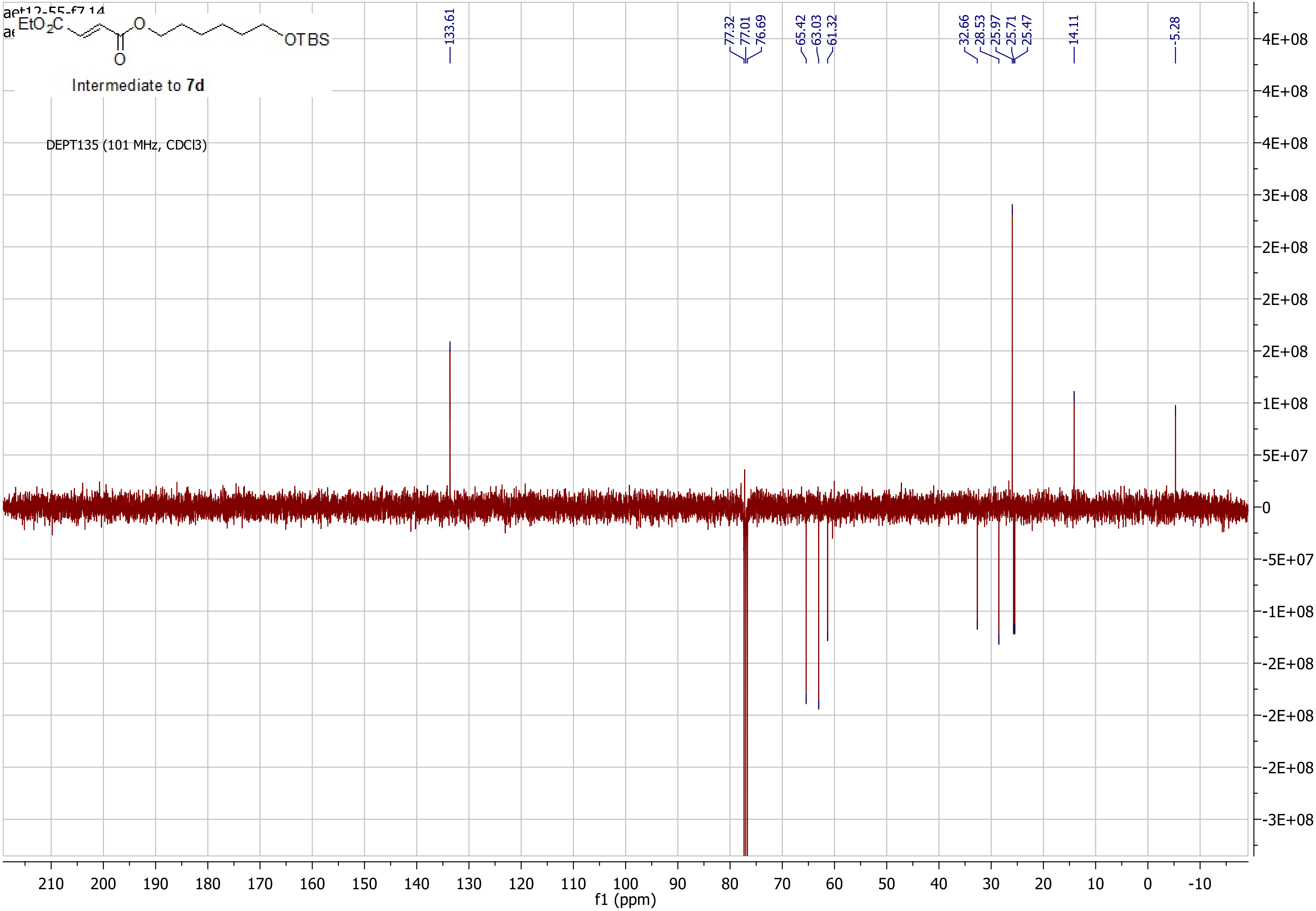

aet12-55-f7.14
EtO2C
O
OTBS
O
Intermediate to 7d
DEPT135 (101 MHz, CDCl3)
133.61
77.32
77.01
76.69
65.42
63.03
61.32
32.66
28.53
25.97
25.71
25.47
14.11
-5.28
f1 (ppm)

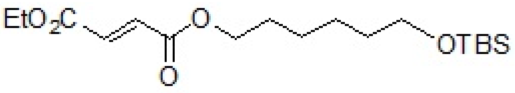

Intermediate to **7d**

COSY (CDCl3)

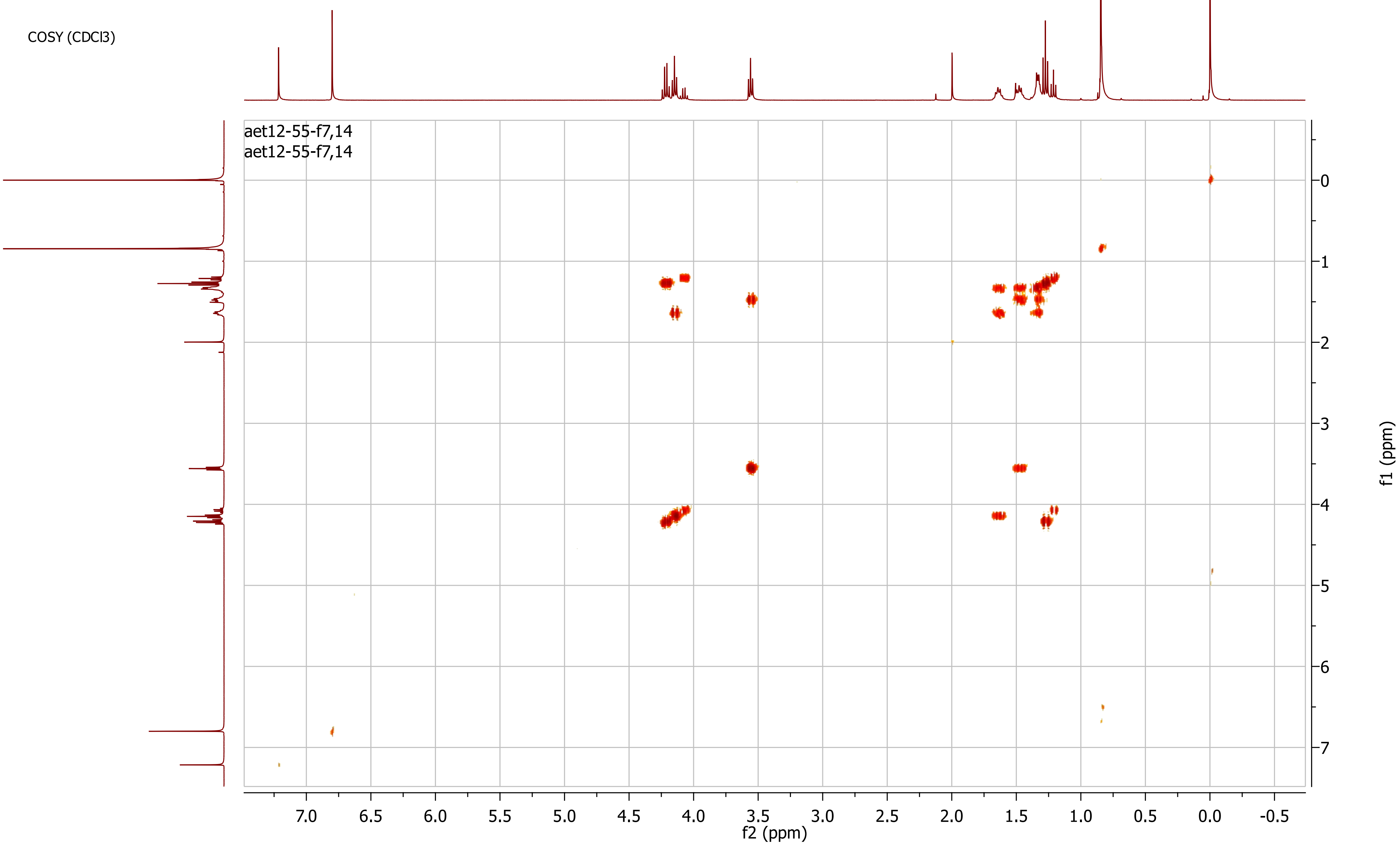

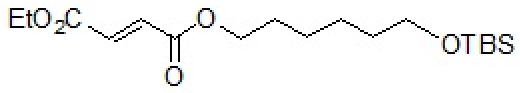

Intermediate to **7d**

HMQC (CDCl3)

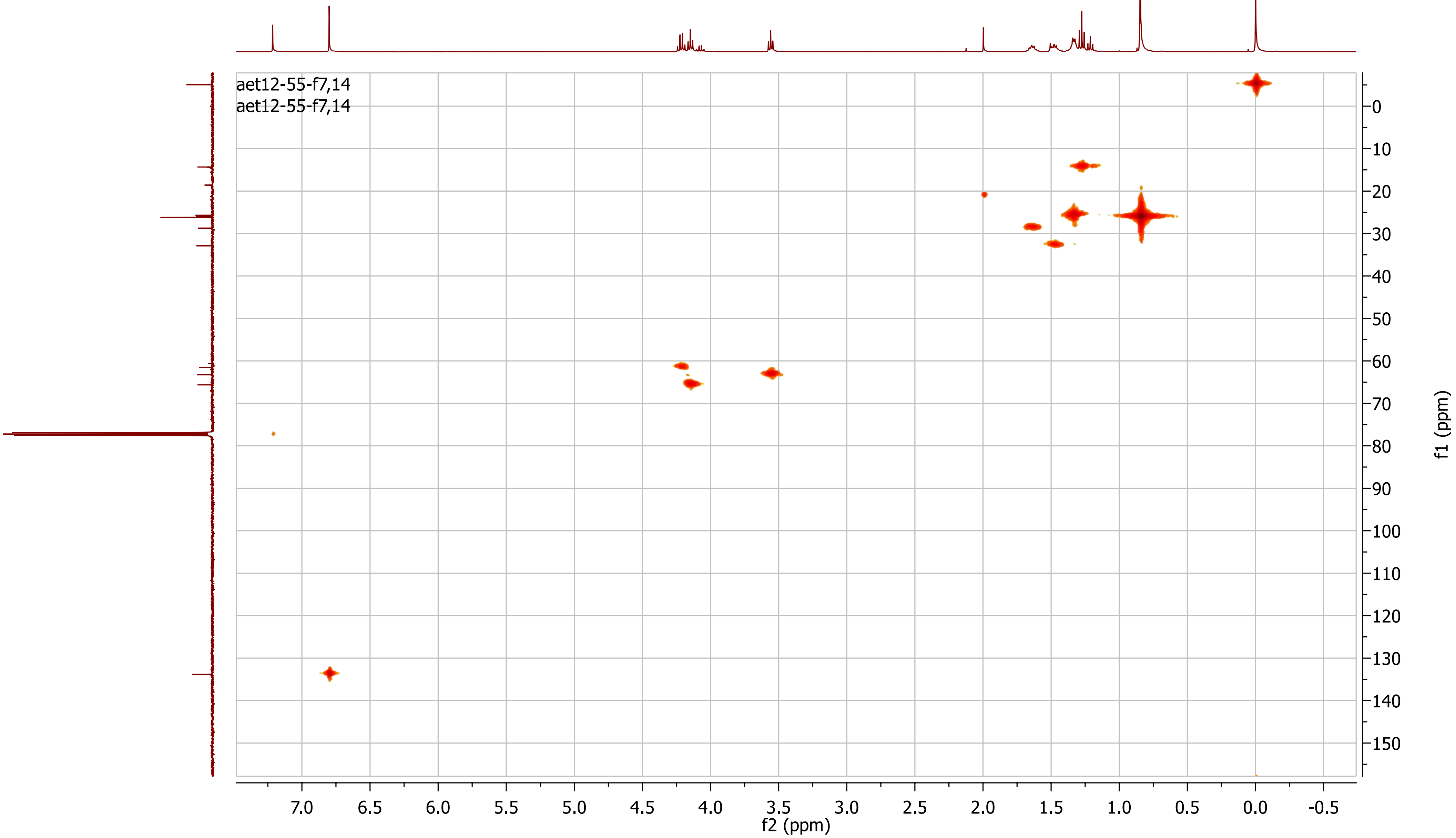

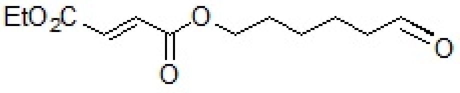
EtO2C
O
O
O

7d

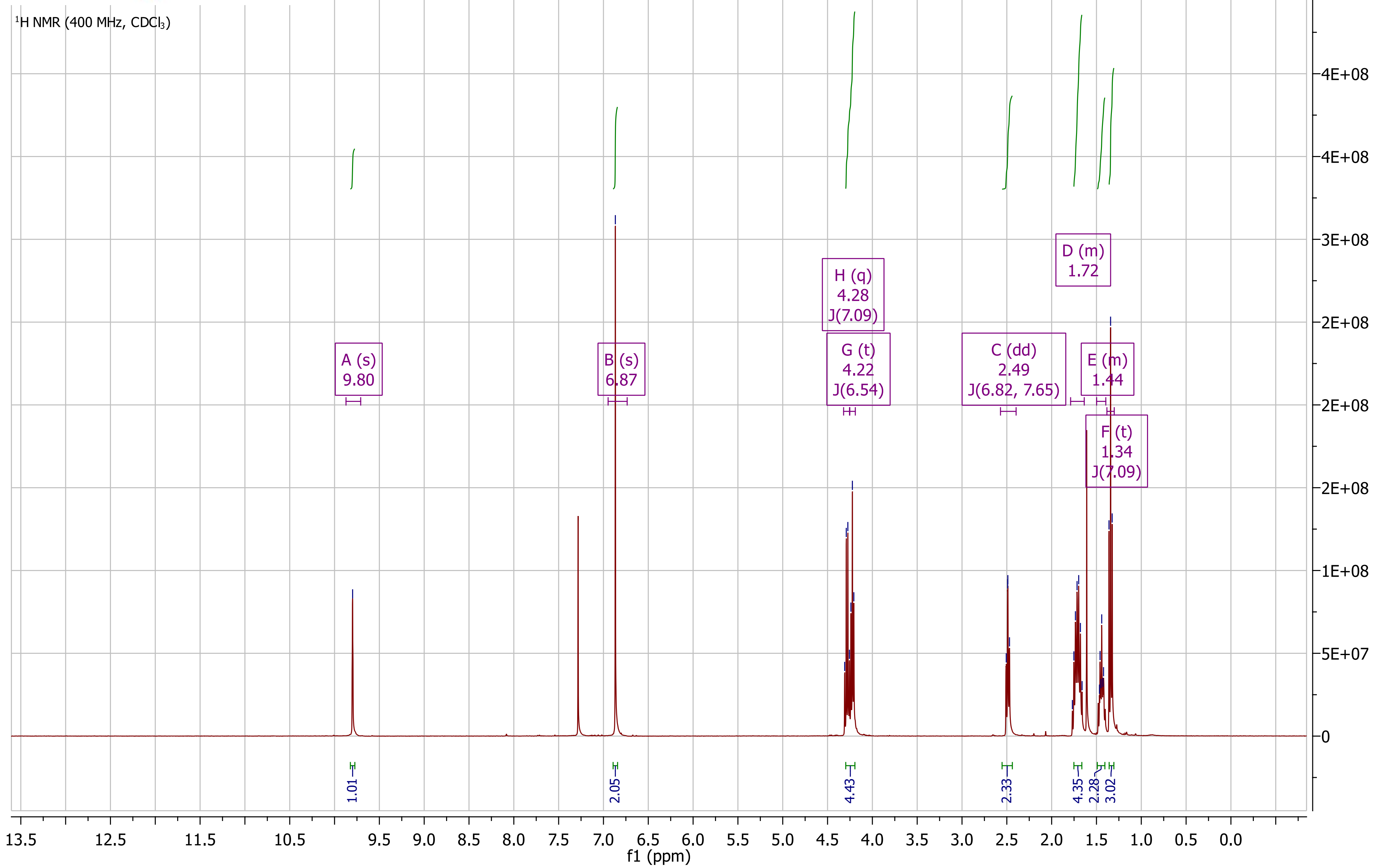
1H NMR (400 MHz, CDCl3)
9.80
6.87
4.31 4.29 4.27 4.26 4.24 4.22 4.21
2.51 2.49 2.49 2.47 1.77 1.75 1.73 1.72 1.70 1.68 1.66 1.47 1.46 1.45 1.44 1.43 1.42 1.36 1.34 1.32
A (s) 9.80
B (s) 6.87
H (q) 4.28 J(7.09)
G (t) 4.22 J(6.54)
C (dd) 2.49 J(6.82, 7.65)
D (m) 1.72
E (m) 1.44
F (t) 1.34 J(7.09)
1.01
2.05
4.43
2.33
4.35
2.28
3.02
4E+08
4E+08
4E+08
3E+08
2E+08
2E+08
2E+08
1E+08
5E+07
0
13.5 12.5 11.5 10.5 9.5 9.0 8.5 8.0 7.5 7.0 6.5 6.0 5.5 5.0 4.5 4.0 3.5 3.0 2.5 2.0 1.5 1.0 0.5 0.0
f1 (ppm)

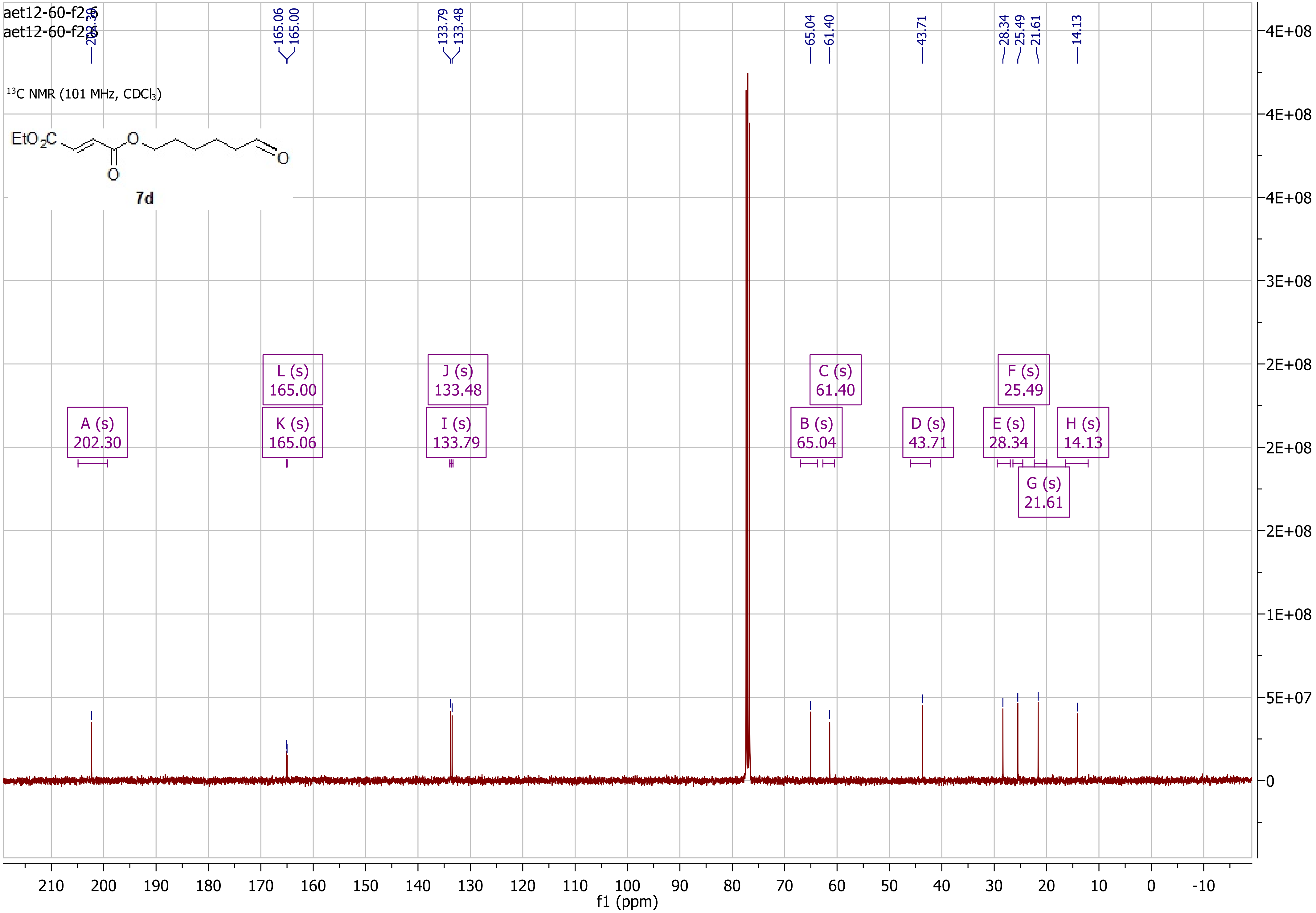

aet12-60-f2.6
aet12-60-f2.6
202.30
165.06
165.00
133.79
133.48
65.04
61.40
43.71
28.34
25.49
21.61
14.13
13C NMR (101 MHz, CDCl3)
EtO2C
O
O
O
7d
A (s) 202.30
L (s) 165.00
K (s) 165.06
J (s) 133.48
I (s) 133.79
C (s) 61.40
B (s) 65.04
D (s) 43.71
F (s) 25.49
E (s) 28.34
H (s) 14.13
G (s) 21.61
f1 (ppm)

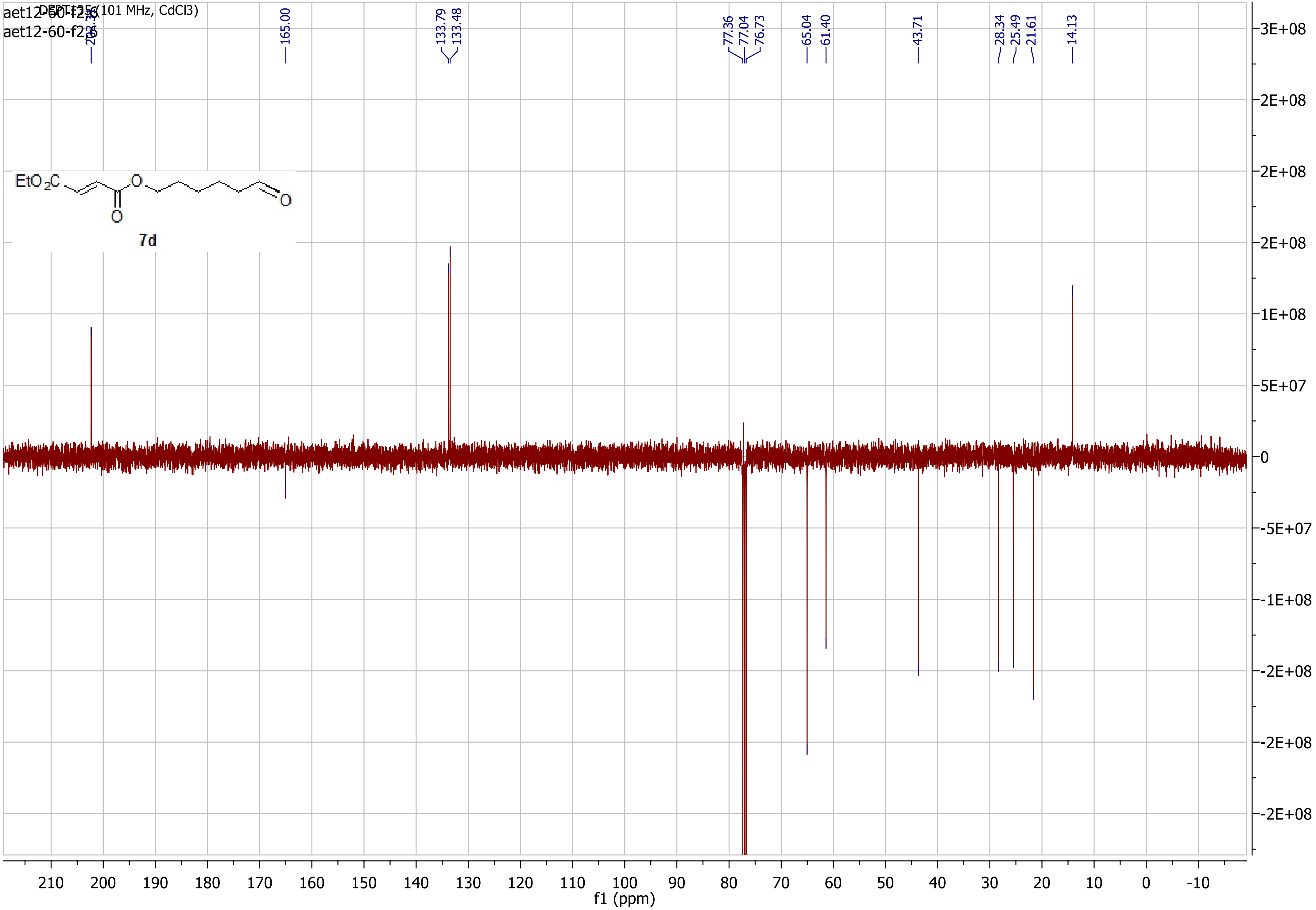
aet12-60-f25 DEPT-135 (101 MHz, CdCl3)
aet12-60-f25
165.00
133.79
133.48
77.36
77.04
76.73
65.04
61.40
43.71
28.34
25.49
21.61
14.13
EtO2C
O
O
O
7d
f1 (ppm)

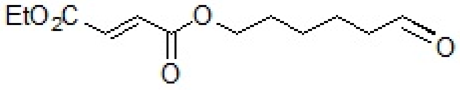

7d

COSY (CDCl3)

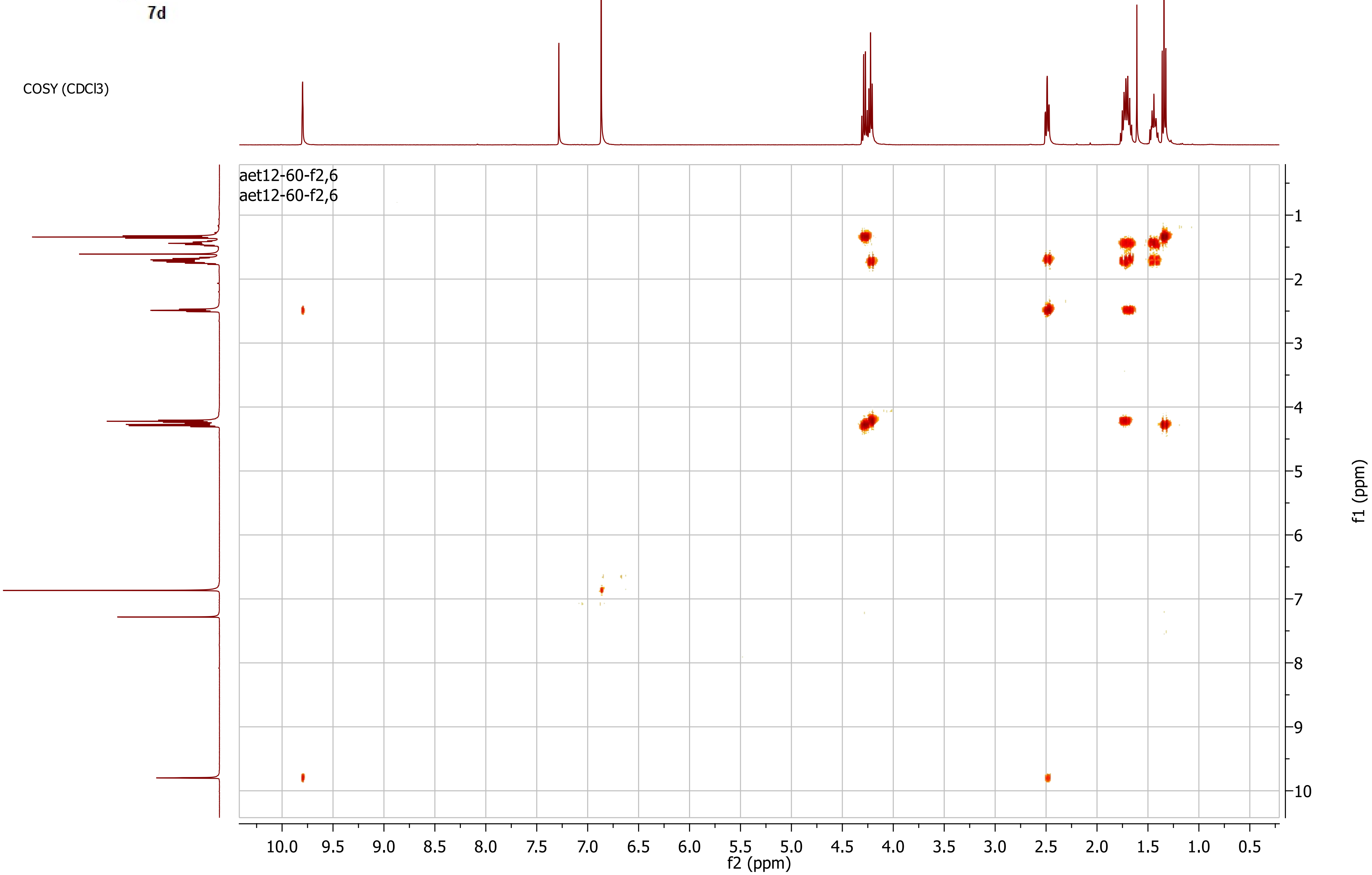

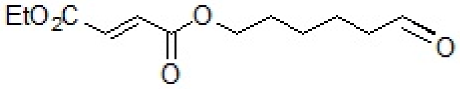

7d

HMQC (CDCl3)

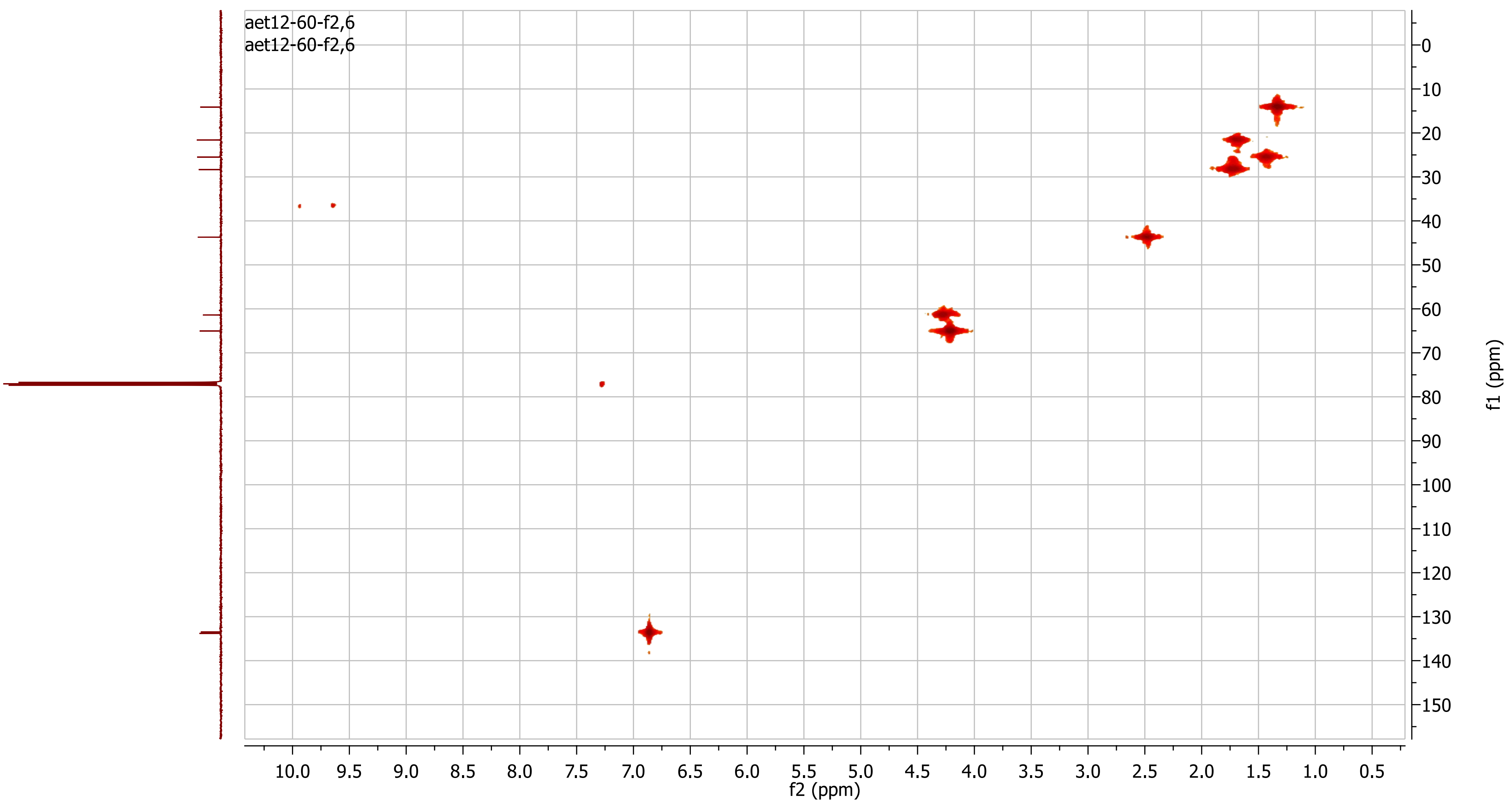

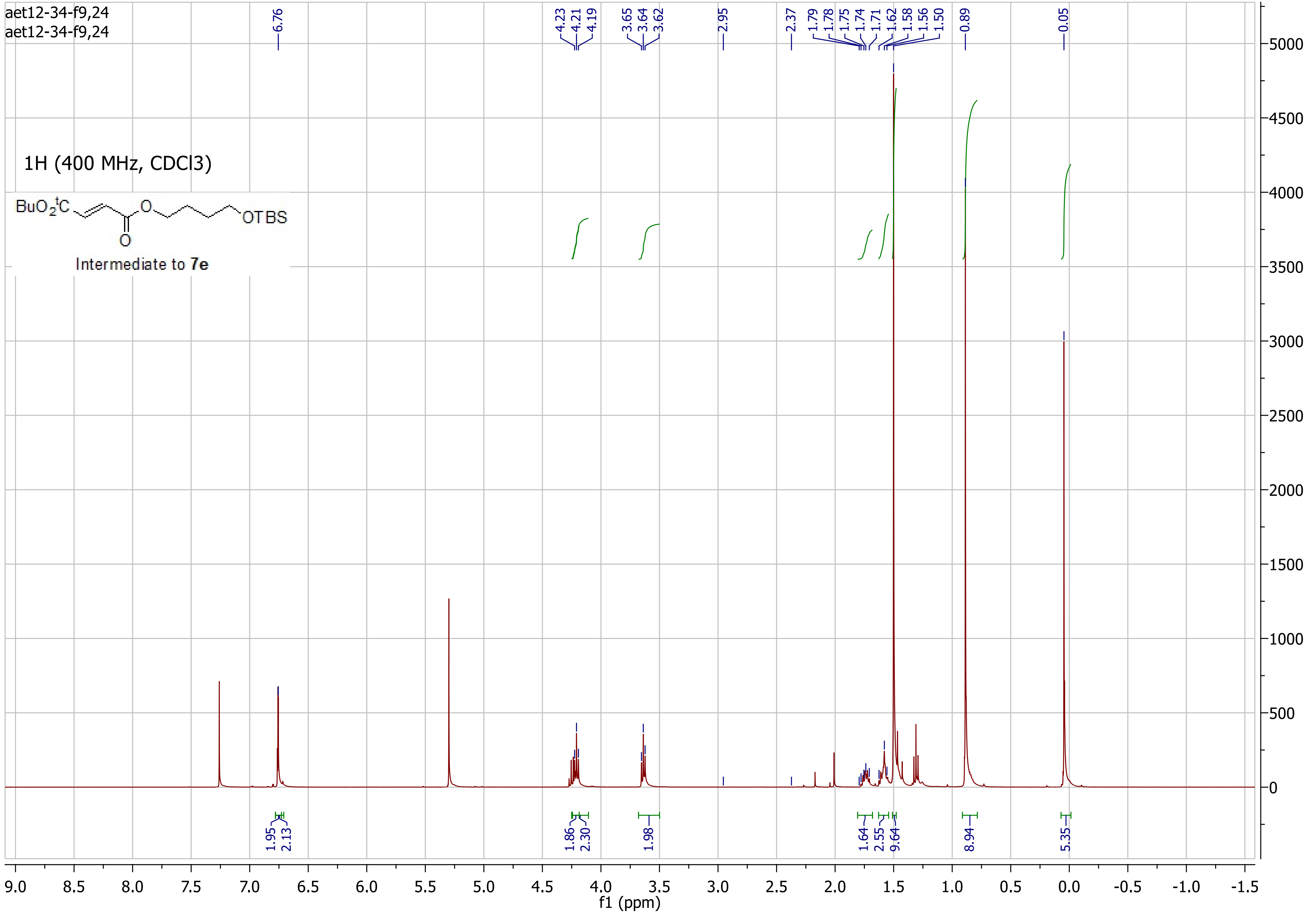
aet12-34-f9,24
aet12-34-f9,24
1H (400 MHz, CDCl3)
BuO2tC
O
O
OTBS
Intermediate to 7e
6.76
4.23
4.21
4.19
3.65
3.64
3.62
2.95
2.37
1.79
1.78
1.75
1.74
1.71
1.62
1.58
1.56
1.50
0.89
0.05
1.95
2.13
1.86
2.30
1.98
1.64
2.55
9.64
8.94
5.35
f1 (ppm)

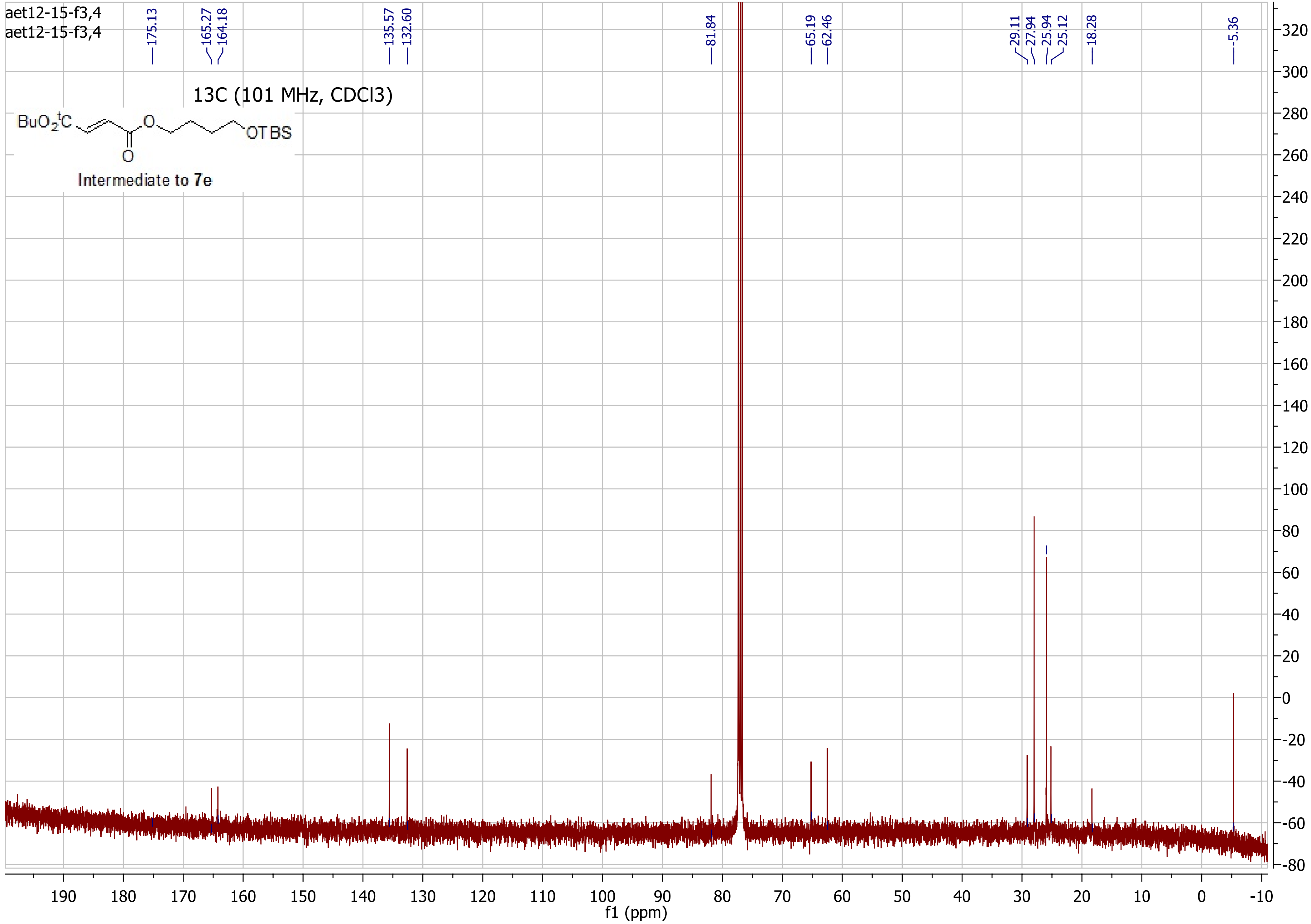
aet12-15-f3,4
aet12-15-f3,4
175.13
165.27
164.18
135.57
132.60
81.84
65.19
62.46
29.11
27.94
25.94
25.12
18.28
-5.36
13C (101 MHz, CDCl3)
BuO2tC
O
OTBS
O
Intermediate to 7e
f1 (ppm)

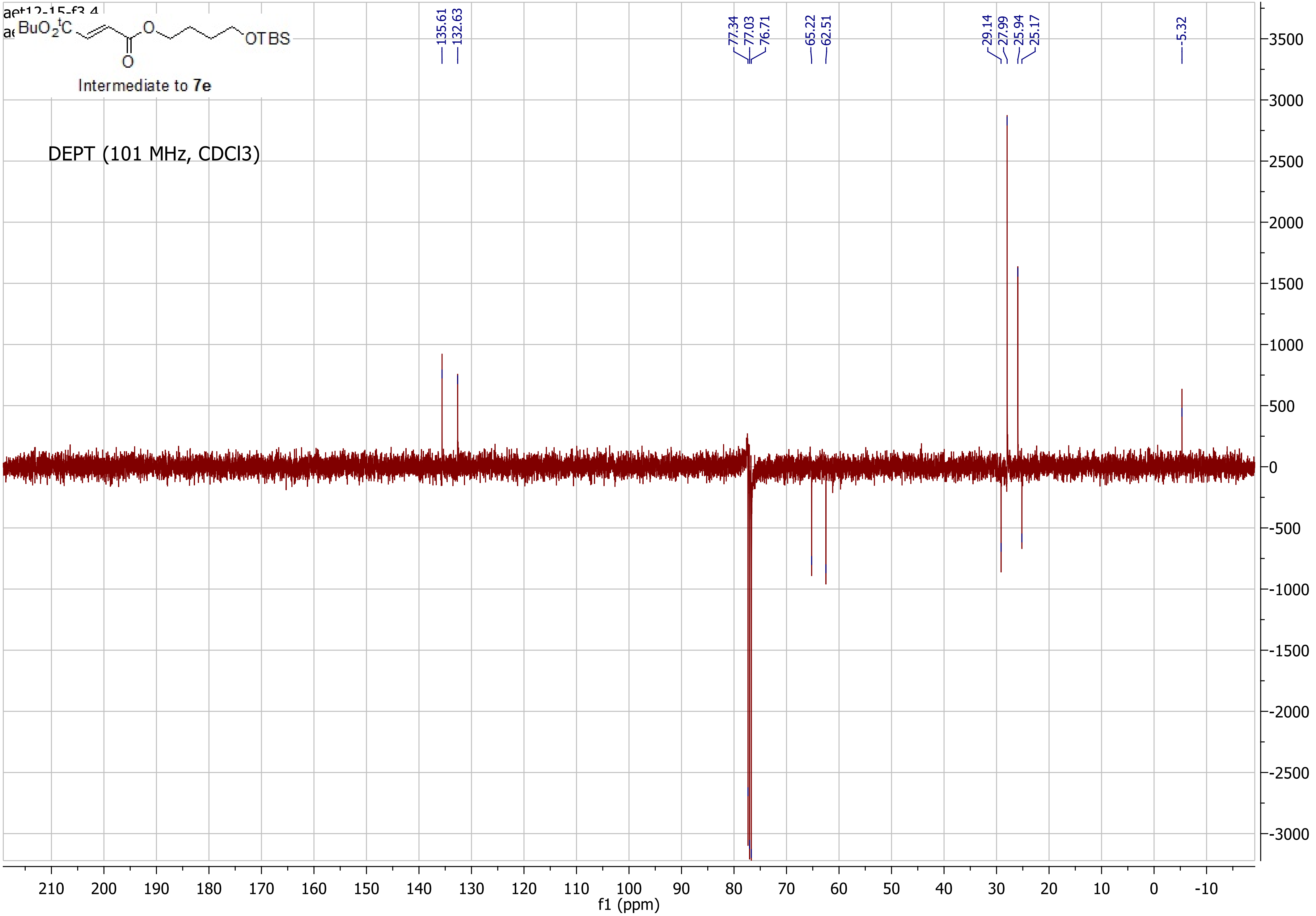
aet12-15-f3.4
^{t}BuO$_2$C
O
O
OTBS
Intermediate to 7e
DEPT (101 MHz, CDCl3)
135.61
132.63
77.34
77.03
76.71
65.22
62.51
29.14
27.99
25.94
25.17
-5.32
f1 (ppm)

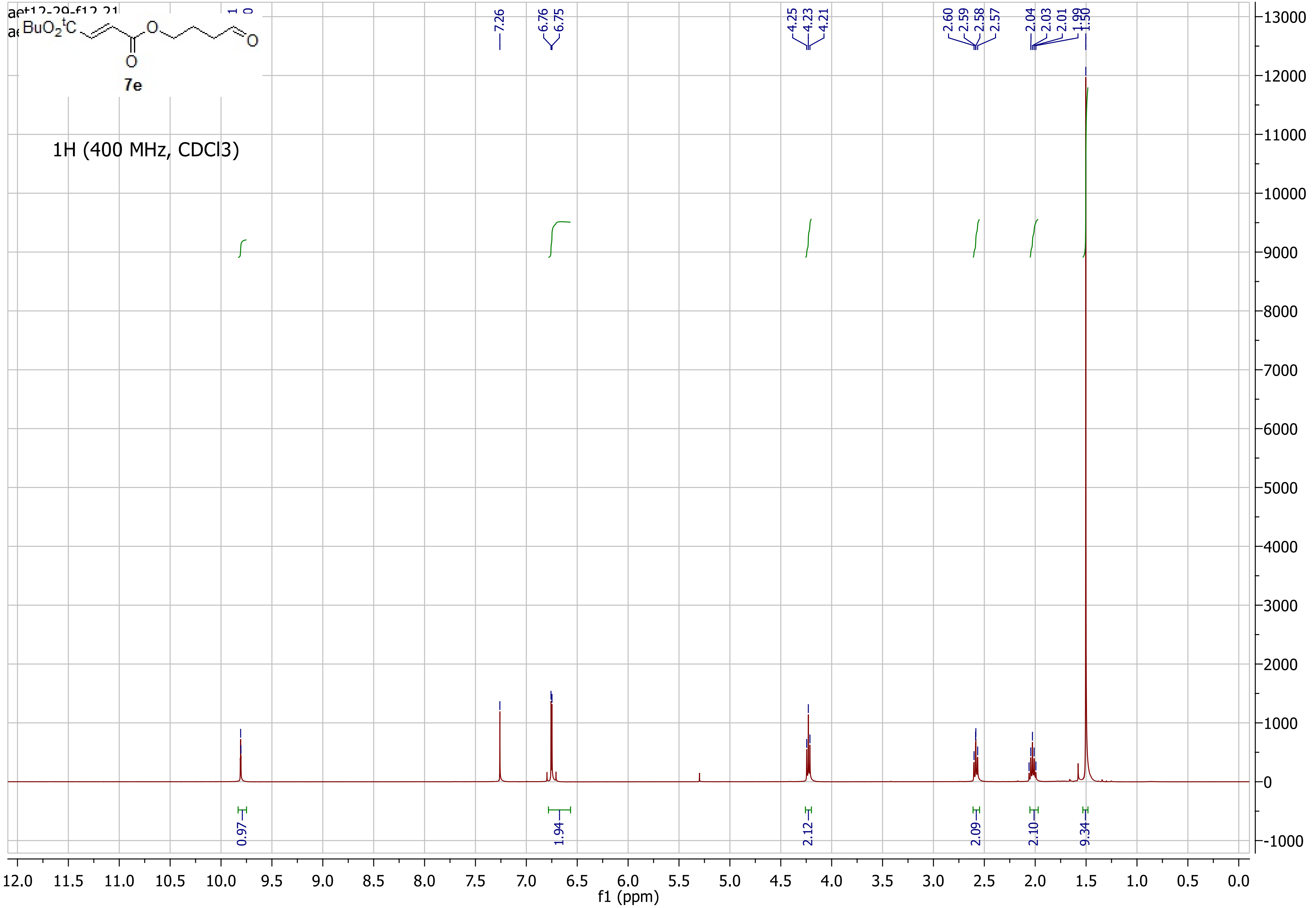

$^tBuO_2C$
7e
1H (400 MHz, CDCl3)
7.26
6.76
6.75
4.25
4.23
4.21
2.60
2.59
2.58
2.57
2.04
2.03
2.01
1.99
1.50
0.97
1.94
2.12
2.09
2.10
9.34
f1 (ppm)

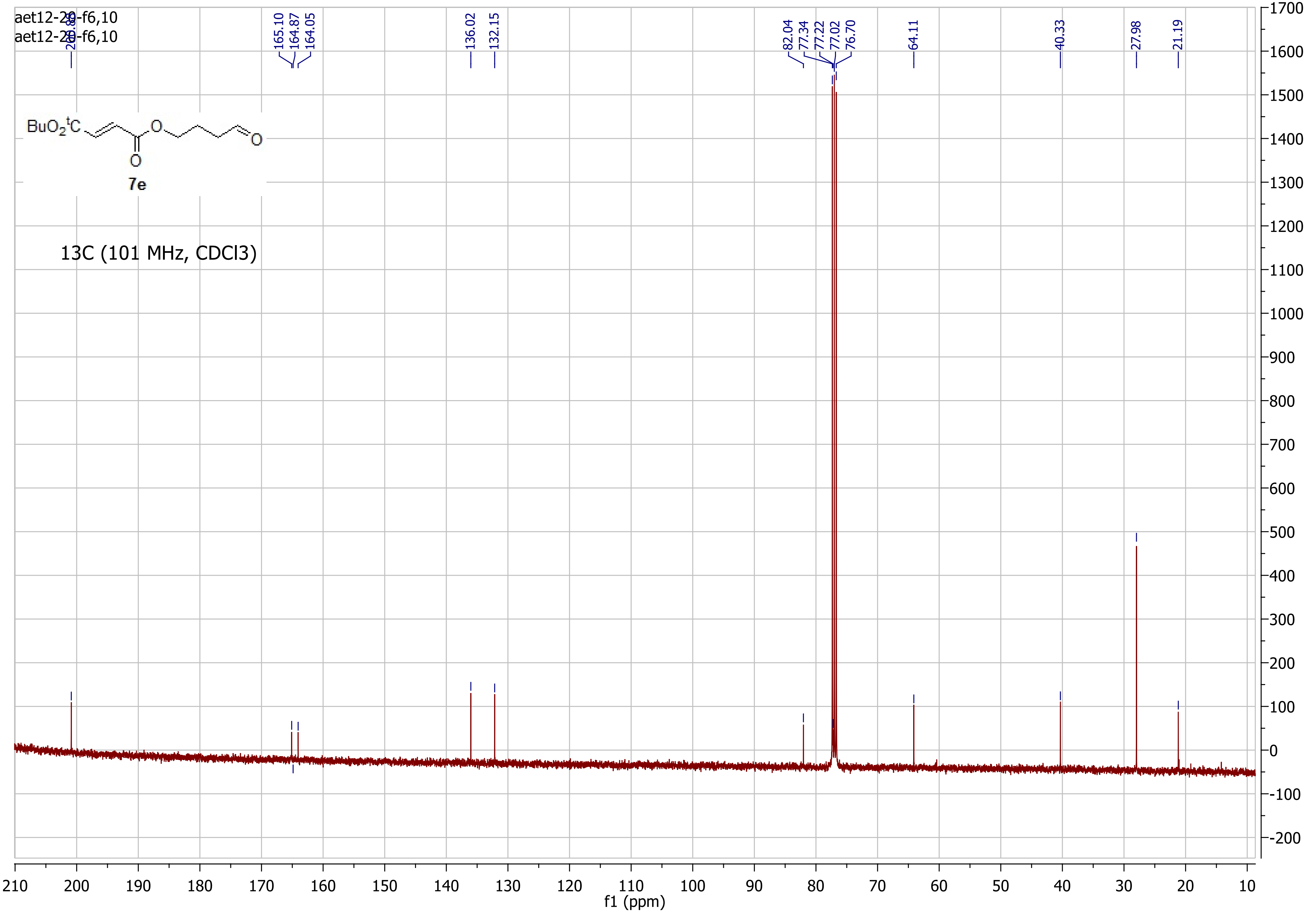
aet12-29-f6,10
aet12-29-f6,10
165.10
164.87
164.05
136.02
132.15
82.04
77.34
77.22
77.02
76.70
64.11
40.33
27.98
21.19
BuO2tC
O
O
O
7e
13C (101 MHz, CDCl3)
f1 (ppm)

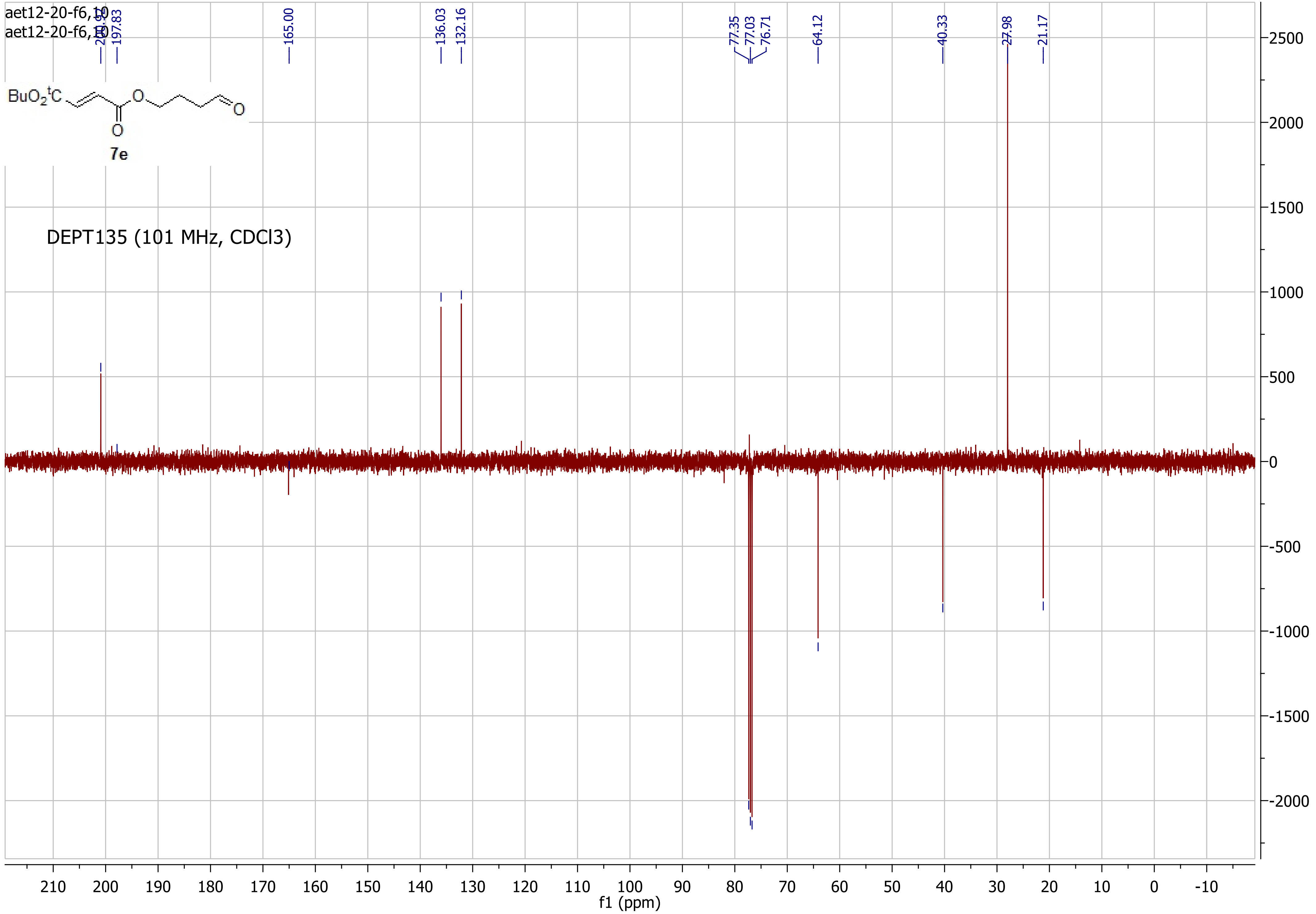
aet12-20-f6,10
aet12-20-f6,10
197.83
165.00
136.03
132.16
77.35
77.03
76.71
64.12
40.33
27.98
21.17
BuO2tC
O
O
O
O
7e
DEPT135 (101 MHz, CDCl3)
2500
2000
1500
1000
500
0
-500
-1000
-1500
-2000
210
200
190
180
170
160
150
140
130
120
110
100
90
80
70
60
50
40
30
20
10
0
-10
f1 (ppm)

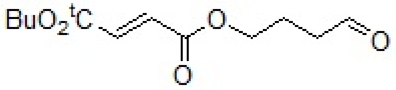

7e

COSY (400 MHz, CDCl3)

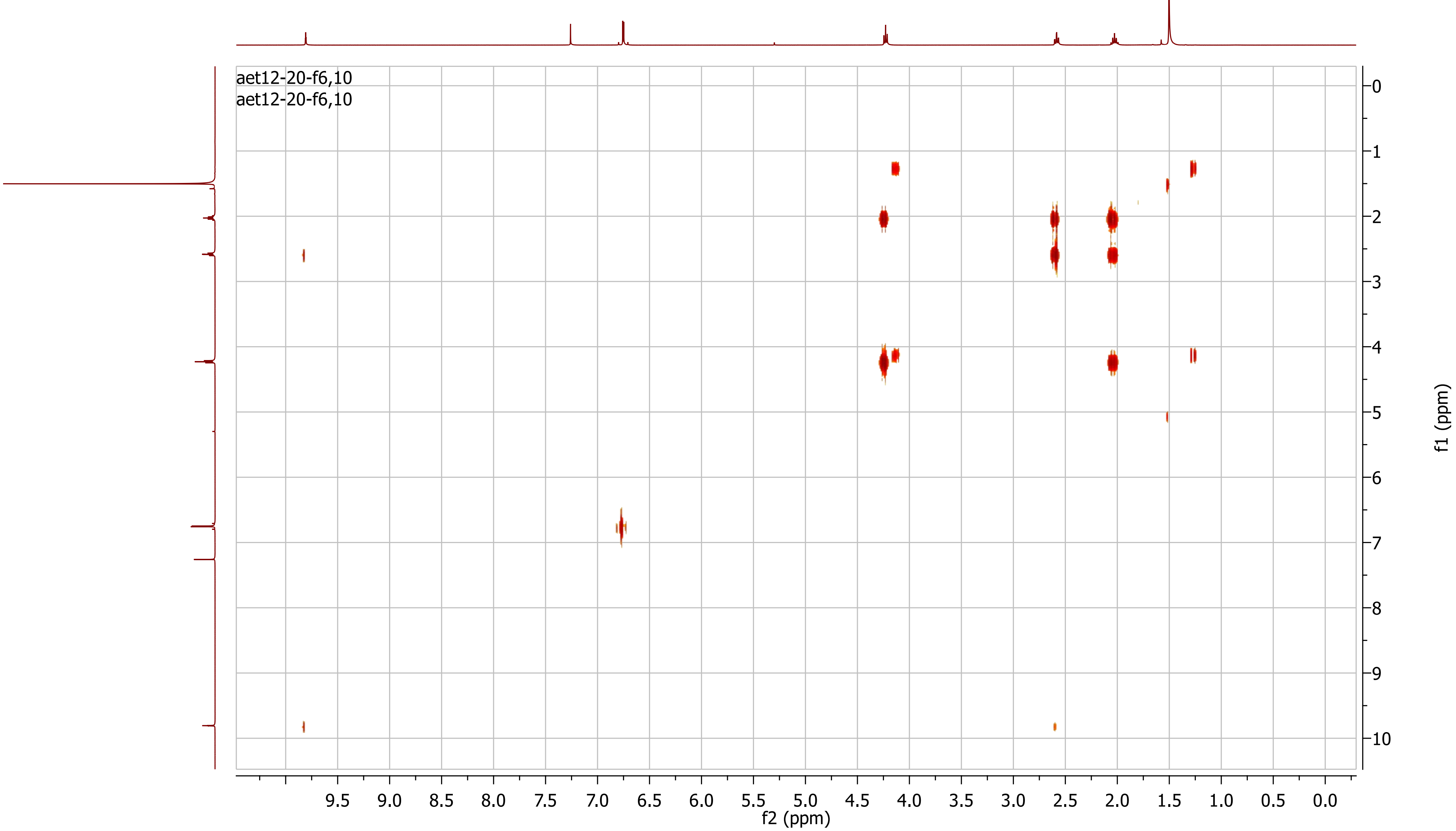

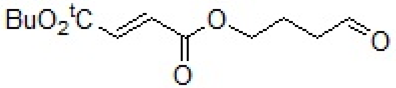

7e

HMQC (CDCl3)

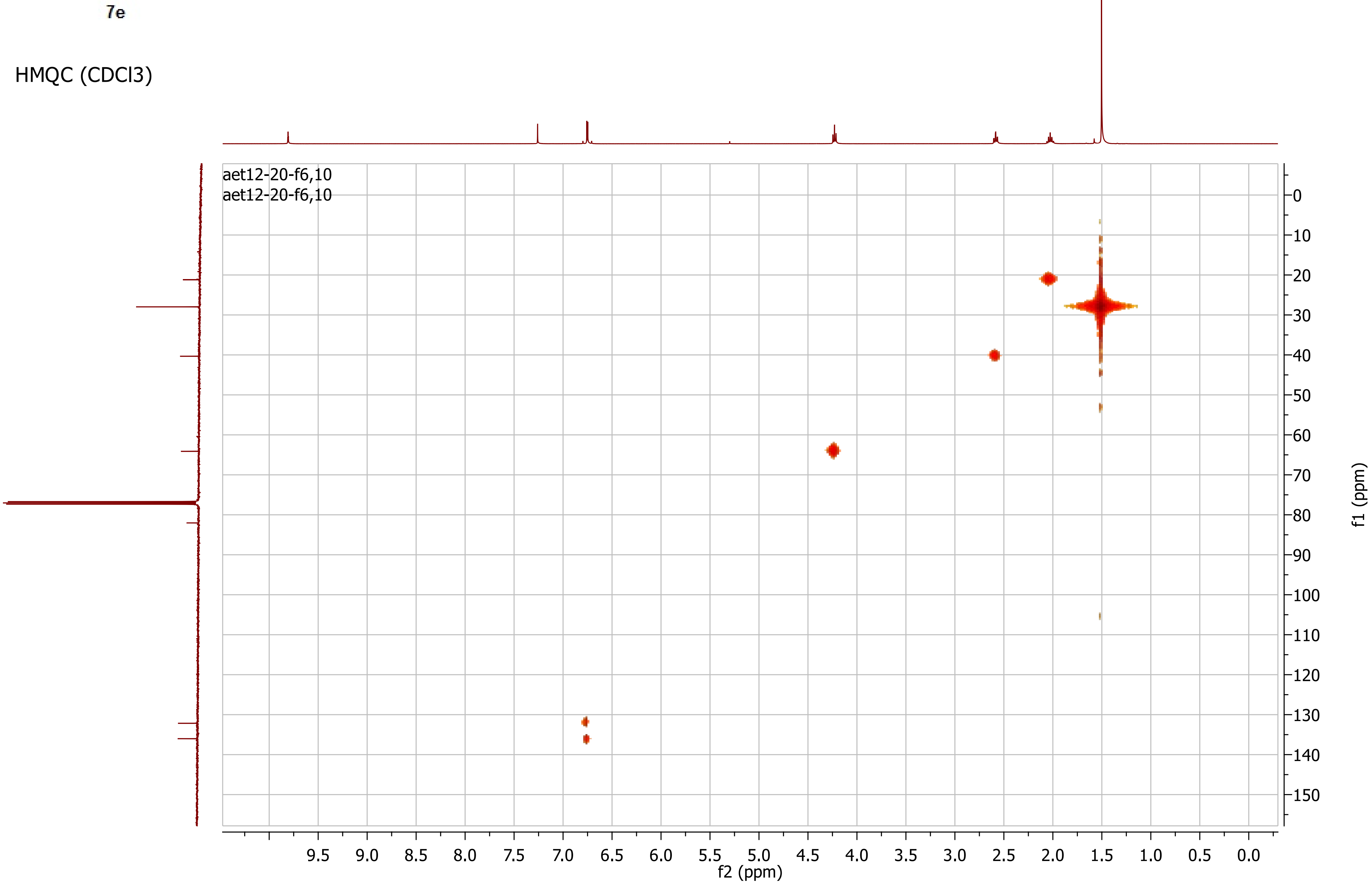

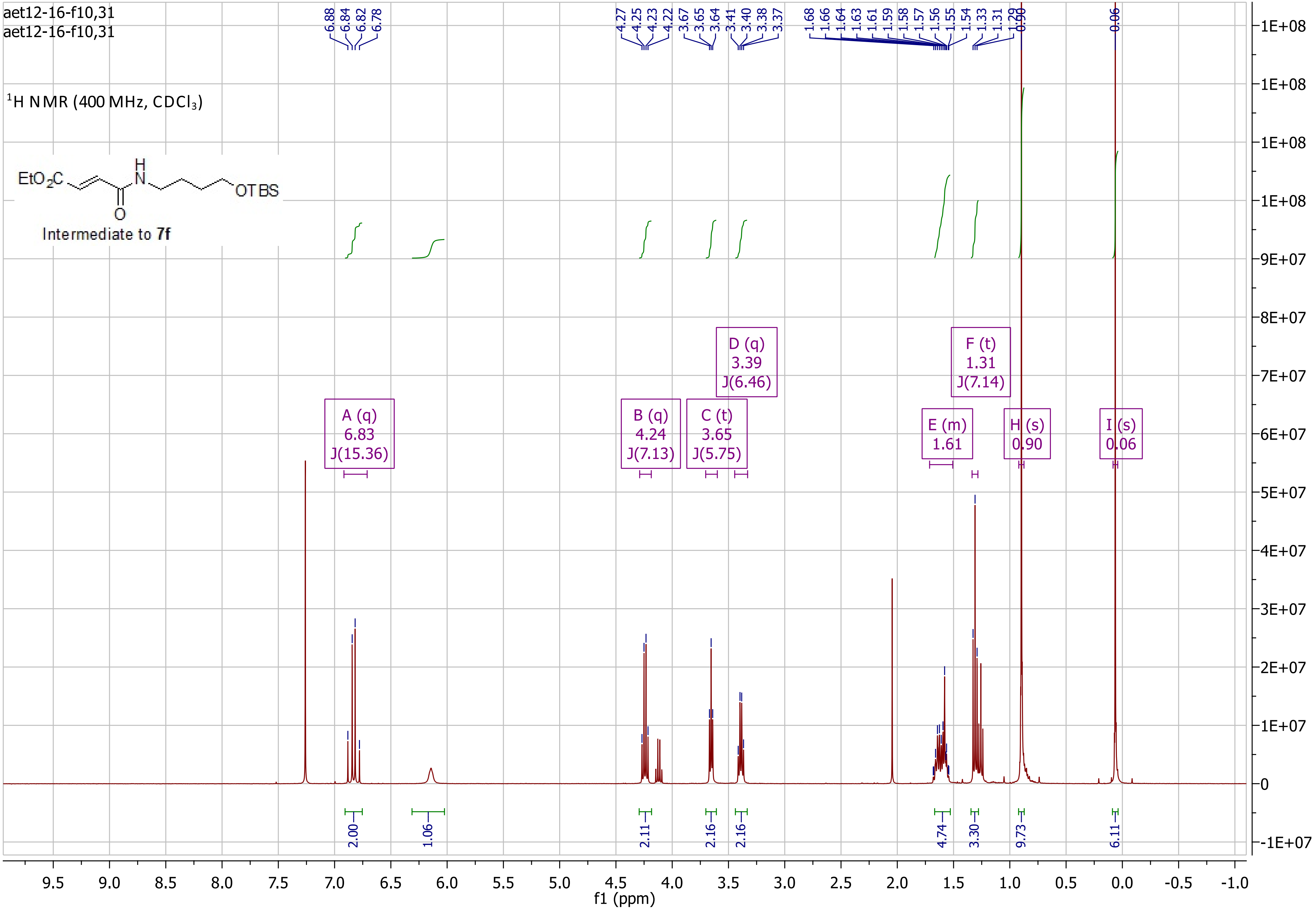

aet12-16-f10,31
aet12-16-f10,31
¹H NMR (400 MHz, CDCl₃)
EtO₂C
H
N
OTBS
O
Intermediate to **7f**
6.88
6.84
6.82
6.78
4.27
4.25
4.23
4.22
3.67
3.65
3.64
3.41
3.40
3.38
3.37
1.68
1.66
1.64
1.63
1.61
1.59
1.58
1.57
1.56
1.55
1.54
1.33
1.31
1.29
0.90
0.06
A (q) 6.83 J(15.36)
B (q) 4.24 J(7.13)
C (t) 3.65 J(5.75)
D (q) 3.39 J(6.46)
E (m) 1.61
F (t) 1.31 J(7.14)
H (s) 0.90
I (s) 0.06
2.00
1.06
2.11
2.16
2.16
4.74
3.30
9.73
6.11
f1 (ppm)

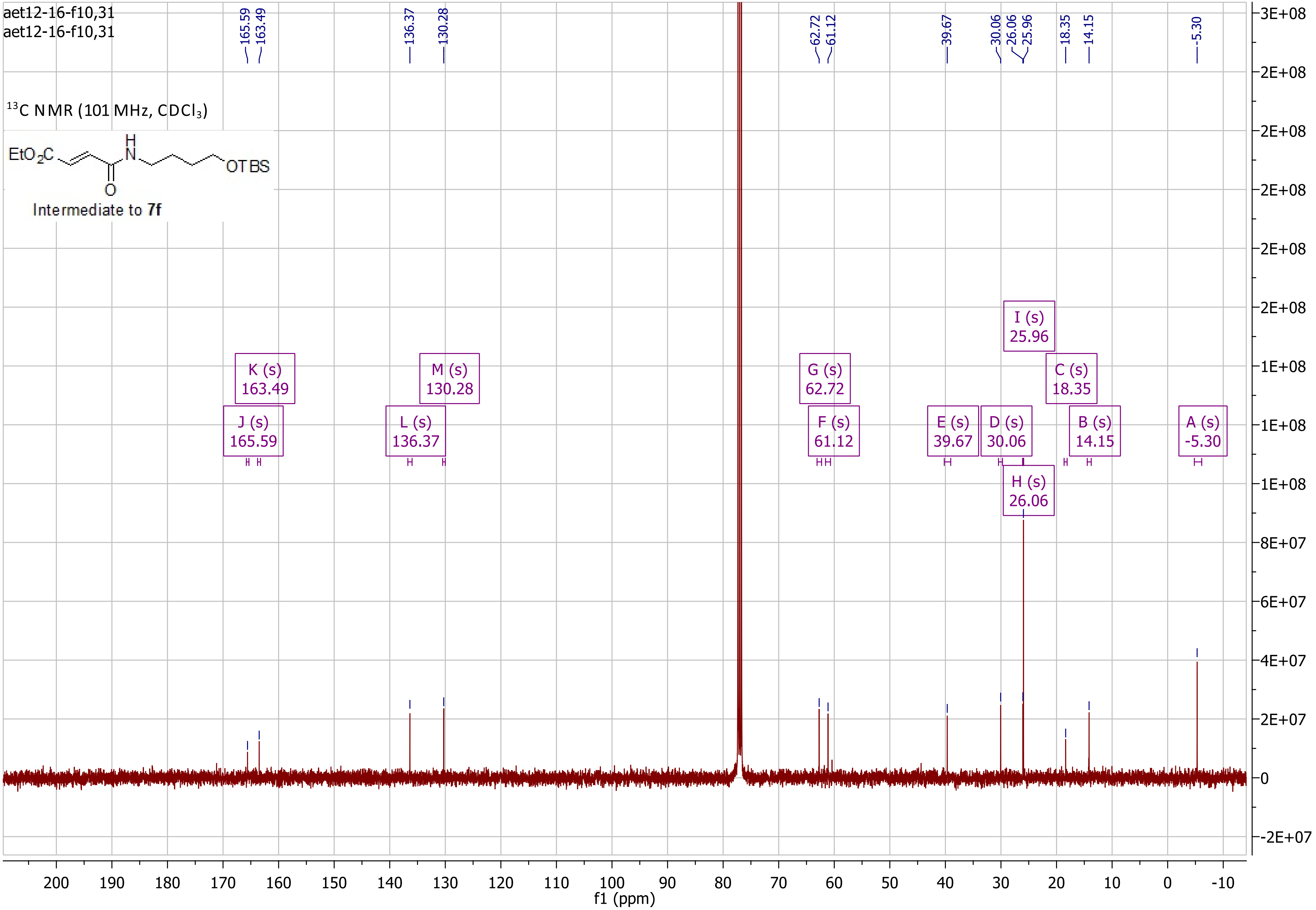
aet12-16-f10,31
aet12-16-f10,31
^{13}C NMR (101 MHz, CDCl$_3$)
EtO2C
H
N
OTBS
O
Intermediate to 7f
165.59
163.49
136.37
130.28
62.72
61.12
39.67
30.06
26.06
25.96
18.35
14.15
-5.30
K (s) 163.49
J (s) 165.59
M (s) 130.28
L (s) 136.37
G (s) 62.72
F (s) 61.12
E (s) 39.67
D (s) 30.06
I (s) 25.96
H (s) 26.06
C (s) 18.35
B (s) 14.15
A (s) -5.30
f1 (ppm)

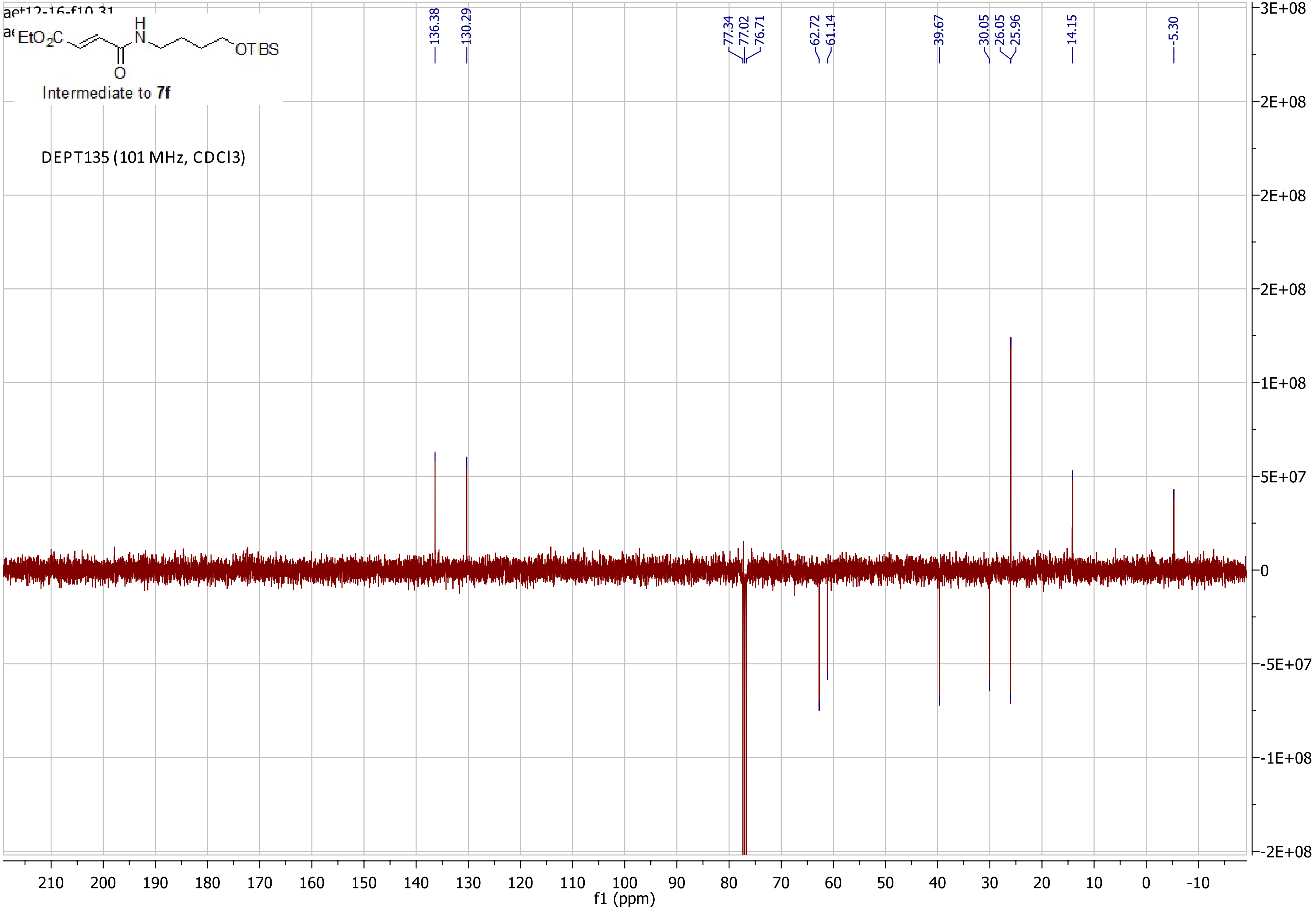

EtO2C
H
N
OTBS
O
Intermediate to 7f
DEPT135 (101 MHz, CDCl3)
136.38
130.29
77.34
77.02
76.71
62.72
61.14
39.67
30.05
26.05
25.96
14.15
-5.30
f1 (ppm)

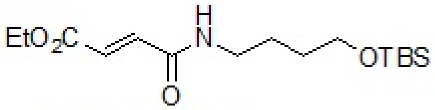

Intermediate to **7f**

COSY (400 MHz, CDCl3)

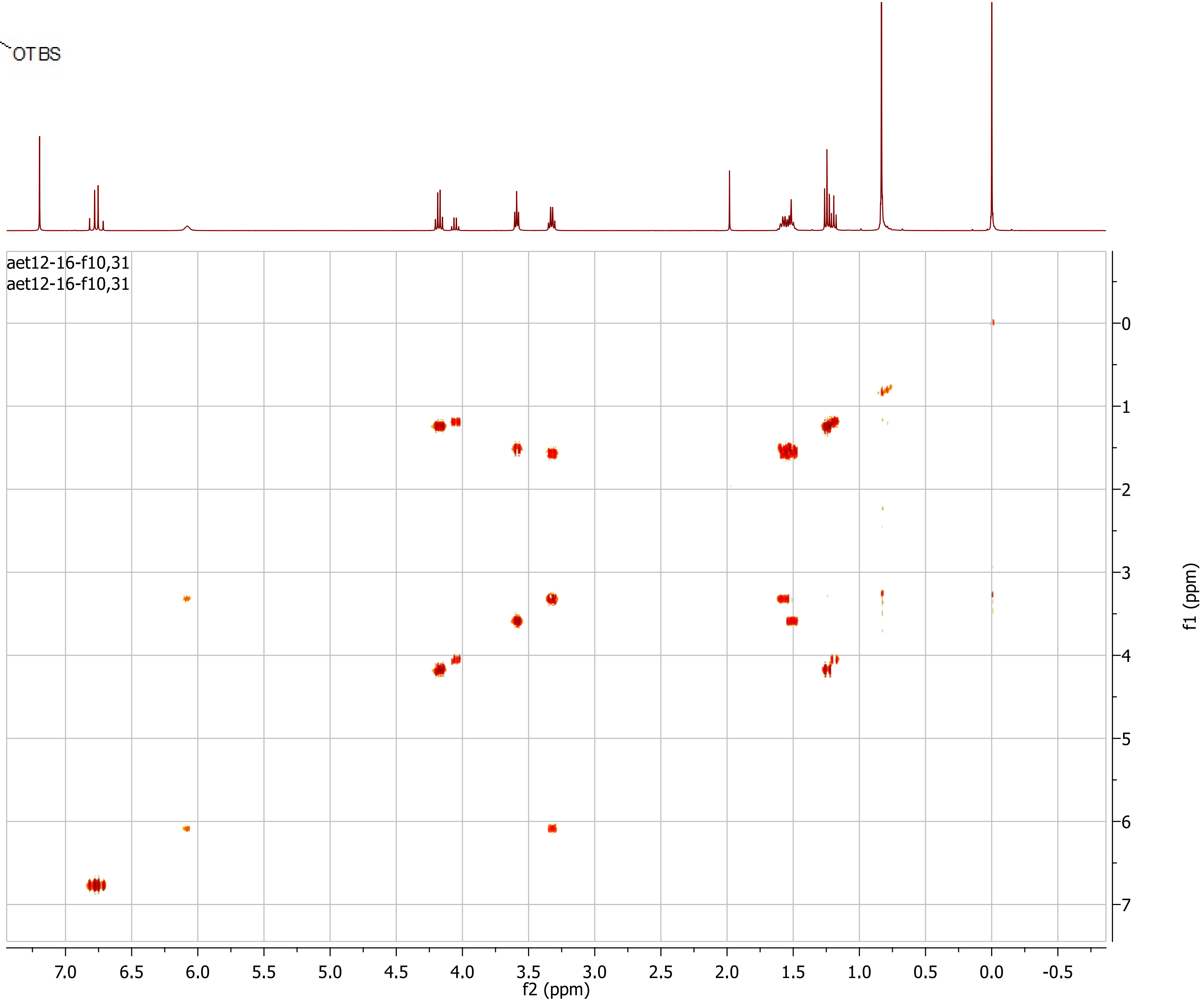

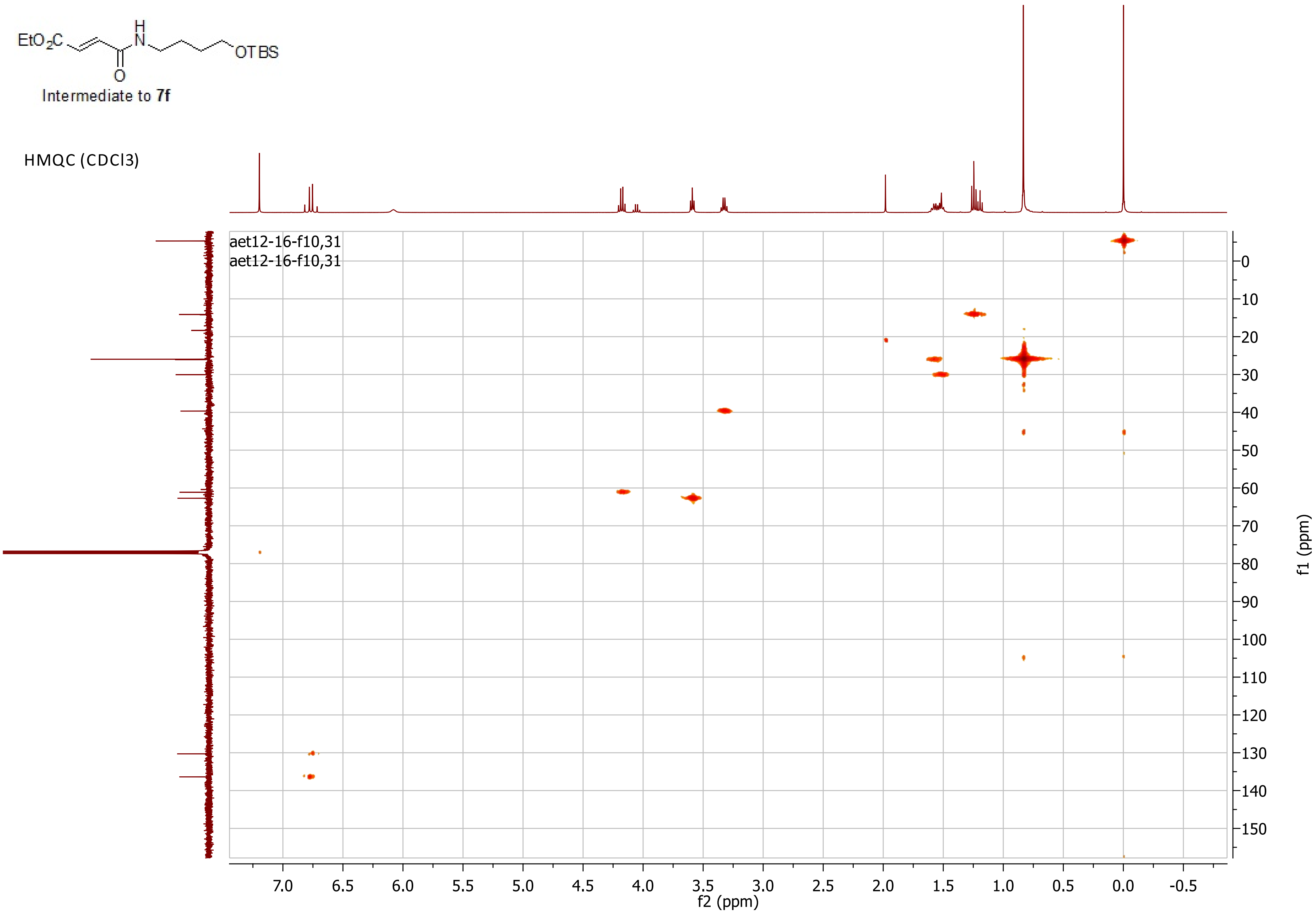
EtO2C
H
N
O
OTBS
Intermediate to 7f
HMQC (CDCl3)
aet12-16-f10,31
aet12-16-f10,31
f2 (ppm)
f1 (ppm)

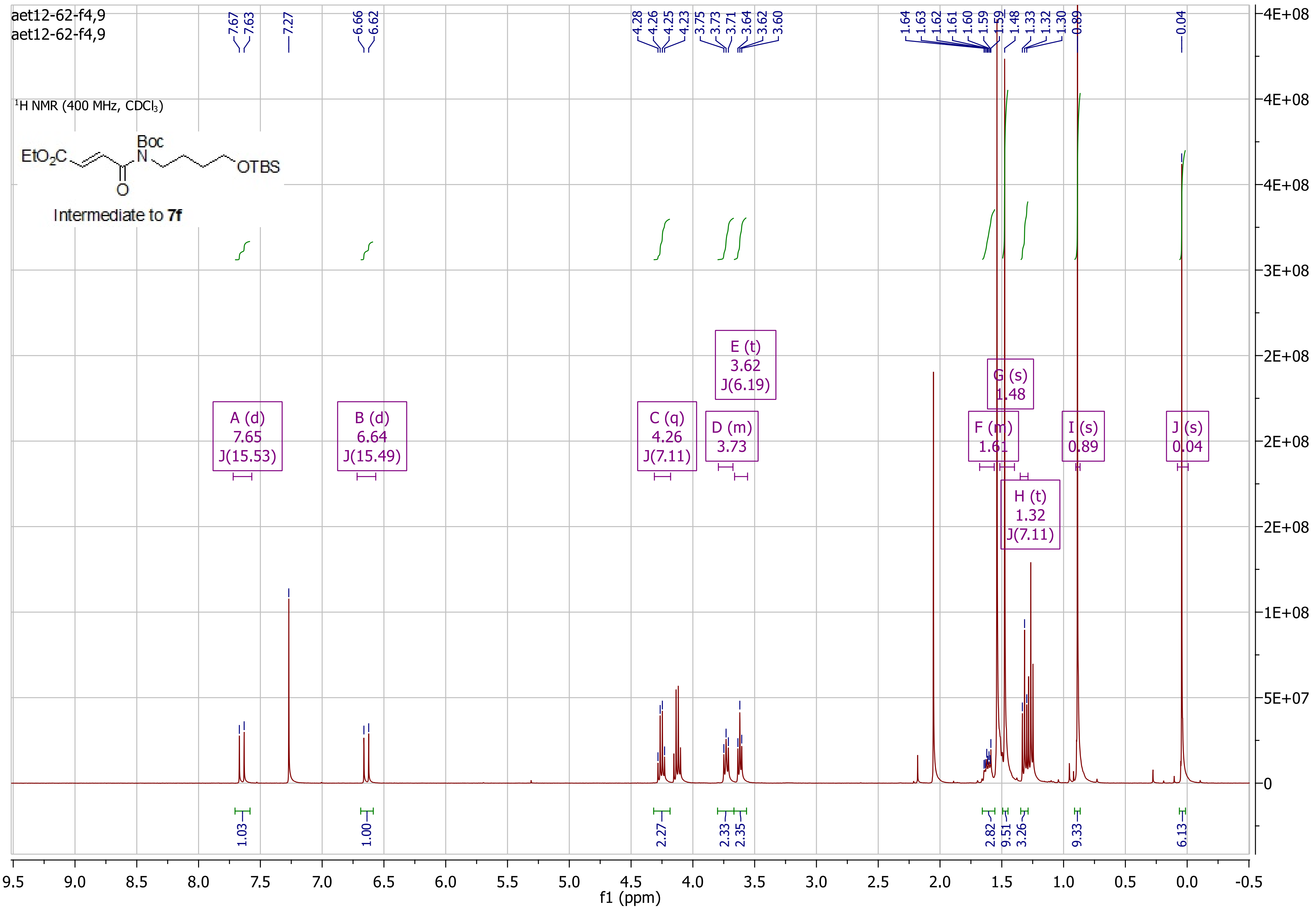
aet12-62-f4,9
aet12-62-f4,9
1H NMR (400 MHz, CDCl3)
EtO2C
Boc
N
OTBS
O
Intermediate to 7f
7.67
7.63
7.27
6.66
6.62
4.28
4.26
4.25
4.23
3.75
3.73
3.71
3.64
3.62
3.60
1.64
1.63
1.62
1.61
1.60
1.59
1.59
1.48
1.33
1.32
1.30
0.89
0.04
A (d) 7.65 J(15.53)
B (d) 6.64 J(15.49)
C (q) 4.26 J(7.11)
D (m) 3.73
E (t) 3.62 J(6.19)
F (m) 1.61
G (s) 1.48
H (t) 1.32 J(7.11)
I (s) 0.89
J (s) 0.04
1.03
1.00
2.27
2.33
2.35
2.82
9.51
3.26
9.33
6.13
f1 (ppm)

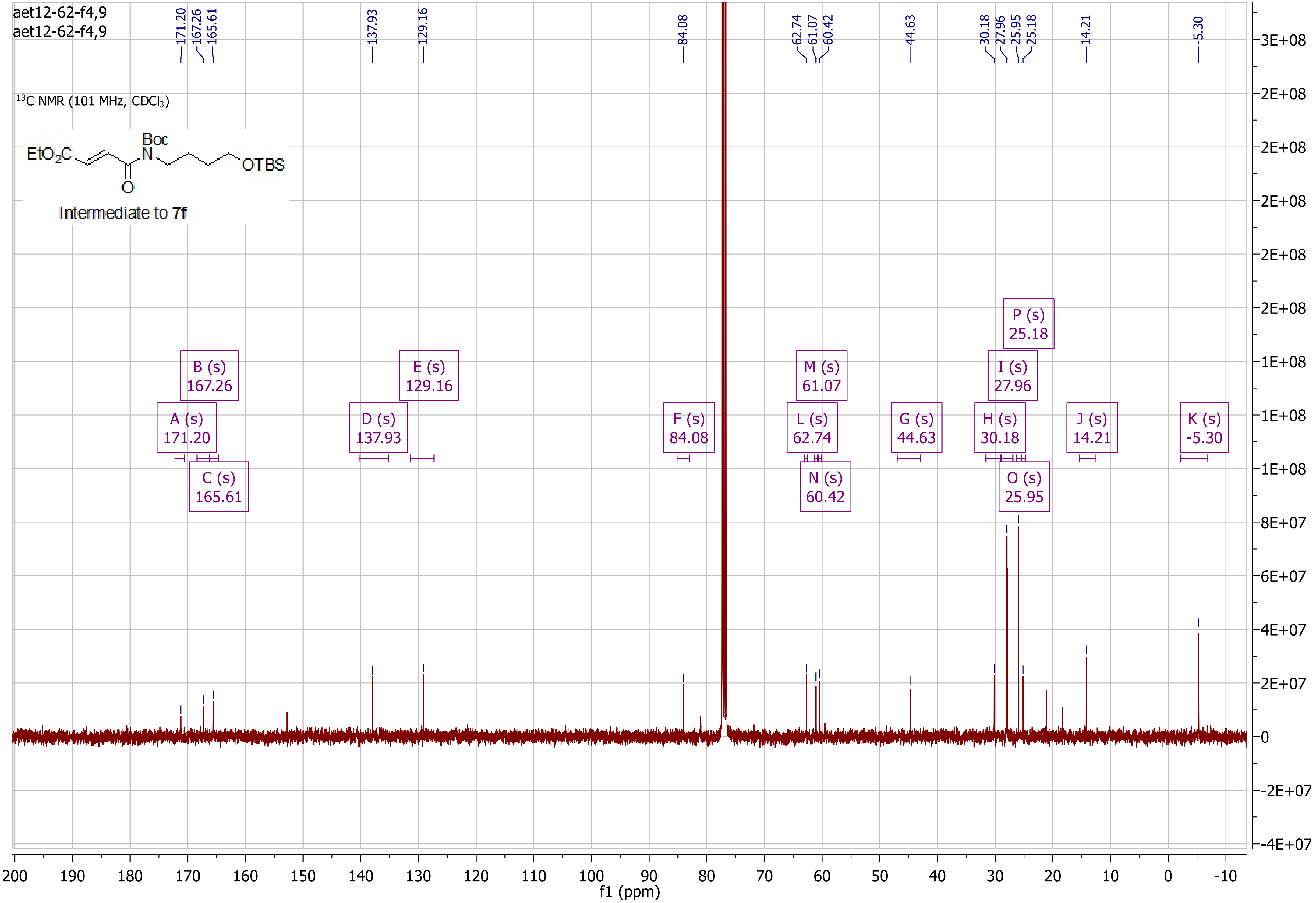
aet12-62-f4,9
aet12-62-f4,9
171.20
167.26
165.61
137.93
129.16
84.08
62.74
61.07
60.42
44.63
30.18
27.96
25.95
25.18
14.21
-5.30
^{13}C NMR (101 MHz, CDCl$_3$)
EtO_2C
Boc
N
OTBS
O
Intermediate to **7f**
A (s) 171.20
B (s) 167.26
C (s) 165.61
D (s) 137.93
E (s) 129.16
F (s) 84.08
G (s) 44.63
H (s) 30.18
I (s) 27.96
J (s) 14.21
K (s) -5.30
L (s) 62.74
M (s) 61.07
N (s) 60.42
O (s) 25.95
P (s) 25.18
3E+08
2E+08
2E+08
2E+08
2E+08
2E+08
1E+08
1E+08
1E+08
8E+07
6E+07
4E+07
2E+07
0
-2E+07
-4E+07
200
190
180
170
160
150
140
130
120
110
100
90
80
70
60
50
40
30
20
10
0
-10
f1 (ppm)

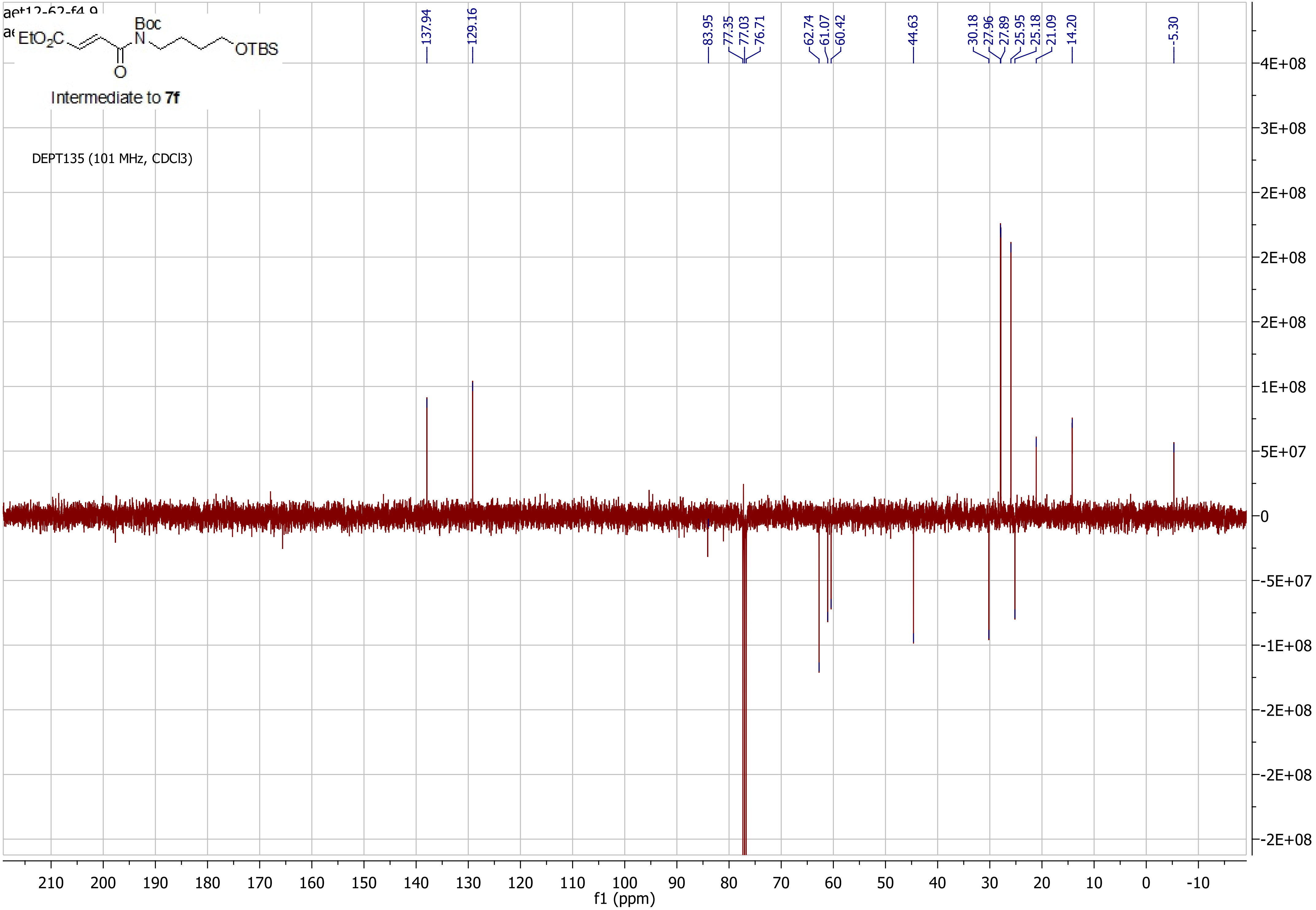

aet12-62-f4.9
EtO2C
Boc
N
O
OTBS
Intermediate to 7f
DEPT135 (101 MHz, CDCl3)
137.94
129.16
83.95
77.35
77.03
76.71
62.74
61.07
60.42
44.63
30.18
27.96
27.89
25.95
25.18
21.09
14.20
-5.30
4E+08
3E+08
2E+08
2E+08
2E+08
1E+08
5E+07
0
-5E+07
-1E+08
-2E+08
-2E+08
-2E+08
210
200
190
180
170
160
150
140
130
120
110
100
90
80
70
60
50
40
30
20
10
0
-10
f1 (ppm)

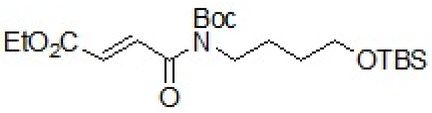

Intermediate to **7f**

COSY (400 MHz, CDCl3)

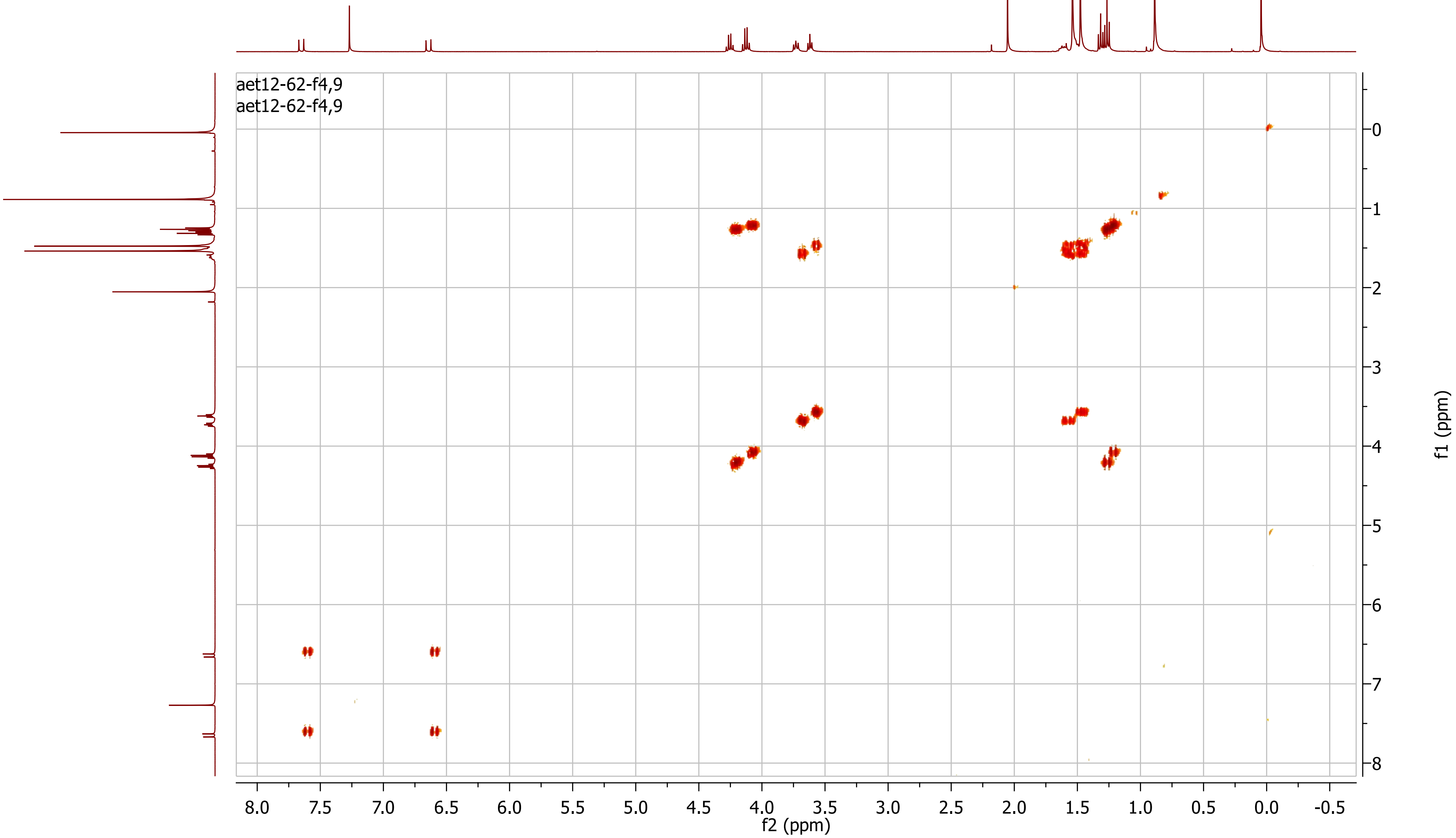

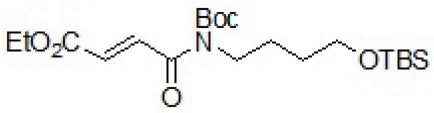

Intermediate to **7f**

HMQC (CDCl3)

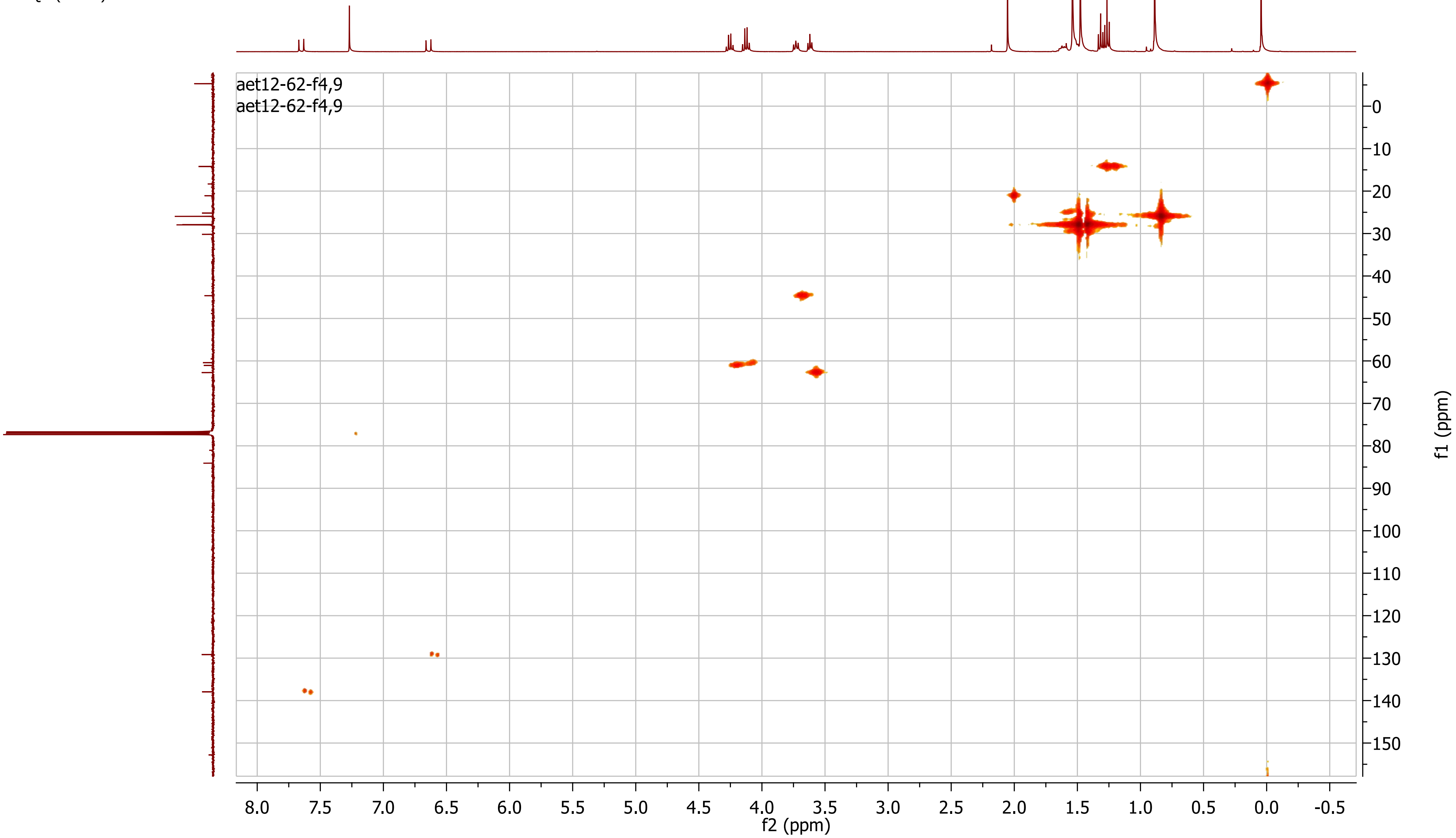

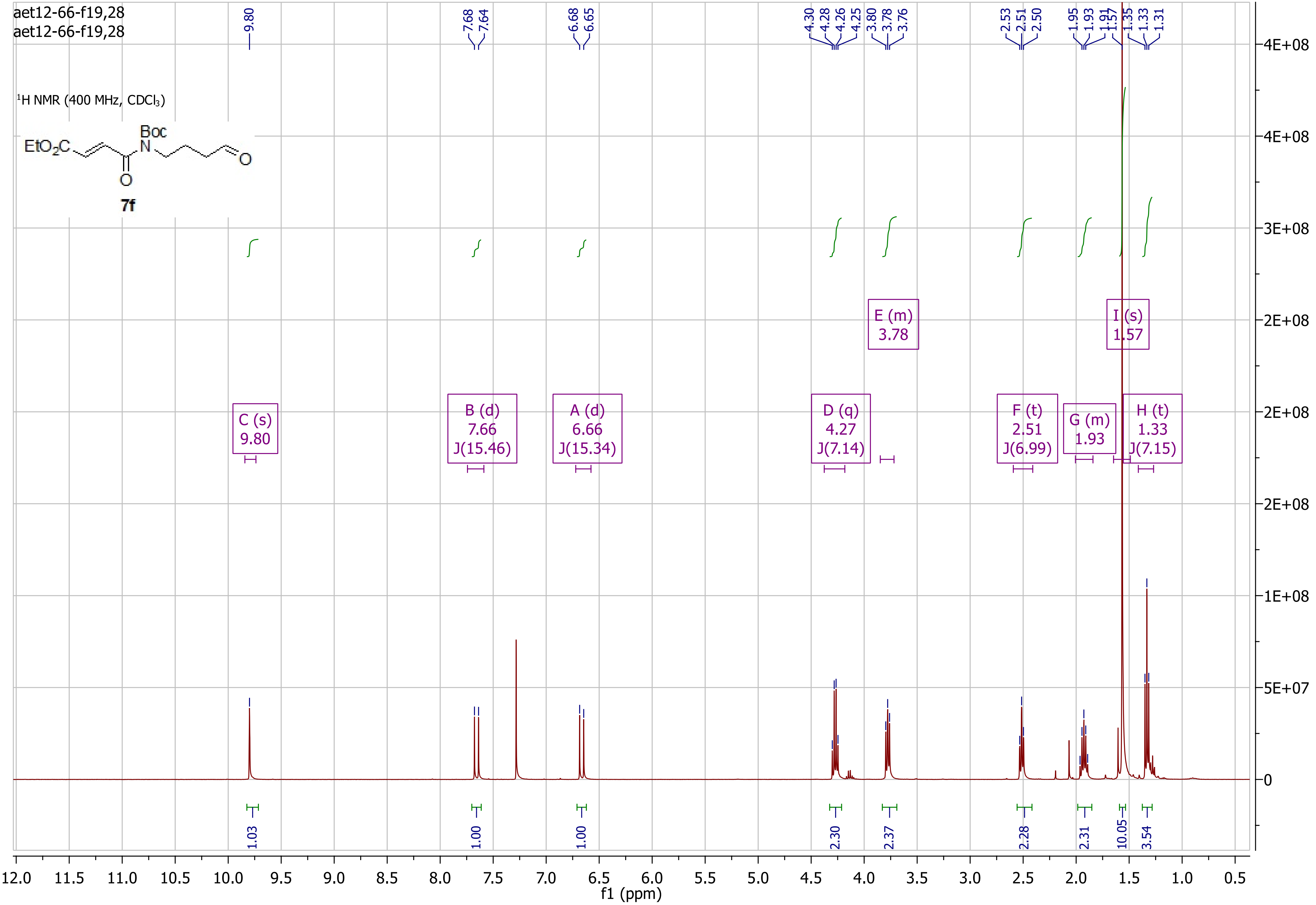
aet12-66-f19,28
aet12-66-f19,28
1H NMR (400 MHz, CDCl3)
EtO2C
Boc
N
O
O
7f
9.80
7.68
7.64
6.68
6.65
4.30
4.28
4.26
4.25
3.80
3.78
3.76
2.53
2.51
2.50
1.95
1.93
1.91
1.57
1.35
1.33
1.31
C (s) 9.80
B (d) 7.66 J(15.46)
A (d) 6.66 J(15.34)
D (q) 4.27 J(7.14)
E (m) 3.78
F (t) 2.51 J(6.99)
G (m) 1.93
I (s) 1.57
H (t) 1.33 J(7.15)
1.03
1.00
1.00
2.30
2.37
2.28
2.31
10.05
3.54
4E+08
4E+08
3E+08
2E+08
2E+08
2E+08
1E+08
5E+07
0
12.0
11.5
11.0
10.5
10.0
9.5
9.0
8.5
8.0
7.5
7.0
6.5
6.0
5.5
5.0
4.5
4.0
3.5
3.0
2.5
2.0
1.5
1.0
0.5
f1 (ppm)

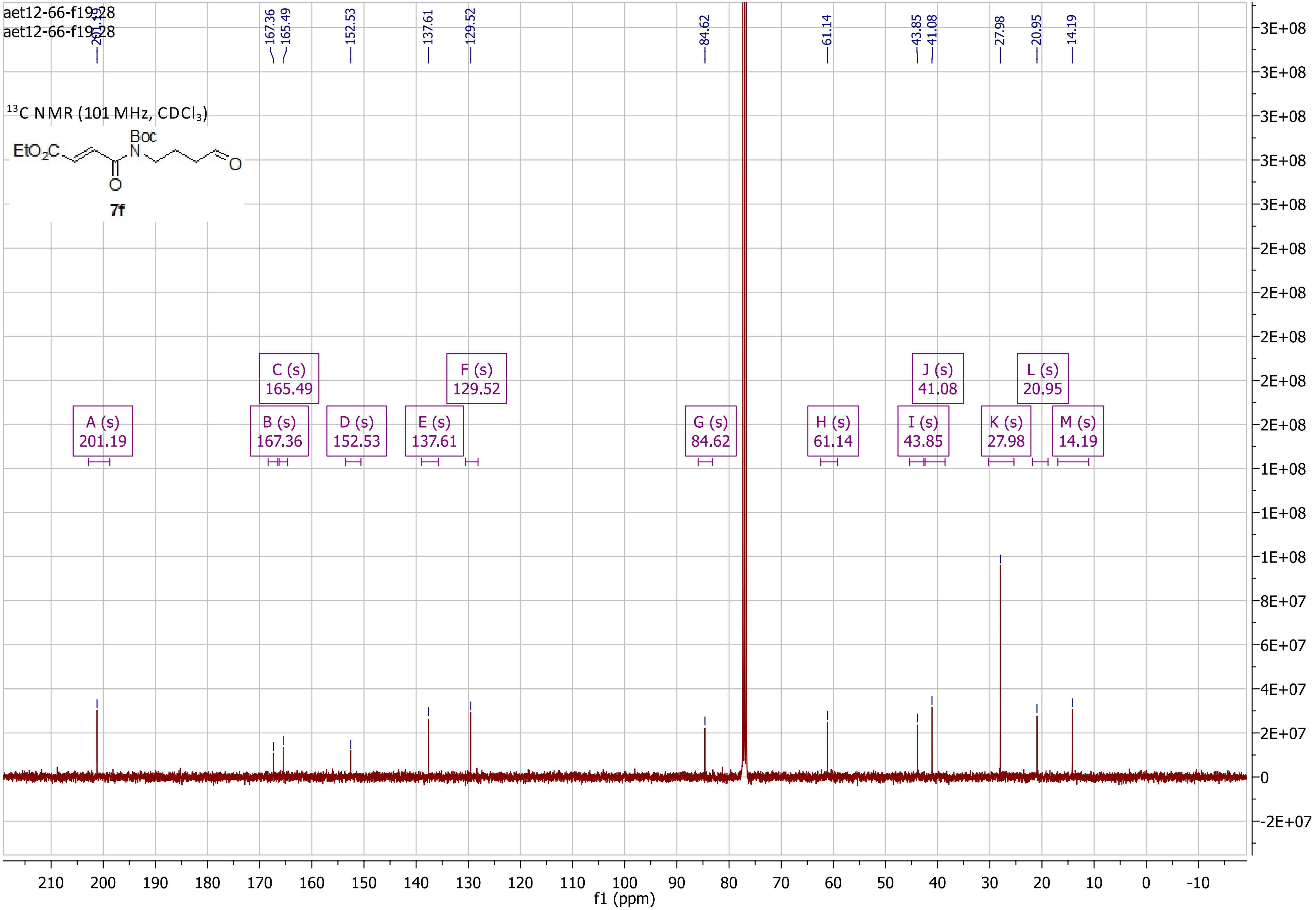
aet12-66-f19-28
aet12-66-f19-28
201.19
167.36
165.49
152.53
137.61
129.52
84.62
61.14
43.85
41.08
27.98
20.95
14.19
13C NMR (101 MHz, CDCl3)
Boc
EtO2C
N
O
O
7f
A (s) 201.19
B (s) 167.36
C (s) 165.49
D (s) 152.53
E (s) 137.61
F (s) 129.52
G (s) 84.62
H (s) 61.14
I (s) 43.85
J (s) 41.08
K (s) 27.98
L (s) 20.95
M (s) 14.19
f1 (ppm)

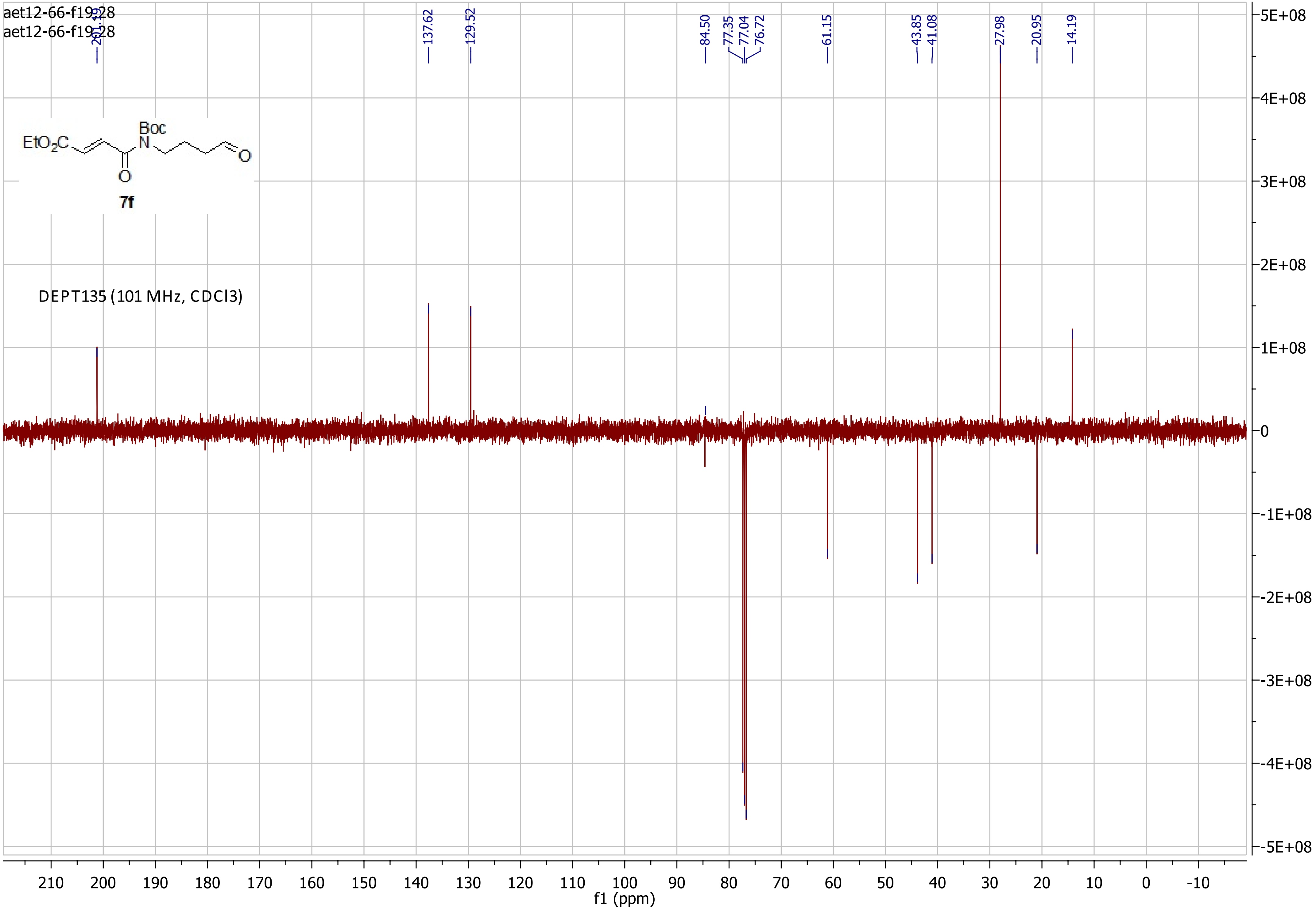
aet12-66-f19.28
aet12-66-f19.28
137.62
129.52
84.50
77.35
77.04
76.72
61.15
43.85
41.08
27.98
20.95
14.19
Boc
EtO2C
N
O
O
7f
DEPT135 (101 MHz, CDCl3)
5E+08
4E+08
3E+08
2E+08
1E+08
0
-1E+08
-2E+08
-3E+08
-4E+08
-5E+08
210 200 190 180 170 160 150 140 130 120 110 100 90 80 70 60 50 40 30 20 10 0 -10
f1 (ppm)

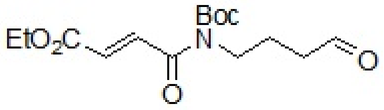

7f

COSY (400 MHz, CDCl3)

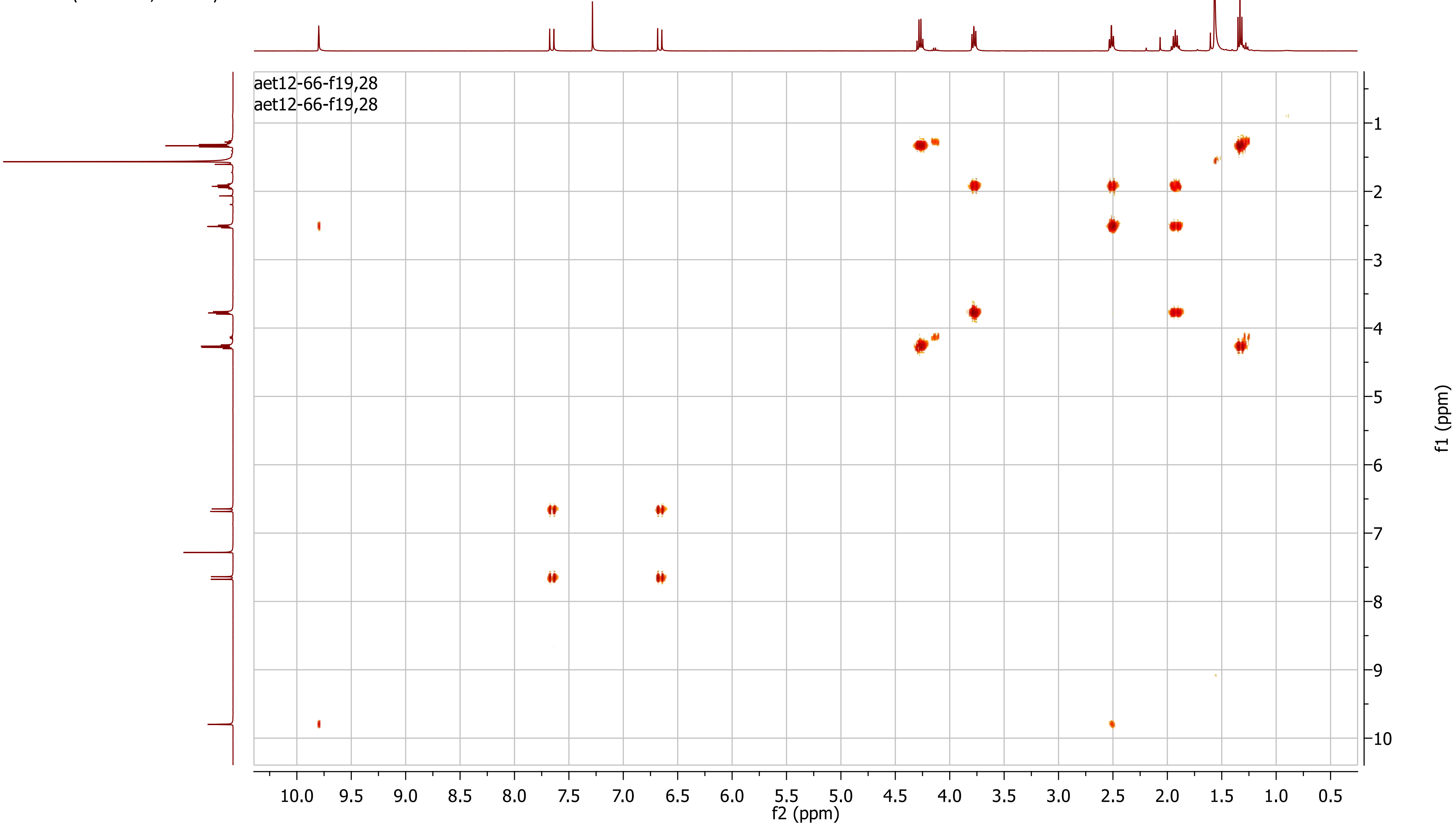

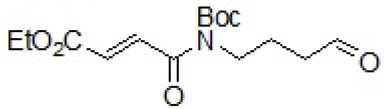

7f

HMQC (CDCl3)

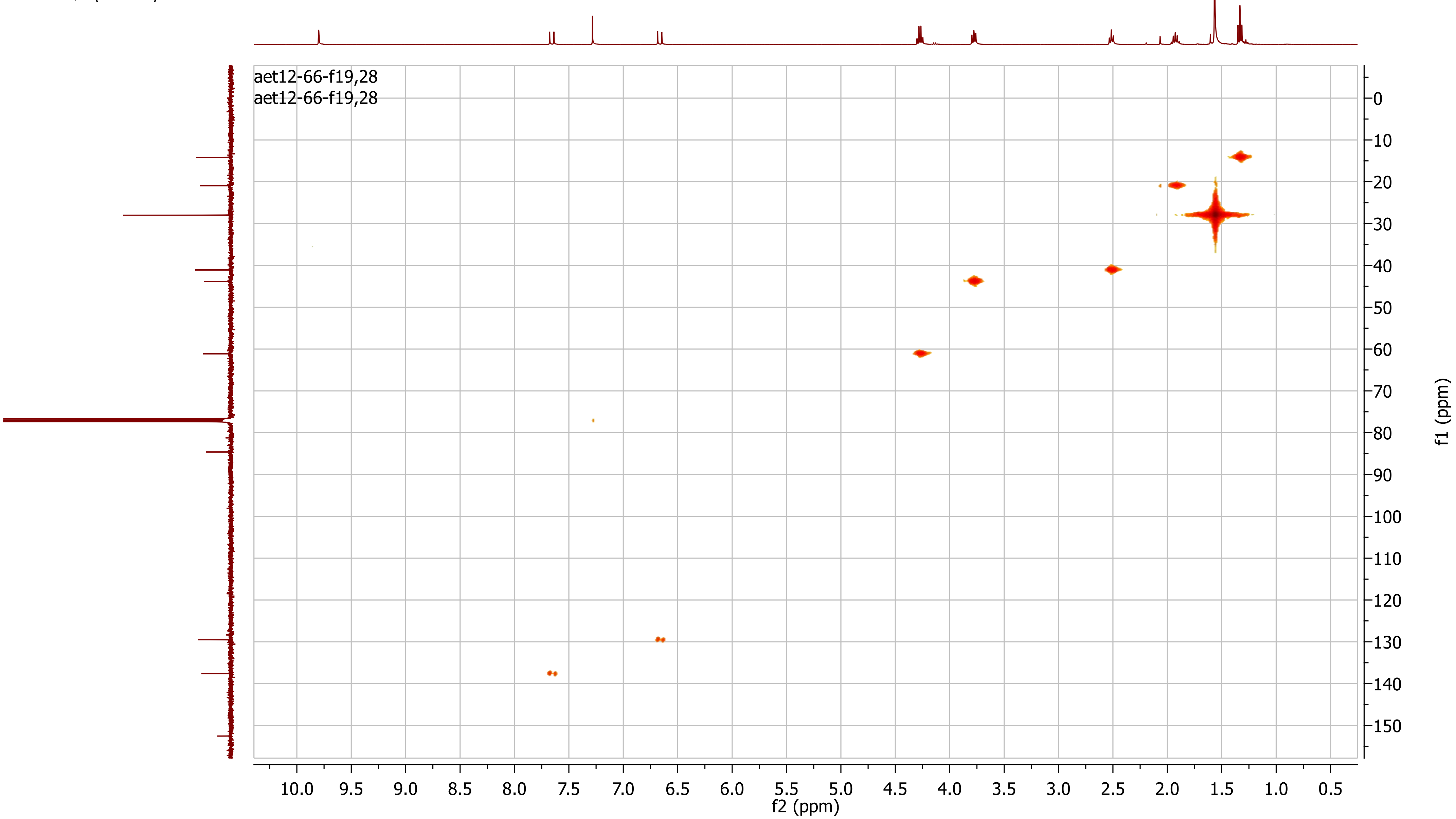

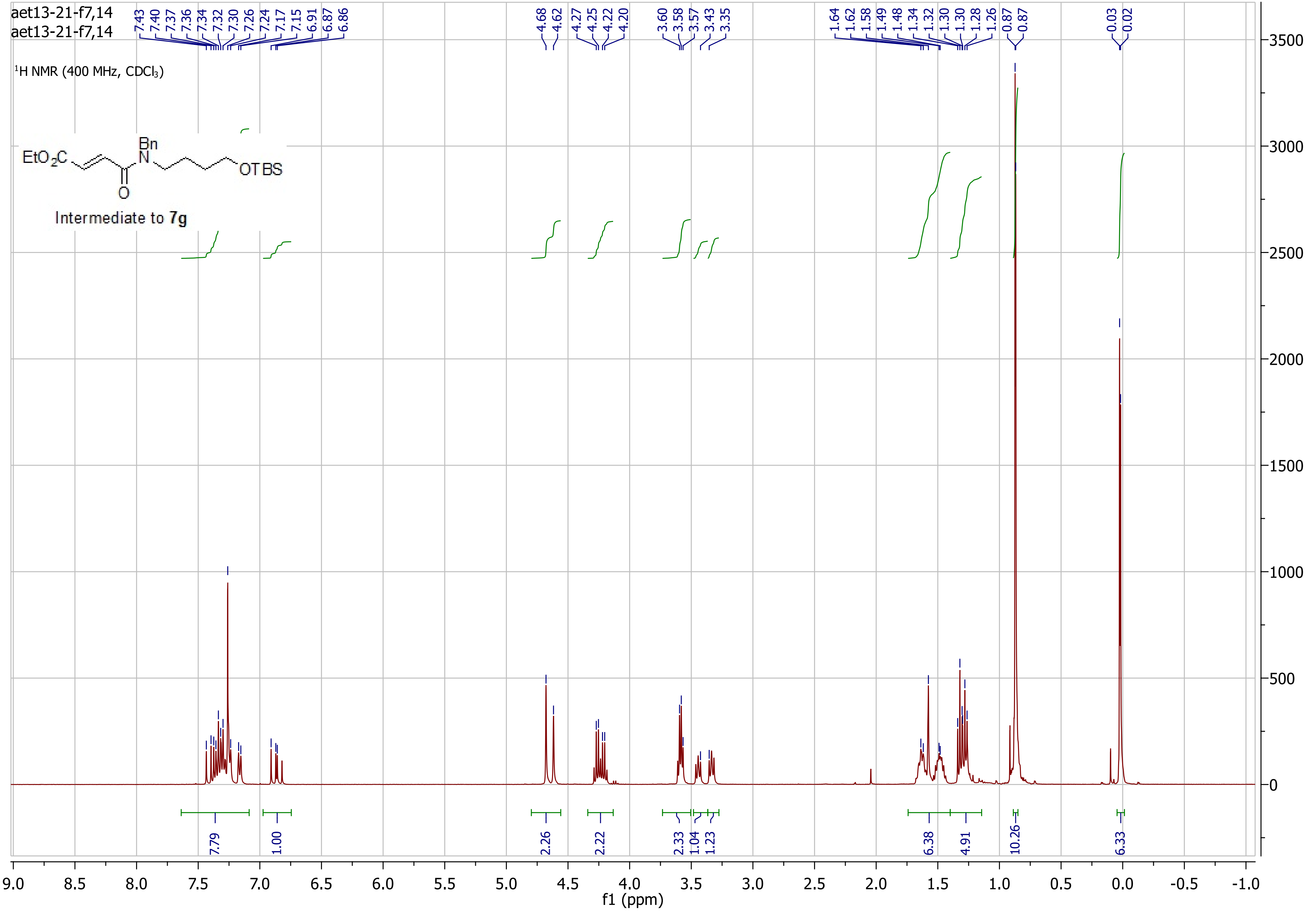

aet13-21-f7,14
aet13-21-f7,14
1H NMR (400 MHz, CDCl3)
EtO2C
Bn
N
OTBS
O
Intermediate to 7g
7.43 7.40 7.37 7.36 7.34 7.32 7.30 7.26 7.24 7.17 7.15 6.91 6.87 6.86
4.68 4.62 4.27 4.25 4.22 4.20
3.60 3.58 3.57 3.43 3.35
1.64 1.62 1.58 1.49 1.48 1.34 1.32 1.30 1.30 1.28 1.26 0.87 0.87
0.03 0.02
7.79 1.00 2.26 2.22 2.33 1.04 1.23 6.38 4.91 10.26 6.33
f1 (ppm)

aet13-10-f10,15
aet13-10-f10,15

^{13}C NMR (101 MHz, $CDCl_3$)

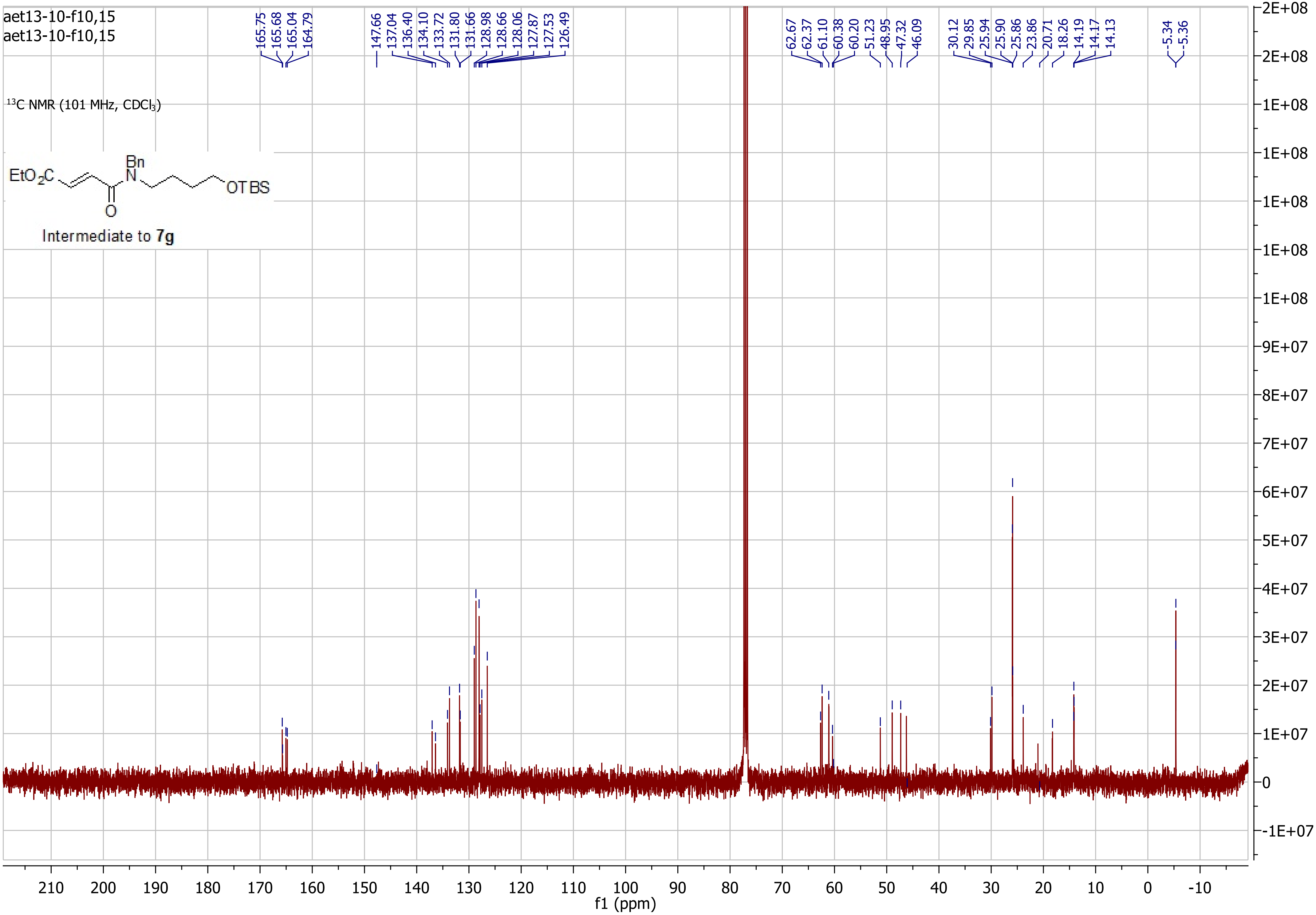

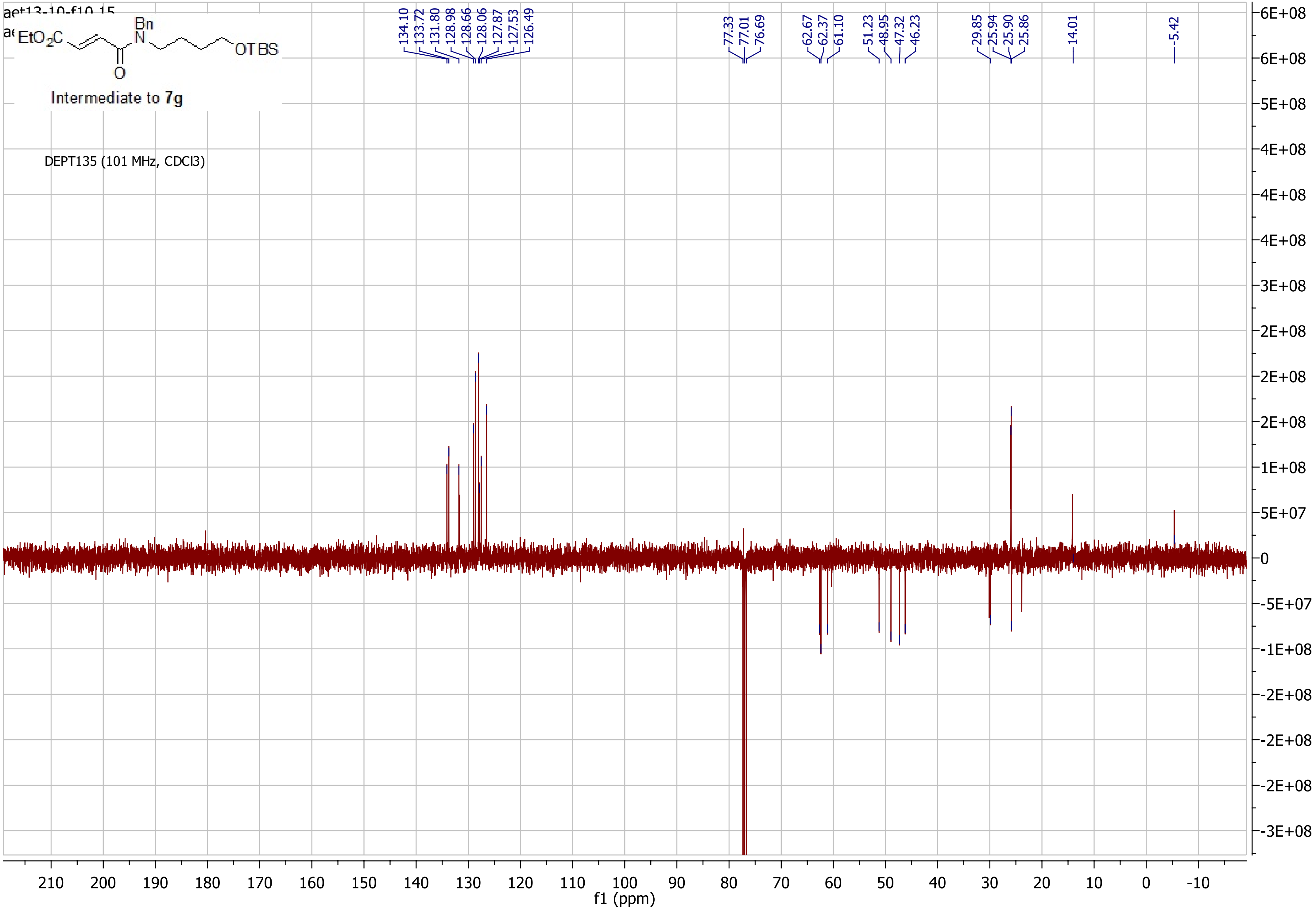

Intermediate to 7g
EtO2C
Bn
N
O
OTBS
DEPT135 (101 MHz, CDCl3)
134.10
133.72
131.80
128.98
128.66
128.06
127.87
127.53
126.49
77.33
77.01
76.69
62.67
62.37
61.10
51.23
48.95
47.32
46.23
29.85
25.94
25.90
25.86
14.01
-5.42
f1 (ppm)

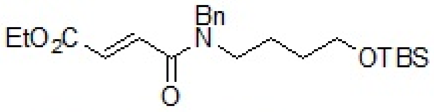

Intermediate to **7g**

COSY (400 MHz, CDCl3)

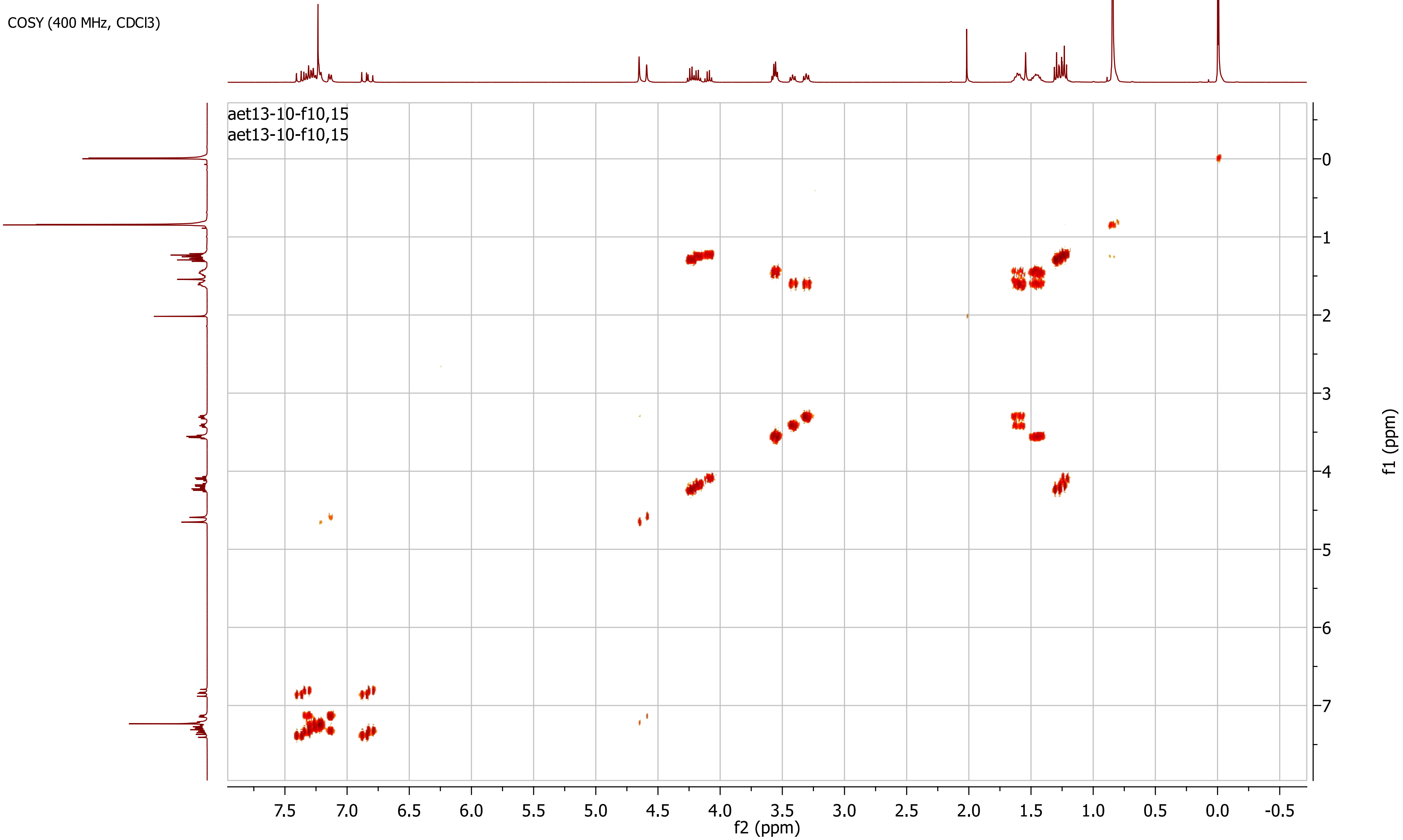

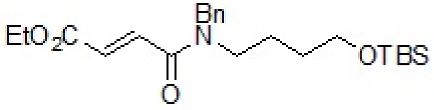

Intermediate to **7g**

DEPT (CDCl3)

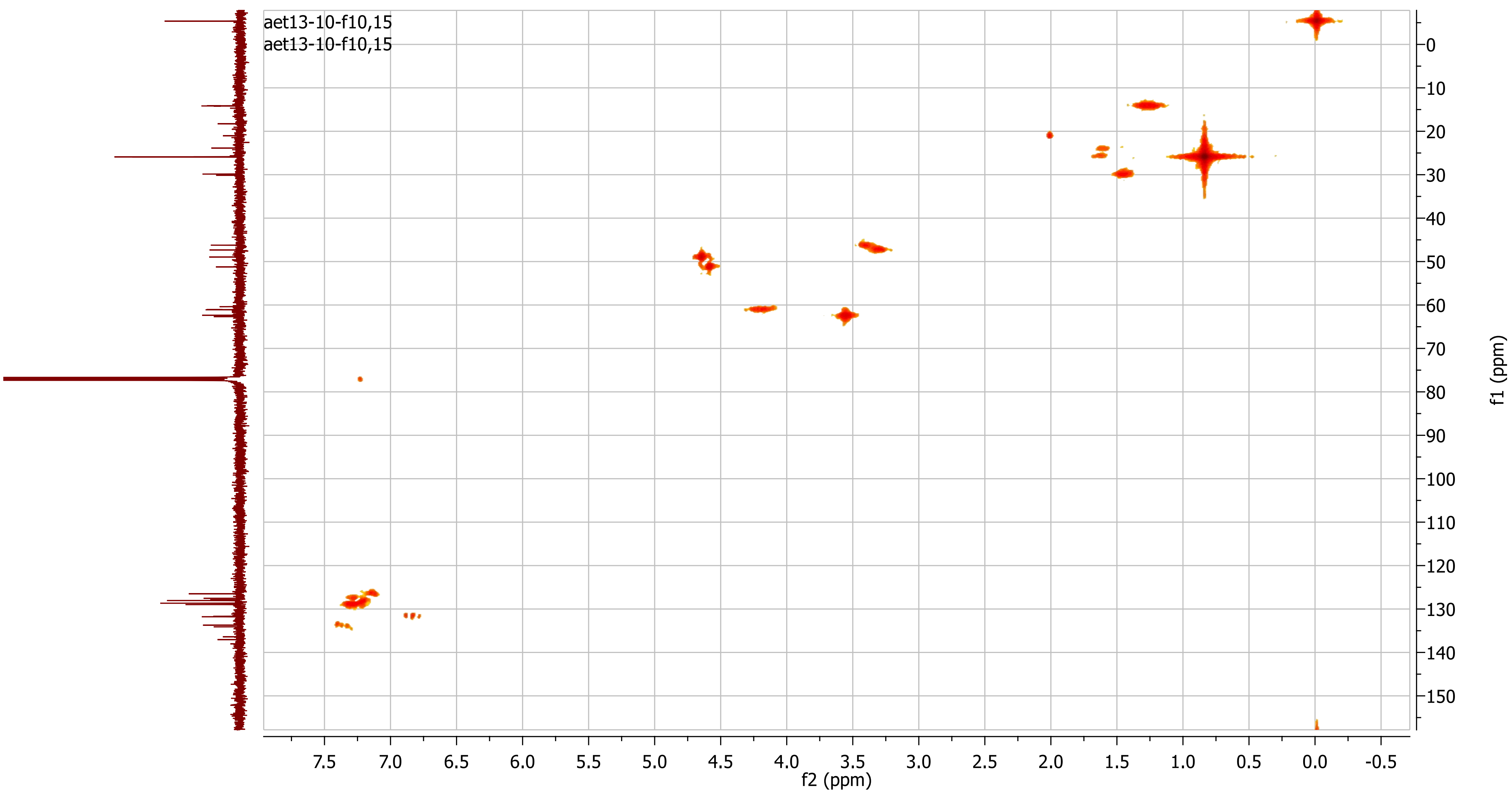

aet13-23-f24,40

aet13-23-f24,40

^{1}H NMR (400 MHz, $CDCl_3$)

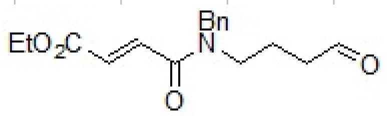

7g

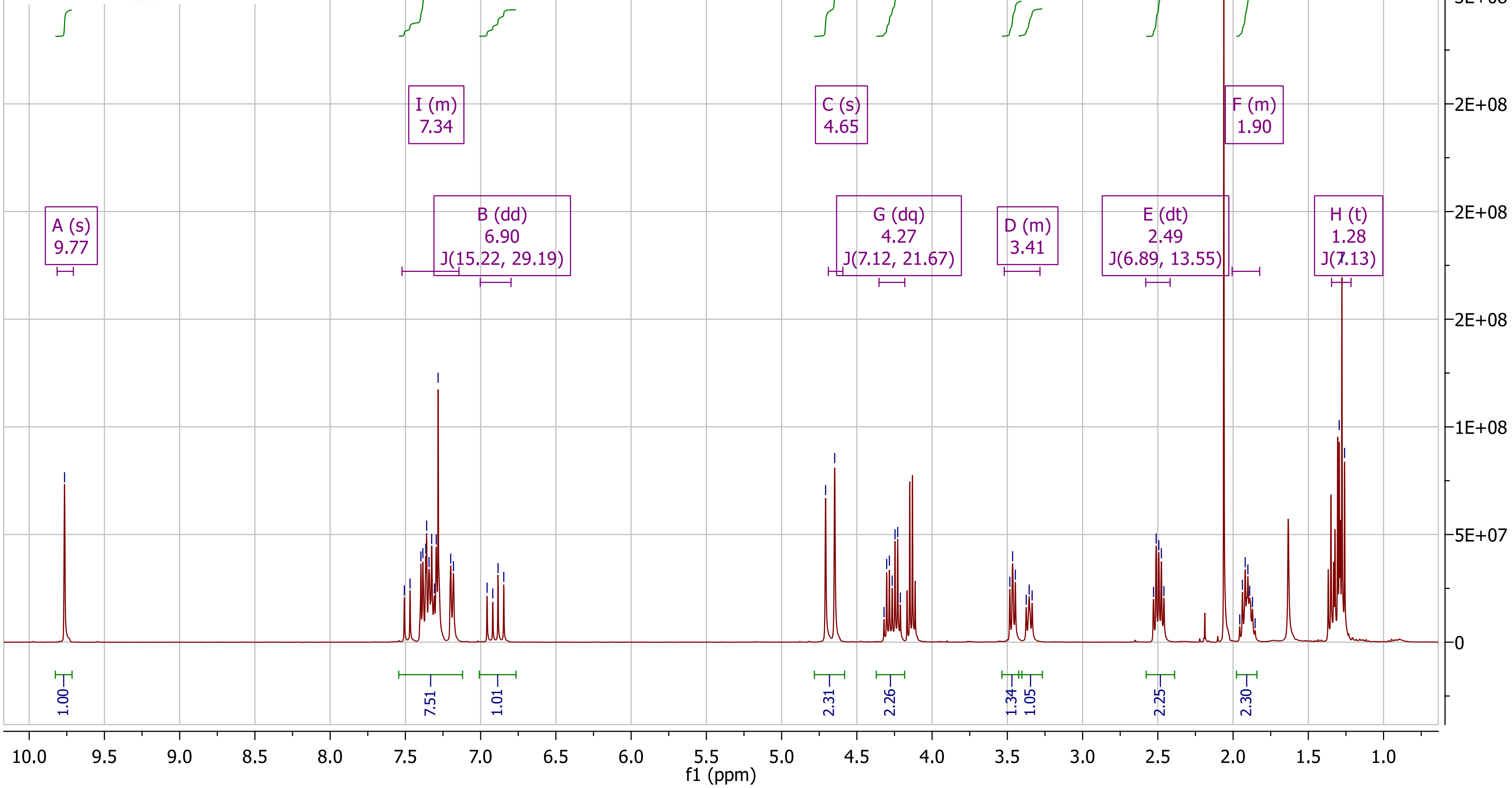

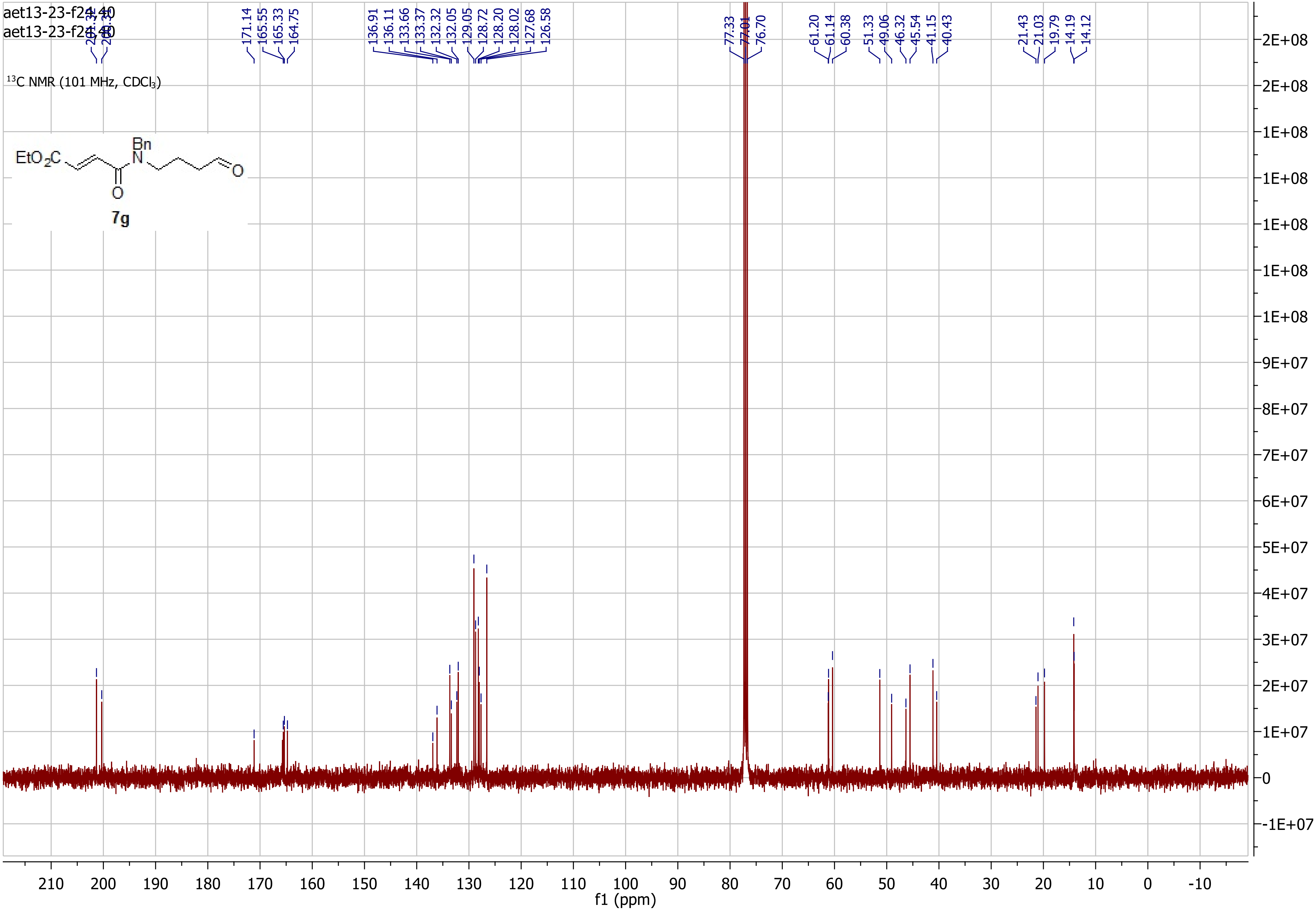

13C NMR (101 MHz, CDCl3)
171.14
165.55
165.33
164.75
136.91
136.11
133.66
133.37
132.32
132.05
129.05
128.72
128.20
128.02
127.68
126.58
77.33
77.01
76.70
61.20
61.14
60.38
51.33
49.06
46.32
45.54
41.15
40.43
21.43
21.03
19.79
14.19
14.12
EtO2C
Bn
N
O
O
7g
f1 (ppm)

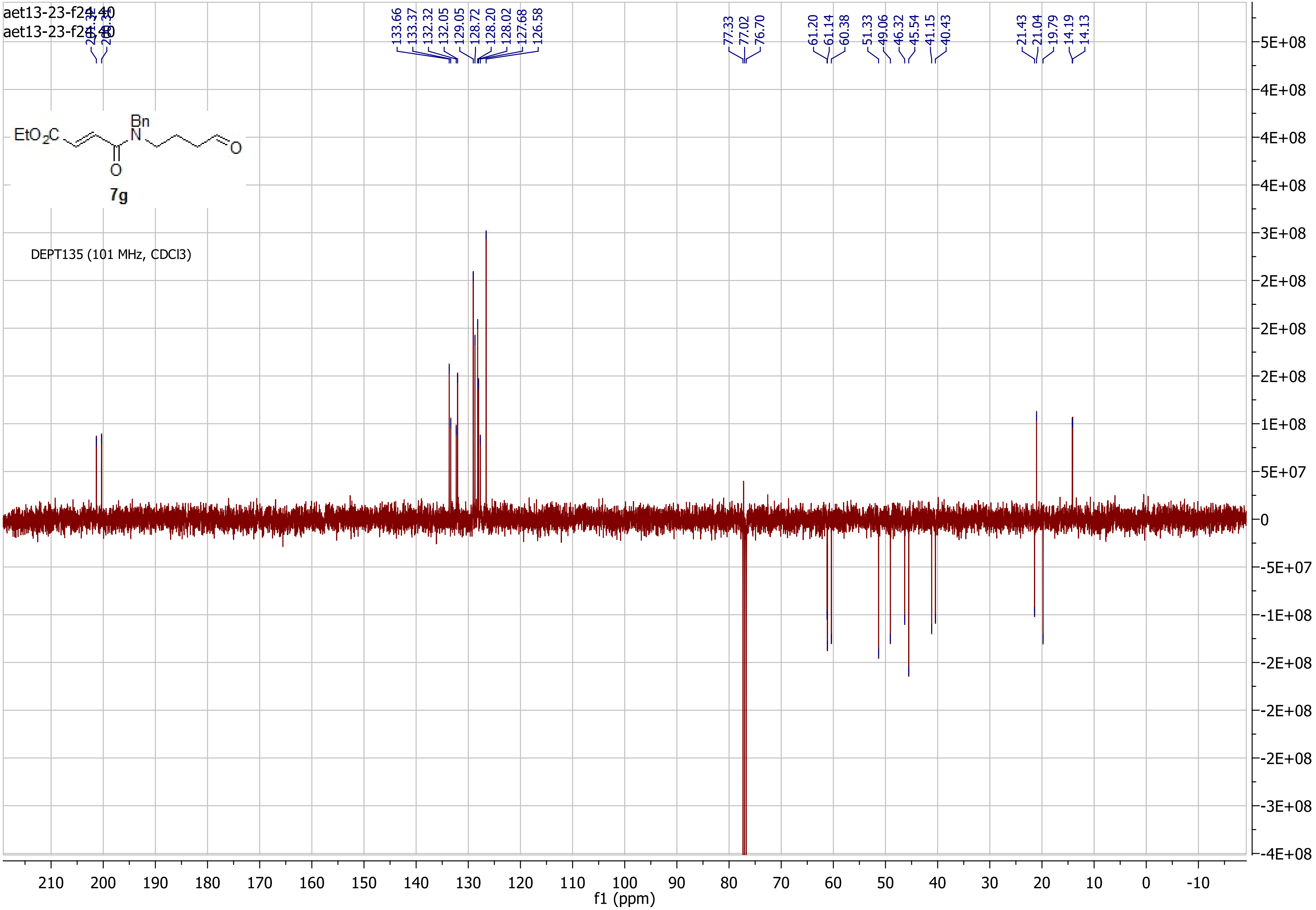

aet13-23-f24.40
133.66
133.37
132.32
132.05
129.05
128.72
128.20
128.02
127.68
126.58
77.33
77.02
76.70
61.20
61.14
60.38
51.33
49.06
46.32
45.54
41.15
40.43
21.43
21.04
19.79
14.19
14.13
EtO2C
Bn
N
O
O
7g
DEPT135 (101 MHz, CDCl3)
f1 (ppm)

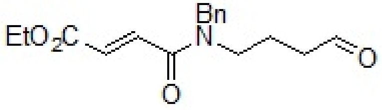

7h

COSY (400 MHz, CDCl3)

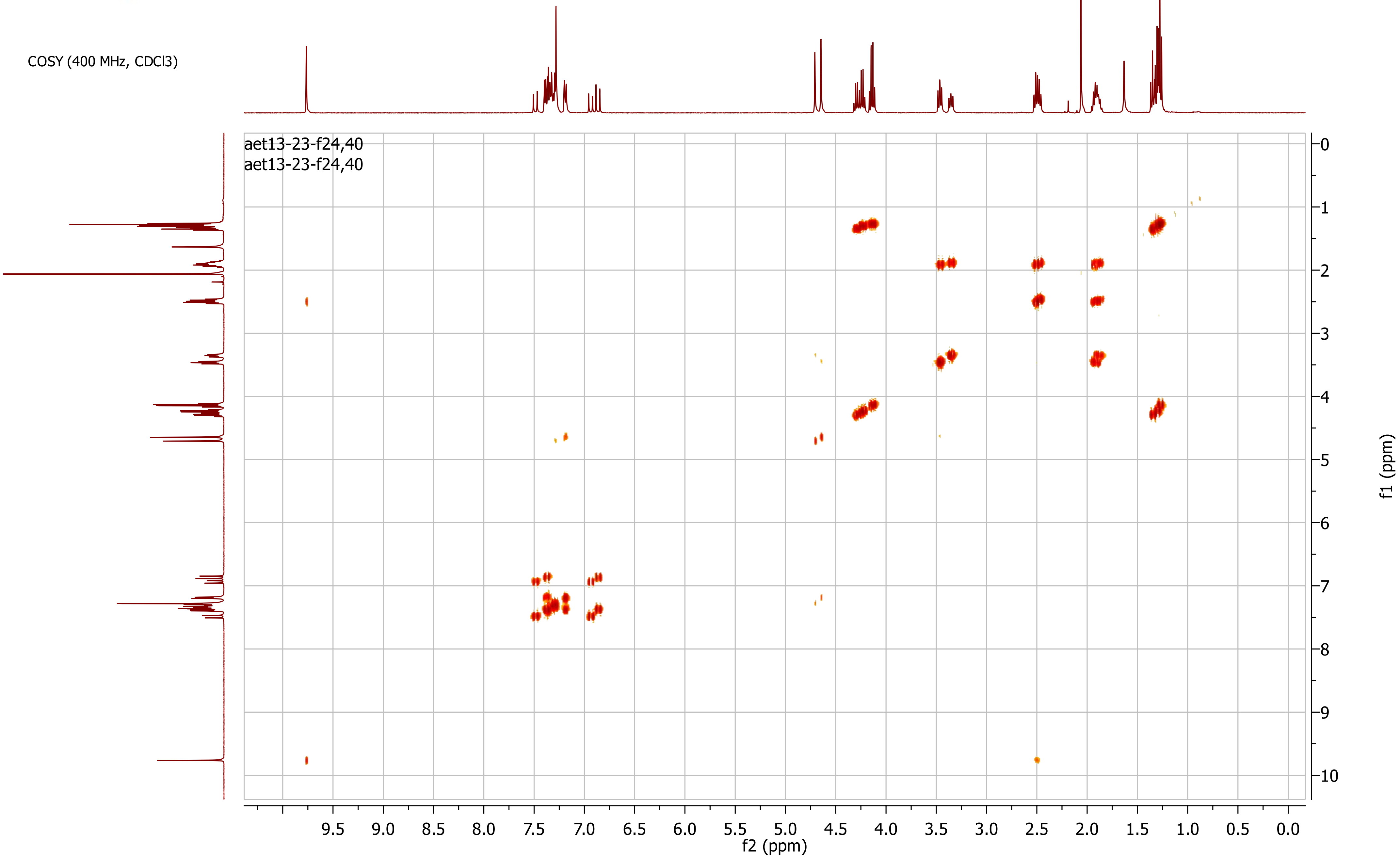

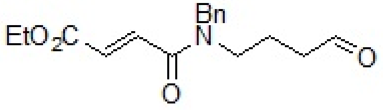

7g

HMQC (CDCl3)

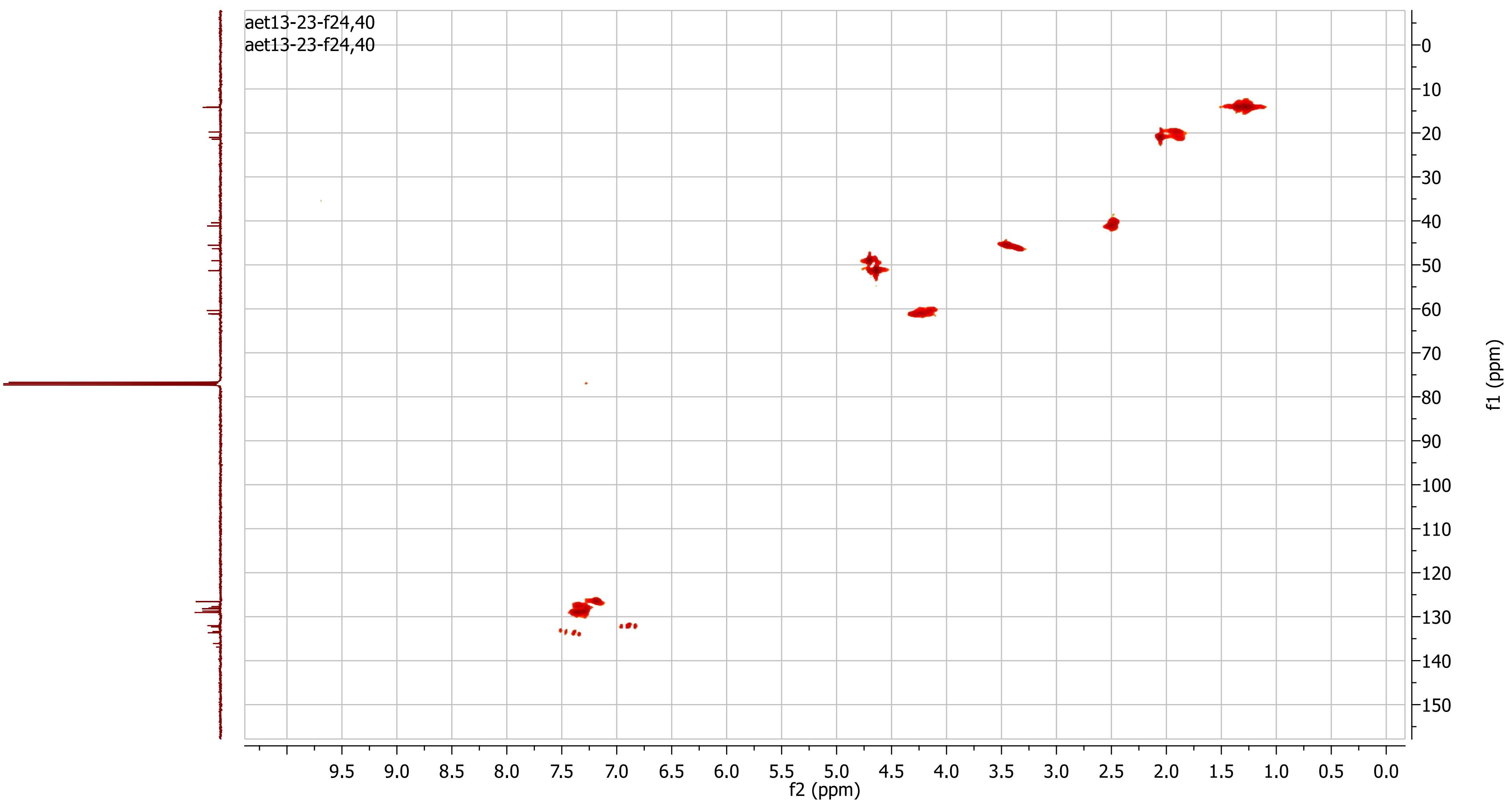

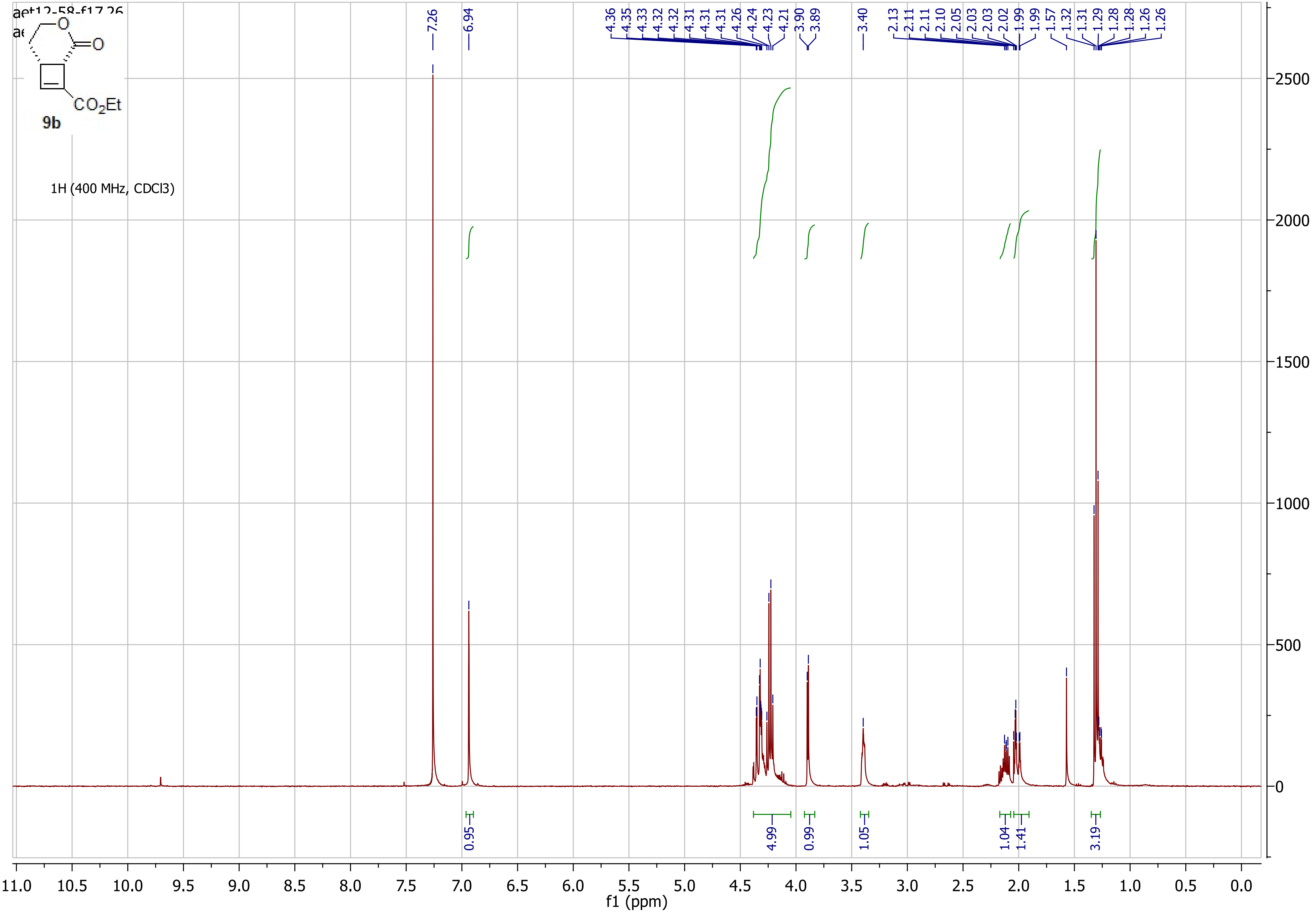

CO2Et
9b
1H (400 MHz, CDCl3)
f1 (ppm)

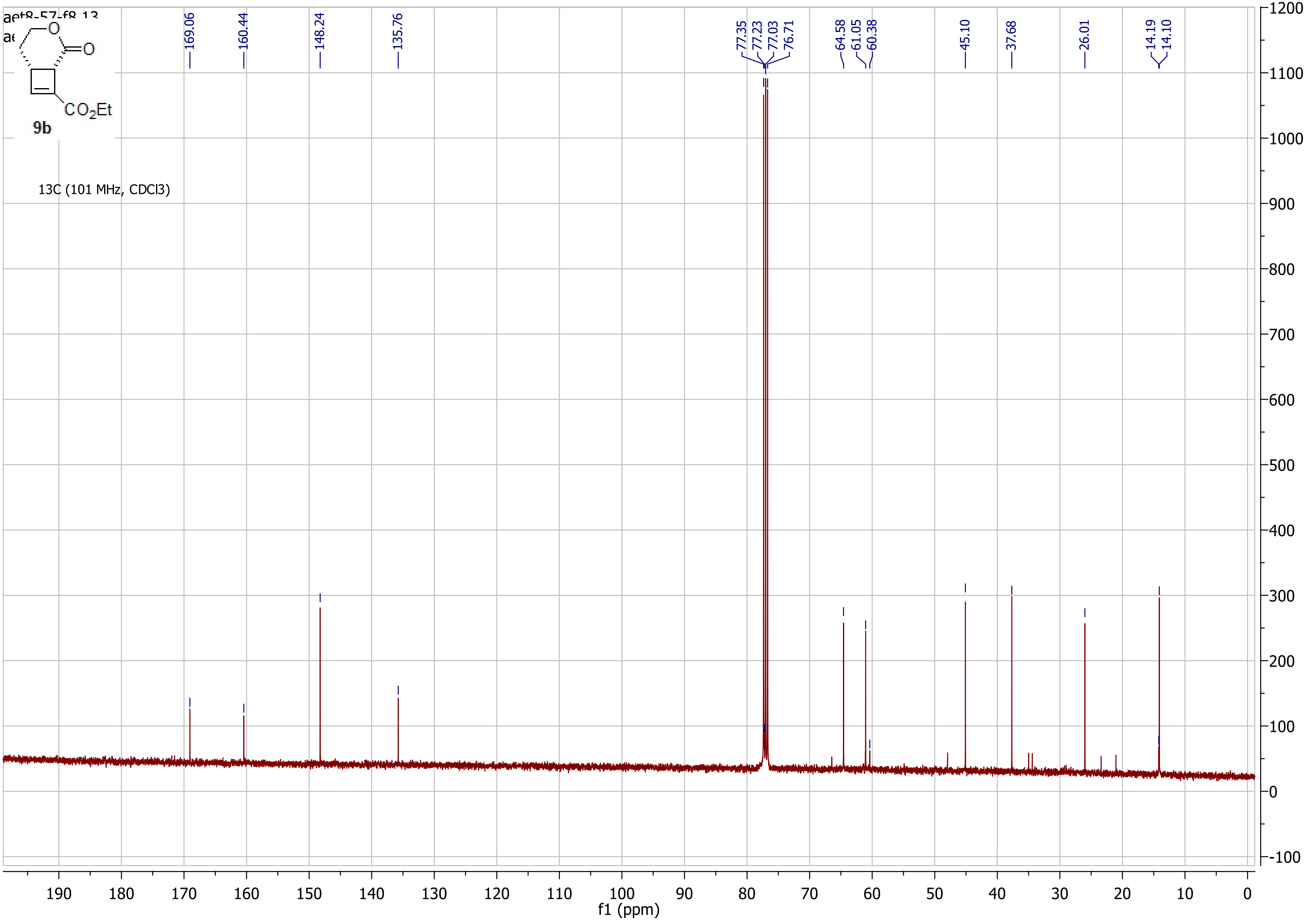

CO2Et
9b
13C (101 MHz, CDCl3)
169.06
160.44
148.24
135.76
77.35
77.23
77.03
76.71
64.58
61.05
60.38
45.10
37.68
26.01
14.19
14.10
f1 (ppm)

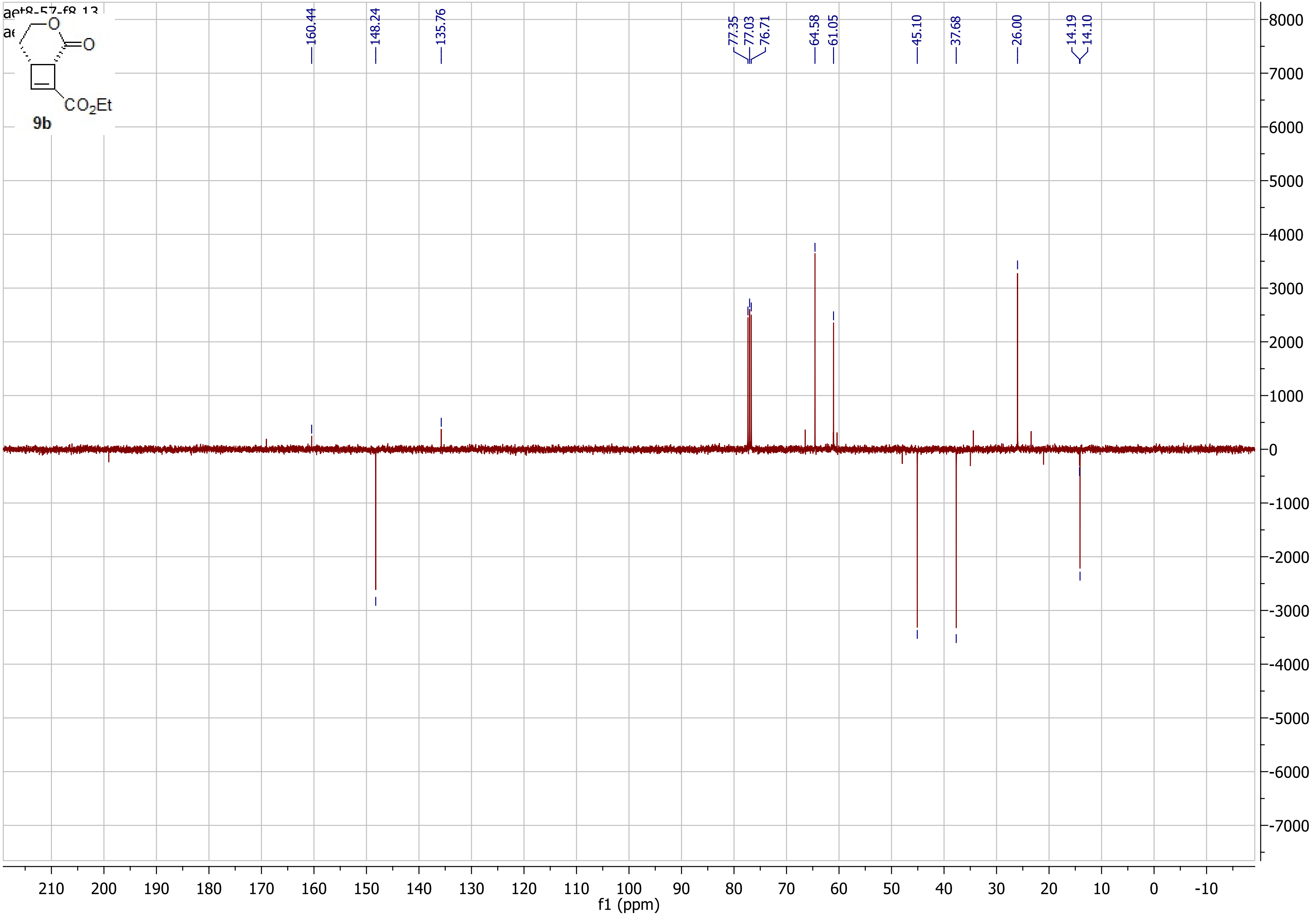

CO2Et
9b
160.44
148.24
135.76
77.35
77.03
76.71
64.58
61.05
45.10
37.68
26.00
14.19
14.10
8000
7000
6000
5000
4000
3000
2000
1000
0
-1000
-2000
-3000
-4000
-5000
-6000
-7000
210
200
190
180
170
160
150
140
130
120
110
100
90
80
70
60
50
40
30
20
10
0
-10
f1 (ppm)

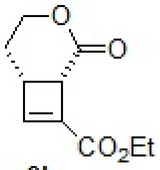

9b

COSY (400 MHz, CDCl3)

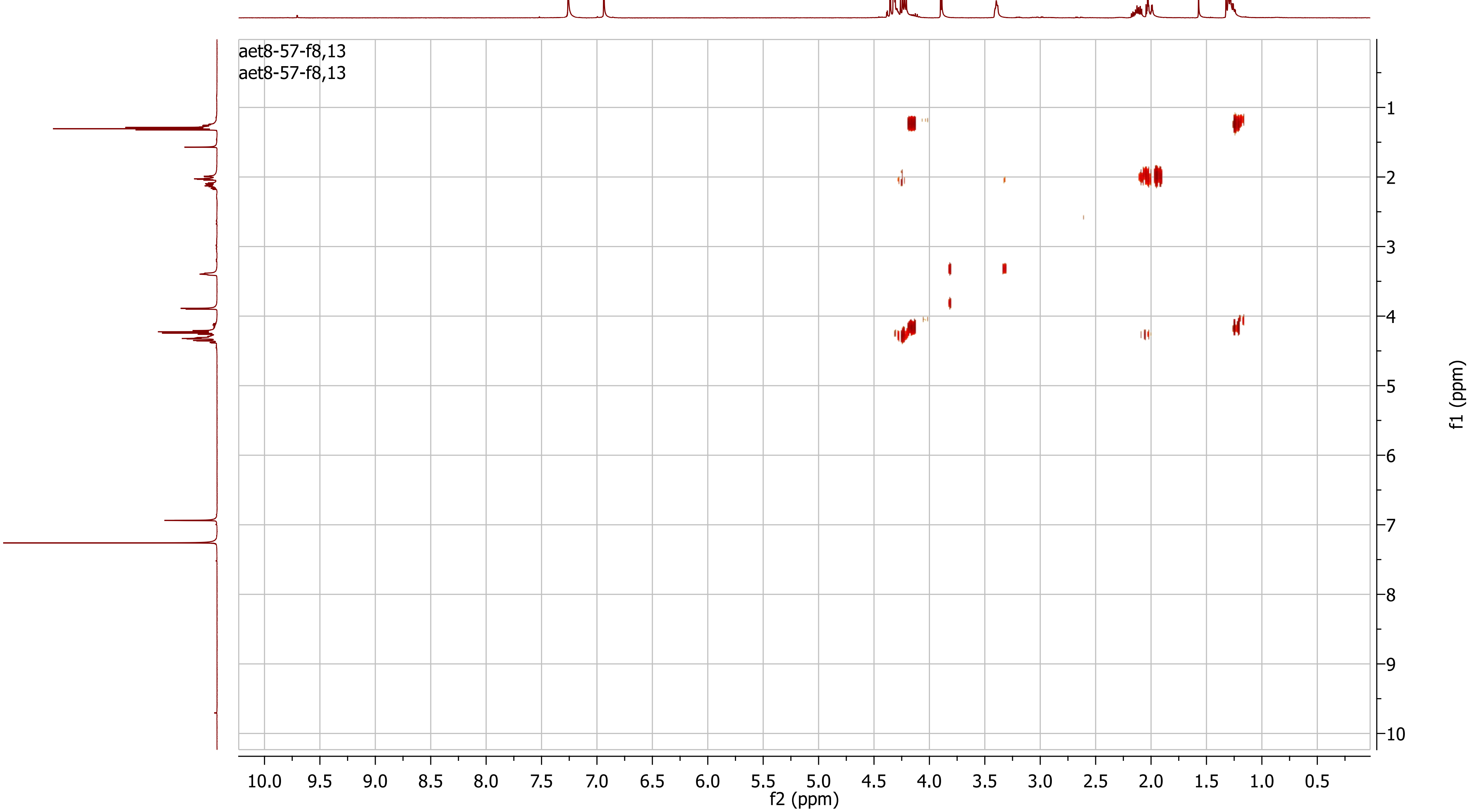

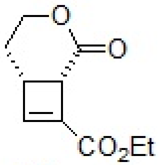

9b

COSY (400 MHz, CDCl3)

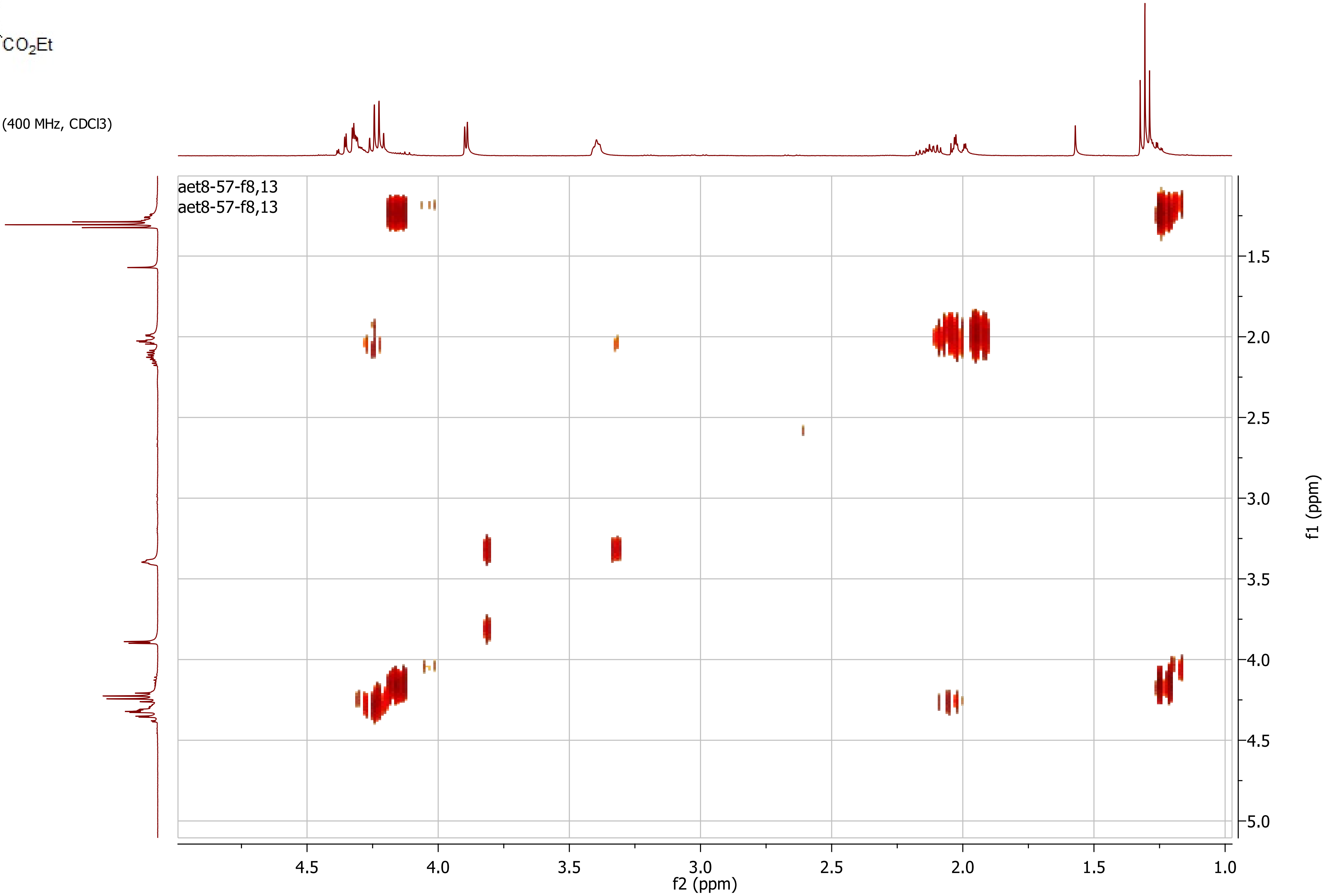

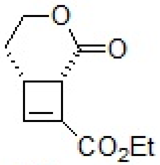

9b

HMQC (CDCl3)

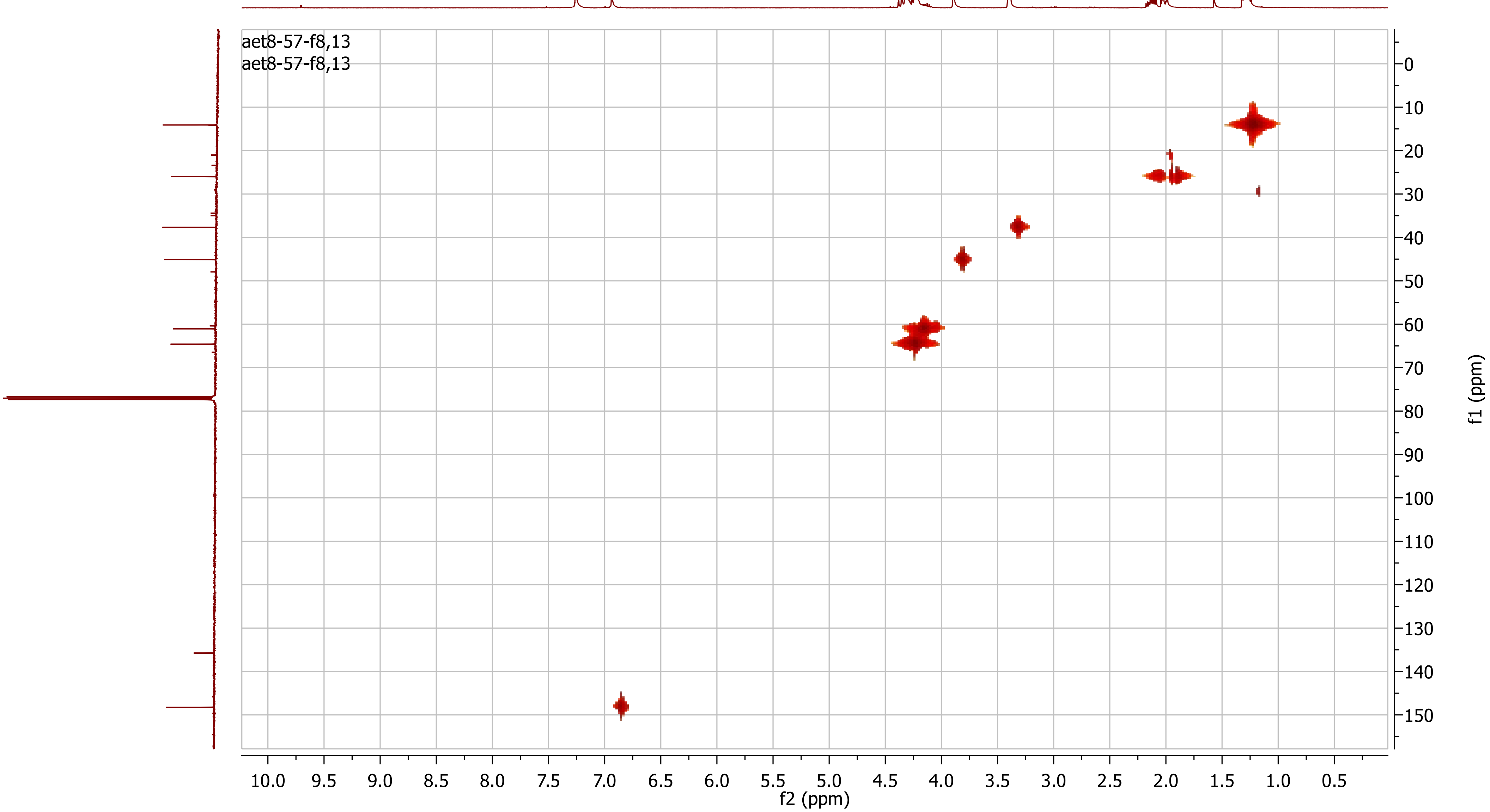

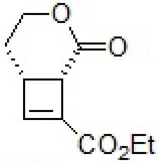

9b

NOESY (CDCl3)

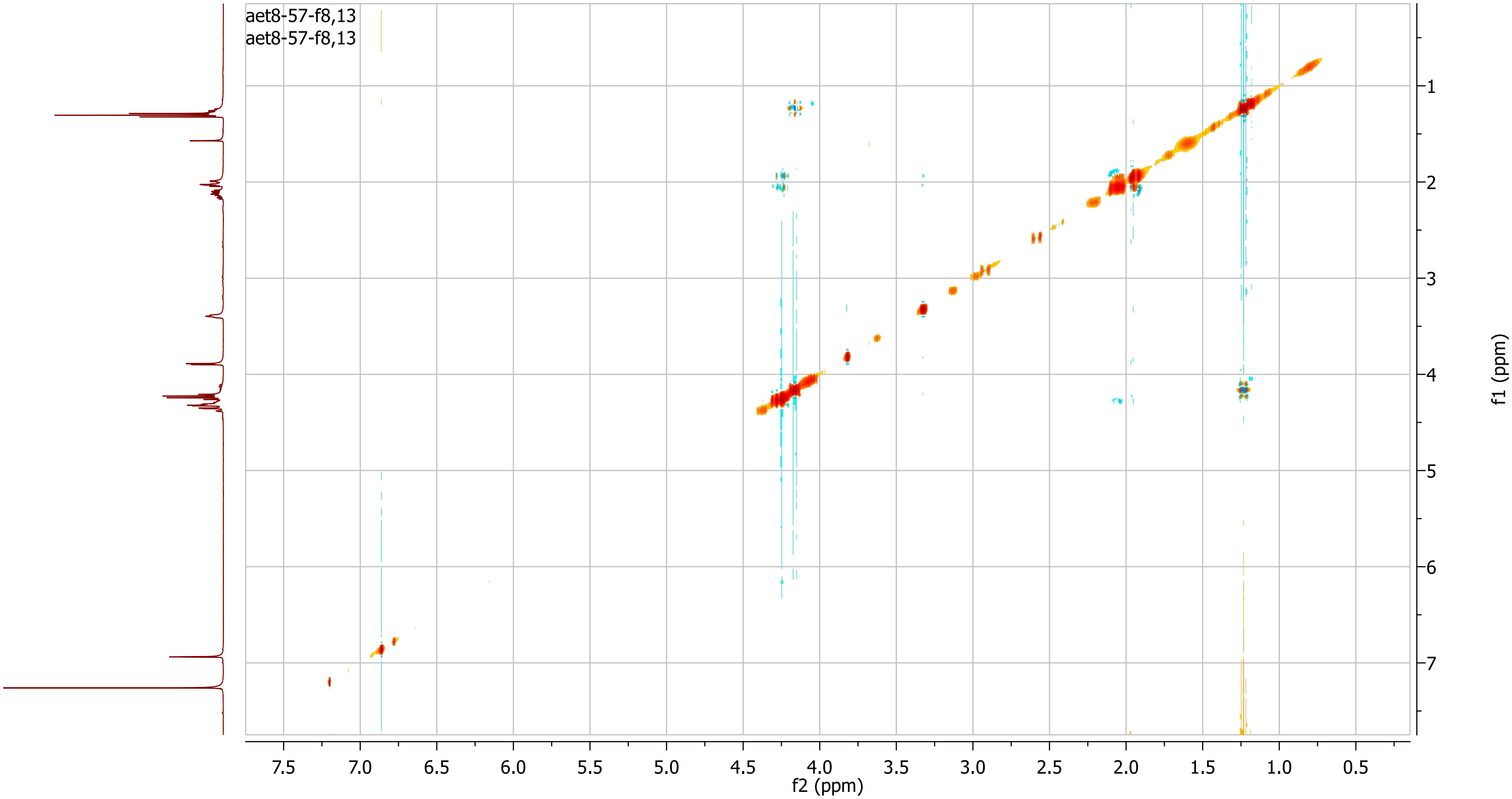

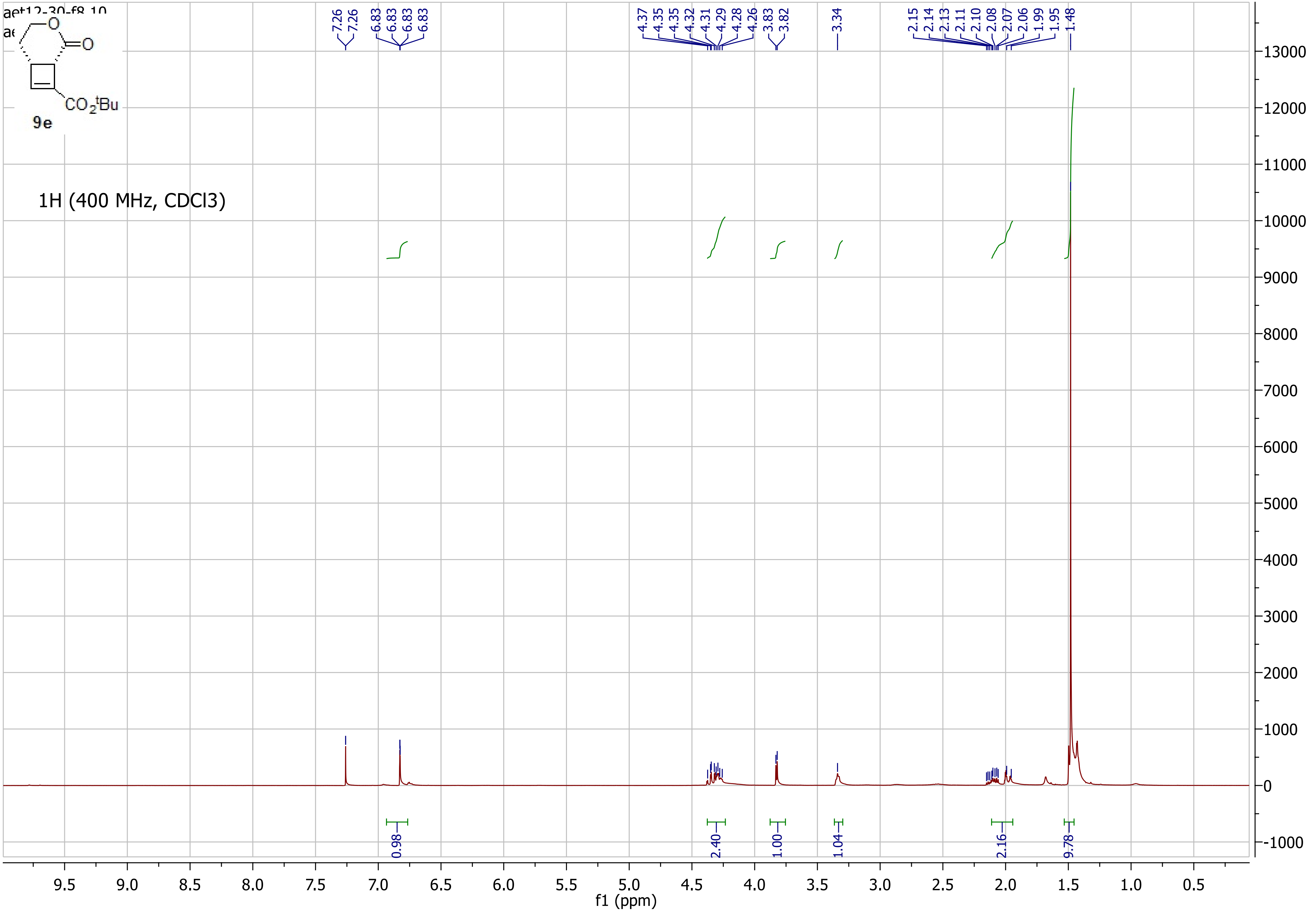

9e
1H (400 MHz, CDCl3)
7.26
6.83
4.37
4.35
4.32
4.31
4.29
4.28
4.26
3.83
3.82
3.34
2.15
2.14
2.13
2.11
2.10
2.08
2.07
2.06
1.99
1.95
1.48
0.98
2.40
1.00
1.04
2.16
9.78
f1 (ppm)

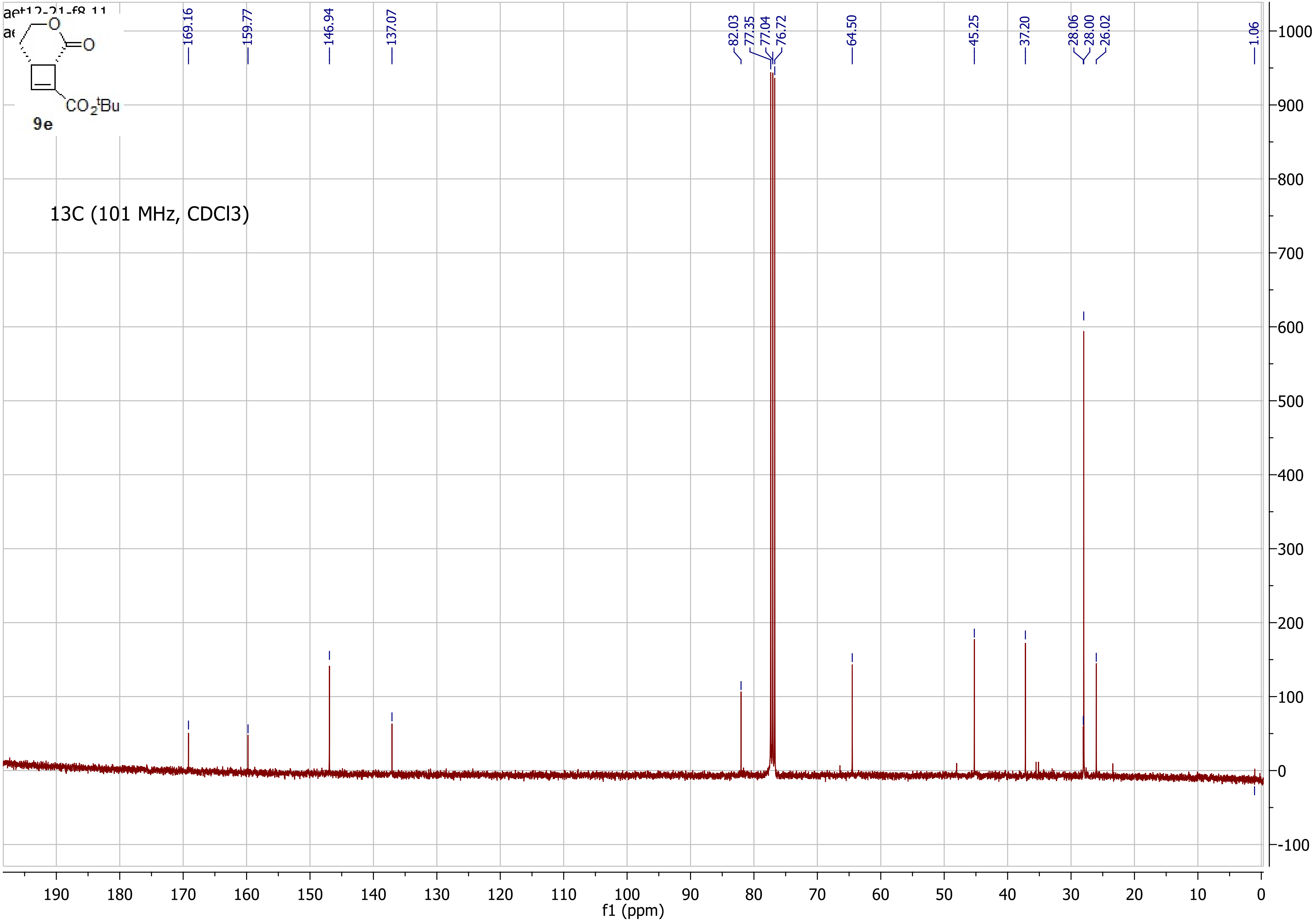

13C (101 MHz, CDCl3)
9e
O
O
CO2tBu
169.16
159.77
146.94
137.07
82.03
77.35
77.04
76.72
64.50
45.25
37.20
28.06
28.00
26.02
1.06
f1 (ppm)

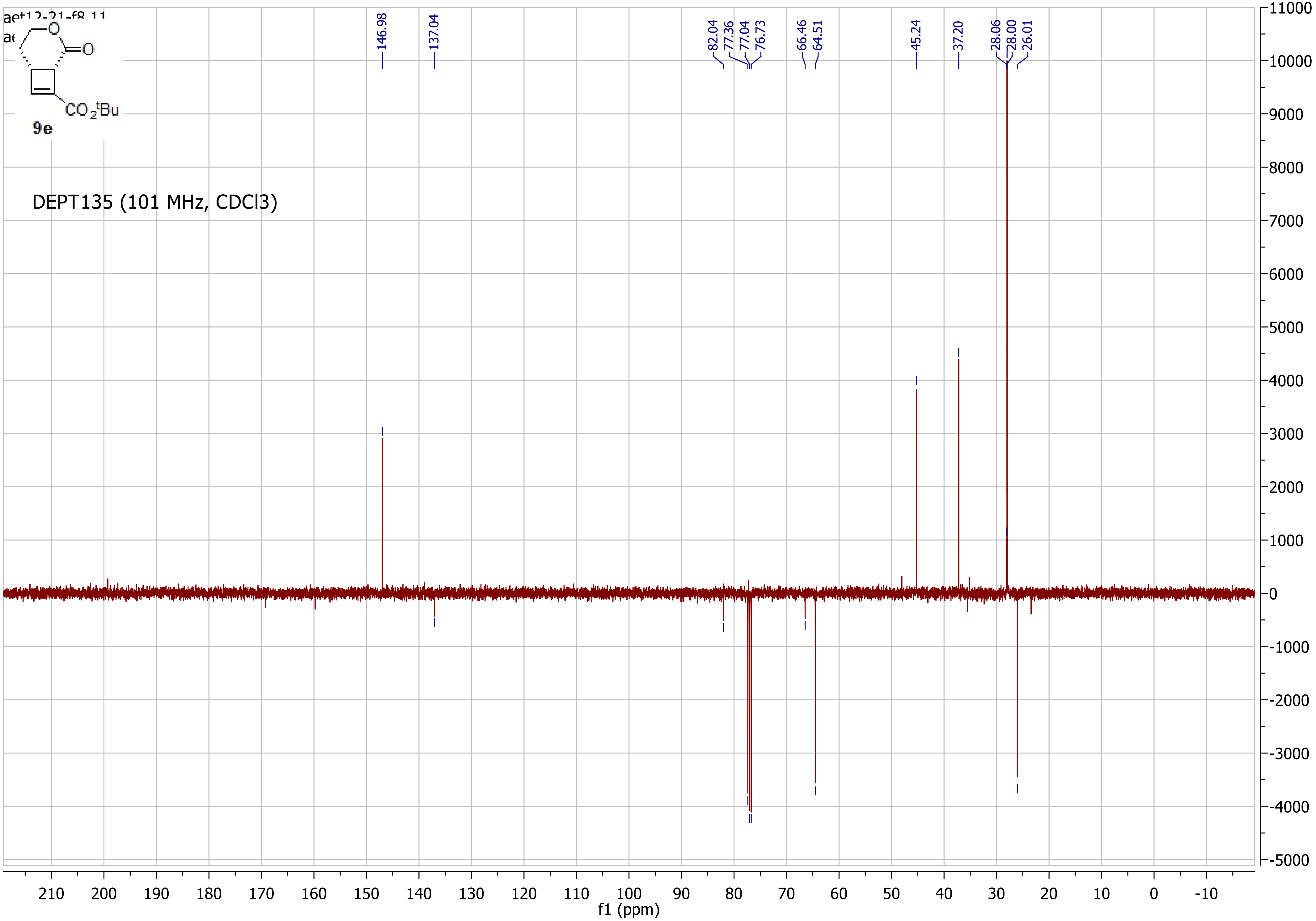
aet12-21-f8.11
O
O
CO2tBu
9e
DEPT135 (101 MHz, CDCl3)
146.98
137.04
82.04
77.36
77.04
76.73
66.46
64.51
45.24
37.20
28.06
28.00
26.01
f1 (ppm)

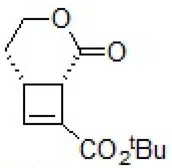

9e

COSY (400 MHz, CDCl3)

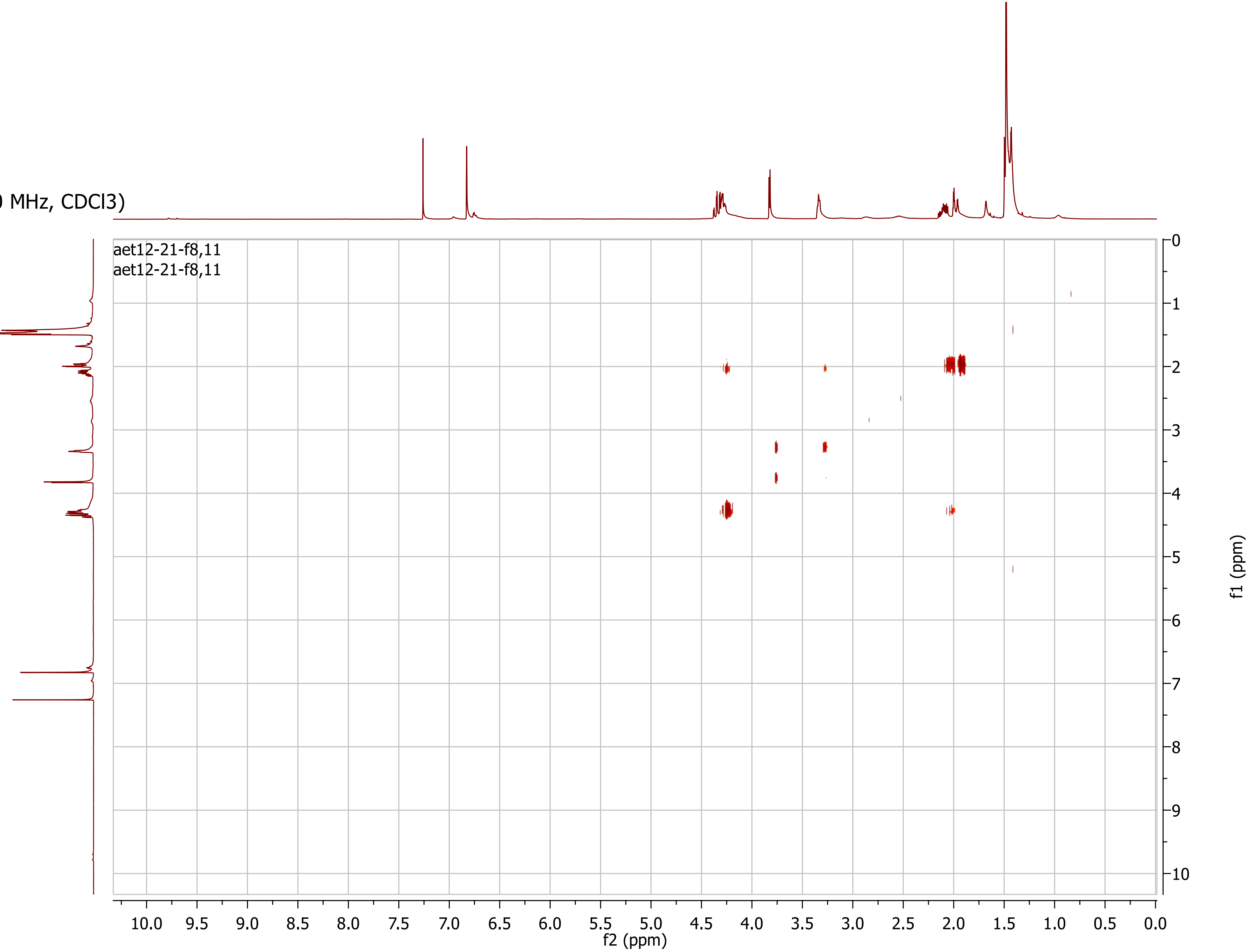

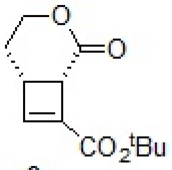

9e

COSY (400 MHz, CDCl3)

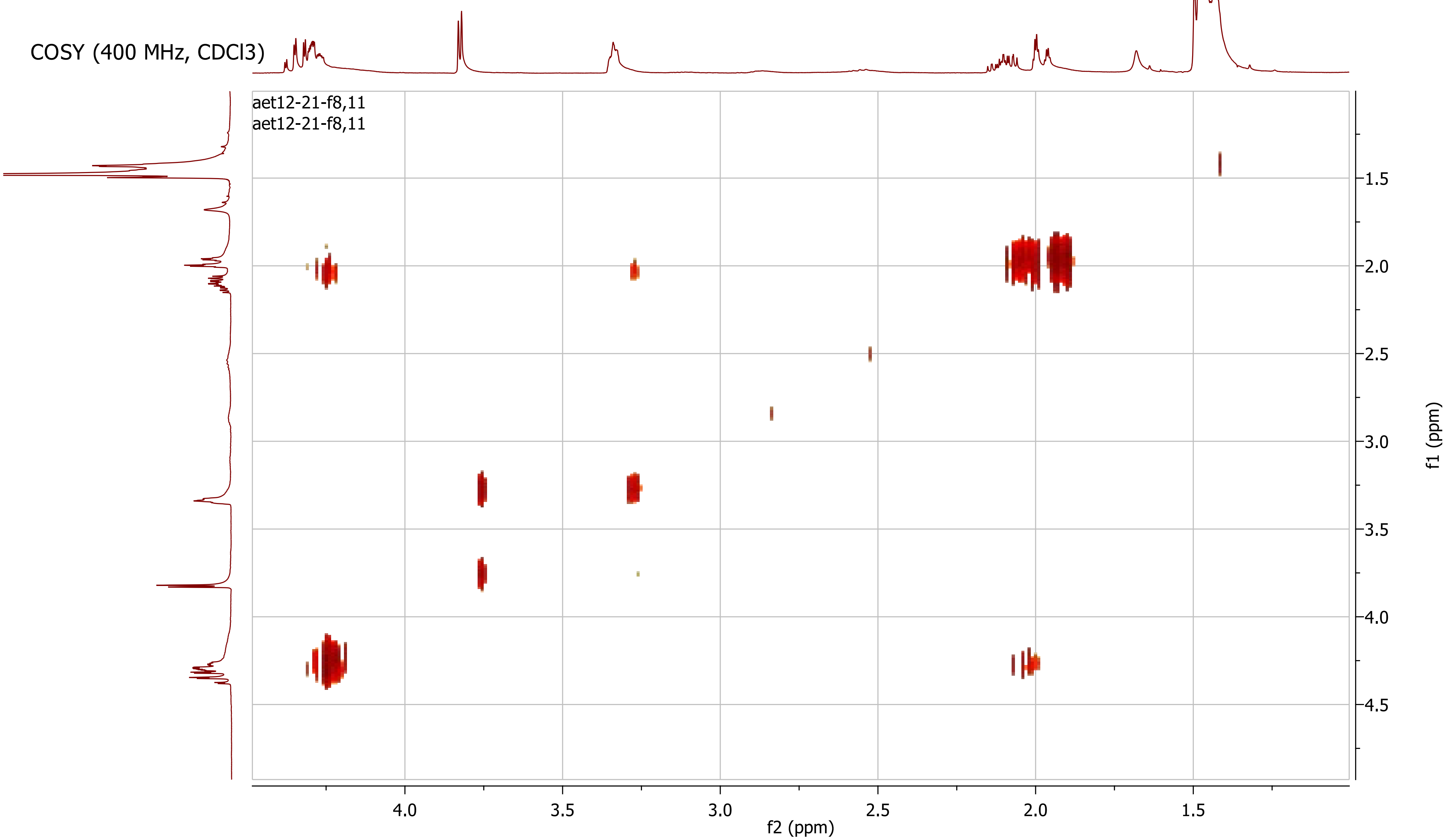

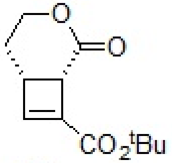

9e

HMQC (CDCl3)

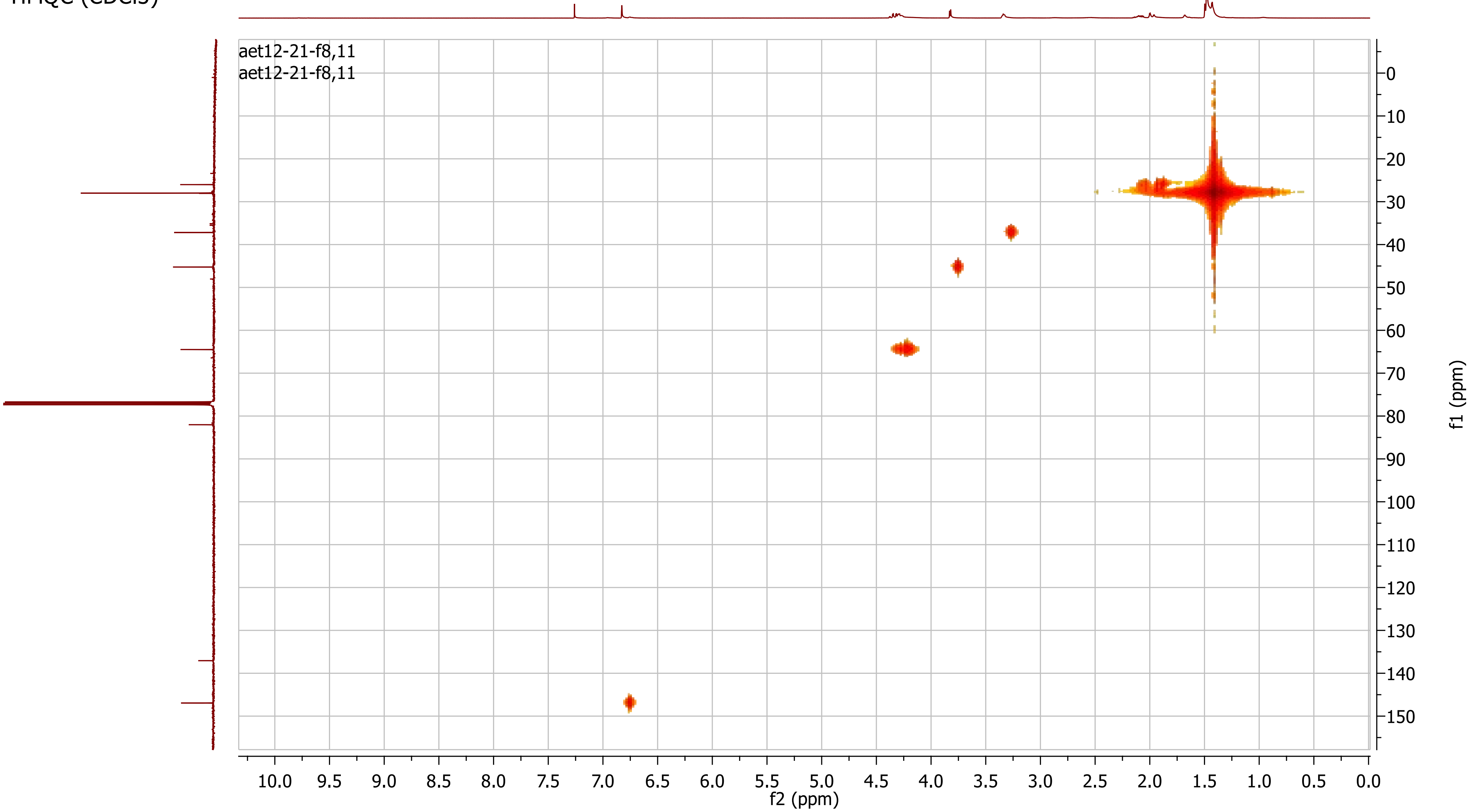

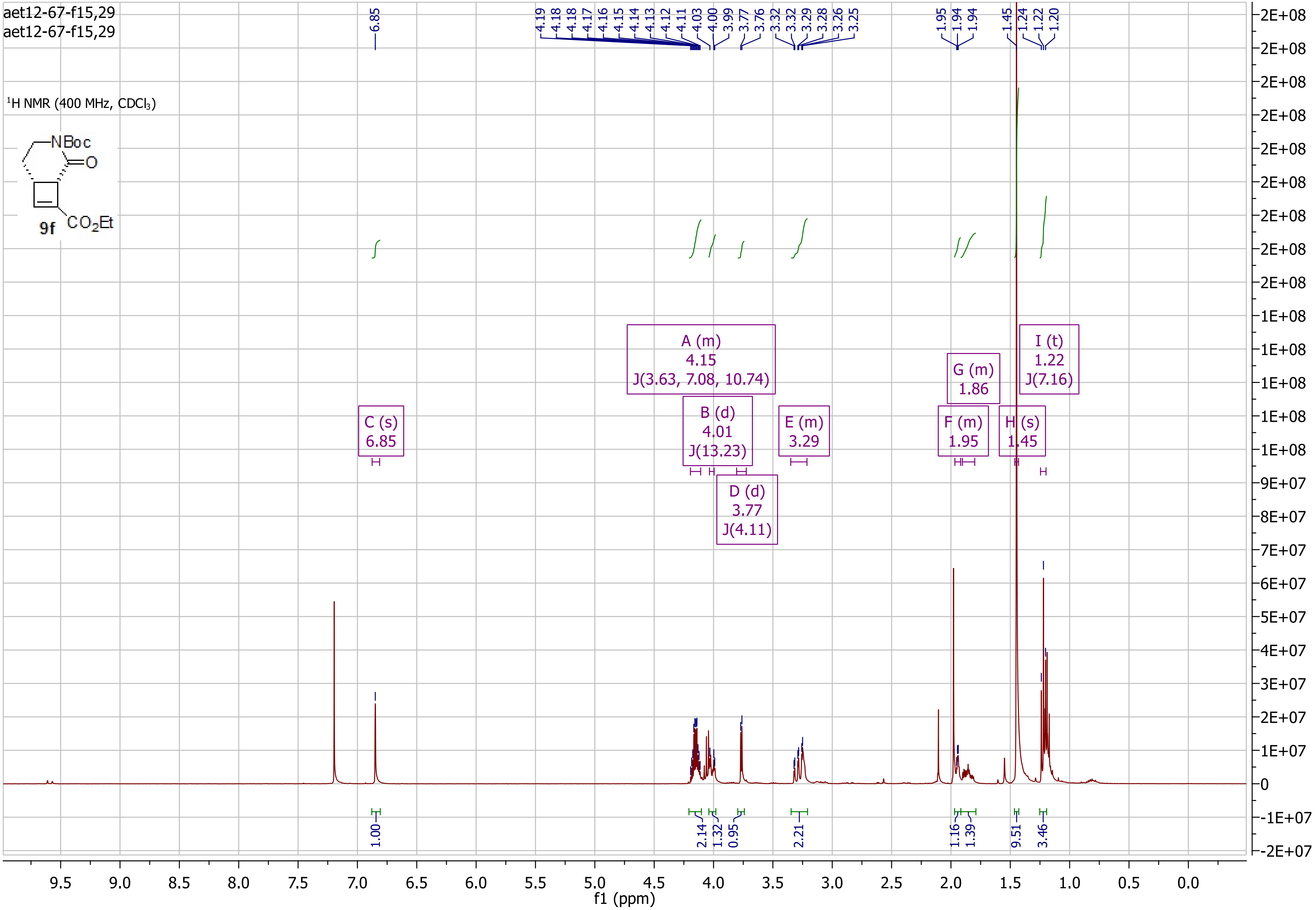

aet12-67-f15,29
aet12-67-f15,29
1H NMR (400 MHz, CDCl3)
NBoc
O
9f
CO2Et
6.85
4.19 4.18 4.18 4.17 4.16 4.15 4.14 4.13 4.12 4.11 4.03 4.00 3.99 3.77 3.76 3.32 3.32 3.29 3.28 3.26 3.25
1.95 1.94 1.94 1.45 1.24 1.22 1.20
A (m) 4.15 J(3.63, 7.08, 10.74)
B (d) 4.01 J(13.23)
C (s) 6.85
D (d) 3.77 J(4.11)
E (m) 3.29
F (m) 1.95
G (m) 1.86
H (s) 1.45
I (t) 1.22 J(7.16)
1.00 2.14 1.32 0.95 2.21 1.16 1.39 9.51 3.46
f1 (ppm)

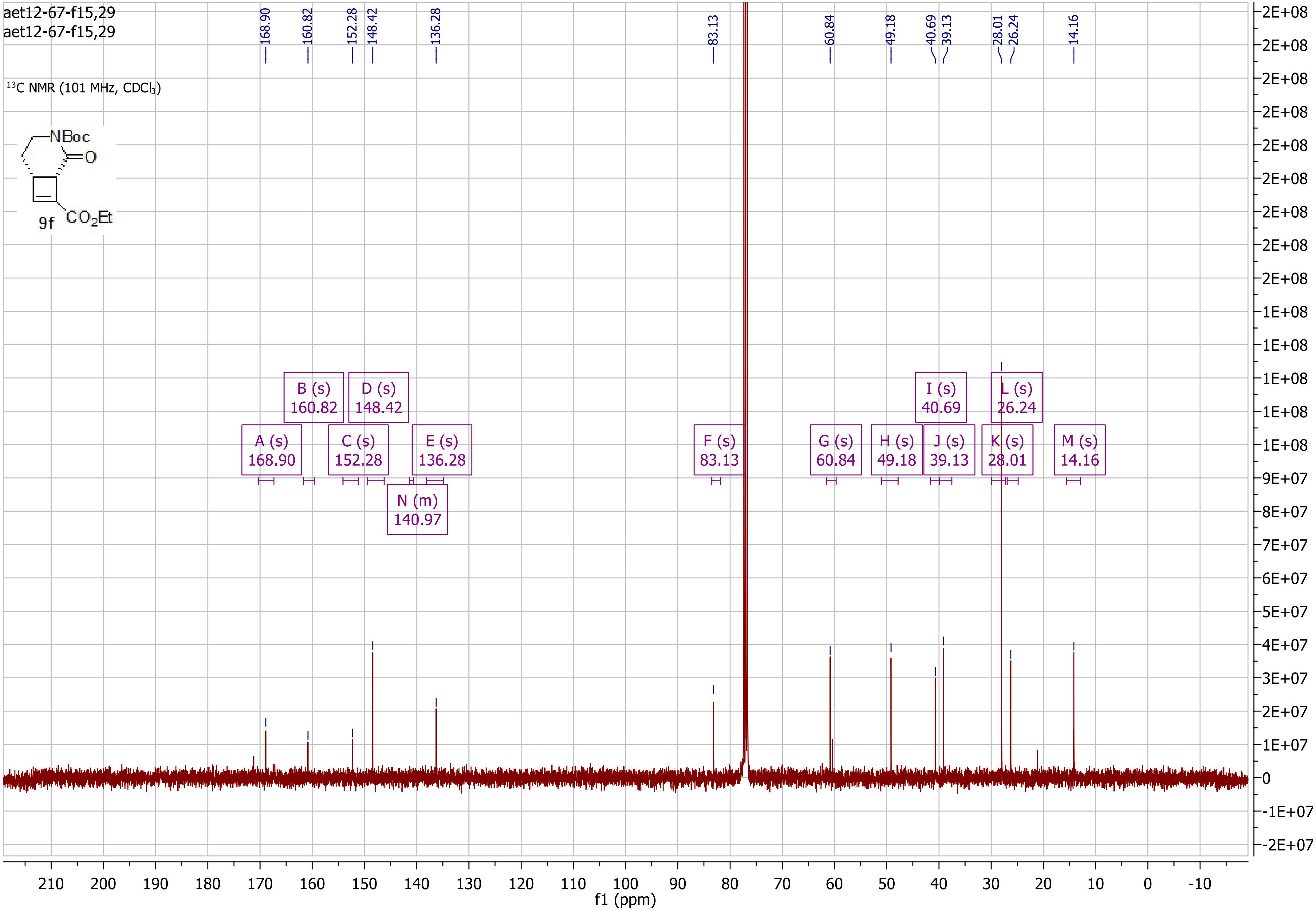
aet12-67-f15,29
aet12-67-f15,29
^{13}C NMR (101 MHz, $CDCl_3$)
NBoc
O
9f
CO_2Et
168.90
160.82
152.28
148.42
136.28
83.13
60.84
49.18
40.69
39.13
28.01
26.24
14.16
A (s) 168.90
B (s) 160.82
C (s) 152.28
D (s) 148.42
E (s) 136.28
N (m) 140.97
F (s) 83.13
G (s) 60.84
H (s) 49.18
I (s) 40.69
J (s) 39.13
K (s) 28.01
L (s) 26.24
M (s) 14.16
f1 (ppm)

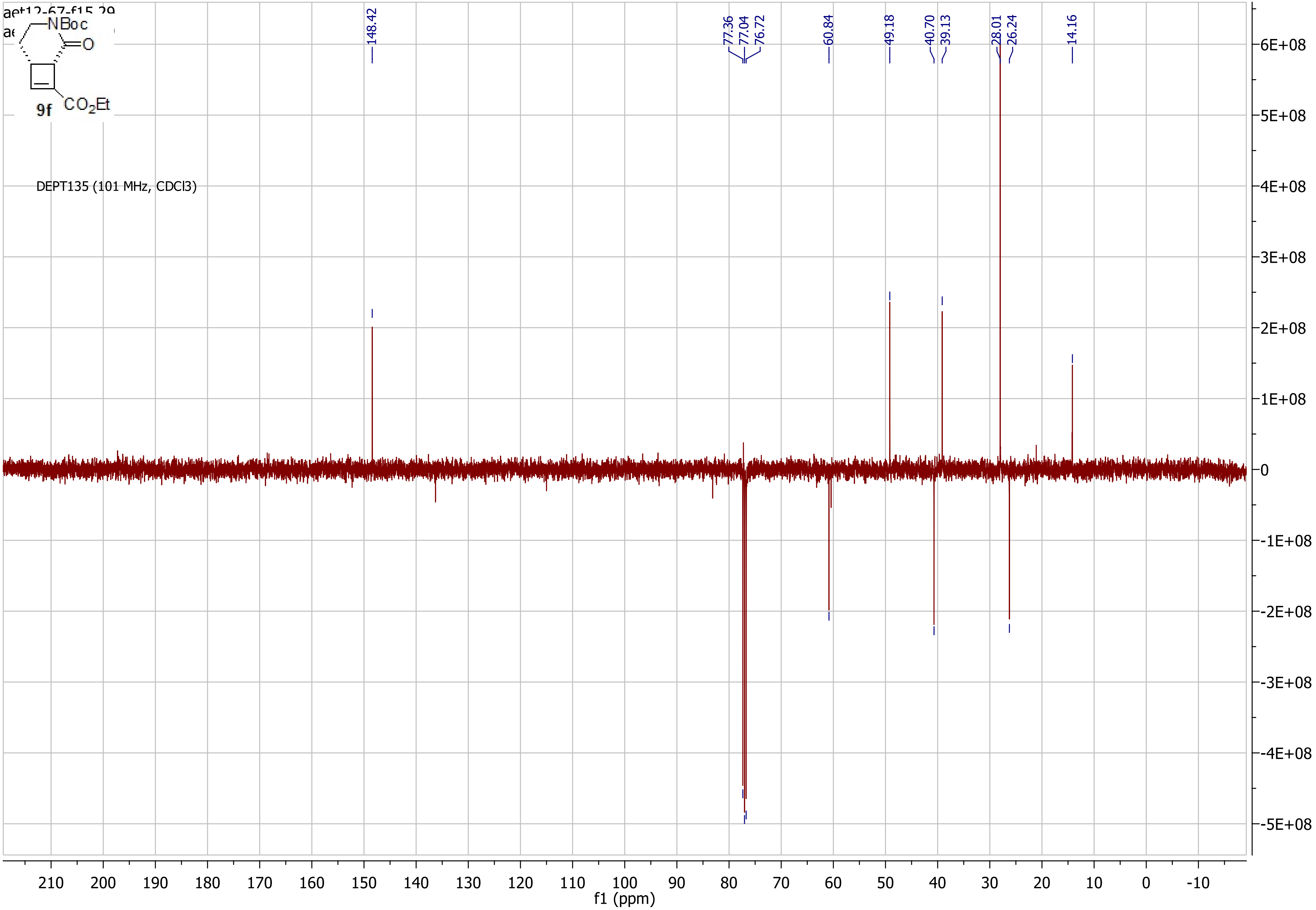

aet12-67-f15.29
NBoc
O
9f
CO_2Et
DEPT135 (101 MHz, CDCl3)
148.42
77.36
77.04
76.72
60.84
49.18
40.70
39.13
28.01
26.24
14.16
6E+08
5E+08
4E+08
3E+08
2E+08
1E+08
0
-1E+08
-2E+08
-3E+08
-4E+08
-5E+08
210 200 190 180 170 160 150 140 130 120 110 100 90 80 70 60 50 40 30 20 10 0 -10
f1 (ppm)

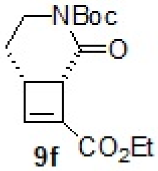

9f

COSY (400 MHz, CDCl3)

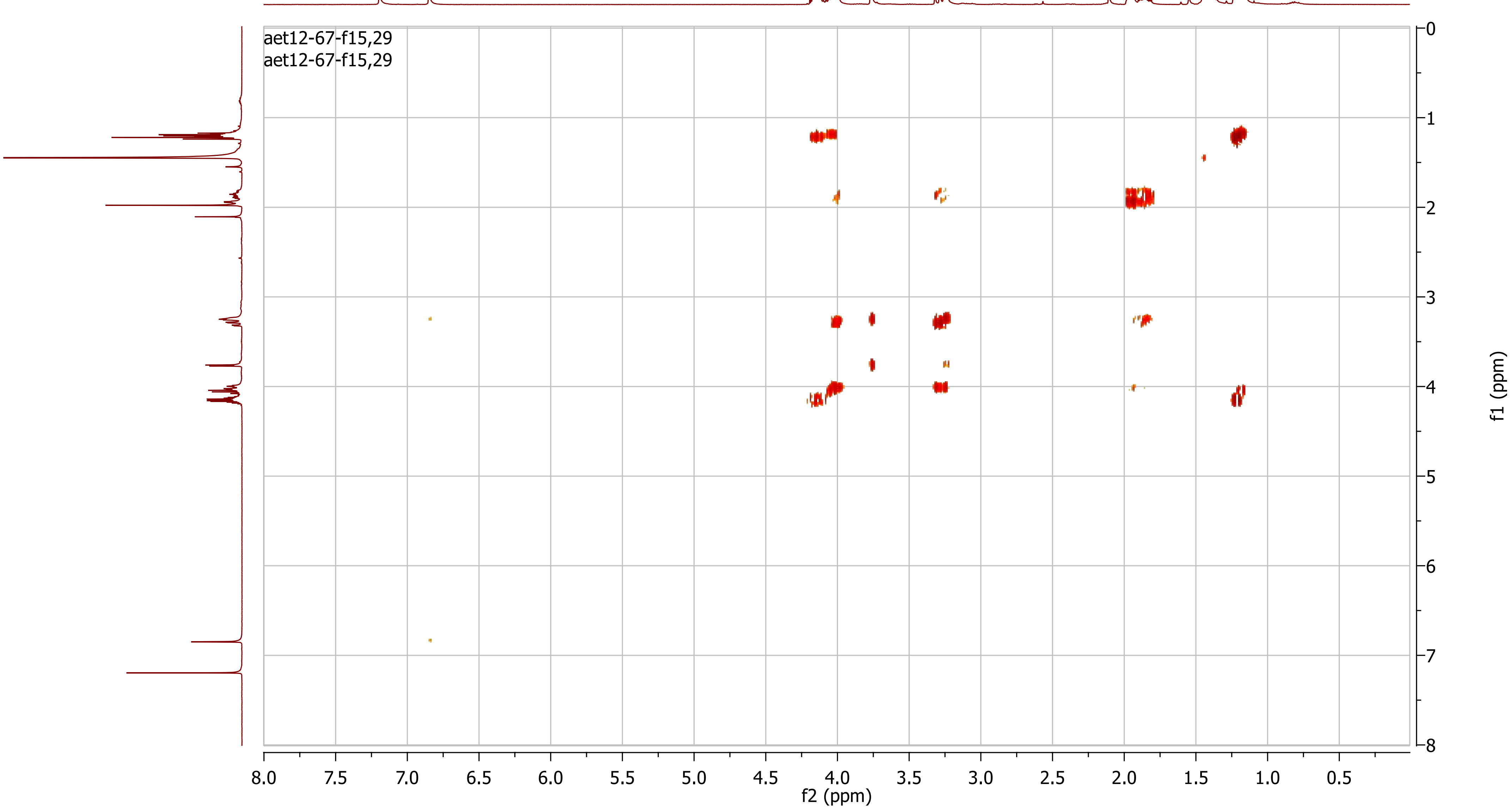

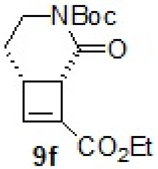

9f

COSY (400 MHz, CDCl3)

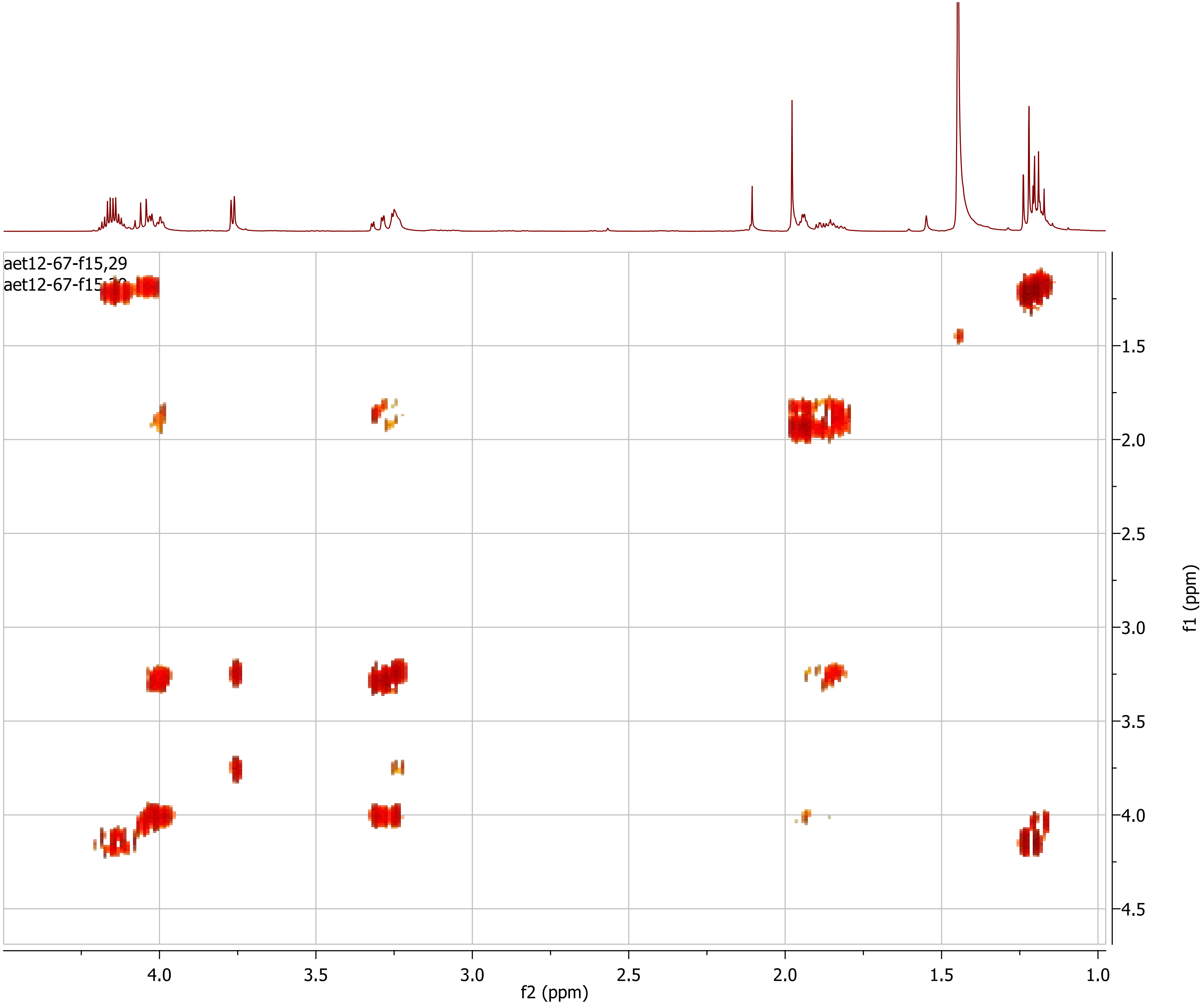

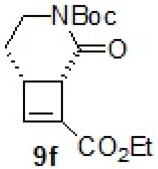

9f

HMQC (CDCl3)

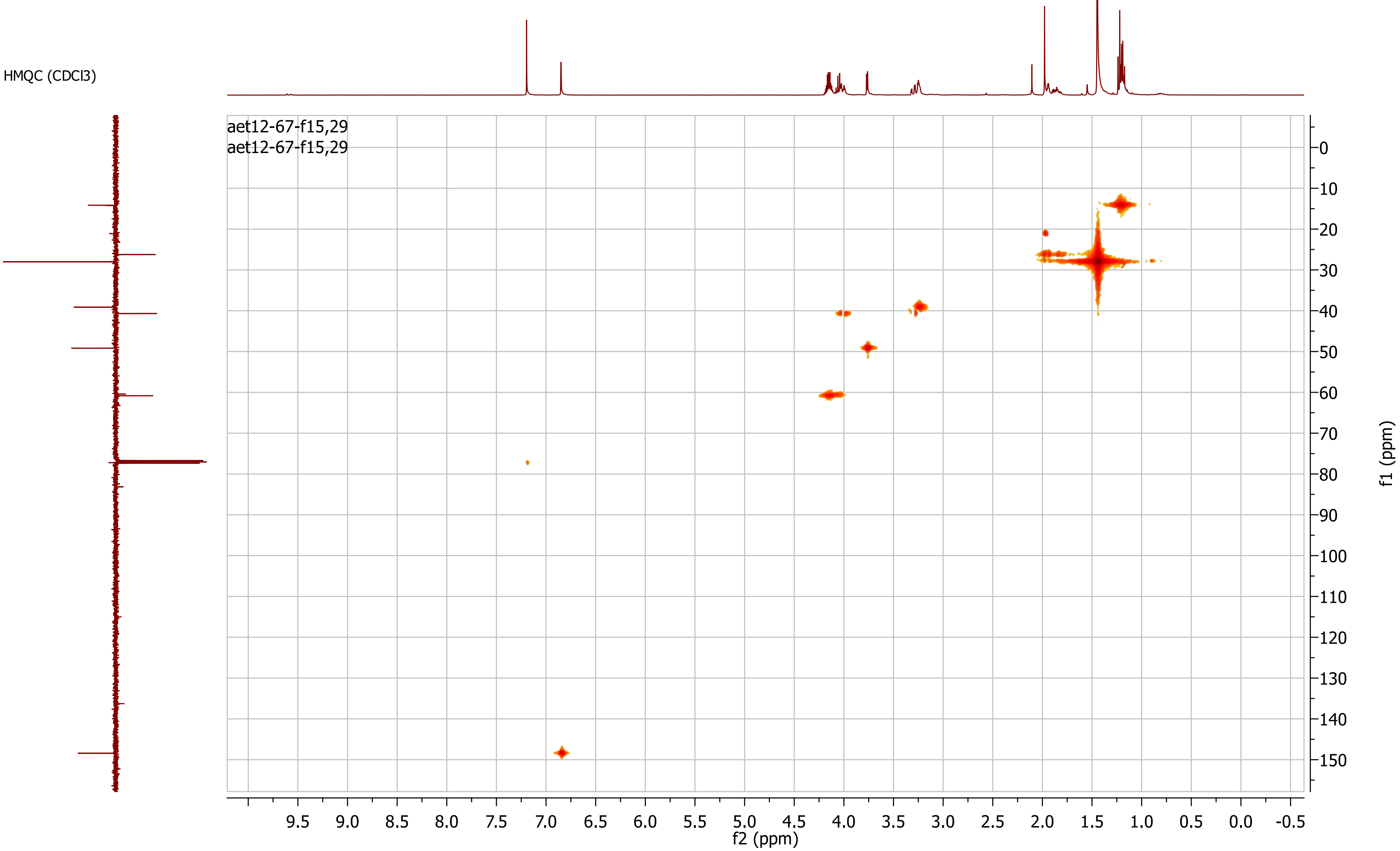

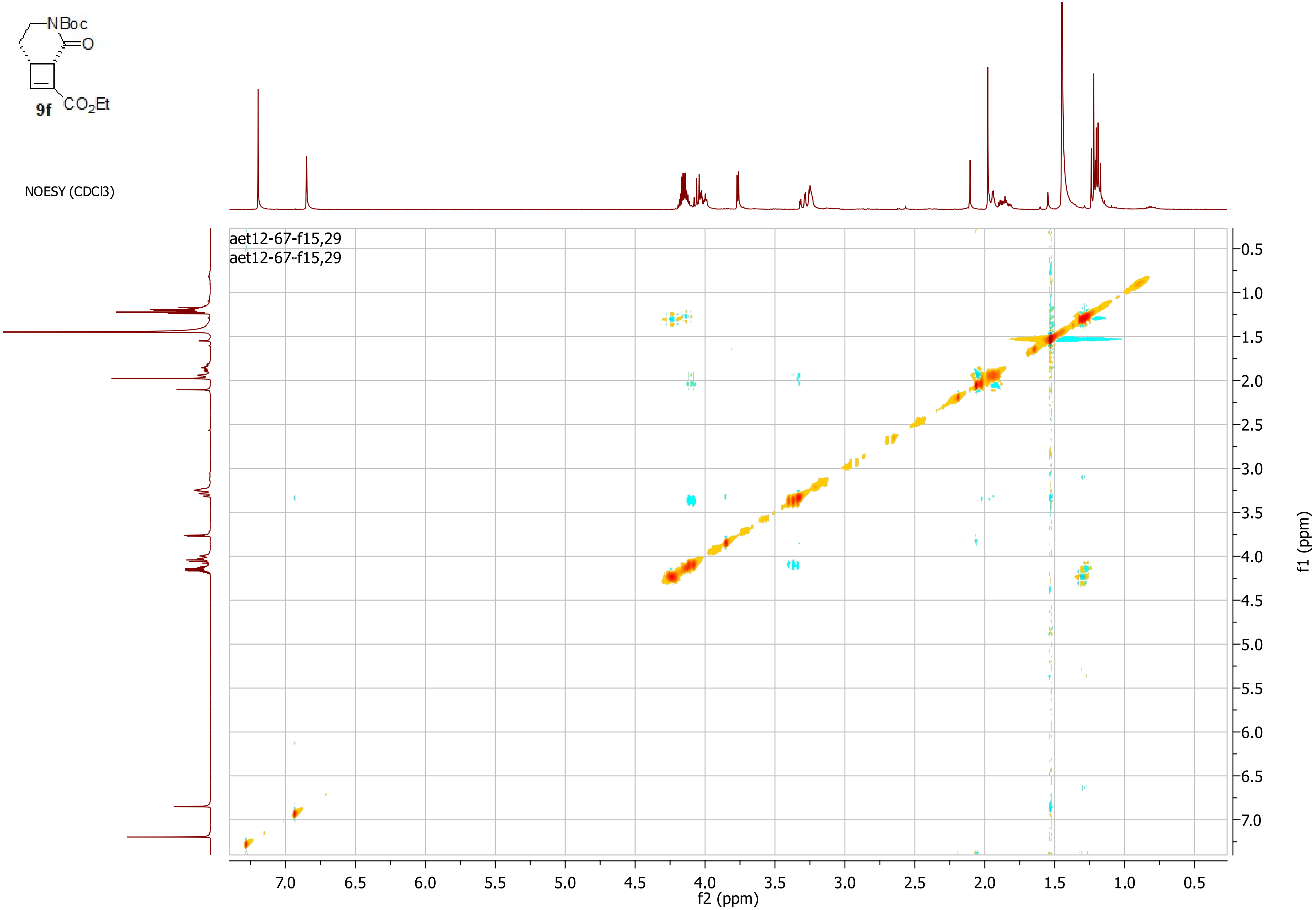
NBoc
O
9f
CO_2Et
NOESY (CDCl3)
aet12-67-f15,29
aet12-67-f15,29
f2 (ppm)
f1 (ppm)

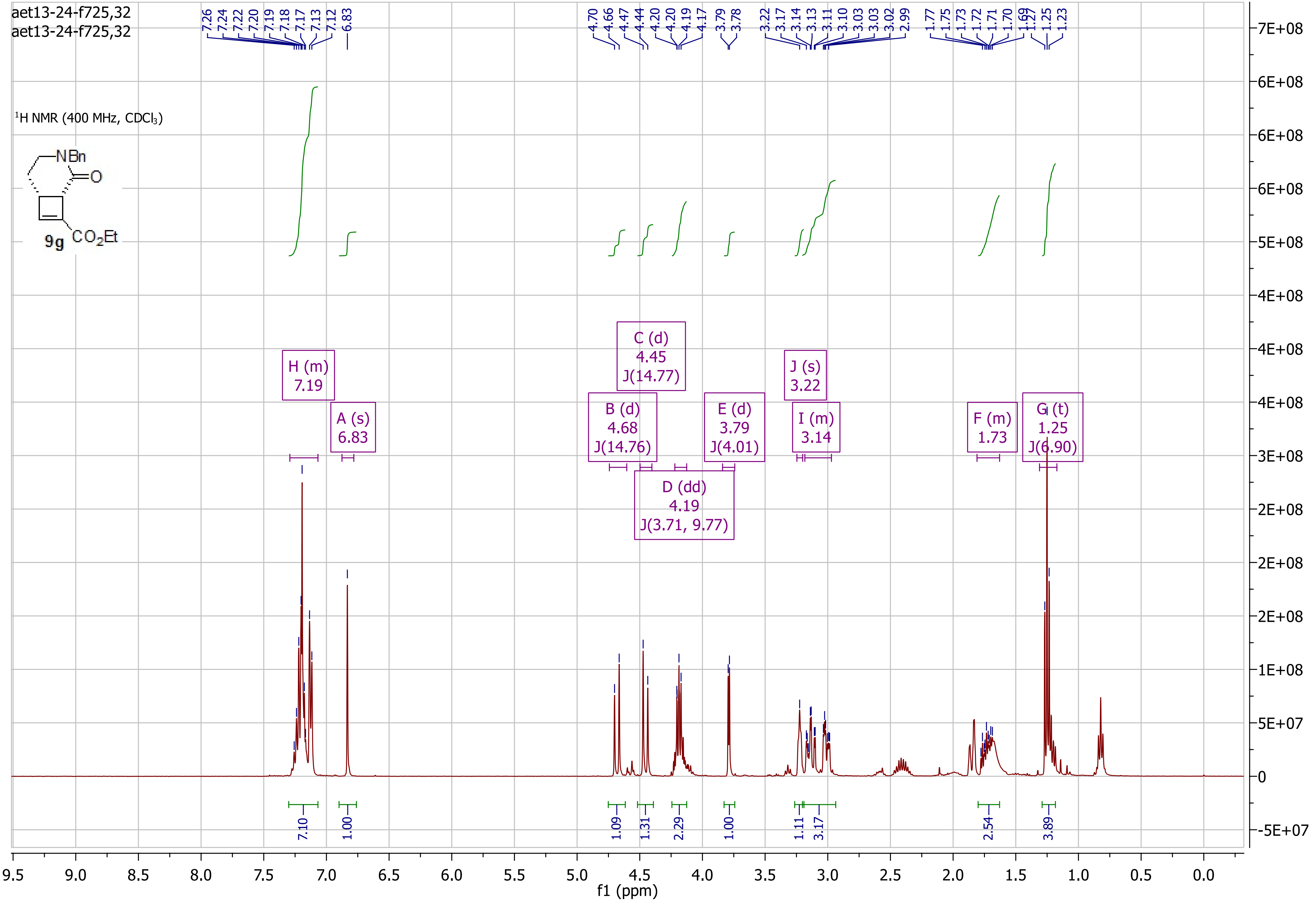
aet13-24-f725,32
aet13-24-f725,32
1H NMR (400 MHz, CDCl3)
NBn
O
CO2Et
9g
7.26 7.24 7.22 7.20 7.19 7.18 7.17 7.13 7.12 6.83
4.70 4.66 4.47 4.44 4.20 4.20 4.19 4.17 3.79 3.78
3.22 3.17 3.14 3.13 3.11 3.10 3.03 3.03 3.02 2.99
1.77 1.75 1.73 1.72 1.71 1.70 1.69 1.27 1.25 1.23
H (m) 7.19
A (s) 6.83
C (d) 4.45 J(14.77)
B (d) 4.68 J(14.76)
D (dd) 4.19 J(3.71, 9.77)
E (d) 3.79 J(4.01)
J (s) 3.22
I (m) 3.14
F (m) 1.73
G (t) 1.25 J(6.90)
7.10 1.00 1.09 1.31 2.29 1.00 1.11 3.17 2.54 3.89
f1 (ppm)

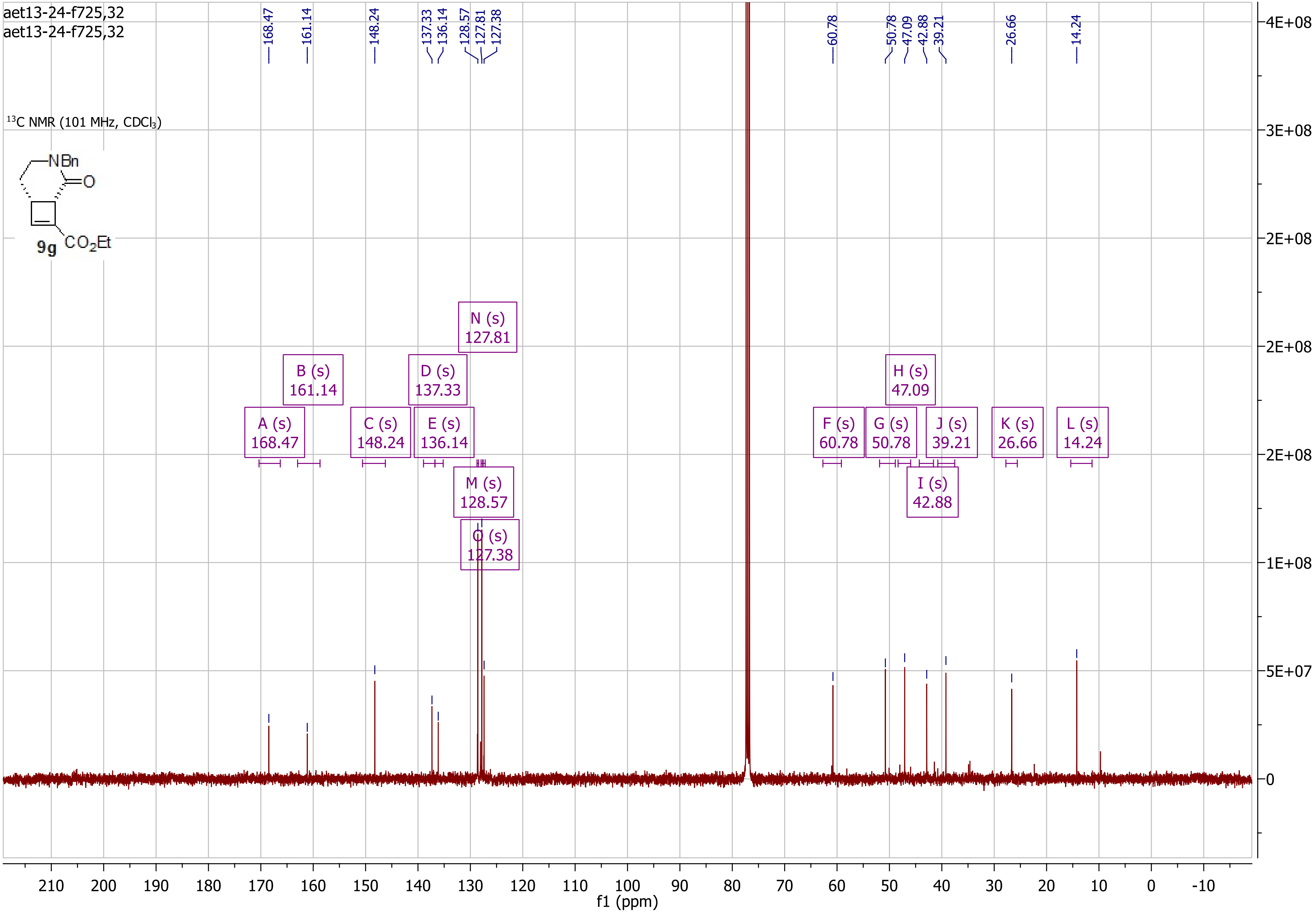
aet13-24-f725,32
aet13-24-f725,32
^{13}C NMR (101 MHz, CDCl$_3$)
NBn
O
9g
CO$_2$Et
168.47
161.14
148.24
137.33
136.14
128.57
127.81
127.38
60.78
50.78
47.09
42.88
39.21
26.66
14.24
A (s) 168.47
B (s) 161.14
C (s) 148.24
D (s) 137.33
E (s) 136.14
F (s) 60.78
G (s) 50.78
H (s) 47.09
I (s) 42.88
J (s) 39.21
K (s) 26.66
L (s) 14.24
M (s) 128.57
N (s) 127.81
O (s) 127.38
4E+08
3E+08
2E+08
2E+08
2E+08
1E+08
5E+07
0
210 200 190 180 170 160 150 140 130 120 110 100 90 80 70 60 50 40 30 20 10 0 -10
f1 (ppm)

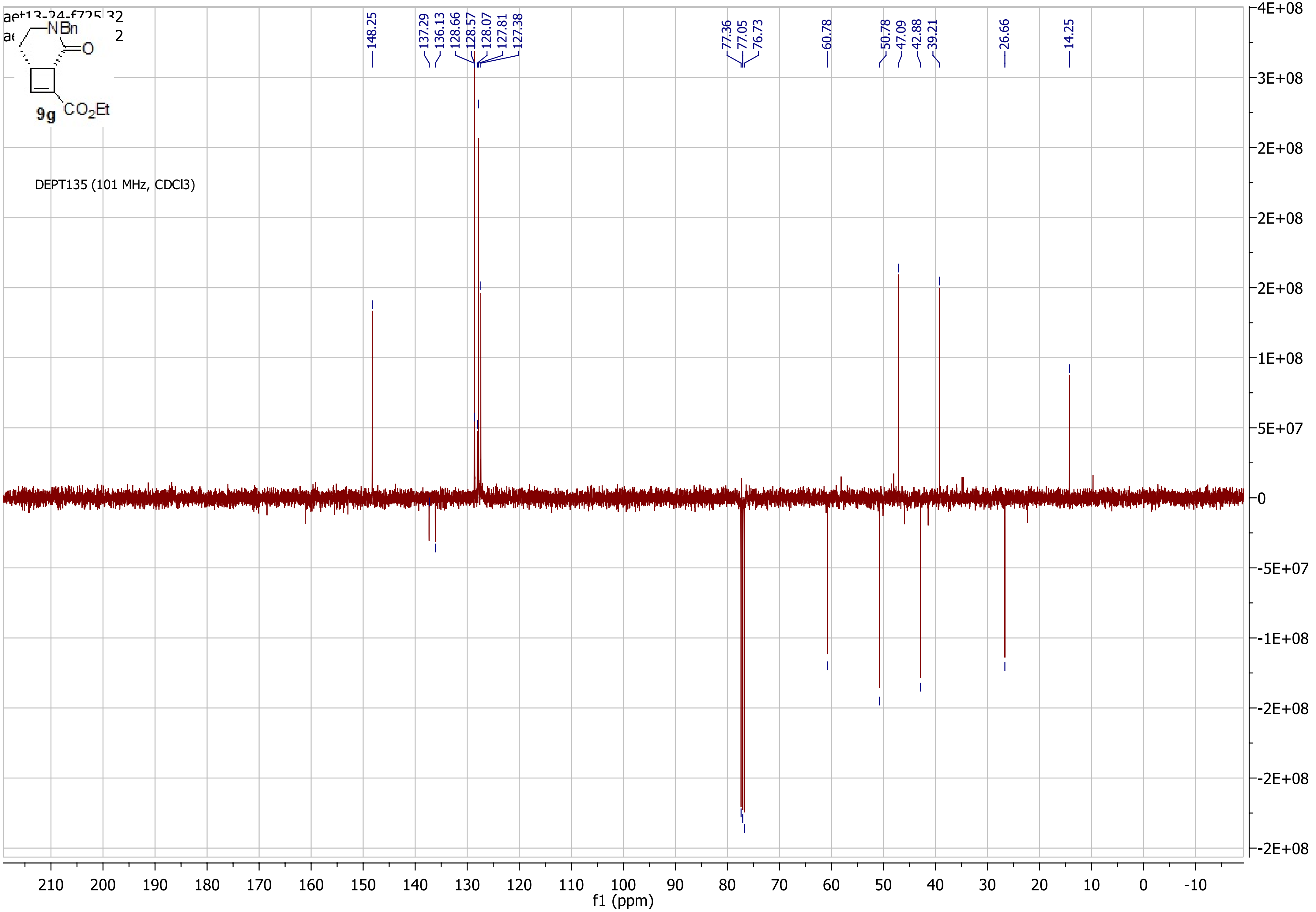

NBn
O
9g
CO2Et
DEPT135 (101 MHz, CDCl3)
148.25
137.29
136.13
128.66
128.57
128.07
127.81
127.38
77.36
77.05
76.73
60.78
50.78
47.09
42.88
39.21
26.66
14.25
f1 (ppm)

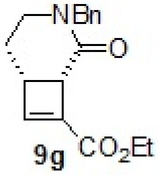

9g

COSY (400 MHz, CDCl3)

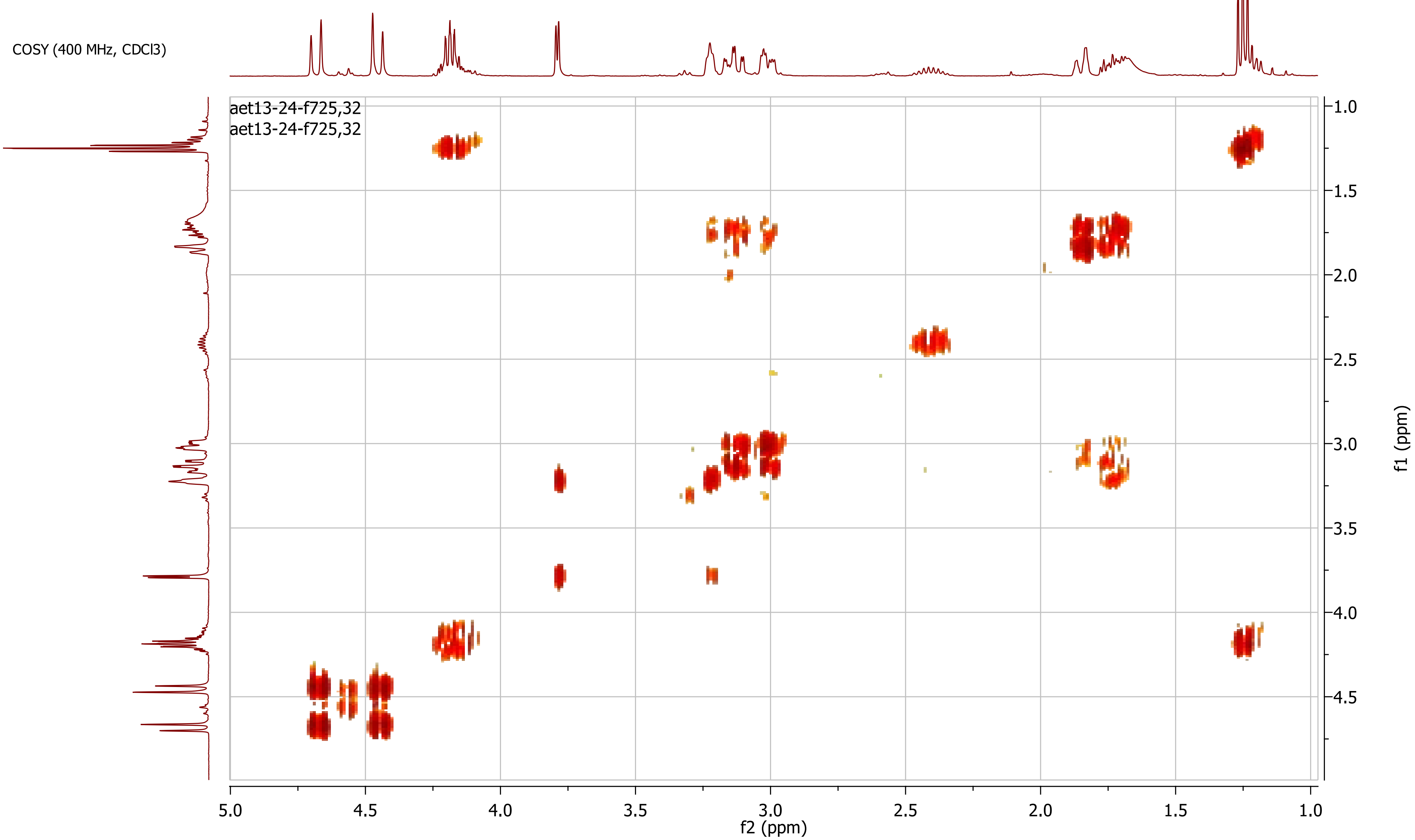

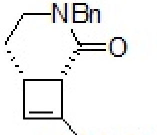

9g

COSY (400 MHz, CDCl3)

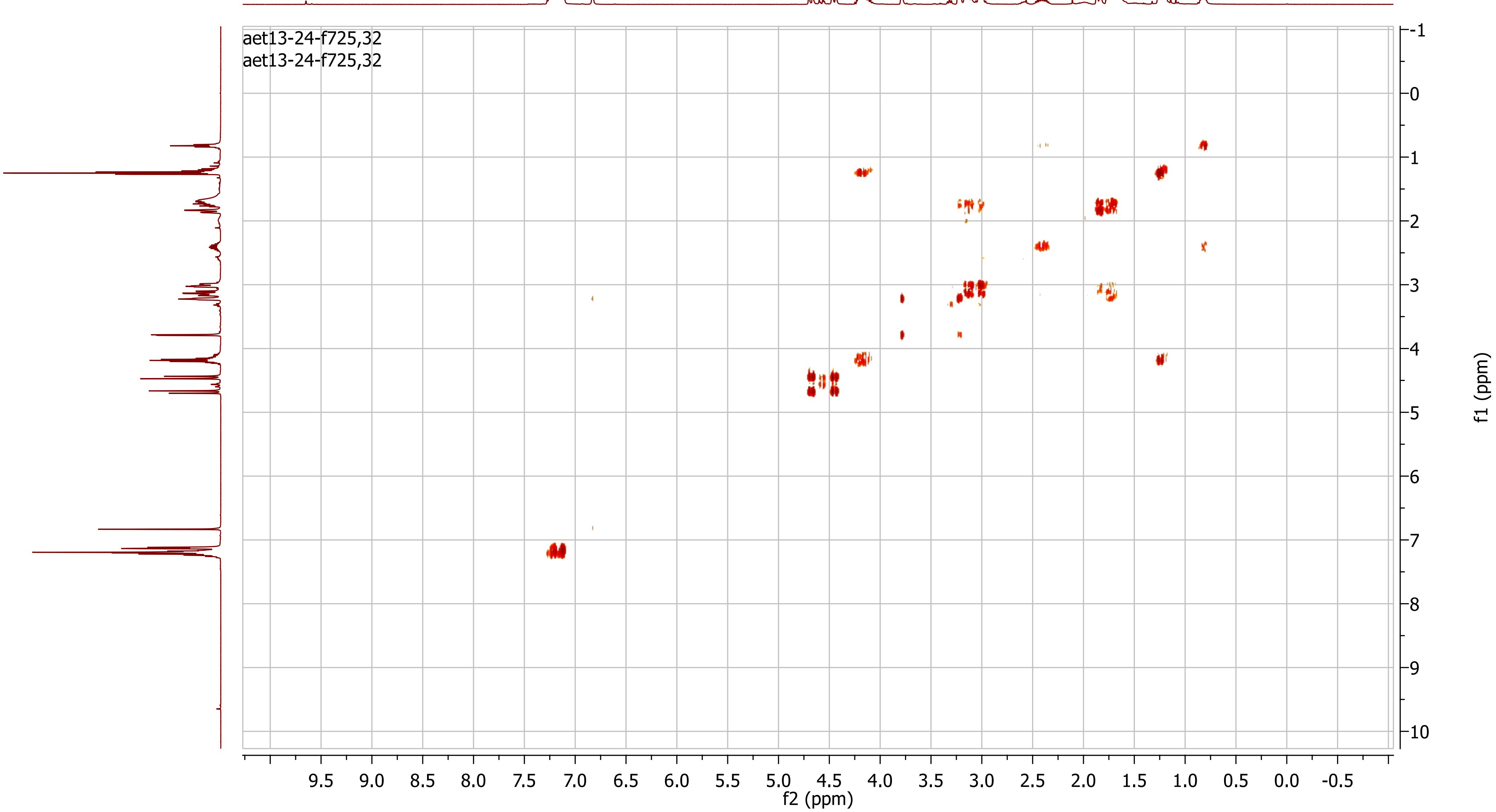

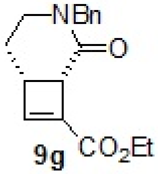
NBn
O
CO2Et

9g

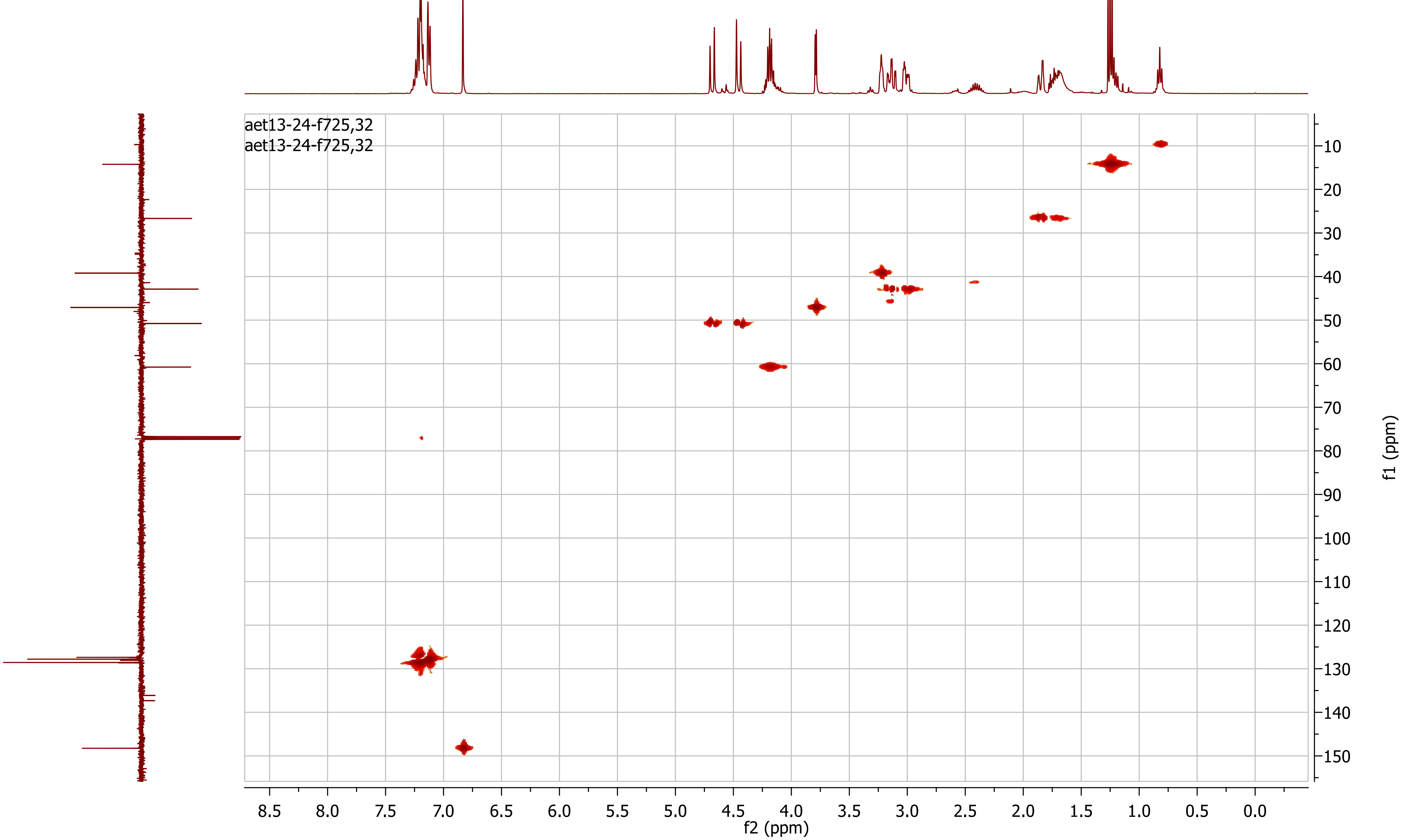
aet13-24-f725,32
aet13-24-f725,32
f2 (ppm)
f1 (ppm)
8.5 8.0 7.5 7.0 6.5 6.0 5.5 5.0 4.5 4.0 3.5 3.0 2.5 2.0 1.5 1.0 0.5 0.0
10 20 30 40 50 60 70 80 90 100 110 120 130 140 150

aet12-79-f8,15a
aet12-79-f8,15a

^{1}H NMR (400 MHz, $CDCl_3$)

10c

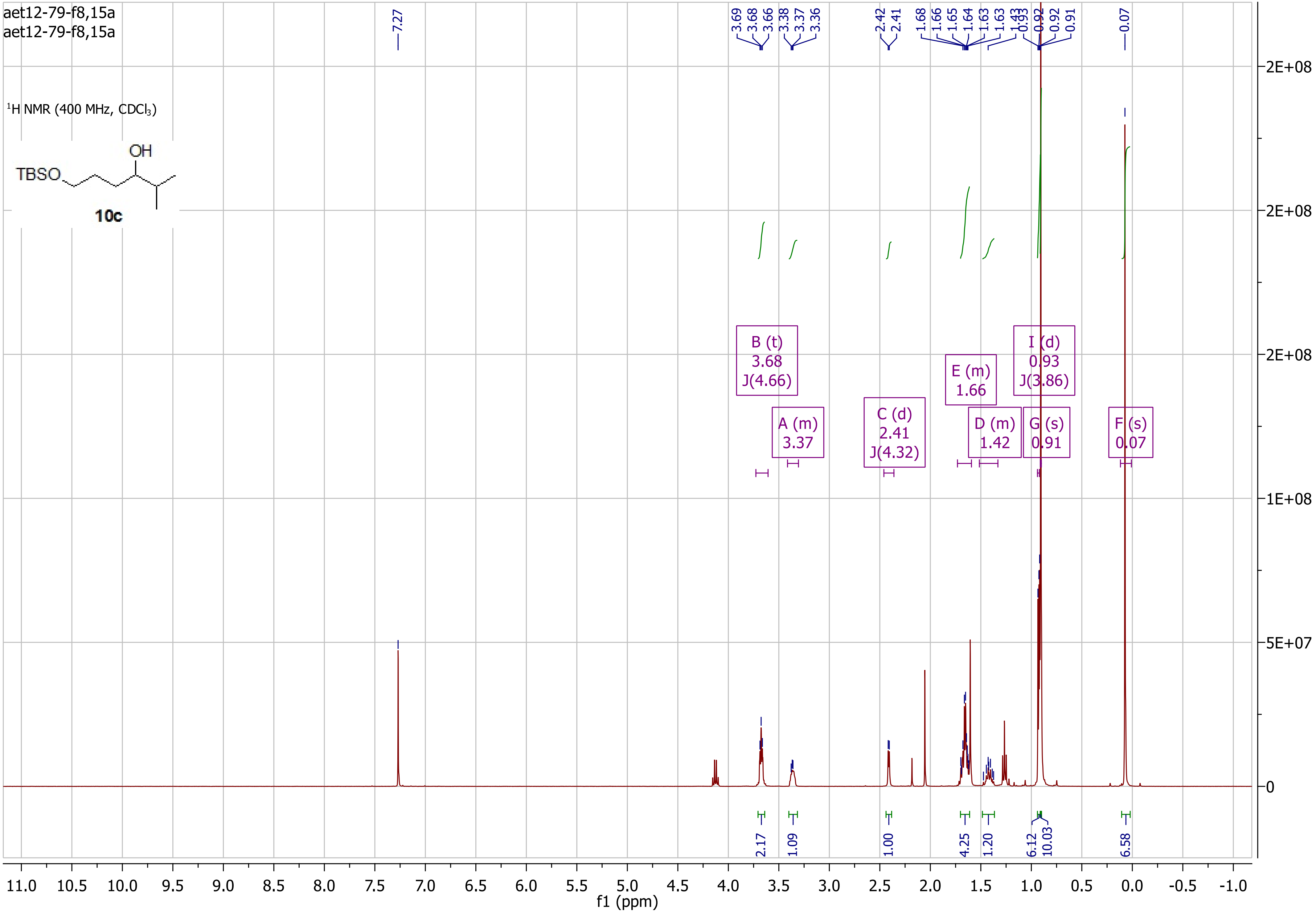

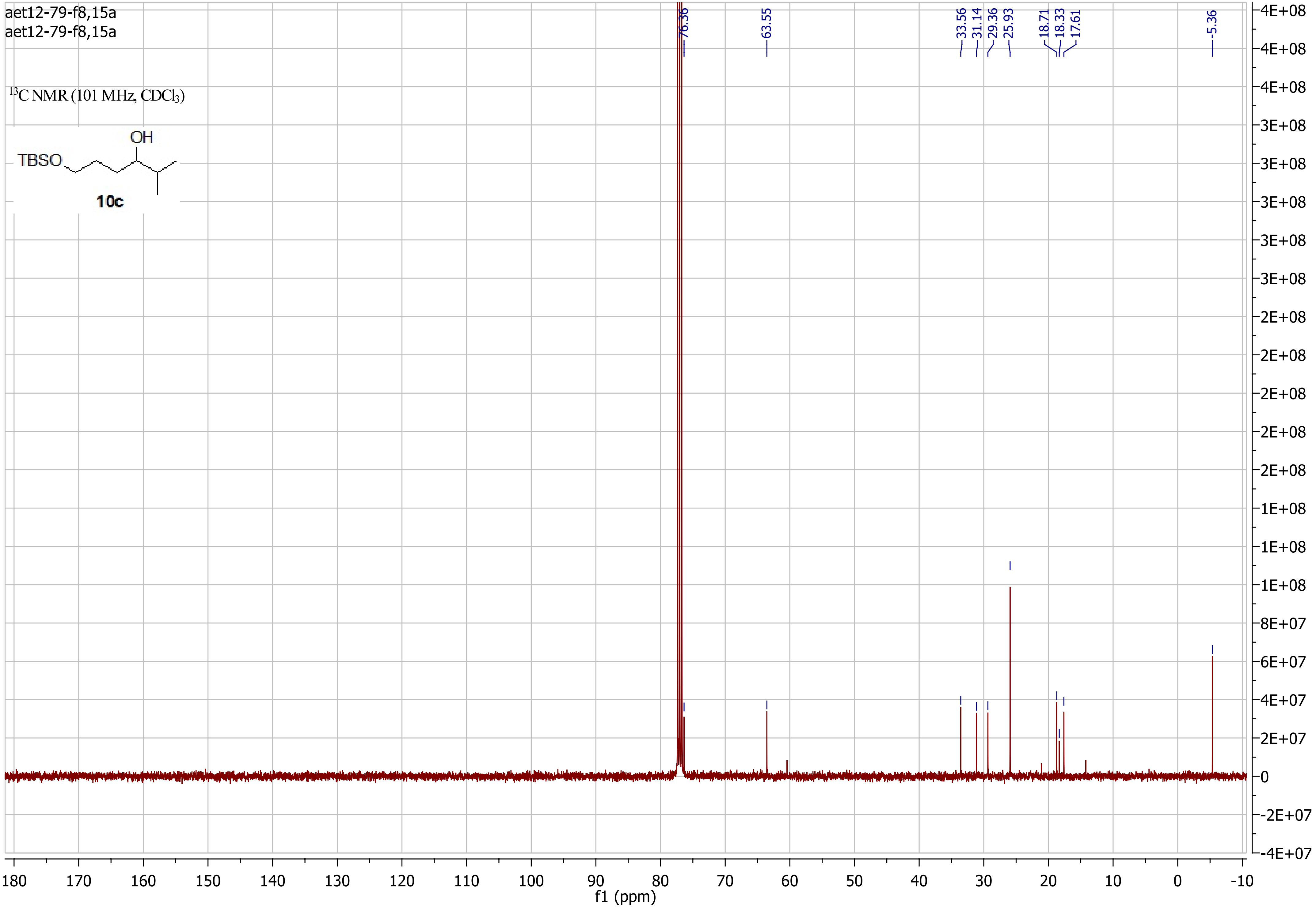
aet12-79-f8,15a
aet12-79-f8,15a
13C NMR (101 MHz, CDCl3)
OH
TBSO
10c
76.36
63.55
33.56
31.14
29.36
25.93
18.71
18.33
17.61
-5.36
f1 (ppm)

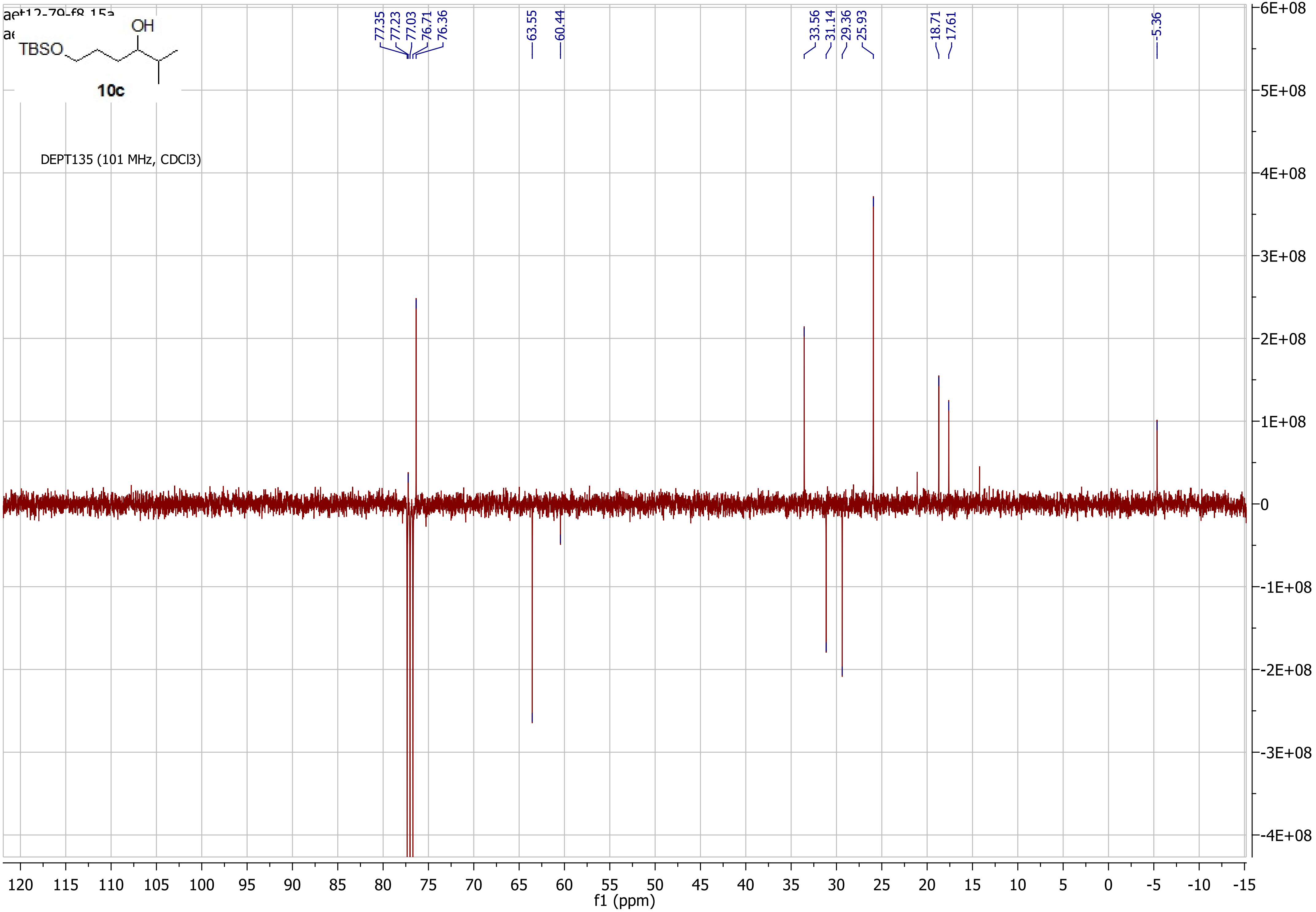

aet12-79-f8.15a
OH
TBSO
10c
DEPT135 (101 MHz, CDCl3)
77.35
77.23
77.03
76.71
76.36
63.55
60.44
33.56
31.14
29.36
25.93
18.71
17.61
-5.36
6E+08
5E+08
4E+08
3E+08
2E+08
1E+08
0
-1E+08
-2E+08
-3E+08
-4E+08
120 115 110 105 100 95 90 85 80 75 70 65 60 55 50 45 40 35 30 25 20 15 10 5 0 -5 -10 -15
f1 (ppm)

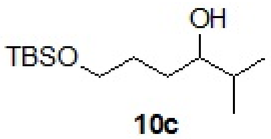

10c

COSY (400 MHz, CDCl3)

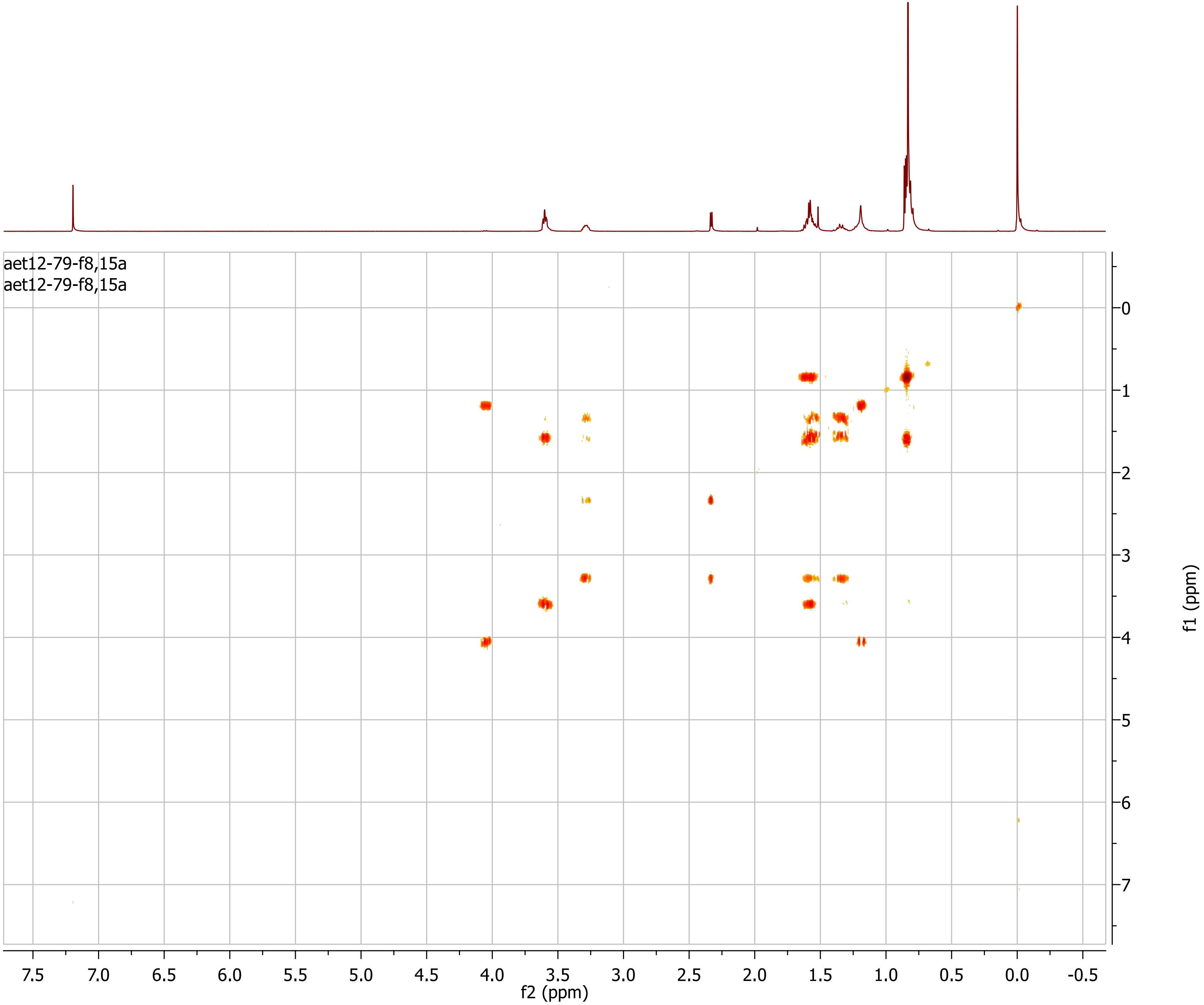

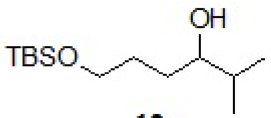

10c

HMQC (CDCl3)

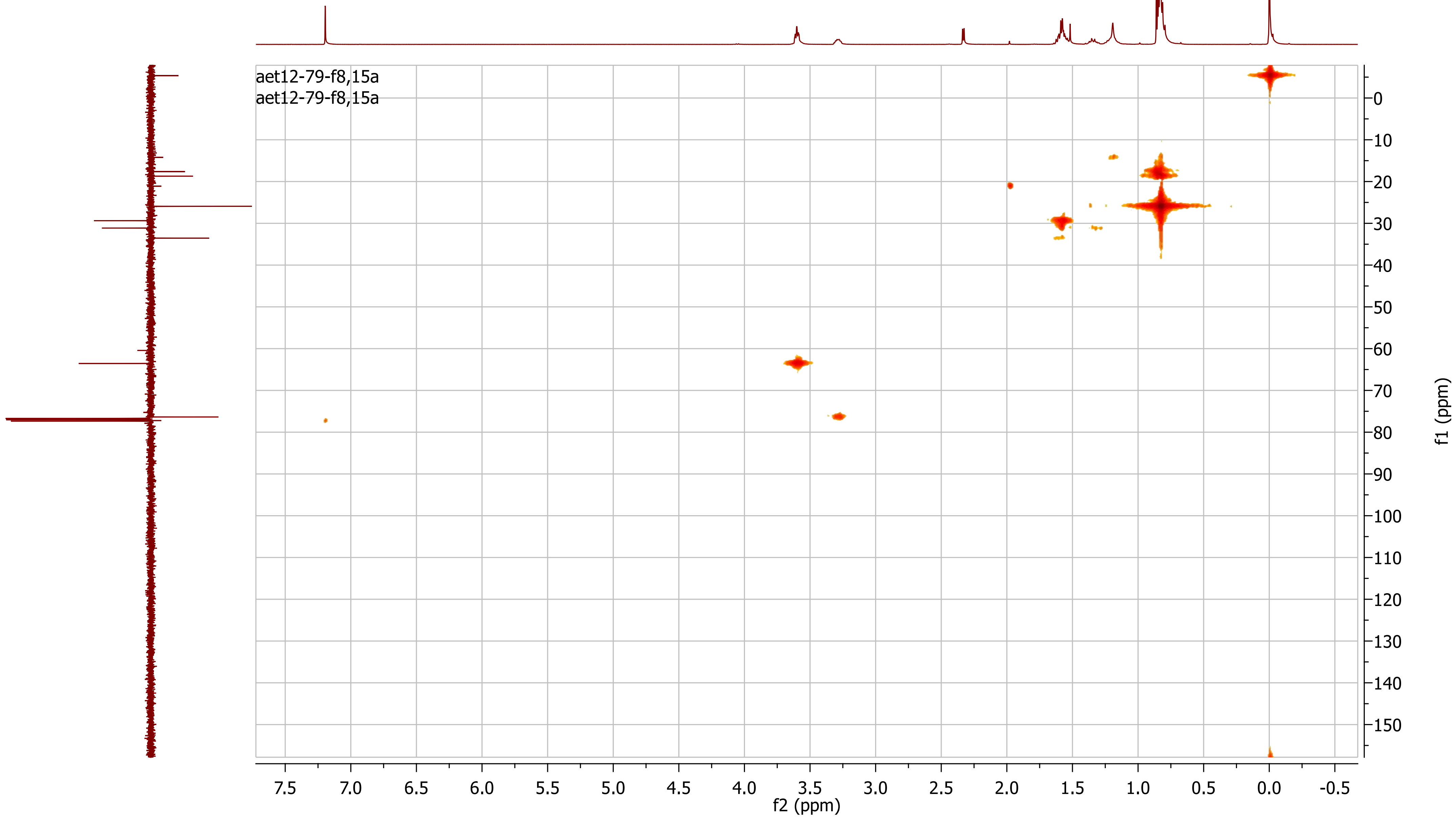

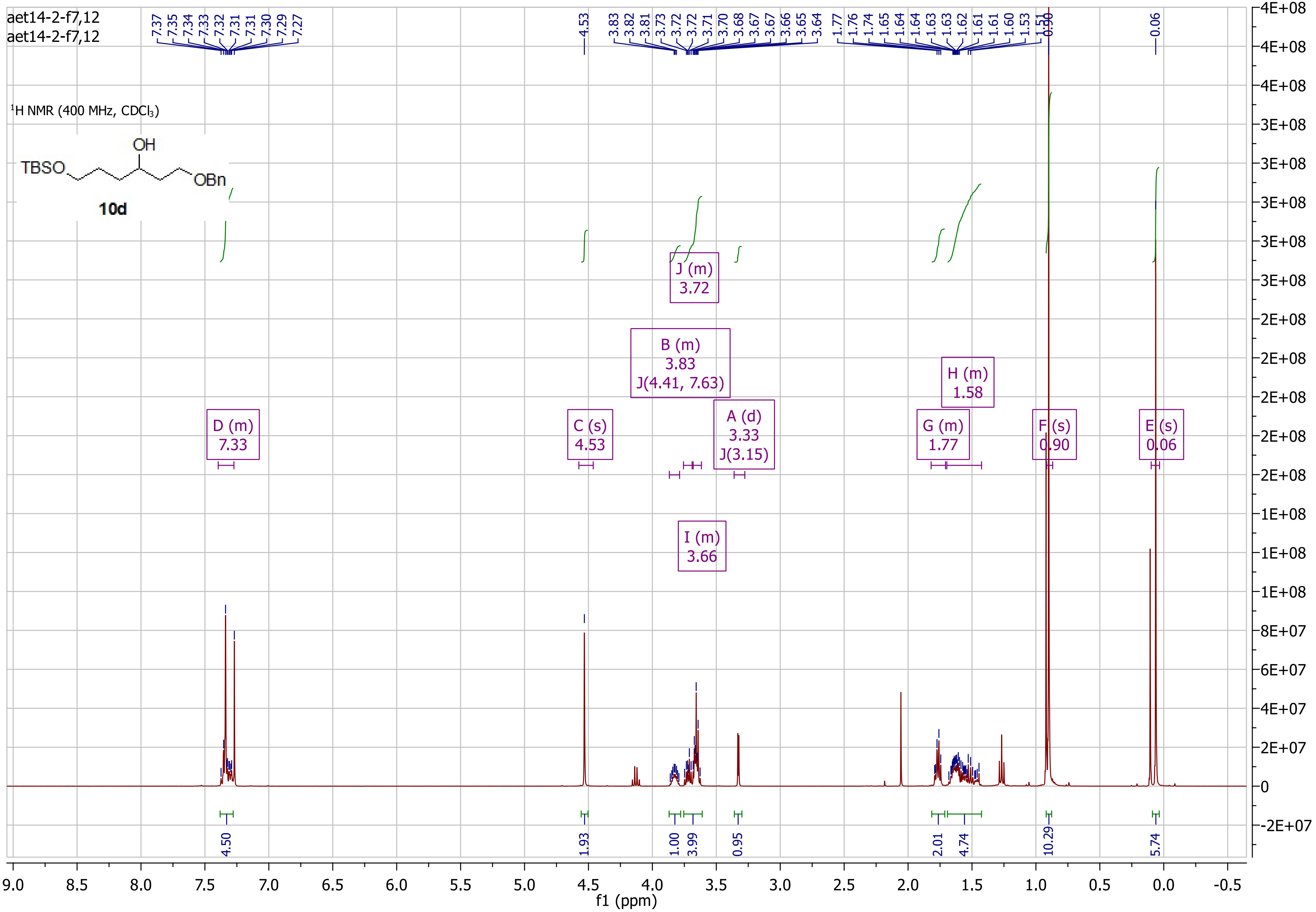

aet14-2-f7,12
aet14-2-f7,12
1H NMR (400 MHz, CDCl3)
OH
TBSO
OBn
10d
7.37 7.35 7.34 7.33 7.32 7.31 7.31 7.30 7.29 7.27
4.53
3.83 3.82 3.81 3.73 3.72 3.72 3.71 3.70 3.68 3.67 3.67 3.66 3.65 3.64
1.77 1.76 1.74 1.65 1.64 1.64 1.63 1.63 1.62 1.61 1.61 1.60 1.53 1.51 0.90
0.06
J (m) 3.72
B (m) 3.83 J(4.41, 7.63)
H (m) 1.58
D (m) 7.33
C (s) 4.53
A (d) 3.33 J(3.15)
G (m) 1.77
F (s) 0.90
E (s) 0.06
I (m) 3.66
4.50
1.93
1.00
3.99
0.95
2.01
4.74
10.29
5.74
9.0 8.5 8.0 7.5 7.0 6.5 6.0 5.5 5.0 4.5 4.0 3.5 3.0 2.5 2.0 1.5 1.0 0.5 0.0 -0.5
f1 (ppm)

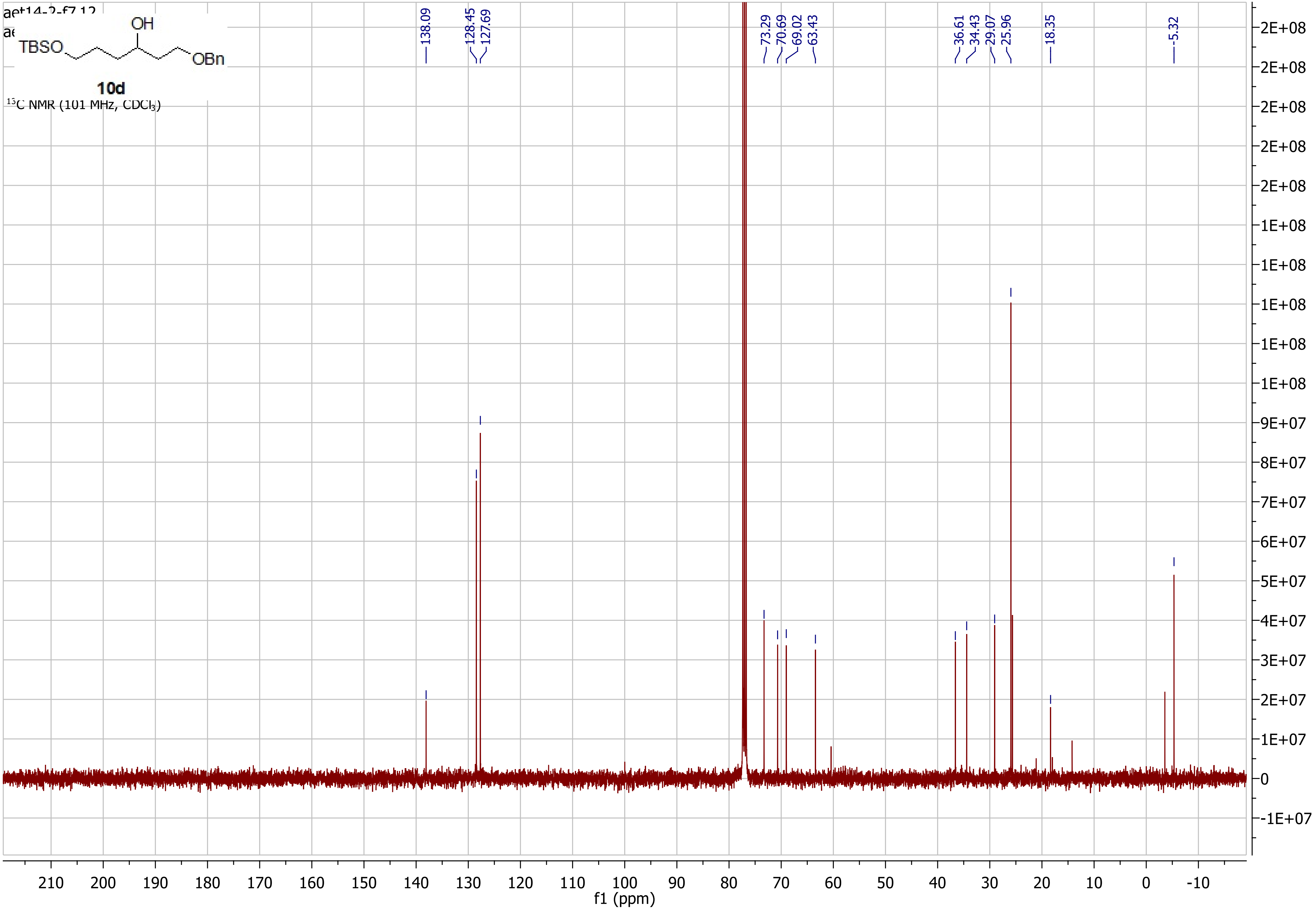

OH
TBSO
OBn
10d
13C NMR (101 MHz, CDCl3)
138.09
128.45
127.69
73.29
70.69
69.02
63.43
36.61
34.43
29.07
25.96
18.35
-5.32
f1 (ppm)

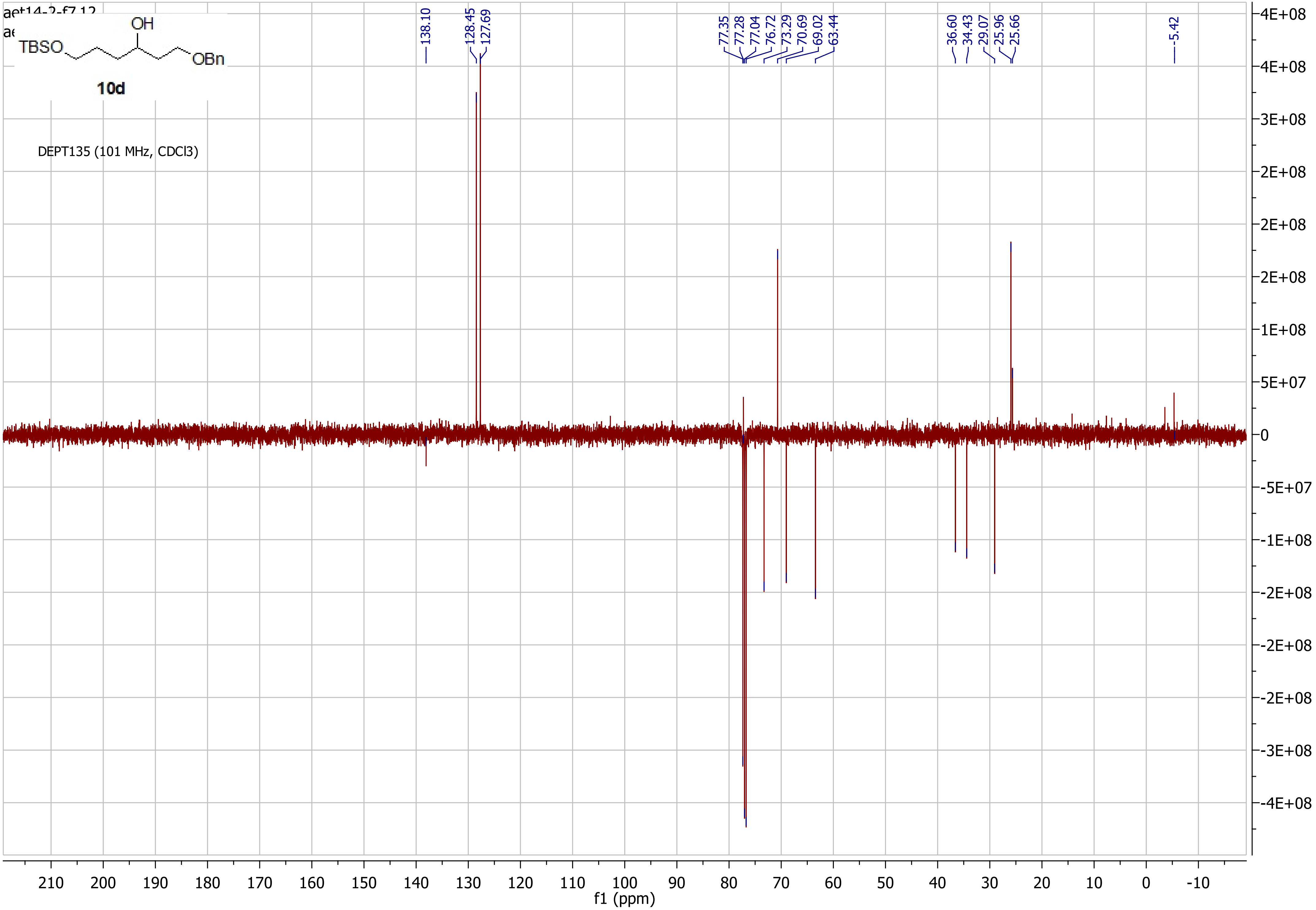

OH
TBSO
OBn
10d
DEPT135 (101 MHz, CDCl3)
138.10
128.45
127.69
77.35
77.28
77.04
76.72
73.29
70.69
69.02
63.44
36.60
34.43
29.07
25.96
25.66
-5.42
f1 (ppm)

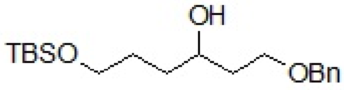

10d

COSY (400 MHz, CDCl3)

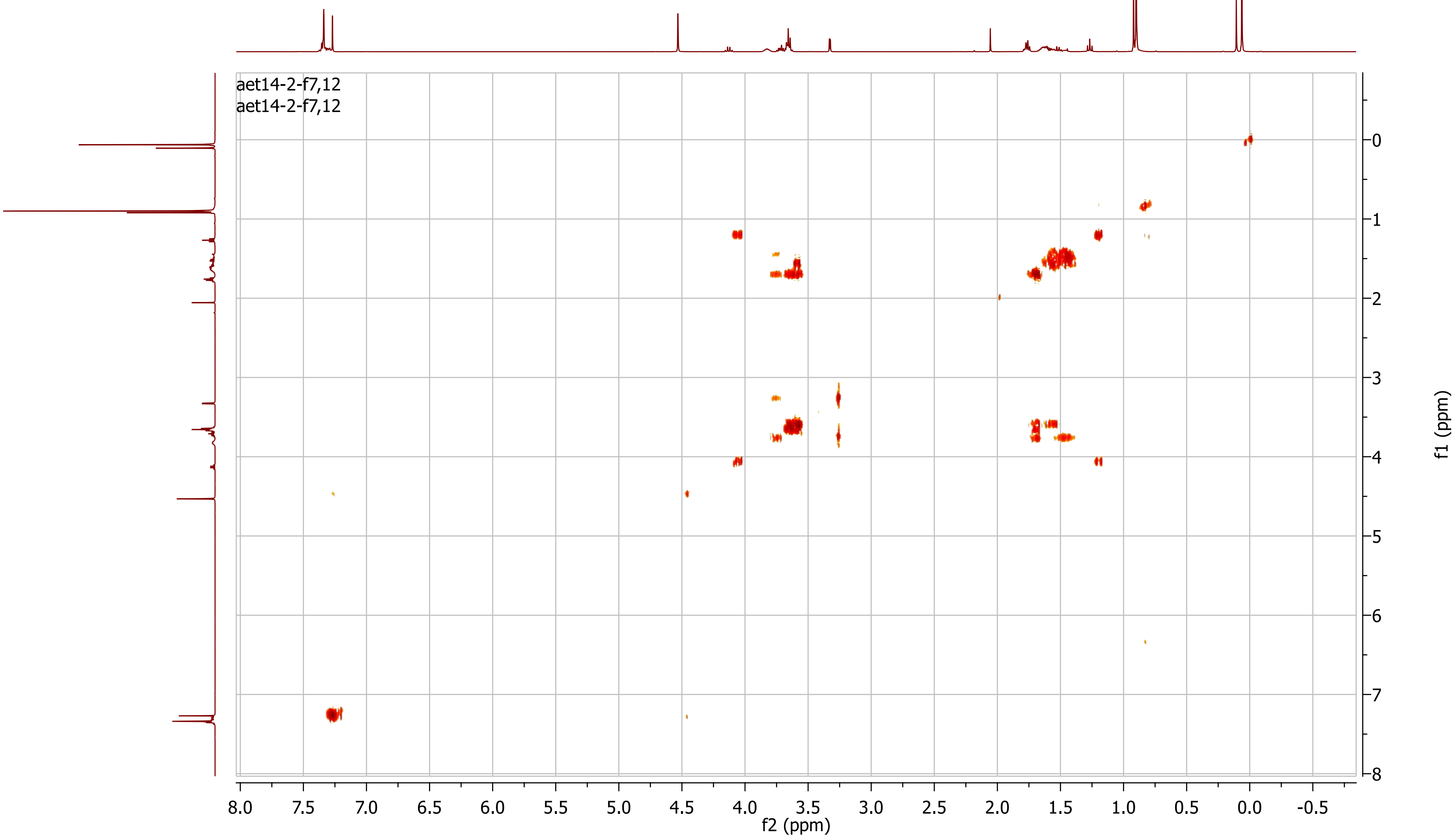

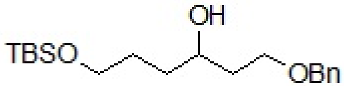

10d

HMQC (CDCl3)

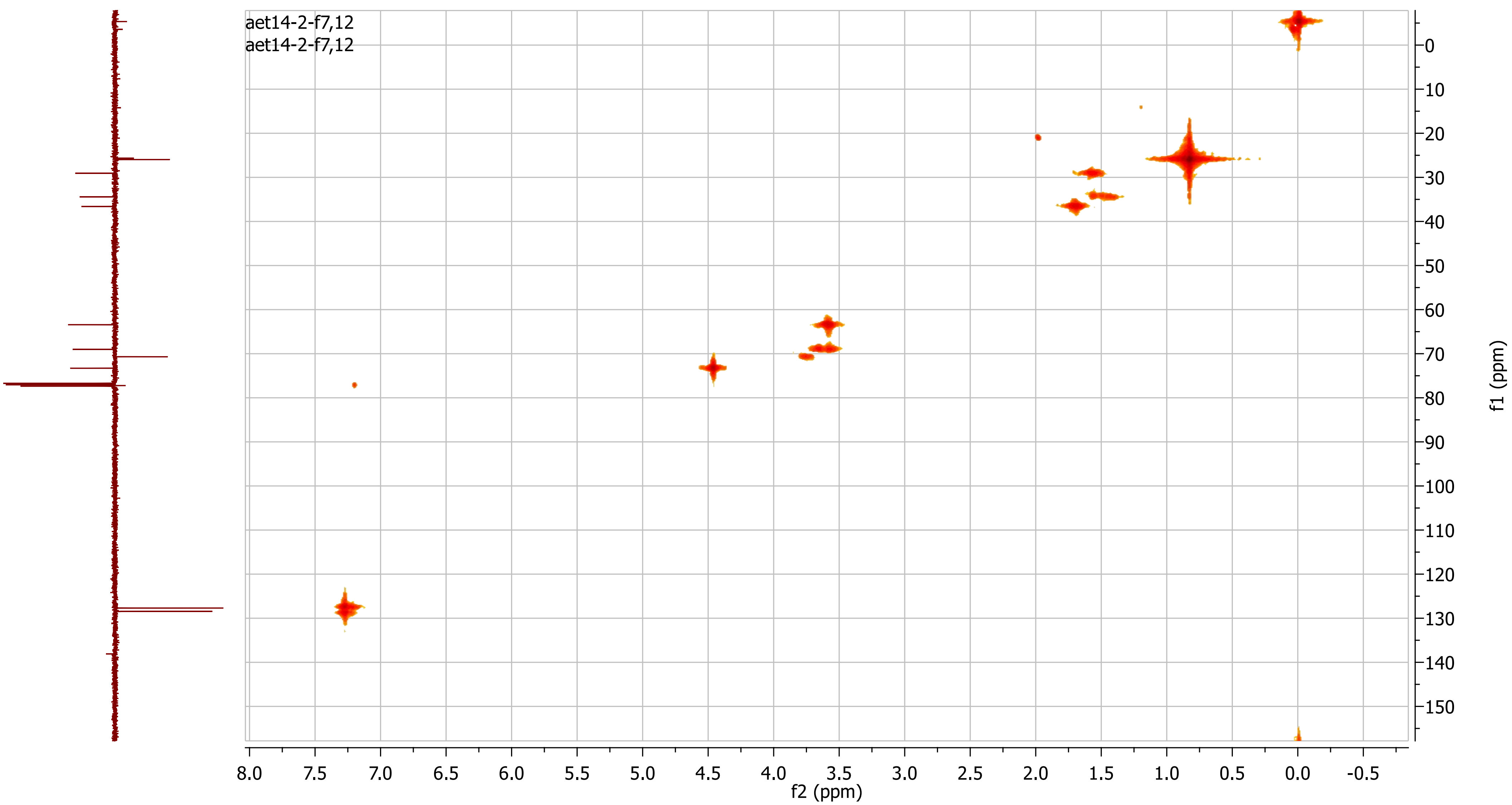

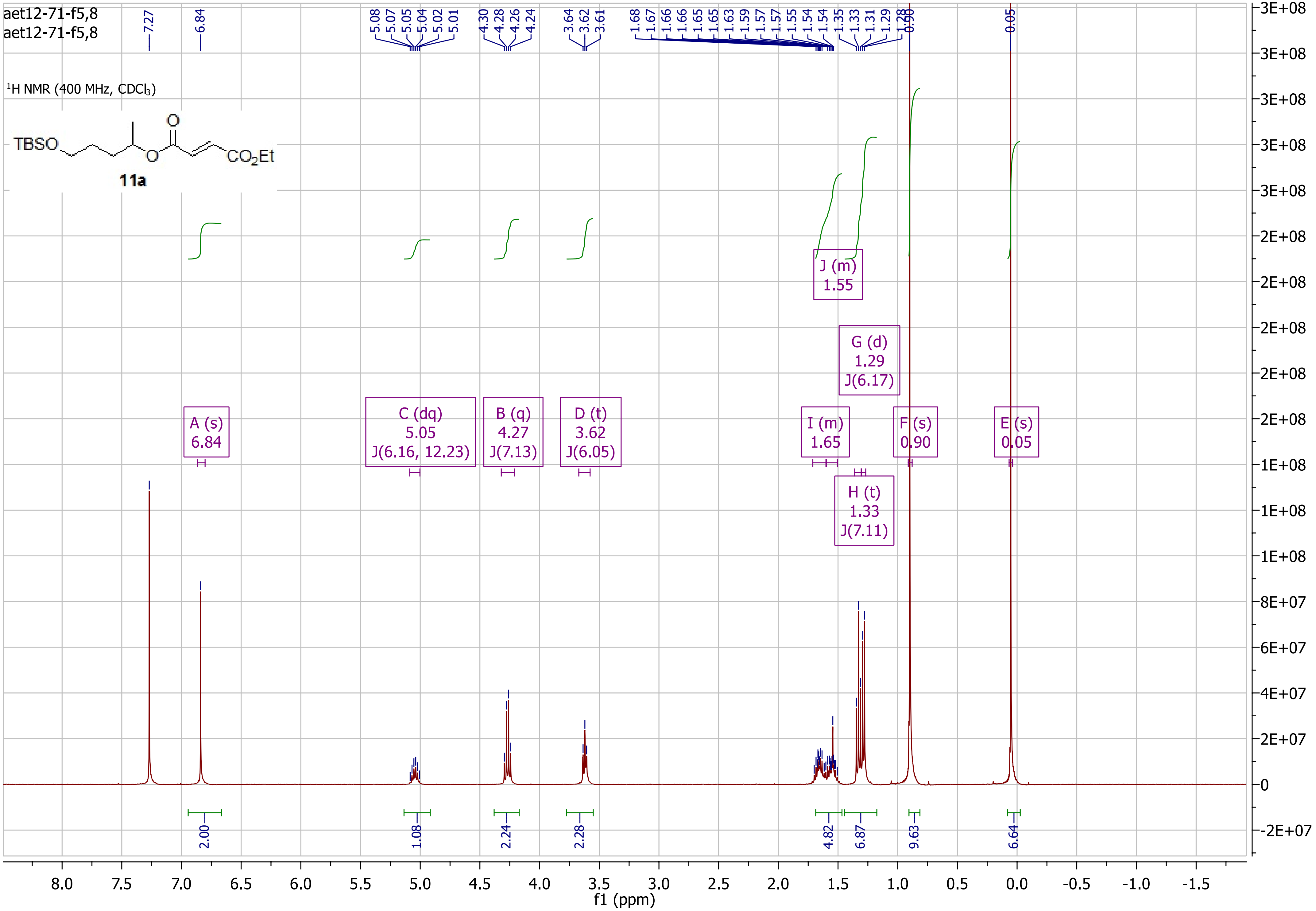

aet12-71-f5,8
aet12-71-f5,8
1H NMR (400 MHz, CDCl3)
TBSO
O
O
CO2Et
11a
7.27
6.84
5.08 5.07 5.05 5.04 5.02 5.01
4.30 4.28 4.26 4.24
3.64 3.62 3.61
1.68 1.67 1.66 1.66 1.65 1.65 1.63 1.59 1.57 1.57 1.55 1.54 1.54 1.35 1.33 1.31 1.29 1.28 0.90
0.05
A (s) 6.84
C (dq) 5.05 J(6.16, 12.23)
B (q) 4.27 J(7.13)
D (t) 3.62 J(6.05)
J (m) 1.55
G (d) 1.29 J(6.17)
I (m) 1.65
H (t) 1.33 J(7.11)
F (s) 0.90
E (s) 0.05
2.00
1.08
2.24
2.28
4.82
6.87
9.63
6.64
f1 (ppm)

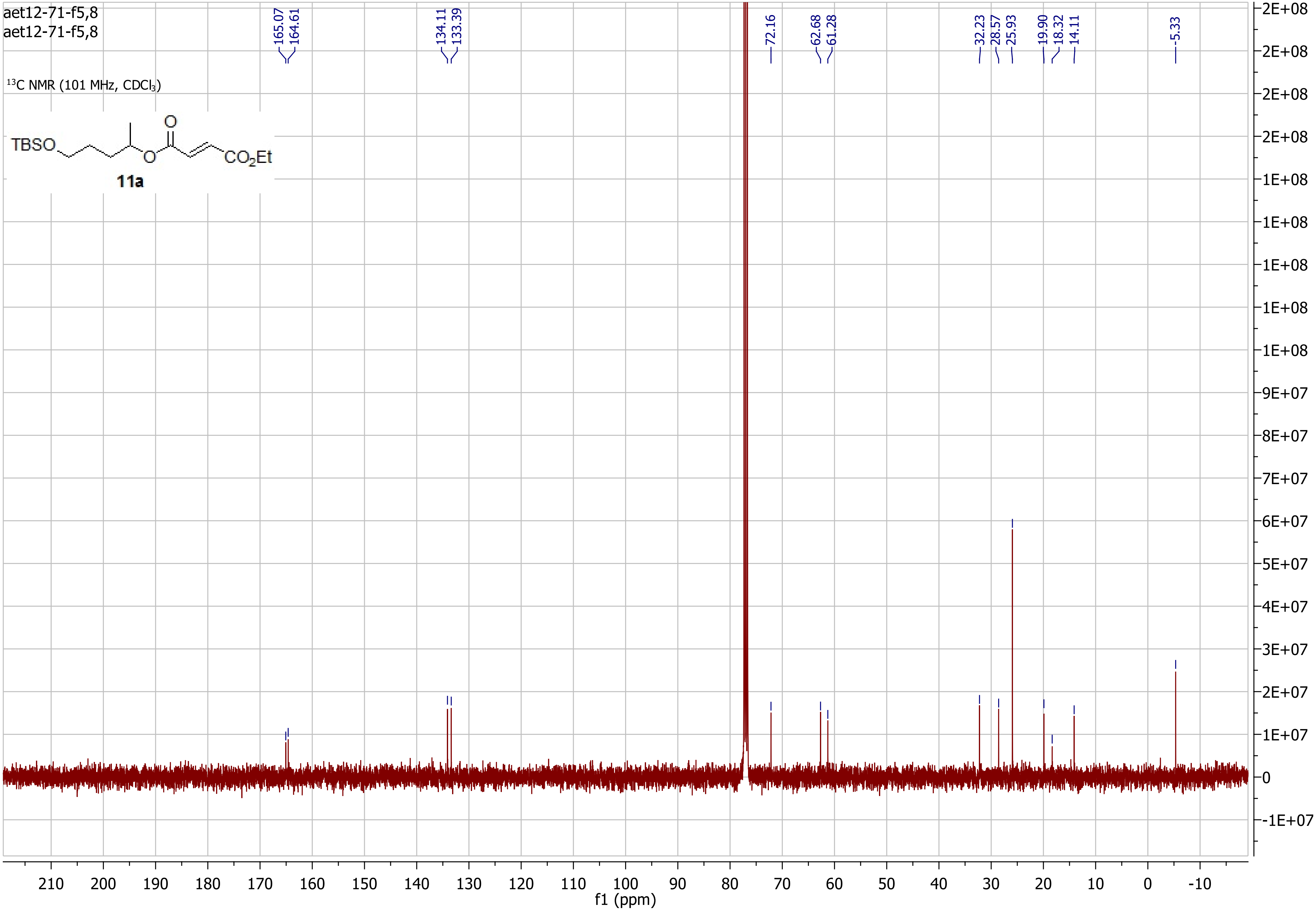
aet12-71-f5,8
aet12-71-f5,8
^{13}C NMR (101 MHz, CDCl$_3$)
TBSO
O
O
CO$_2$Et
11a
165.07
164.61
134.11
133.39
72.16
62.68
61.28
32.23
28.57
25.93
19.90
18.32
14.11
-5.33
f1 (ppm)

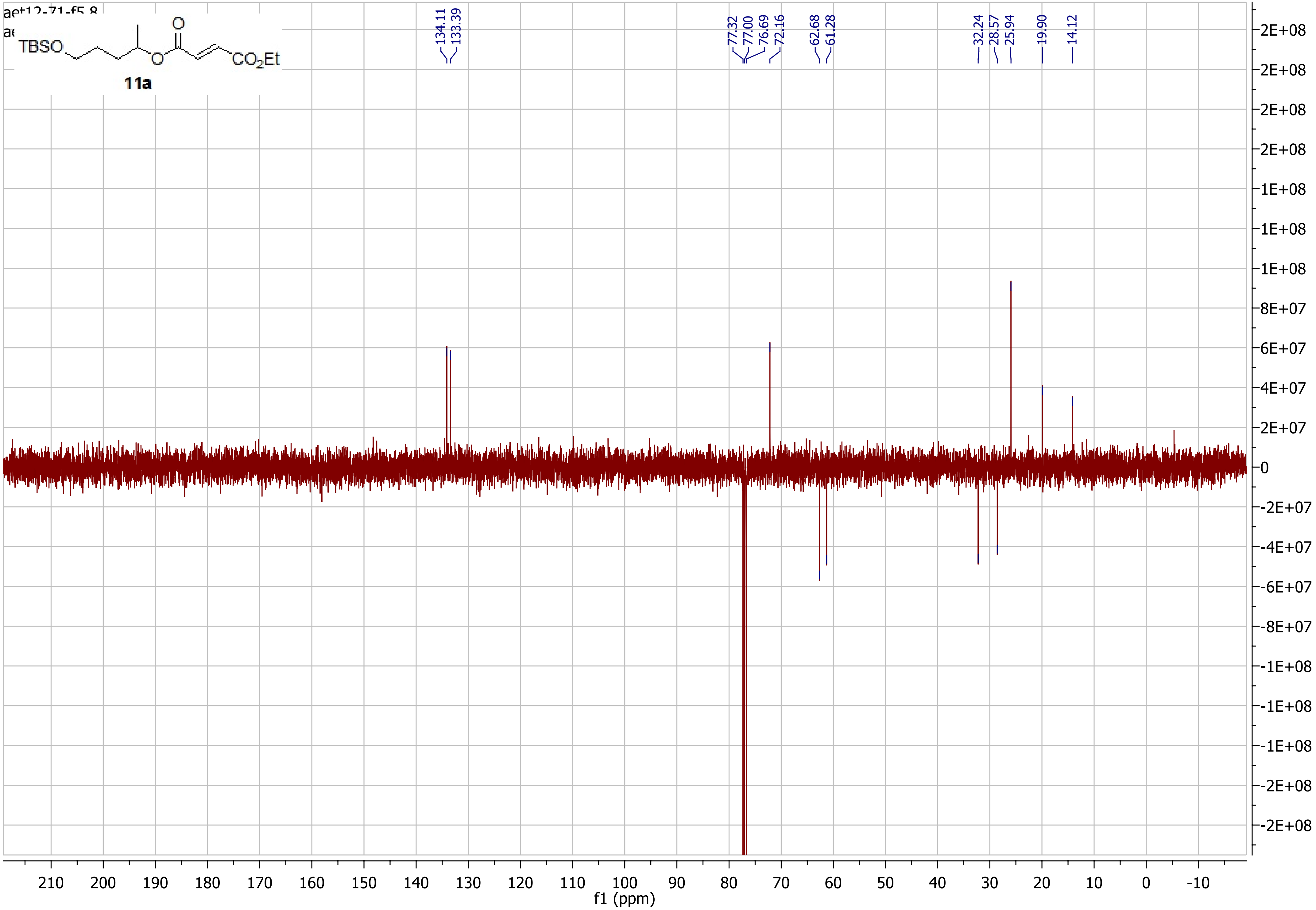
aet12-71-f5.8
TBSO
O
O
CO_2Et
11a
134.11
133.39
77.32
77.00
76.69
72.16
62.68
61.28
32.24
28.57
25.94
19.90
14.12
210 200 190 180 170 160 150 140 130 120 110 100 90 80 70 60 50 40 30 20 10 0 -10
f1 (ppm)
2E+08
1E+08
8E+07
6E+07
4E+07
2E+07
0
-2E+07
-4E+07
-6E+07
-8E+07
-1E+08
-2E+08

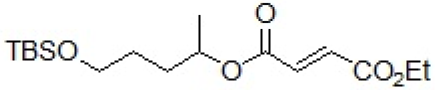

11a

COSY (400 MHz, CDCl3)

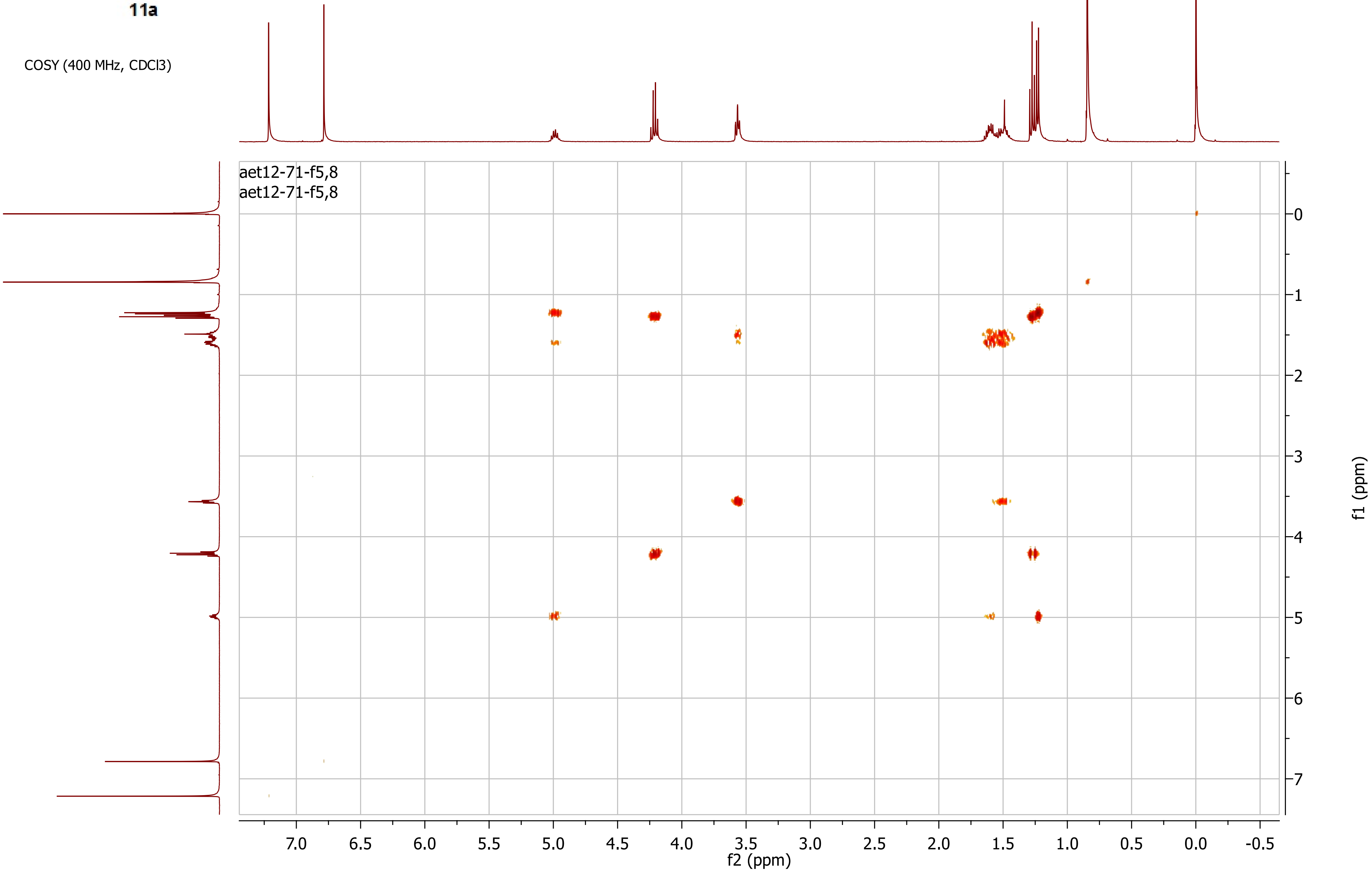

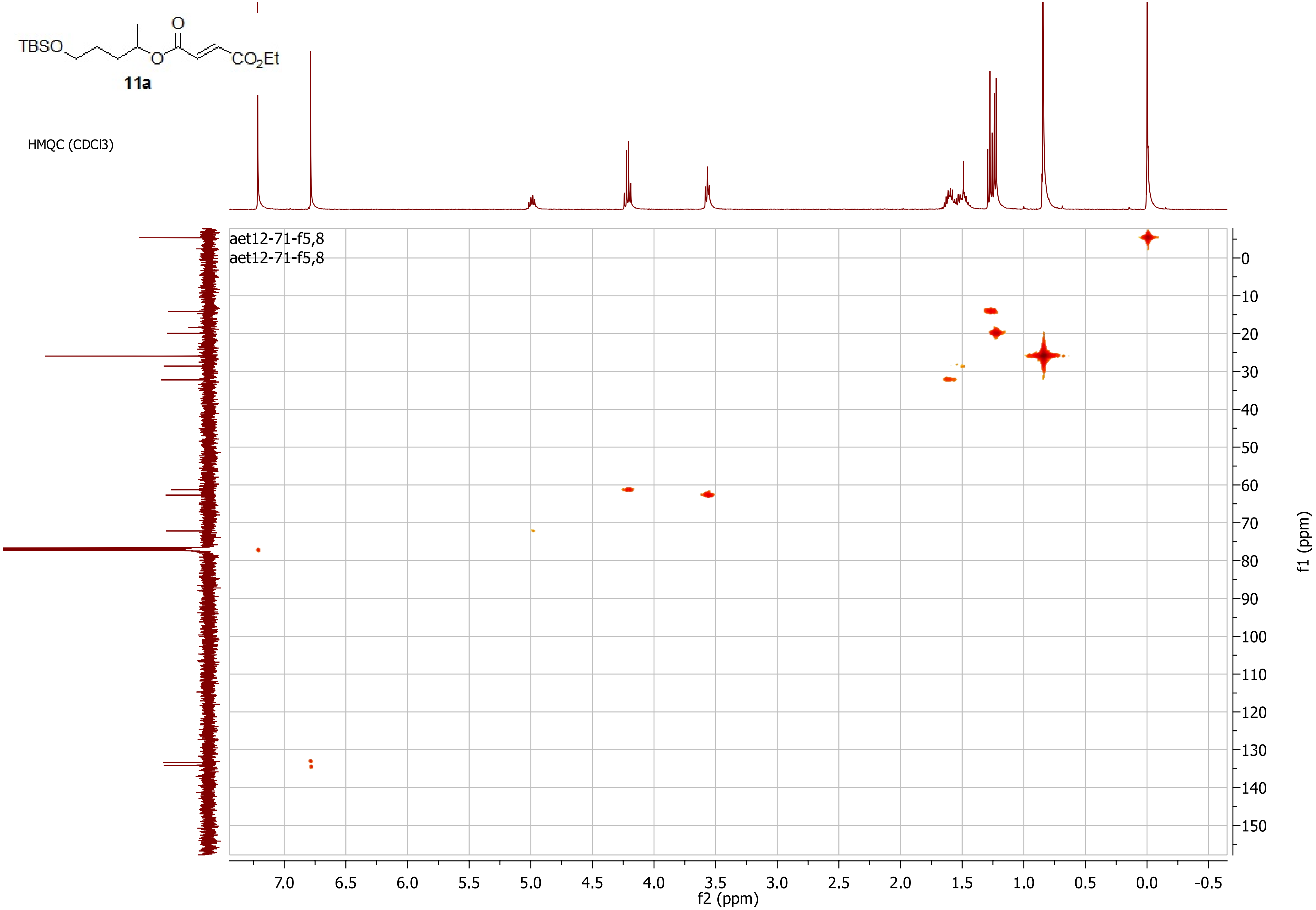
TBSO
O
O
CO2Et
11a
HMQC (CDCl3)
aet12-71-f5,8
aet12-71-f5,8
f2 (ppm)
f1 (ppm)

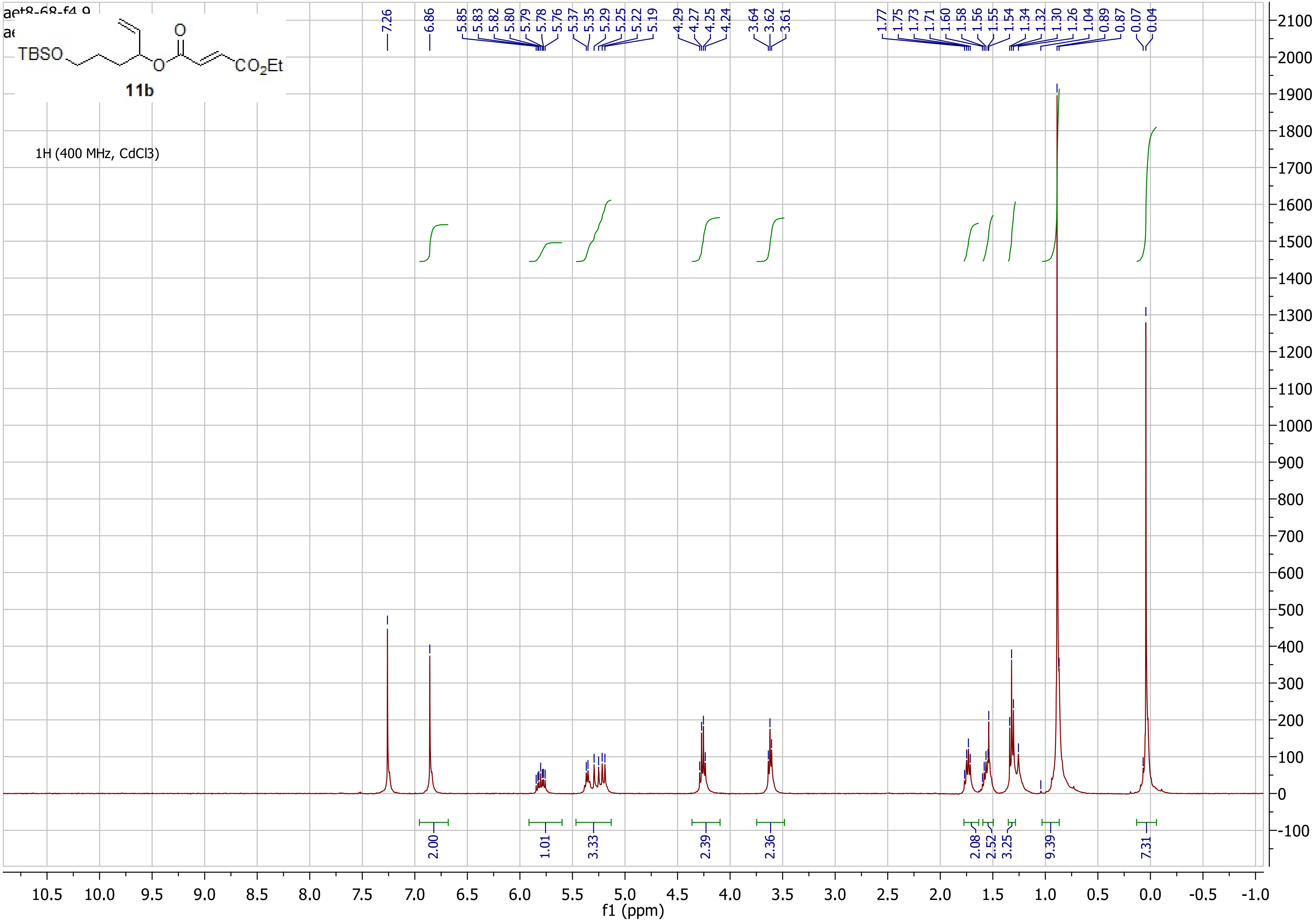

TBSO
O
CO2Et
11b
1H (400 MHz, CdCl3)
7.26
6.86
5.85
5.83
5.82
5.80
5.79
5.78
5.76
5.37
5.35
5.29
5.25
5.22
5.19
4.29
4.27
4.25
4.24
3.64
3.62
3.61
1.77
1.75
1.73
1.71
1.60
1.58
1.56
1.55
1.54
1.34
1.32
1.30
1.26
1.04
0.89
0.87
0.07
0.04
2.00
1.01
3.33
2.39
2.36
2.08
2.52
3.25
9.39
7.31
f1 (ppm)

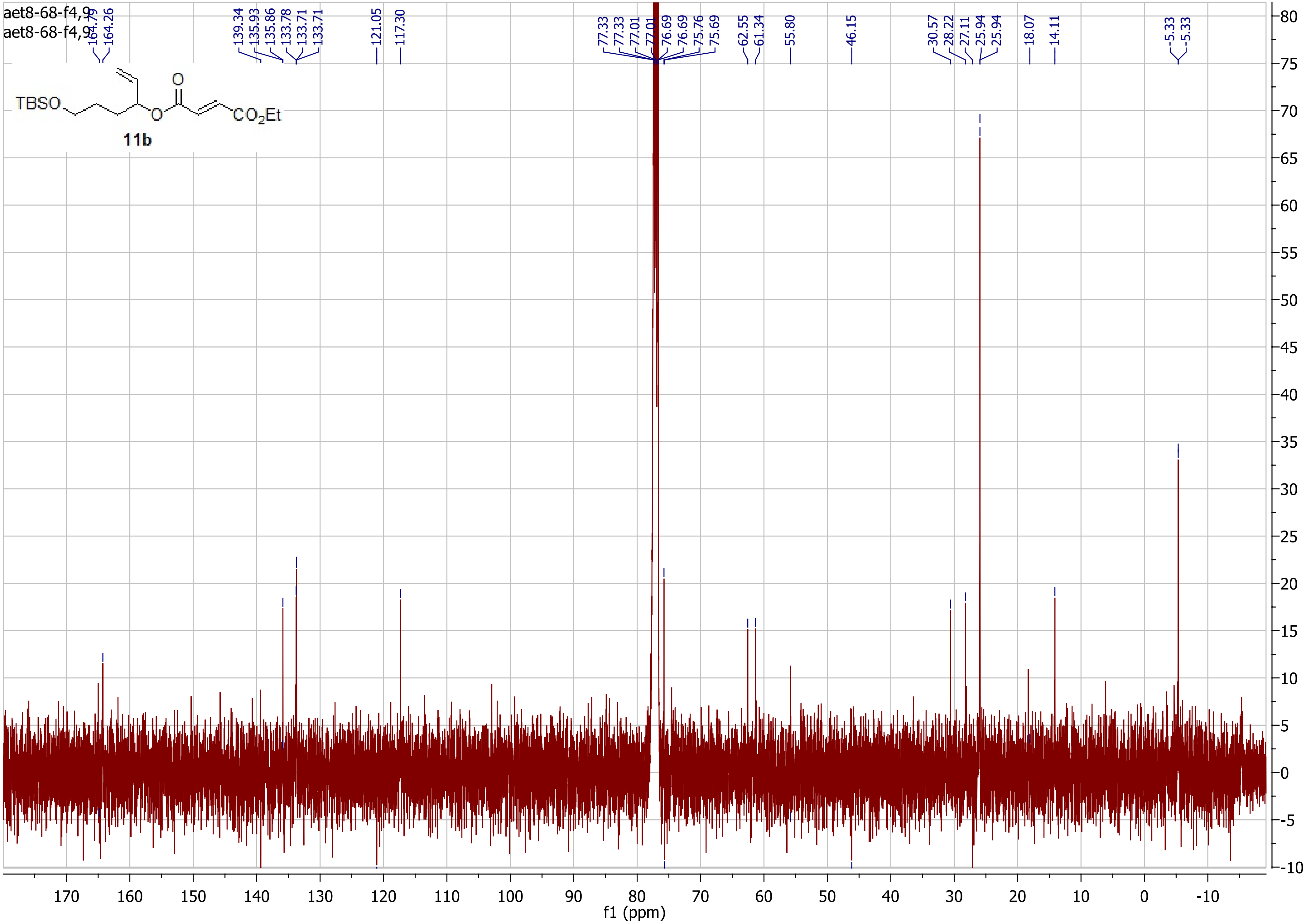
aet8-68-f4,9
aet8-68-f4,9
164.79
164.26
139.34
135.93
135.86
133.78
133.71
133.71
121.05
117.30
77.33
77.33
77.01
77.01
76.69
76.69
75.76
75.69
62.55
61.34
55.80
46.15
30.57
28.22
27.11
25.94
25.94
18.07
14.11
-5.33
-5.33
TBSO
O
O
CO_2Et
11b
f1 (ppm)

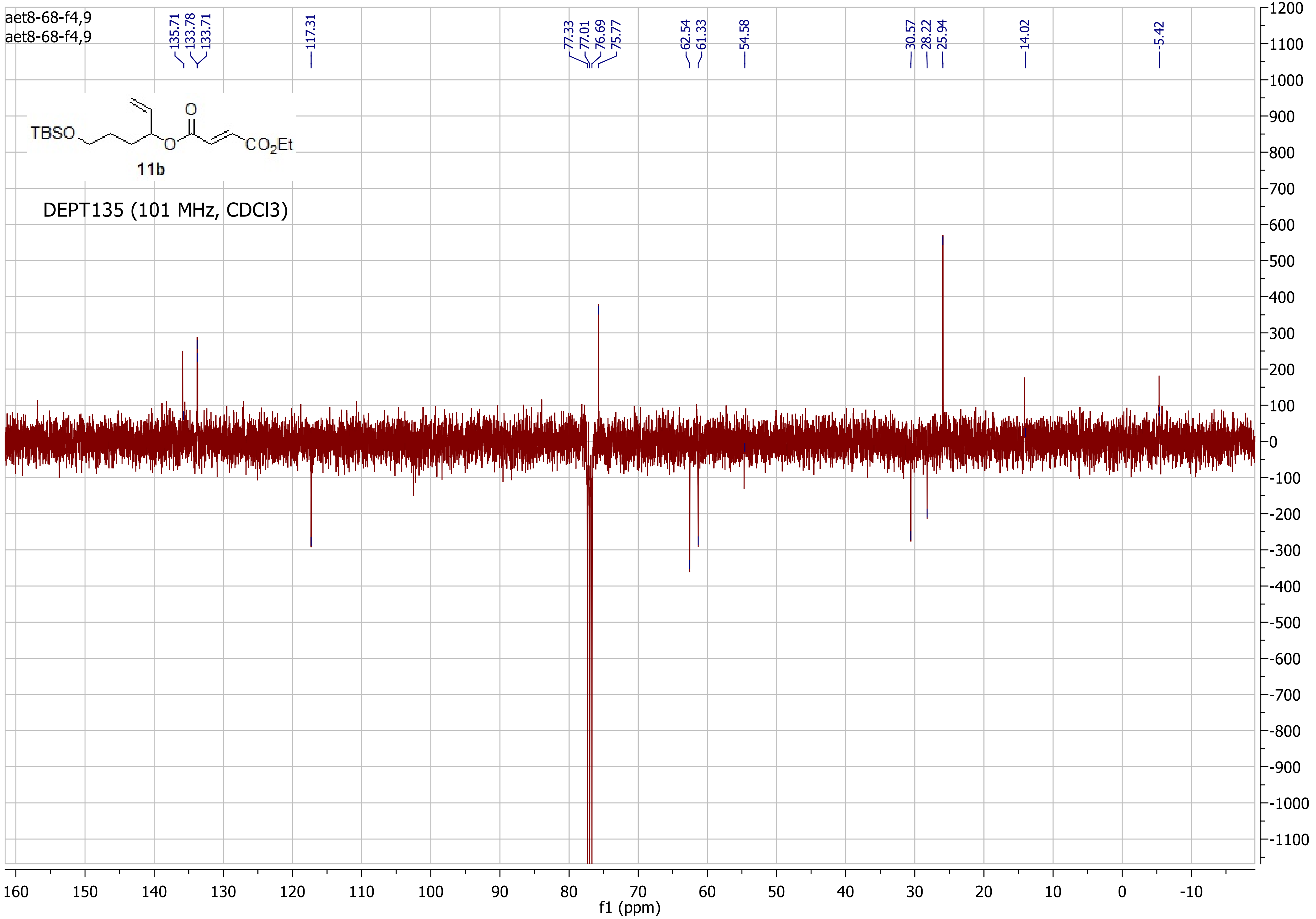
aet8-68-f4,9
aet8-68-f4,9
135.71
133.78
133.71
117.31
77.33
77.01
76.69
75.77
62.54
61.33
54.58
30.57
28.22
25.94
14.02
-5.42
TBSO
O
O
CO2Et
11b
DEPT135 (101 MHz, CDCl3)
f1 (ppm)

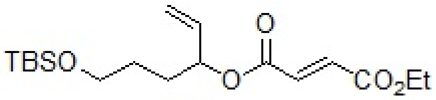

11b

COSY (400 MHz, CDCl3)

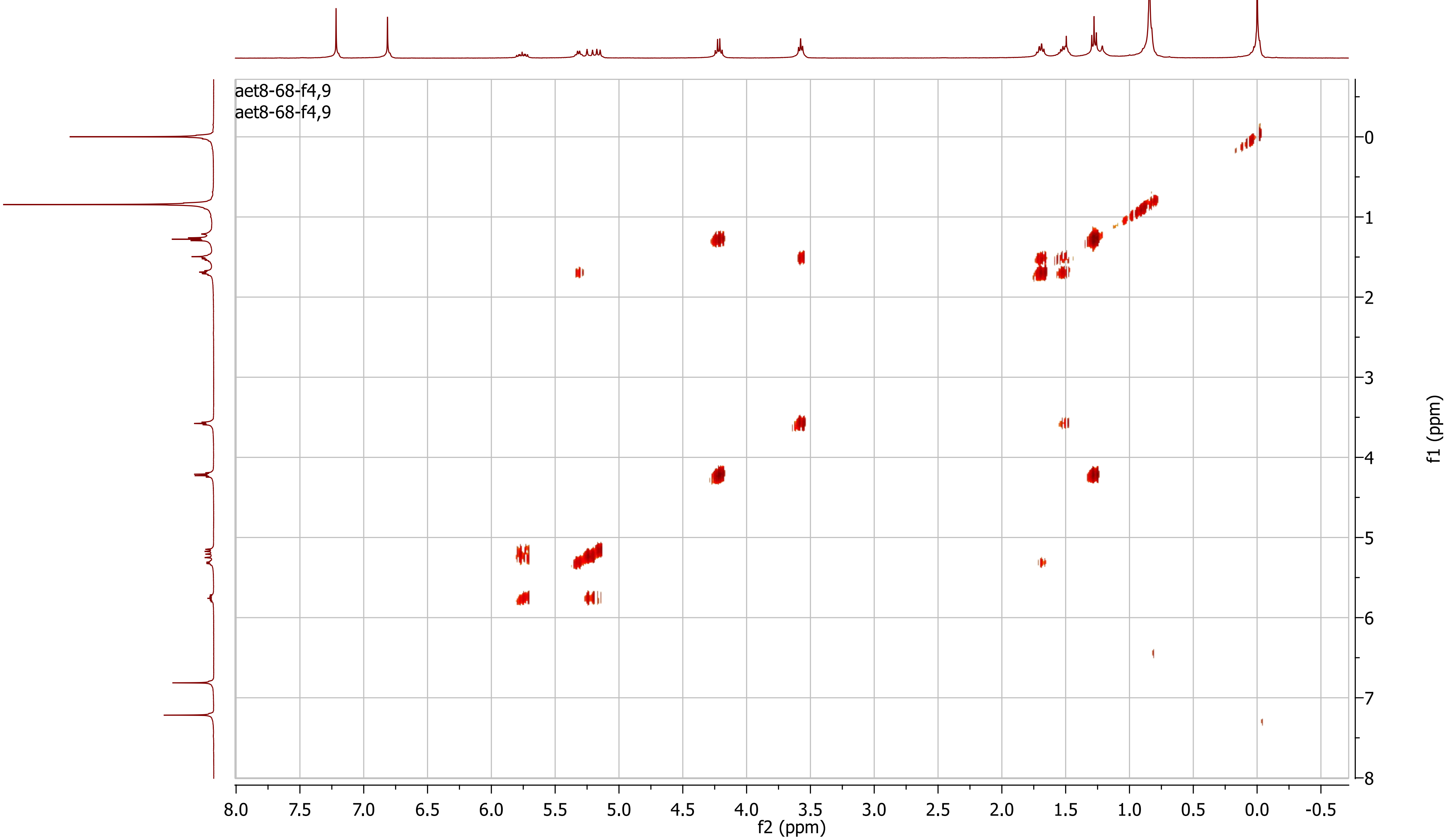

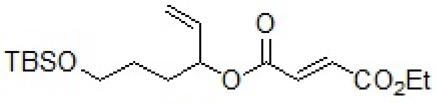

11b

HMQC (CDCl3)

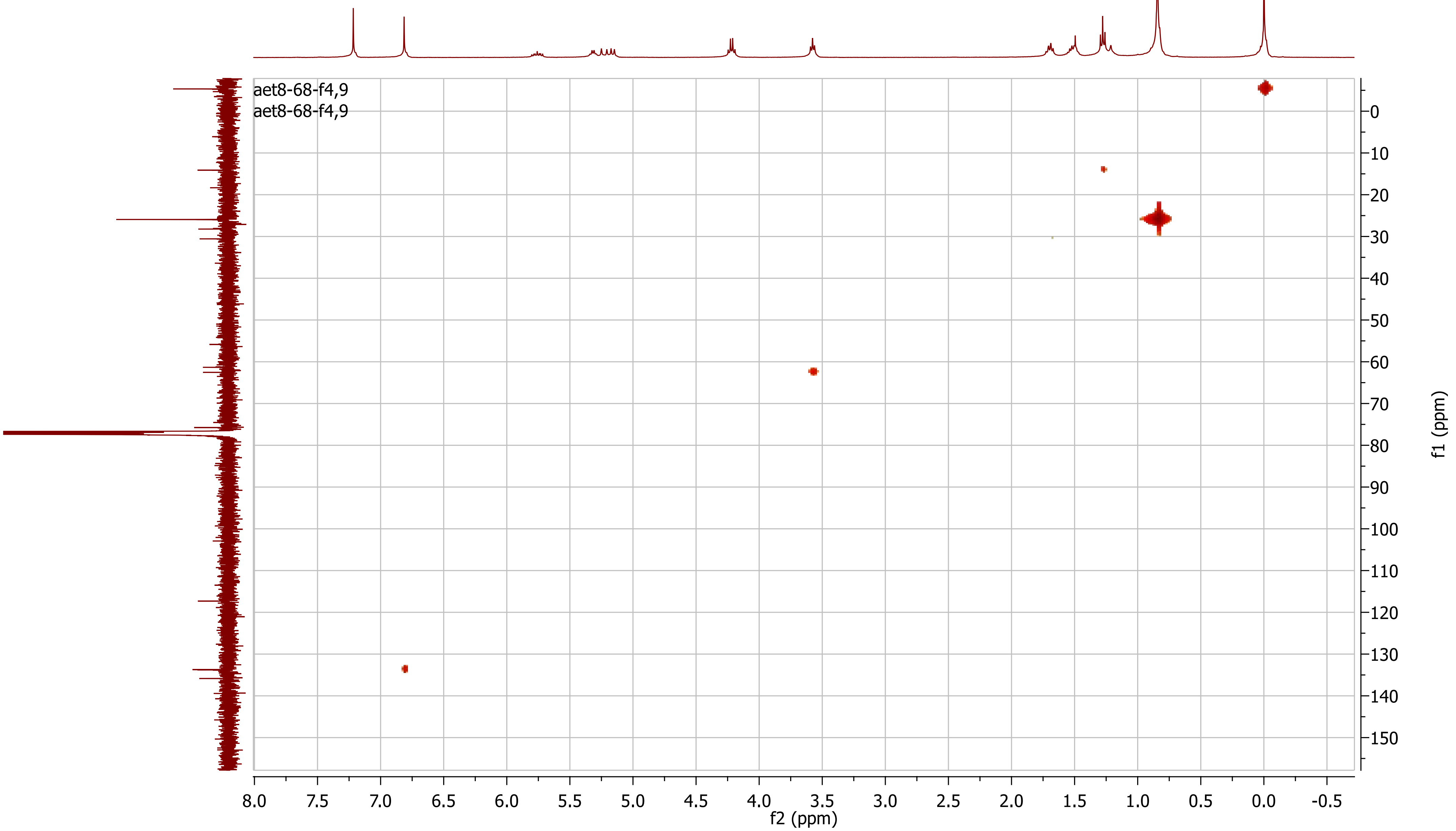

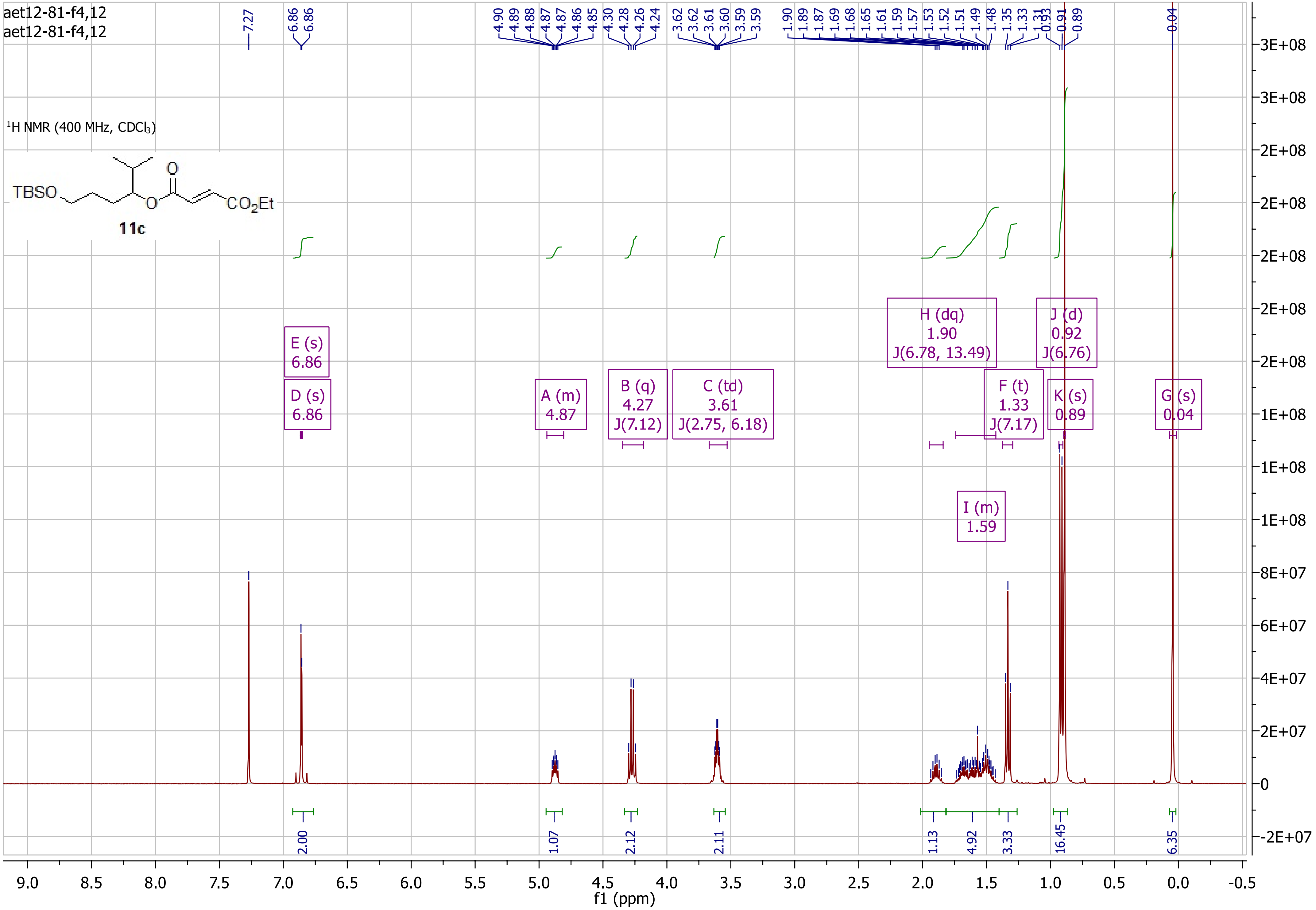
aet12-81-f4,12
aet12-81-f4,12
¹H NMR (400 MHz, CDCl₃)
TBSO
O
O
CO₂Et
11c
7.27
6.86
6.86
4.90 4.89 4.88 4.87 4.87 4.86 4.85
4.30 4.28 4.26 4.24
3.62 3.62 3.61 3.60 3.59 3.59
1.90 1.89 1.87 1.69 1.68 1.65 1.61 1.59 1.57 1.53 1.52 1.51 1.49 1.48 1.35 1.33 1.31 0.93 0.91 0.89
0.04
E (s) 6.86
D (s) 6.86
A (m) 4.87
B (q) 4.27 J(7.12)
C (td) 3.61 J(2.75, 6.18)
H (dq) 1.90 J(6.78, 13.49)
I (m) 1.59
F (t) 1.33 J(7.17)
J (d) 0.92 J(6.76)
K (s) 0.89
G (s) 0.04
2.00
1.07
2.12
2.11
1.13
4.92
3.33
16.45
6.35
f1 (ppm)

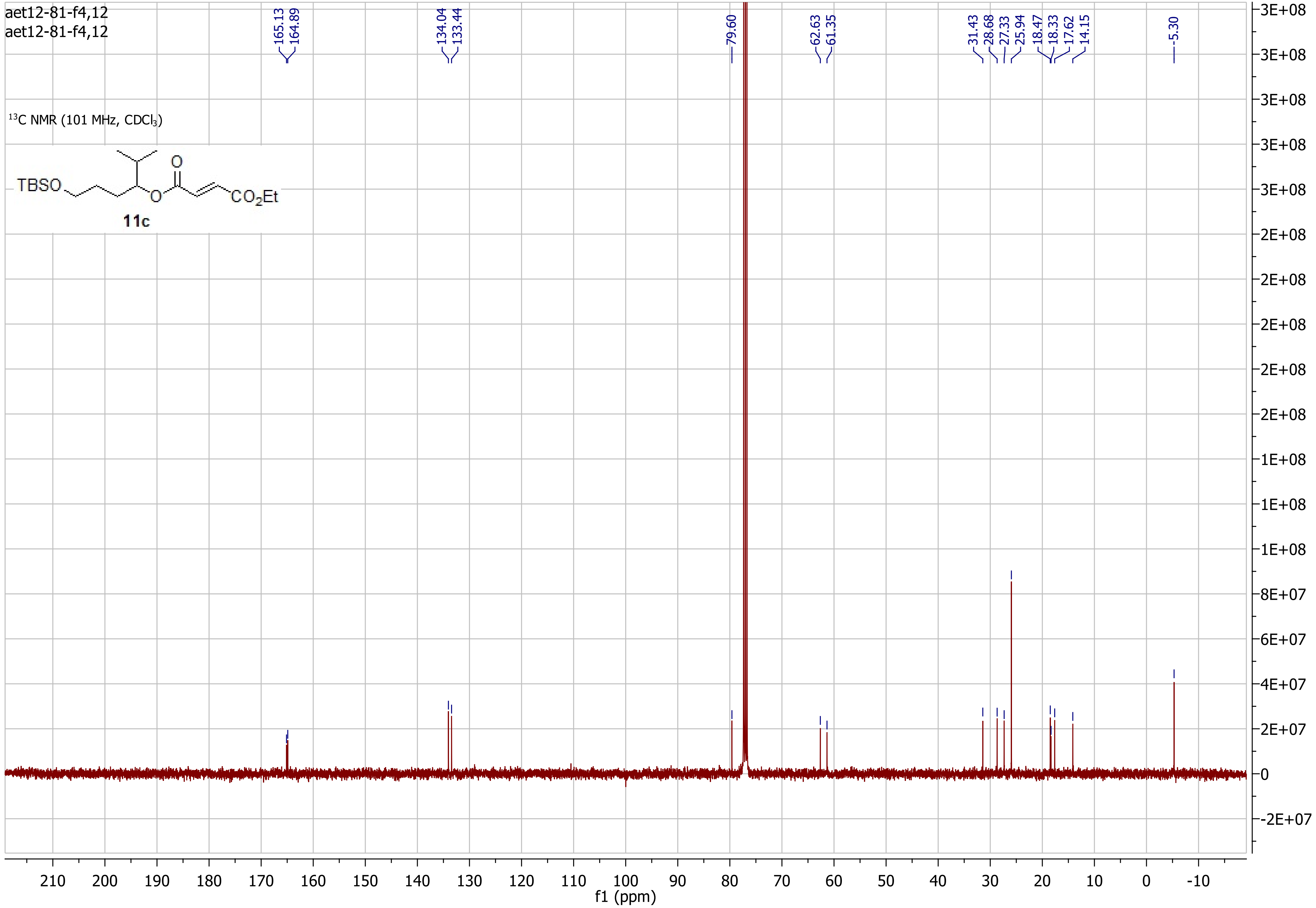

aet12-81-f4,12
aet12-81-f4,12
^{13}C NMR (101 MHz, $CDCl_3$)
TBSO
O
O
CO_2Et
11c
165.13
164.89
134.04
133.44
79.60
62.63
61.35
31.43
28.68
27.33
25.94
18.47
18.33
17.62
14.15
-5.30
3E+08
3E+08
3E+08
3E+08
3E+08
2E+08
2E+08
2E+08
2E+08
2E+08
1E+08
1E+08
1E+08
8E+07
6E+07
4E+07
2E+07
0
-2E+07
210
200
190
180
170
160
150
140
130
120
110
100
90
80
70
60
50
40
30
20
10
0
-10
f1 (ppm)

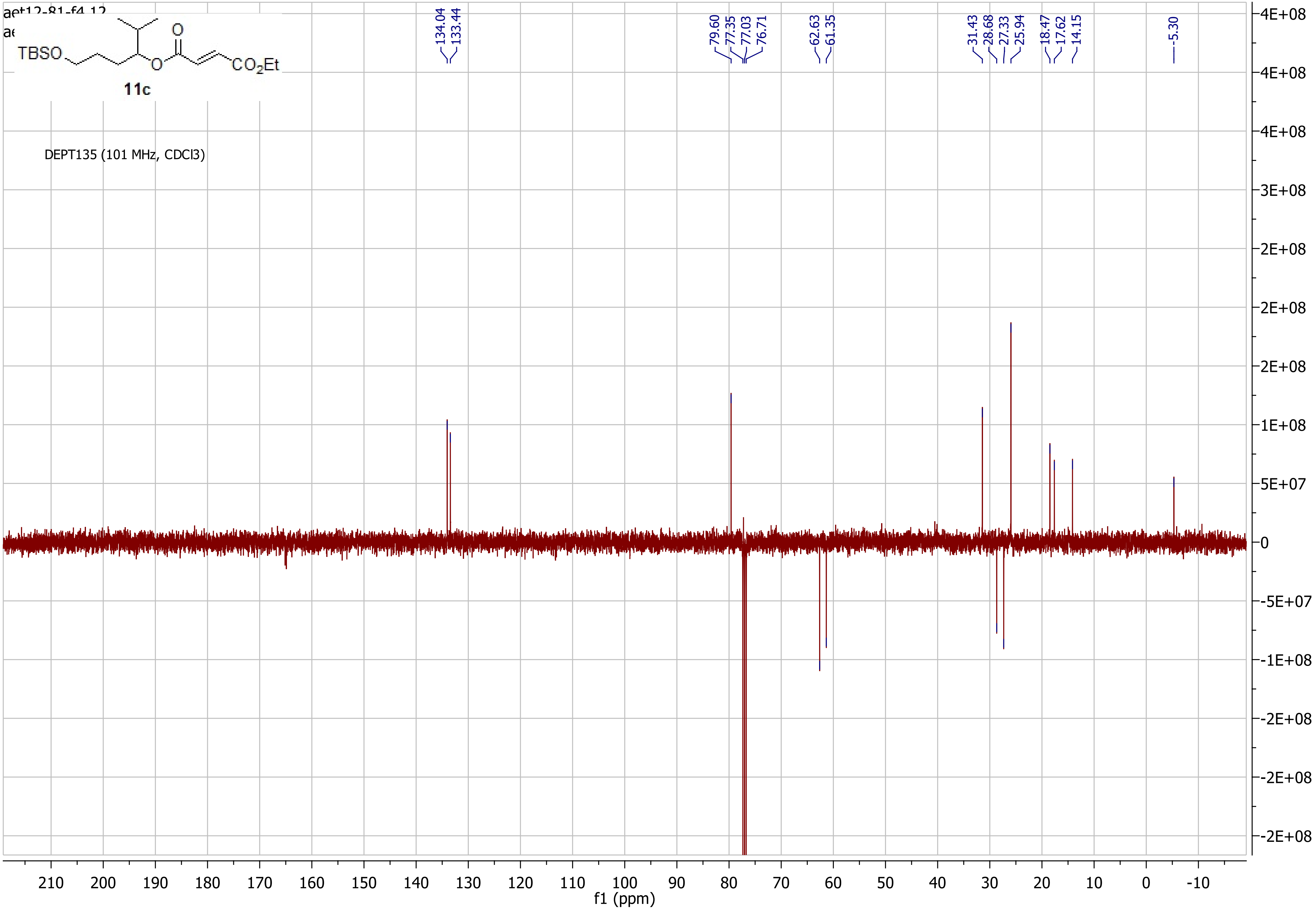
aet12-81-f4.12
TBSO
O
O
CO_2Et
11c
DEPT135 (101 MHz, CDCl3)
134.04
133.44
79.60
77.35
77.03
76.71
62.63
61.35
31.43
28.68
27.33
25.94
18.47
17.62
14.15
-5.30
f1 (ppm)

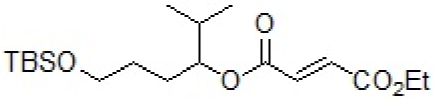

11c

COSY (400 MHz, CDCl3)

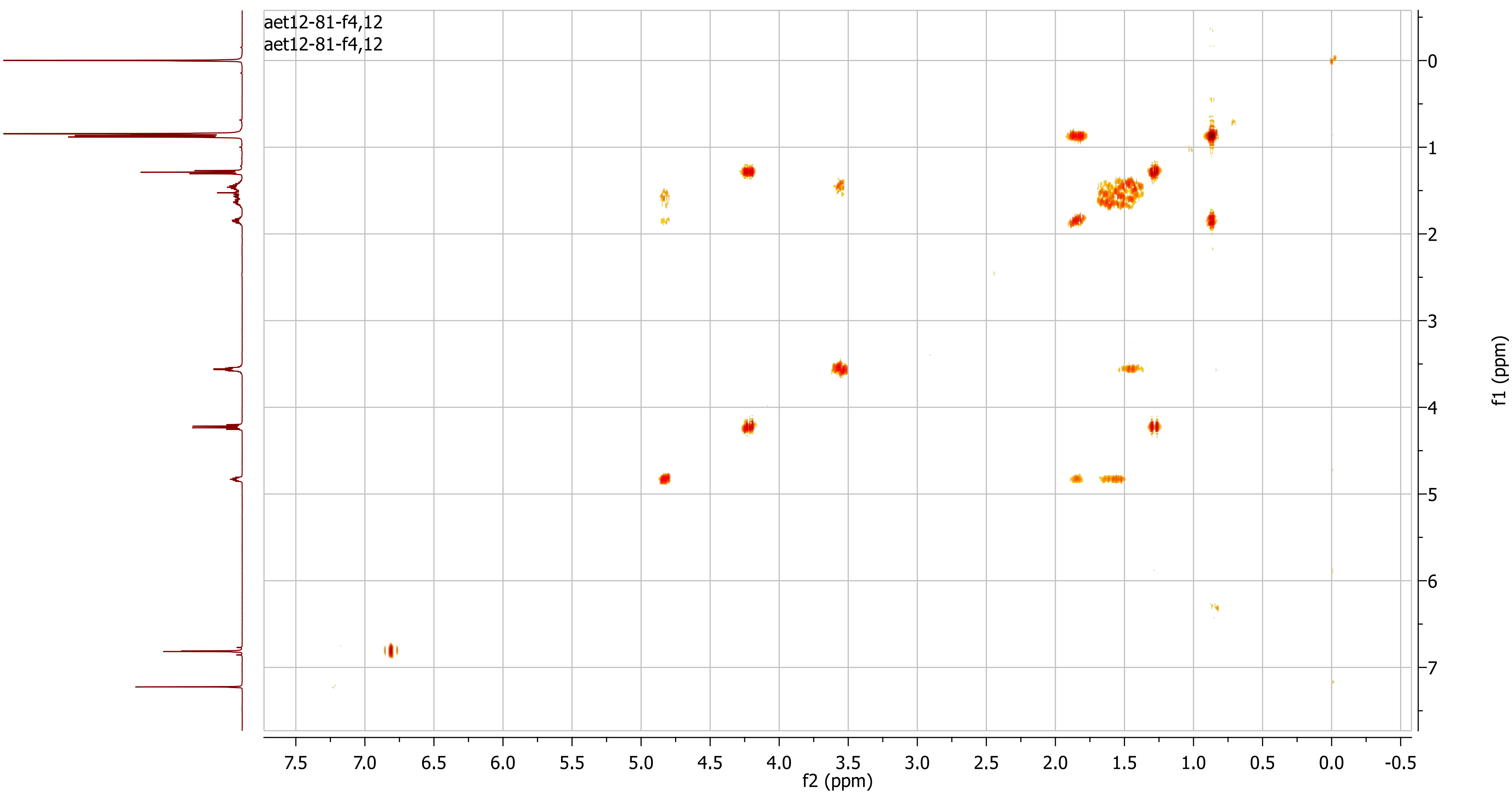

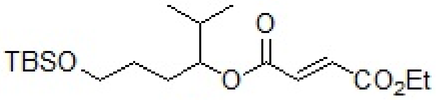

11c

HMQC (CDCl3)

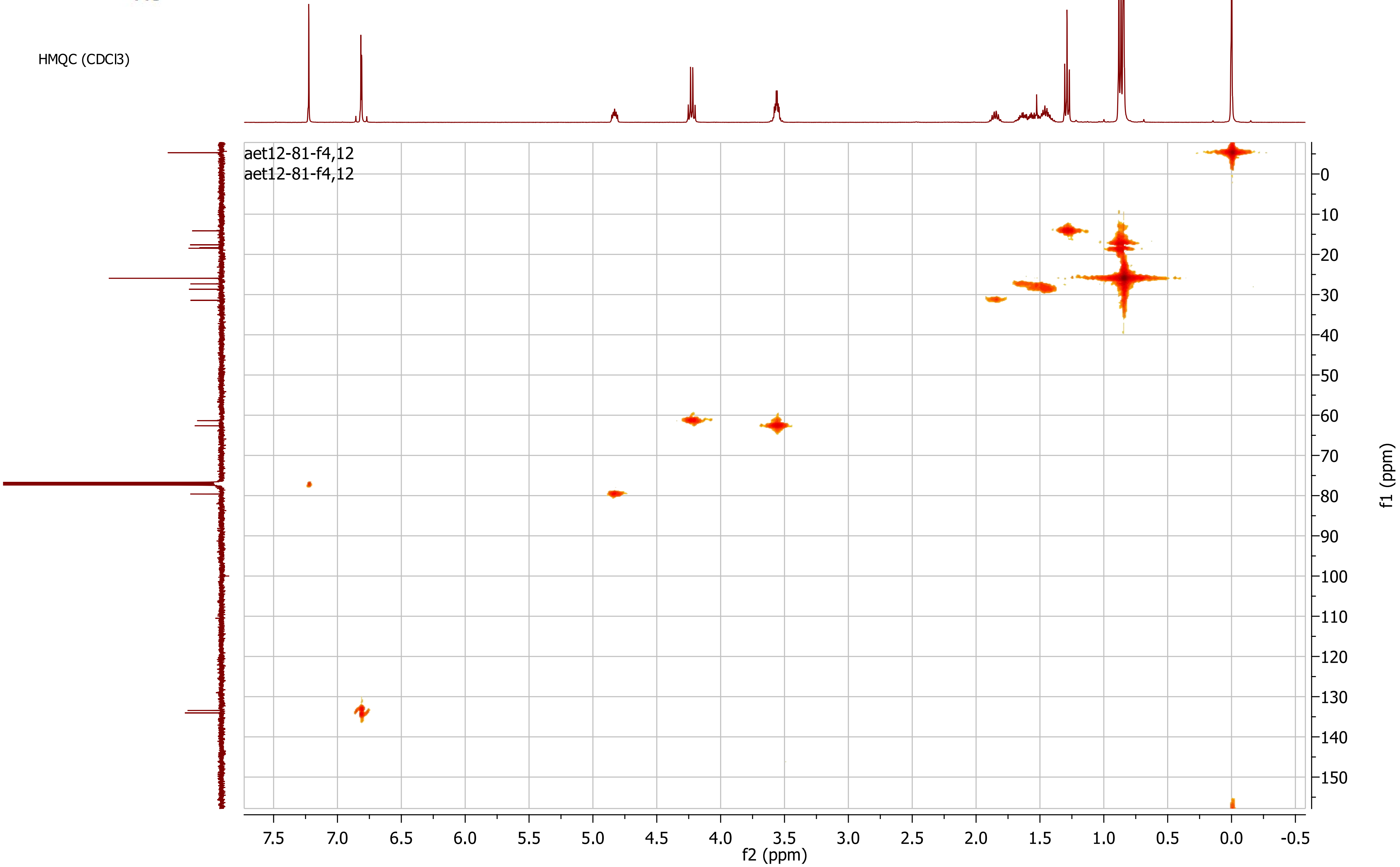

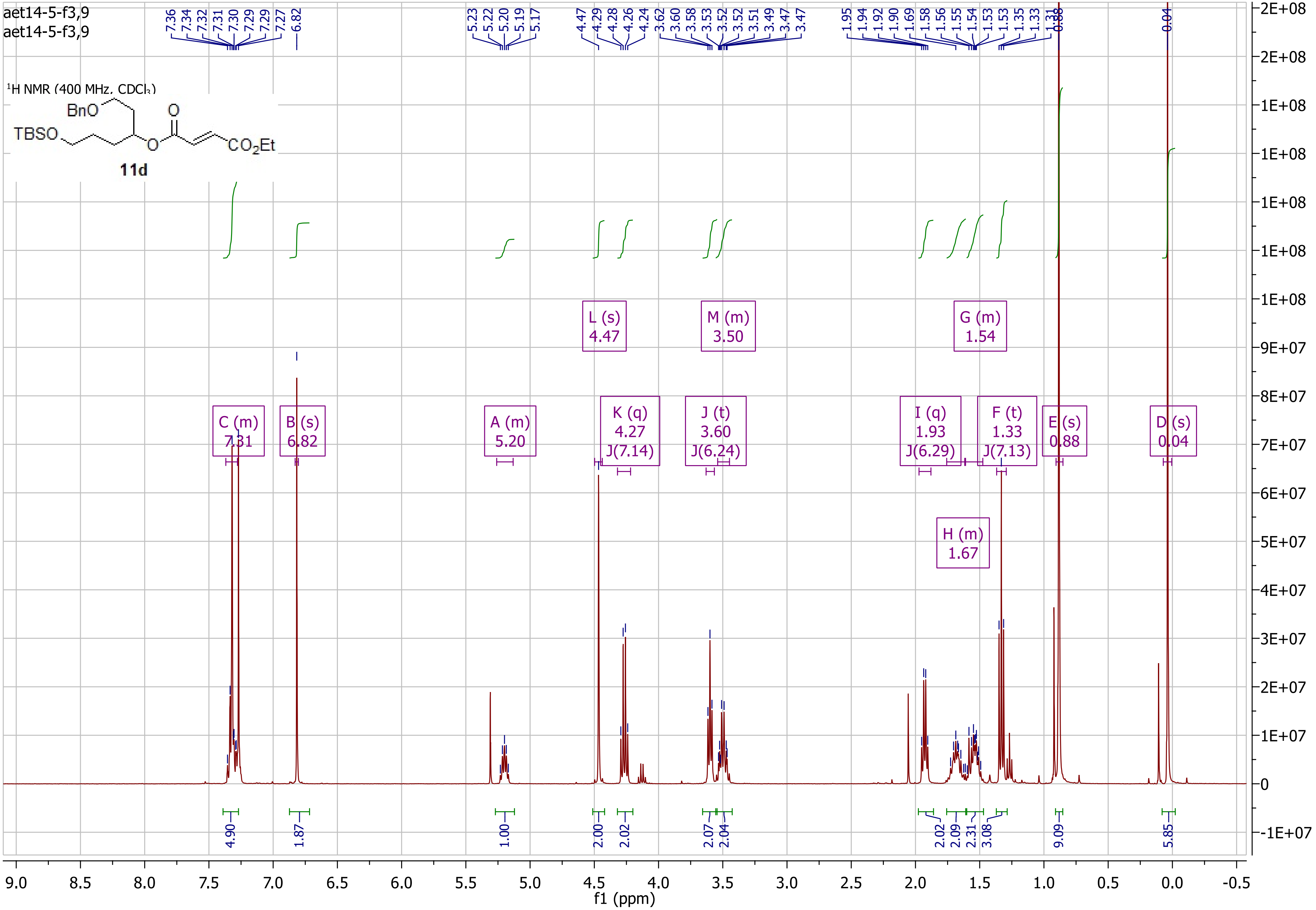
aet14-5-f3,9
aet14-5-f3,9
^{1}H NMR (400 MHz, CDCl$_3$)
BnO
TBSO
O
CO$_2$Et
11d
7.36 7.34 7.32 7.31 7.30 7.29 7.29 7.27 6.82
5.23 5.22 5.20 5.19 5.17
4.47 4.29 4.28 4.26 4.24 3.62 3.60 3.58 3.53 3.52 3.52 3.51 3.49 3.47 3.47
1.95 1.94 1.92 1.90 1.69 1.58 1.56 1.55 1.54 1.53 1.53 1.35 1.33 1.31 0.88
0.04
C (m) 7.31
B (s) 6.82
A (m) 5.20
L (s) 4.47
K (q) 4.27 J(7.14)
J (t) 3.60 J(6.24)
M (m) 3.50
I (q) 1.93 J(6.29)
H (m) 1.67
G (m) 1.54
F (t) 1.33 J(7.13)
E (s) 0.88
D (s) 0.04
4.90 1.87 1.00 2.00 2.02 2.07 2.04 2.02 2.09 2.31 3.08 9.09 5.85
f1 (ppm)

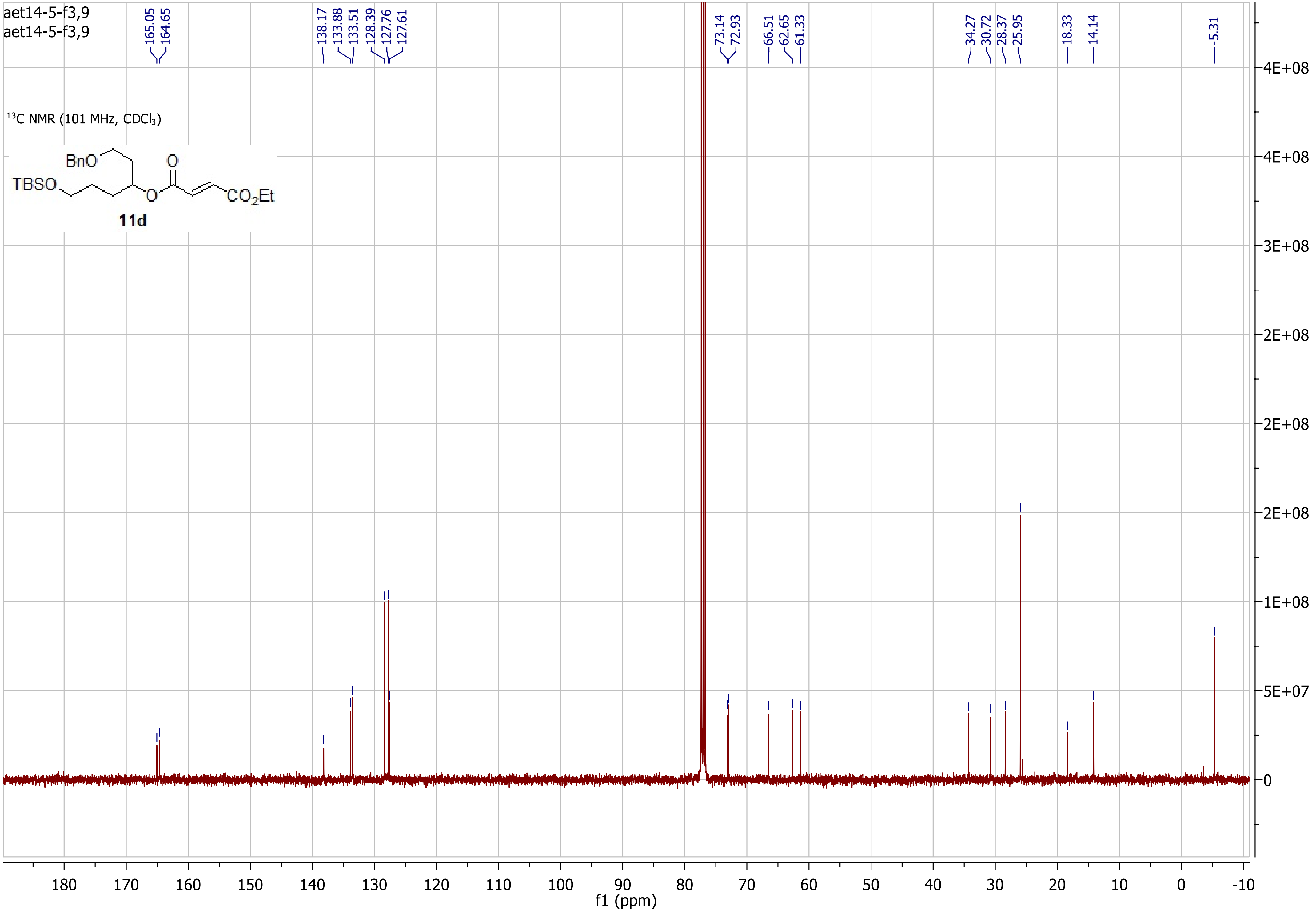

aet14-5-f3,9
aet14-5-f3,9
165.05
164.65
138.17
133.88
133.51
128.39
127.76
127.61
73.14
72.93
66.51
62.65
61.33
34.27
30.72
28.37
25.95
18.33
14.14
-5.31
13C NMR (101 MHz, CDCl3)
BnO
TBSO
O
O
CO2Et
11d
4E+08
4E+08
3E+08
2E+08
2E+08
2E+08
1E+08
5E+07
0
180
170
160
150
140
130
120
110
100
90
80
70
60
50
40
30
20
10
0
-10
f1 (ppm)

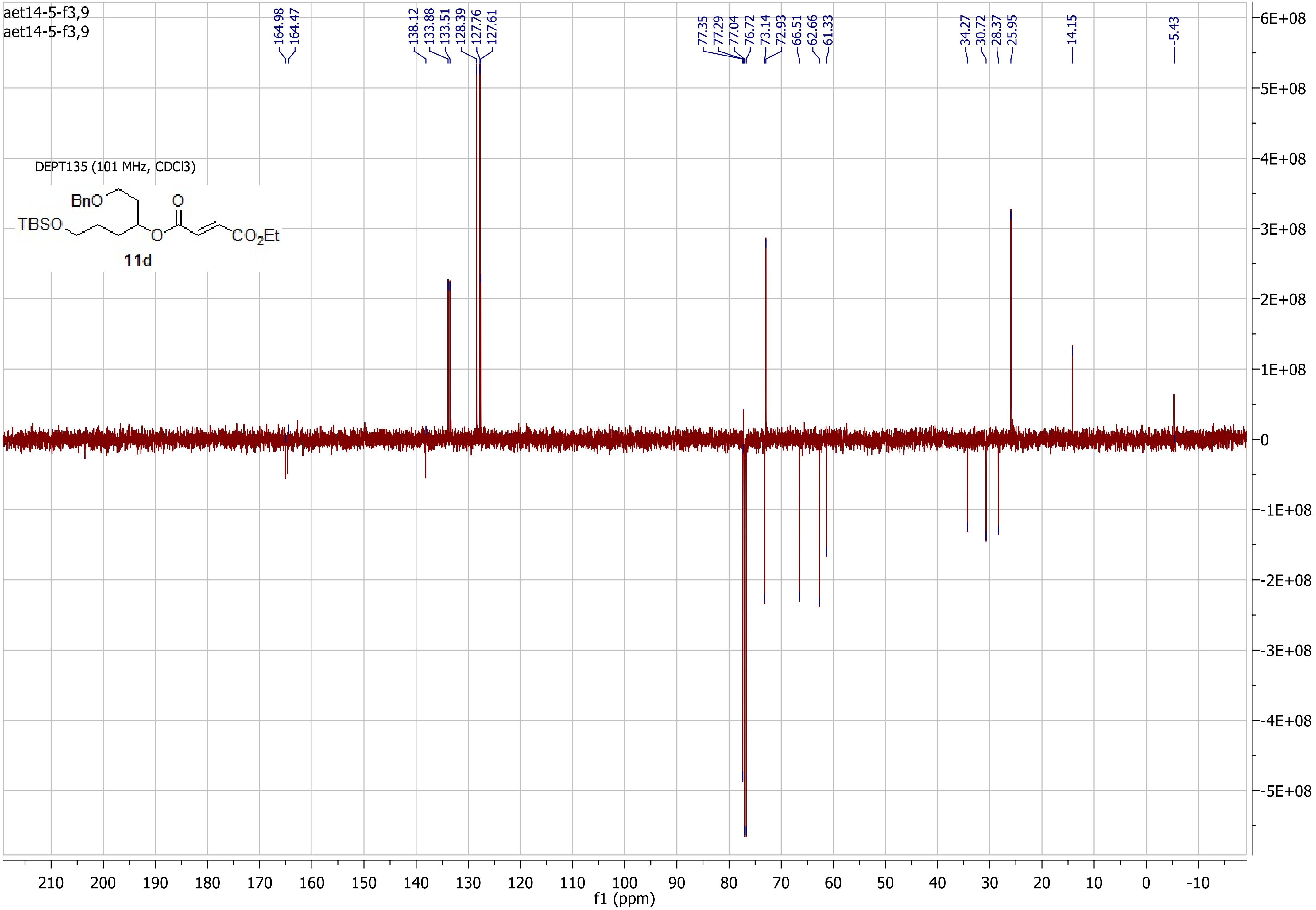

aet14-5-f3,9
aet14-5-f3,9
DEPT135 (101 MHz, CDCl3)
BnO
TBSO
O
O
CO2Et
11d
164.98
164.47
138.12
133.88
133.51
128.39
127.76
127.61
77.35
77.29
77.04
76.72
73.14
72.93
66.51
62.66
61.33
34.27
30.72
28.37
25.95
14.15
-5.43
f1 (ppm)

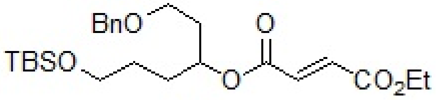

11d

COSY (400 MHz, CDCl3)

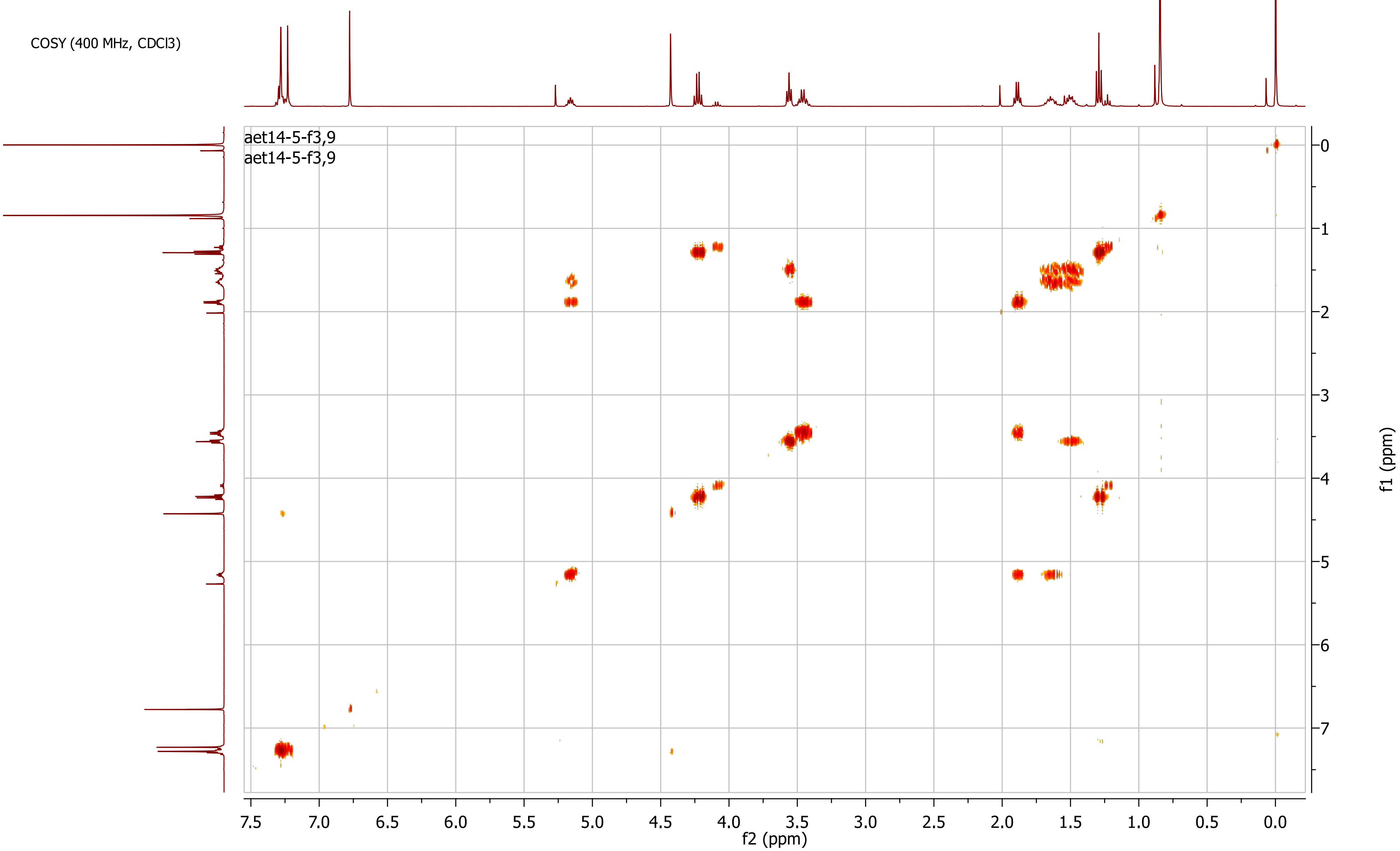

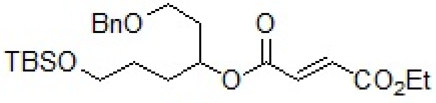

11d

HMQC (CDCl3)

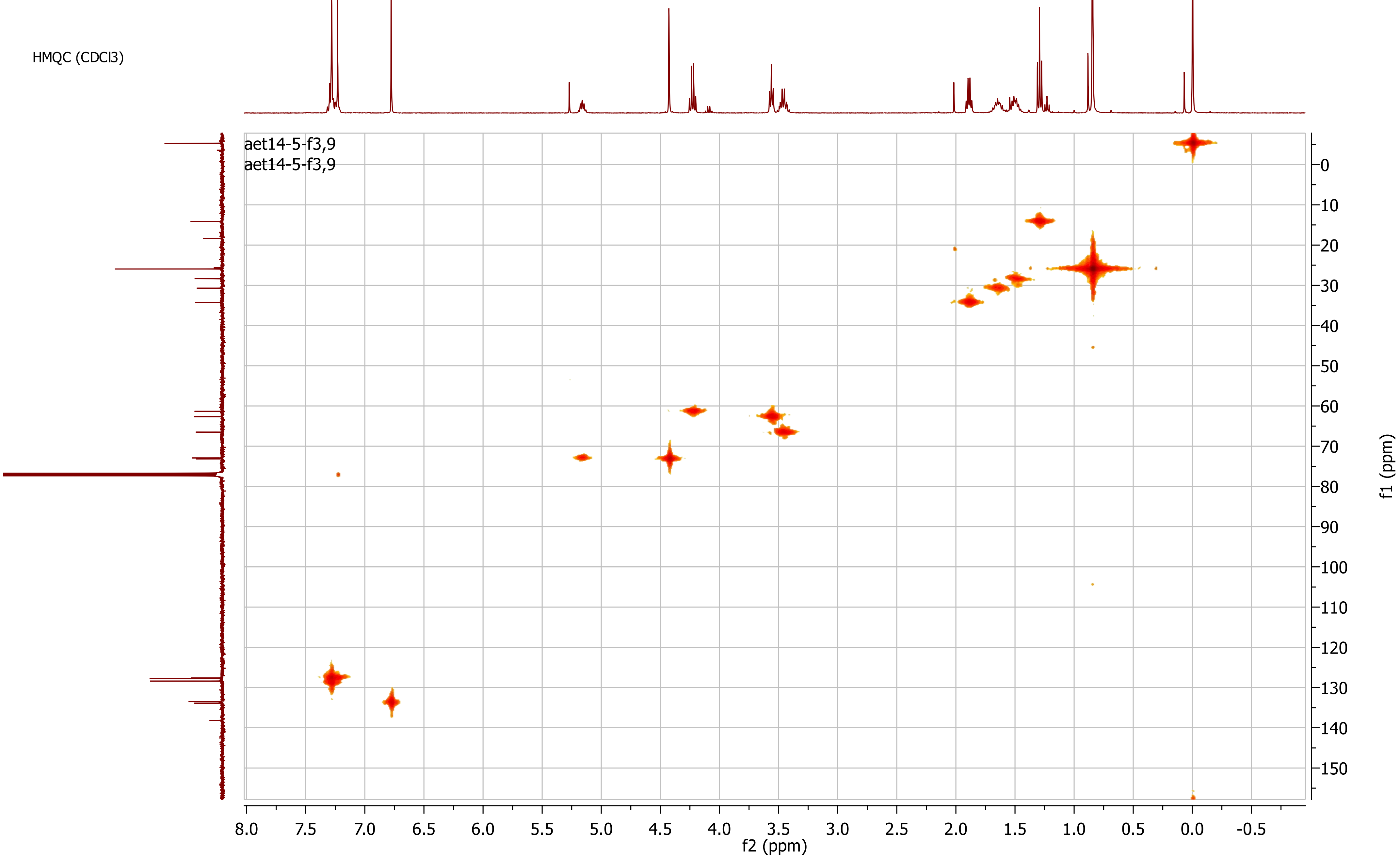

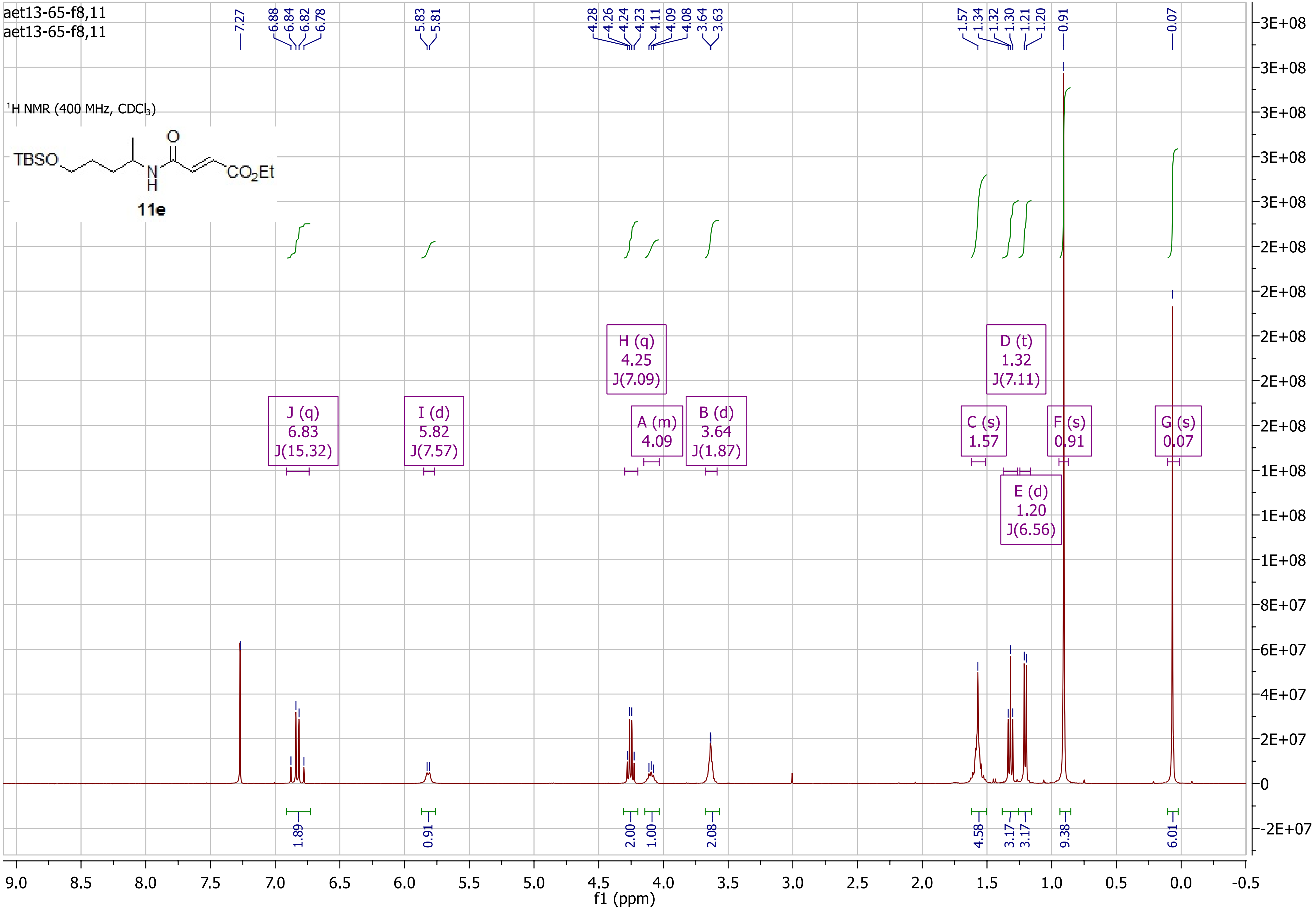
aet13-65-f8,11
aet13-65-f8,11
1H NMR (400 MHz, CDCl3)
TBSO
O
N
H
CO2Et
11e
7.27
6.88
6.84
6.82
6.78
5.83
5.81
4.28
4.26
4.24
4.23
4.11
4.09
4.08
3.64
3.63
1.57
1.34
1.32
1.30
1.21
1.20
0.91
0.07
H (q)
4.25
J(7.09)
D (t)
1.32
J(7.11)
J (q)
6.83
J(15.32)
I (d)
5.82
J(7.57)
A (m)
4.09
B (d)
3.64
J(1.87)
C (s)
1.57
F (s)
0.91
G (s)
0.07
E (d)
1.20
J(6.56)
1.89
0.91
2.00
1.00
2.08
4.58
3.17
3.17
9.38
6.01
3E+08
3E+08
3E+08
3E+08
3E+08
2E+08
2E+08
2E+08
2E+08
2E+08
1E+08
1E+08
1E+08
8E+07
6E+07
4E+07
2E+07
0
-2E+07
9.0
8.5
8.0
7.5
7.0
6.5
6.0
5.5
5.0
4.5
4.0
3.5
3.0
2.5
2.0
1.5
1.0
0.5
0.0
-0.5
f1 (ppm)

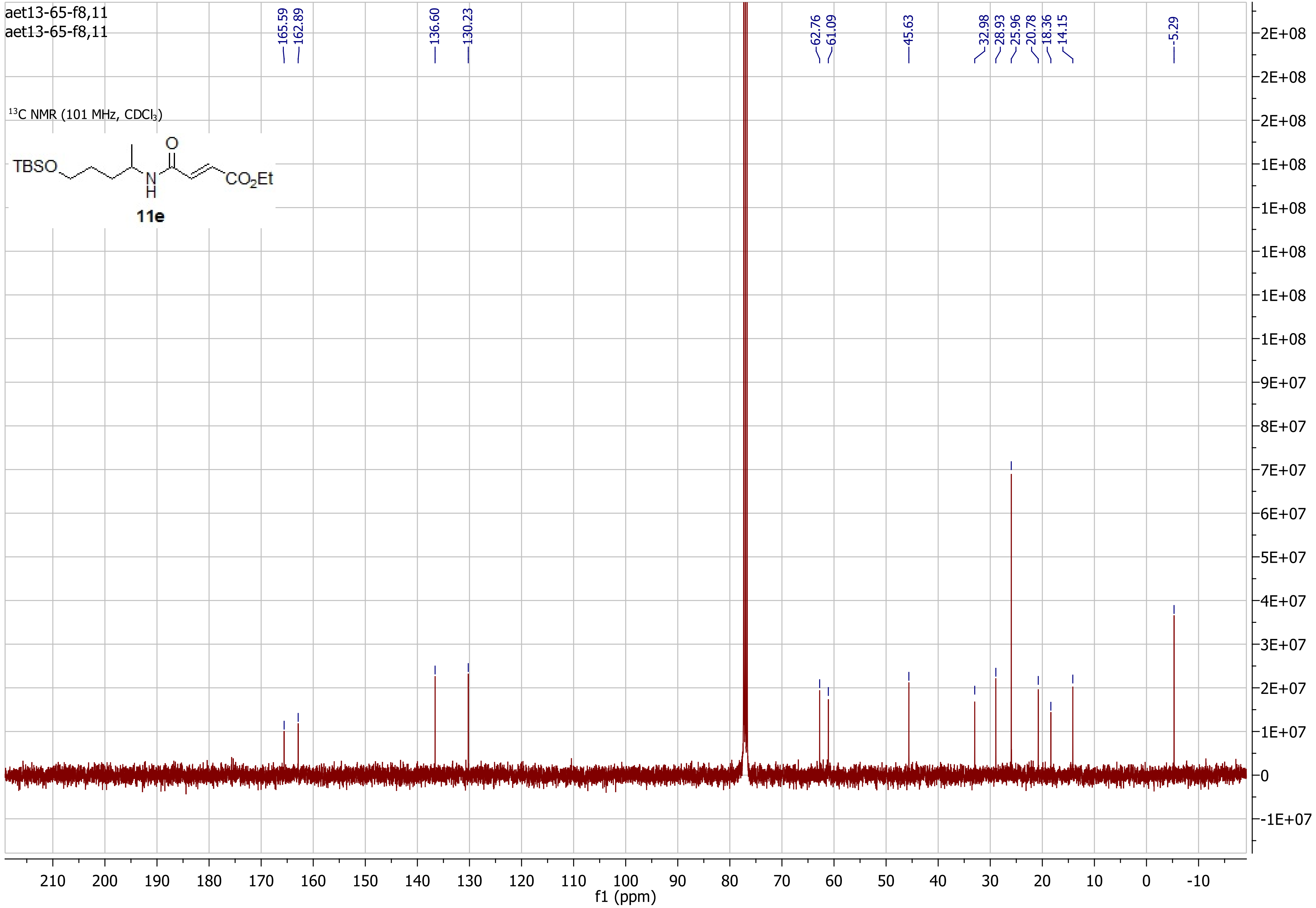
aet13-65-f8,11
aet13-65-f8,11
¹³C NMR (101 MHz, CDCl₃)
TBSO
O
N
H
CO₂Et
11e
165.59
162.89
136.60
130.23
62.76
61.09
45.63
32.98
28.93
25.96
20.78
18.36
14.15
-5.29
f1 (ppm)

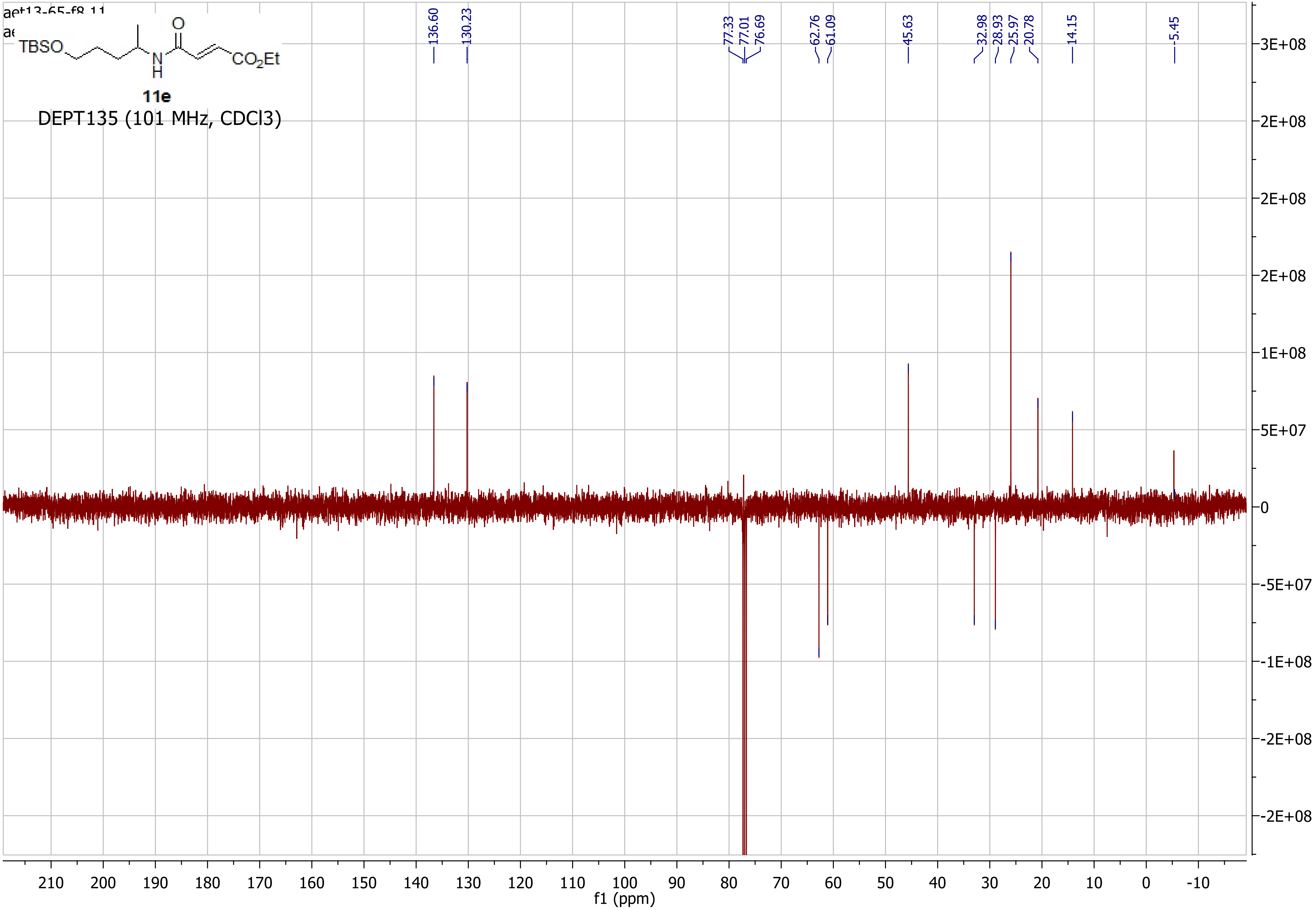
TBSO
O
N
H
CO2Et
11e
DEPT135 (101 MHz, CDCl3)
136.60
130.23
77.33
77.01
76.69
62.76
61.09
45.63
32.98
28.93
25.97
20.78
14.15
-5.45
3E+08
2E+08
2E+08
2E+08
1E+08
5E+07
0
-5E+07
-1E+08
-2E+08
-2E+08
210
200
190
180
170
160
150
140
130
120
110
100
90
80
70
60
50
40
30
20
10
0
-10
f1 (ppm)

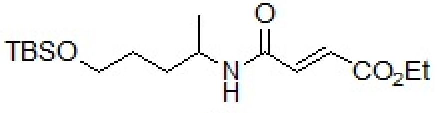

11e

COSY (400 MHz, CDCl3)

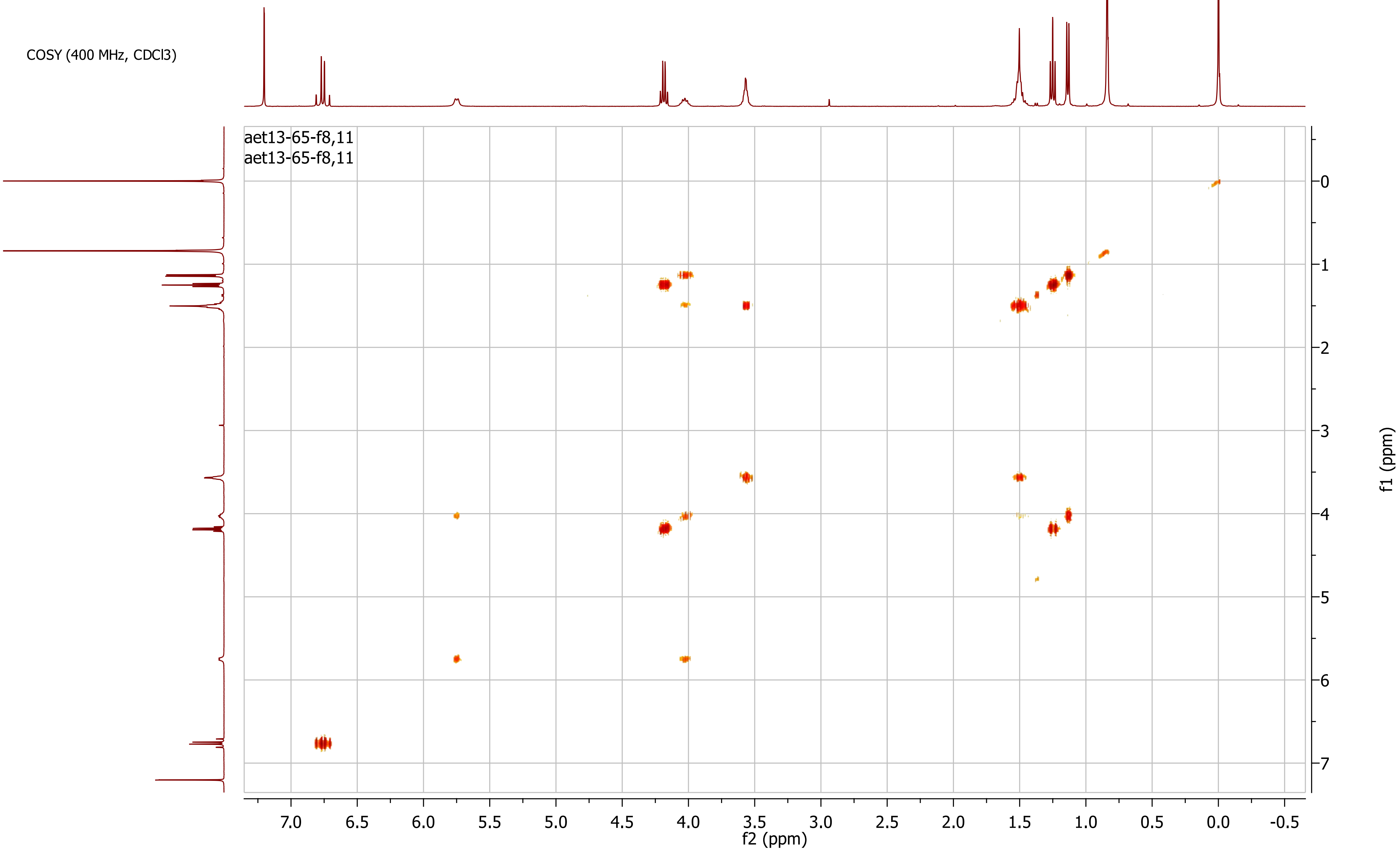

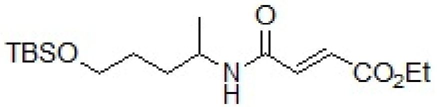

11e

HMQC (CDCL3)

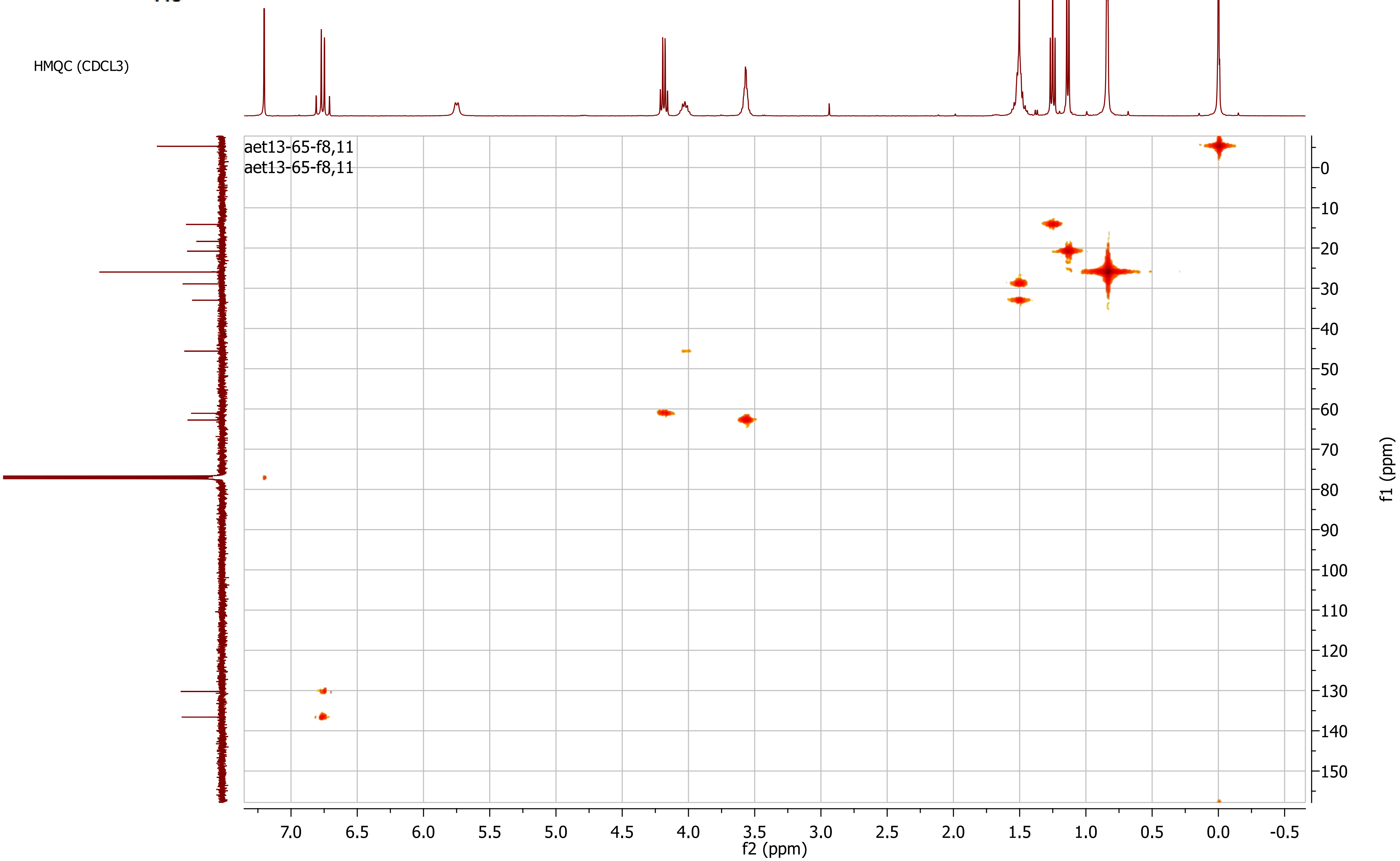

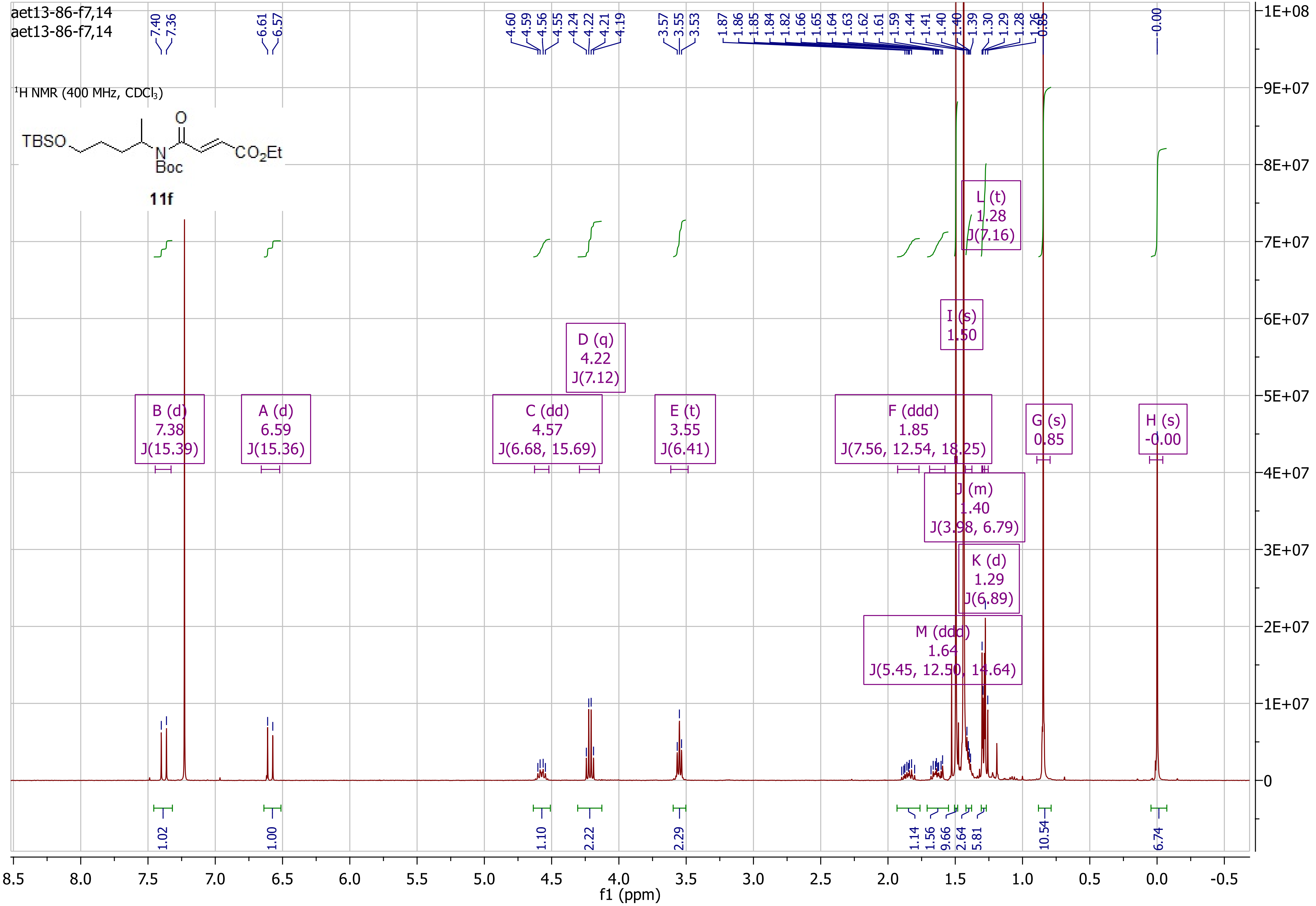

aet13-86-f7,14
aet13-86-f7,14
¹H NMR (400 MHz, CDCl3)
TBSO
O
N
Boc
CO2Et
11f
7.40
7.36
6.61
6.57
4.60
4.59
4.56
4.55
4.24
4.22
4.21
4.19
3.57
3.55
3.53
1.87
1.86
1.85
1.84
1.82
1.66
1.65
1.64
1.63
1.62
1.61
1.59
1.44
1.41
1.40
1.40
1.39
1.30
1.29
1.28
1.26
0.85
-0.00
B (d) 7.38 J(15.39)
A (d) 6.59 J(15.36)
C (dd) 4.57 J(6.68, 15.69)
D (q) 4.22 J(7.12)
E (t) 3.55 J(6.41)
F (ddd) 1.85 J(7.56, 12.54, 18.25)
M (ddd) 1.64 J(5.45, 12.50, 14.64)
I (s) 1.50
J (m) 1.40 J(3.98, 6.79)
K (d) 1.29 J(6.89)
L (t) 1.28 J(7.16)
G (s) 0.85
H (s) -0.00
1.02
1.00
1.10
2.22
2.29
1.14
1.56
9.66
2.64
5.81
10.54
6.74
1E+08
9E+07
8E+07
7E+07
6E+07
5E+07
4E+07
3E+07
2E+07
1E+07
0
8.5
8.0
7.5
7.0
6.5
6.0
5.5
5.0
4.5
4.0
3.5
3.0
2.5
2.0
1.5
1.0
0.5
0.0
-0.5
f1 (ppm)

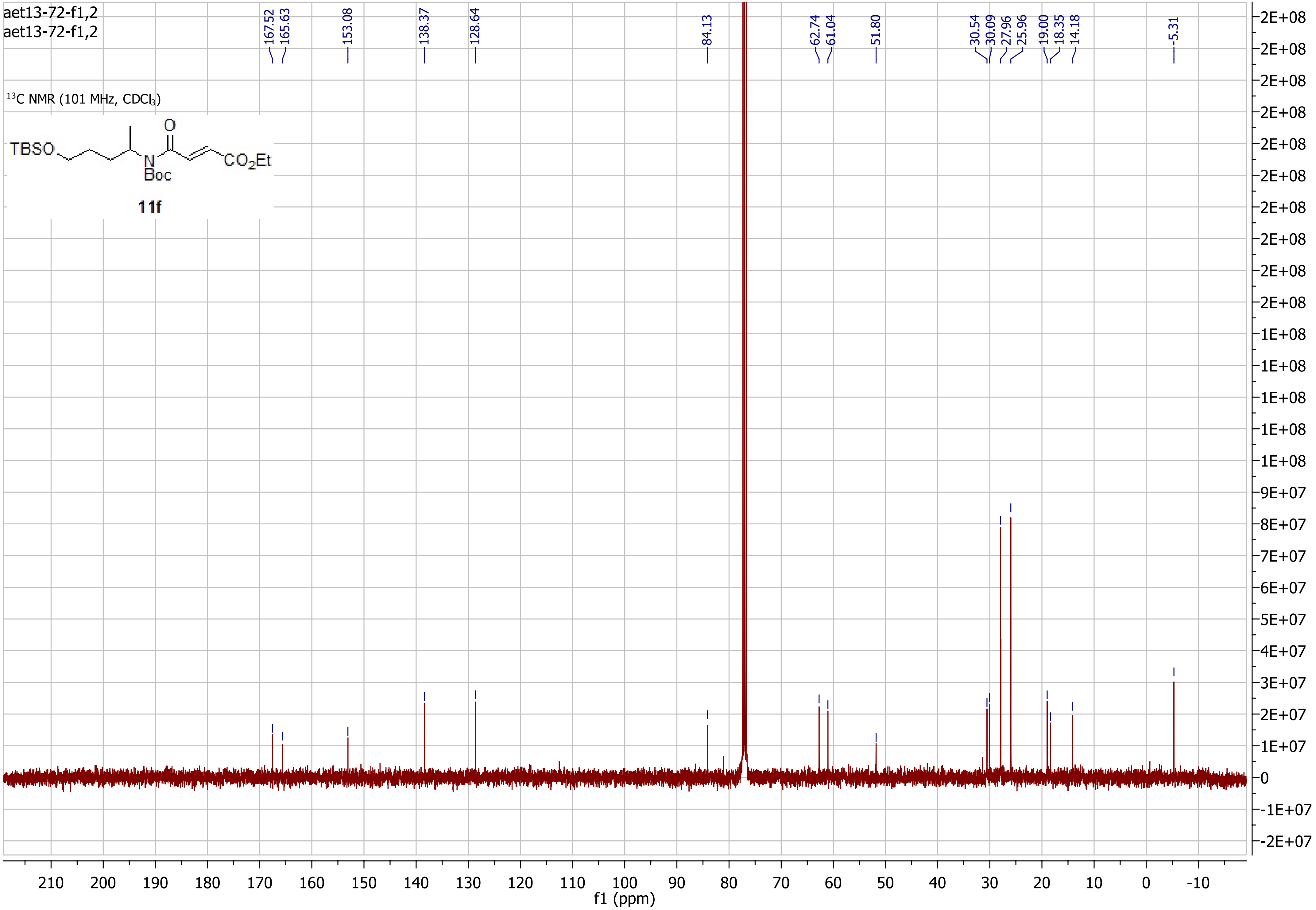
aet13-72-f1,2
aet13-72-f1,2
13C NMR (101 MHz, CDCl3)
TBSO
N
Boc
O
CO2Et
11f
167.52
165.63
153.08
138.37
128.64
84.13
62.74
61.04
51.80
30.54
30.09
27.96
25.96
19.00
18.35
14.18
-5.31
f1 (ppm)

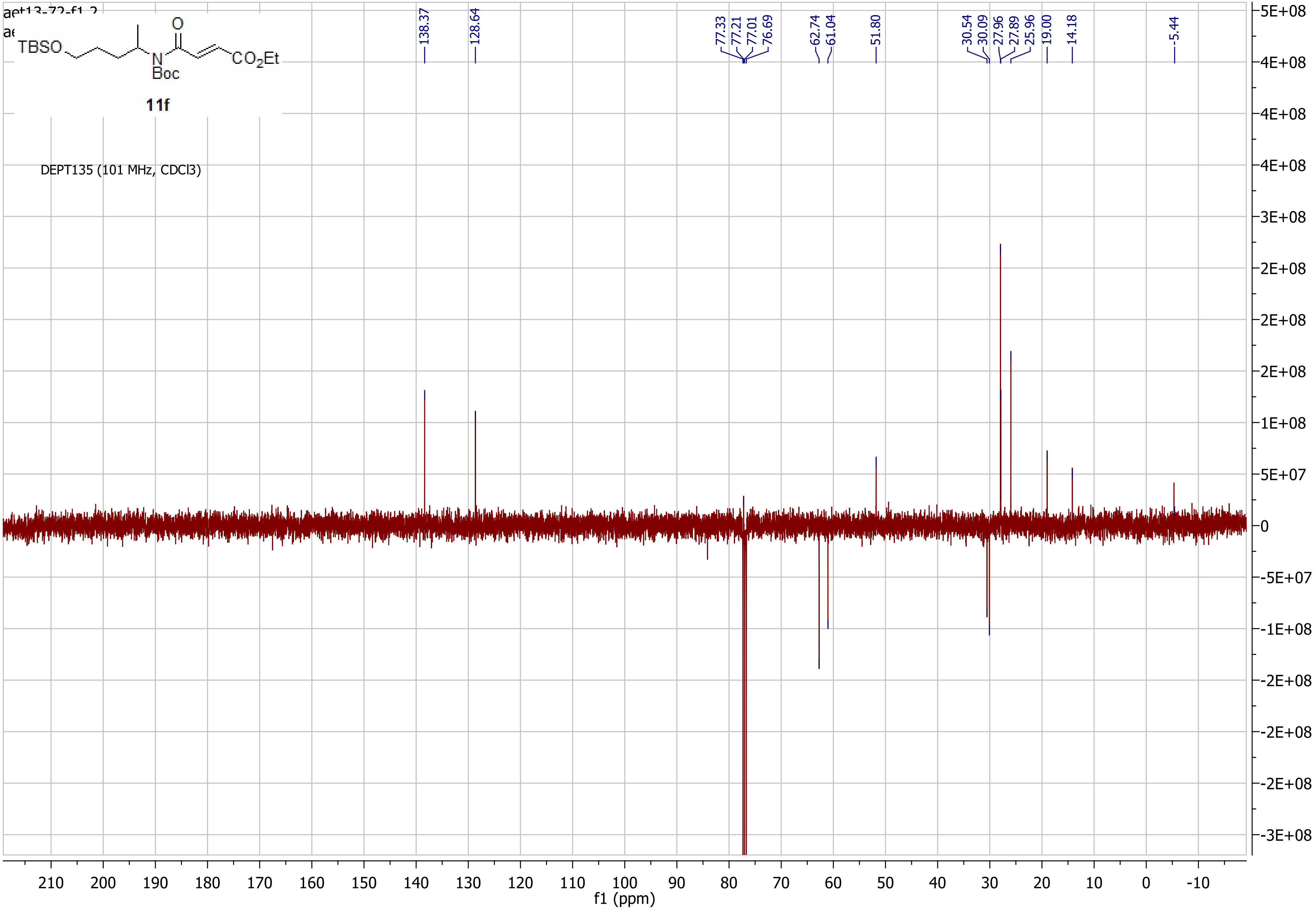

aet13-72-f1.2
TBSO
N
Boc
O
CO2Et
11f
DEPT135 (101 MHz, CDCl3)
138.37
128.64
77.33
77.21
77.01
76.69
62.74
61.04
51.80
30.54
30.09
27.96
27.89
25.96
19.00
14.18
-5.44
f1 (ppm)

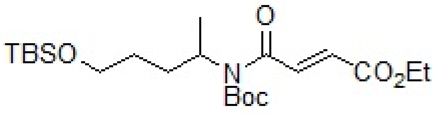

11f

COSY (400 MHz, CDCl3)

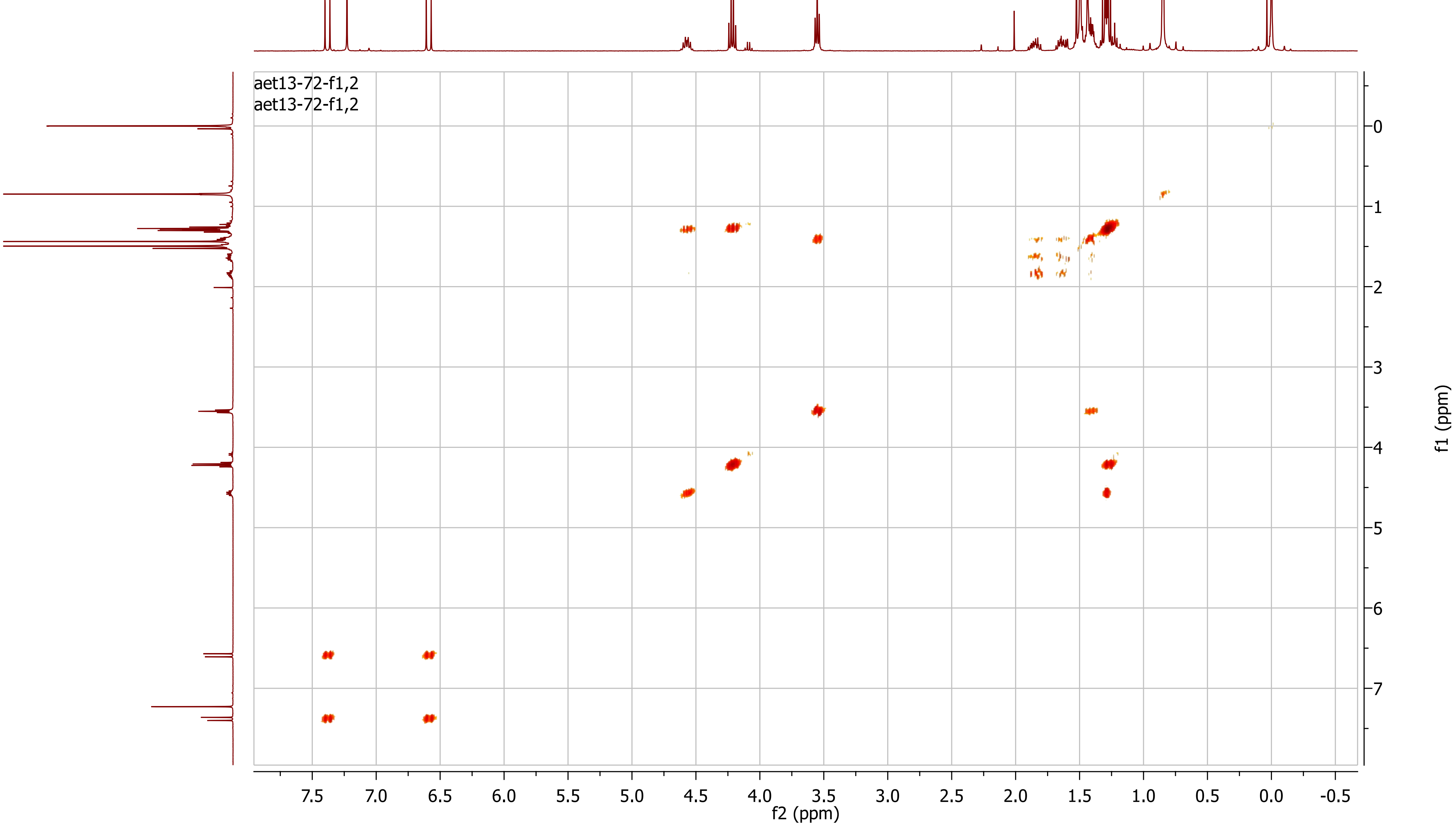

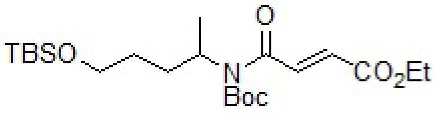

11f

HMQC (CDCl3)

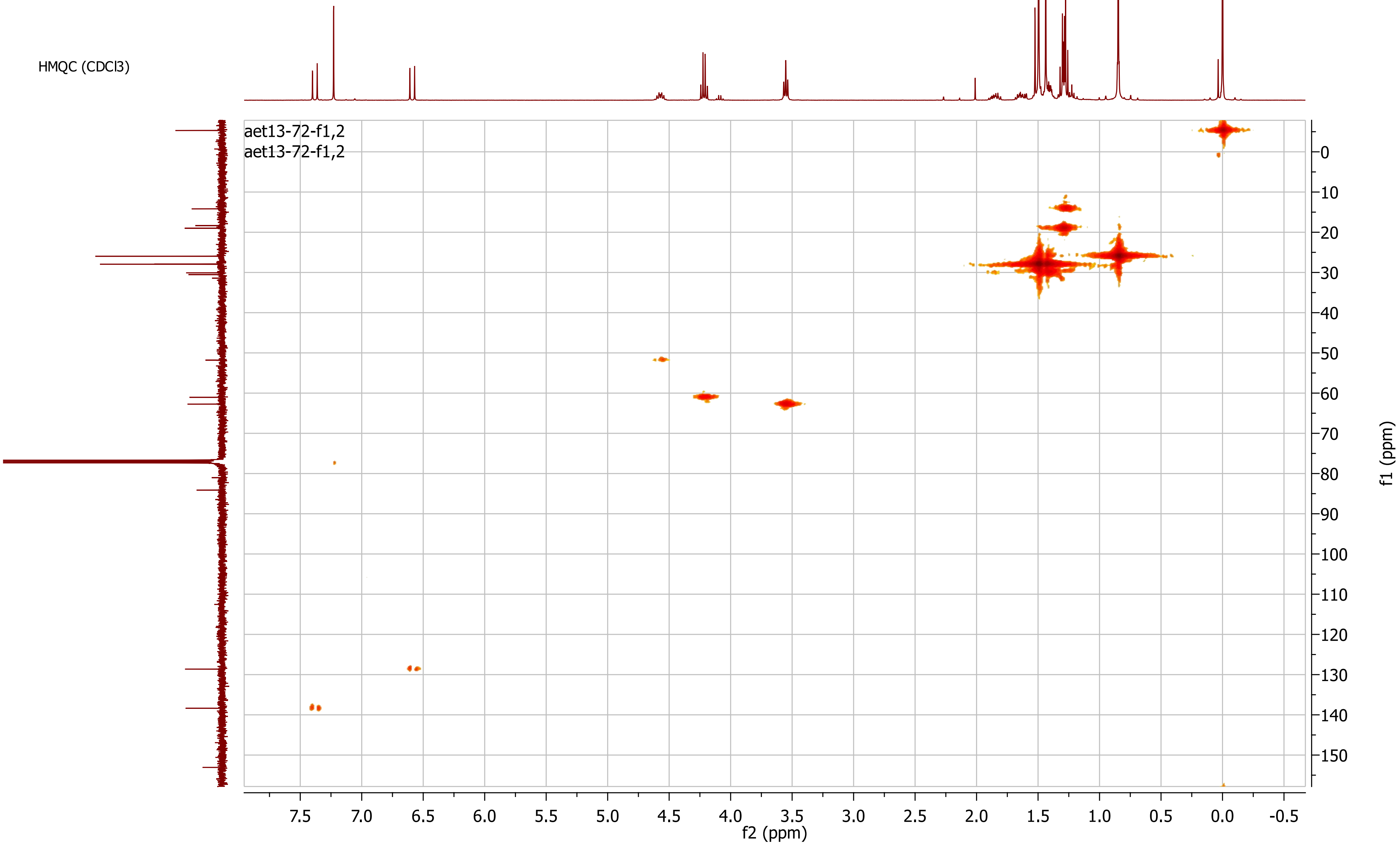

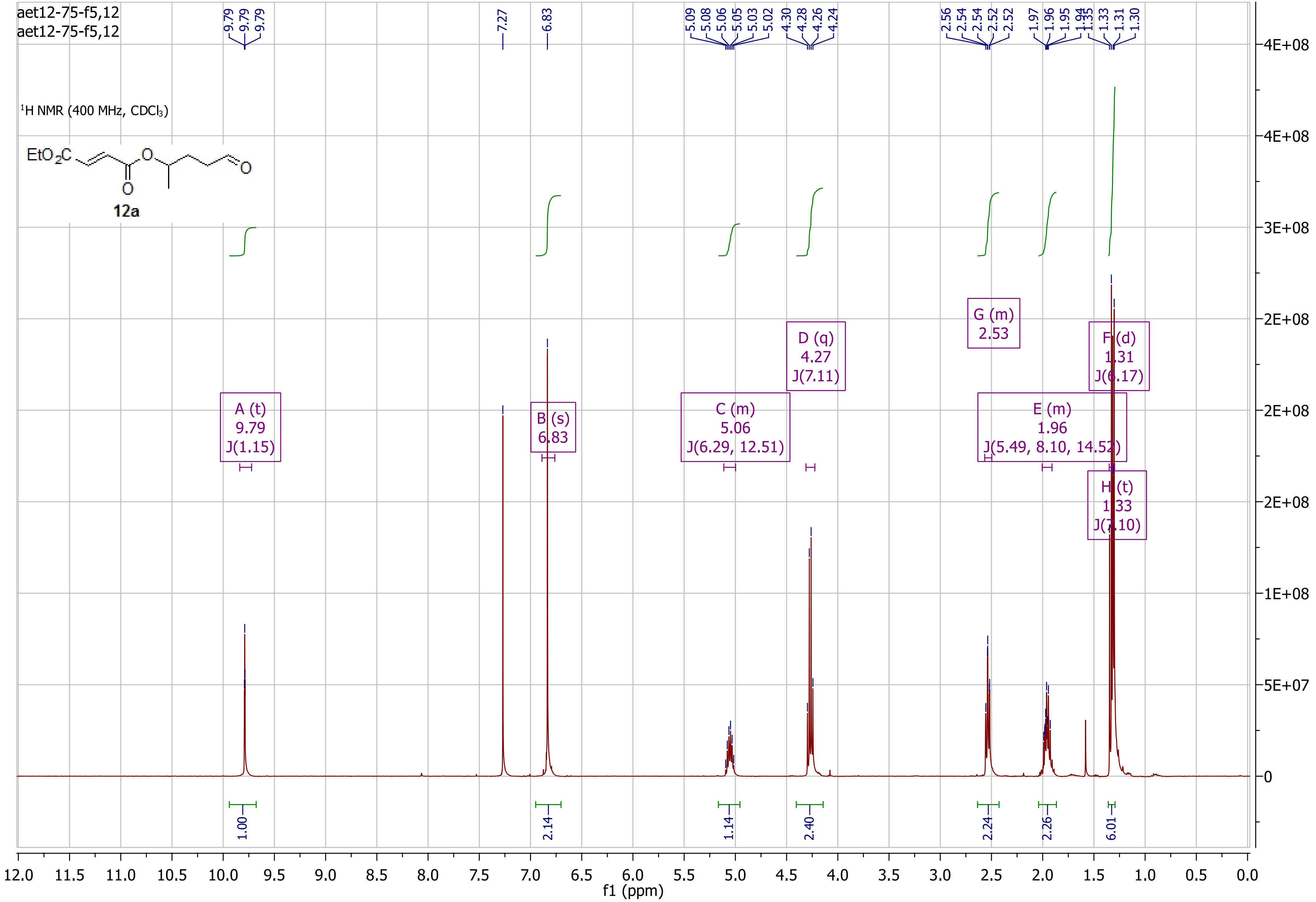
aet12-75-f5,12
aet12-75-f5,12
1H NMR (400 MHz, CDCl3)
EtO2C
O
O
O
O
12a
9.79 9.79 9.79
7.27
6.83
5.09 5.08 5.06 5.05 5.03 5.02 4.30 4.28 4.26 4.24
2.56 2.54 2.54 2.52 2.52
1.97 1.96 1.95 1.94 1.35 1.33 1.31 1.30
A (t) 9.79 J(1.15)
B (s) 6.83
C (m) 5.06 J(6.29, 12.51)
D (q) 4.27 J(7.11)
G (m) 2.53
E (m) 1.96 J(5.49, 8.10, 14.52)
F (d) 1.31 J(6.17)
H (t) 1.33 J(7.10)
1.00
2.14
1.14
2.40
2.24
2.26
6.01
4E+08
4E+08
3E+08
2E+08
2E+08
2E+08
1E+08
5E+07
0
12.0 11.5 11.0 10.5 10.0 9.5 9.0 8.5 8.0 7.5 7.0 6.5 6.0 5.5 5.0 4.5 4.0 3.5 3.0 2.5 2.0 1.5 1.0 0.5 0.0
f1 (ppm)

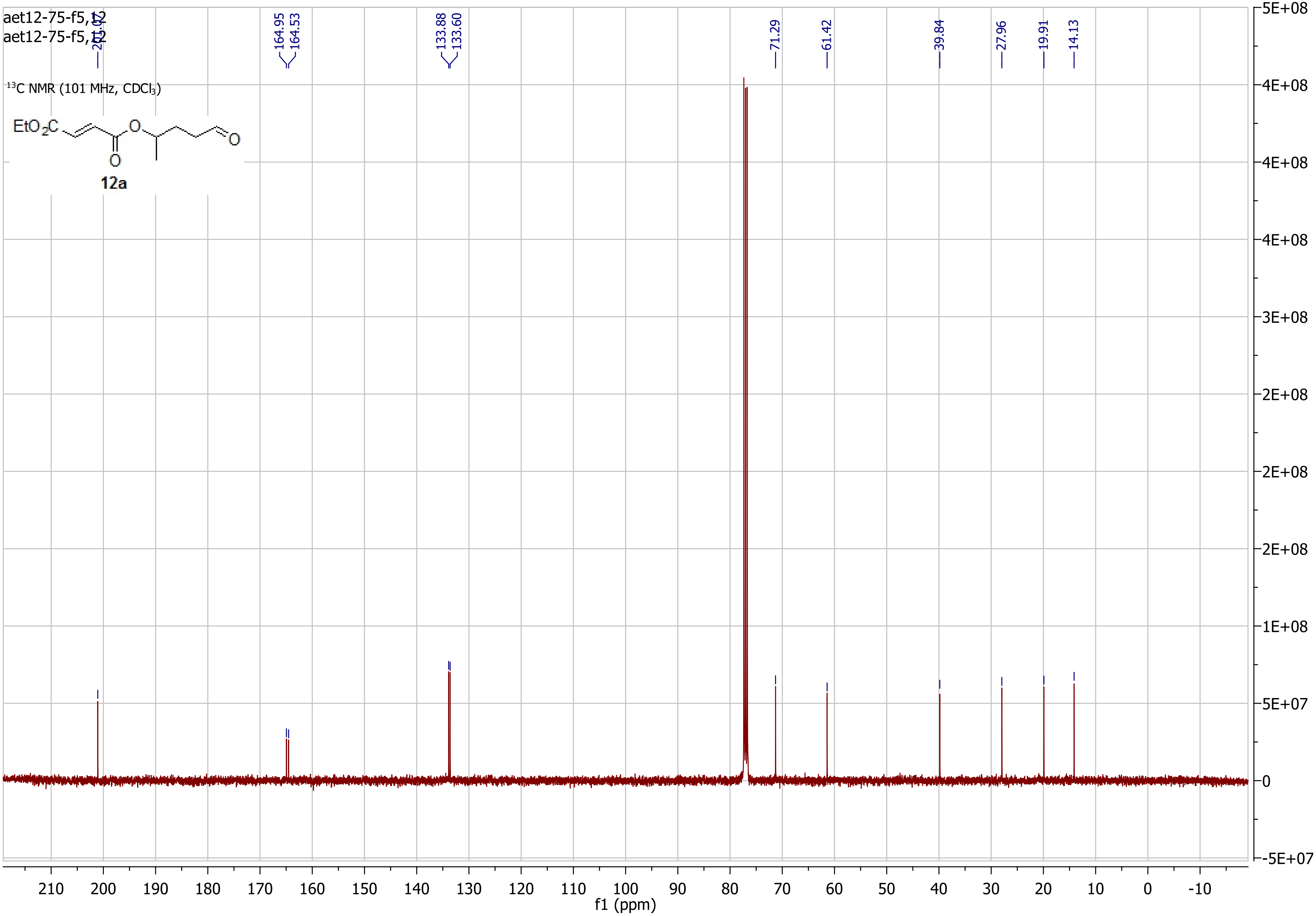

aet12-75-f5,12
aet12-75-f5,12
164.95
164.53
133.88
133.60
71.29
61.42
39.84
27.96
19.91
14.13
13C NMR (101 MHz, CDCl3)
EtO2C
O
O
O
12a
f1 (ppm)
210 200 190 180 170 160 150 140 130 120 110 100 90 80 70 60 50 40 30 20 10 0 -10
5E+08 4E+08 4E+08 4E+08 3E+08 2E+08 2E+08 2E+08 1E+08 5E+07 0 -5E+07

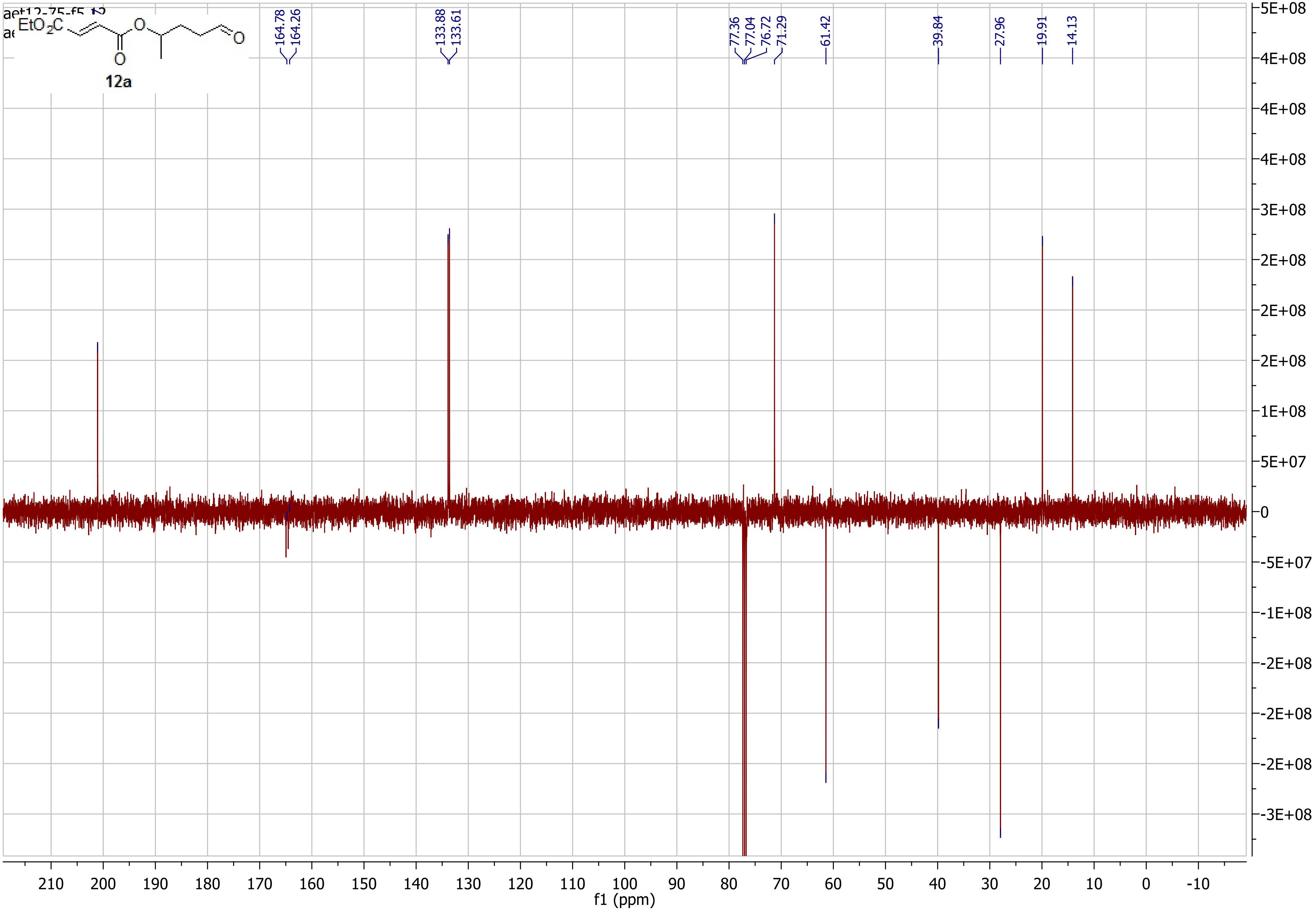

EtO_2C
O
O
O
12a
164.78
164.26
133.88
133.61
77.36
77.04
76.72
71.29
61.42
39.84
27.96
19.91
14.13
5E+08
4E+08
3E+08
2E+08
1E+08
5E+07
0
-5E+07
-1E+08
-2E+08
-3E+08
210 200 190 180 170 160 150 140 130 120 110 100 90 80 70 60 50 40 30 20 10 0 -10
f1 (ppm)

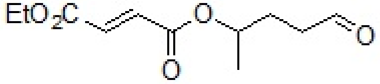

12a

COSY (400 MHz, CDCl3)

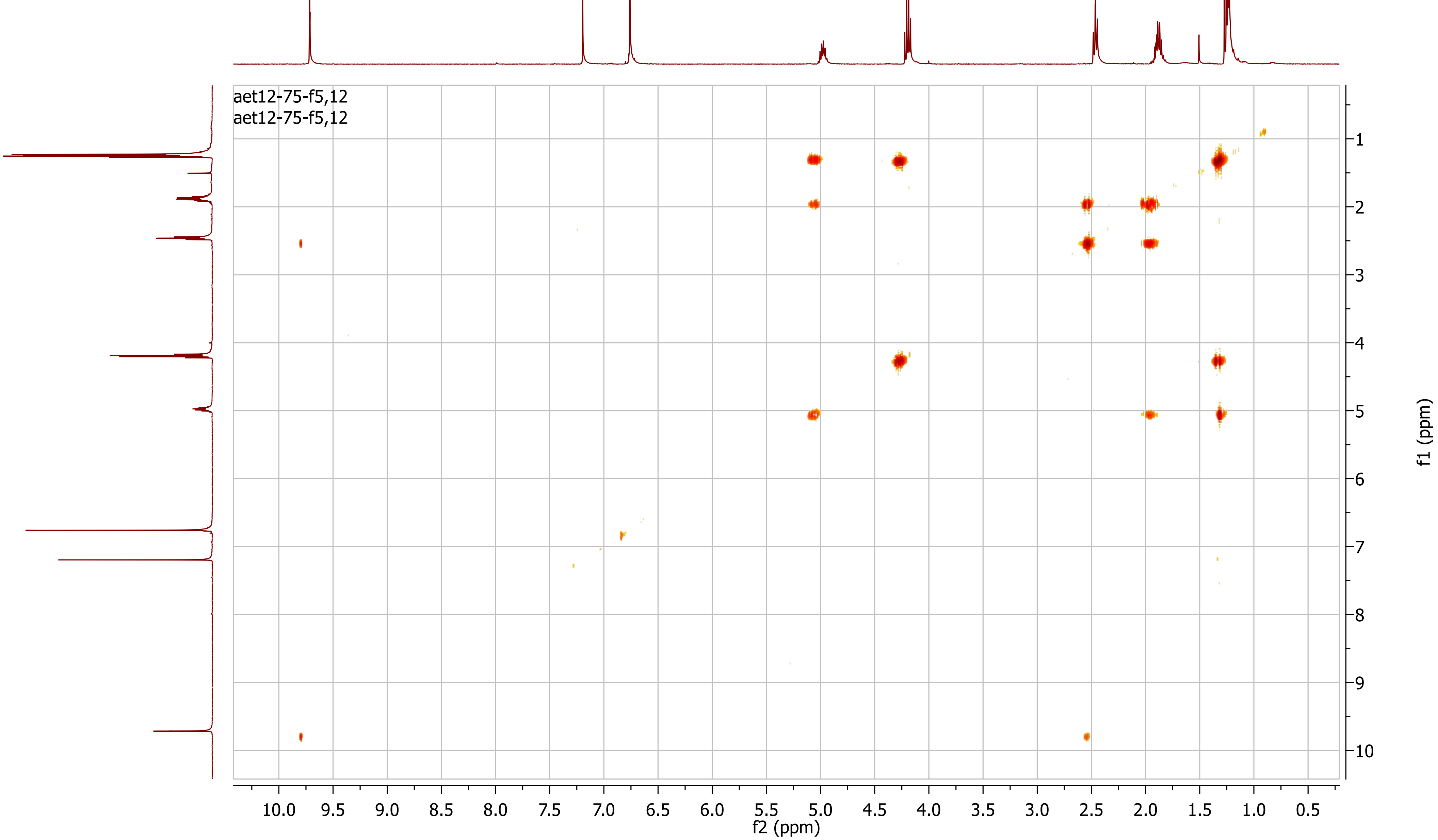

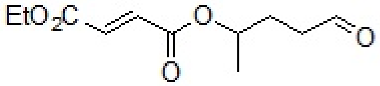

12a

HMQC (CDCl3)

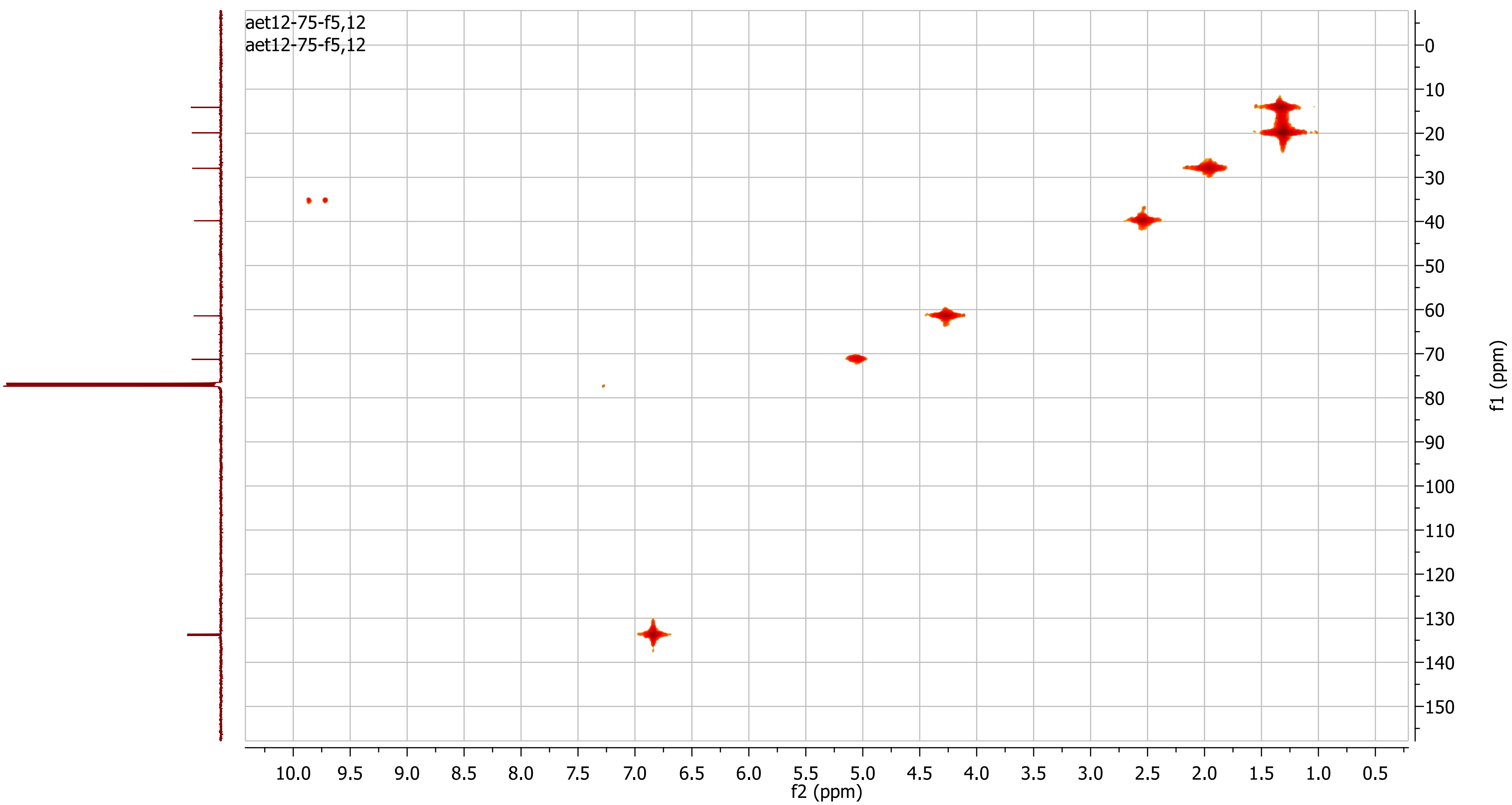

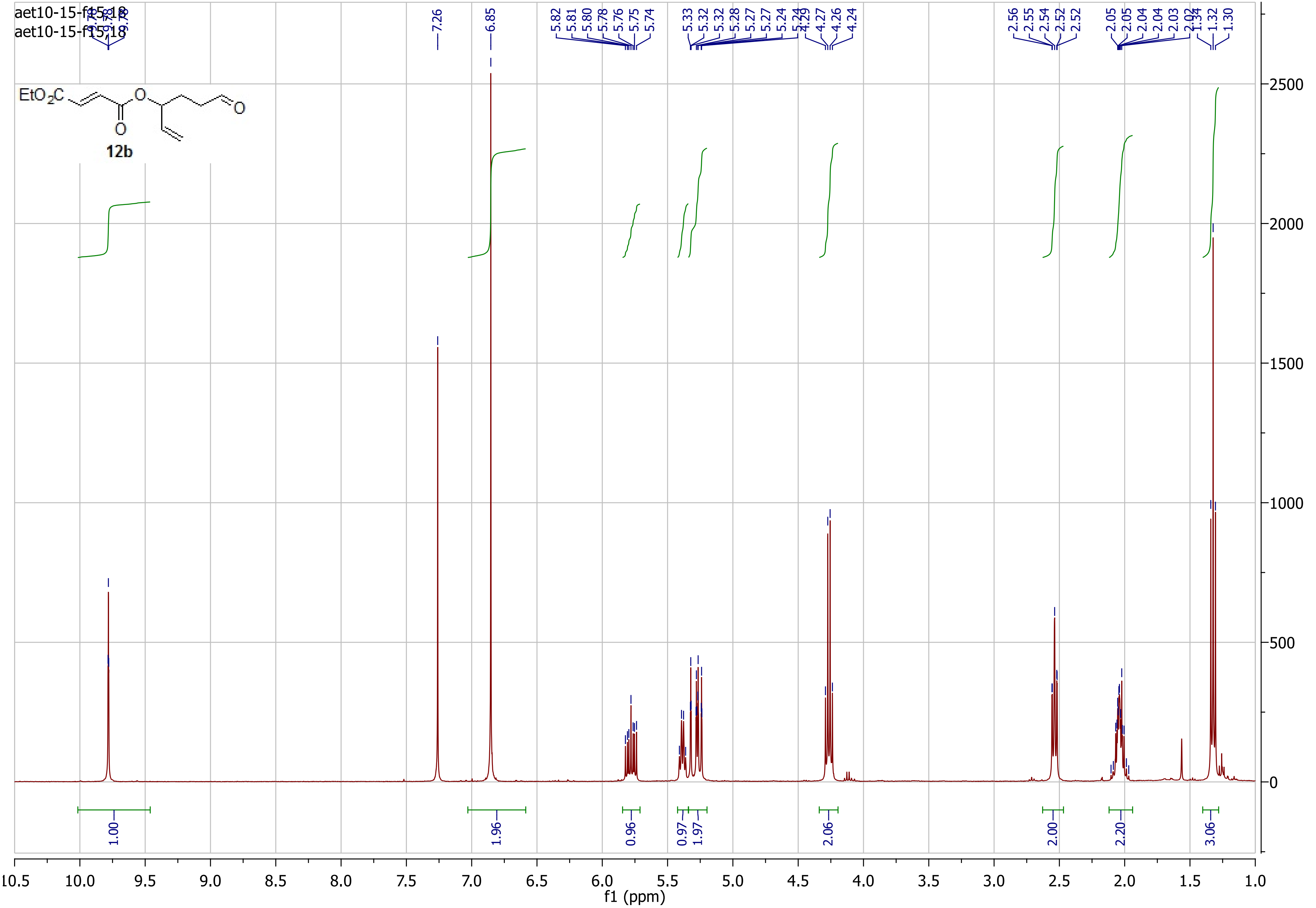

aet10-15-f15;18
aet10-15-f15;18
7.26
6.85
5.82
5.81
5.80
5.78
5.76
5.75
5.74
5.33
5.32
5.32
5.28
5.27
5.27
5.24
5.24
4.29
4.27
4.26
4.24
2.56
2.55
2.54
2.52
2.52
2.05
2.05
2.04
2.04
2.03
2.02
1.34
1.32
1.30
EtO2C
O
O
O
12b
1.00
1.96
0.96
0.97
1.97
2.06
2.00
2.20
3.06
f1 (ppm)

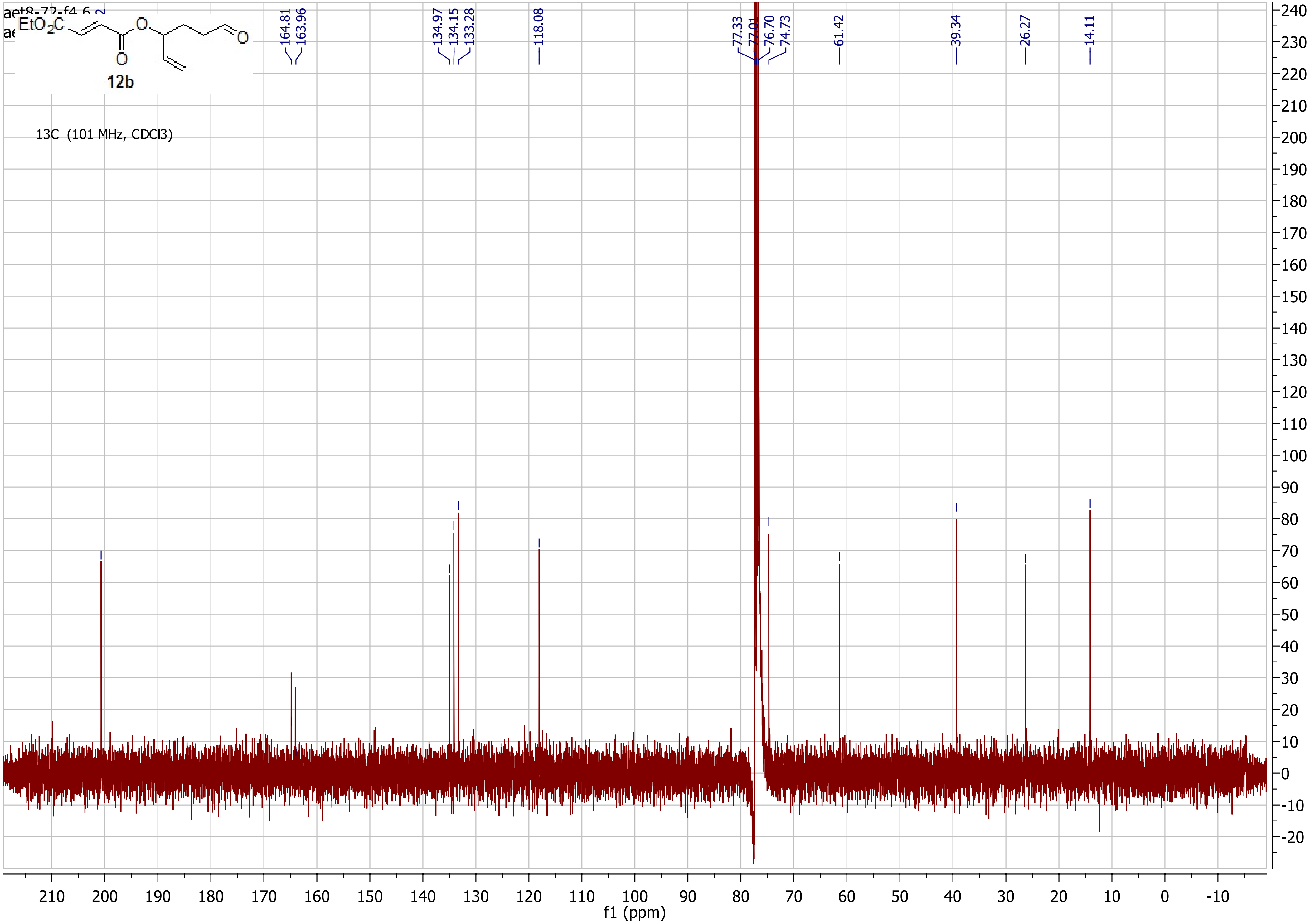

EtO2C
O
O
O
12b
13C (101 MHz, CDCl3)
164.81
163.96
134.97
134.15
133.28
118.08
77.33
77.01
76.70
74.73
61.42
39.34
26.27
14.11
f1 (ppm)

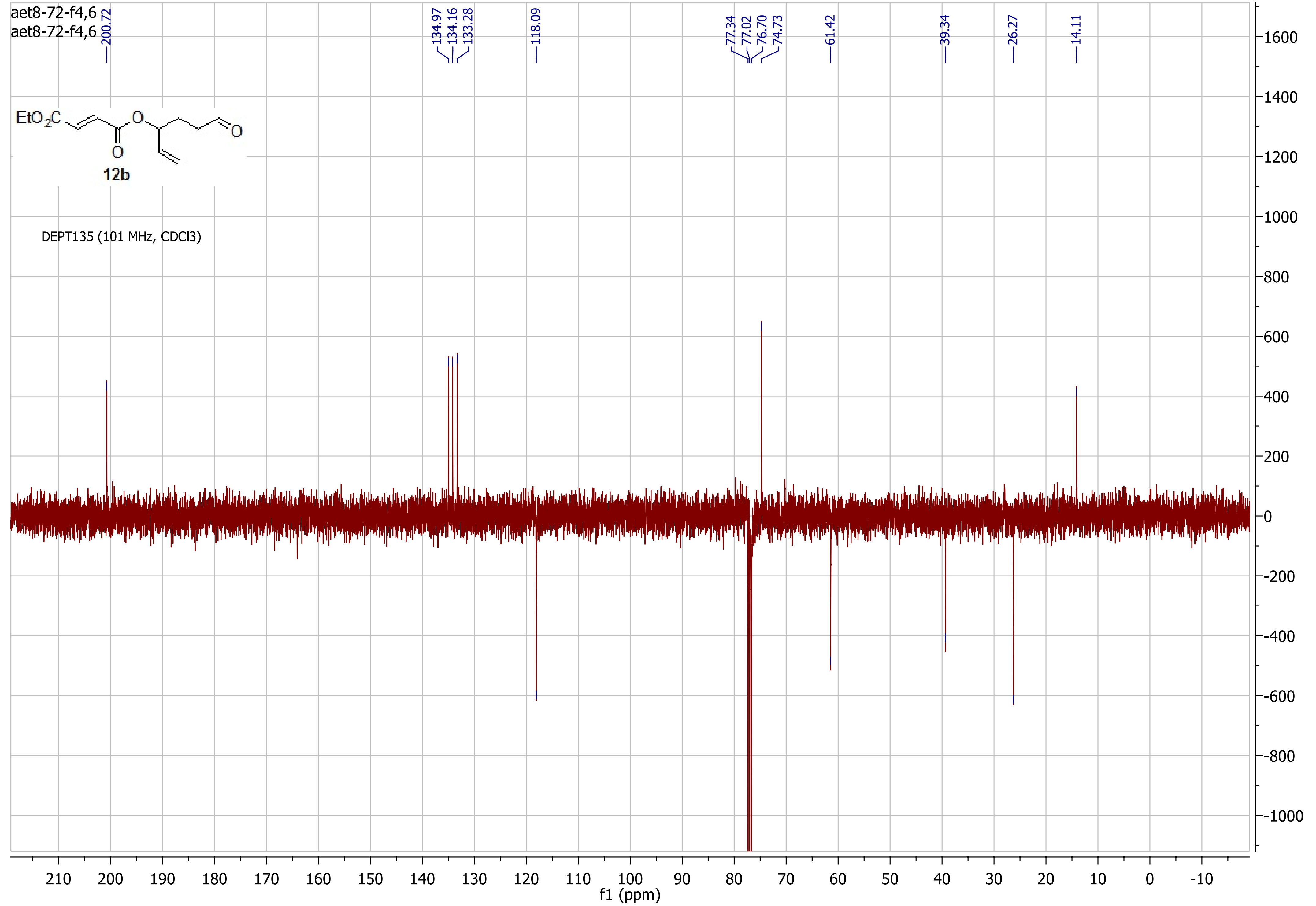
aet8-72-f4,6
aet8-72-f4,6
200.72
134.97
134.16
133.28
118.09
77.34
77.02
76.70
74.73
61.42
39.34
26.27
14.11
EtO2C
O
O
O
12b
DEPT135 (101 MHz, CDCl3)
f1 (ppm)

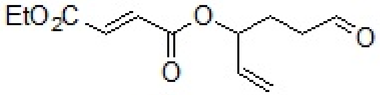

12b

COSY (400 MHz, CDCl3)

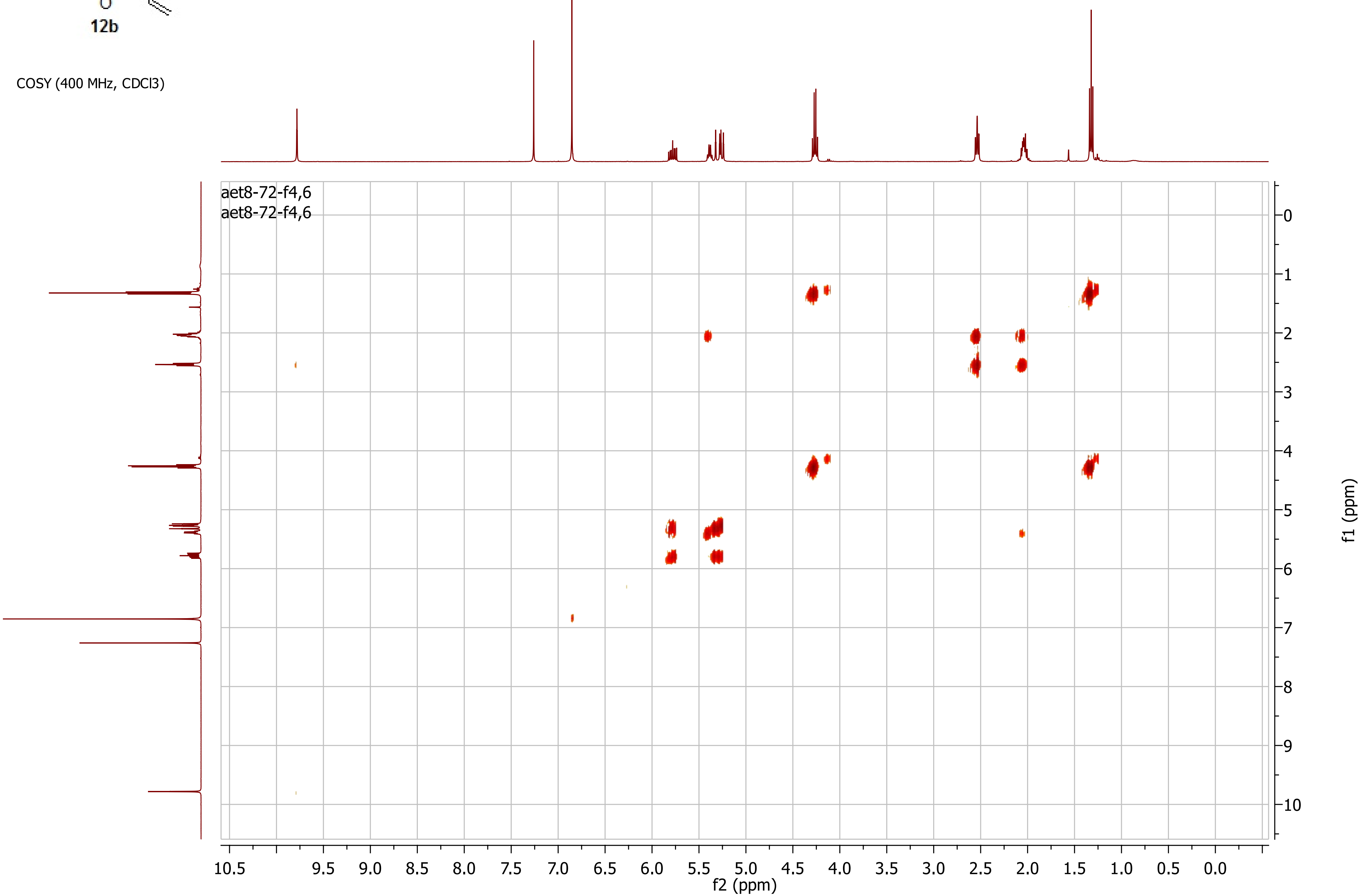

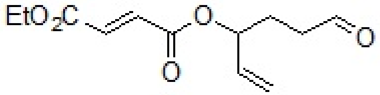

12b

HMQC (CDCl3)

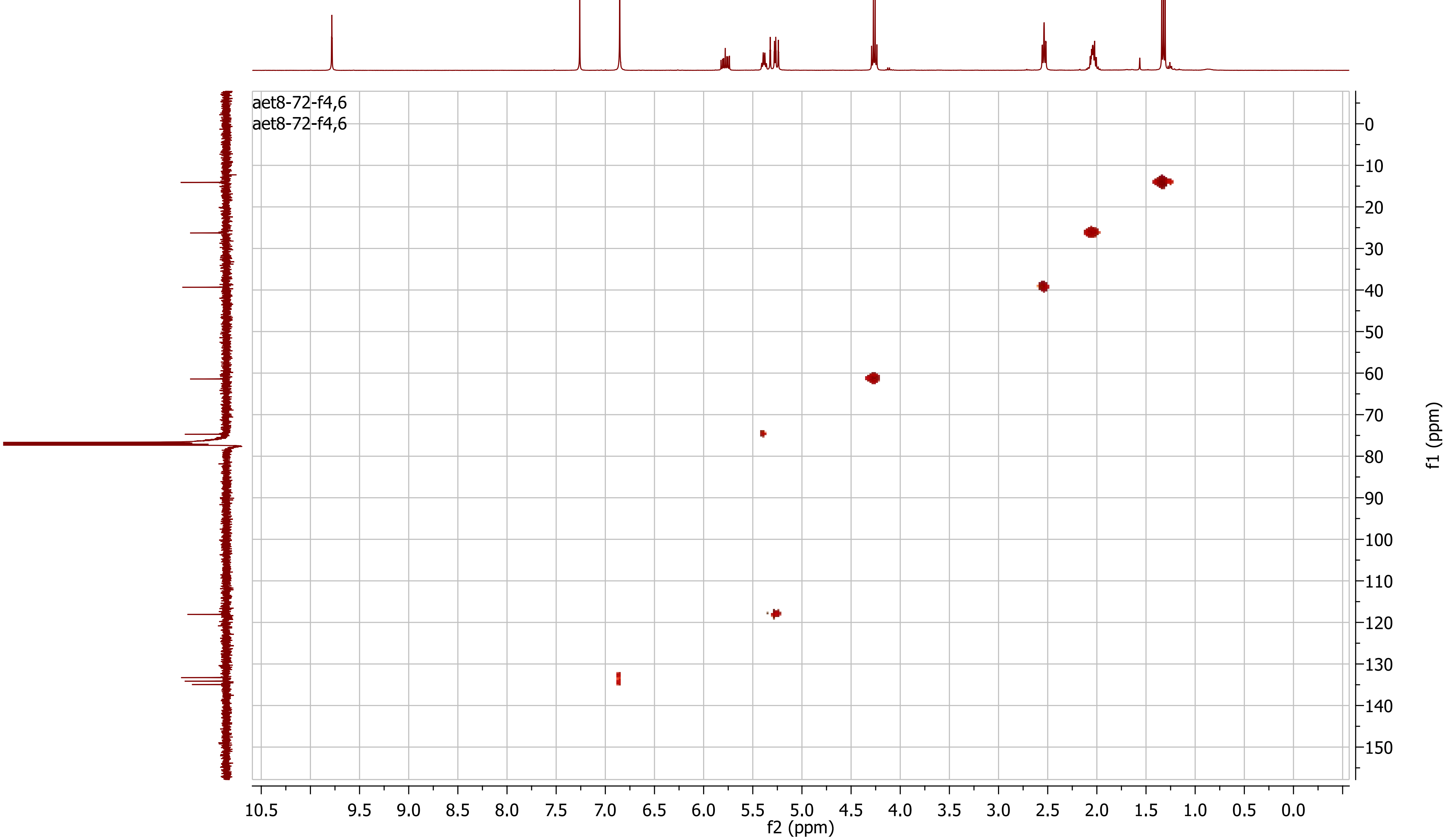

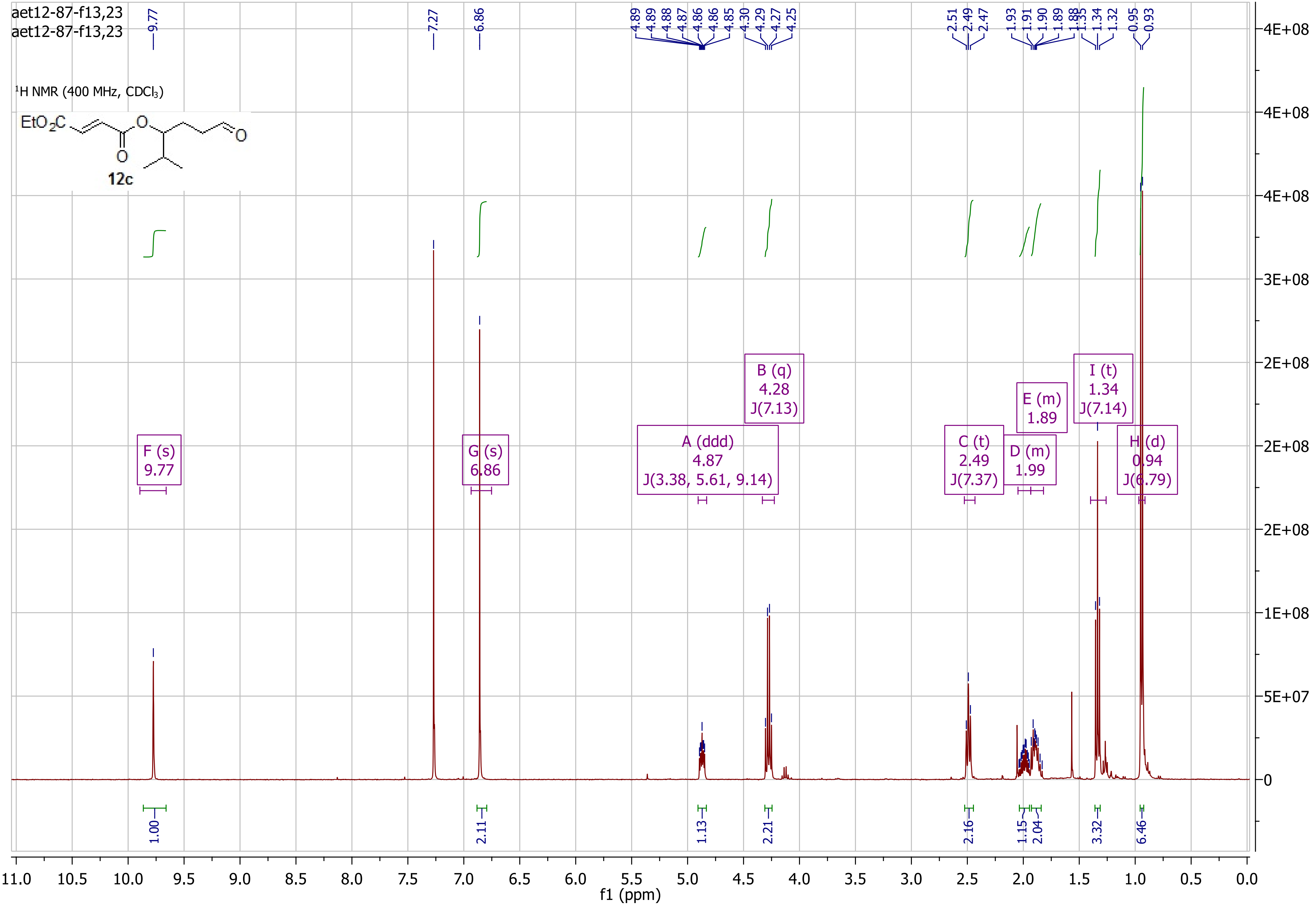
aet12-87-f13,23
aet12-87-f13,23
¹H NMR (400 MHz, CDCl₃)
EtO₂C
O
O
O
12c
F (s) 9.77
G (s) 6.86
A (ddd) 4.87 J(3.38, 5.61, 9.14)
B (q) 4.28 J(7.13)
C (t) 2.49 J(7.37)
D (m) 1.99
E (m) 1.89
I (t) 1.34 J(7.14)
H (d) 0.94 J(6.79)
f1 (ppm)

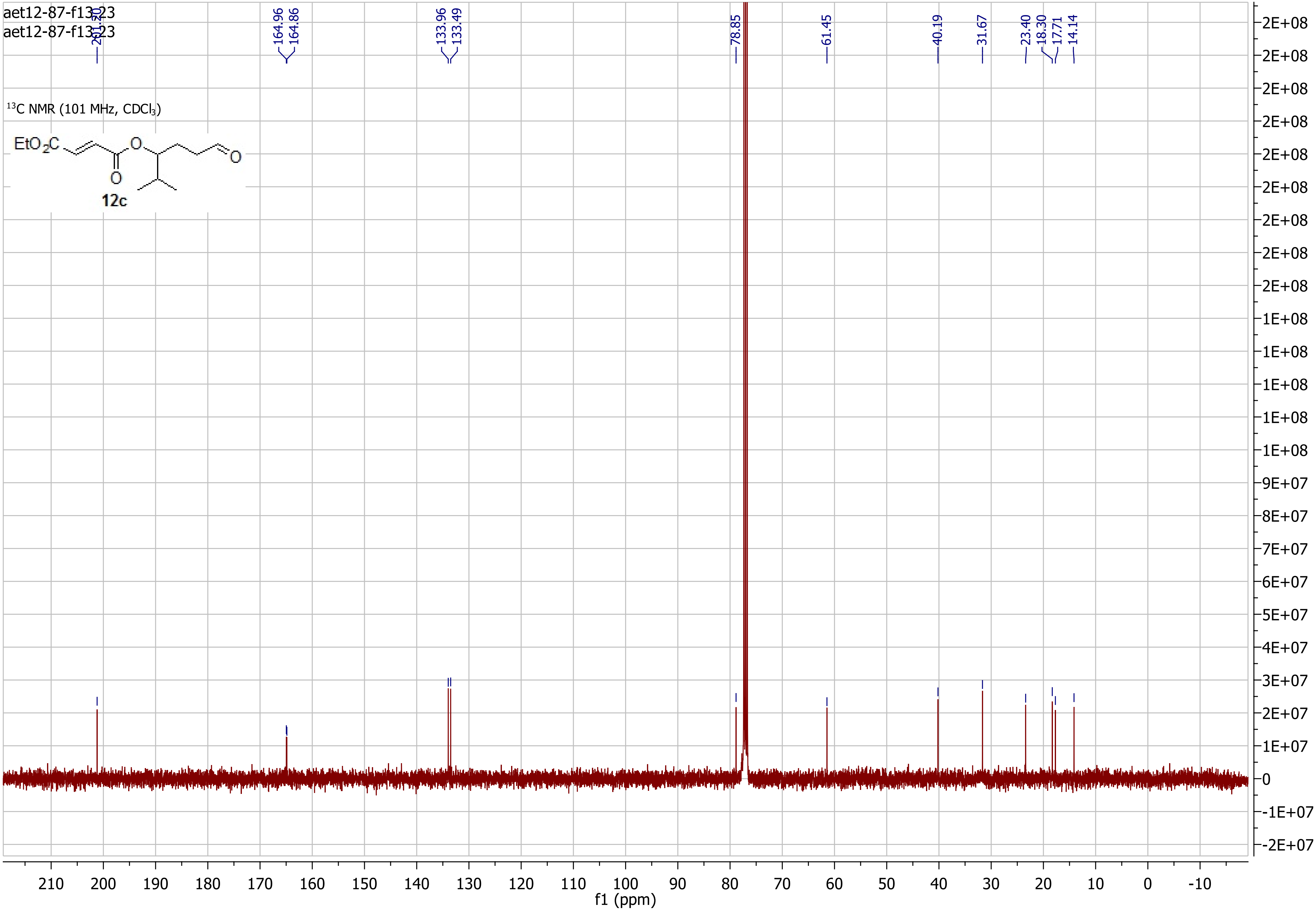
aet12-87-f13-23
aet12-87-f13-23
201.20
164.96
164.86
133.96
133.49
78.85
61.45
40.19
31.67
23.40
18.30
17.71
14.14
13C NMR (101 MHz, CDCl3)
EtO2C
O
O
O
12c
f1 (ppm)

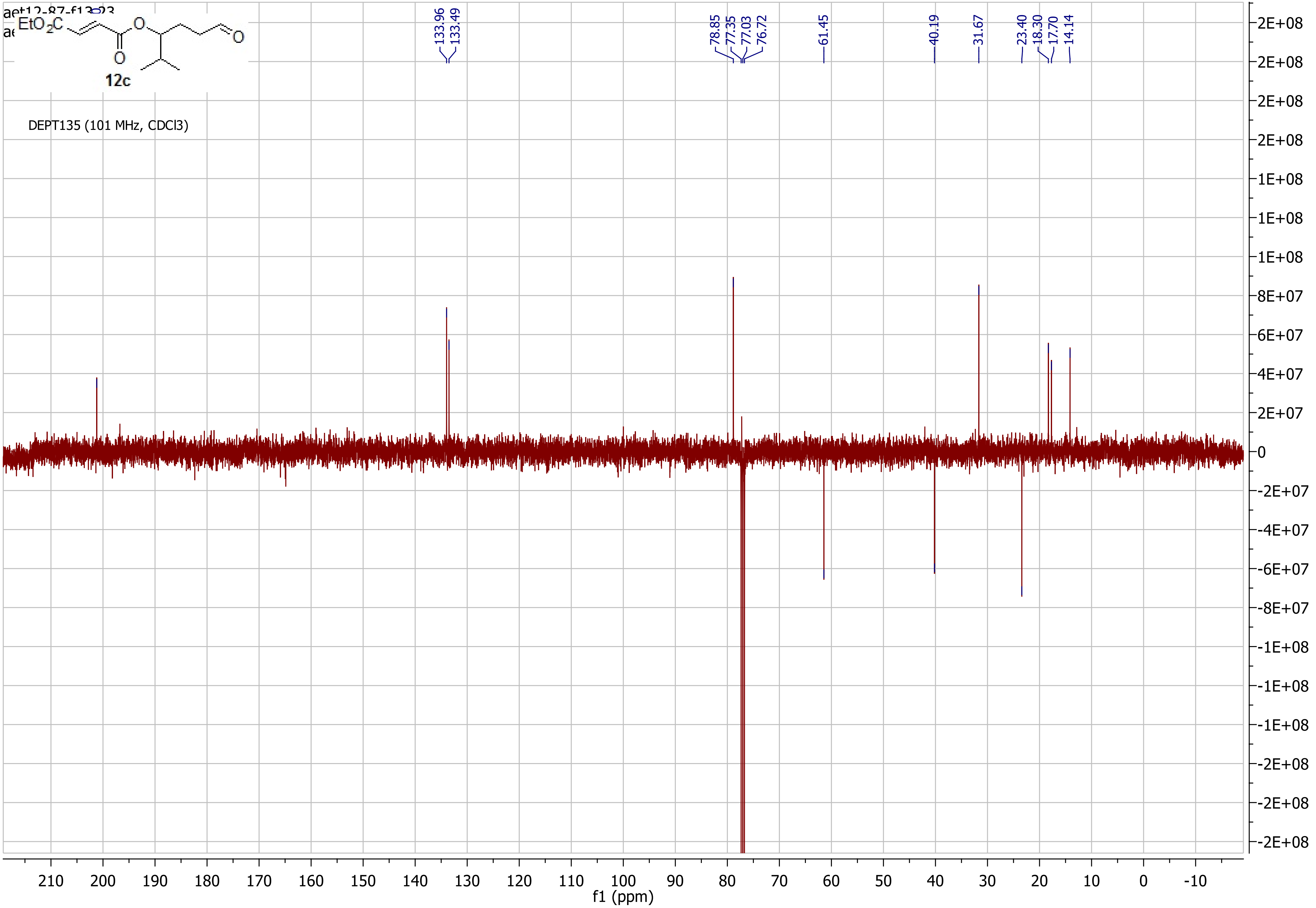

EtO2C
O
O
O
12c
DEPT135 (101 MHz, CDCl3)
133.96
133.49
78.85
77.35
77.03
76.72
61.45
40.19
31.67
23.40
18.30
17.70
14.14
f1 (ppm)

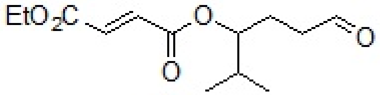

12c

COSY (400 MHz, CDCl3)

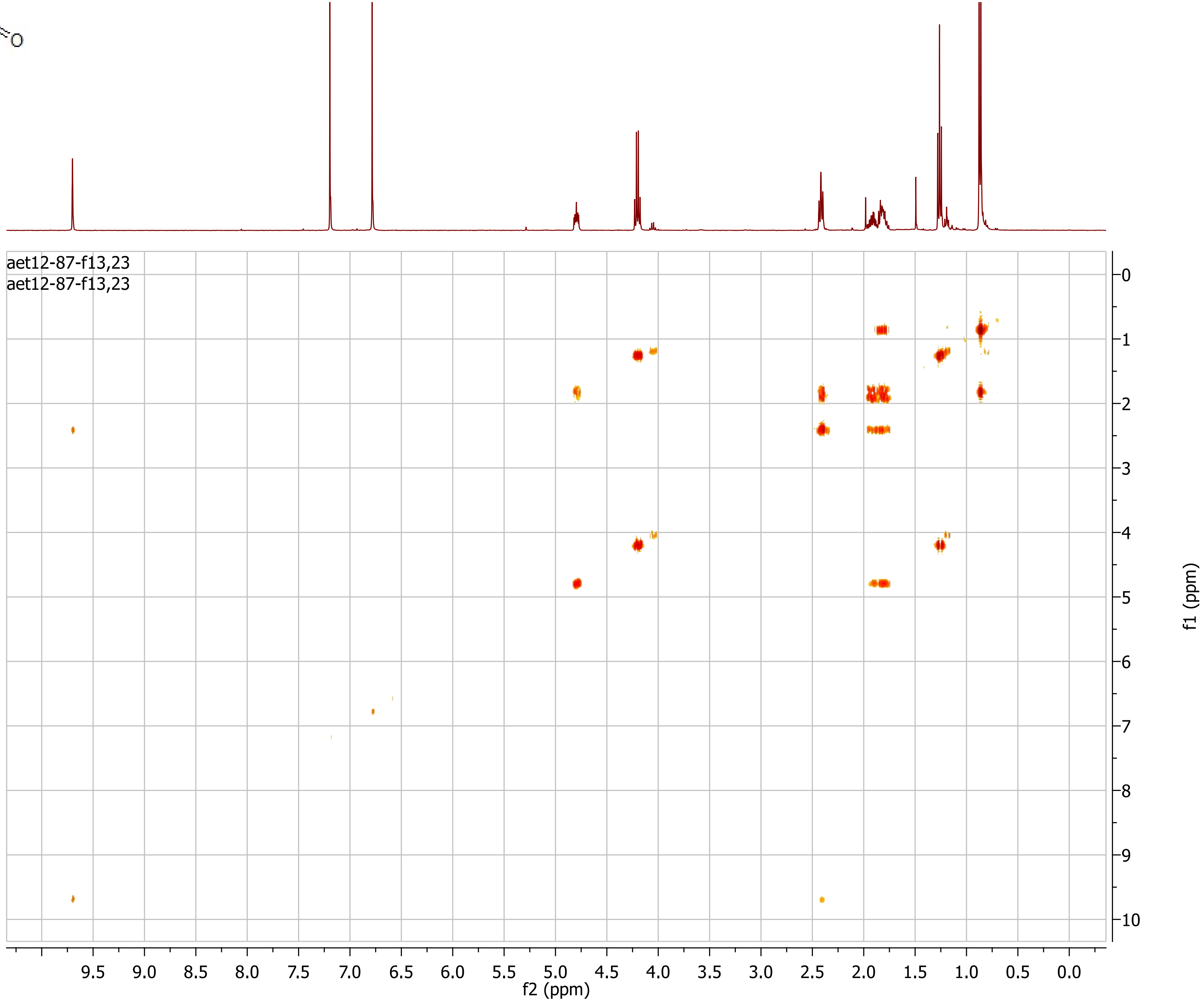

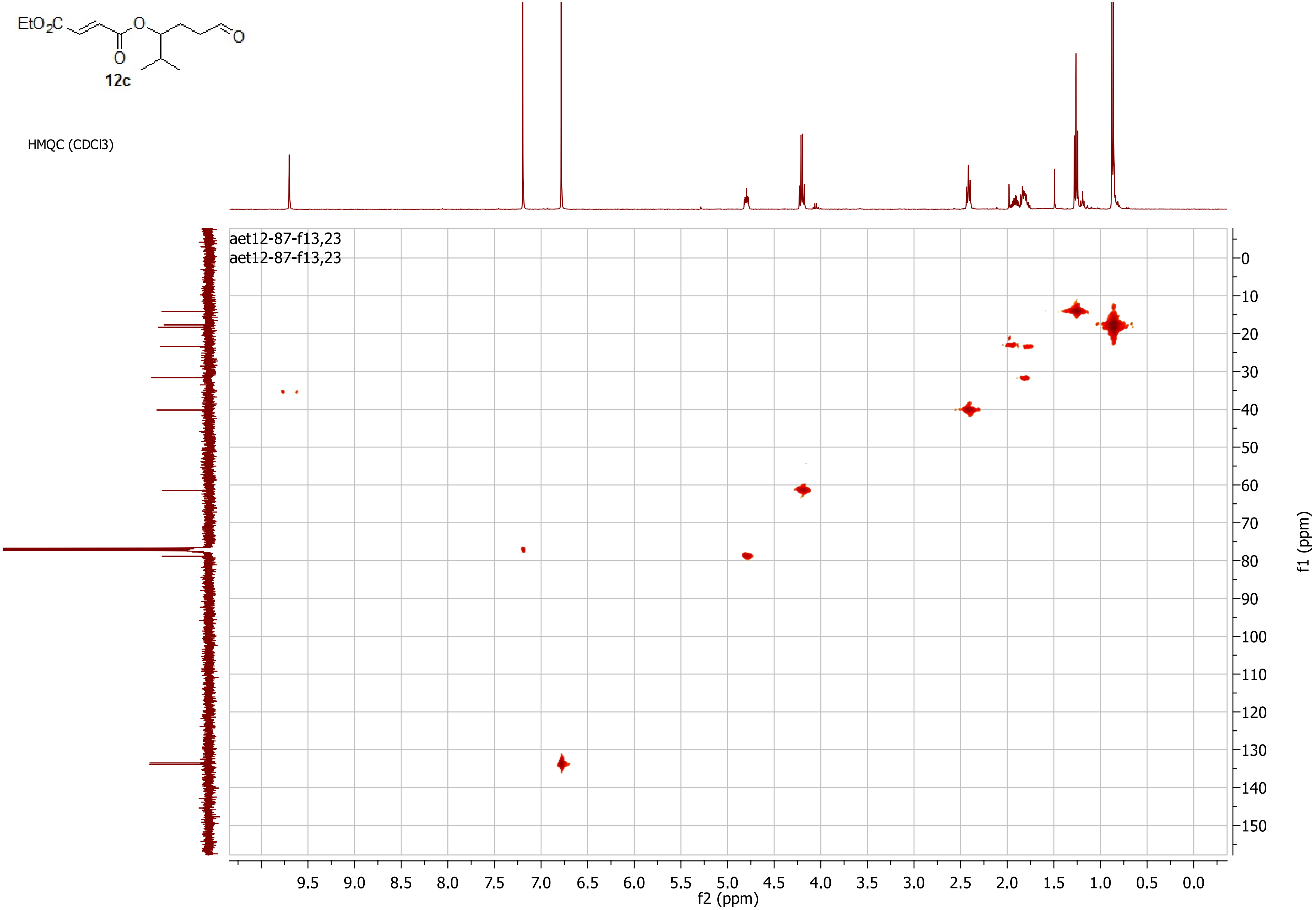
12c
HMQC (CDCl3)
aet12-87-f13,23
aet12-87-f13,23
f2 (ppm)
f1 (ppm)

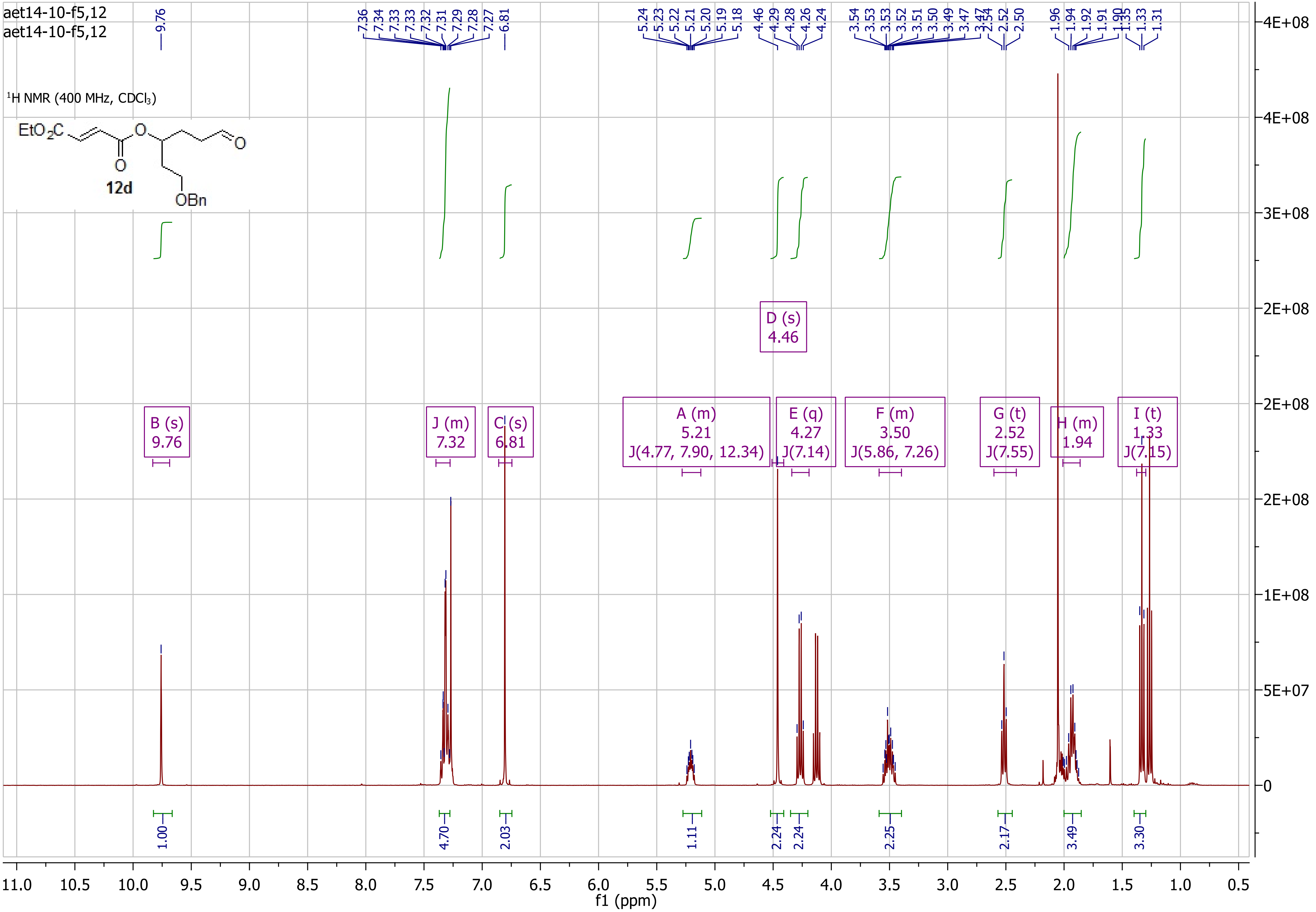
aet14-10-f5,12
aet14-10-f5,12
1H NMR (400 MHz, CDCl3)
EtO2C
O
O
O
OBn
12d
9.76
7.36 7.34 7.33 7.33 7.32 7.31 7.29 7.28 7.27 6.81
5.24 5.23 5.22 5.21 5.20 5.19 5.18 4.46 4.29 4.28 4.26 4.24
3.54 3.53 3.53 3.52 3.51 3.50 3.49 3.47 2.52 2.50
1.96 1.94 1.92 1.91 1.35 1.33 1.31
B (s) 9.76
J (m) 7.32
C (s) 6.81
A (m) 5.21 J(4.77, 7.90, 12.34)
D (s) 4.46
E (q) 4.27 J(7.14)
F (m) 3.50 J(5.86, 7.26)
G (t) 2.52 J(7.55)
H (m) 1.94
I (t) 1.33 J(7.15)
1.00
4.70
2.03
1.11
2.24
2.24
2.25
2.17
3.49
3.30
4E+08
4E+08
3E+08
2E+08
2E+08
2E+08
1E+08
5E+07
0
11.0 10.5 10.0 9.5 9.0 8.5 8.0 7.5 7.0 6.5 6.0 5.5 5.0 4.5 4.0 3.5 3.0 2.5 2.0 1.5 1.0 0.5
f1 (ppm)

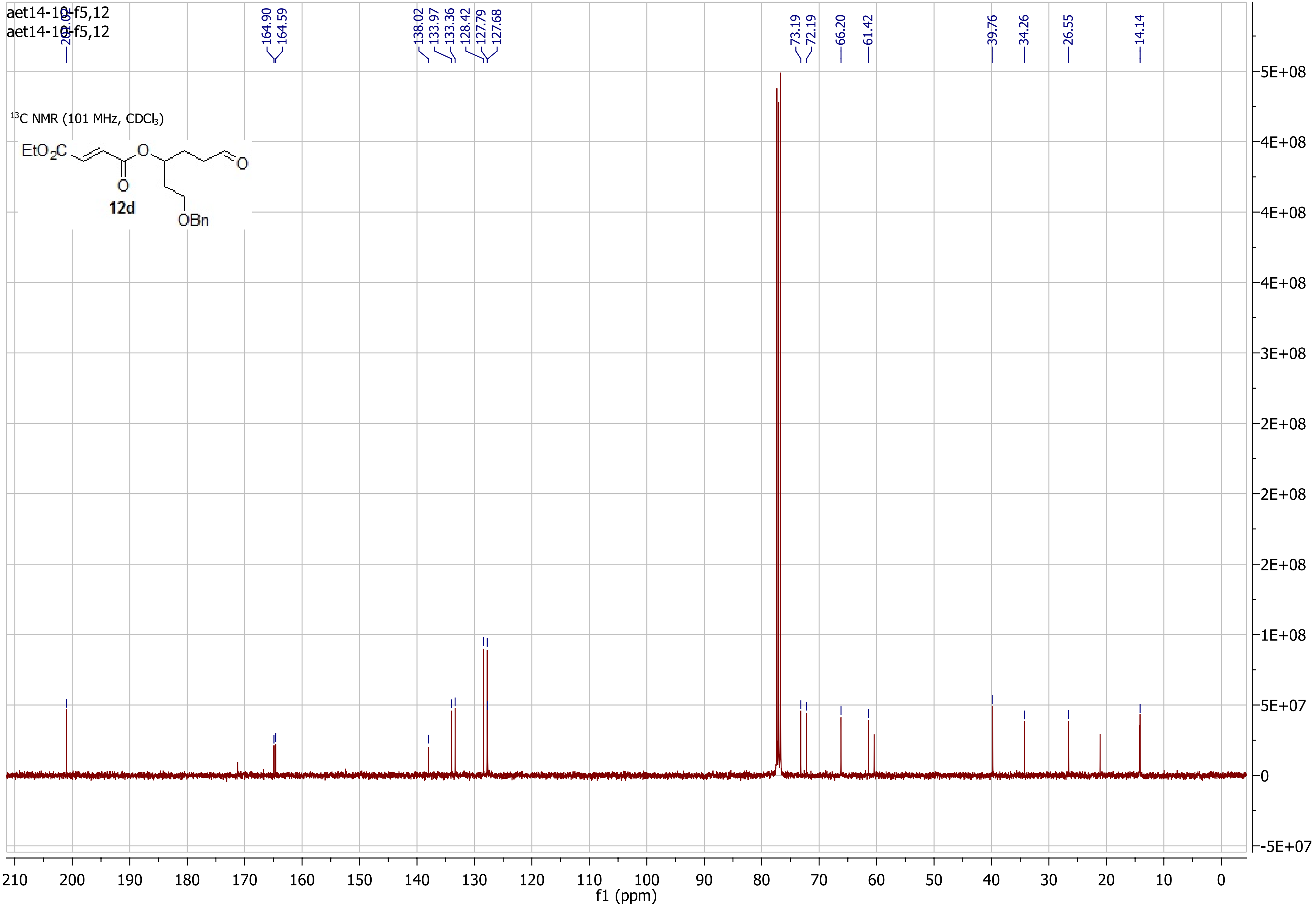

aet14-10-f5,12
aet14-10-f5,12
201.02
164.90
164.59
138.02
133.97
133.36
128.42
127.79
127.68
73.19
72.19
66.20
61.42
39.76
34.26
26.55
14.14
13C NMR (101 MHz, CDCl3)
EtO2C
O
O
O
12d
OBn
5E+08
4E+08
4E+08
4E+08
3E+08
2E+08
2E+08
2E+08
1E+08
5E+07
0
-5E+07
210
200
190
180
170
160
150
140
130
120
110
100
90
80
70
60
50
40
30
20
10
0
f1 (ppm)

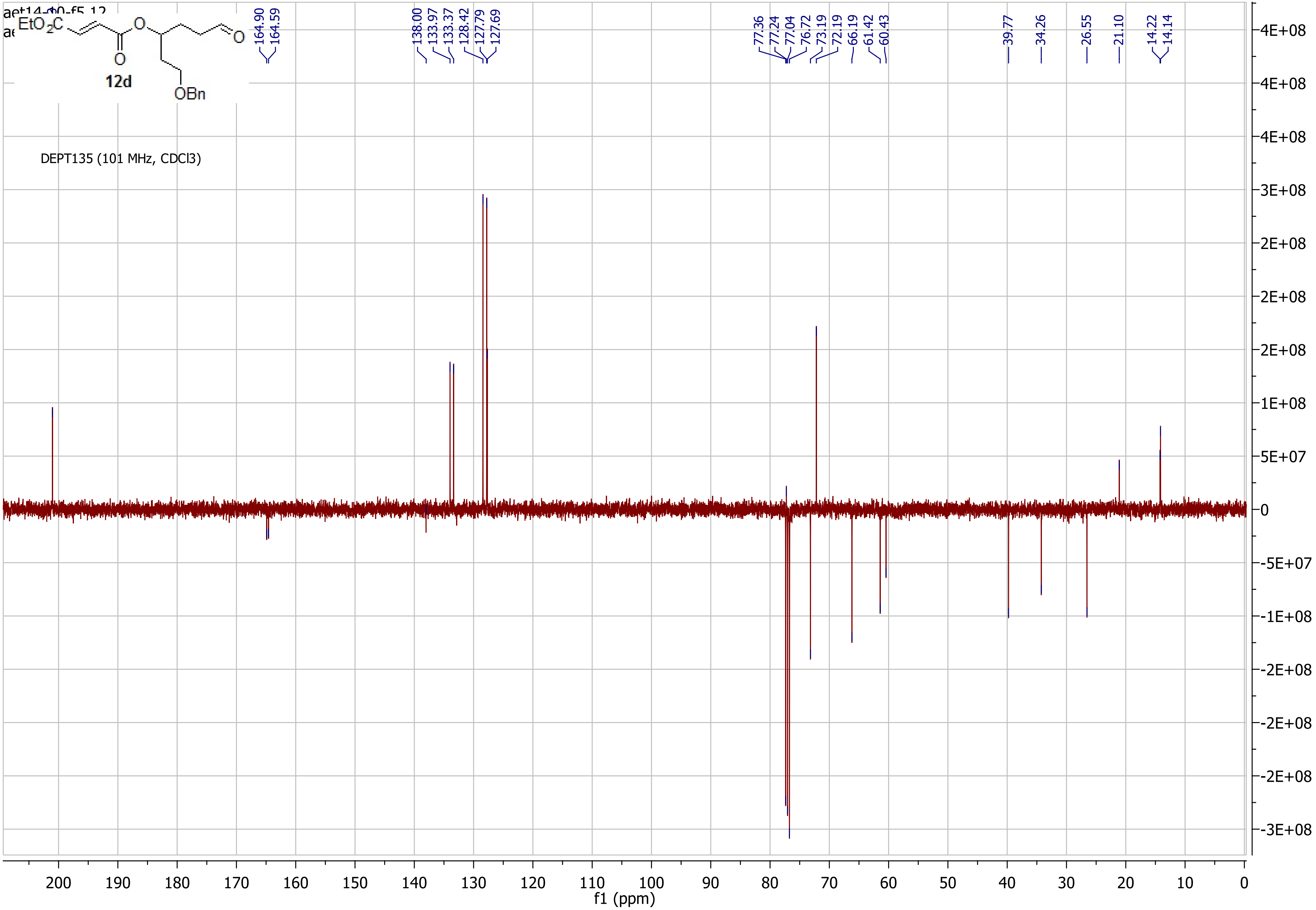

12d
OBn
DEPT135 (101 MHz, CDCl3)
164.90
164.59
138.00
133.97
133.37
128.42
127.79
127.69
77.36
77.24
77.04
76.72
73.19
72.19
66.19
61.42
60.43
39.77
34.26
26.55
21.10
14.22
14.14
f1 (ppm)

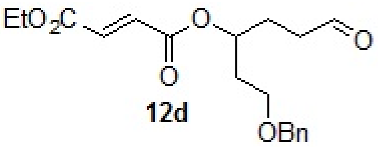

12d

COSY (400 MHz, CDCl3)

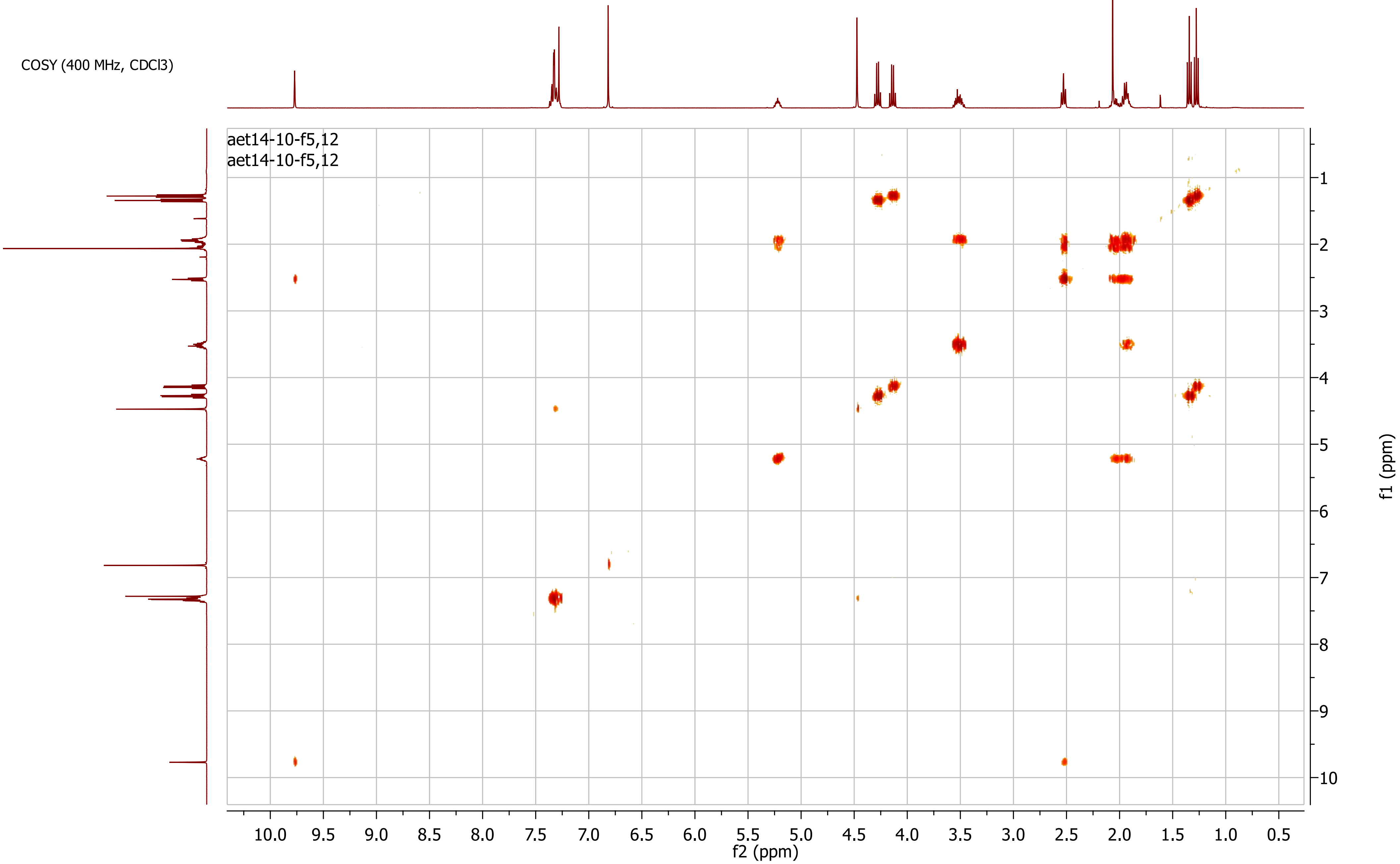

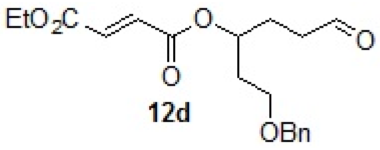

12d

HMQC (CDCl3)

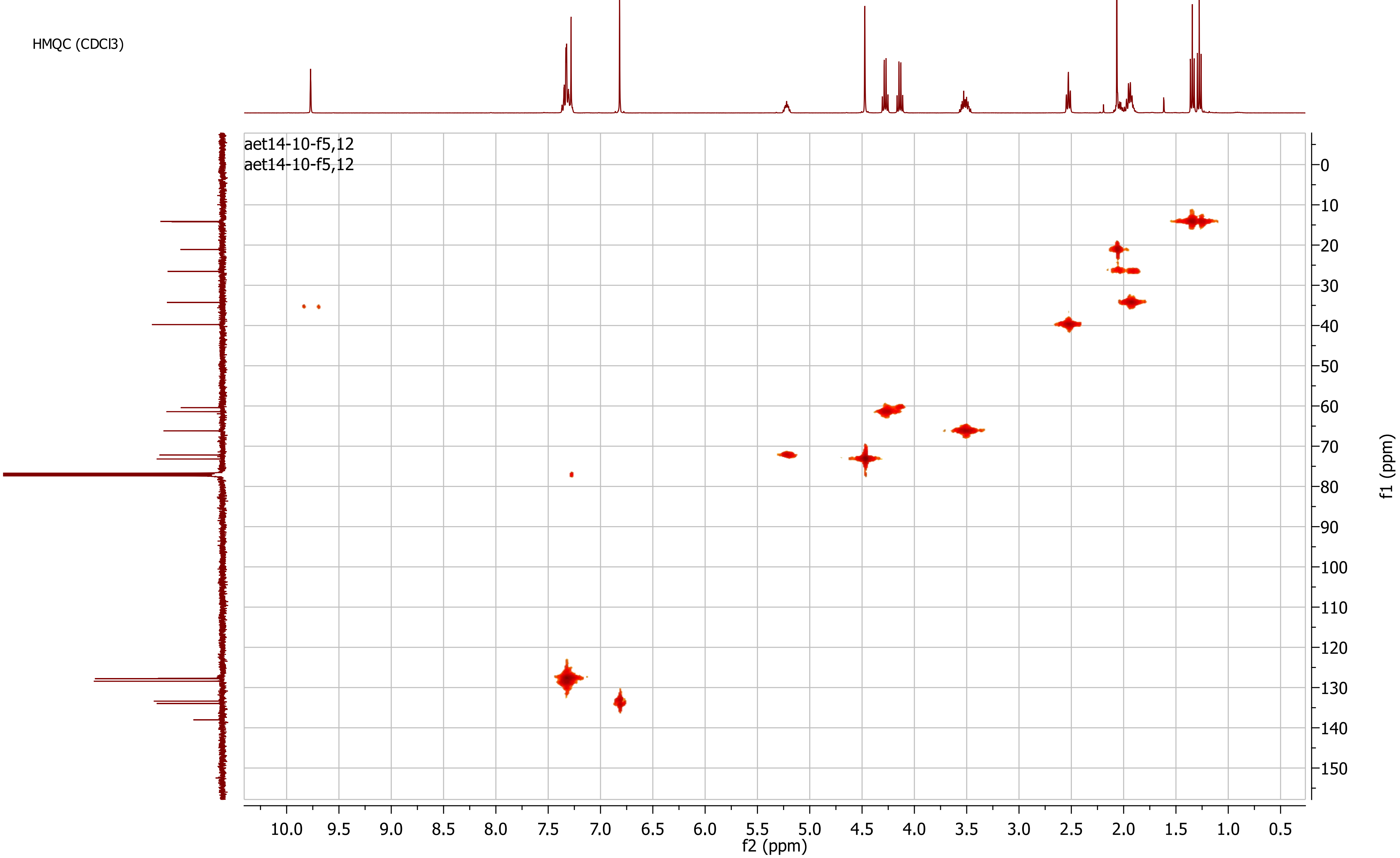

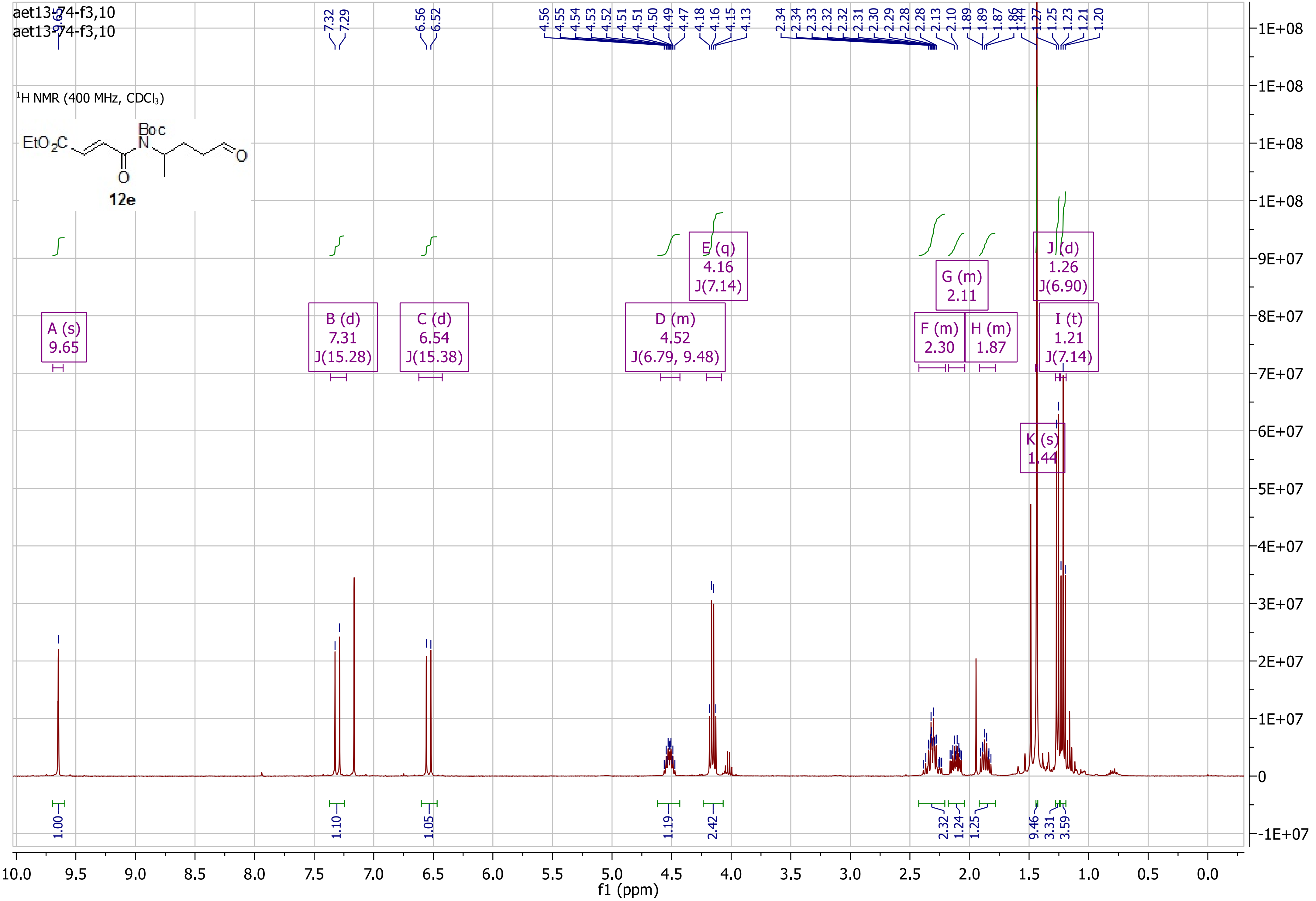
aet13-74-f3,10
aet13-74-f3,10
1H NMR (400 MHz, CDCl3)
EtO2C
Boc
N
O
O
12e
A (s) 9.65
B (d) 7.31 J(15.28)
C (d) 6.54 J(15.38)
D (m) 4.52 J(6.79, 9.48)
E (q) 4.16 J(7.14)
F (m) 2.30
G (m) 2.11
H (m) 1.87
I (t) 1.21 J(7.14)
J (d) 1.26 J(6.90)
K (s) 1.44
7.32
7.29
6.56
6.52
4.56
4.55
4.54
4.53
4.52
4.51
4.51
4.50
4.49
4.47
4.18
4.16
4.15
4.13
2.34
2.34
2.33
2.32
2.32
2.31
2.30
2.29
2.28
2.28
2.13
2.10
1.89
1.89
1.87
1.86
1.44
1.27
1.25
1.23
1.21
1.20
1.00
1.10
1.05
1.19
2.42
2.32
1.24
1.25
9.46
3.31
3.59
f1 (ppm)

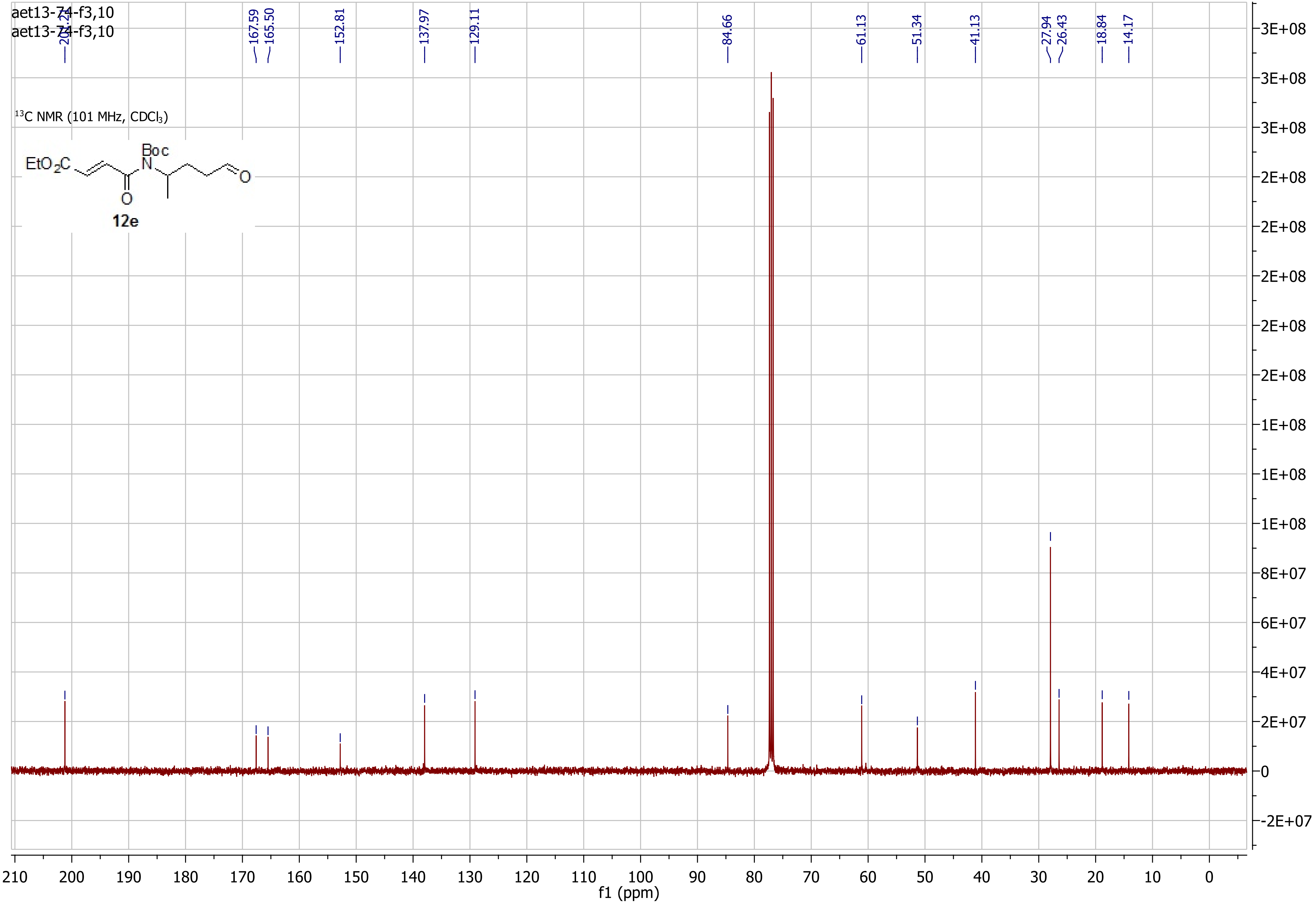
aet13-74-f3,10
aet13-74-f3,10
167.59
165.50
152.81
137.97
129.11
84.66
61.13
51.34
41.13
27.94
26.43
18.84
14.17
13C NMR (101 MHz, CDCl3)
EtO2C
Boc
N
O
O
12e
f1 (ppm)

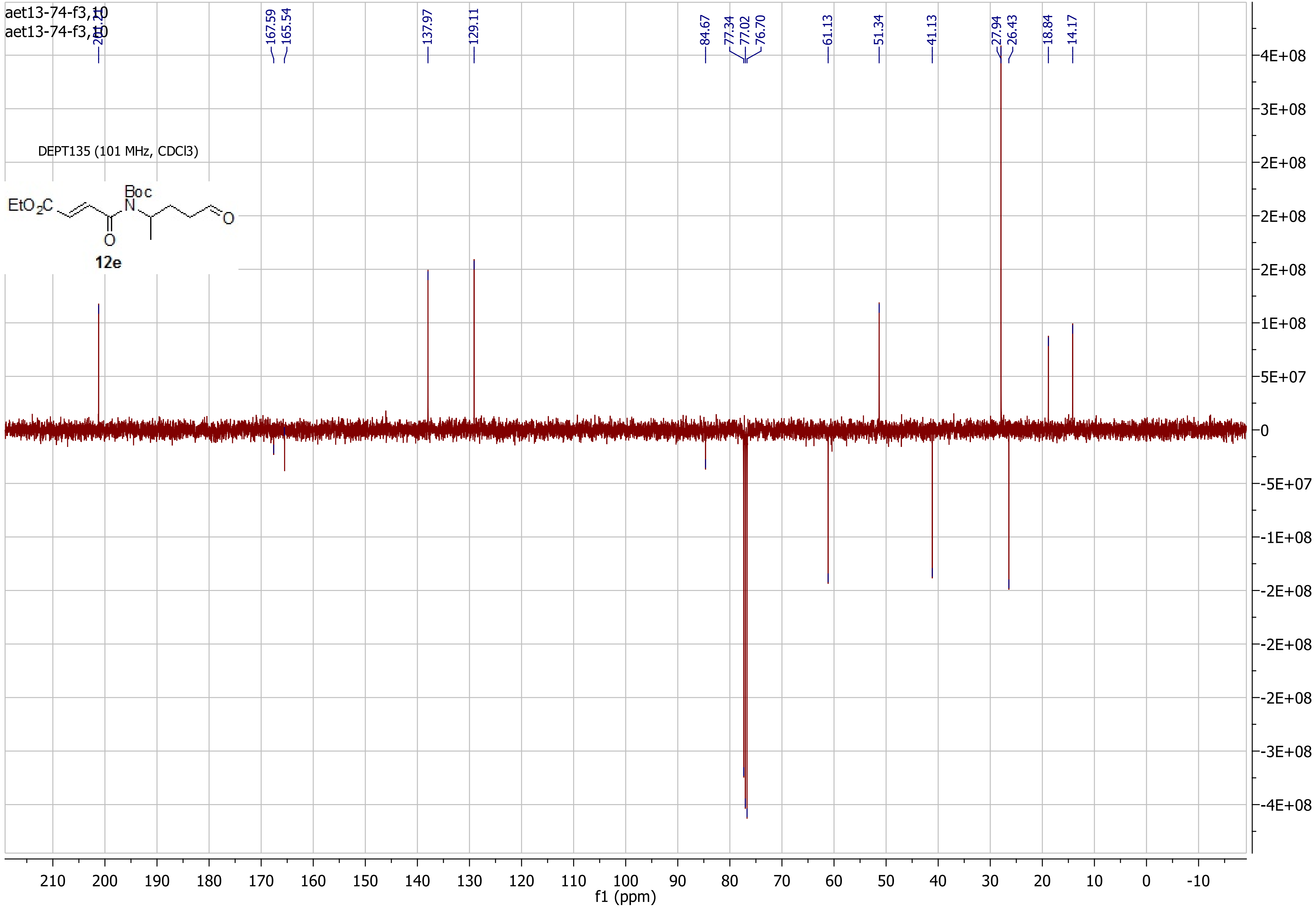

aet13-74-f3,10
aet13-74-f3,10
201.21
167.59
165.54
137.97
129.11
84.67
77.34
77.02
76.70
61.13
51.34
41.13
27.94
26.43
18.84
14.17
DEPT135 (101 MHz, CDCl3)
EtO2C
Boc
N
O
O
12e
4E+08
3E+08
2E+08
2E+08
2E+08
1E+08
5E+07
0
-5E+07
-1E+08
-2E+08
-2E+08
-2E+08
-3E+08
-4E+08
210
200
190
180
170
160
150
140
130
120
110
100
90
80
70
60
50
40
30
20
10
0
-10
f1 (ppm)

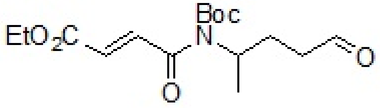

12e

COSY (400 MHz, CDCl3)

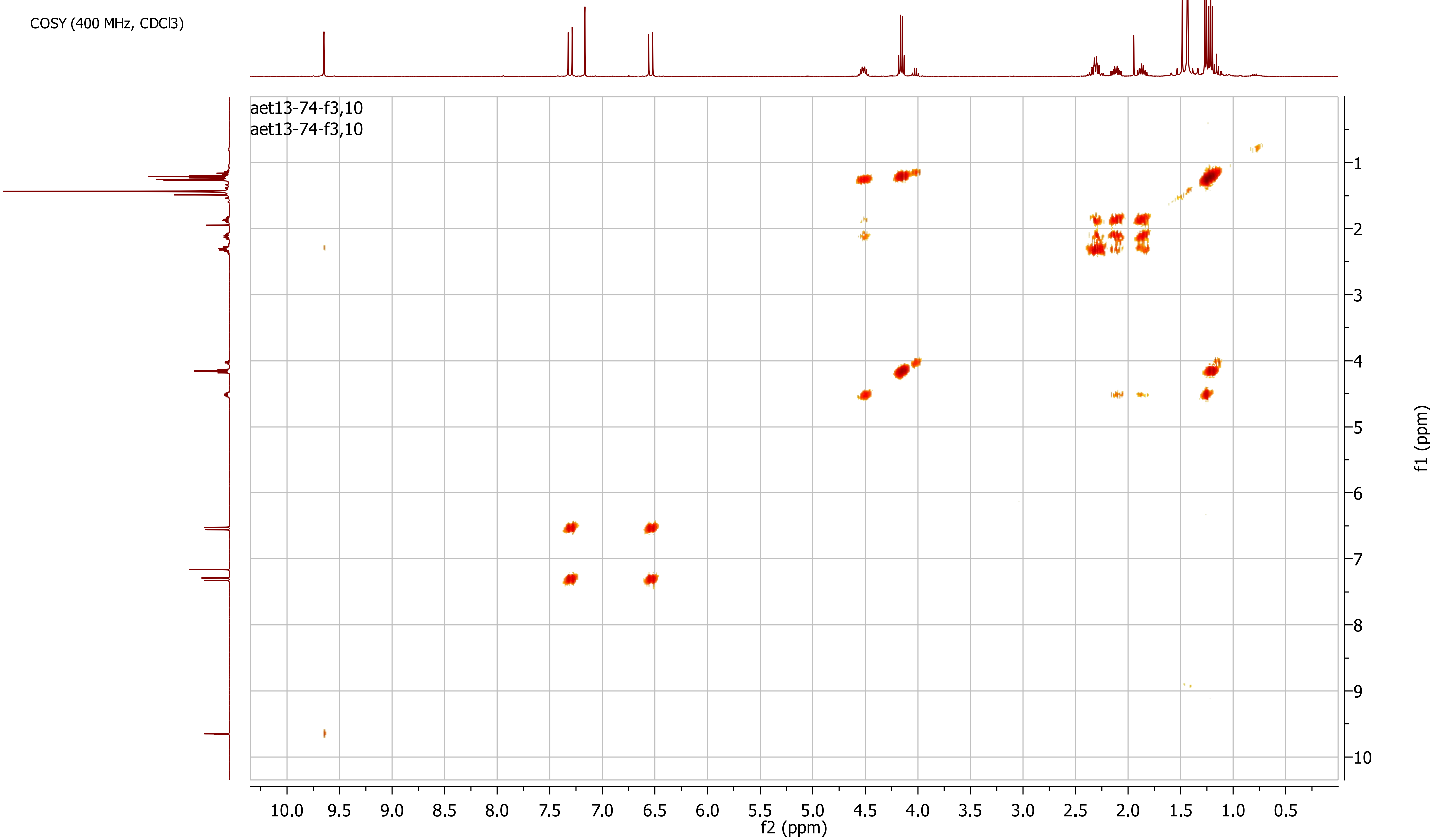

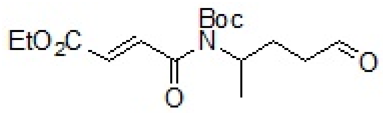

12e

HMQC (CDCl3)

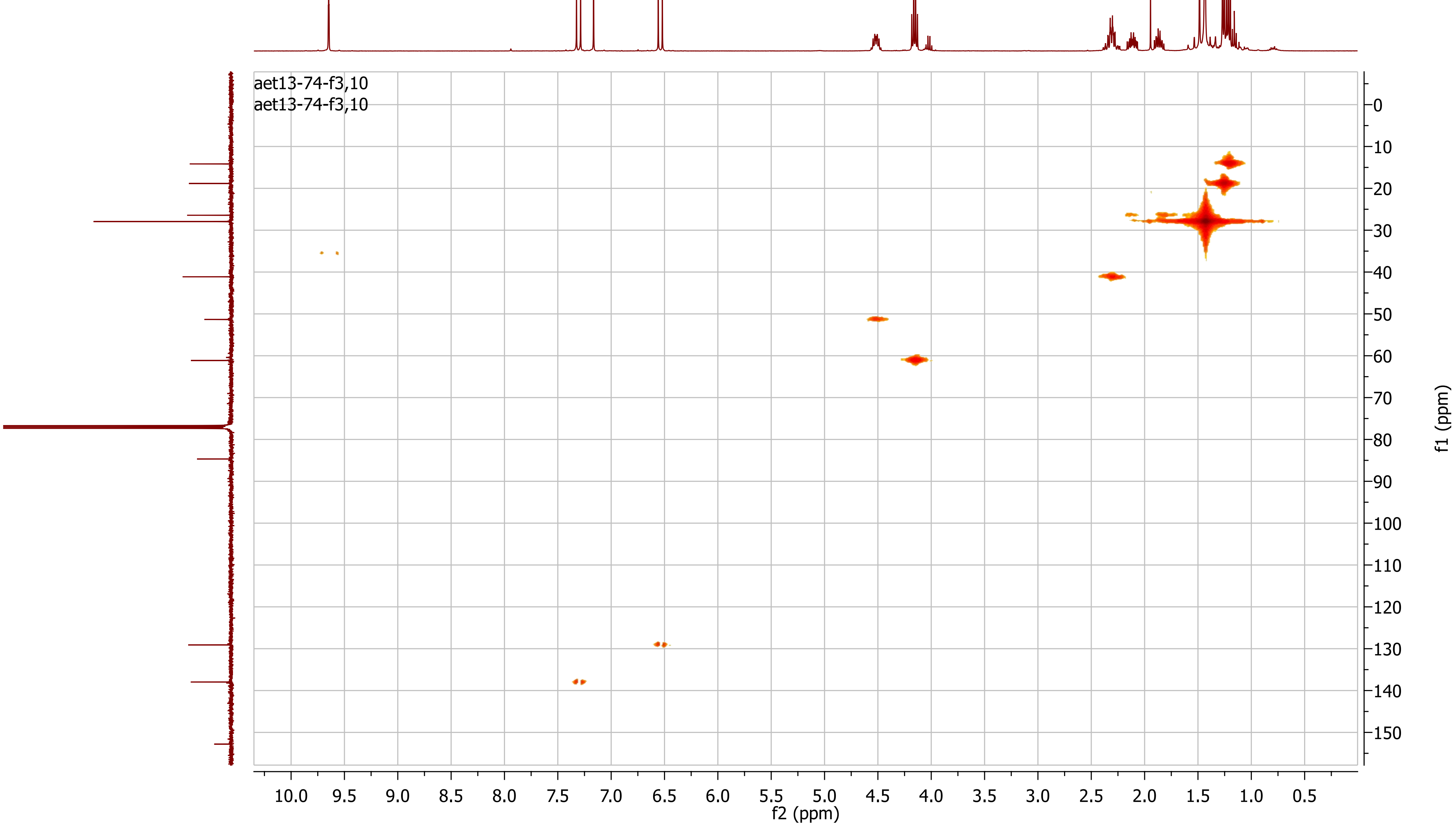

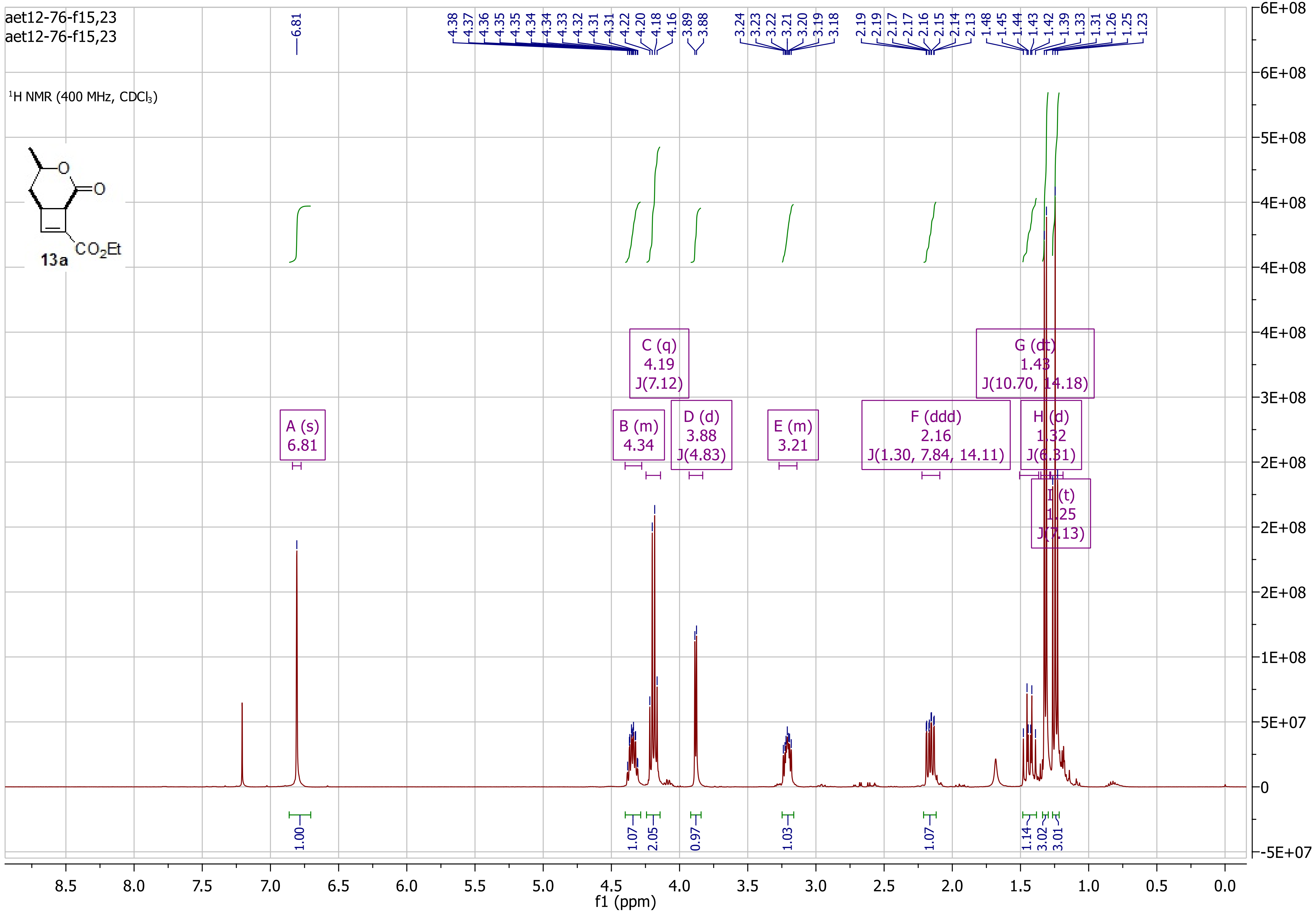
aet12-76-f15,23
aet12-76-f15,23
¹H NMR (400 MHz, CDCl3)
13a
CO2Et
A (s) 6.81
B (m) 4.34
C (q) 4.19 J(7.12)
D (d) 3.88 J(4.83)
E (m) 3.21
F (ddd) 2.16 J(1.30, 7.84, 14.11)
G (dt) 1.43 J(10.70, 14.18)
H (d) 1.32 J(6.31)
I (t) 1.25 J(7.13)
1.00
1.07
2.05
0.97
1.03
1.07
1.14
3.02
3.01
f1 (ppm)

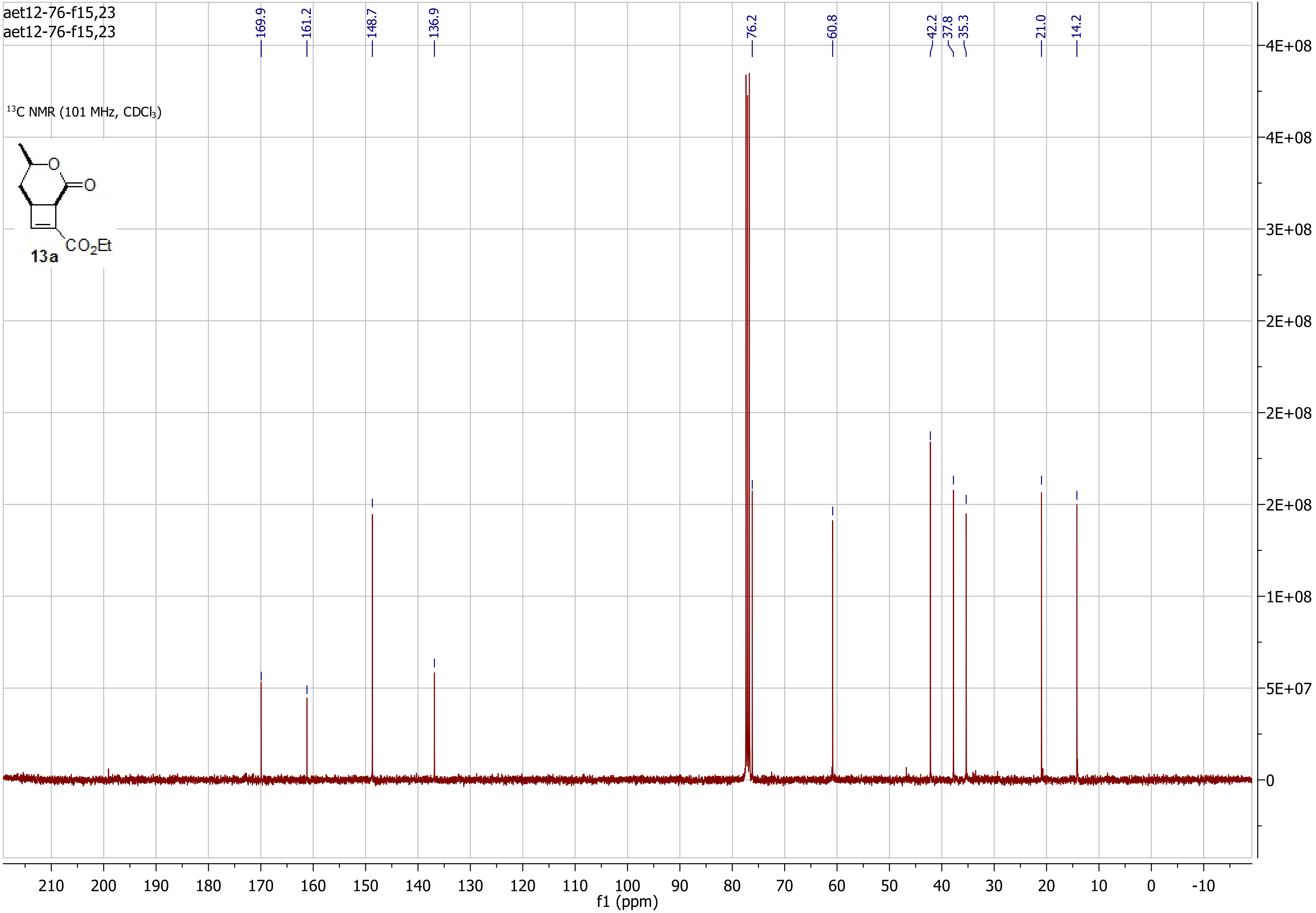
aet12-76-f15,23
aet12-76-f15,23
13C NMR (101 MHz, CDCl3)
13a
CO2Et
169.9
161.2
148.7
136.9
76.2
60.8
42.2
37.8
35.3
21.0
14.2
f1 (ppm)

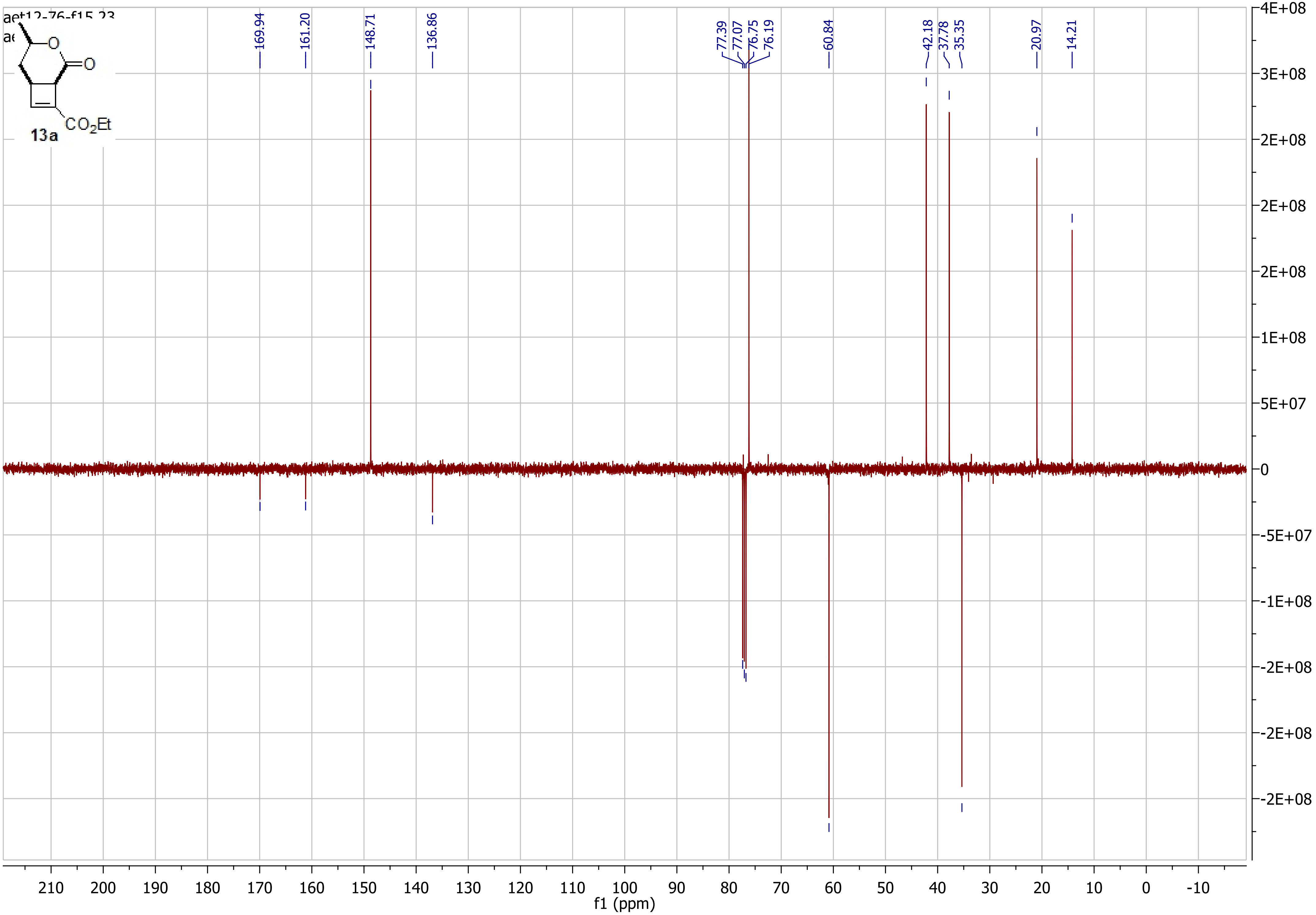

13a
CO_2Et
169.94
161.20
148.71
136.86
77.39
77.07
76.75
76.19
60.84
42.18
37.78
35.35
20.97
14.21
4E+08
3E+08
2E+08
1E+08
5E+07
0
-5E+07
-1E+08
-2E+08
210 200 190 180 170 160 150 140 130 120 110 100 90 80 70 60 50 40 30 20 10 0 -10
f1 (ppm)

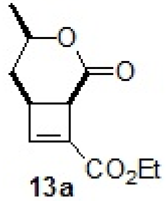

13a

COSY (400 MHz, CDCl3)

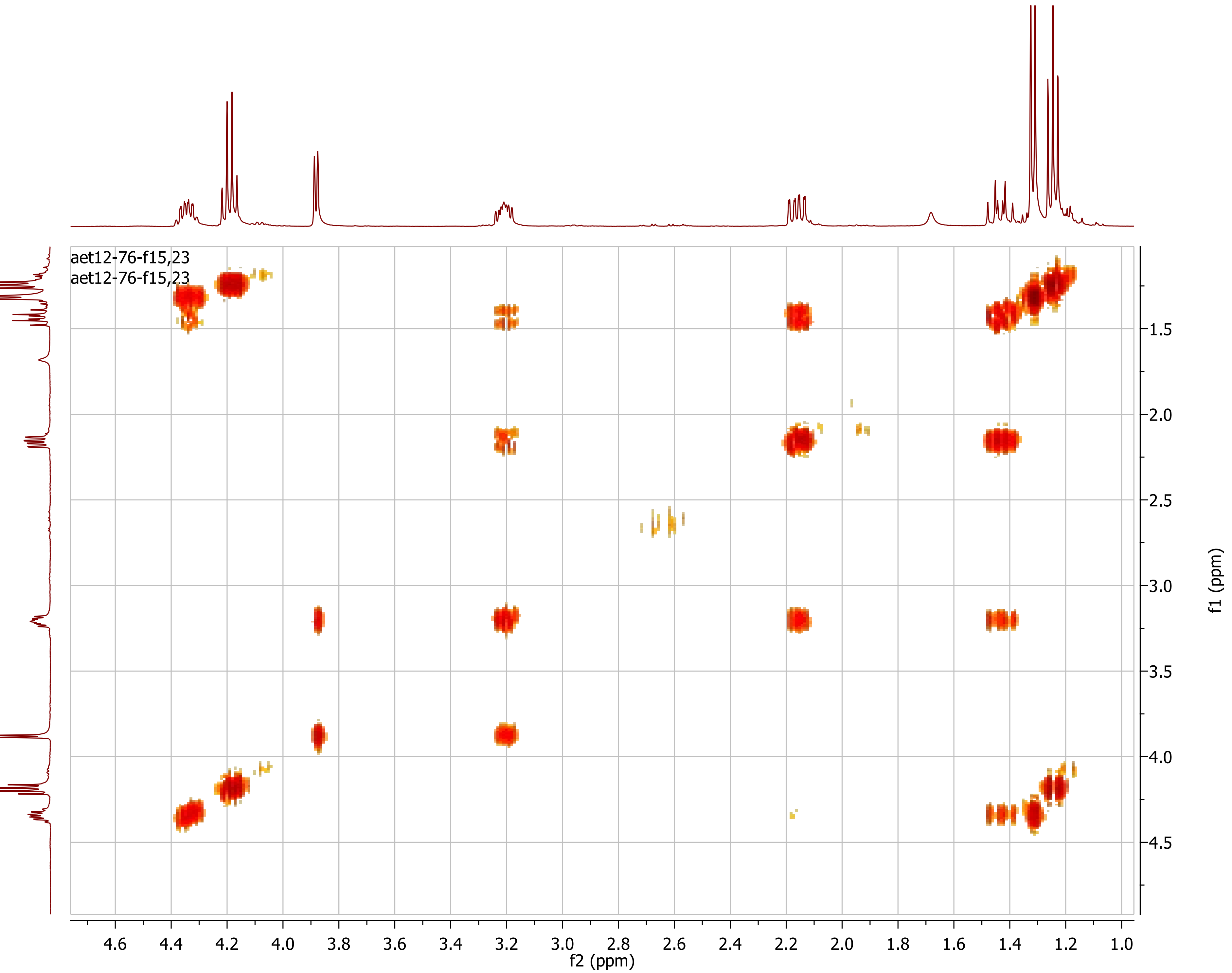

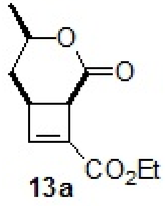

COSY (400 MHz, CDCl3)

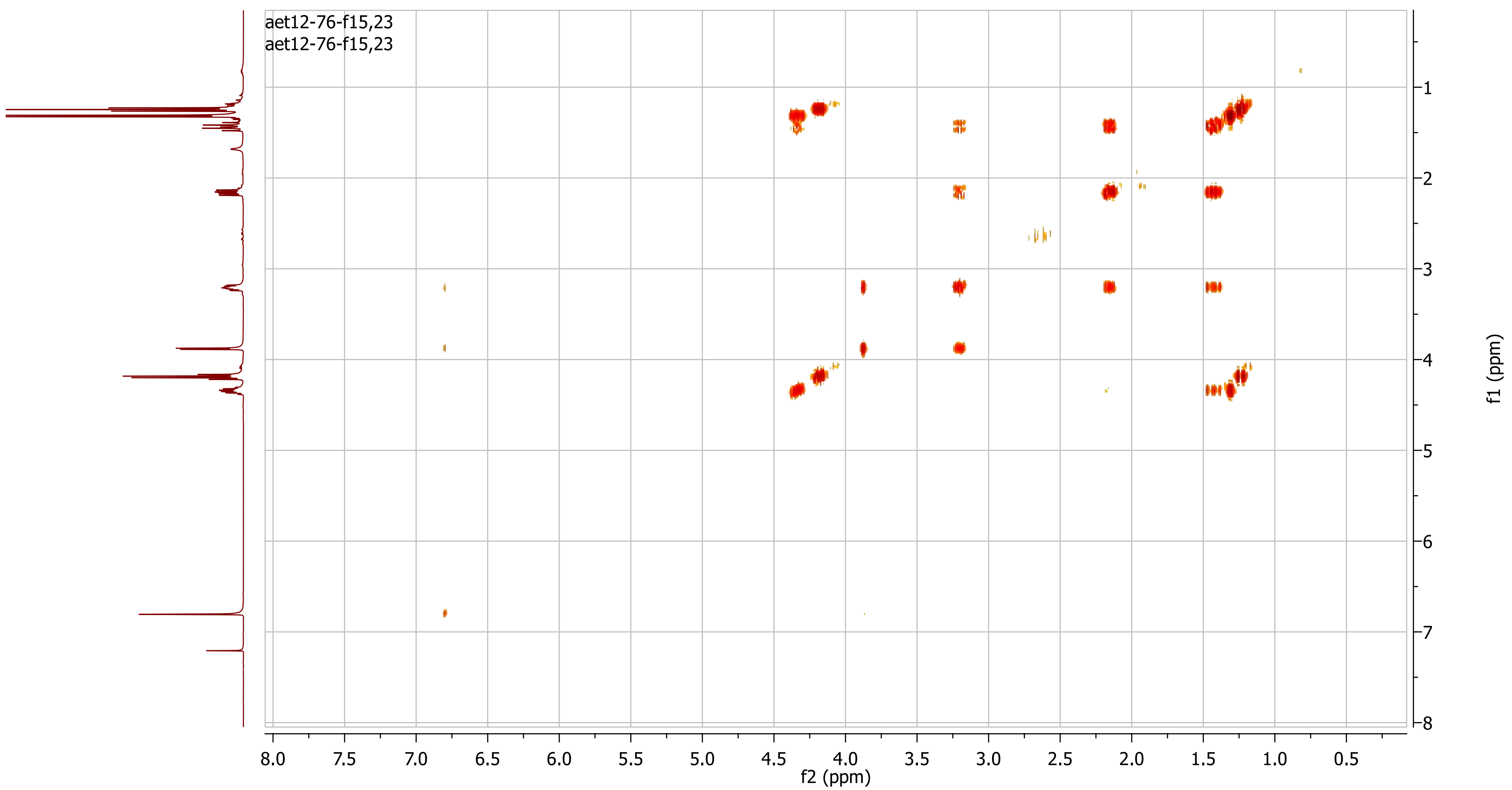

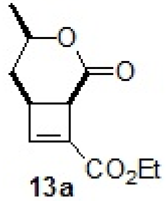

13a

HMQC (CDCl3)

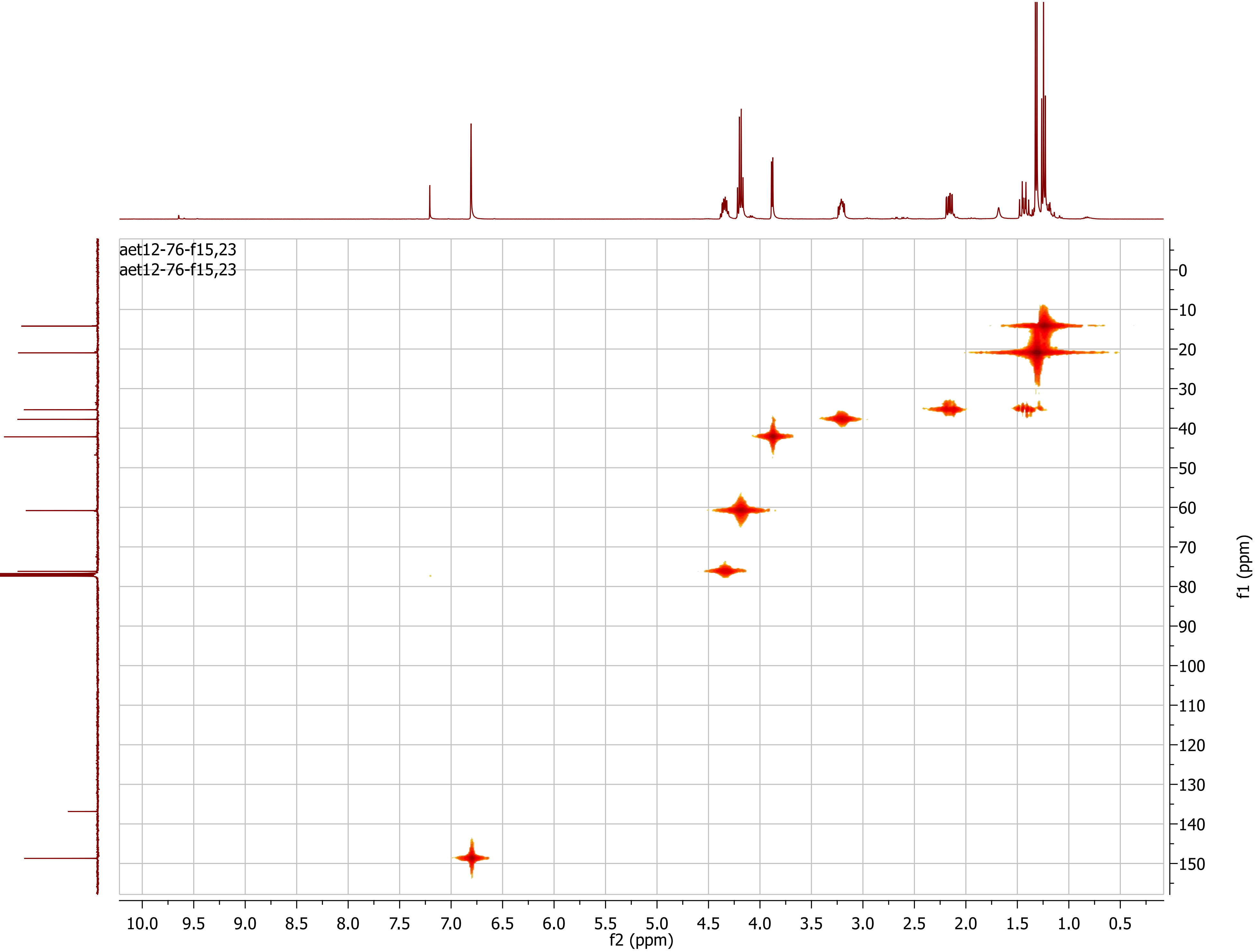

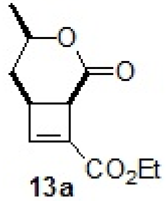

13a

NOESY (400 MHz, CDCl3)

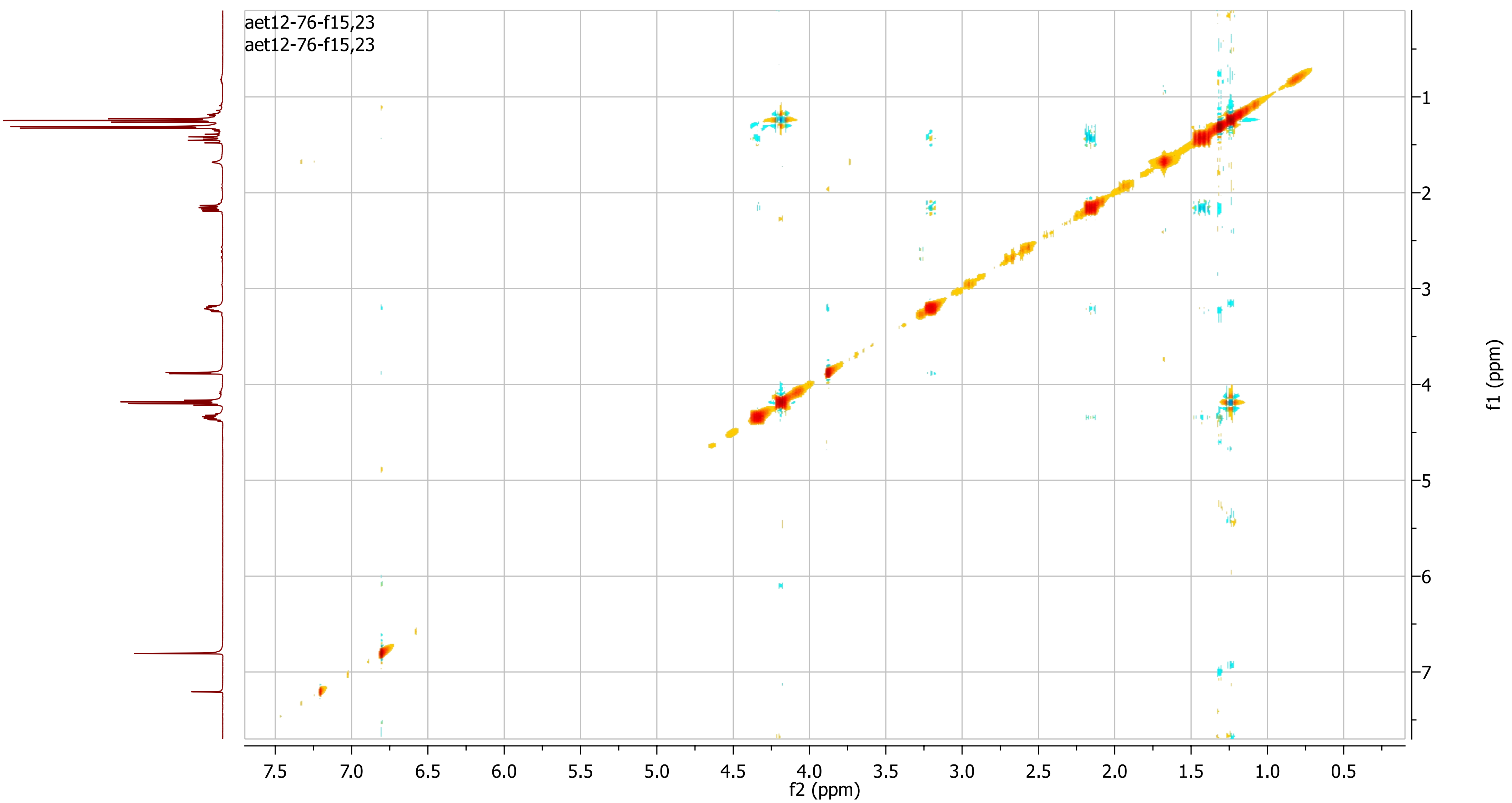

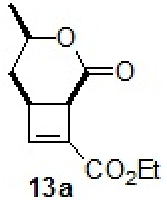

13a

NOESY (400 MHz, CDCl3)

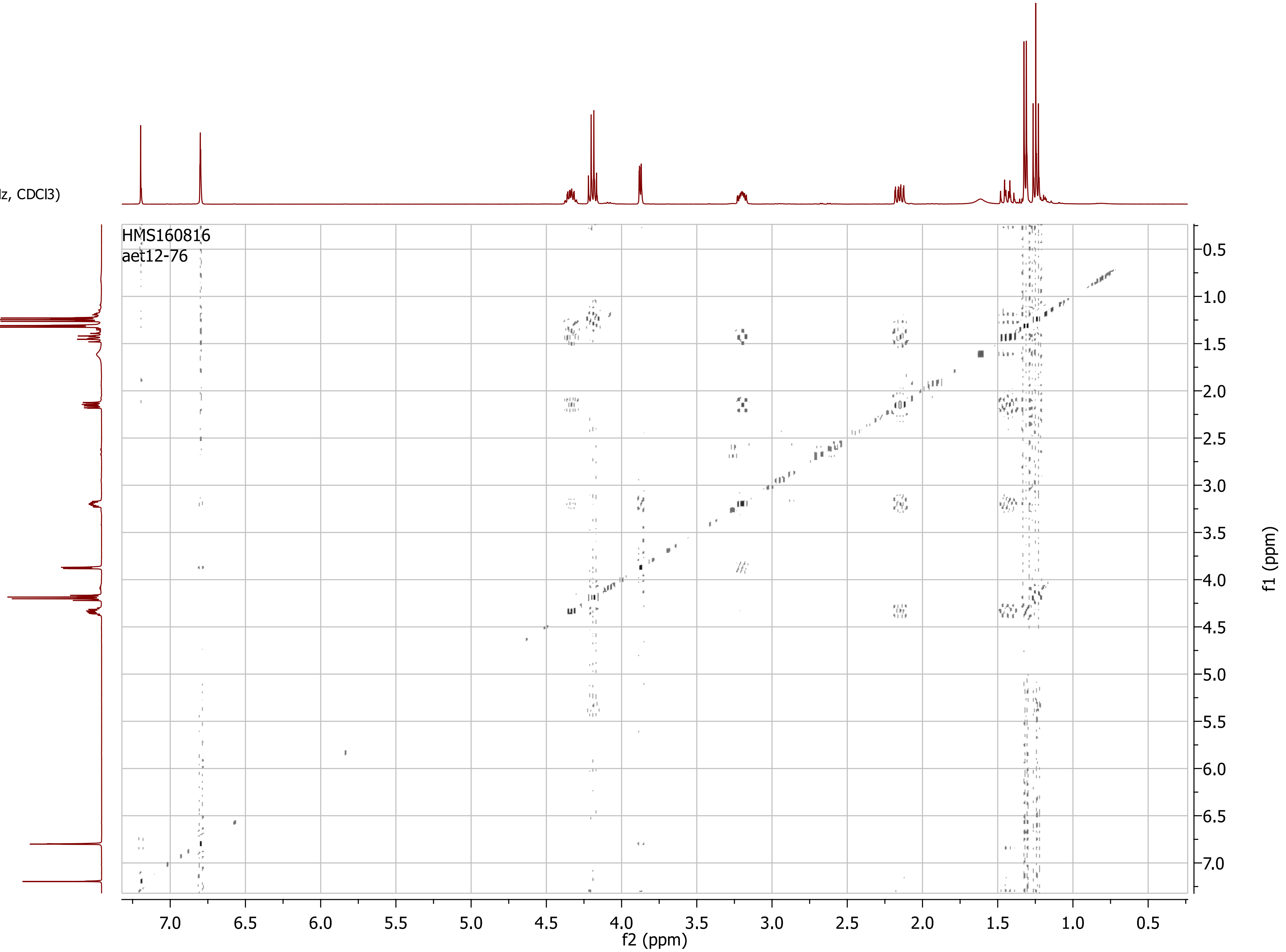

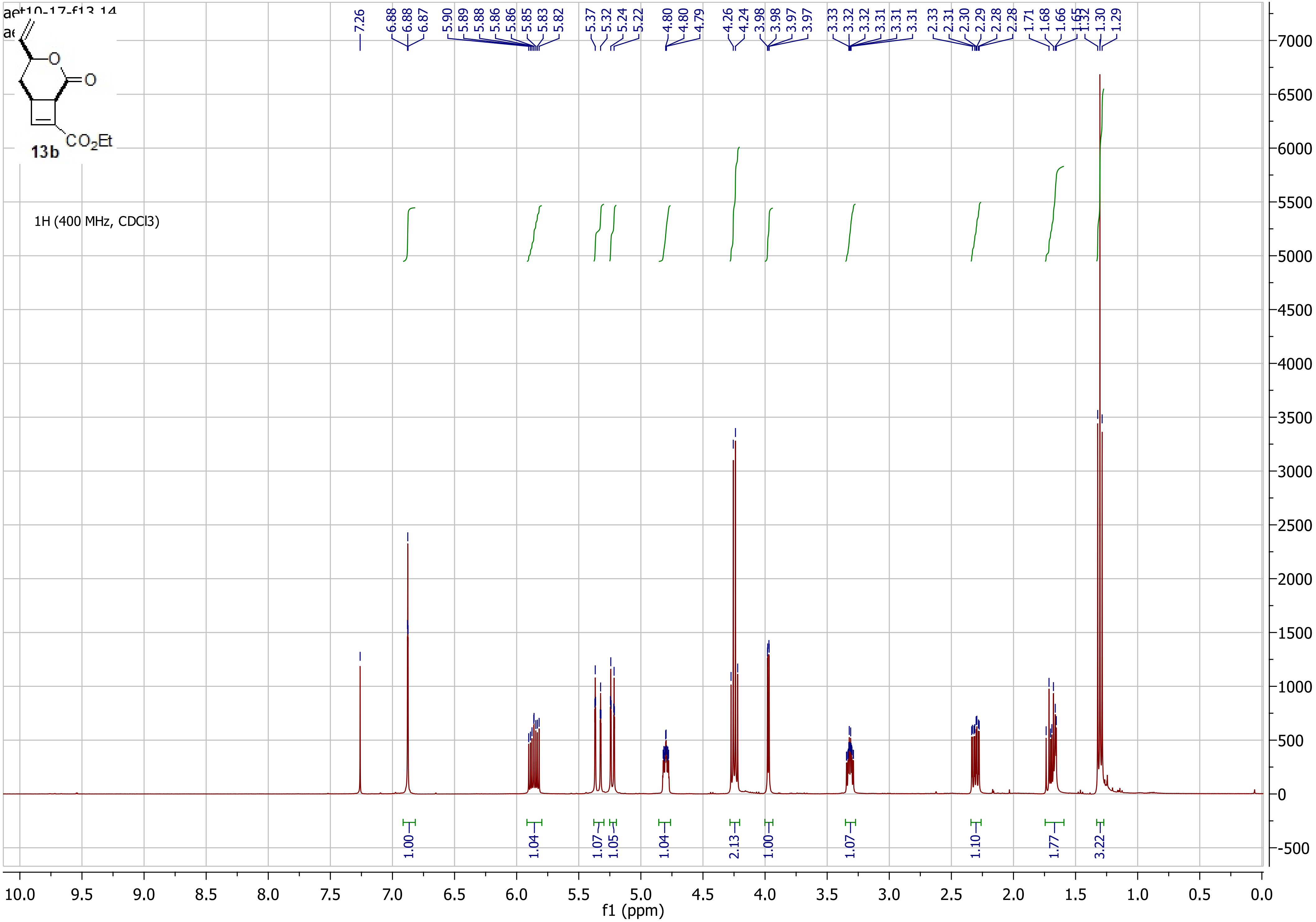

13b
CO_2Et
1H (400 MHz, CDCl3)
7.26
6.88
6.88
6.87
5.90
5.89
5.88
5.86
5.86
5.85
5.83
5.82
5.37
5.32
5.24
5.22
4.80
4.80
4.79
4.26
4.24
3.98
3.98
3.97
3.97
3.33
3.32
3.32
3.31
3.31
3.31
2.33
2.31
2.30
2.29
2.28
2.28
1.71
1.68
1.66
1.65
1.32
1.30
1.29
1.00
1.04
1.07
1.05
1.04
2.13
1.00
1.07
1.10
1.77
3.22
f1 (ppm)

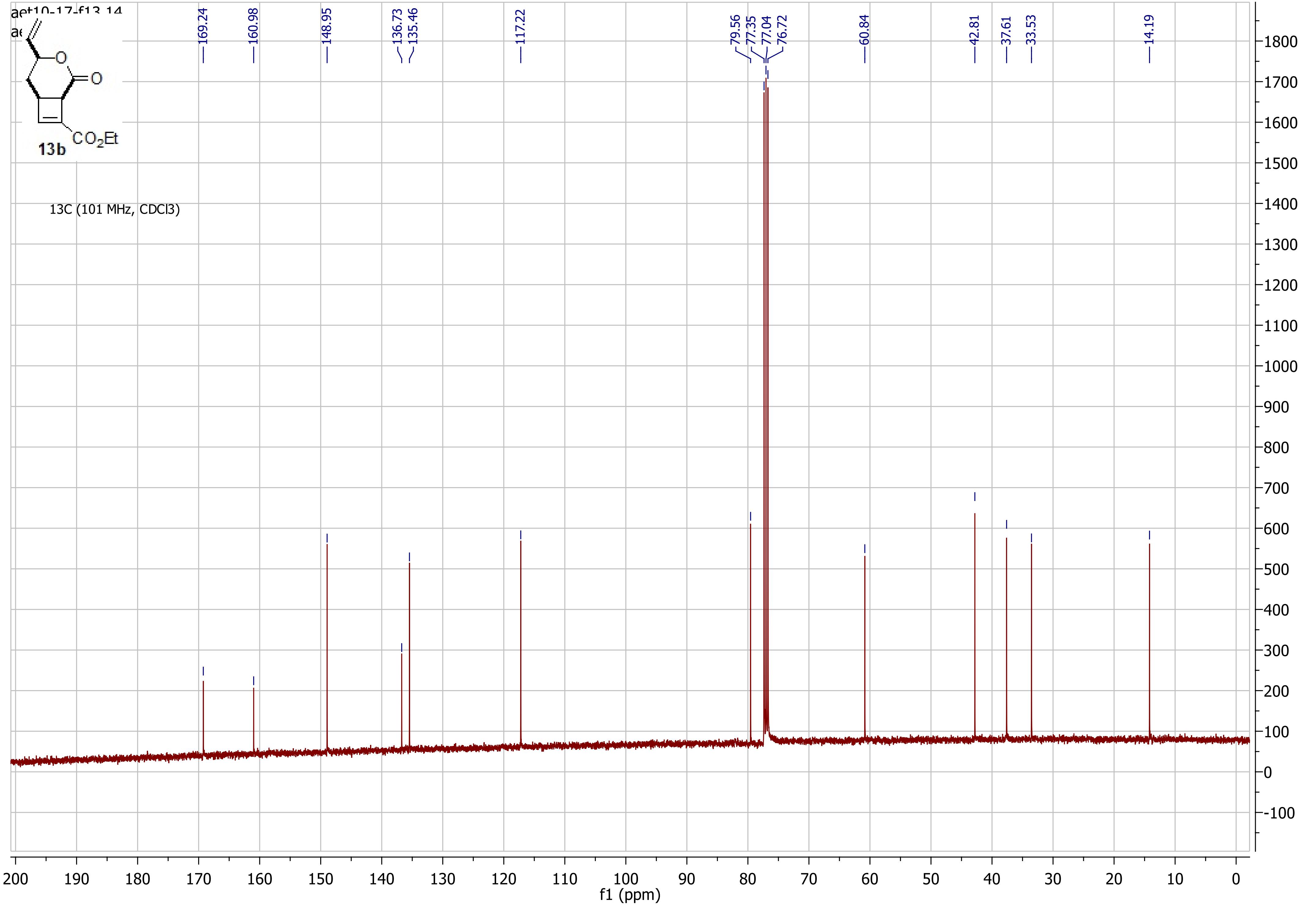

13b
CO2Et
O
13C (101 MHz, CDCl3)
169.24
160.98
148.95
136.73
135.46
117.22
79.56
77.35
77.04
76.72
60.84
42.81
37.61
33.53
14.19
f1 (ppm)

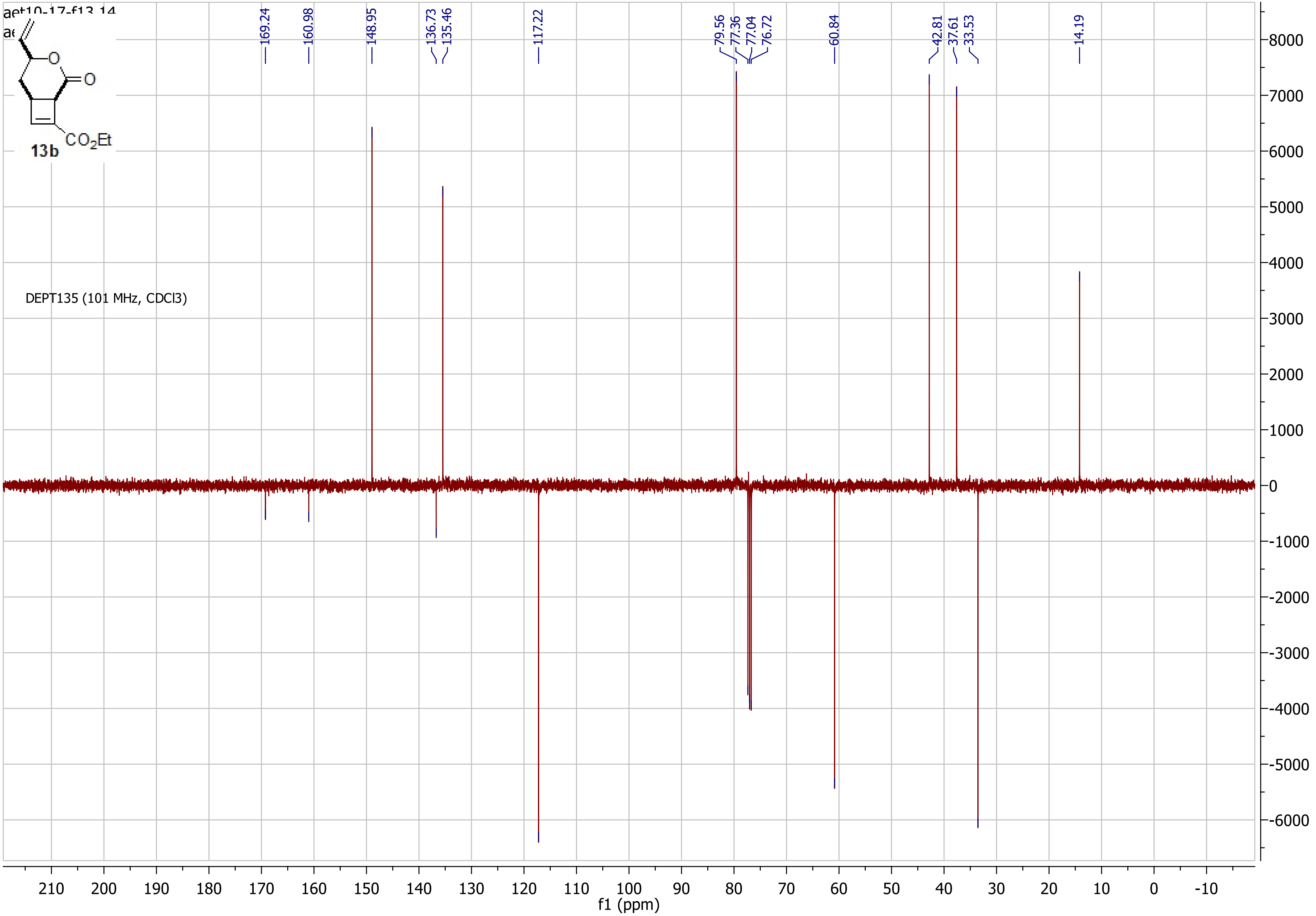
13b
CO2Et
DEPT135 (101 MHz, CDCl3)
169.24
160.98
148.95
136.73
135.46
117.22
79.56
77.36
77.04
76.72
60.84
42.81
37.61
33.53
14.19
f1 (ppm)

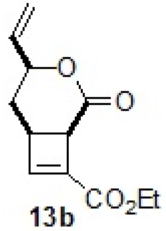

13b

COSY (400 MHz, CDCl3)

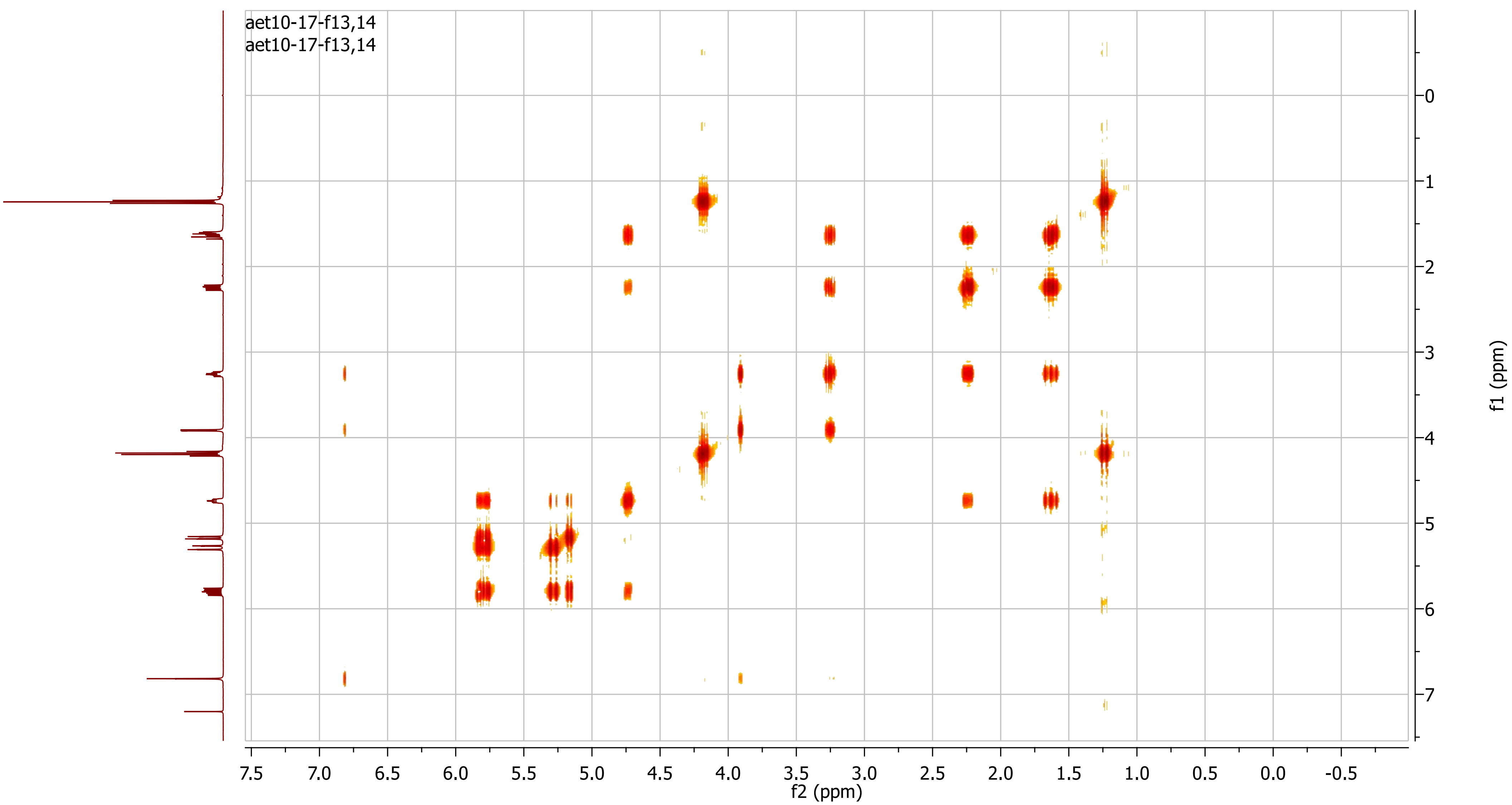

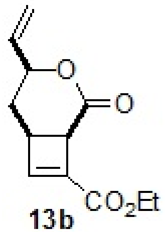

13b

HMQC (CDCl3)

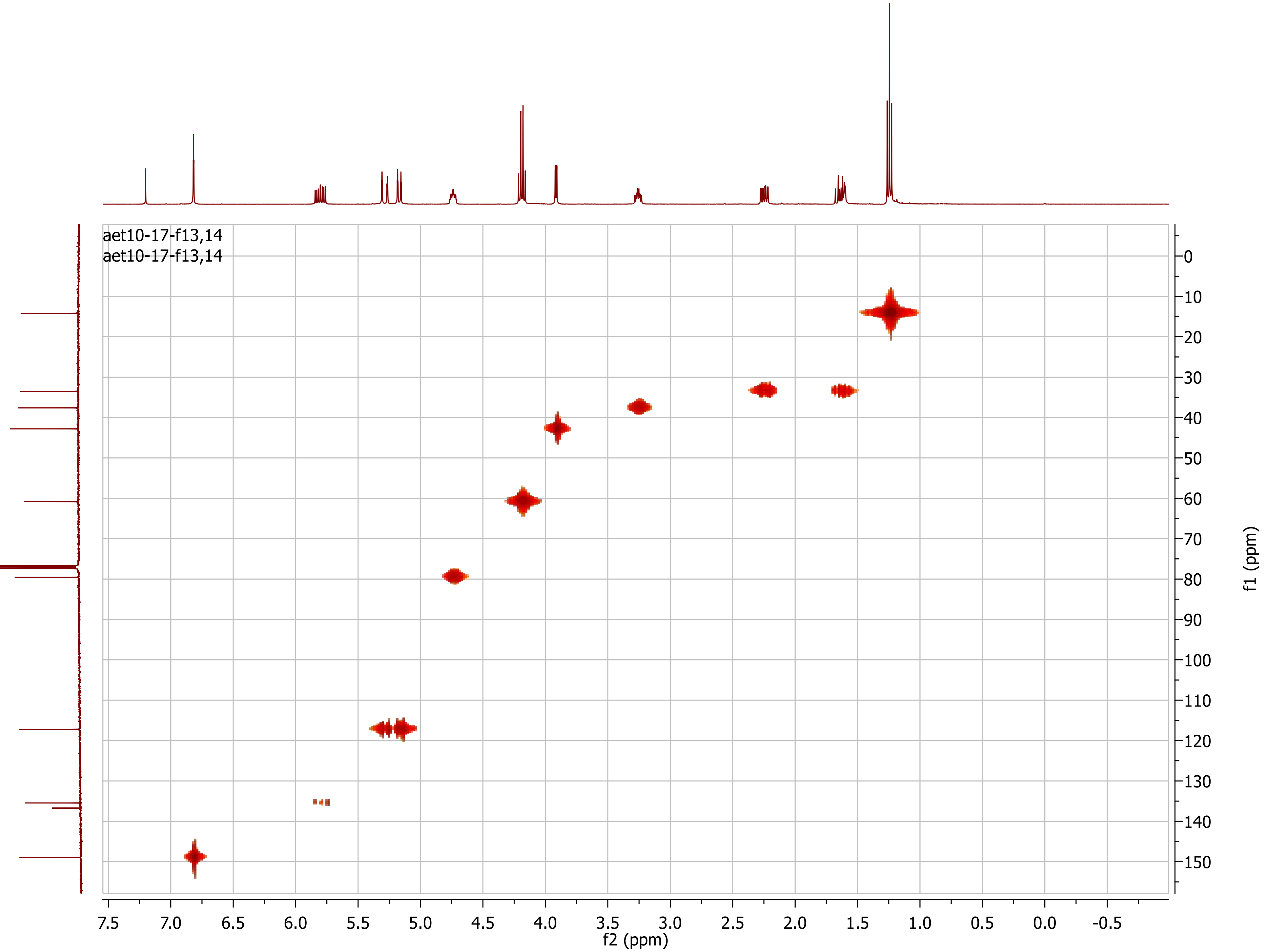

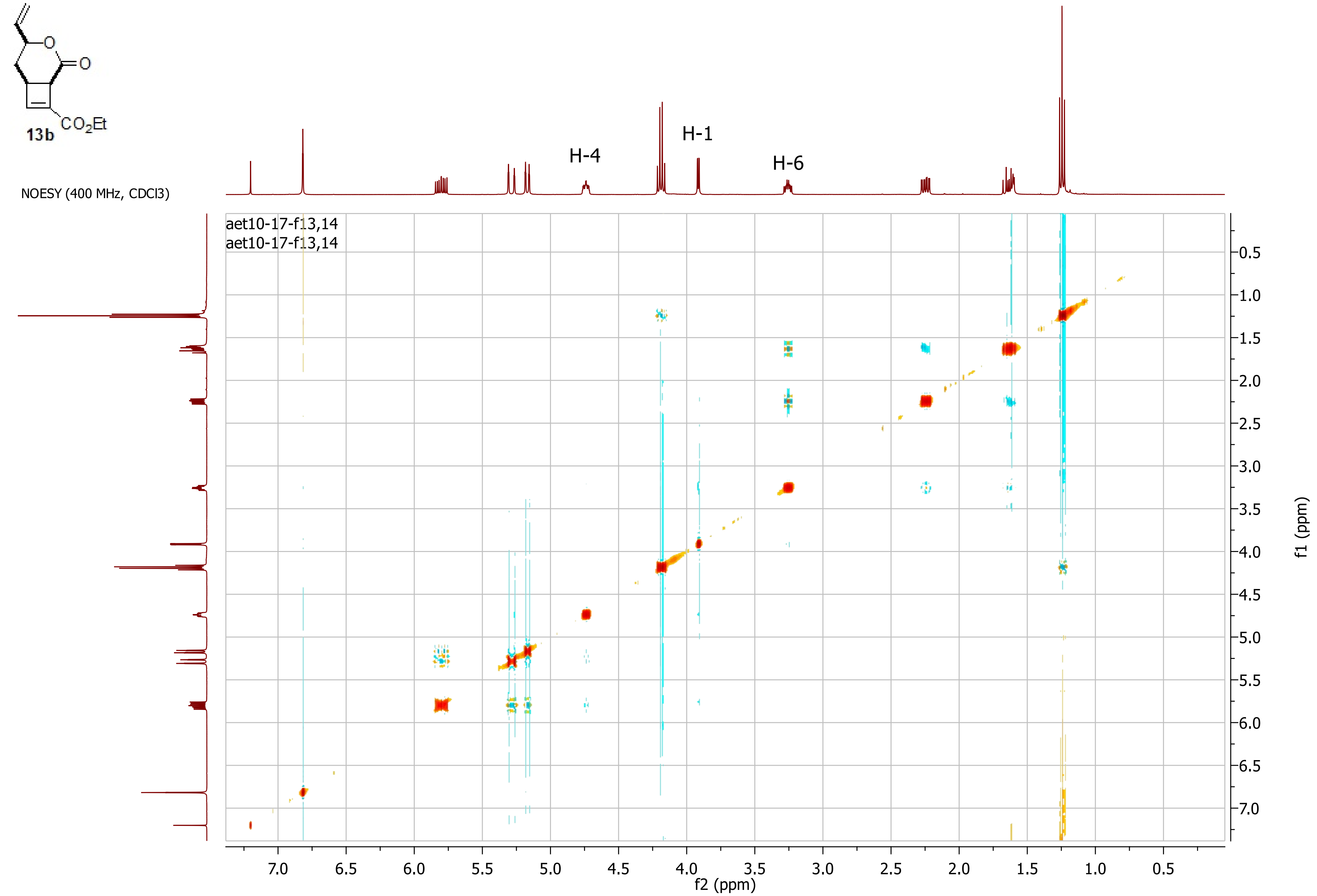

O
O
CO2Et
13b
NOESY (400 MHz, CDCl3)
H-4
H-1
H-6
aet10-17-f13,14
aet10-17-f13,14
f2 (ppm)
f1 (ppm)

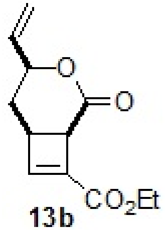

13b

NOESY (400 MHz, CDCl3)

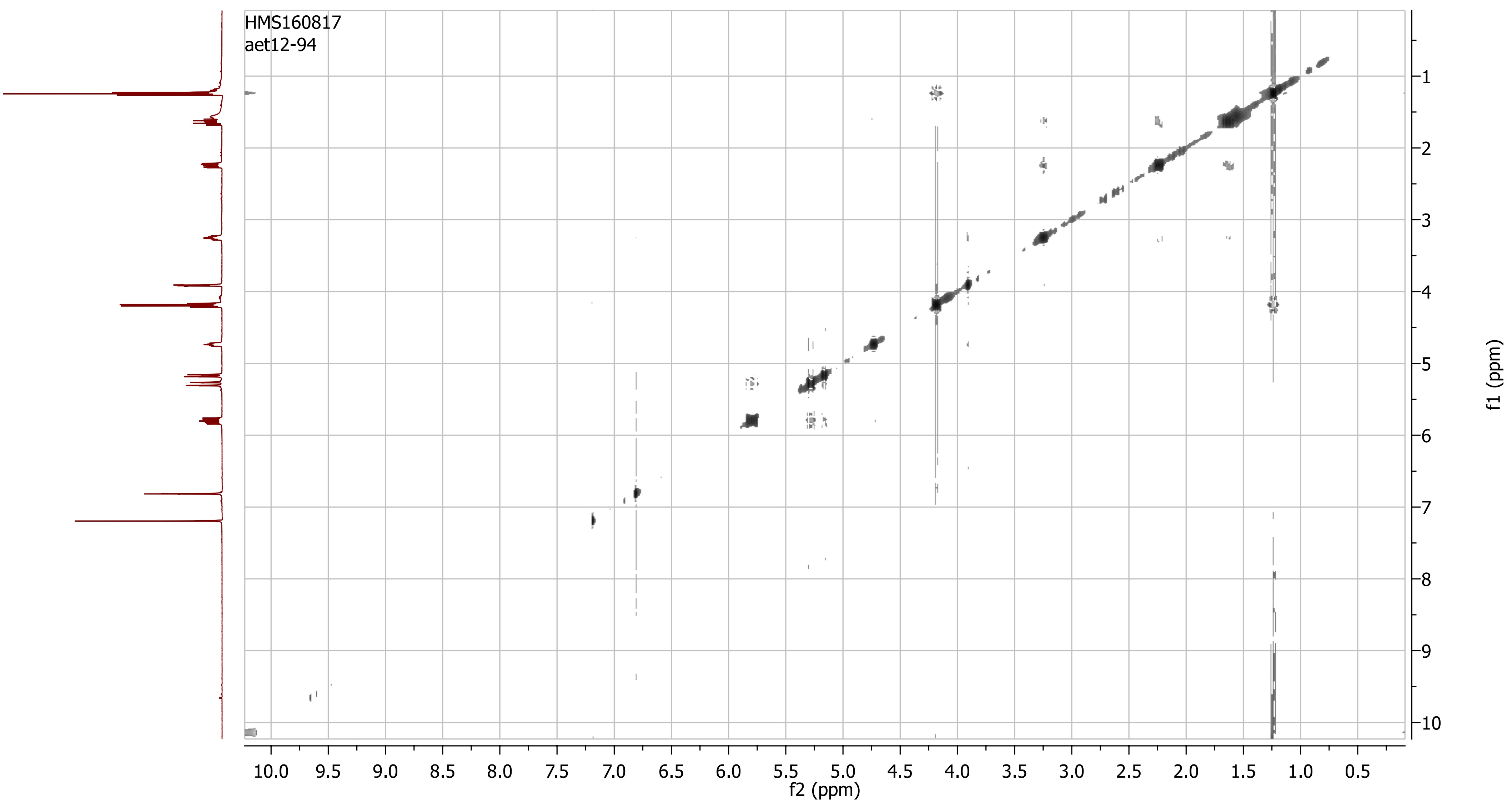

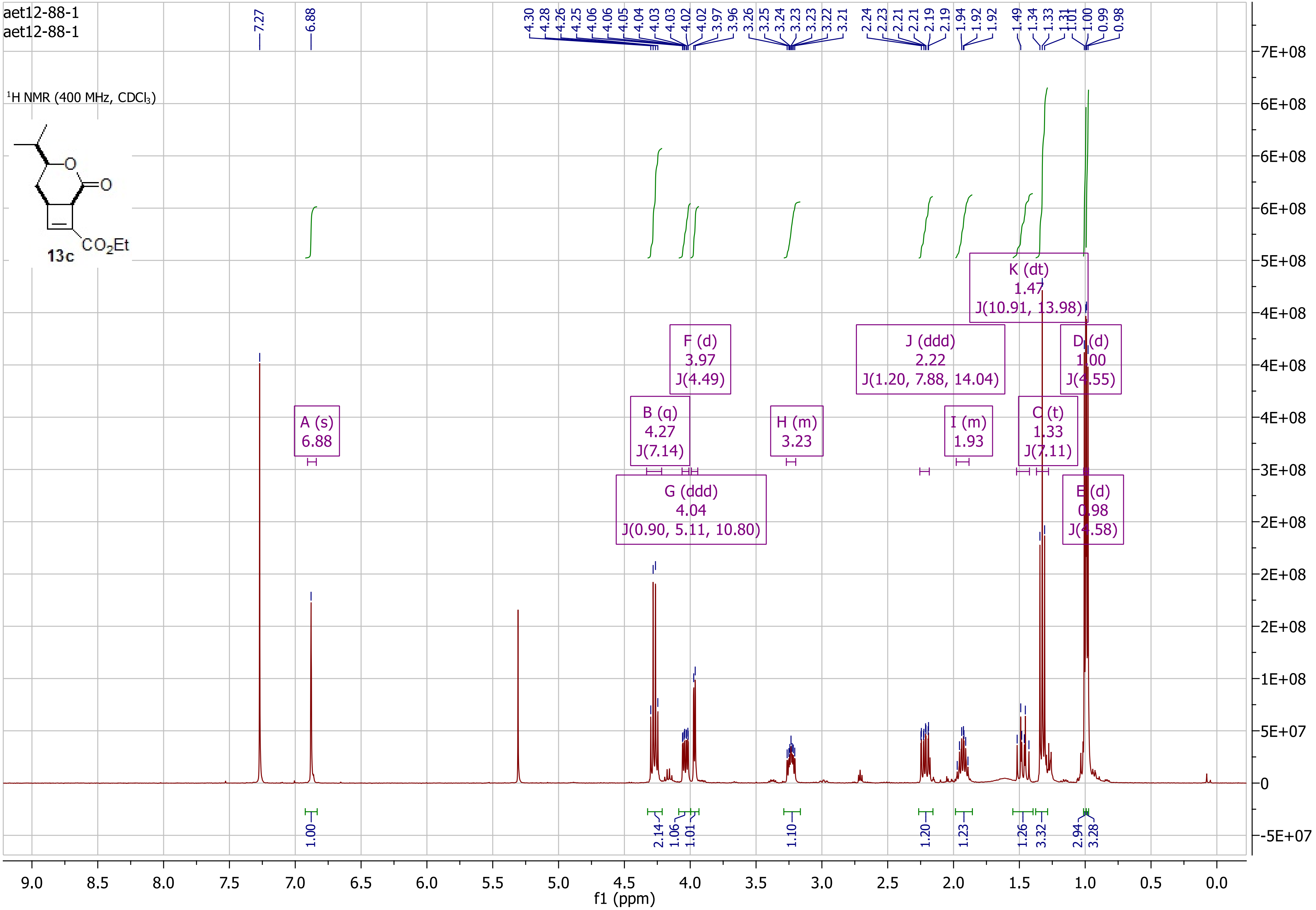

aet12-88-1
aet12-88-1
1H NMR (400 MHz, CDCl3)
13c
CO2Et
O
7.27
6.88
4.30
4.28
4.26
4.25
4.06
4.06
4.05
4.04
4.03
4.03
4.02
4.02
3.97
3.96
3.26
3.25
3.24
3.23
3.23
3.22
3.21
2.24
2.23
2.21
2.21
2.19
2.19
1.94
1.92
1.92
1.49
1.34
1.33
1.31
1.01
1.00
0.99
0.98
A (s)
6.88
B (q)
4.27
J(7.14)
F (d)
3.97
J(4.49)
G (ddd)
4.04
J(0.90, 5.11, 10.80)
H (m)
3.23
J (ddd)
2.22
J(1.20, 7.88, 14.04)
I (m)
1.93
K (dt)
1.47
J(10.91, 13.98)
C (t)
1.33
J(7.11)
D (d)
1.00
J(4.55)
E (d)
0.98
J(4.58)
1.00
2.14
1.06
1.01
1.10
1.20
1.23
1.26
3.32
2.94
3.28
7E+08
6E+08
6E+08
6E+08
5E+08
4E+08
4E+08
4E+08
3E+08
2E+08
2E+08
2E+08
1E+08
5E+07
0
-5E+07
9.0
8.5
8.0
7.5
7.0
6.5
6.0
5.5
5.0
4.5
4.0
3.5
3.0
2.5
2.0
1.5
1.0
0.5
0.0
f1 (ppm)

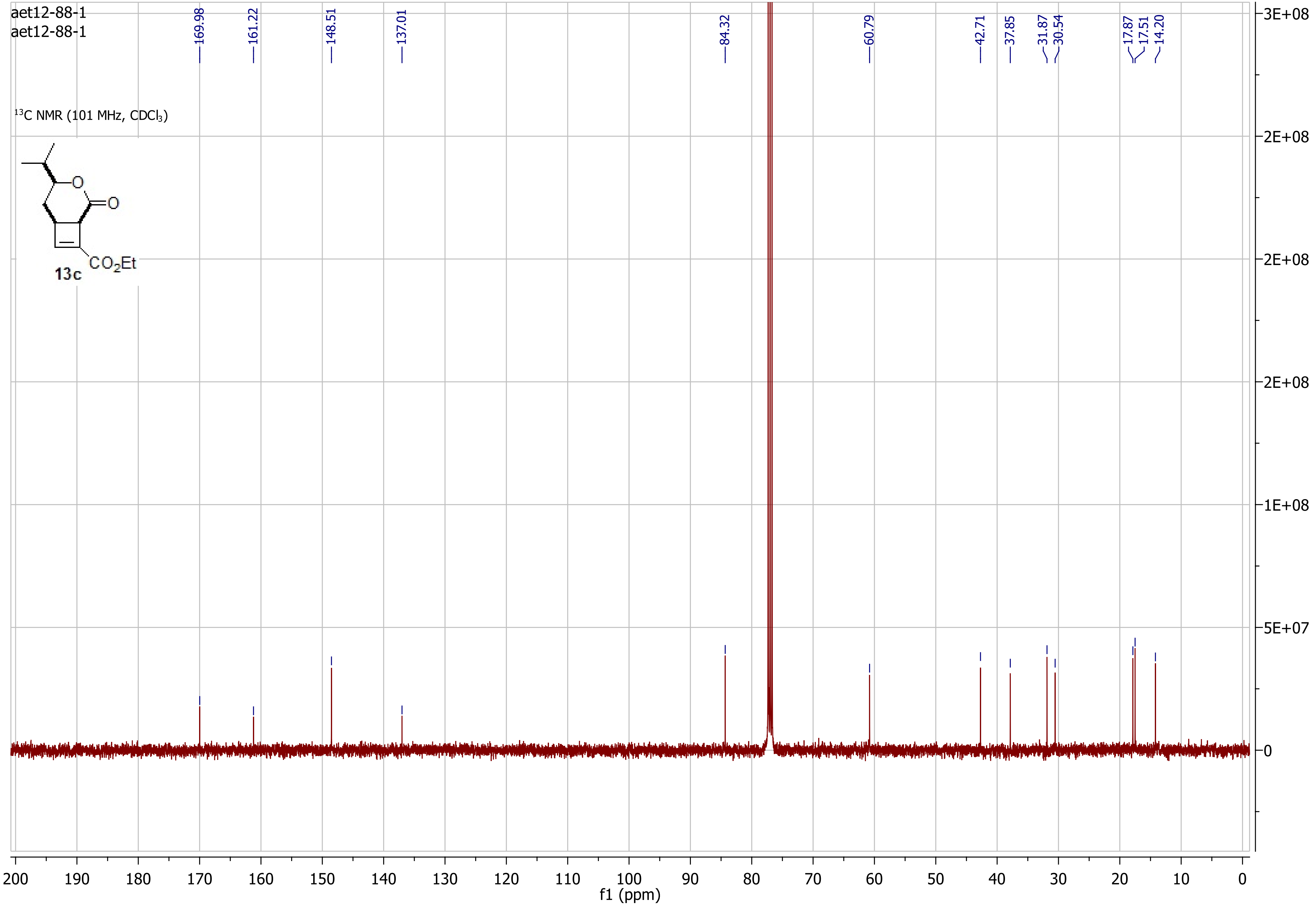
aet12-88-1
aet12-88-1
169.98
161.22
148.51
137.01
84.32
60.79
42.71
37.85
31.87
30.54
17.87
17.51
14.20
13C NMR (101 MHz, CDCl3)
CO2Et
13c
3E+08
2E+08
2E+08
2E+08
1E+08
5E+07
0
200
190
180
170
160
150
140
130
120
110
100
90
80
70
60
50
40
30
20
10
0
f1 (ppm)

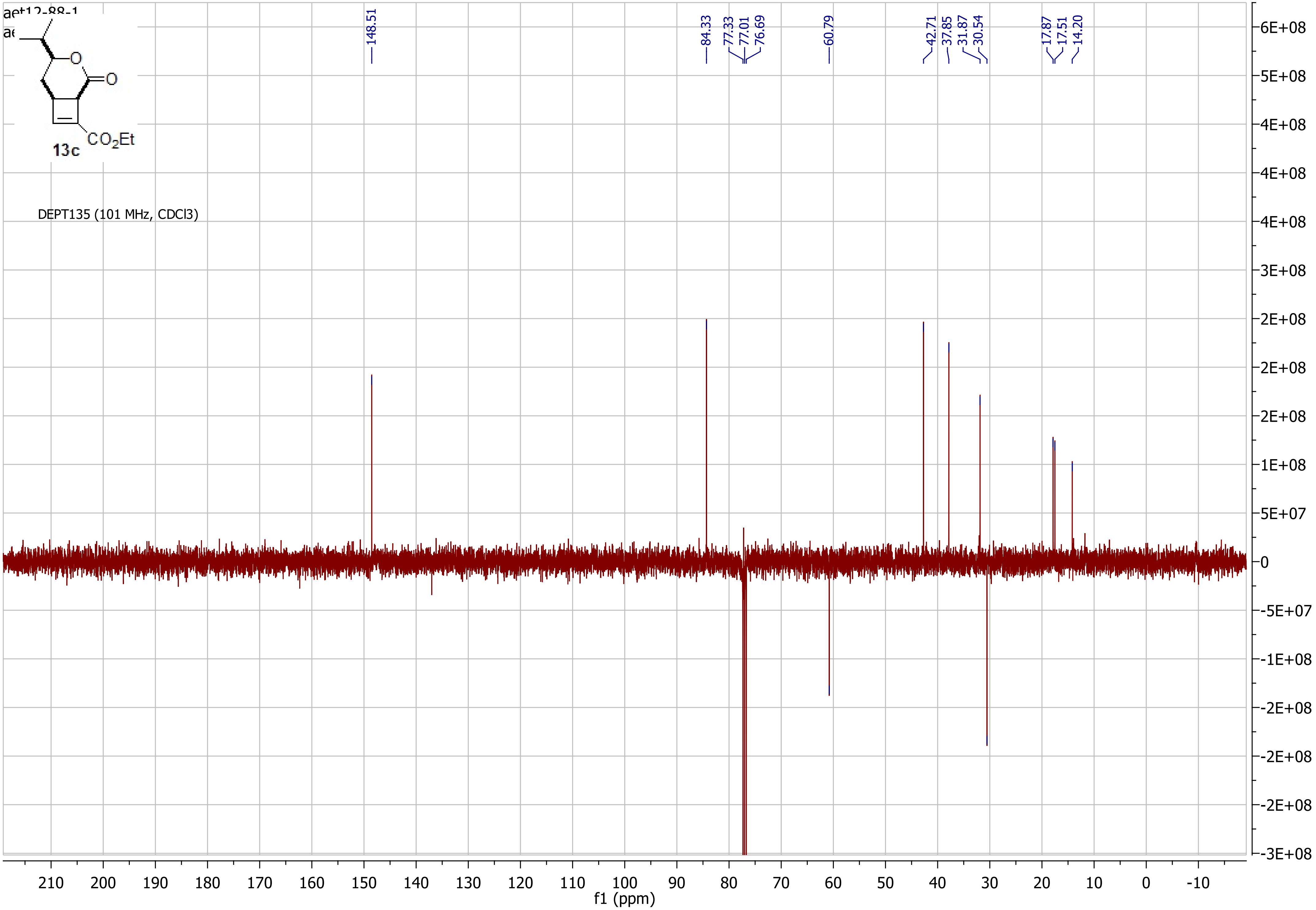

DEPT135 (101 MHz, CDCl3)
13c
CO2Et
148.51
84.33
77.33
77.01
76.69
60.79
42.71
37.85
31.87
30.54
17.87
17.51
14.20
f1 (ppm)
210 200 190 180 170 160 150 140 130 120 110 100 90 80 70 60 50 40 30 20 10 0 -10
6E+08
5E+08
4E+08
4E+08
4E+08
3E+08
2E+08
2E+08
2E+08
1E+08
5E+07
0
-5E+07
-1E+08
-2E+08
-2E+08
-2E+08
-3E+08

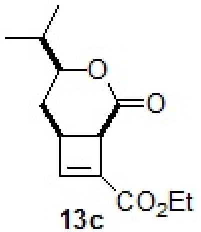

13c

COSY (400 MHz, CDCl3)

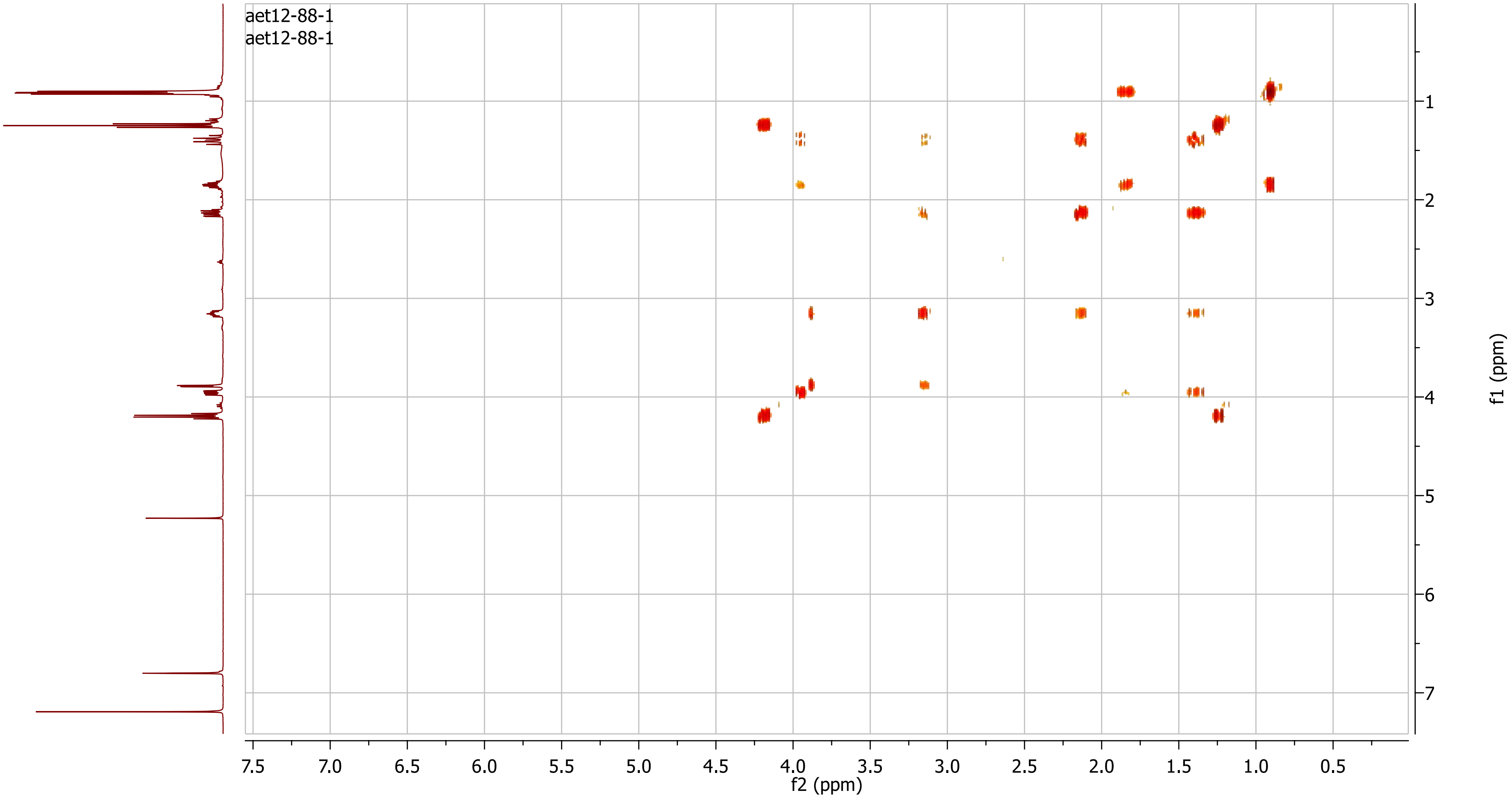

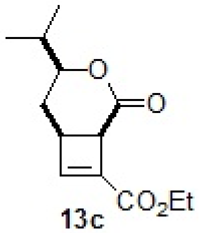

13c

COSY (400 MHz, CDCl3)

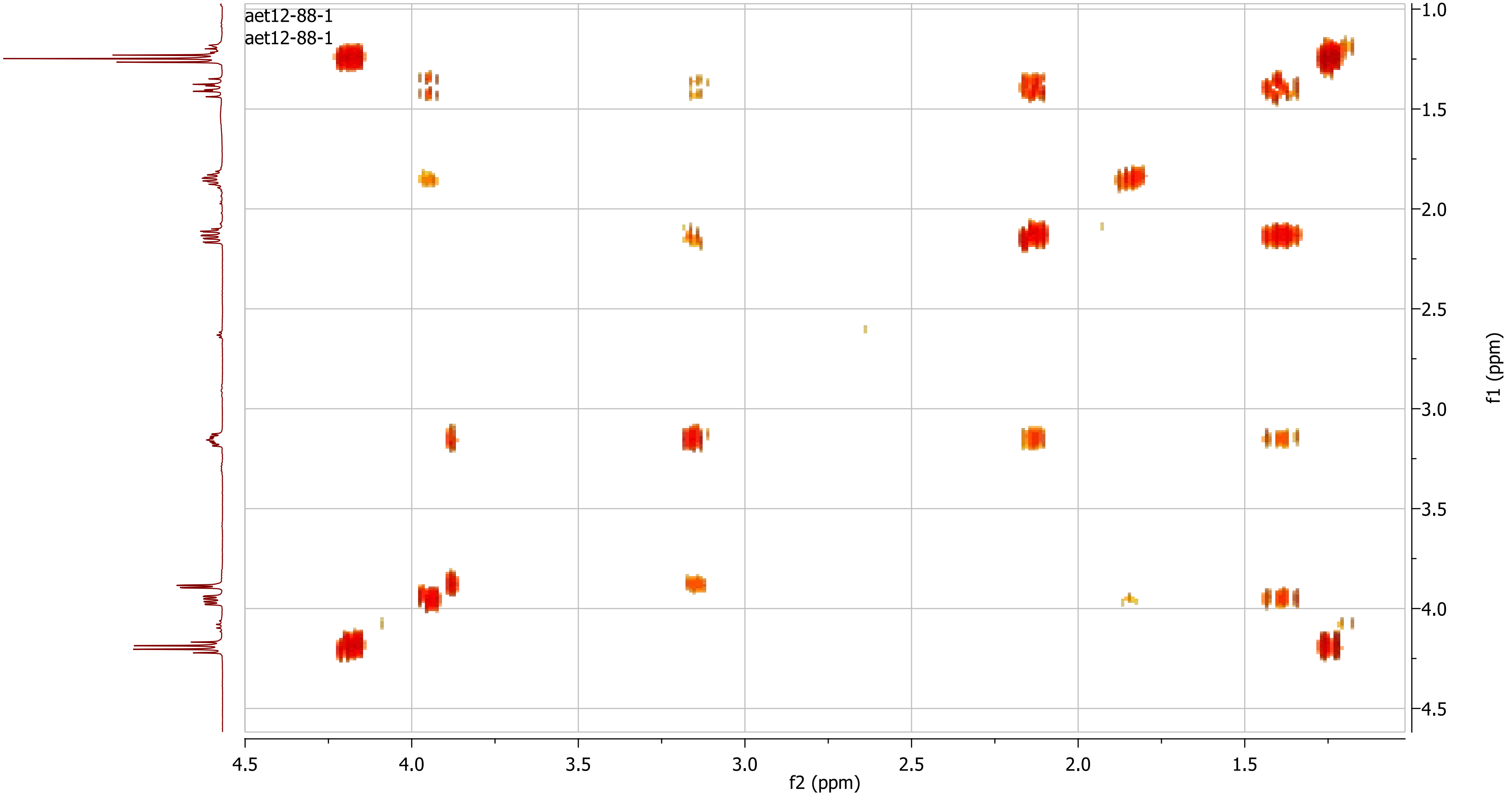

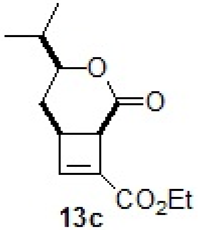

13c

HMQC (CDCl3)

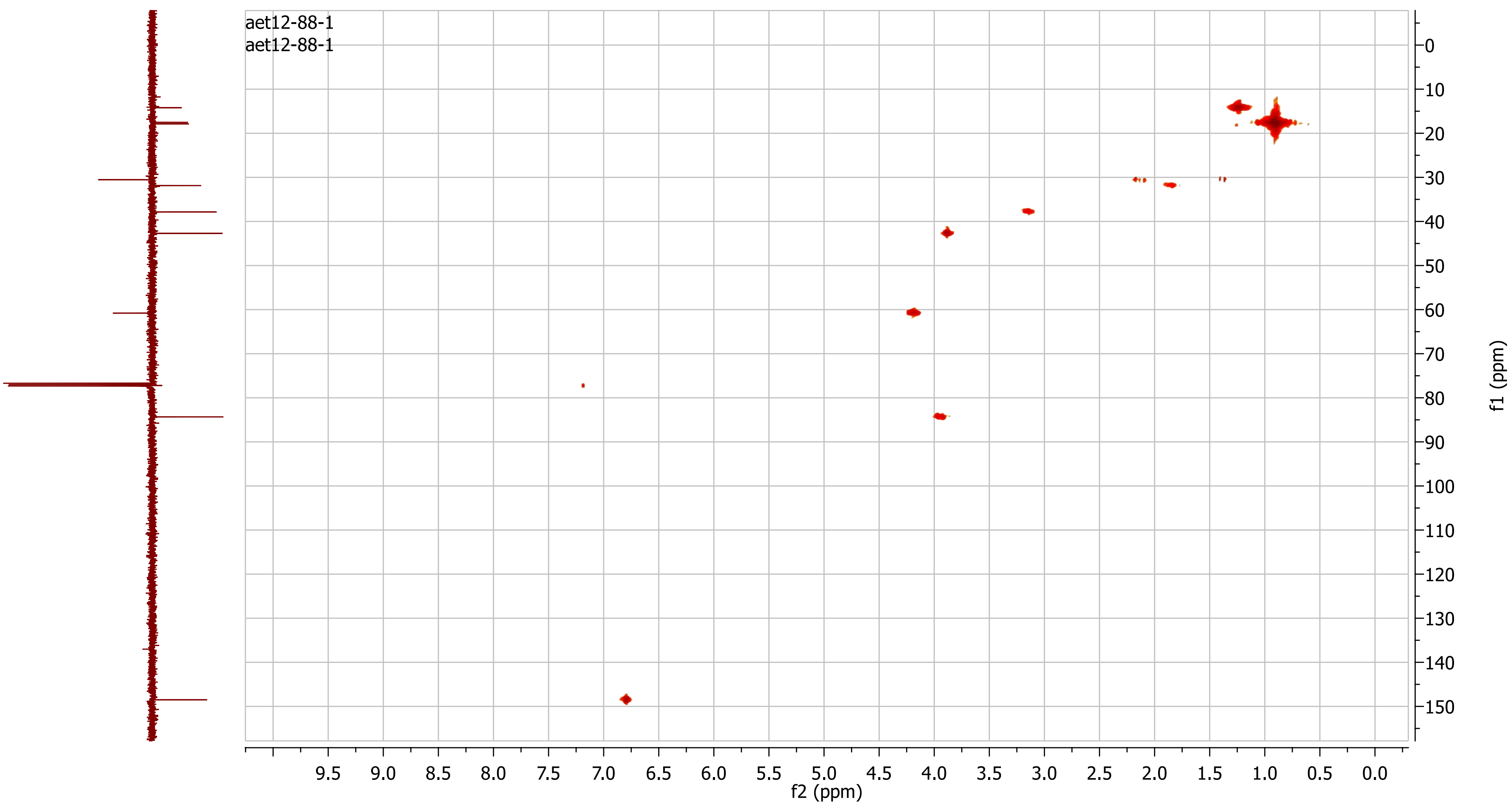

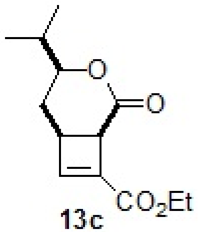

13c

NOESY (400 MHz, CDCl3)

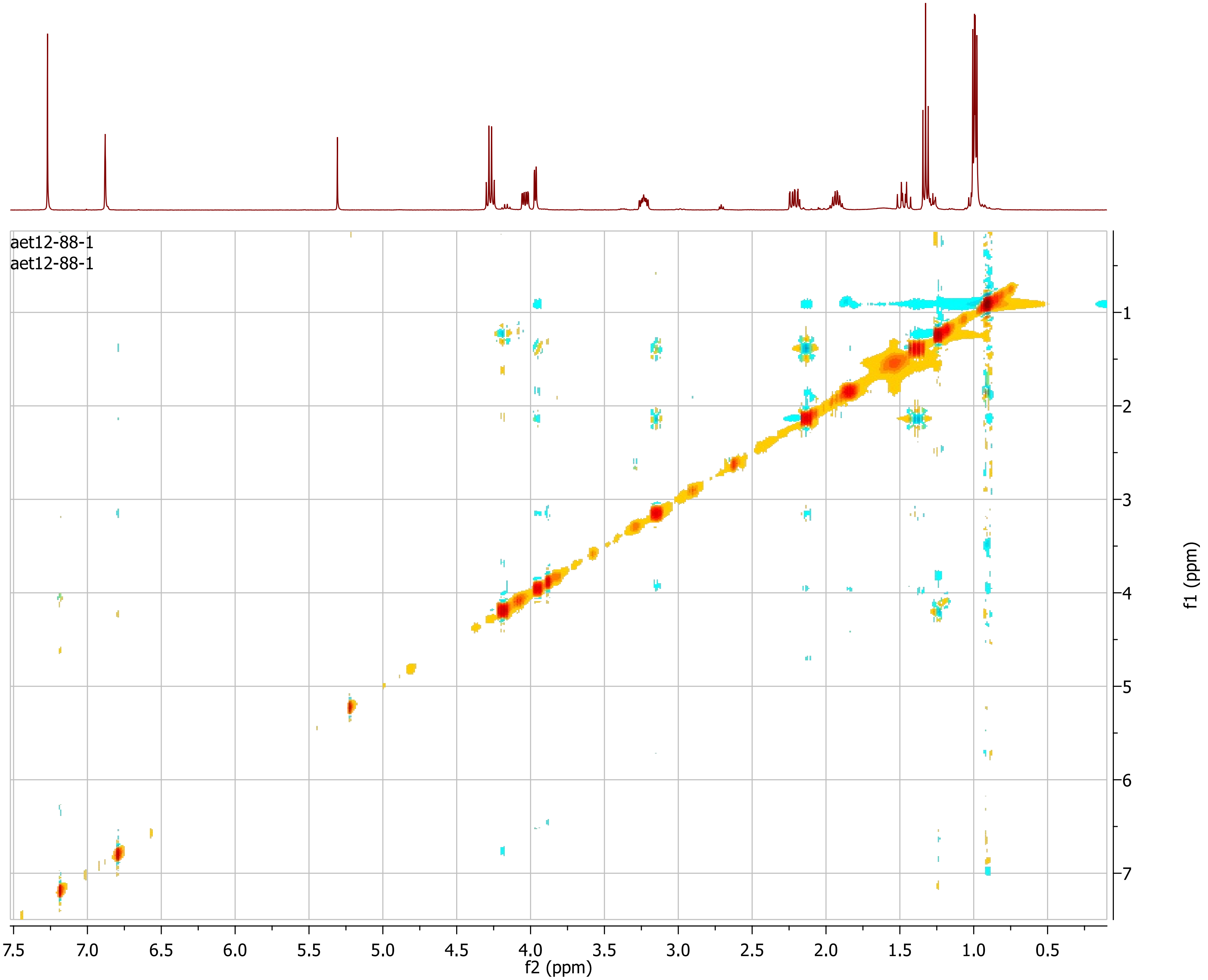

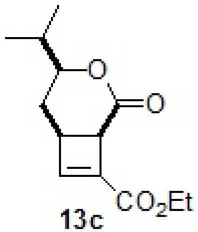

13c

NOESY (400 MHz, CDCl3)

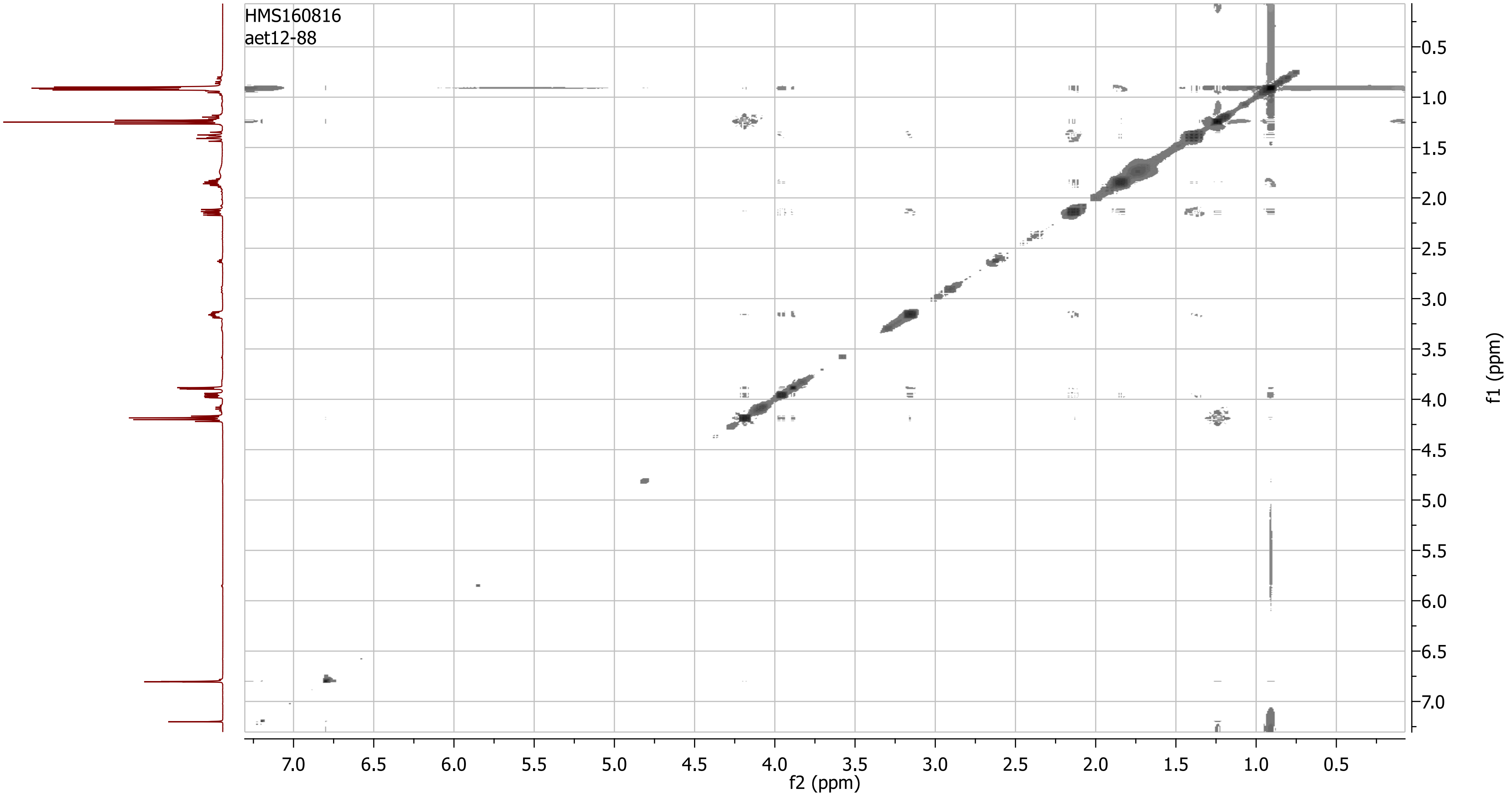

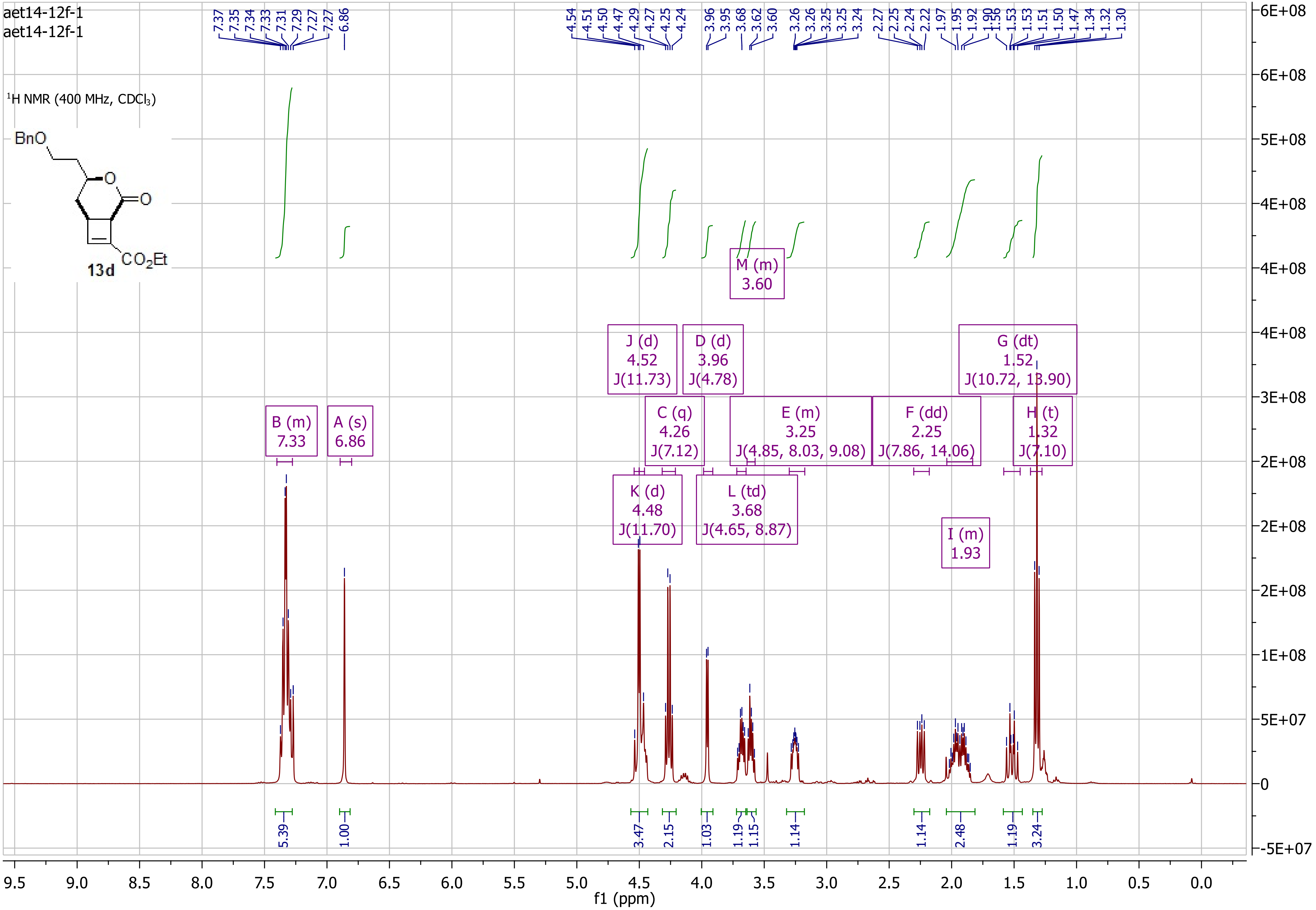
aet14-12f-1
aet14-12f-1
1H NMR (400 MHz, CDCl3)
BnO
O
O
CO2Et
13d
7.37 7.35 7.34 7.33 7.31 7.29 7.27 7.27 6.86
4.54 4.51 4.50 4.47 4.29 4.27 4.25 4.24
3.96 3.95 3.68 3.62 3.60 3.26 3.26 3.25 3.25 3.24
2.27 2.25 2.24 2.22 1.97 1.95 1.92 1.53 1.53 1.51 1.50 1.47 1.34 1.32 1.30
M (m) 3.60
J (d) 4.52 J(11.73)
D (d) 3.96 J(4.78)
G (dt) 1.52 J(10.72, 13.90)
B (m) 7.33
A (s) 6.86
C (q) 4.26 J(7.12)
E (m) 3.25 J(4.85, 8.03, 9.08)
F (dd) 2.25 J(7.86, 14.06)
H (t) 1.32 J(7.10)
K (d) 4.48 J(11.70)
L (td) 3.68 J(4.65, 8.87)
I (m) 1.93
5.39 1.00 3.47 2.15 1.03 1.19 1.15 1.14 1.14 2.48 1.19 3.24
9.5 9.0 8.5 8.0 7.5 7.0 6.5 6.0 5.5 5.0 4.5 4.0 3.5 3.0 2.5 2.0 1.5 1.0 0.5 0.0
f1 (ppm)
6E+08 6E+08 5E+08 4E+08 4E+08 4E+08 3E+08 2E+08 2E+08 2E+08 1E+08 5E+07 0 -5E+07

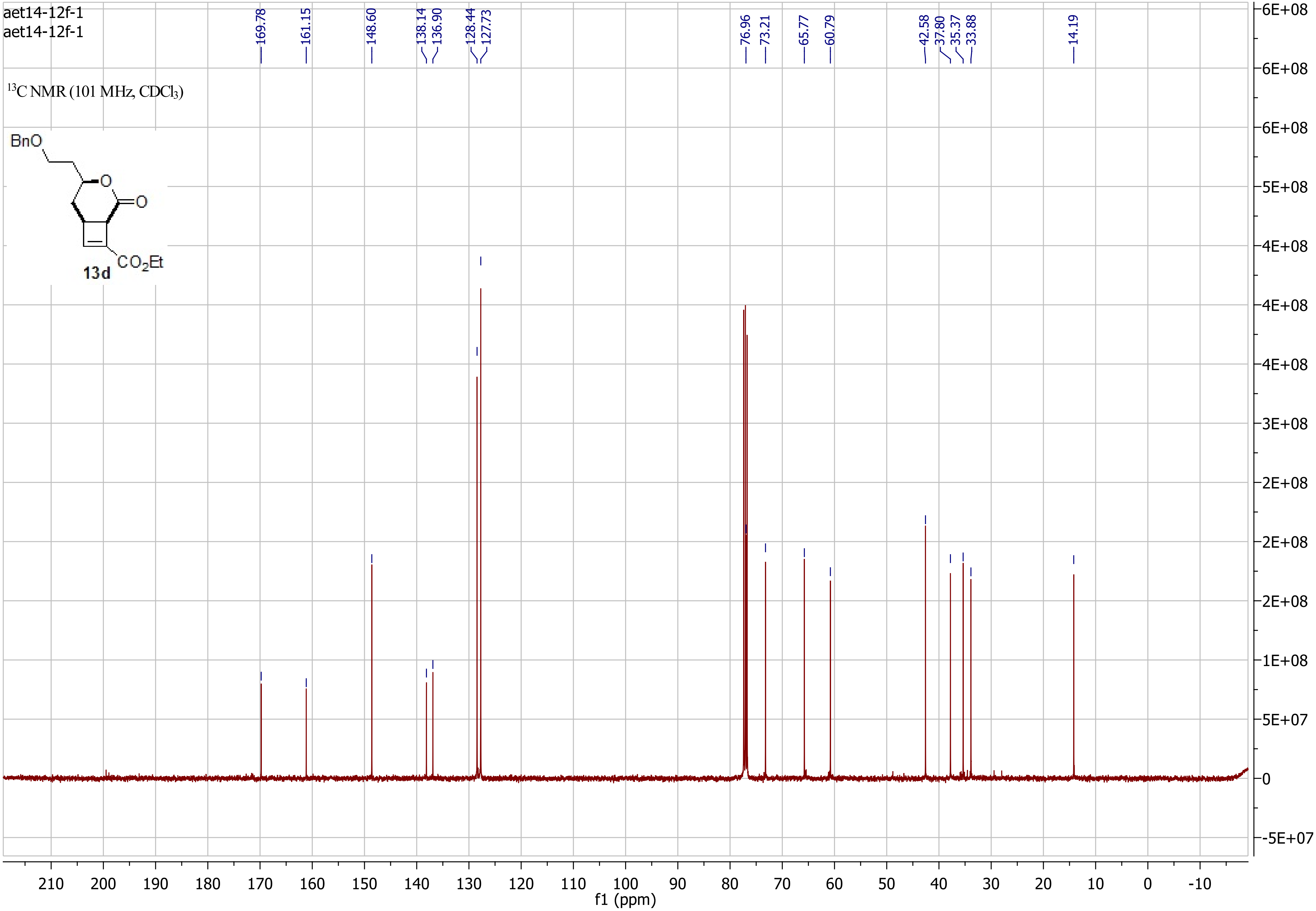
aet14-12f-1
aet14-12f-1
13C NMR (101 MHz, CDCl3)
BnO
O
O
CO2Et
13d
169.78
161.15
148.60
138.14
136.90
128.44
127.73
76.96
73.21
65.77
60.79
42.58
37.80
35.37
33.88
14.19
f1 (ppm)

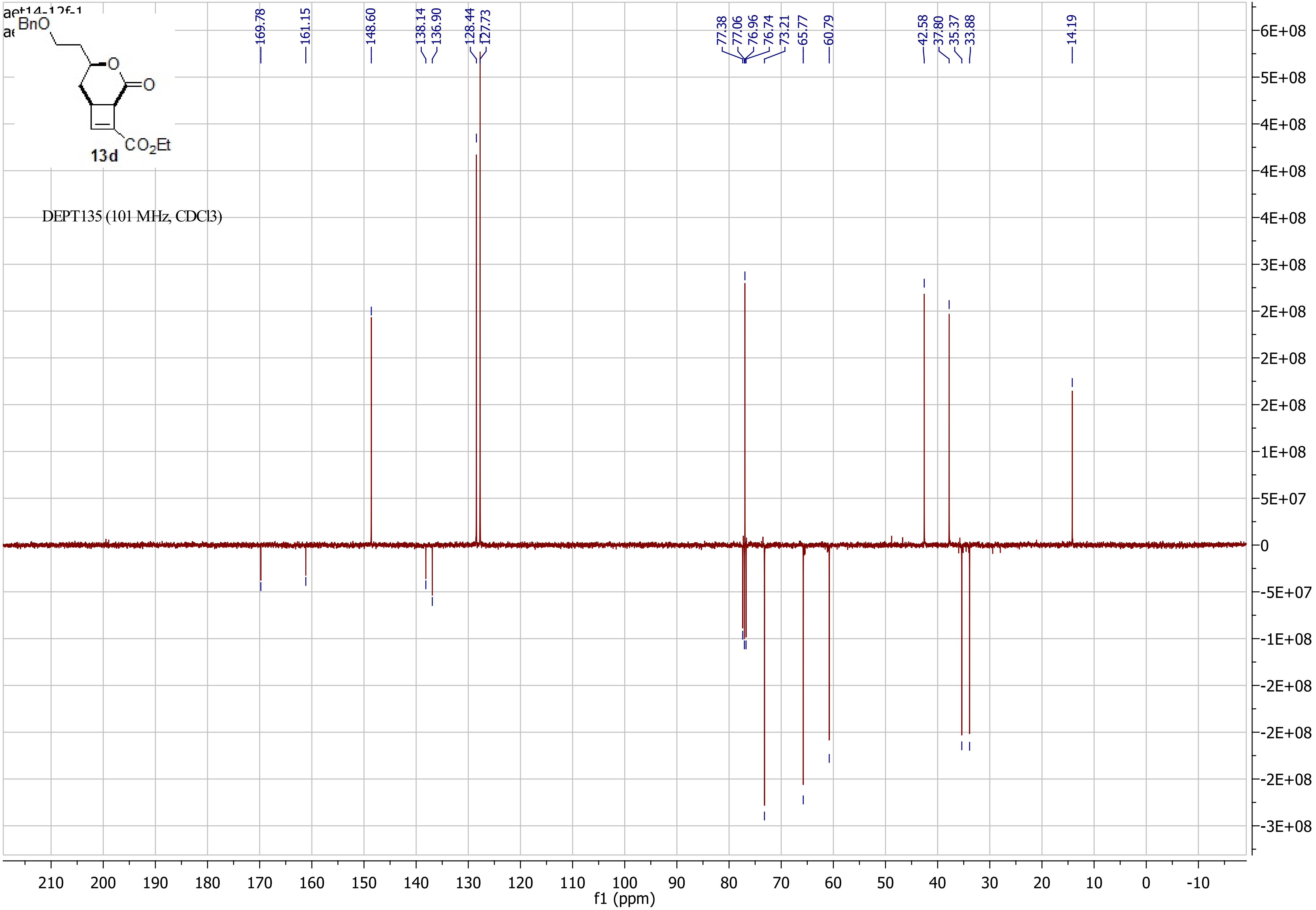

BnO
O
O
CO2Et
13d
DEPT135 (101 MHz, CDCl3)
169.78
161.15
148.60
138.14
136.90
128.44
127.73
77.38
77.06
76.96
76.74
73.21
65.77
60.79
42.58
37.80
35.37
33.88
14.19
6E+08
5E+08
4E+08
4E+08
4E+08
3E+08
2E+08
2E+08
2E+08
1E+08
5E+07
0
-5E+07
-1E+08
-2E+08
-2E+08
-2E+08
-3E+08
210
200
190
180
170
160
150
140
130
120
110
100
90
80
70
60
50
40
30
20
10
0
-10
f1 (ppm)

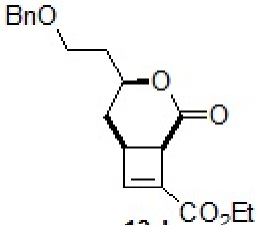

13d

COSY (400 MHz, CDCl3)

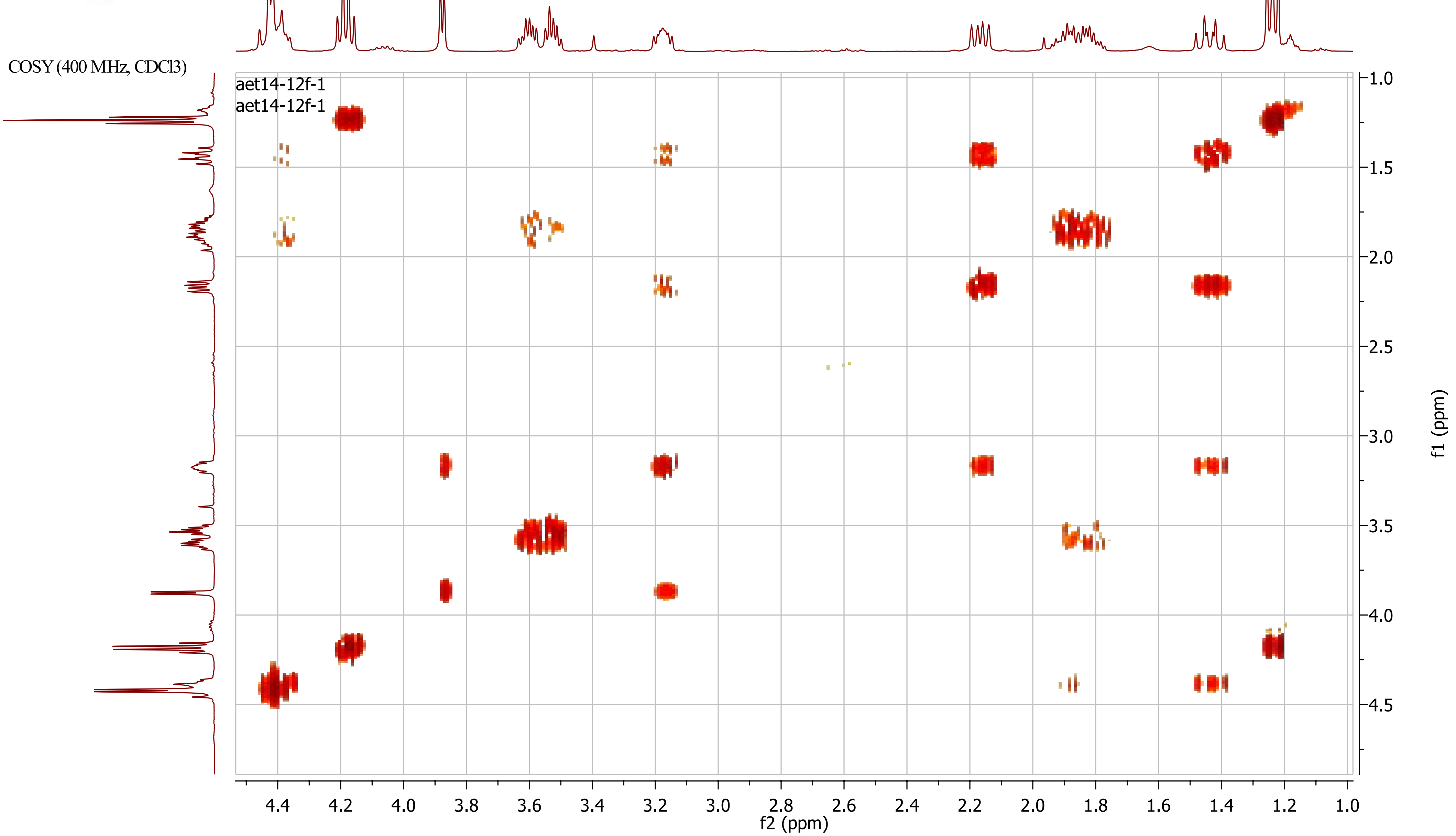

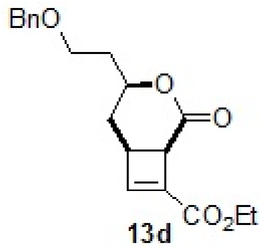

13d

COSY (400 MHz, CDCl3)

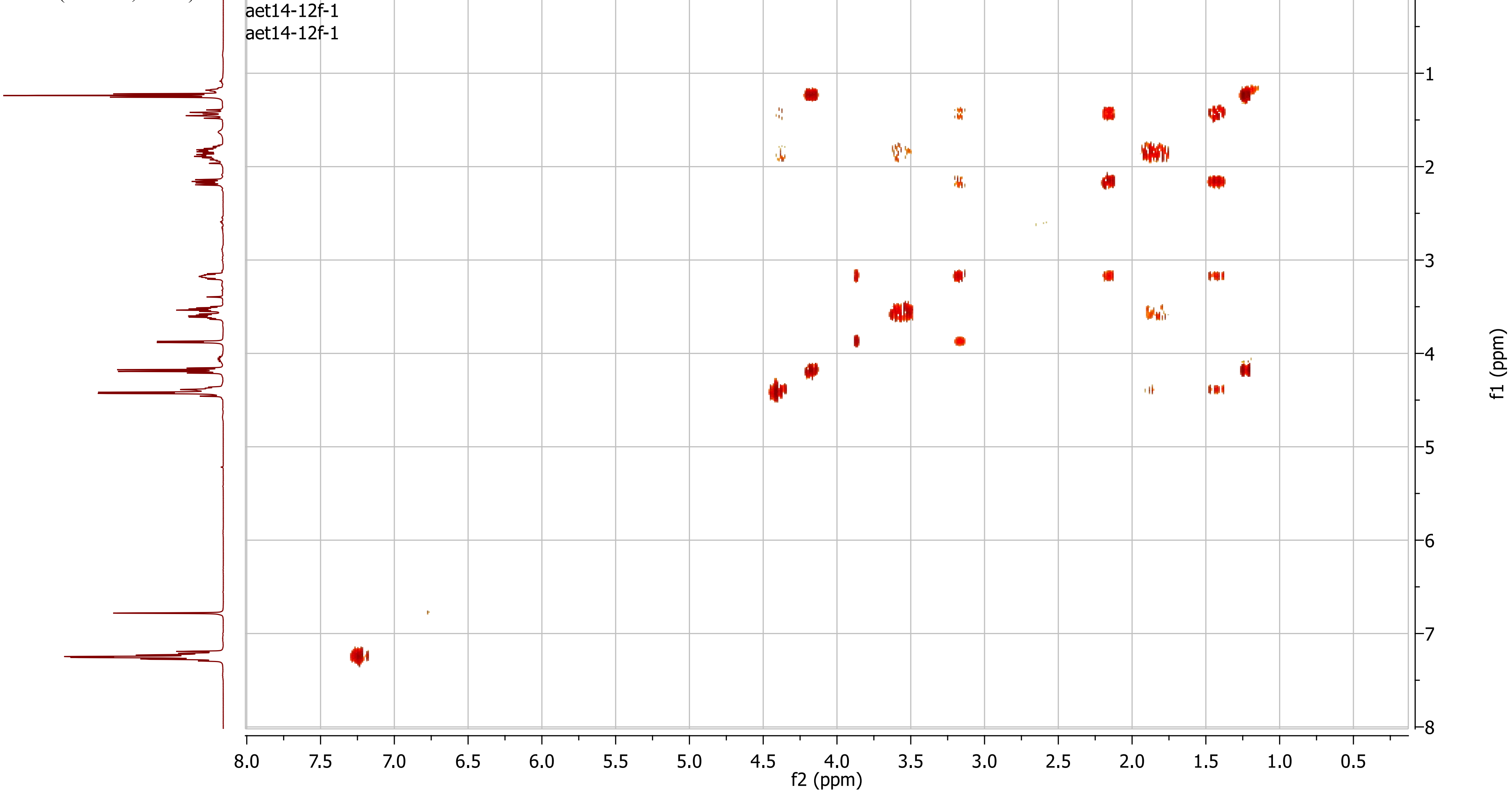

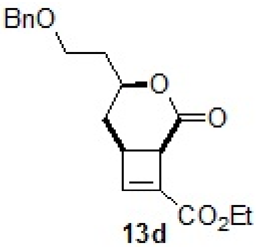

13d

HMQC (CDCl3)

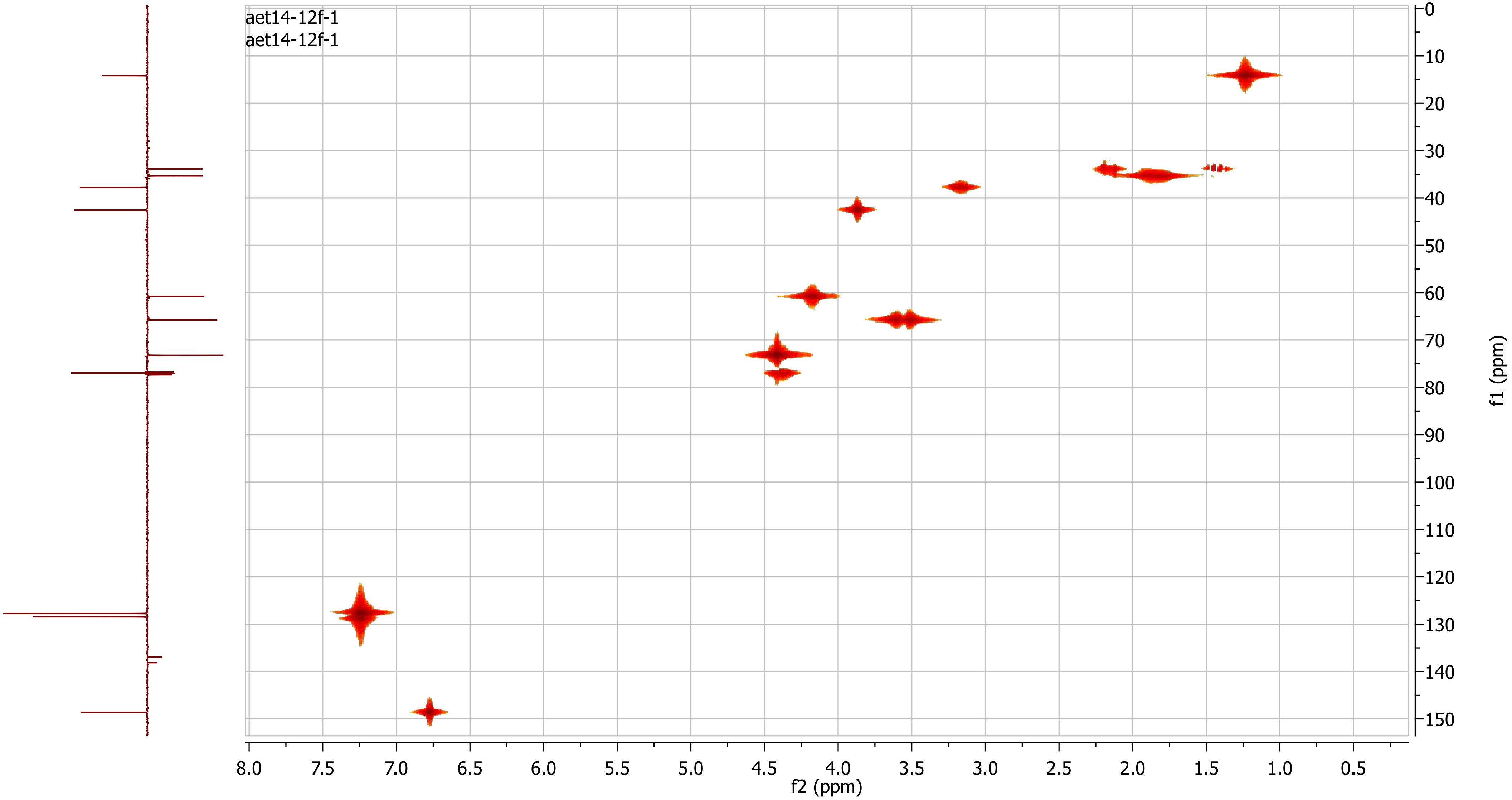

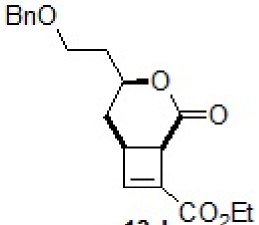

13d

NOESY (400 MHz, $CDCl_3$)

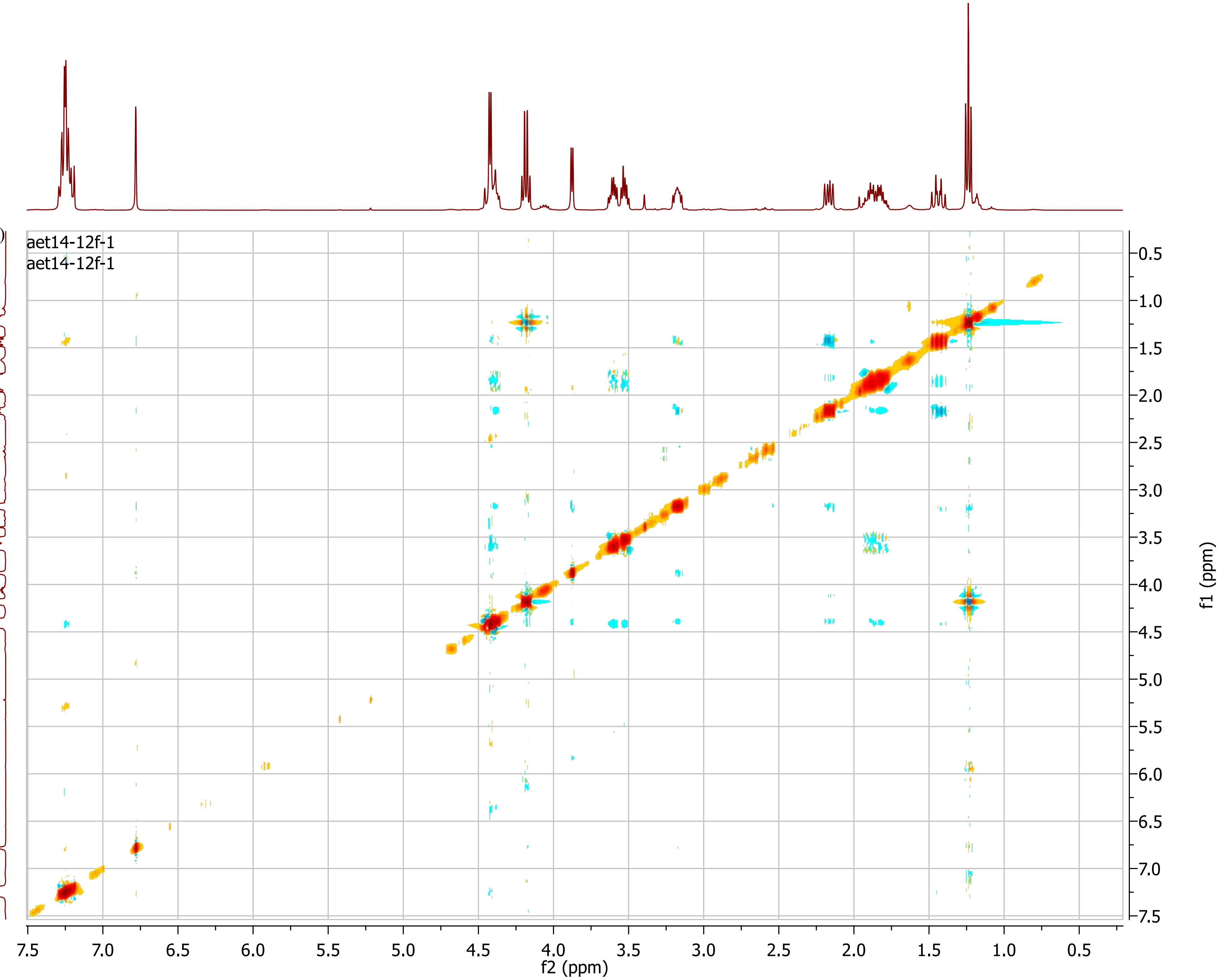

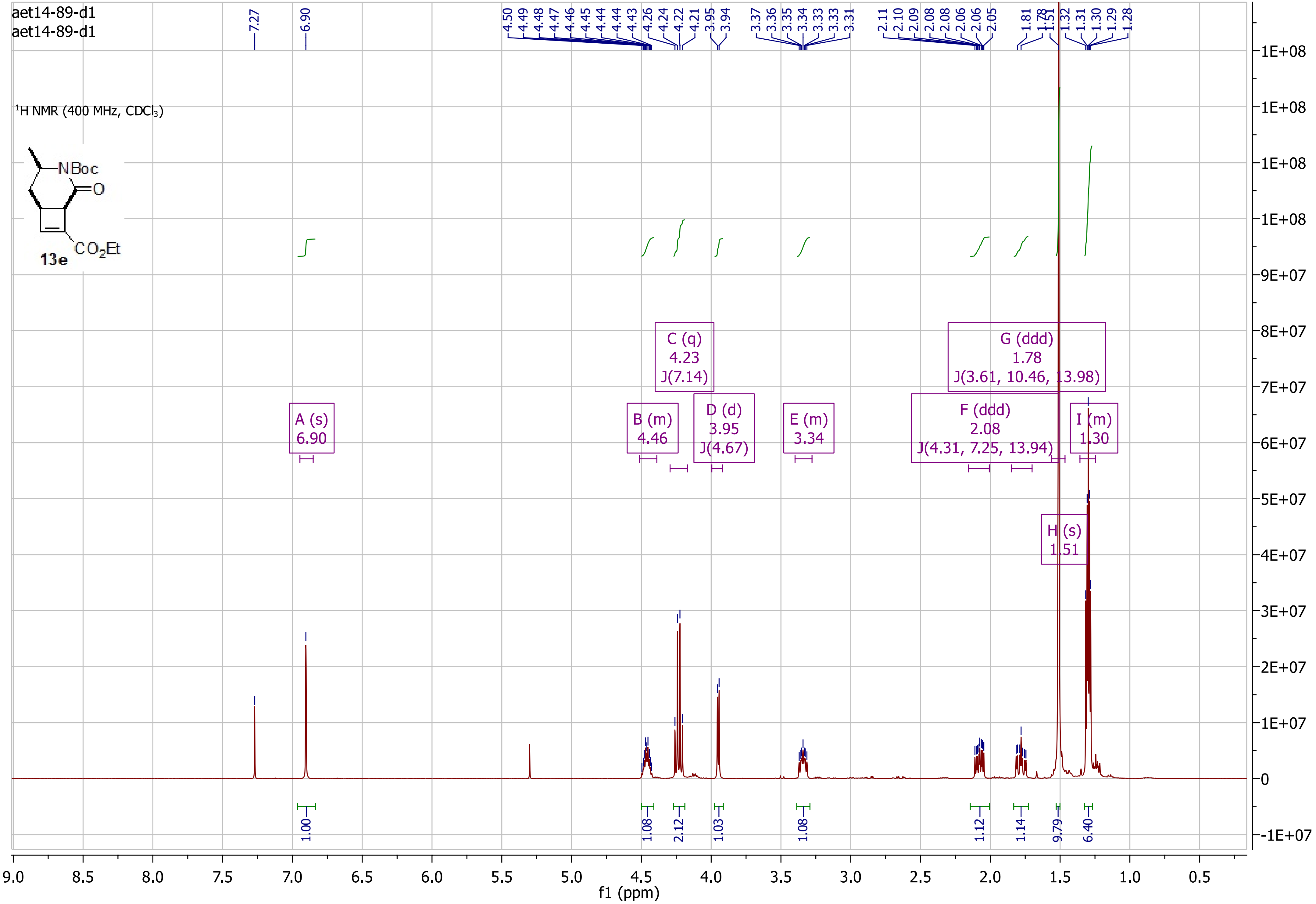
aet14-89-d1
aet14-89-d1
1H NMR (400 MHz, CDCl3)
NBoc
O
CO2Et
13e
7.27
6.90
4.50 4.49 4.48 4.47 4.46 4.45 4.44 4.44 4.43 4.26 4.24 4.22 4.21 3.95 3.94
3.37 3.36 3.35 3.34 3.33 3.33 3.31
2.11 2.10 2.09 2.08 2.08 2.06 2.06 2.05
1.81 1.78 1.51 1.32 1.31 1.30 1.29 1.28
A (s) 6.90
B (m) 4.46
C (q) 4.23 J(7.14)
D (d) 3.95 J(4.67)
E (m) 3.34
F (ddd) 2.08 J(4.31, 7.25, 13.94)
G (ddd) 1.78 J(3.61, 10.46, 13.98)
H (s) 1.51
I (m) 1.30
1.00 1.08 2.12 1.03 1.08 1.12 1.14 9.79 6.40
f1 (ppm)

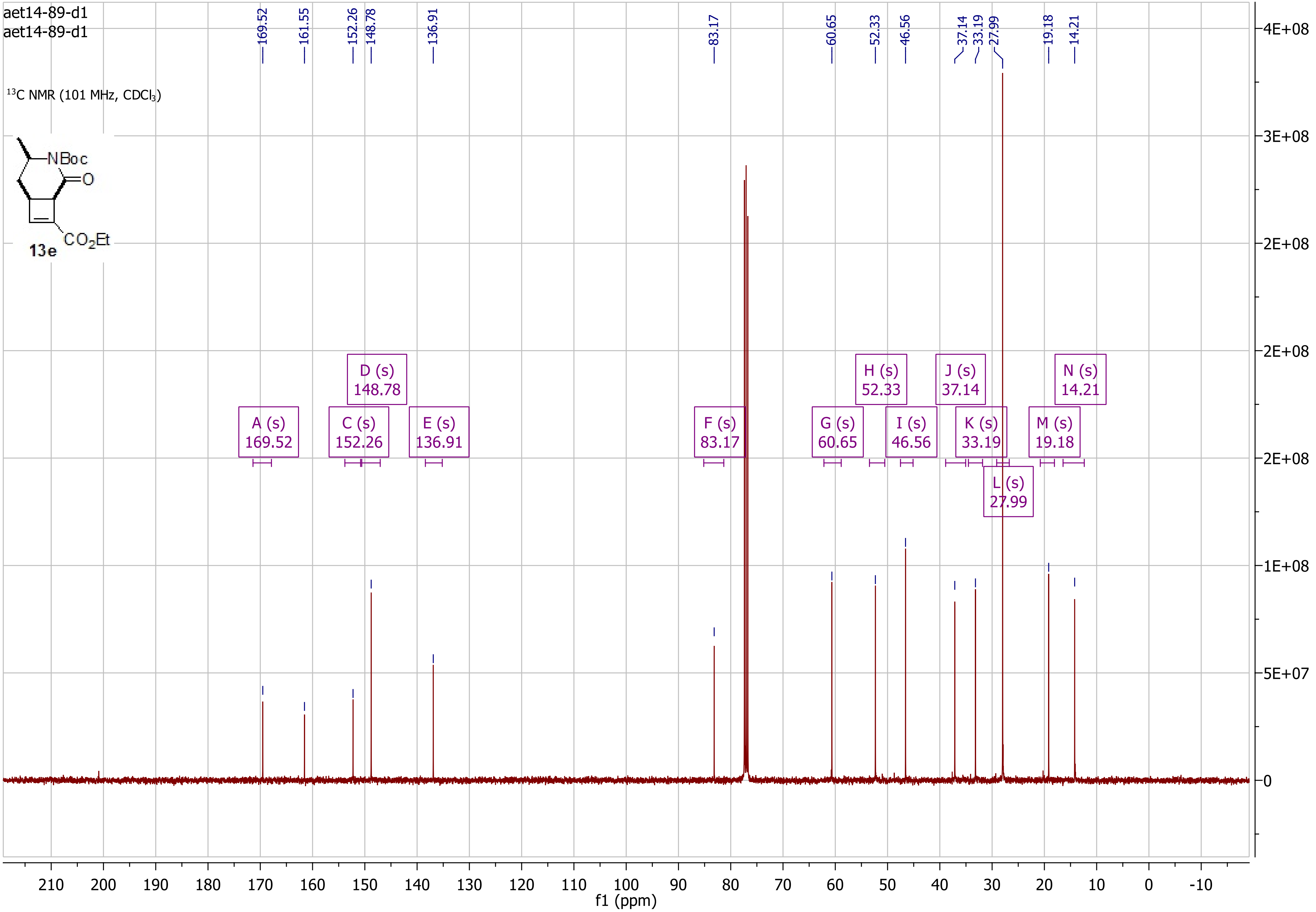

aet14-89-d1
aet14-89-d1
13C NMR (101 MHz, CDCl3)
NBoc
O
CO2Et
13e
169.52
161.55
152.26
148.78
136.91
83.17
60.65
52.33
46.56
37.14
33.19
27.99
19.18
14.21
A (s) 169.52
C (s) 152.26
D (s) 148.78
E (s) 136.91
F (s) 83.17
G (s) 60.65
H (s) 52.33
I (s) 46.56
J (s) 37.14
K (s) 33.19
L (s) 27.99
M (s) 19.18
N (s) 14.21
4E+08
3E+08
2E+08
2E+08
2E+08
1E+08
5E+07
0
210 200 190 180 170 160 150 140 130 120 110 100 90 80 70 60 50 40 30 20 10 0 -10
f1 (ppm)

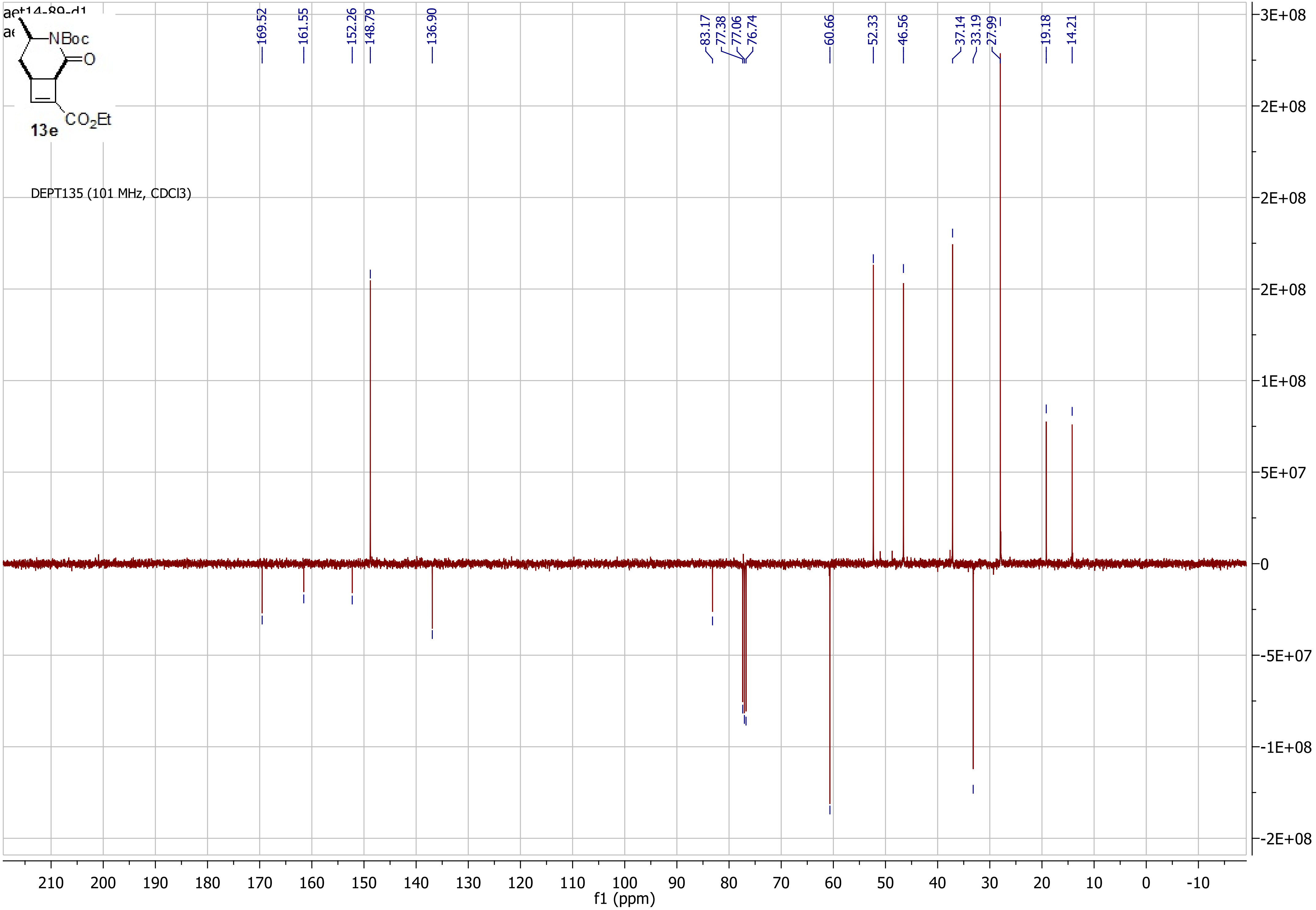
NBoc
O
CO2Et
13e
DEPT135 (101 MHz, CDCl3)
169.52
161.55
152.26
148.79
136.90
83.17
77.38
77.06
76.74
60.66
52.33
46.56
37.14
33.19
27.99
19.18
14.21
f1 (ppm)

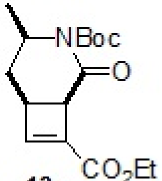

13e

COSY (400 MHz, CDCl3)

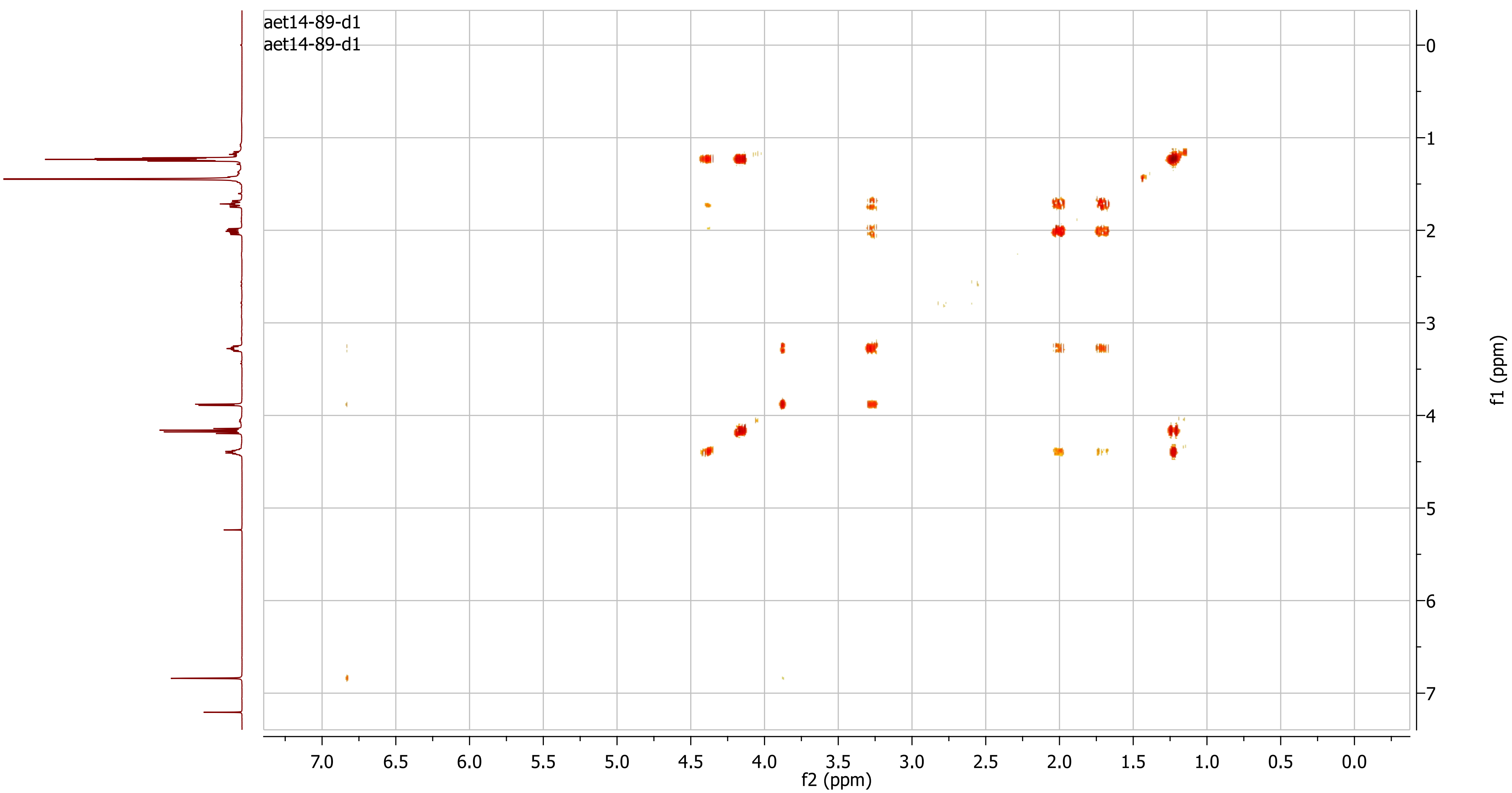

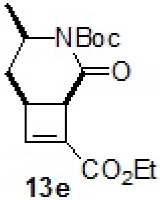

13e

COSY (400 MHz, $CDCl_3$)

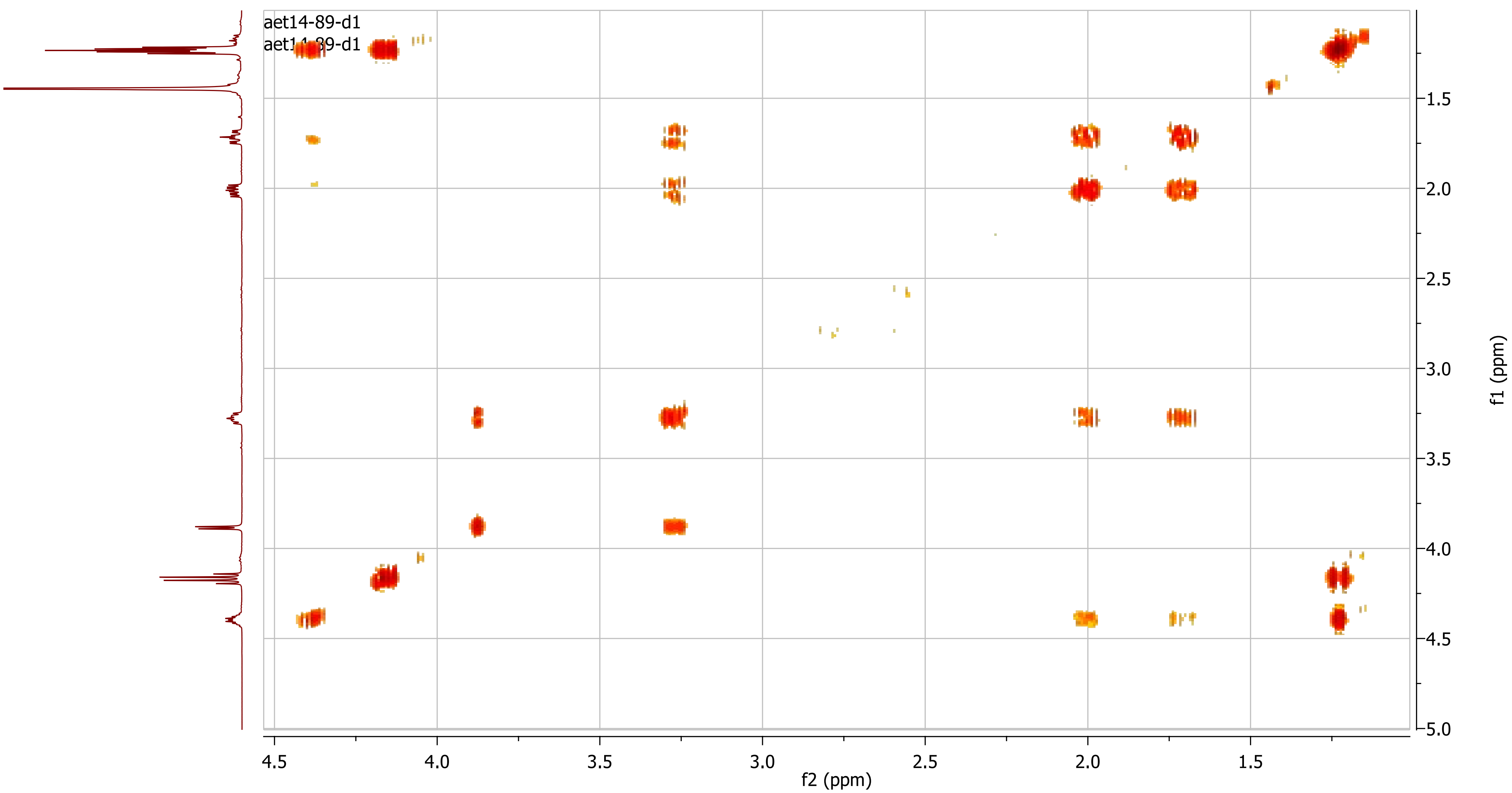

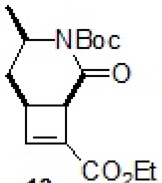

13e

HMQC (CDCL3)

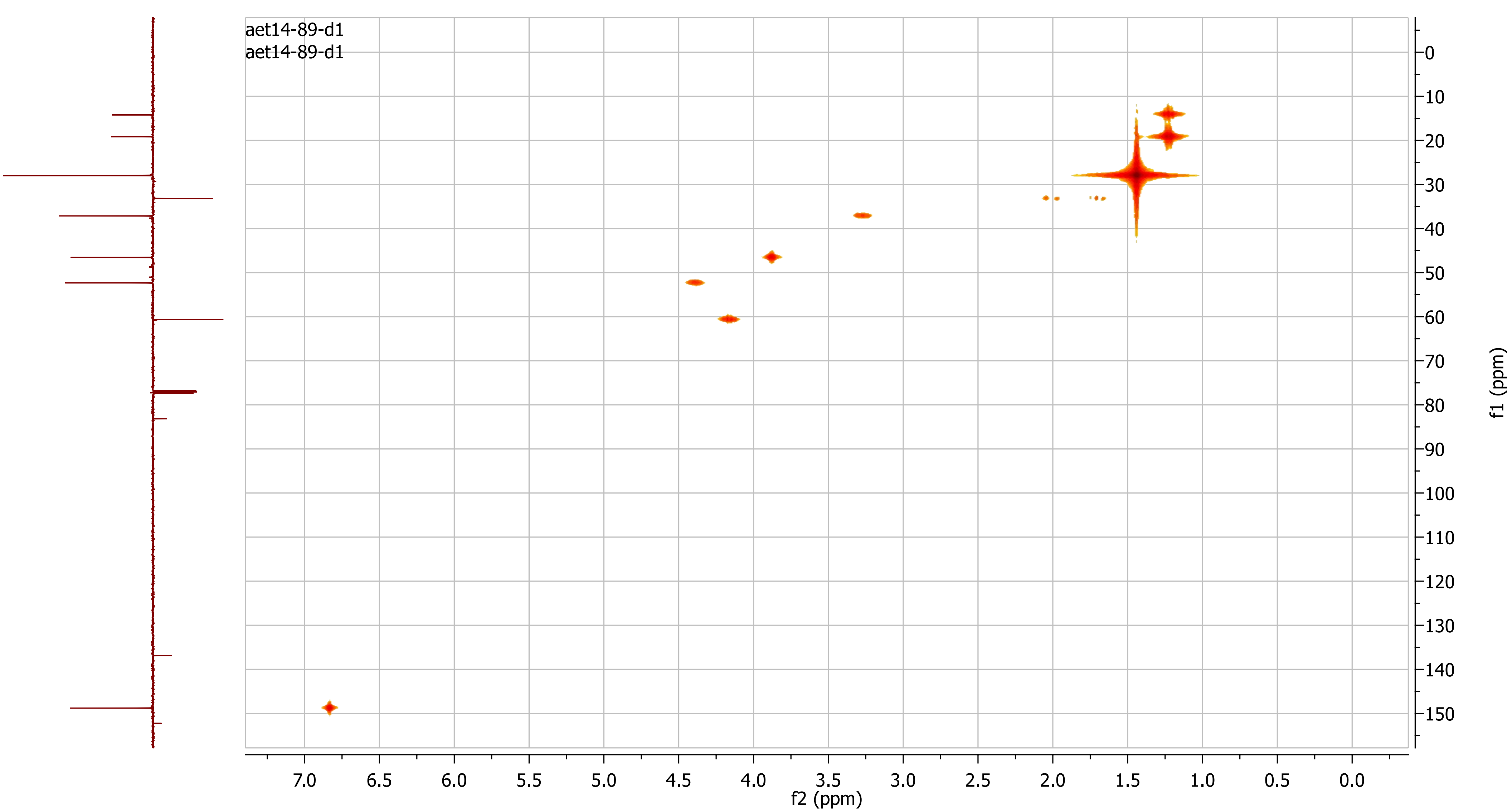

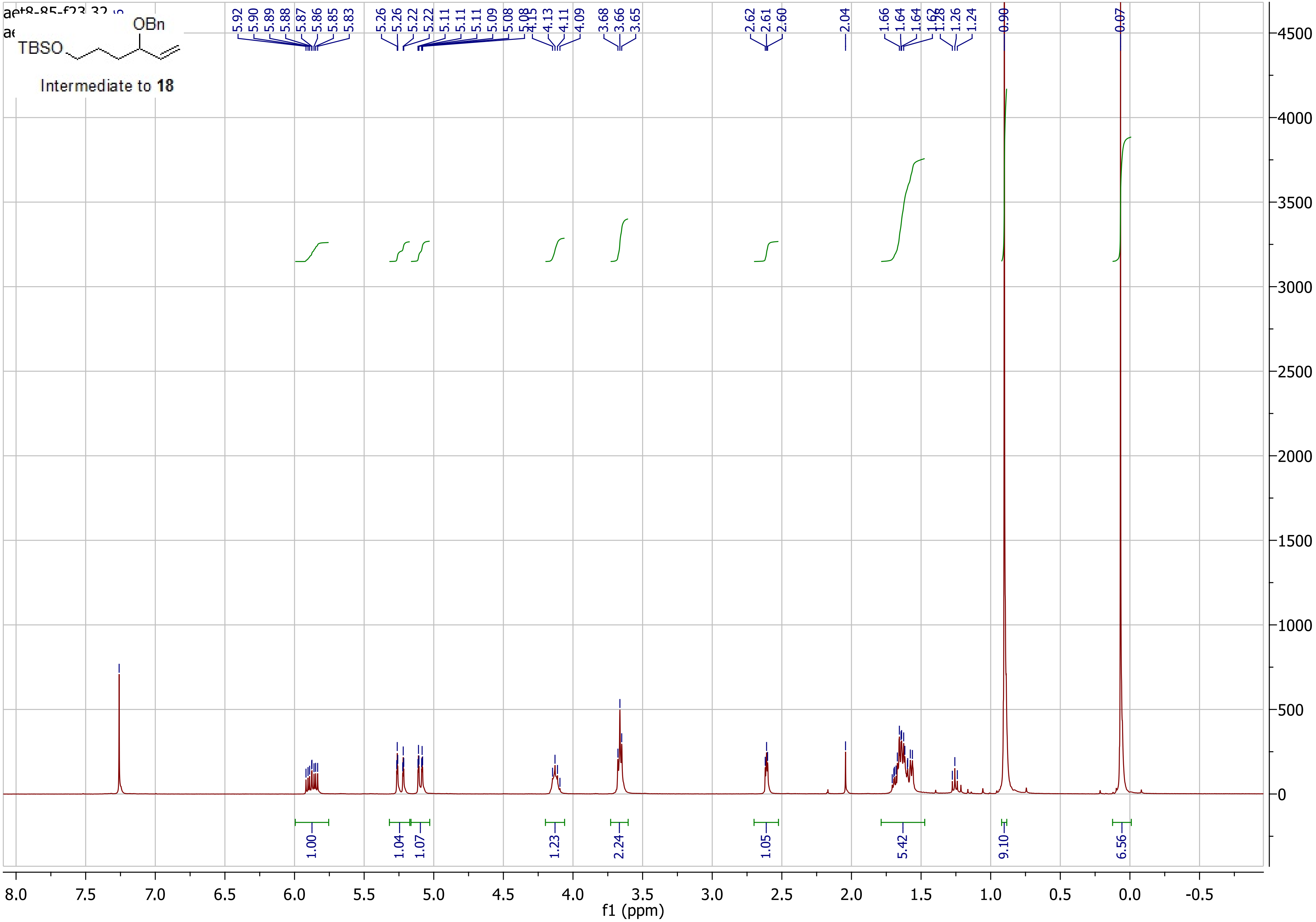

TBSO
OBn
Intermediate to 18
5.92 5.90 5.89 5.88 5.87 5.86 5.85 5.83 5.26 5.26 5.22 5.22 5.11 5.11 5.11 5.09 5.08 4.15 4.13 4.11 4.09 3.68 3.66 3.65 2.62 2.61 2.60 2.04 1.66 1.64 1.64 1.62 1.28 1.26 1.24 0.90 0.07
1.00 1.04 1.07 1.23 2.24 1.05 5.42 9.10 6.56
f1 (ppm)

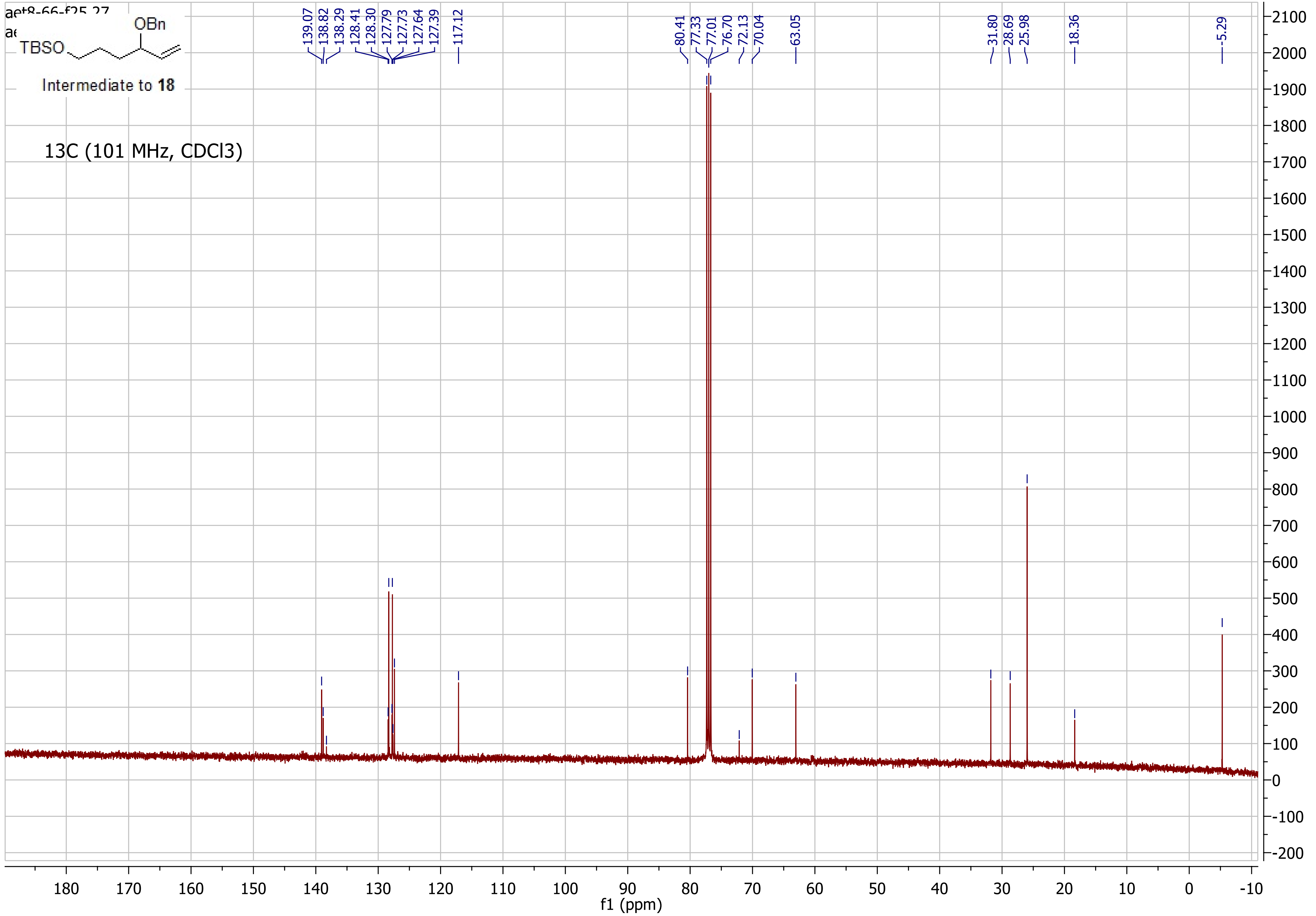

OBn
TBSO
Intermediate to 18
13C (101 MHz, CDCl3)
139.07
138.82
138.29
128.41
128.30
127.79
127.73
127.64
127.39
117.12
80.41
77.33
77.01
76.70
72.13
70.04
63.05
31.80
28.69
25.98
18.36
-5.29
f1 (ppm)

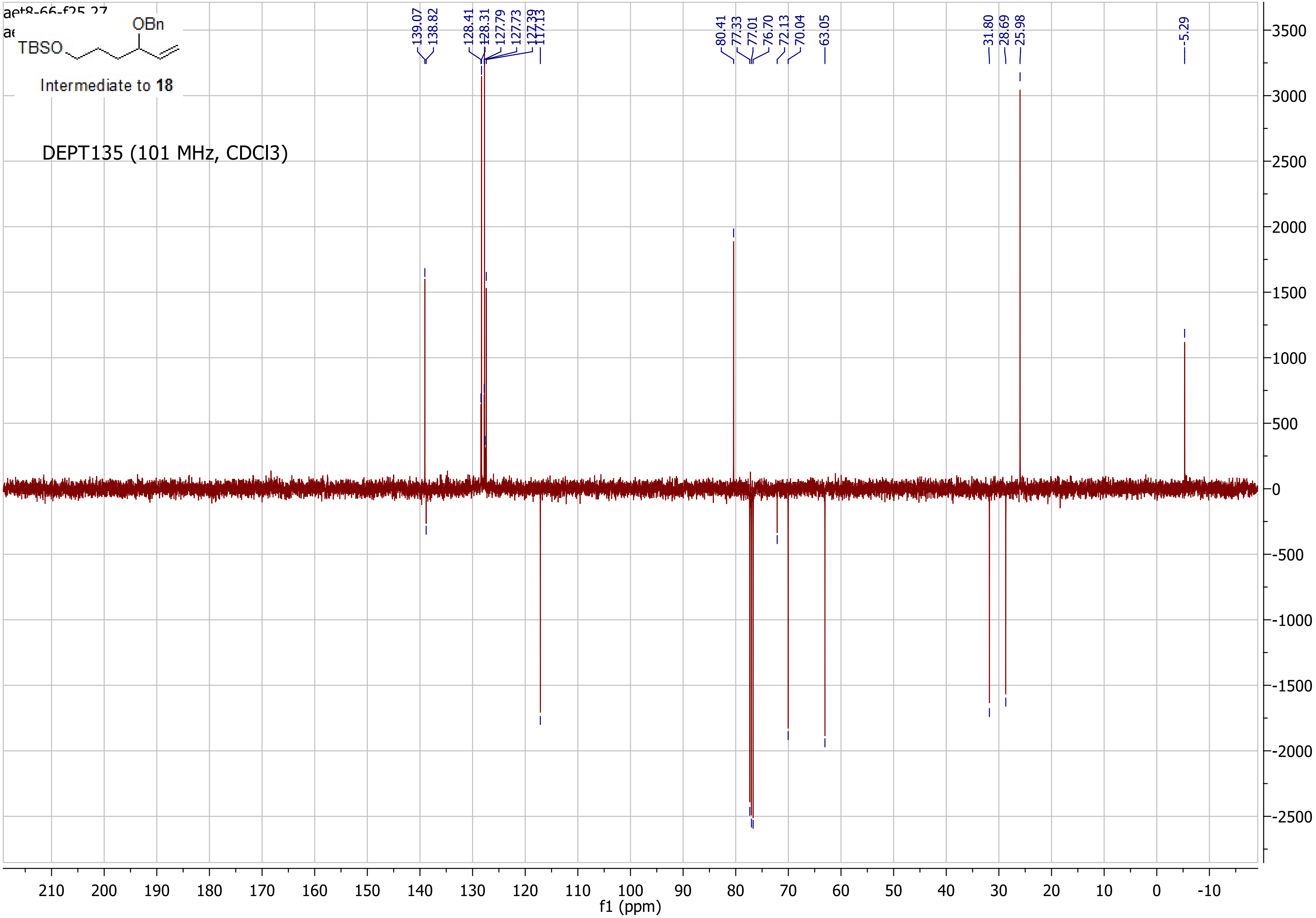

aet8-66-f25.27
OBn
TBSO
Intermediate to 18
DEPT135 (101 MHz, CDCl3)
139.07
138.82
128.41
128.31
127.79
127.73
127.39
117.13
80.41
77.33
77.01
76.70
72.13
70.04
63.05
31.80
28.69
25.98
-5.29
f1 (ppm)

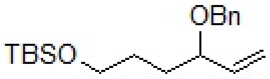

Intermediate to **18**

COSY (400 MHz, CDCl3)

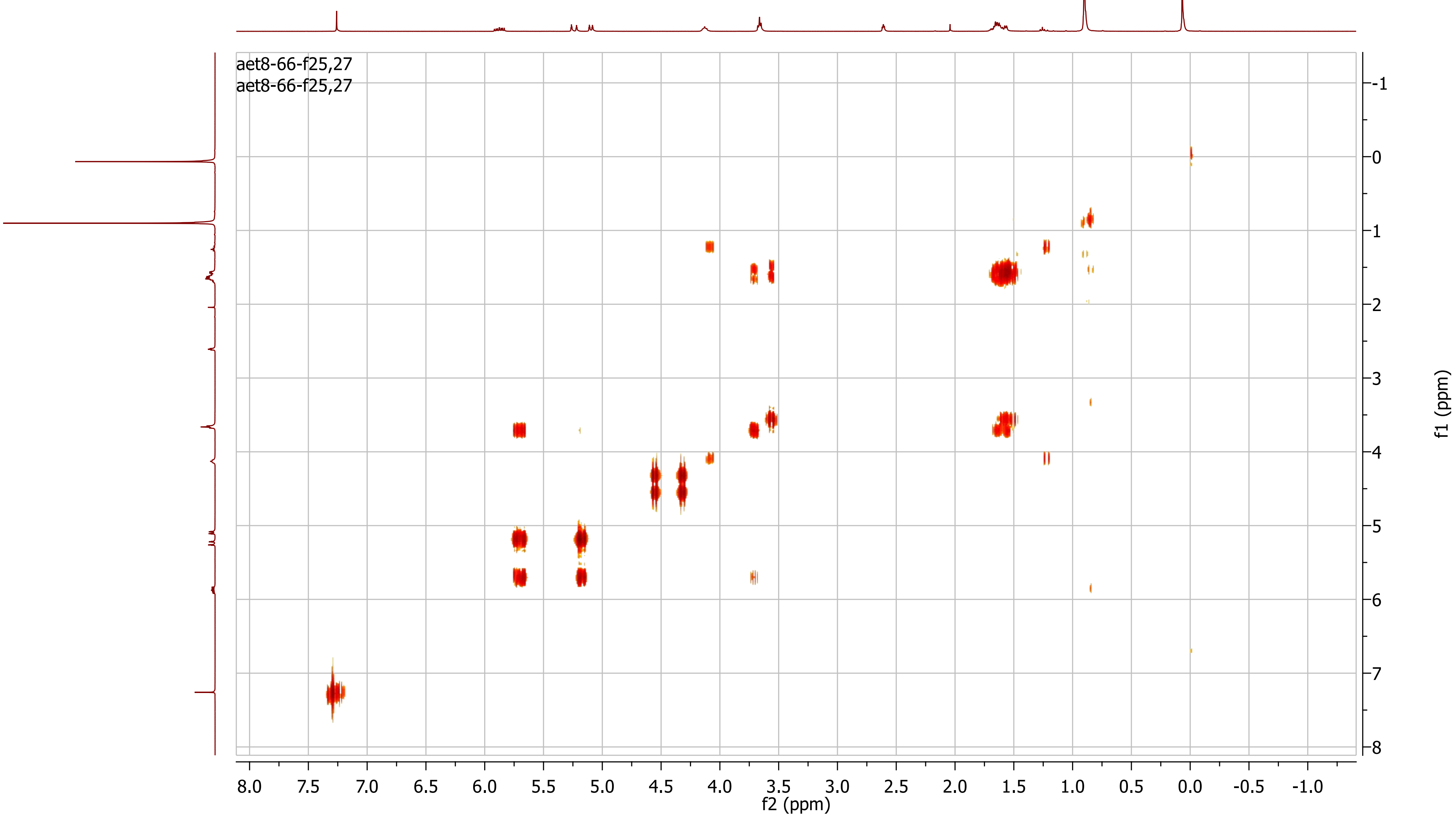

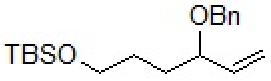

Intermediate to **18**

HMQC (CDCl3)

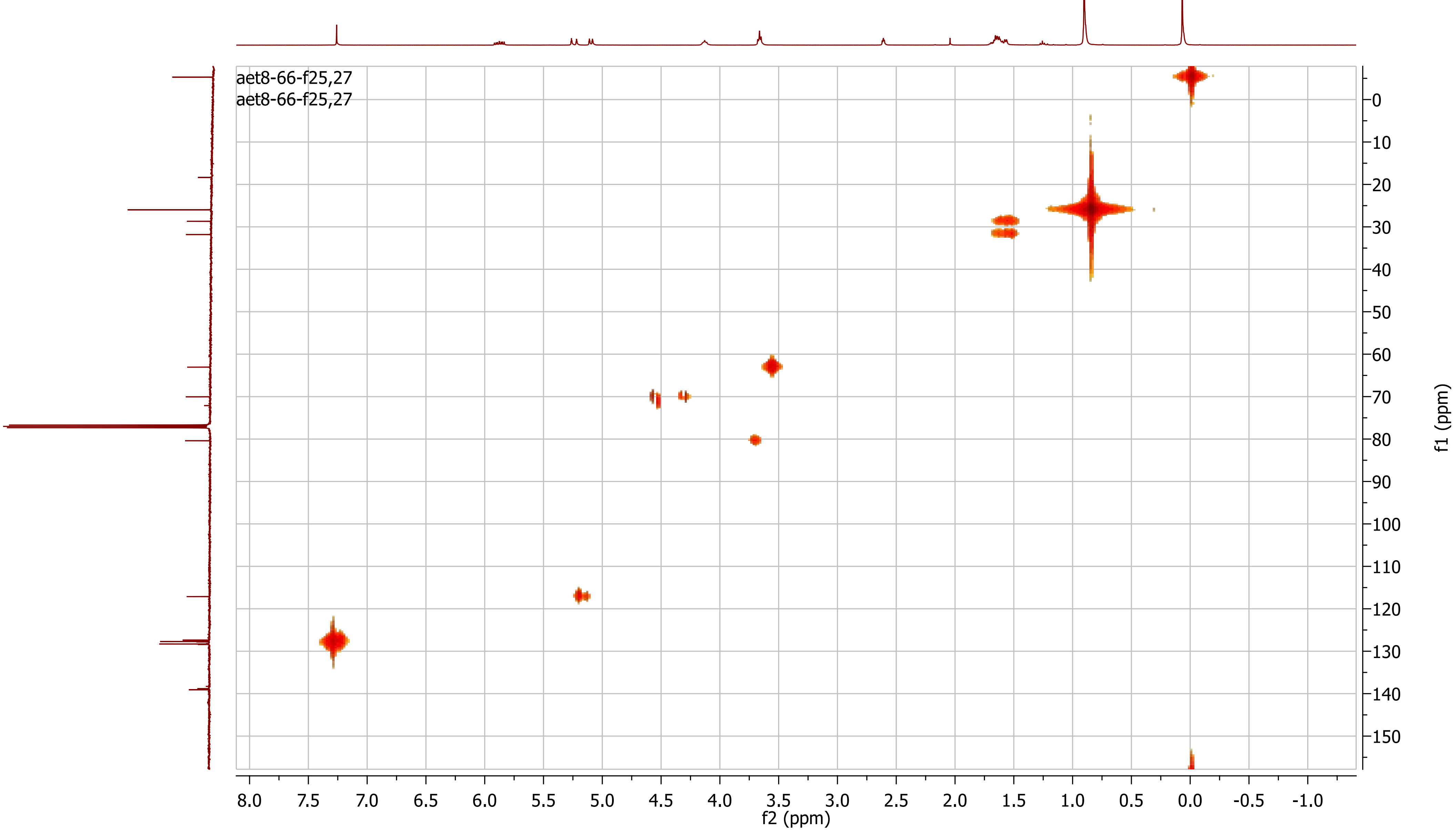

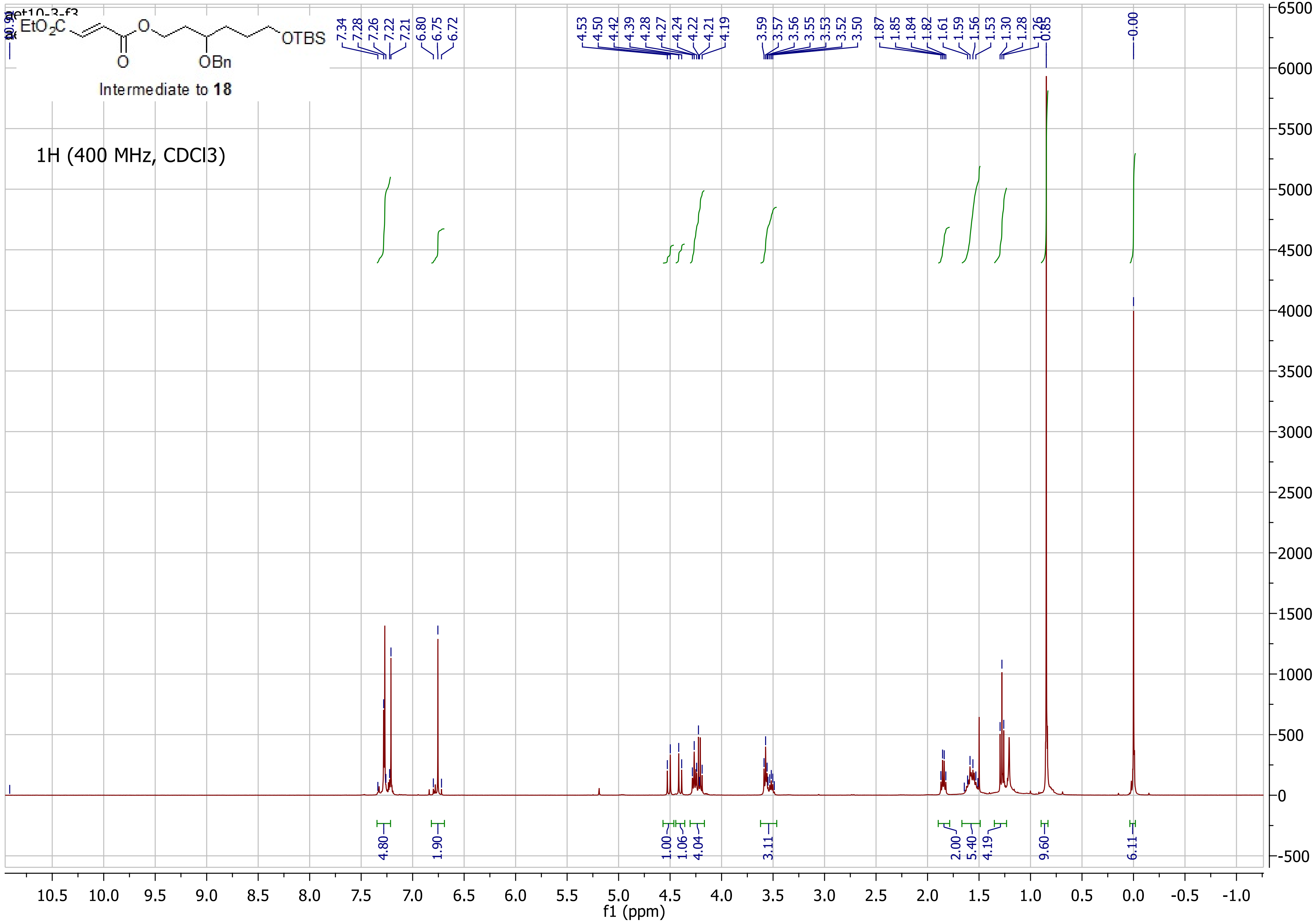

EtO2C
O
OBn
OTBS
Intermediate to 18
1H (400 MHz, CDCl3)
7.34 7.28 7.26 7.22 7.21 6.80 6.75 6.72
4.53 4.50 4.42 4.39 4.28 4.27 4.24 4.22 4.21 4.19
3.59 3.57 3.56 3.55 3.53 3.52 3.50
1.87 1.85 1.84 1.82 1.61 1.59 1.56 1.53 1.30 1.28 1.26 0.85
-0.00
4.80 1.90 1.00 1.06 4.04 3.11 2.00 5.40 4.19 9.60 6.11
f1 (ppm)

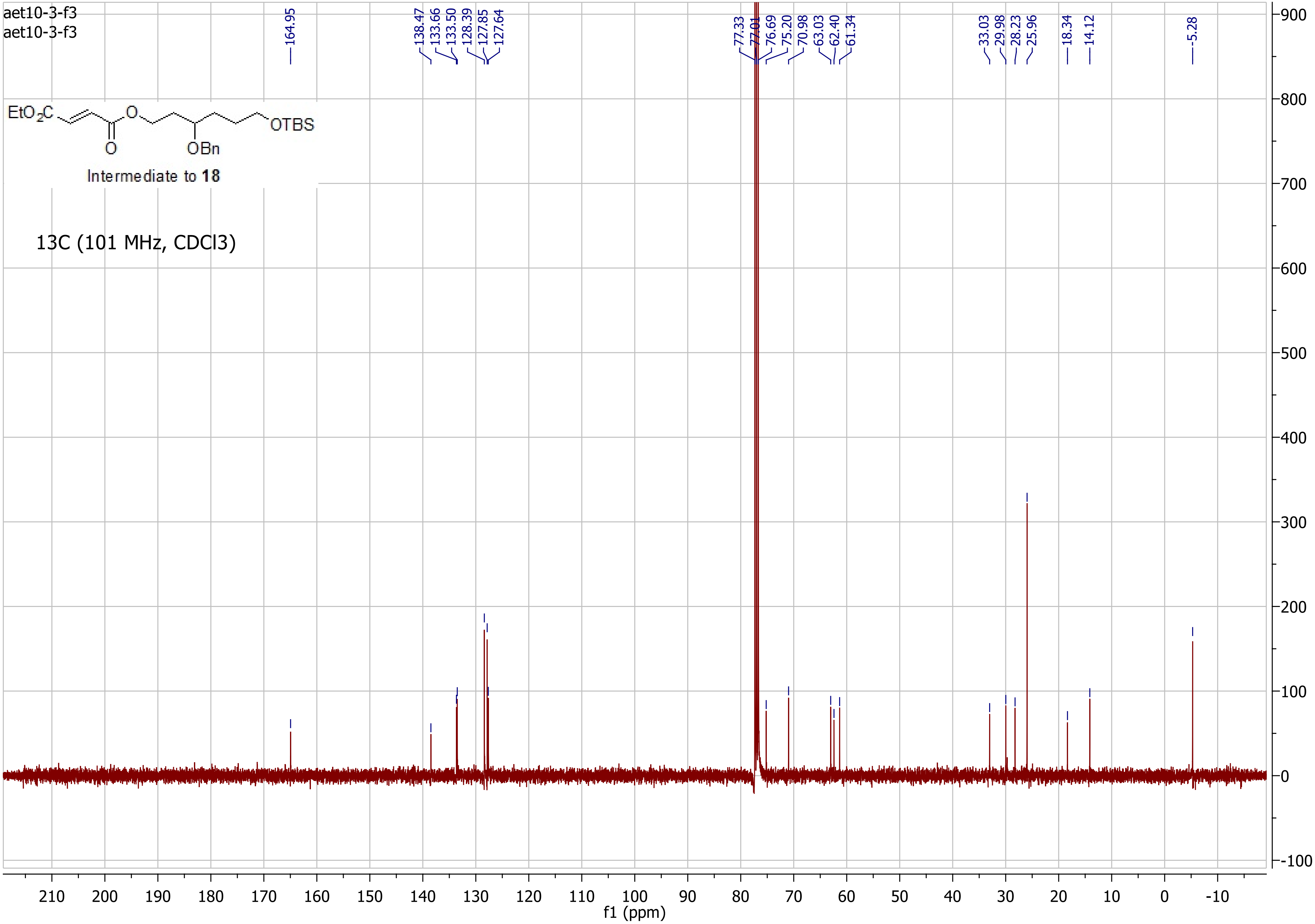

aet10-3-f3
aet10-3-f3
164.95
138.47
133.66
133.50
128.39
127.85
127.64
77.33
77.01
76.69
75.20
70.98
63.03
62.40
61.34
33.03
29.98
28.23
25.96
18.34
14.12
-5.28
EtO2C
O
O
OBn
OTBS
Intermediate to 18
13C (101 MHz, CDCl3)
f1 (ppm)

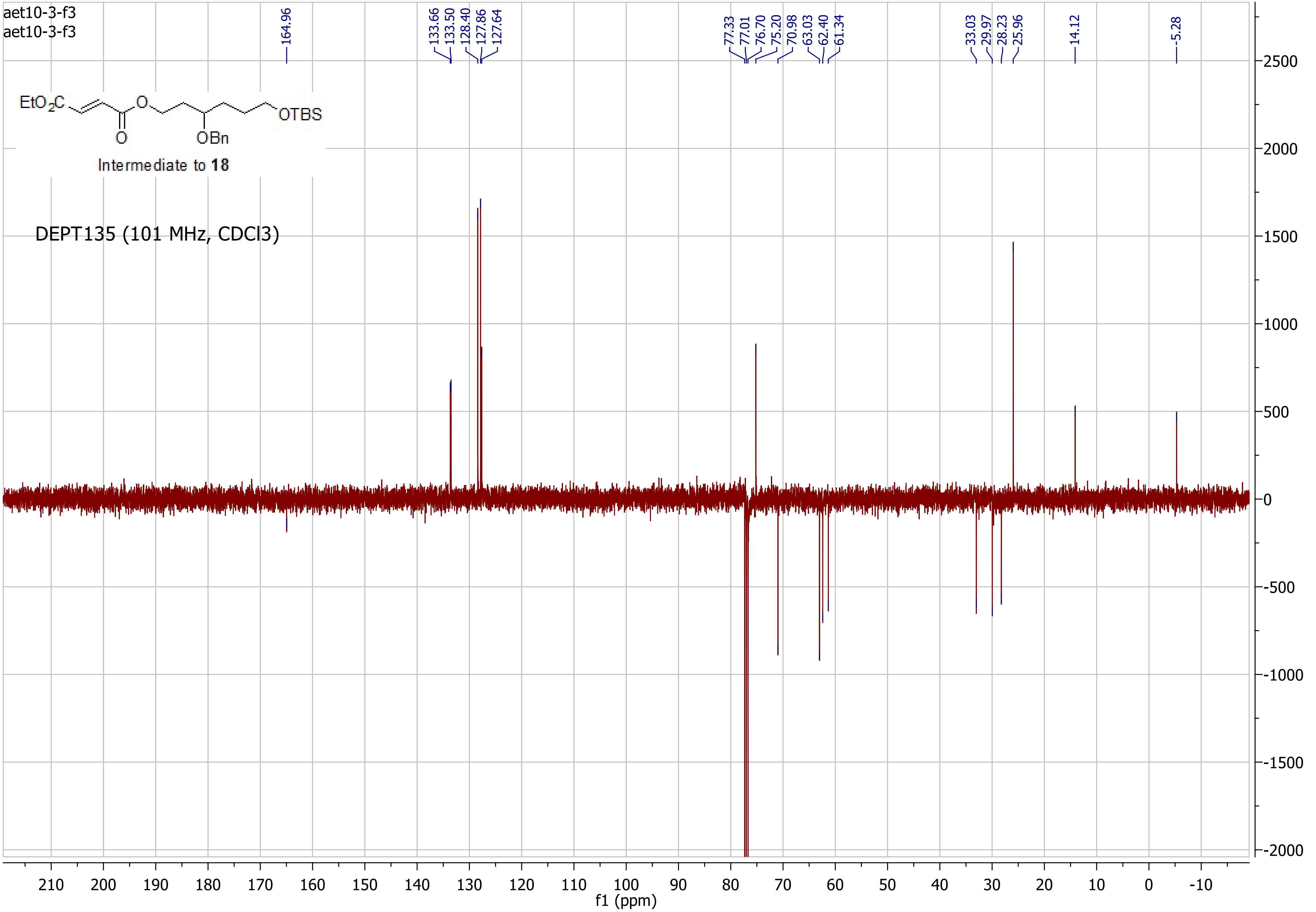
aet10-3-f3
aet10-3-f3
164.96
133.66
133.50
128.40
127.86
127.64
77.33
77.01
76.70
75.20
70.98
63.03
62.40
61.34
33.03
29.97
28.23
25.96
14.12
-5.28
EtO2C
O
O
OBn
OTBS
Intermediate to 18
DEPT135 (101 MHz, CDCl3)
f1 (ppm)
2500
2000
1500
1000
500
0
-500
-1000
-1500
-2000
210
200
190
180
170
160
150
140
130
120
110
100
90
80
70
60
50
40
30
20
10
0
-10

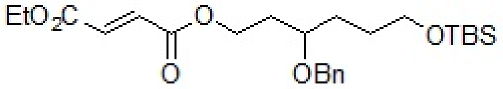

Intermediate to **18**

COSY (400 MHz, CDCl3)

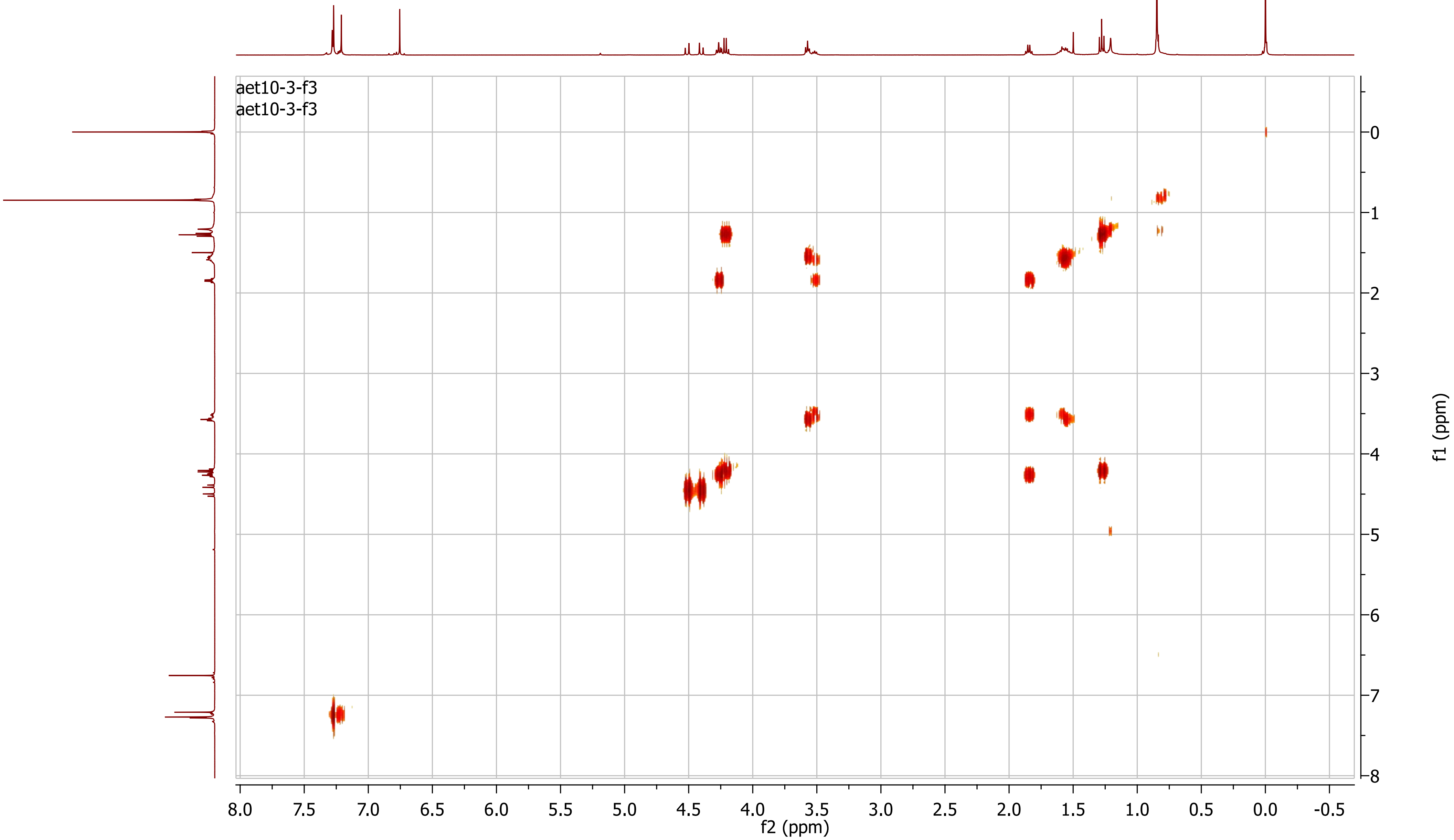

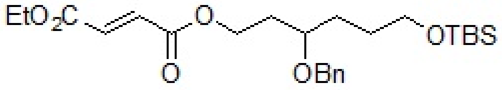

Intermediate to **18**

HMQC (CDCl3)

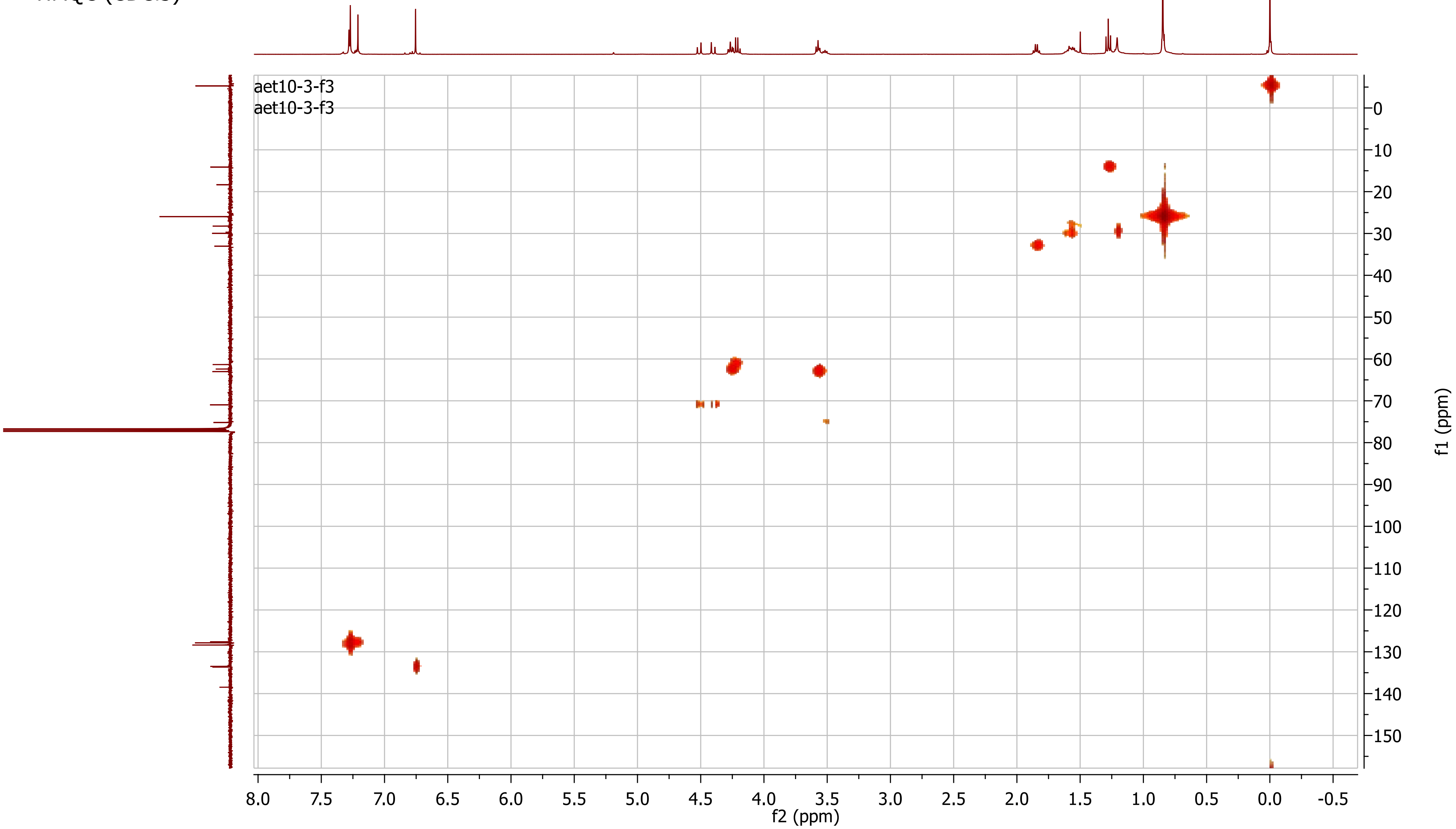

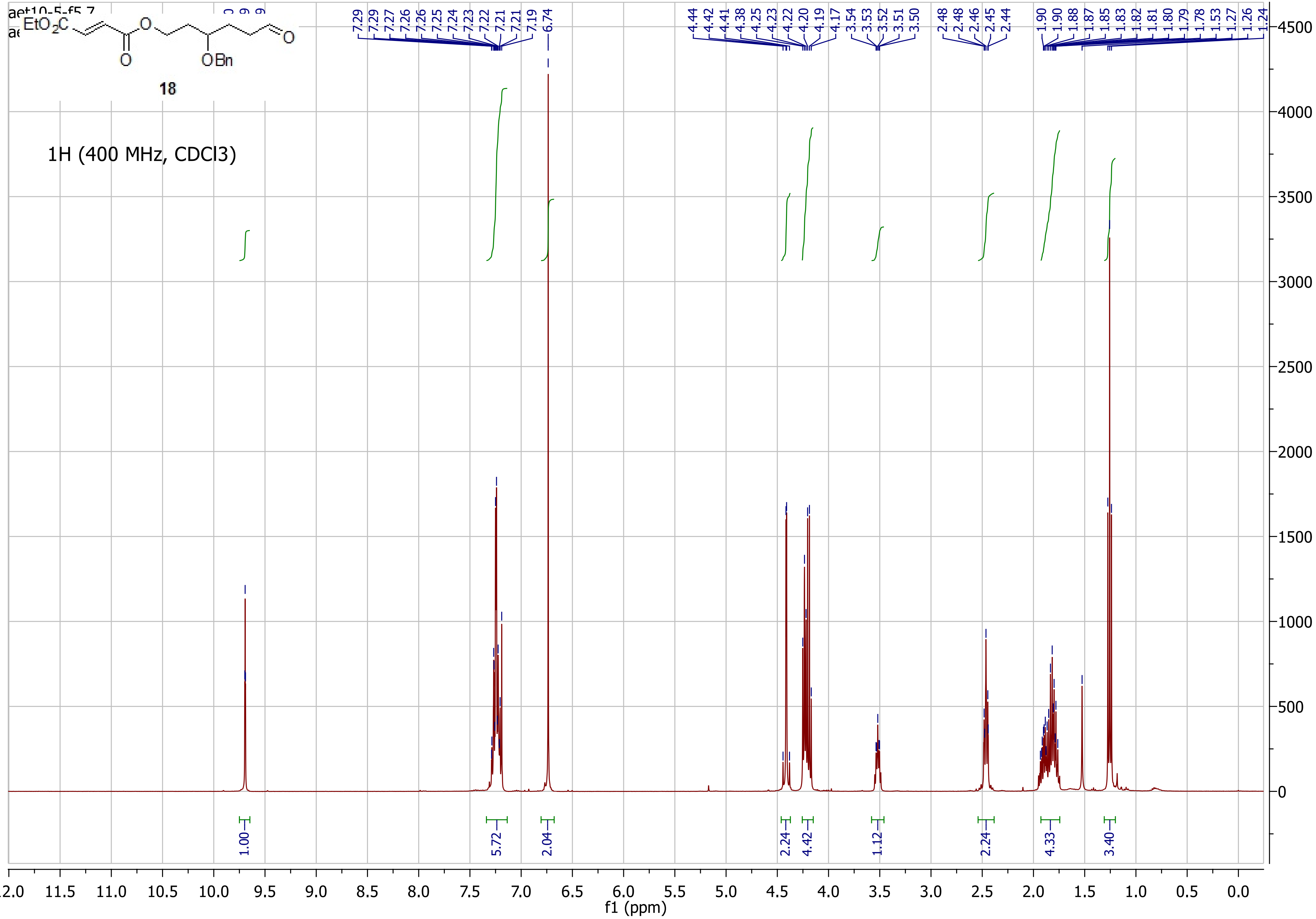

1H (400 MHz, CDCl3)
18
f1 (ppm)
7.29
7.29
7.27
7.26
7.26
7.25
7.24
7.23
7.22
7.21
7.21
7.19
6.74
4.44
4.42
4.41
4.38
4.25
4.23
4.22
4.20
4.19
4.17
3.54
3.53
3.52
3.51
3.50
2.48
2.48
2.46
2.45
2.44
1.90
1.90
1.88
1.87
1.85
1.83
1.82
1.81
1.80
1.79
1.78
1.53
1.27
1.26
1.24
1.00
5.72
2.04
2.24
4.42
1.12
2.24
4.33
3.40

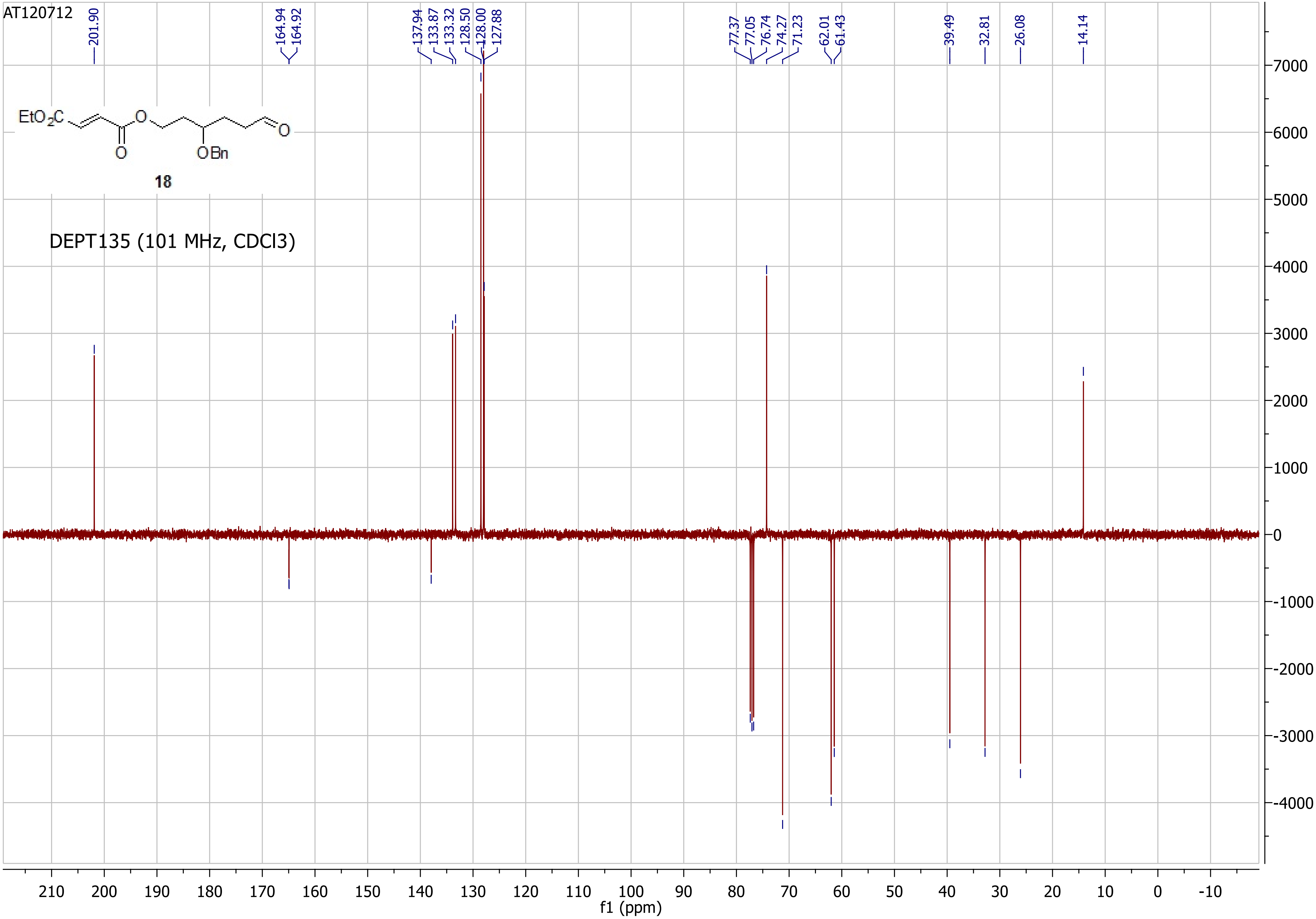
AT120712
201.90
164.94
164.92
137.94
133.87
133.32
128.50
128.00
127.88
77.37
77.05
76.74
74.27
71.23
62.01
61.43
39.49
32.81
26.08
14.14
EtO2C
O
O
OBn
O
18
DEPT135 (101 MHz, CDCl3)
7000
6000
5000
4000
3000
2000
1000
0
-1000
-2000
-3000
-4000
210
200
190
180
170
160
150
140
130
120
110
100
90
80
70
60
50
40
30
20
10
0
-10
f1 (ppm)
